# Supplementary material for: Two Sides of the Same Coin for Health: Adaptogenic Botanicals as Nutraceuticals for Nutrition and Pharmaceuticals in Medicine
Source: Pharmaceuticals (Basel). 2025 Sep 8;18(9):1346. doi: 10.3390/ph18091346 (PMC12472958; doi:10.3390/ph18091346)
Supplement: Supplementary file 1 [file pharmaceuticals-18-01346-s001.zip › Supplement S1_Hippocampus 25072017.pdf]

Report NCAG 15/17 H – Hippocampus slice preparation

1 Title Page

**1.**	**Study title**	**In-vitro Characterization of Different Extracts in the Hippocampus-slice Preparation of Rats.**
	**Study no.**	**NCAG 15/17 Hippocampus slice preparation**
**1.1**	**Method**	**Hippocampus Slice in vitro**

1.2 Names, addresses and telephone numbers persons of involved in the study:

Principal investigator
Prof. Dr. Wilfried Dimpfel
NeuroCode AG
Sportparkstr. 9
D-35578 Wetzlar
w.dimpfel@neurocode-ag.com
Phone +49 6441 2002033
Fax. +49 6441 2002039

Performance
Leonie Schombert
info@neurocode-ag.com
Phone +49 6441 2002030

Quality assurance
Ingrid K. Keplinger-Dimpfel
NeuroCode AG
Sportparkstr. 9
D-35578 Wetzlar
keplinger@neurocode-ag.com
Phone +49 6441 2002030
Fax. +49 6441 2002039

1.3 Sponsor
Terrence J. Lemerond
EuroPharma, Inc.
955 Challenger Drive, Green Bay,
Wisconsin 54311, USA

Prof. Dr. Alexander Panossian
Science and Research Director
ap.phytomedicine@gmail.com

1.4 Trial centre
Hippocampus Laboratory of
NeuroCode AG
Sportparkstr. 9
D-35578 Wetzlar
info@neurocode-ag.com
Phone +49 6441 2002030
Fax. +49 6441 2002039

Wetzlar, 25/07/2017

____
(Prof. Dr. Wilfried Dimpfel)

2 Content

1 Title Page **1**
2 Content **2**
3 Summary **3**
4 Introduction **5**
5 Materials and Methods **5**
6 Results **8**
6.1 Population spike analysis in the presence of Salidroside 8
6.2 Population spike analysis in the presence of Rosavin 9
6.3 Population spike analysis in the presence of RR-EUR-S 10
6.4 Population spike analysis in the presence of RR-Chi-R 11
6.5 Population spike analysis in the presence of RR-Chi-S 12
6.6 Population spike analysis in the presence of RR-Alt-S 13
6.7 Population spike analysis in the presence of RR-Alt-B 14
6.8 Population spike analysis in the presence of RR-Alt-X 15
6.9 Population spike analysis in the presence of RR-Alt-G 16
6.10 Population spike analysis in the presence of WS KSM66 17
6.11 Population spike analysis in the presence of Bryonia alba 19
6.12 Population spike analysis in the presence of the combination of RR-EUR-S and WS KSM66 23
6.13 Population spike analysis in the presence of a combination of RR-EUR-S and Bryonia alba 24
7 Discussion **26**
8 Literature **28**
9 Appendix **29**

NeuroCode AG
Sportparkstr. 9
D-35578 Wetzlar / Germany

EuroPharm, Inc
955 Challenger Drive, Green Bay
Wisconsin 54311, USA

Report NCAG 15/17 H – Hippocampus slice preparation

3 Summary

The hippocampus slice model in vitro was used to compare different Rhodiola extracts among each other with respect to pyramidal cell response to electric stimulation. Measurement of the population spike amplitude representing the number of responding cells in the presence of electric single shock stimulation as well as during theta burst stimulation served as parameter of effectiveness. Two chinese and four Rhodiola extracts from Altai were investigasted besides the two main ingredients Rosavin and Salidroside (s. table below). In addition Withania somnifera and Bryonia alba extract were investigated. Finally, these two extracts were mixed with Rhodiola extract in order to test for possible additive or potentiating effects. All extracts as well as Rosavin and Salidroside enhanced the amplitude of the population spikes in a concentration dependent manner from 0.25 to 30 mg/L. Only Withania extract enhanced the spike amplitude up to 1 mg/L, however had no effect at 2.5 and 5 mg/L and attenuated the signal somewhat at a considerable higher concentration of 10 mg/L. However, it is not excluded, that other extracts can also attenuate the signal in considerable higher concentrations, but this has not been tested, because such high dosages probably relate more to possible side effects. 10 mg/L would roughly correspond to a dose of 10 mg/kg in humans (according to our experience with several herbal extracts tested in humans and the hippocampus slice preparation). Results were fitted by a mathematical tangens hyperbolicus function, from which EC_{50} values were calculated. The sample „RR-EUR-S" gave the lowest values for the Rhodiola extracts with about 5 mg/L for both stimulation patterns. Corresponding dosage in humans would be about 5 mg/kg. For Rosavin and Salidroside an EC_{50} of about 0.5 mg/L was calculated. An overview on EC_{50} values in the presence of single shock stimulation (SS) and theta burst stimulation (TBS) is given in the following table.

	EC_{50} Values for SS	EC_{50} Values for TBS
RR-EUR-S	4.81	4.60
RR-Chi-R	14.60	19.54
RR-Chi-S	9.79	15.03
RR-Alt-S	8.89	7.73
RR-Alt-B	15.74	13.77
RR-Alt-X	10.68	14.42
RR-Alt-G	12.39	17.69
Rosavin	0.44	0.57
Salidroside	0.47	0.49
WS KSM66	0.50	0.57
Bryonia Alba	0.53	0.91
WS+RR	3.98	5.09
BA+RR	4.67	6.02

The EC_{50} values did not relate to the content of Rosavin or Salidroside except for the fact, that RR-EUR-S had the highest content and also provided the lowest EC_{50}. From this it might be concluded that also other ingredients contained in Rhodiola extracts are able to enhance the pyramidal cell response and

produce long term potentiation a lower concentration , which relates to better time and space dependent memory. Interestingly, extracts from Withania somnifera and Bryonia alba had 10-fold lower EC_{50} values. The combination of Rhodiola RR-EUR-S (5 mg/L) plus Bryonia alba extract (0.5 mg/L) did not show additive effects, whereas the combination of RR-EUR-S (5 mg/L) with Withania somnifera extract (0.5 mg/L) developed at least additive effects and should be followed in future experiments for the detection of possible potentiating effects.

4 Introduction

Behavior is governed by the interaction of numerous electrical circuits distributed over many parts of the brain. These circuits are activated or attenuated during cognitive and emotional activity. One region related to mental activity and memory is the hippocampus. Selection of one of the electric hippocampal circuits between Schaffer collaterals and the pyramidal cells allows for a very focussed electrophysiological analysis. The hippocampal slice preparation has been shown to be a validated model for direct analysis of interaction of substances with living neuronal tissue (Lynch and Schubert, 1980; Dingledine, 1984). Due to the preservation of the three dimensional structure of the hippocampal tissue substance effects on the excitability of pyramidal cells can be studied in a unique manner providing much more accurate and detailed analysis of effects than behavioral tests. In addition, using the in vitro model of the hippocampus slice allows for detection of the mechanism of action on a molecular level by trying to antagonize the measured effect by receptor selective antagonists within the glutamatergic system. This approach has been successfully applied during the investigation of Sideritis scardica extract in the past (Dimpfel et al., 2016a). This is not possible in behavioral experiments. The stimulation of Schaffer Collaterals leads to release of glutamate resulting in excitation of the postsynaptic pyramidal cells. The result of the electrical stimulation can be recorded as a so-called population spike (pop-spike). The amplitude of the resulting population spike represents the number of recruited pyramidal cells. The response of the pyramidal cells to electric stimulation in form of the amplitude of the population spike indicates activation (increase of the amplitude) or calming and sedating effects (attenuation of the amplitude). Of special interest is the response to theta burst stimulation resulting in so-called "long term potentiation" which relates to increase of time- and space-dependent memory.The advantage of the model not only consists in the possibility of recording in vitro during 8 hours but also to recognize the excitability of the system in order to test the actual physiological condition. It therefore provides an excellent model to characterize the action of a new therapeutical principle with respect to changes of brain function under strictly controlled laboratory conditions. Especially, the clear interpretation of physiological changes within the hippocampus with respect to cognitive activation and memory predestine this model for characterization of herbal extracts like Rhodiola rosea extract as published (Dimpfel et al., 2016b), where a concentration dependent increase of the amplitude of the population spike was reported. These results relate very well to clinical results obtained and published earlier (Dimpfel et al., 2014). An interesting result using this model was for example also the ability of memantine, a substance used in the treatment of dementia, to increase population spike amplitude in response to single stimuli and to increase long term potentiation (Dimpfel, 1995).

5 Materials and Methods

Hippocampus slices were obtained from 48 adult male Sprague-Dawlay rats (Charles River Wiga, Sulzbach, Germany). Rats were kept under a reversed day/night cycle for 2 weeks prior start of the experiments to allow recording of in vitro activity from slices during the active phase of their circadian rhythm (Dimpfel et al., 1994). Animals were exsanguinated under ether anaesthesia, the brain was removed in total and the hippocampal formation was isolated under microstereoscopic sight. The midsection of the hippocampus was fixed to the table of a vibrating microtome (Rhema Labortechnik, Hofheim, Germany) using a cyanoacrylate adhesive, submerged in chilled bicarbonate-buffered saline (artificial cerebrospinal fluid (ACSF)): NaCl: 124 mM, KCl: 5 mM, CaCl2: 2

mM, MgSO4: 2 mM, NaHCO3: 26 mM, glucose: 10 mM, and cut into slices of 400 µm thickness. All slices were pre-incubated for at least 1 h in Carbogen saturated ACSF (pH 7.4) in a pre-chamber before use (Dimpfel et al., 1991).

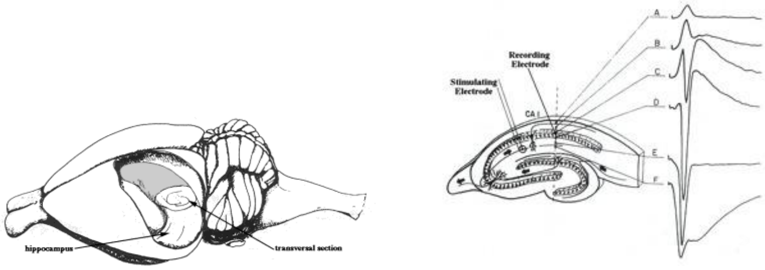

Fig. 1 Anatomical view of the position of the hippocampus in the rat. Direction of the cut of slices is depicted. Documentation of signals as obtained from different recording places of transversal section. In the present study recording was performed from the location "D".

During the experiment the slices were held and treated in a special superfusion chamber (List Electronics, Darmstadt, Germany) according to Haas et al., 1979 at 35°C (Schiff and Somjen, 1985). The preparation was superfused with artificial cerebrospinal fluid (ACSF) at 180-230 ml/h of the sample containing preparation at the indicated concentration.. Electrical stimulation (200 µA constant current pulses of 200 µs pulse width) of the Schaffer Collaterals within the CA2 area and recording of extracellular field potentials from the pyramidal cell layer of CA1 (Dimpfel et al., 1991) as shown in Fig. 1 was performed according to conventional electrophysiological methods using the "Labteam" Computer system "NeuroTool" software package (MediSyst GmbH, Linden, Germany). Measurements were performed at 10 min intervals to avoid potentiation mechanisms. Four stimulations – each 20 s apart – were averaged for each time point. After obtaining three stable responses to single stimuli (SS) long term potentiation was induced by applying a theta burst type pattern (TBS). The mean amplitudes of three signals were averaged to give the mean of absolute voltage values (Microvolt) ± standard error of the mean for four slices representing one of the experimental conditions (Fig. 2). Four slices were used per day.

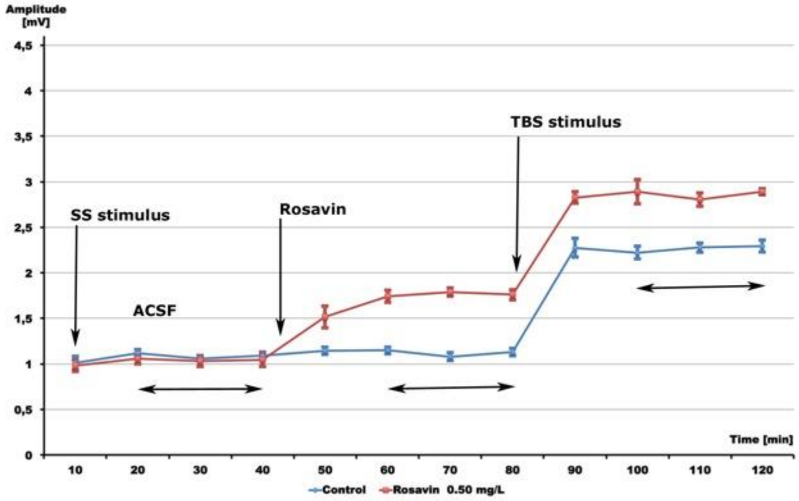

Fig. 2 Time line of experimental recording. Example of 0.5 mg/L of Rosavin in comparison to control (ACSF). ← → : averaged to give the mean value (s. text above).

Report NCAG 15/17 H – Hippocampus slice preparation

Test sample	Description	Rosavins content	Salidroside content	Rosavin
**RR-Eur-S**	Rhodiola - Europharma	5.1 %	3.1	3.7
**RR-Chi-R**	Rhodiola - China - Rosavin 3%, Sal. 1%	2.1 %	2.5	1.2
**RR-Chi-S**	Rhodiola - China - Sal. 1%	0.0 %	1.1	0
**RR-Alt-S**	Rhodiola Altai Sobolev	3.2 %	1.9	1.3
**RR-Alt-B**	Rhodiola Altai Bykov	2.1 %	0.9	1.1
**RR-Alt-X**	Rhodiola Altai Xoren	1.5 %	1.8	0.1
**RR-Alt-G**	Rhodiola Altai Narine	4.8 %	2.1	3.1
**Rosavin**	Sigma batch Nr 84954-92-7	100%	0%	0
**Salidroside**	Sigma batch Nr HW 101 551	0%	100%	100
**KSM 66**	Withanian somnifera			
**BA**	Bryonia alba			
**KSM 66 + RR**	Withania + Rhodiola-Europharma, 1.5:10			
**BA + RR**	Bryonia + Rhodiola-Europharma, 1.5:10			

Tab. 1 Test items as used in this experimental series.

Results were fitted by a mathematical tangens hyperbolicus function to calculate EC50 values according to " y= a * tan H (b * (x-c)) + d ". Parameters are fitted in order to minimize the sum of error squares.

6 Results

6.1 Population spike analysis in the presence of Salidroside

In the presence of Salidroside amplitudes of the population spike were enhanced in a concentration dependent manner. During single stimuli amplitudes reached about 2 mV, during theta burst stimulation about 4 mV (Fig. 3). EC_{50} values of 0.47 and 0.49 mg/L were calculated during single stimuli and TBS, respectively. For comparison with other preparations see Tab. 2.

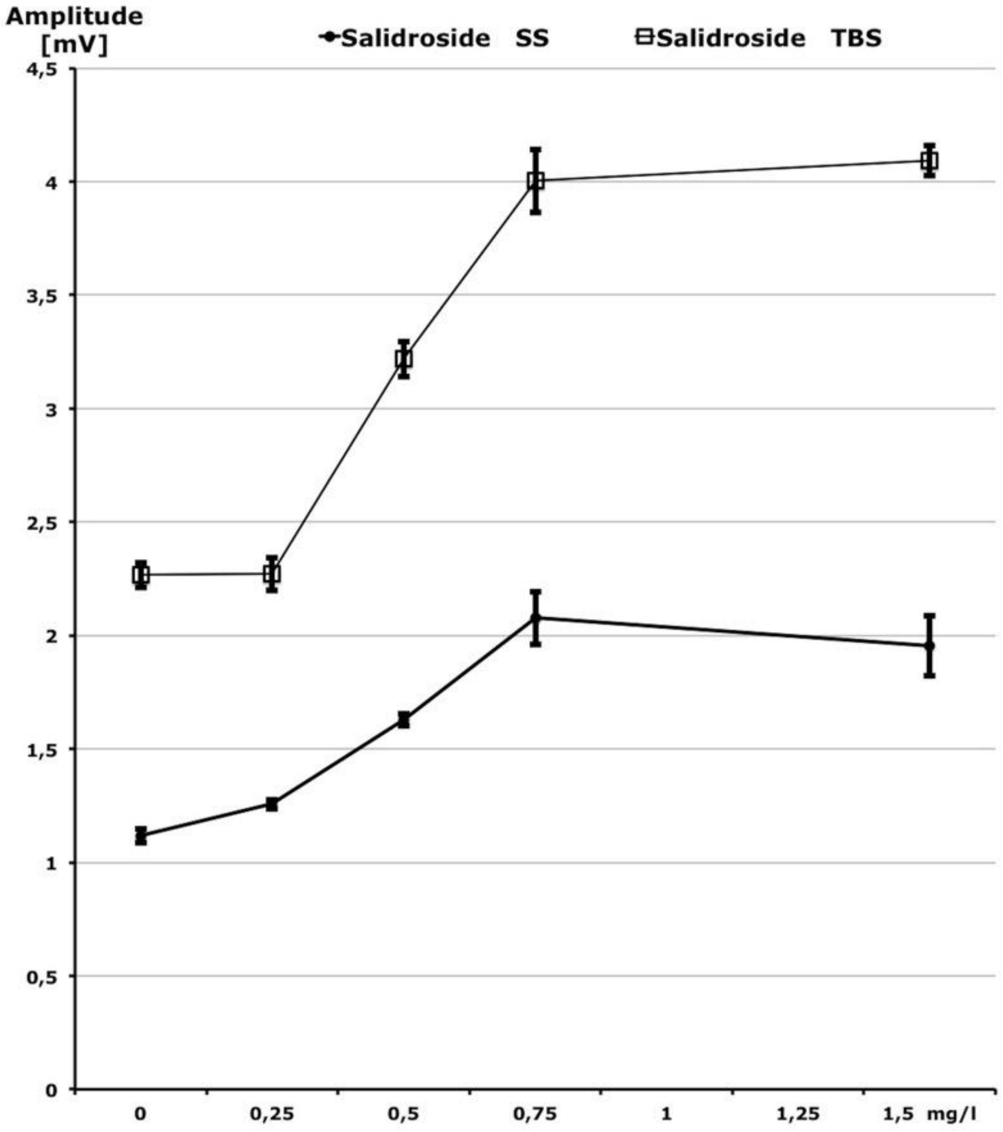

Fig. 3 Concentration dependent effects of Salidroside on pyramidal cell activity in terms of changes of population spike amplitudes (as voltage on the ordinate). Results are obtained after performance of single stimuli (60-80 min) or after burst stimuli (90-120 min). Data are given as mean ± S.E.M. of n=4 slices (all concentrations).

6.2 Population spike analysis in the presence of Rosavin

In the presence of Rosavin amplitudes of the population spike were enhanced in a concentration dependent manner. During single stimuli amplitudes reached about 2.3 mV, during theta burst stimulation about 4.3 mV (Fig. 4). EC_{50} values of 0.44 and 0.57 mg/L were calculated during single stimuli and TBS, respectively. For comparison with other preparations see Tab. 2.

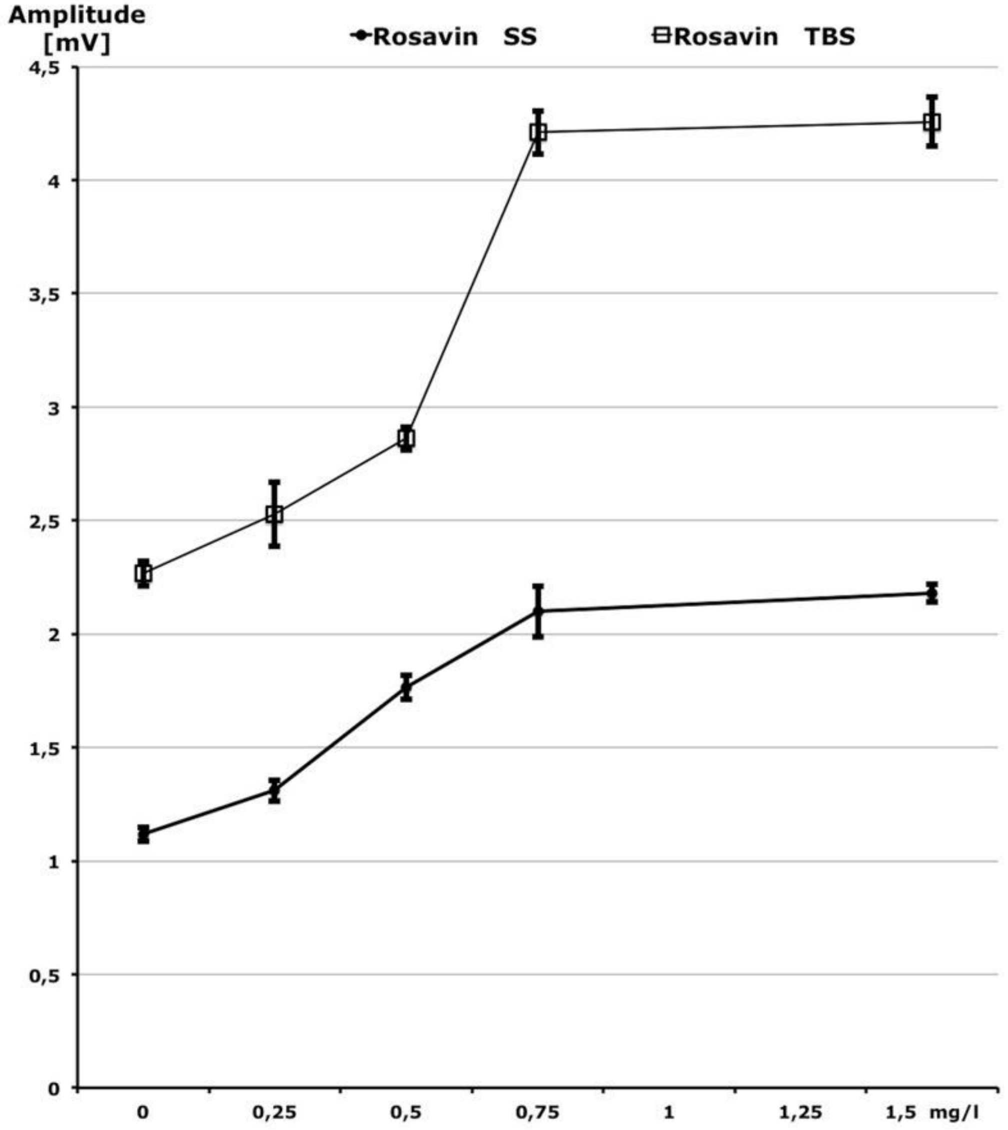

Fig. 4 Concentration dependent effects of Rosavin on pyramidal cell activity in terms of changes of population spike amplitudes (as voltage on the ordinate). Results are obtained after performance of single stimuli (60-80 min) or after burst stimuli (90-120 min). Data are given as mean ± S.E.M. of n=4 slices (all concentrations).

6.3 Population spike analysis in the presence of RR-EUR-S

In the presence of RR-EUR-S amplitudes of the population spike were enhanced in a concentration dependent manner. During single stimuli amplitudes reached about 2.3 mV, during theta burst stimulation about 4.3 mV (Fig. 5). EC_{50} values of 4.81 and 4.60 mg/L were calculated during single stimuli and TBS, respectively. For comparison with other preparations see Tab. 2.

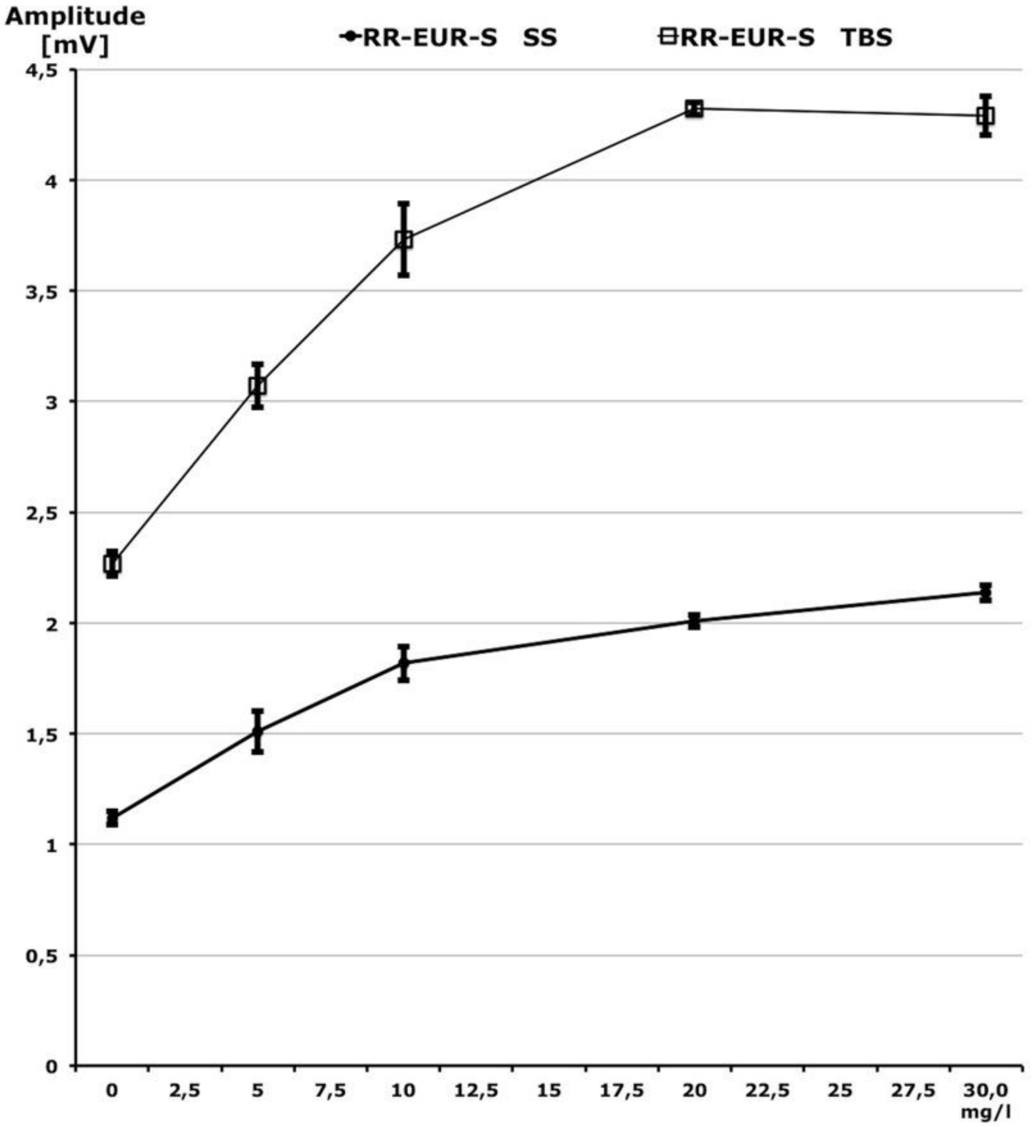

Fig. 5 Concentration dependent effects of RR-Eur S on pyramidal cell activity in terms of changes of population spike amplitudes (as voltage on the ordinate). Results are obtained after performance of single stimuli (60-80 min) or after burst stimuli (90-120 min). Data are given as mean ± S.E.M. of n=4 slices (all concentrations).

6.4 Population spike analysis in the presence of RR-Chi-R

In the presence of RR-Chi-R amplitudes of the population spike were enhanced in a concentration dependent manner. During single stimuli amplitudes reached about 2.2 mV, during theta burst stimulation about 4.2 mV (Fig. 6). EC_{50} values of 14.60 and 19.54 mg/L were calculated during single stimuli and TBS, respectively. For comparison with other preparations see Tab. 2.

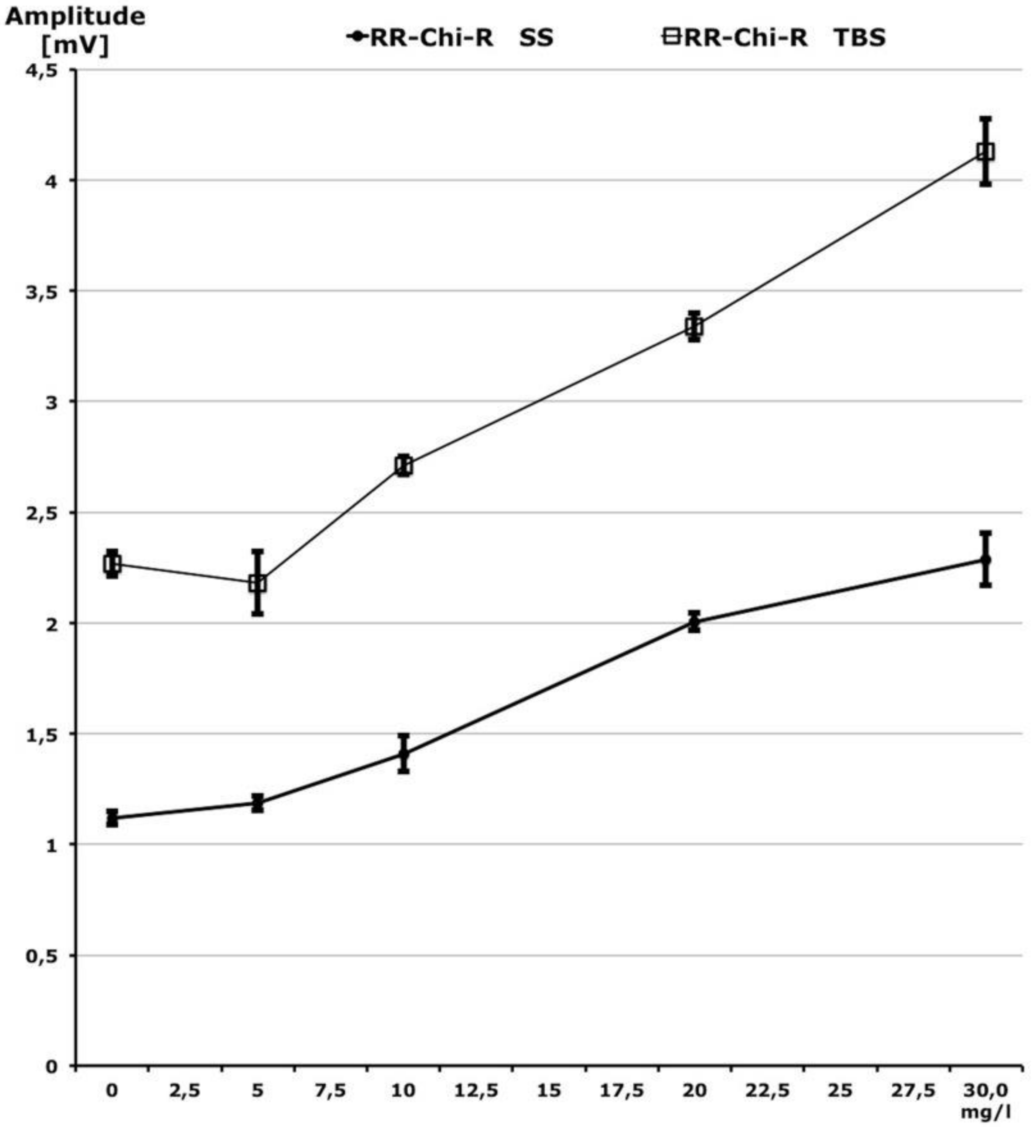

Fig. 6 Concentration dependent effects of RR-Chi-R on pyramidal cell activity in terms of changes of population spike amplitudes (as voltage on the ordinate). Results are obtained after performance of single stimuli (60-80 min) or after burst stimuli (90-120 min). Data are given as mean ± S.E.M. of n=4 slices (all concentrations).

6.5 Population spike analysis in the presence of RR-Chi-S

In the presence of RR-Chi-S amplitudes of the population spike were enhanced in a concentration dependent manner. During single stimuli amplitudes reached about 2.2 mV, during theta burst stimulation about 4.2 mV (Fig. 7). EC_{50} values of 9.79 and 15.03 mg/L were calculated during single stimuli and TBS, respectively. For comparison with other preparations see Tab. 2.

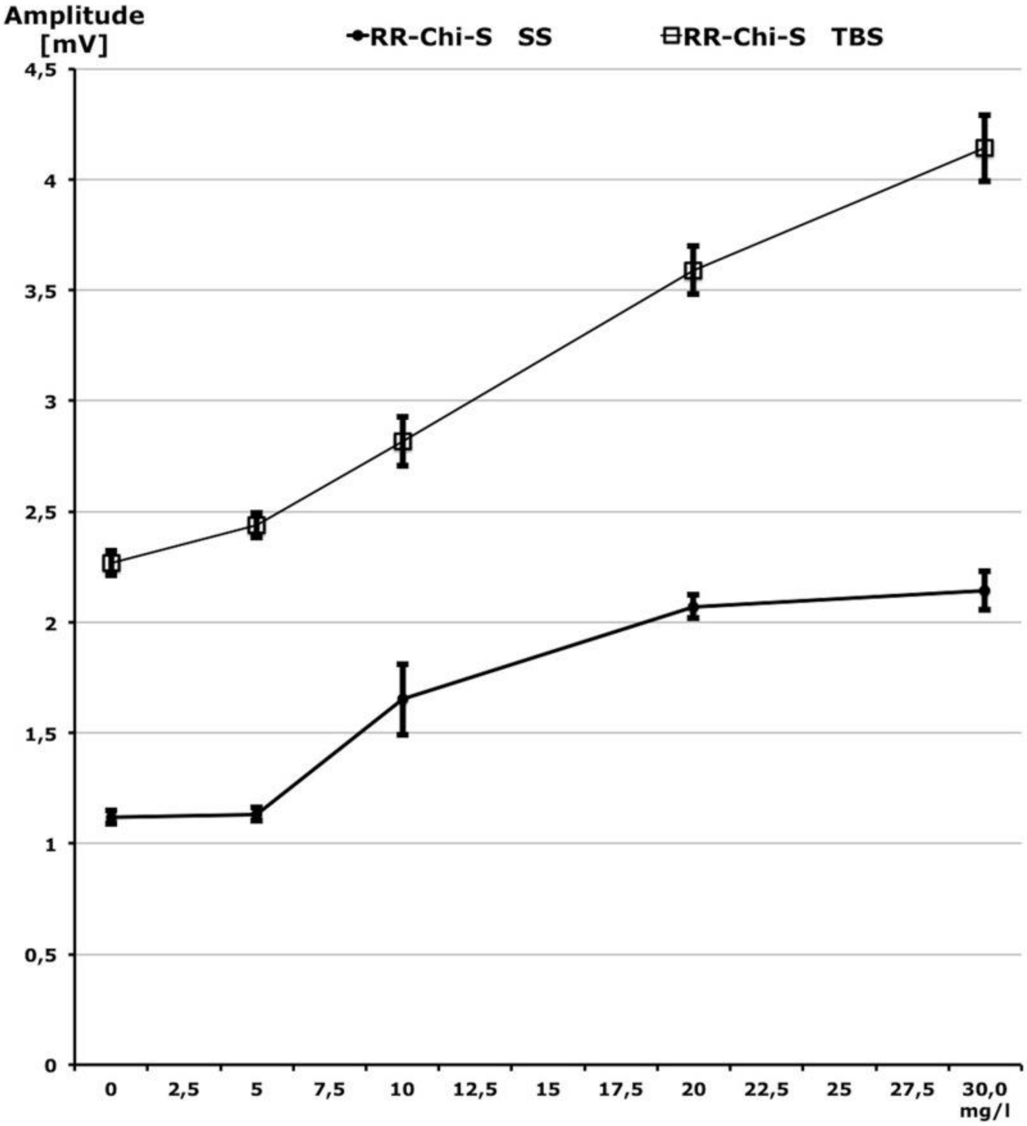

Fig. 7 Concentration dependent effects of RR-Chi-S on pyramidal cell activity in terms of changes of population spike amplitudes (as voltage on the ordinate). Results are obtained after performance of single stimuli (60-80 min) or after burst stimuli (90-120 min). Data are given as mean ± S.E.M. of n=4 slices (all concentrations).

6.6 Population spike analysis in the presence of RR-Alt-S

In the presence of RR-Alt-S amplitudes of the population spike were enhanced in a concentration dependent manner. During single stimuli amplitudes reached about 2.1 mV, during theta burst stimulation about 4.2 mV (Fig. 8). EC_{50} values of 8.89 and 7.73 mg/L were calculated during single stimuli and TBS, respectively. For comparison with other preparations see Tab. 2.

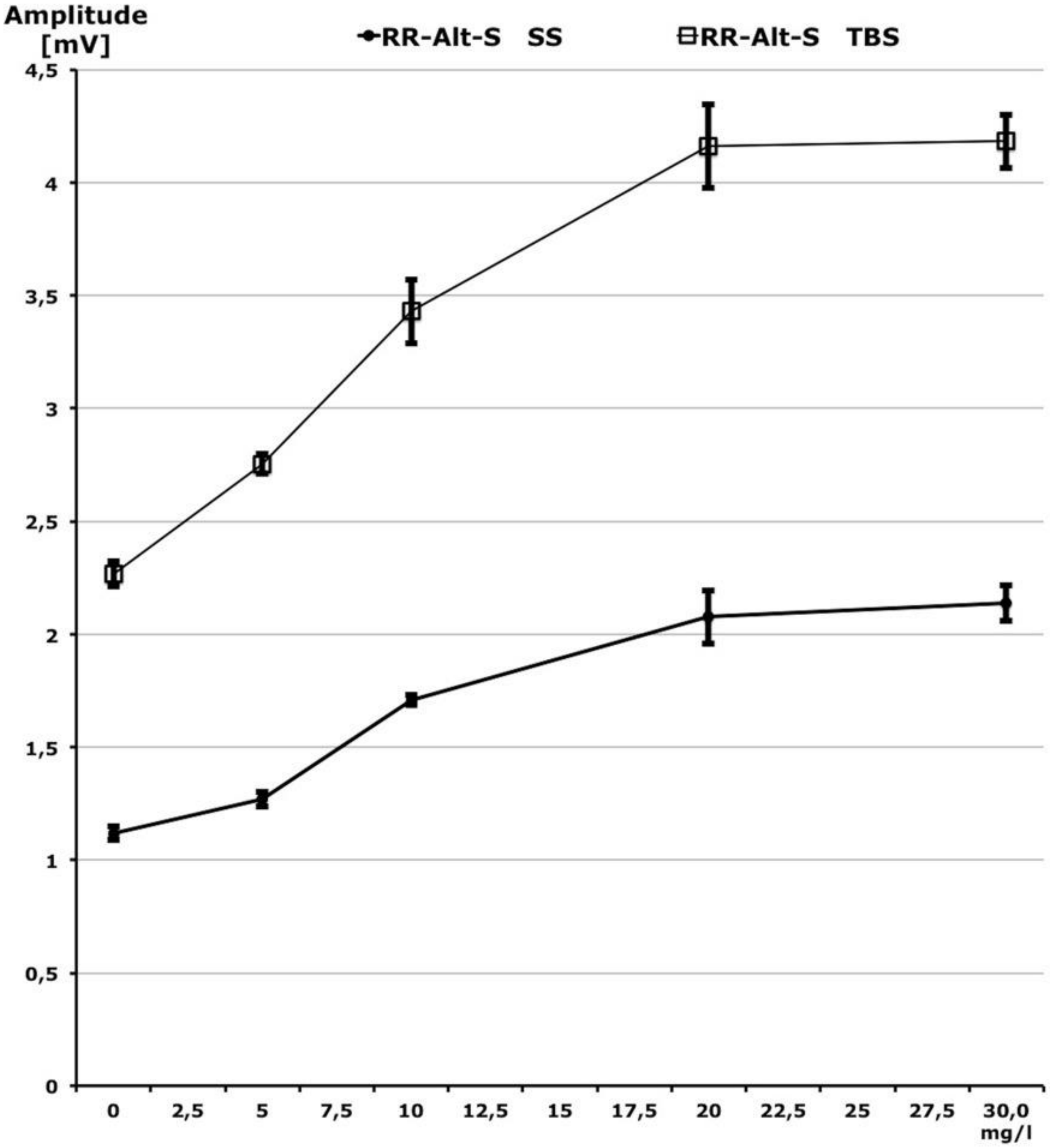

Fig. 8 Concentration dependent effects of RR-Alt-S on pyramidal cell activity in terms of changes of population spike amplitudes (as voltage on the ordinate). Results are obtained after performance of single stimuli (60-80 min) or after burst stimuli (90-120 min). Data are given as mean ± S.E.M. of n=4 slices (all concentrations).

6.7 Population spike analysis in the presence of RR-Alt-B

In the presence of RR-Alt-B amplitudes of the population spike were enhanced in a concentration dependent manner. During single stimuli amplitudes reached about 2.2 mV, during theta burst stimulation about 4.1 mV (Fig. 9). EC_{50} values of 15.74 and 13.77 mg/L were calculated during single stimuli and TBS, respectively. For comparison with other preparations see Tab. 2.

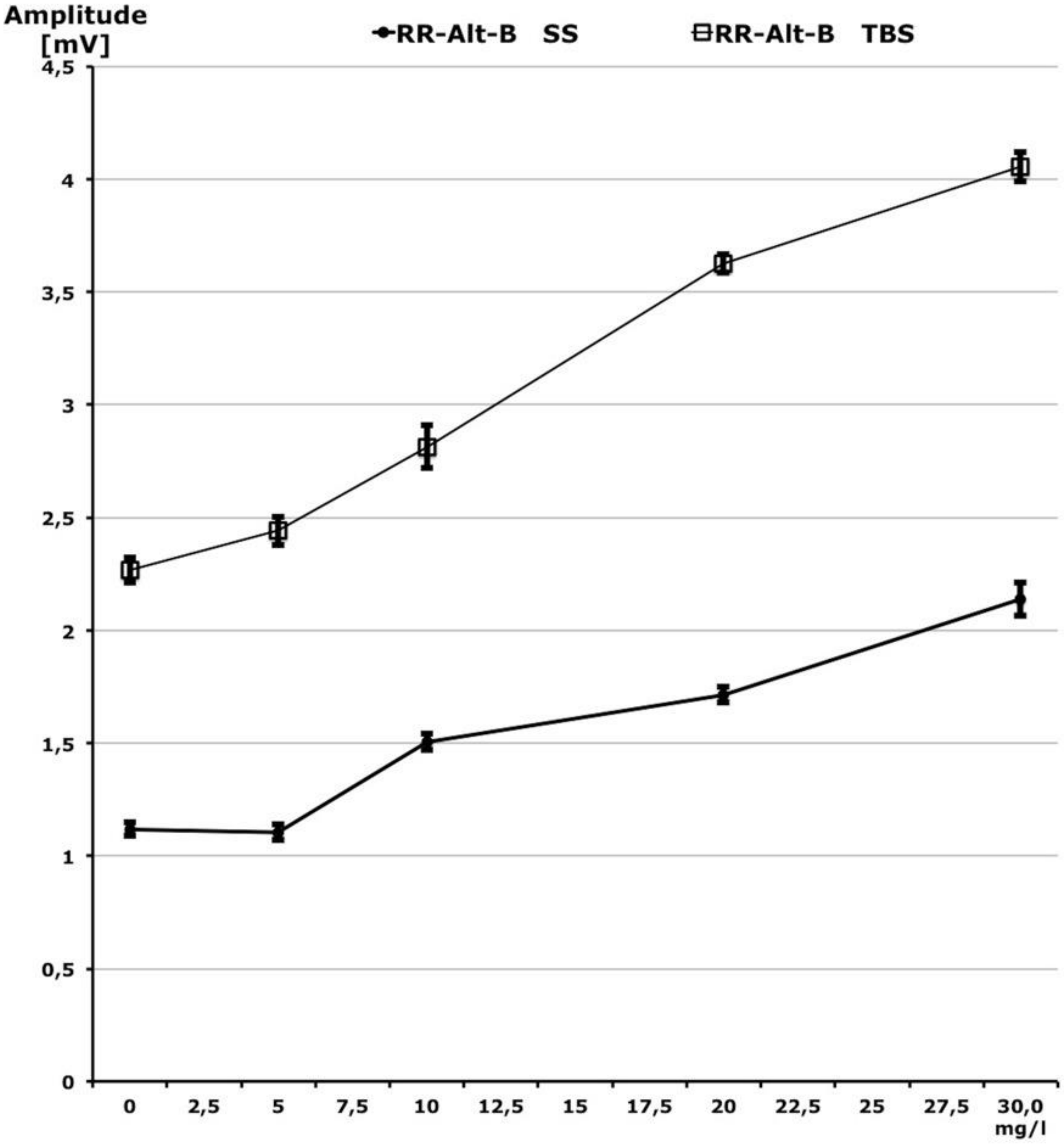

Fig. 9 Concentration dependent effects of RR-Alt-B on pyramidal cell activity in terms of changes of population spike amplitudes (as voltage on the ordinate). Results are obtained after performance of single stimuli (60-80 min) or after burst stimuli (90-120 min). Data are given as mean ± S.E.M. of n=4 slices (except for 10 mg/L= 3 slices due to one outlier).

6.8 Population spike analysis in the presence of RR-Alt-X

In the presence of RR-Alt-X amplitudes of the population spike were enhanced in a concentration dependent manner. During single stimuli amplitudes reached about 2.2 mV, during theta burst stimulation about 4.4 mV (Fig. 10). EC_{50} values of 10.68 and 14.42 mg/L were calculated during single stimuli and TBS, respectively. For comparison with other preparations see Tab. 2.

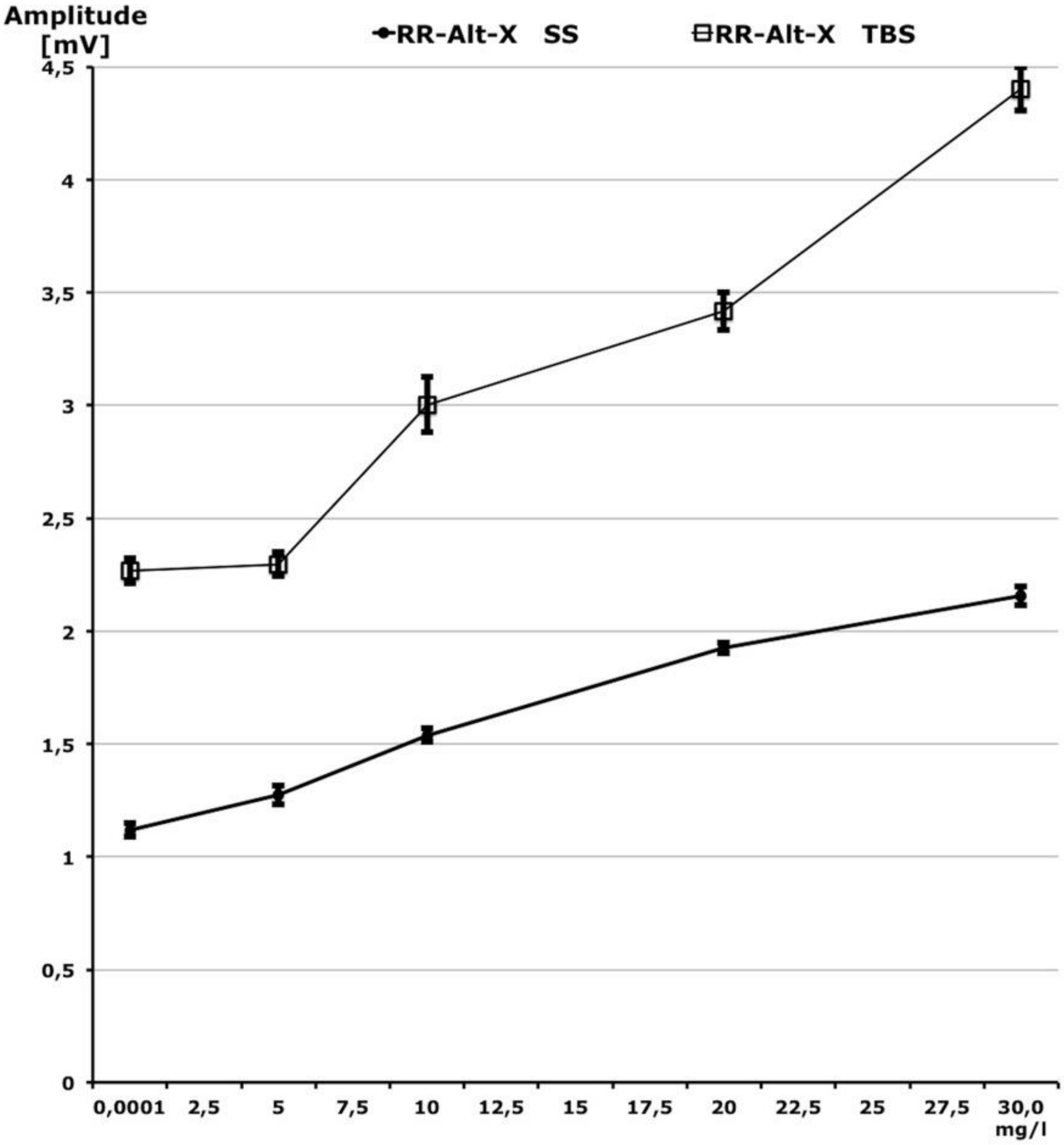

Fig. 10 Concentration dependent effects of RR-Alt-X on pyramidal cell activity in terms of changes of population spike amplitudes (as voltage on the ordinate). Results are obtained after performance of single stimuli (60-80 min) or after burst stimuli (90-120 min). Data are given as mean ± S.E.M. of n=4 slices except for the 10 mg/L concentration (n=3 slices) due to one outlier.

6.9 Population spike analysis in the presence of RR-Alt-G

In the presence of RR-Alt-G amplitudes of the population spike were enhanced in a concentration dependent manner. During single stimuli amplitudes reached about 2.3 mV, during theta burst stimulation about 4.2 mV (Fig. 11). EC_{50} values of 12.39 and 17.69 mg/L were calculated during single stimuli and TBS, respectively. For comparison with other preparations see Tab. 2.

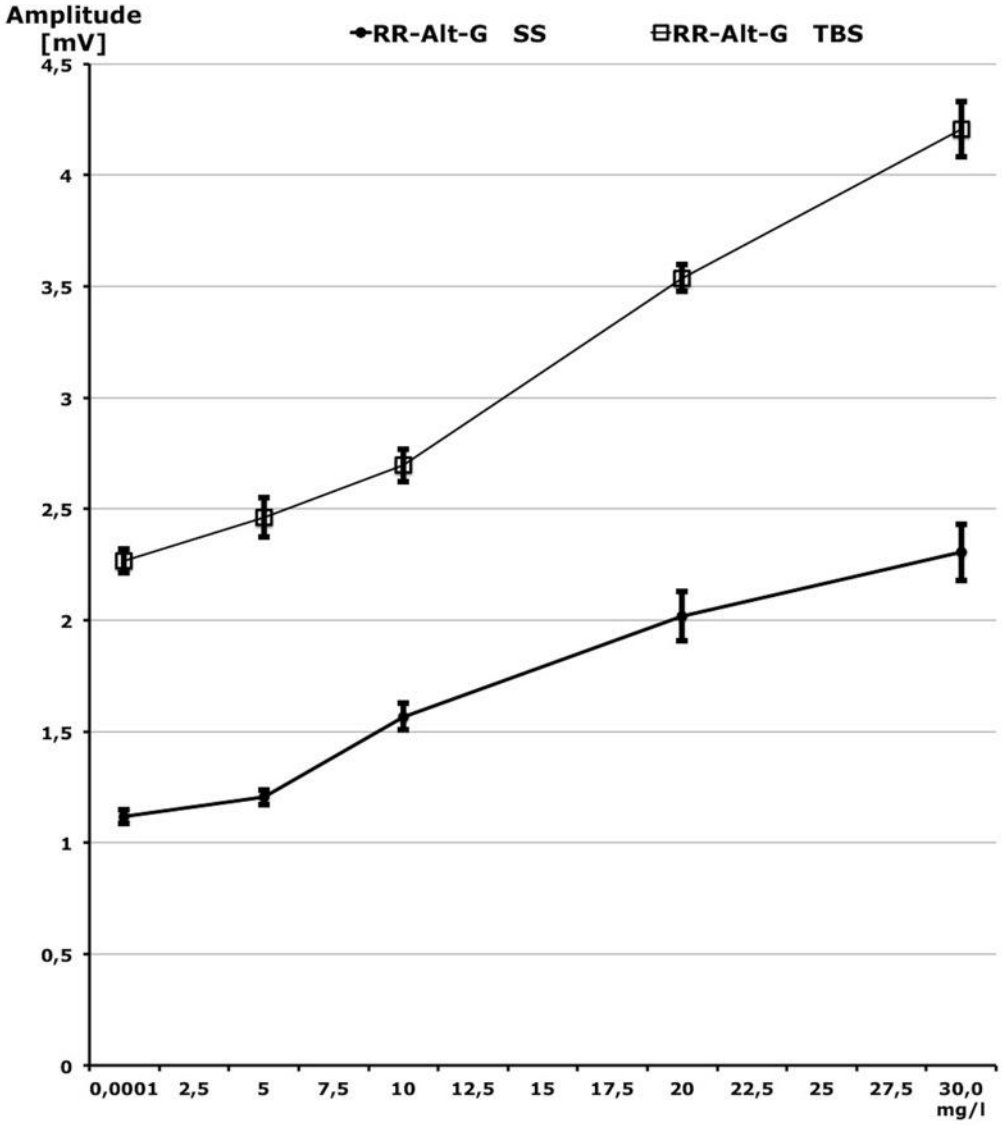

Fig. 11 Concentration dependent effects of RR-Alt-G on pyramidal cell activity in terms of changes of population spike amplitudes (as voltage on the ordinate). Results are obtained after performance of single stimuli (60-80 min) or after burst stimuli (90-120 min). Data are given as mean ± S.E.M. of n=4 slices (all concentrations).

6.10 Population spike analysis in the presence of WS KSM66

In the presence of WS KSM66 amplitudes of the population spike were enhanced in a concentration dependent manner up to 1.5 mg/L. During single stimuli amplitudes reached about 2.2 mV, during theta burst stimulation about 3.7 mV (Fig. 12). EC_{50} values of 0.50 and 0.57 mg/L were calculated during single stimuli and TBS, respectively. For comparison with other preparations see Tab. 2. However, higher concentrations up to 10 mg/L (not shown) had an opposite effect resulting in concentration dependent **decreases** of the population spike amplitude.

[illegible]paration

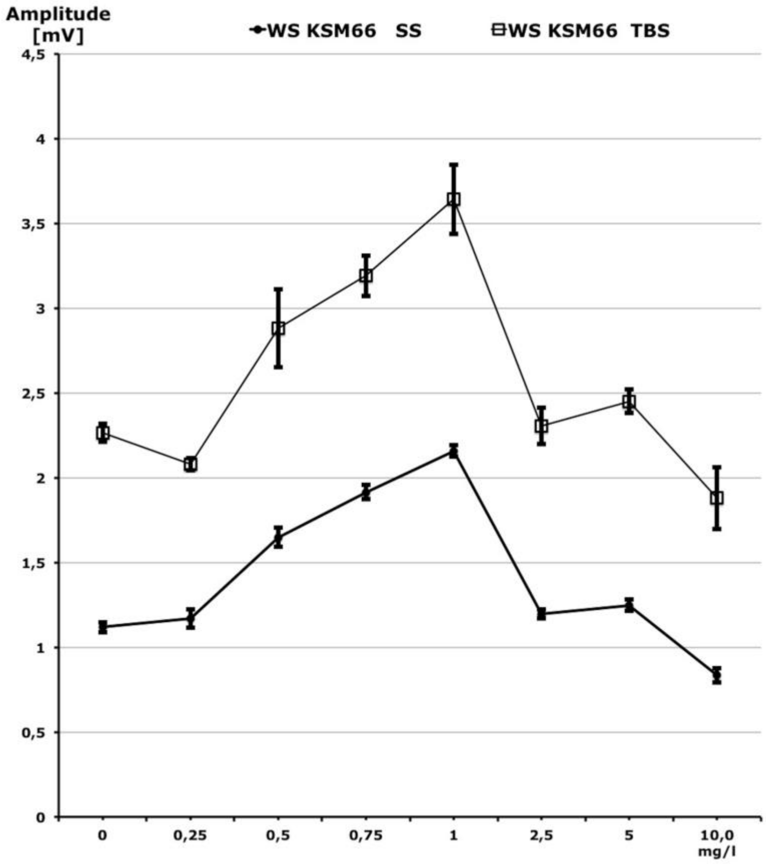

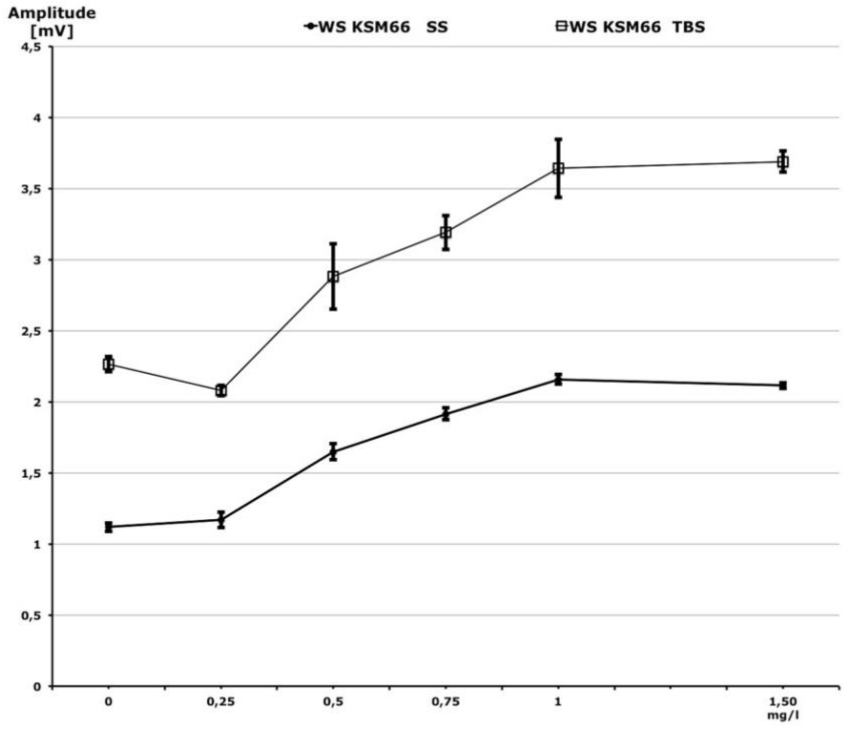

Fig. 12 Concentration dependent effects of WS KSM66 on pyramidal cell activity in terms of changes of population spike amplitudes (as voltage on the ordinate). Results are obtained after performance of single stimuli (60-80 min) or after burst stimuli (90-120 min). Data are given as mean ± S.E.M. of n=4 slices (all concentrations).

6.11 Population spike analysis in the presence of Bryonia alba

In the presence of Bryonia alba amplitudes of the population spike were enhanced in a concentration dependent manner. During single stimuli amplitudes reached about 2.1 mV, during theta burst stimulation about 4.1 mV (Fig. 13). EC_{50} values of 0.53 and 0.91 mg/L were calculated during single stimuli and TBS, respectively. For comparison with other preparations see Tab. 2.

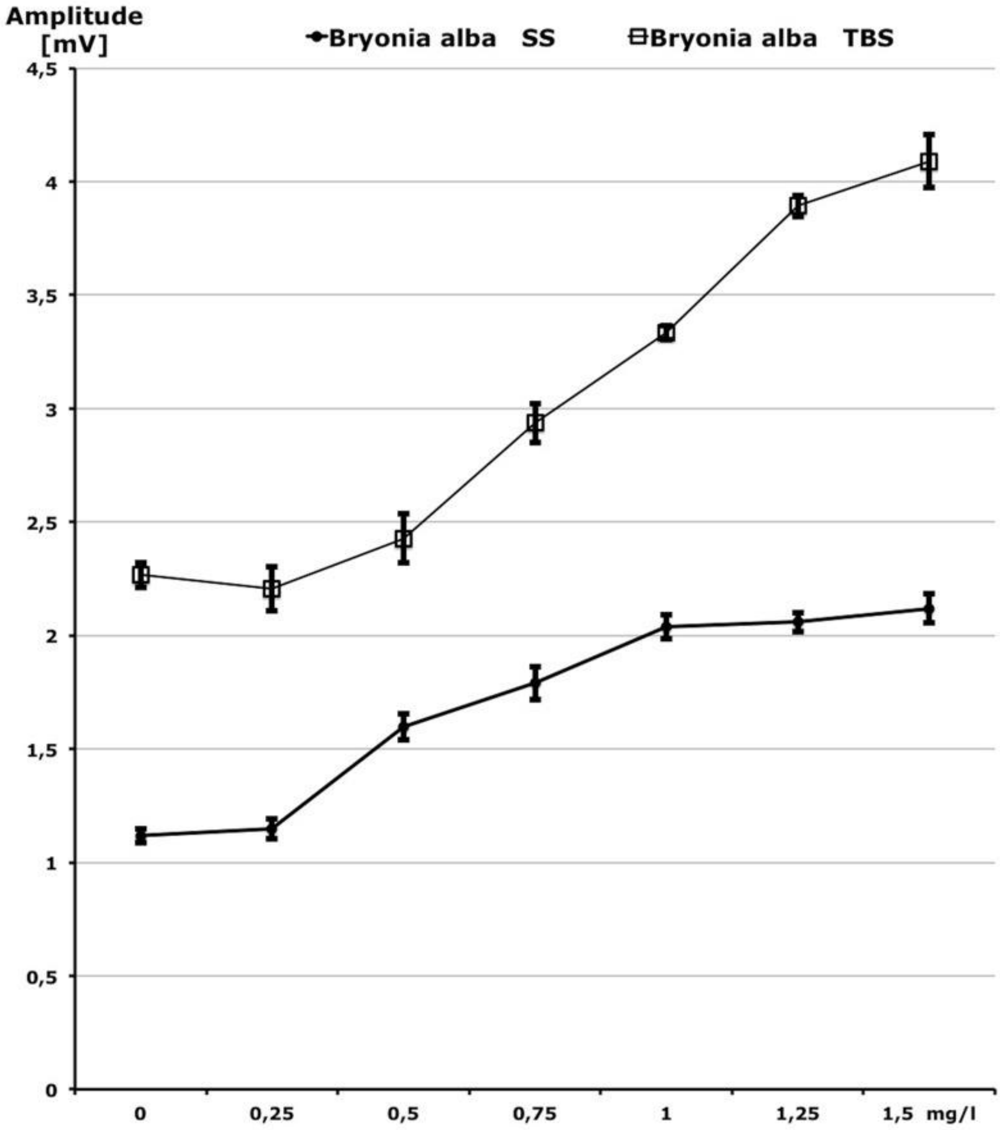

Fig. 13 Concentration dependent effects of Bryonia alba on pyramidal cell activity in terms of changes of population spike amplitudes (as voltage on the ordinate). Results are obtained after performance of single stimuli (60-80 min) or after burst stimuli (90-120 min). Data are given as mean ± S.E.M. of n=4 slices (all concentrations).

Report NCAG 15/17 H – Hippocampus slice preparation

Regarding now the concentration – response relationships for single compounds and the different extracts maximum values during single stimuli of about 2 mV were recorded, whereas TBS resulted in amplitudes of the population spike of about 4 mV, in general. Only WS KMS66 (Withania somnifera) induced somewhat lower maximum values and produced **decreases** of the amplitudes at considerable higher concentrations. In order to allow a more direct comparison of all samples results were fitted by a tangens hyperbolicus function resulting in the documentation of EC_{50} values. Single curves are depicted for Rosavin, Salidroside, Brynonia alba and Withania somnifera in Fig. 14 (please note similar concentration range on abscissa). Results for the different Rhodiola extracts are documented in Fig. 15a and 15b (Please note similar concentration range on abscissa).

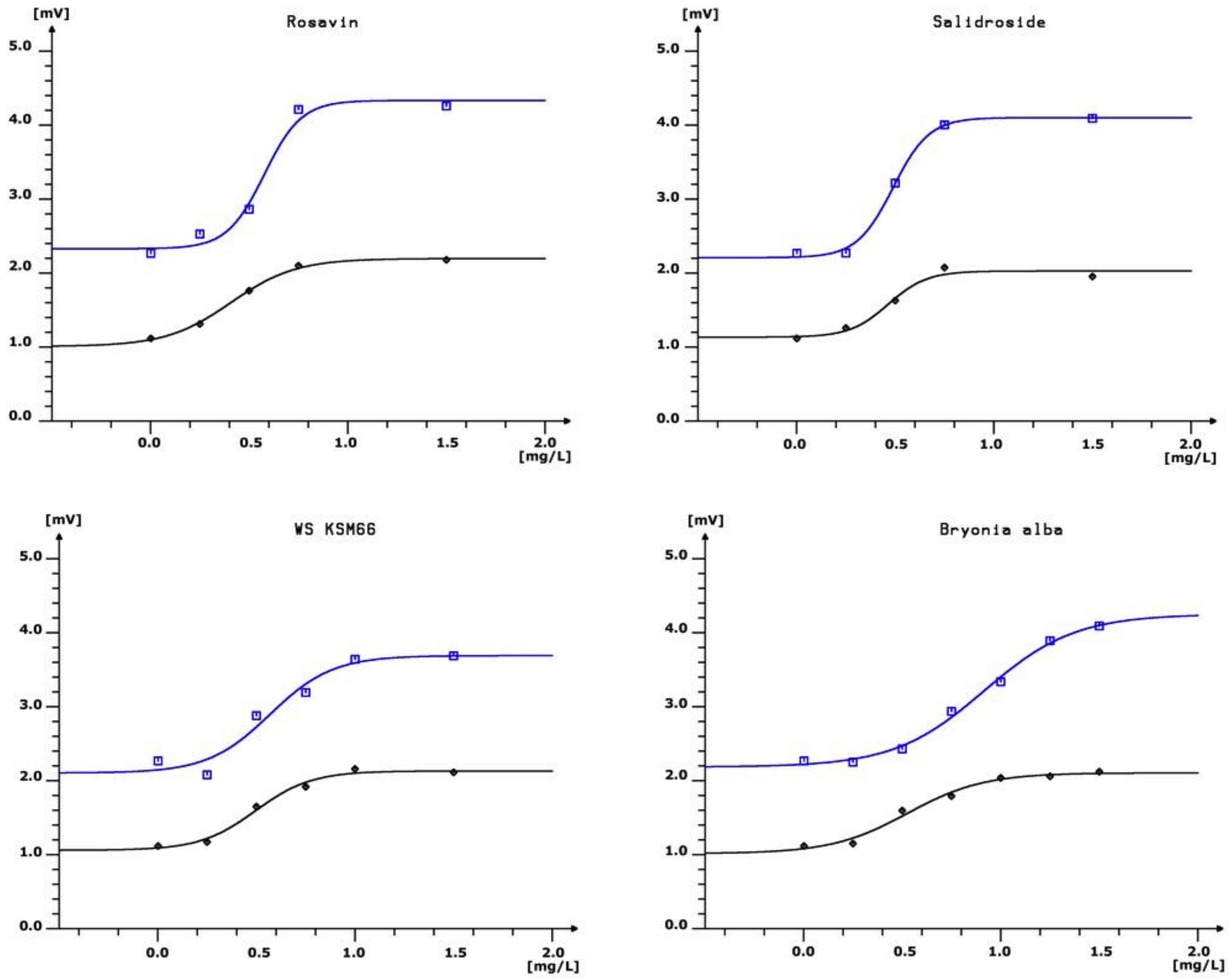

Fig. 14 Tangens hyperbolicus fits for concentration dependent effects on population spike amplitude in the presence of different samples.

Report NCAG 15/17 H – Hippocampus slice preparation

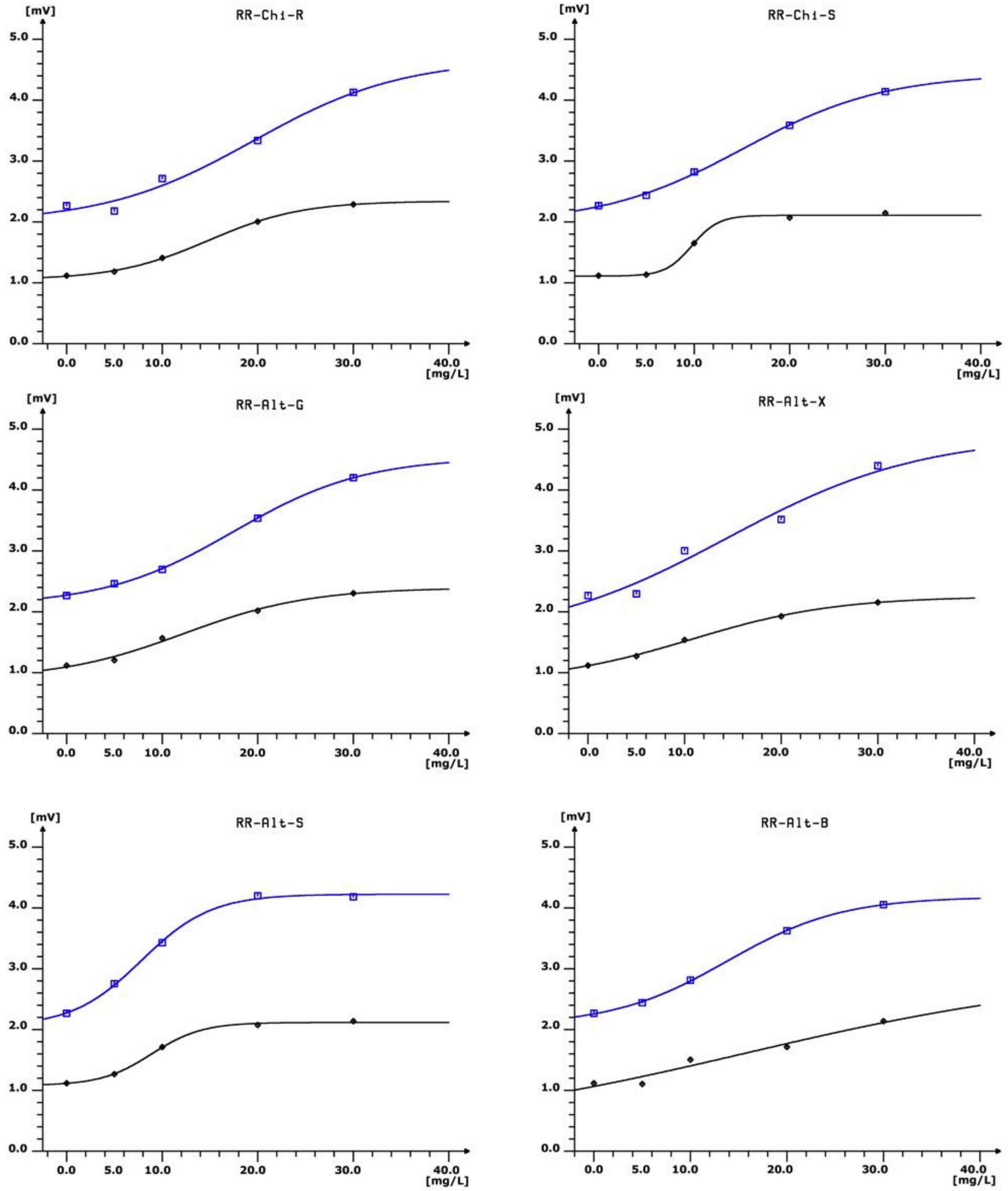

Fig. 15a Tangens hyperbolicus fits for concentration dependent effects on population spike amplitude in the presence of different samples. Please note different concentration range in comparison to Fig. 14.

Report NCAG 15/17 H – Hippocampus slice preparation

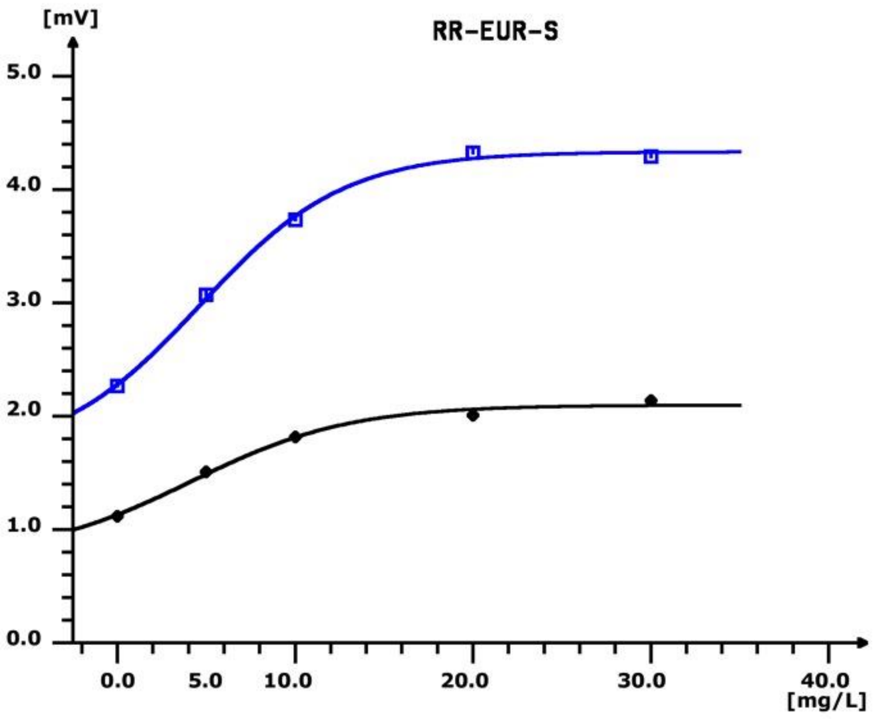

Fig. 15b Tangens hyperbolicus fit for concentration dependent effects on population spike amplitude in the presence of RR-EUR-S. Please note different concentration range in comparison to Fig. 14.

Based on these mathematical fits EC_{50} values were calculated. Among the Rhodiola extracts RR-EUR-S had the lowest EC_{50}. Rosavin and Salidroside had EC_{50} values around 0.5 mg/L. Withania somnifera extract also had an EC_{50} of about 0.5 during single stimulation and TBS. For Bryonia alba extract an EC_{50} of about 0.5 mg/L was calculated during single shock stimulation and 0.9 mg/L for TBS. An overview on all EC_{50} values is given in Tab. 2.

	**EC_{50} Values for SS**	**EC_{50} Values for TBS**
**RR-EUR-S**	**4.81**	**4.60**
**RR-Chi-R**	**14.60**	**19.54**
**RR-Chi-S**	**9.79**	**15.03**
**RR-Alt-S**	**8.89**	**7.73**
**RR-Alt-B**	**15.74**	**13.77**
**RR-Alt-X**	**10.68**	**14.42**
**RR-Alt-G**	**12.39**	**17.69**
**Rosavin**	**0.44**	**0.57**
**Salidroside**	**0.47**	**0.49**
**WS KSM66**	**0.50**	**0.57**
**Bryonia Alba**	**0.53**	**0.91**
**WS+RR, 1.5:10**		
**BA+RR, 1.5:10**		

Tab. 2 EC_{50} values in the presence of single shock stimulation (SS) and theta burst stimulation (TBS). Numbers refer to mg/L.

6.12 Population spike analysis in the presence of the combination of RR-EUR-S and WS KSM66

In the presence of the combination of RR-EUR-S plus WS KSM66 amplitudes of the population spike were enhanced. During single stimuli amplitudes reached about 2.2 mV, during theta burst stimulation about 4.2 mV (Fig. 16). At least an additive effect is observe since the combination of the two EC_{50} dosages results in reaching maximal values observed at the highest concentrations for the single extracts as depicted in Fig. 17.

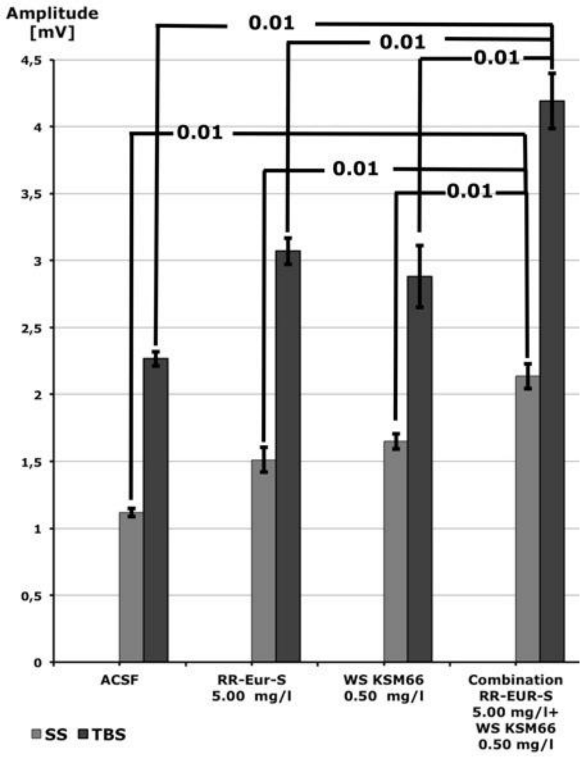

Fig. 16 Effect of a combination of RR-EUR-S (EC_{50}) plus WS KSM66 (EC_{50}) on pyramidal cell activity in terms of changes of population spike amplitudes (as voltage on the ordinate). Results are obtained after performance of single stimuli (grey) or after burst stimuli (black). Data are given as mean ± S.E.M. of n=4 slices.

Report NCAG 15/17 H – Hippocampus slice preparation

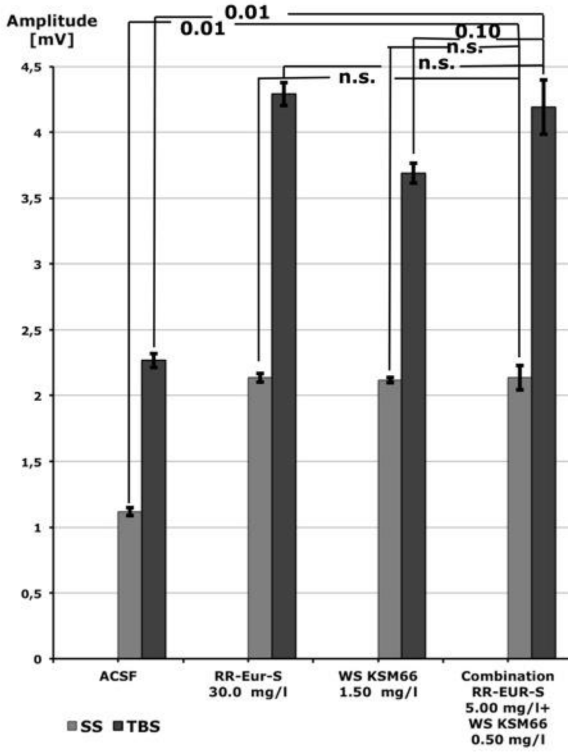

Fig. 17 Effect of a combination of RR-EUR-S (EC_{50}) plus WS KSM66 (EC_{50}) on pyramidal cell activity in terms of changes of population spike amplitudes (as voltage on the ordinate). Comparison to the maximum values obtained with the highest dosage. Results are obtained after performance of single stimuli (grey) or after burst stimuli (black min). Data are given as mean ± S.E.M. of n=4 slices.

6.13 Population spike analysis in the presence of a combination of RR-EUR-S and Bryonia alba

In the presence of the combination of RR-EUR-S plus Bryonia alba amplitudes of the population spike were not higher than in the presence of the EC_{50} dosages, but higher than during control. During single stimuli amplitudes reached about 1.5 mV, during theta burst stimulation about 3.3 mV (Fig. 18). Combination of the two EC_{50} dosages did not result in reaching maximal values as depicted in Fig. 19. Neither an additive effect nor a potentiation was observed.

Report NCAG 15/17 H – Hippocampus slice preparation

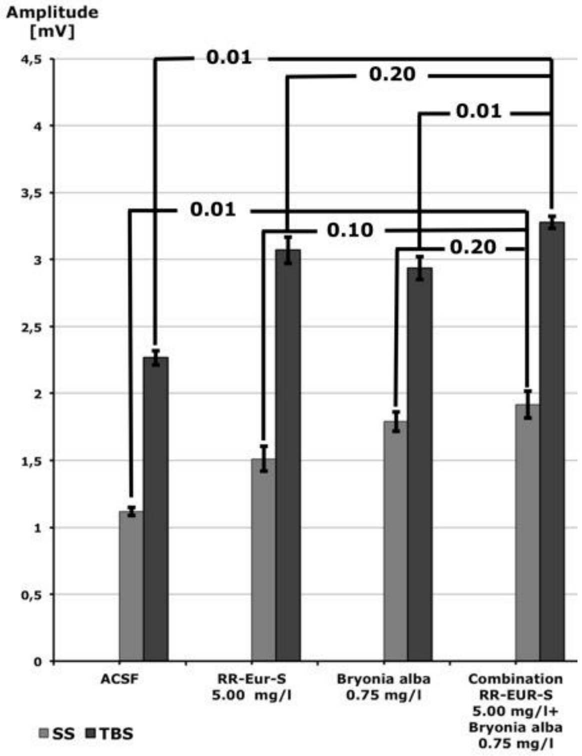

Fig. 18 Effect of a combination of RR-EUR-S (EC_{50}) plus Bryonia alba (EC_{50}) on pyramidal cell activity in terms of changes of population spike amplitudes (as voltage on the ordinate). Results are obtained after performance of single stimuli (grey) or after burst stimuli (black min). Data are given as mean ± S.E.M. of n=4 slices.

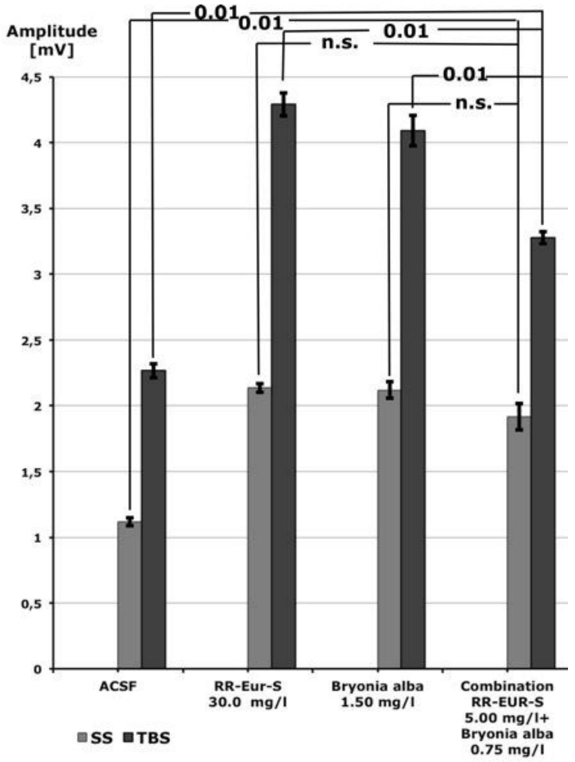

Fig. 19 Effect of a combination of RR-EUR-S (EC_{50}) plus Bryonia alba (EC_{50}) on pyramidal cell activity in terms of changes of population spike amplitudes (as voltage on the ordinate). Results are obtained after performance of single stimuli (grey) or after burst stimuli (black min). Data are given as mean ± S.E.M. of n=4 slices.

7 Discussion

The current investigation aimed at the comparison of different Rhodiola extracts with respect to their efficacy in the hippocampus in vitro slice model in order to identify the best one for a clinical study. During single stimuli as well as during theta burst stimulation the concentration dependence was tested by recording the population spike amplitude created by pyramidal cells in response to the stimuli. Main constituents of Rhodiola rosea extract, namely Rosavin and Salidroside, were tested in separate. Both ingredients as well as all Rhodiola extracts induced concentration dependent increases of the population spike amplitudes reaching maximum values of 2 mV (single stimuli) or about 4 mV (TBS). Only Withania somniferum extract did not reach these maximum values.
All results were fitted by a mathematical tangens hyperbolicus function to give EC_{50} values (effective concentration to induce half-maximal effect). The lower these EC_{50} values are, the less of a compound is needed to exert its pharmacological effect. The lower the effective concentration, the less side effects have to be expected, especially in herbal preparations, where many chemicals might become active only at higher concentrations. According to this analysis the sample RR-EUR-S had the lowest EC_{50} among the Rhodiola extracts (s. Tab. 2) and should be selected for a clinical study. Comparing the two chinese extracts, which differ with respect to their content of Rosavin and Salidroside, provide different EC_{50} values. RR-Chi-S has lower values of about 10 mg/L in the presence of single shock stimulation and about 15 mg/L in the presence of TBS. RR-Chi-R has definitely higher EC_{50} values of 15 mg/L and about 20 mg/L, respectively. The samples from Altai also differ with respect to EC_{50} values. RR-Alt-S was recognized to have the lowest values of about 8 mg/L for both stimulation patterns. RR-Alt-X seems to be the second best of this Alt-group. For the other two of this group - RR-Alt-G and RR-Alt-B - obviously higher concentrations were needed to induce maximum effects despite the fact, that RR-Alt-G contained the highest amount of Rosavin within this group from Altai. Thus, comparing the EC_{50} values for the different extracts with their contents of Rosavin and/or Salidroside no correlation or relationship was seen despite the fact, that RR-EUR-S had the highest content of both. From this one might conclude that not only Rosavin or Salidroside might account for the efficacy of the extracts in this model, but there are some other active compounds in the extract and synergistic interactions are possible. This result speaks in favor of the idea that other ingredients contained in the extracts may also play a role. One must also be aware of the fact that we expose the brain directly to the samples and by it circumvent the blood brain barrier. However, both preclinical and clinical studies have proven, that Rhodiola extract exerts actions on the brain, by it suggesting that enough amounts of ingredients pass the blood brain barrier. But, a direct comparison to blood concentrations has not been looked at, so far. However, there is no other methodology, which is able to detect such small differences in effectiveness between extracts. All "in vivo" models suffer from a large inter-individual variability, which asks for very high numbers of animals in order to detect small differences in effectiveness. On the opposite, the strictly controlled in vitro experiments have allowed for discovery of very small and concentration dependent effects with a minimum of effort and costs.
Combining the Rhodiola extract RR-EUR-S with Withania extract WS KSM66 resulted in at least an additive effect of both, since only the EC_{50} concentrations were added, but induced already a maximum effect. Possibly, even lower concentrations might result in maximal effects pointing to real potentiating effects. On the

opposite, combination of the same Rhodiola extract with Bryonia alba extract (EC_{50} concentration for each) only reached somewhat higher population spike amplitudes during TBS as compared to single EC_{50} concentrations alone. Thus, mixing various extracts might be very meaningful and the success can be tested with reasonable effort in this model.

In summary, Rosavin, Salidroside, various extracts from Rhodiola rosea, an extract from Withania somniferum, an extract of Bryonia alba as well as their combination with Rhodiola extract RR-EUR-S were investigated using hippocampus slices in vitro. Electric stimulation of an intra-hippocampal electric circuit resulted in higher responses of the pyramidal cells in the presence of all extracts. The responses were concentration dependent. Lowest half-maximal effective concentration (EC_{50}) was detected in the presence of Rosavin, Salidroside, Withania and Bryonia extract (about 0.5 mg/L). Best Rhodiola extract was RR-EUR-S reaching half-maximum effectiveness already at 5 mg/L, whereas other extracts only reached 15 to 20 mg/L. A combination of RR-EUR-S with Withania extract revealed at least additive effects, whereas the combination with Bryonia extract did not induce a higher response. According to our experience a dosage of 5 mg/kg of RR-EUR-S - administered orally - would be appropriate for the clinical trial.

8 Literature

Dimpfel W, Spüler M, Dalhoff A, Hoffmann W, Schlüter G (1991) Hippocampal activity in the presence of quinolones and fenbufen in-vitro. Antimicrobial Agents and Chemotherapy, June, 1142-1146.

Dimpfel W, Dalhoff B, Hofmann W, Schlüter G (1994) Electrically evoked potentials in the rat hippocampus slice in the presence of aminophylline alone and in combination with quinolones. European Neuropsychopharmacology, 4, 151-156.

Dimpfel W (1995) Effects of Memantine on Synaptic Transmission in the Hippocampus in vitro. Arzneim.-Forsch./Drug. Res, 45, 1, 1-5.

Dimpfel W, Schombert L, Feistel, B (2016a) Ex vivo Characterization of the Action of Sideritis Extract Using Electrical Activity in the Hippocampus Slice Preparation. Pharmacology & Pharmacy, 7, 407-416.

Dimpfel W, Schombert L , Vega-Morales T and Wiebe J (2016b) Neuropharmacological Characterization of Extracts from Rhodiola rosea, Oenothera paradoxa and Paullinia cupana in Comparison to Caffeine. Pharmacology & Pharmacy, 7, 290-303.

Dimpfel W (2014) Neurophysiological Effects of Rhodiola Rosea Extract Containing Capsules (A Double-Blind, Randomised, Placebo-Controlled Study). International Journal of Nutrition and Food Sciences, 3, 157-165.

Dingledine R (1984) Brain Slices, Plenum Press New York, London.

Haas HL, Schaerer B, Vosmansky M (1979) A simple perfusion chamber for the study of nervous tissue slices in vitro. J Neurosci Methods, 1, 323-5.

Lynch G, Schubert P (1980) The use of in-vitro brain slices for multidisciplnary studies of synaptic function. Ann Rev Neurosci, 3, 1-22.

Schiff SJ, Somjen GG (1985) The Effects of Temperature on Synaptic Transmission in Hippocampal Tissue Slices. Brain Research, 345, 279-284.

Acknowledgement. We greatly appreciate the experimental work as well as the data documentation performed by Mrs. Leoni Schombert. Quality control was performed by Ingrid K. Keplinger-Dimpfel.

9 Appendix

Control															
slice	ACSF +SS					ACSF +SS					ACSF +TBS				
time [min]	10	20	30	40	Mean 20-40	50	60	70	80	Mean 60-80	90	100	110	120	Mean 100-120
1	-1105,00	-1113,00	-1080,00	-899,35	**-1030,78**	-1013,00	-1092,00	-855,45	-1264,00	**-1070,48**	-2235,00	-2699,00	-2359,00	-2689,00	**-2582,33**
2	-981,85	-1102,00	-1142,00	-1113,00	**-1119,00**	-1286,00	-1233,00	-1316,00	-1232,00	**-1260,33**	-2719,00	-2565,00	-2567,00	-2492,00	**-2541,33**
3	-549,34	-1365,00	-1179,00	-1133,00	**-1225,67**	-1092,00	-1234,00	-975,13	-1288,00	**-1165,71**	-2347,00	-2248,00	-2278,00	-2293,00	**-2273,00**
4	-1041,00	-1216,00	-1050,00	-1063,00	**-1109,67**	-1232,00	-1175,00	-1069,00	-943,51	**-1062,50**	-2137,00	-2035,00	-2209,00	-2164,00	**-2136,00**
5	-1059,00	-1091,00	-937,05	-946,53	**-991,53**	-934,76	-945,95	-903,57	-827,57	**-892,36**	-2250,00	-1933,00	-2163,00	-2073,00	**-2056,33**
6	-851,44	-940,91	-1008,00	-1113,00	**-1020,64**	-1169,00	-1038,00	-1098,00	-1037,00	**-1057,67**	-2098,00	-2051,00	-2086,00	-2349,00	**-2162,00**
7	-717,92	-867,28	-1124,00	-896,54	**-962,61**	-1248,00	-1056,00	-1061,00	-1061,00	**-1059,33**	-1466,00	-2162,00	-2122,00	-2088,00	**-2124,00**
8	-1298,00	-1072,00	-1058,00	-1326,00	**-1152,00**	-1045,00	-1117,00	-1280,00	-1097,00	**-1164,67**	-2230,00	-2184,00	-2245,00	-2124,00	**-2184,33**
9	-840,06	-908,15	-1033,00	-1214,00	**-1051,72**	-1257,00	-1252,00	-1214,00	-1168,00	**-1211,33**	-2622,00	-2422,00	-2074,00	-1994,00	**-2163,33**
10	-893,26	-1099,00	-939,95	-961,62	**-1000,19**	-946,03	-1009,00	-898,36	-1210,00	**-1039,12**	-2151,00	-1896,00	-2166,00	-2173,00	**-2078,33**
11	-1551,00	-1347,00	-1074,00	-1120,00	**-1180,33**	-1255,00	-1377,00	-1143,00	-1224,00	**-1248,00**	-2837,00	-2336,00	-2523,00	-2553,00	**-2470,67**
12	-1218,00	-1242,00	-1057,00	-1297,00	**-1198,67**	-1232,00	-1237,00	-1136,00	-1190,00	**-1187,67**	-2198,00	-2203,00	-2529,00	-2542,00	**-2424,67**
Mean	-1008,82	-1113,61	-1056,83	-1090,25	**-1086,90**	-1142,48	-1147,16	-1079,13	-1128,51	**-1118,27**	-2274,17	-2227,83	-2276,75	-2294,50	**-2266,36**
SD	269,21	159,53	72,95	144,38	**88,99**	129,61	124,80	149,88	139,58	**106,49**	352,78	244,55	177,74	227,35	**187,63**
SEM	77,81	46,11	21,08	41,73	25,72	37,46	36,07	43,32	40,34	30,78	101,96	70,68	51,37	65,71	54,23

Tab. 2 Effects of Placebo on pyramidal cell activity in terms of changes of population spike amplitudes. Results from single slices as obtained after single stimuli (SS) or after burst stimuli (TBS). Overview on final results after averaging 12 slices. SD=standard deviation; SEM=standard error of mean. ACSF=artificial cerebro-spinal fluid.

NCAG 1517															
slice	ACSF +SS					RR-EUR-S 5.00 mg/l + SS					RR-EUR-S 5.00 mg/l + TBS				
time [min]	10	20	30	40	Mean 20-40	50	60	70	80	Mean 60-80	90	100	110	120	Mean 100-120
1	-993,96	-1138,00	-872,96	-1025,00	**-1011,99**	-1138,00	-1430,00	-1577,00	-1882,00	**-1629,67**	-2690,00	-2709,00	-3018,00	-3332,00	**-3019,67**
2	-582,41	-993,62	-1065,00	-1091,00	**-1049,87**	-1267,00	-1432,00	-1505,00	-1407,00	**-1448,00**	-2726,00	-3142,00	-3141,00	-2967,00	**-3083,33**
3	-1150,00	-1036,00	-960,99	-992,00	**-996,33**	-1047,00	-1214,00	-1326,00	-1293,00	**-1277,67**	-2154,00	-2911,00	-2529,00	-3117,00	**-2852,33**
4	-1207,00	-1123,00	-1109,00	-1129,00	**-1120,33**	-1302,00	-1703,00	-1672,00	-1681,00	**-1685,33**	-3268,00	-3467,00	-3365,00	-3153,00	**-3328,33**
Mean	-983,34	-1072,66	-1001,99	-1059,25	**-1044,63**	-1188,50	-1444,75	-1520,00	-1565,75	**-1510,17**	-2709,50	-3057,25	-3013,25	-3142,25	**-3070,92**
SD	282,05	69,27	106,07	62,10	**55,25**	117,78	200,27	146,30	266,39	**185,19**	455,03	325,45	353,35	149,97	**197,33**
SEM	141,03	34,63	53,03	31,05	27,62	58,89	100,13	73,15	133,19	92,59	227,51	162,72	176,68	74,98	98,67
P<	n.s.	n.s.	n.s.	n.s.	n.s.	n.s.	0.02	0.01	0.01	0.01	n.s.	0.01	0.01	0.01	0.01

Tab. 3 Effects of RR-Eur-S 5.00 mg/l on pyramidal cell activity in terms of changes of population spike amplitudes. Results from single slices as obtained after single stimuli (SS) or after burst stimuli (TBS). Overview on final results after averaging 4 slices. SD=standard deviation; SEM=standard error of mean. P<=Wilcoxon Mann Whitny U-Test. ACSF=artificial cerebro-spinal fluid.

Report NCAG 15/17 H – Hippocampus slice preparation

NCAG 1517

slice	ACSF +SS					RR-EUR-S 10.0 mg/l + SS					RR-EUR-S 10.0 mg/l + TBS				
time [min]	10	20	30	40	Mean 20-40	50	60	70	80	Mean 60-80	90	100	110	120	Mean 100-120
1	-1283,00	-944,85	-987,44	-1040,00	**-990,76**	-1556,00	-1609,00	-1557,00	-1764,00	**-1643,33**	-3243,00	-4114,00	-4064,00	-4399,00	**-4192,33**
2	-1097,00	-981,47	-983,71	-975,02	**-980,07**	-1551,00	-1691,00	-1814,00	-1783,00	**-1762,67**	-4050,00	-3841,00	-3930,00	-3384,00	**-3718,33**
3	-961,01	-1215,00	-1093,00	-1080,00	**-1129,33**	-1319,00	-1912,00	-2054,00	-2034,00	**-2000,00**	-3450,00	-3378,00	-3629,00	-3568,00	**-3525,00**
4	-916,41	-1082,00	-951,88	-938,57	**-990,82**	-976,58	-1881,00	-1810,00	-1895,00	**-1862,00**	-3392,00	-3433,00	-3712,00	-3328,00	**-3491,00**
Mean	-1064,36	-1055,83	-1004,01	-1008,40	**-1022,75**	-1350,65	-1773,25	-1808,75	-1869,00	**-1817,00**	-3533,75	-3691,50	-3833,75	-3669,75	**-3731,67**
SD	164,76	120,92	61,44	63,55	**71,24**	272,79	146,75	202,94	124,26	**151,25**	355,04	349,27	199,19	496,86	**323,02**
SEM	82,38	60,46	30,72	31,77	35,62	136,39	73,37	101,47	62,13	75,62	177,52	174,63	99,59	248,43	161,51
P<	n.s.	n.s.	n.s.	n.s.	n.s.	0.01	0.01	0.01	0.01	0.01	0.01	0.01	0.01	0.01	0.01

Tab. 4 Effects of RR-Eur-S 10.0 mg/l on pyramidal cell activity in terms of changes of population spike amplitudes. Results from single slices as obtained after single stimuli (SS) or after burst stimuli (TBS). Overview on final results after averaging 4 slices. SD=standard deviation; SEM=standard error of mean. P<=Wilcoxon Mann Whitny U-Test. ACSF=artificial cerebro-spinal fluid.

NCAG 1517

slice	ACSF +SS					RR-EUR-S 20.0 mg/l + SS					RR-EUR-S 20.0 mg/l + TBS				
time [min]	10	20	30	40	Mean 20-40	50	60	70	80	Mean 60-80	90	100	110	120	Mean 100-120
1	-1080,00	-1107,00	-1052,00	-1075,00	**-1078,00**	-2416,00	-2095,00	-2015,00	-1953,00	**-2021,00**	-4582,00	-4889,00	-3868,00	-4119,00	**-4292,00**
2	-1000,00	-1297,00	-1089,00	-1067,00	**-1151,00**	-2051,00	-1963,00	-2116,00	-2017,00	**-2032,00**	-4635,00	-4448,00	-4463,00	-4321,00	**-4410,67**
3	-927,26	-940,89	-1033,00	-1048,00	**-1007,30**	-1947,00	-1840,00	-2069,00	-1875,00	**-1928,00**	-3261,00	-4285,00	-4127,00	-4478,00	**-4296,67**
4	-831,72	-1293,00	-1001,00	-851,34	**-1048,45**	-2186,00	-2221,00	-1960,00	-1962,00	**-2047,67**	-4123,00	-4751,00	-3983,00	-4140,00	**-4291,33**
Mean	-959,75	-1159,47	-1043,75	-1010,34	**-1071,19**	-2150,00	-2029,75	-2040,00	-1951,75	**-2007,17**	-4150,25	-4593,25	-4110,25	-4264,50	**-4322,67**
SD	105,72	170,56	36,78	106,60	**60,60**	202,54	164,62	67,43	58,47	**53,90**	635,85	275,96	257,93	168,77	**58,71**
SEM	52,86	85,28	18,39	53,30	30,30	101,27	82,31	33,72	29,23	26,95	317,92	137,98	128,97	84,38	29,36
P<	n.s.	n.s.	n.s.	n.s.	n.s.	0.01	0.01	0.01	0.01	0.01	0.01	0.01	0.01	0.01	0.01

Tab. 5 Effects of RR-Eur-S 20.0 mg/l on pyramidal cell activity in terms of changes of population spike amplitudes. Results from single slices as obtained after single stimuli (SS) or after burst stimuli (TBS). Overview on final results after averaging 4 slices. SD=standard deviation; SEM=standard error of mean. P<=Wilcoxon Mann Whitny U-Test. ACSF=artificial cerebro-spinal fluid.

NeuroCode AG
Sportparkstr. 9
D-35578 Wetzlar / Germany

EuroPharm, Inc
955 Challenger Drive, Green Bay
Wisconsin 54311, USA

Report NCAG 15/17 H – Hippocampus slice preparation

NCAG 1517															
**slice**	**ACSF +SS**					**RR-EUR-S 30.0 mg/l + SS**					**RR-EUR-S 30.0 mg/l + TBS**				
**time [min]**	**10**	**20**	**30**	**40**	**Mean 20-40**	**50**	**60**	**70**	**80**	**Mean 60-80**	**90**	**100**	**110**	**120**	**Mean 100-120**
**1**	-955,10	-971,79	-1036,00	-1052,00	**-1019,93**	-1636,00	-2014,00	-2079,00	-2111,00	**-2068,00**	-4031,00	-4417,00	-4567,00	-4576,00	**-4520,00**
**2**	-941,41	-990,72	-1016,00	-939,85	**-982,19**	-1845,00	-1999,00	-2151,00	-2119,00	**-2089,67**	-3720,00	-4210,00	-4247,00	-4251,00	**-4236,00**
**3**	-863,39	-1029,00	-1120,00	-978,62	**-1042,54**	-1778,00	-2041,00	-2194,00	-2297,00	**-2177,33**	-3981,00	-4601,00	-3739,00	-3958,00	**-4099,33**
**4**	-904,73	-1200,00	-1039,00	-1001,00	**-1080,00**	-2512,00	-2216,00	-2060,00	-2351,00	**-2209,00**	-3148,00	-4380,00	-4226,00	-4326,00	**-4310,67**
**Mean**	-916,16	-1047,88	-1052,75	-992,87	**-1031,17**	-1942,75	-2067,50	-2121,00	-2219,50	**-2136,00**	-3720,00	-4402,00	-4194,75	-4277,75	**-4291,50**
**SD**	41,11	104,17	45,98	46,82	**40,98**	389,38	100,51	62,49	122,71	**67,84**	404,98	160,39	341,56	254,45	**175,68**
**SEM**	20,55	52,08	22,99	23,41	20,49	194,69	50,26	31,24	61,35	33,92	202,49	80,19	170,78	127,23	87,84
**P<**	n.s.	n.s.	n.s.	n.s.	n.s.	0.01	0.01	0.01	0.01	0.01	0.01	0.01	0.01	0.01	0.01

Tab. 6 Effects of RR-Eur-S 30.0 mg/l on pyramidal cell activity in terms of changes of population spike amplitudes. Results from single slices as obtained after single stimuli (SS) or after burst stimuli (TBS). Overview on final results after averaging 4 slices. SD=standard deviation; SEM=standard error of mean. P<=Wilcoxon Mann Whitny U-Test. ACSF=artificial cerebro-spinal fluid.

NCAG 1517															
**slice**	**ACSF +SS**					**RR-Chi-R 5.00 mg/l + SS**					**RR-Chi-R 5.00 mg/l + TBS**				
**time [min]**	**10**	**20**	**30**	**40**	**Mean 20-40**	**50**	**60**	**70**	**80**	**Mean 60-80**	**90**	**100**	**110**	**120**	**Mean 100-120**
**1**	-981,93	-1039,00	-985,49	-1263,00	**-1095,83**	-937,37	-1167,00	-1197,00	-1168,00	**-1177,33**	-1746,00	-2033,00	-2048,00	-2070,00	**-2050,33**
**2**	-1076,00	-1027,00	-1030,00	-1153,00	**-1070,00**	-1292,00	-1166,00	-1068,00	-1188,00	**-1140,67**	-1534,00	-1844,00	-2034,00	-1997,00	**-1958,33**
**3**	-1113,00	-968,66	-964,67	-1023,00	**-985,44**	-1047,00	-1144,00	-1158,00	-1121,00	**-1141,00**	-1991,00	-2185,00	-2083,00	-2093,00	**-2120,33**
**4**	-1032,00	-1162,00	-1122,00	-1200,00	**-1161,33**	-1177,00	-1277,00	-1314,00	-1248,00	**-1279,67**	-1837,00	-2596,00	-2537,00	-2650,00	**-2594,33**
**Mean**	-1050,73	-1049,17	-1025,54	-1159,75	**-1078,15**	-1113,34	-1188,50	-1184,25	-1181,25	**-1184,67**	-1777,00	-2164,50	-2175,50	-2202,50	**-2180,83**
**SD**	56,57	81,26	69,84	101,70	**72,79**	154,21	59,95	101,98	52,62	**65,63**	190,97	319,70	241,88	301,13	**283,54**
**SEM**	28,28	40,63	34,92	50,85	36,39	77,10	29,97	50,99	26,31	32,81	95,48	159,85	120,94	150,56	141,77
**P<**	n.s.	n.s.	n.s.	n.s.	n.s.	n.s.	n.s.	n.s.	n.s.	n.s.	0.10	n.s.	n.s.	n.s.	n.s.

Tab. 7 Effects of RR-Chi-R 5.00 mg/l on pyramidal cell activity in terms of changes of population spike amplitudes. Results from single slices as obtained after single stimuli (SS) or after burst stimuli (TBS). Overview on final results after averaging 4 slices. SD=standard deviation; SEM=standard error of mean. P<=Wilcoxon Mann Whitny U-Test. ACSF=artificial cerebro-spinal fluid.

Report NCAG 15/17 H – Hippocampus slice preparation

NCAG 1517															
**slice**	**ACSF +SS**					**RR-Chi-R 10.0 mg/l + SS**					**RR-Chi-R 10.0 mg/l + TBS**				
**time [min]**	**10**	**20**	**30**	**40**	**Mean 20-40**	**50**	**60**	**70**	**80**	**Mean 60-80**	**90**	**100**	**110**	**120**	**Mean 100-120**
**1**	-934,37	-964,00	-992,25	-1126,00	**-1027,42**	-1425,00	-1370,00	-1351,00	-1375,00	**-1365,33**	-2197,00	-2594,00	-2557,00	-2969,00	**-2706,67**
**2**	-798,04	-1246,00	-1051,00	-985,64	**-1094,21**	-1634,00	-1657,00	-1737,00	-1735,00	**-1709,67**	-3304,00	-2572,00	-2704,00	-2940,00	**-2738,67**
**3**	-890,93	-948,49	-1139,00	-1152,00	**-1079,83**	-957,18	-957,18	-1572,00	-1633,00	**-1387,39**	-2235,00	-2669,00	-2503,00	-2853,00	**-2675,00**
**4**	-918,78	-954,73	-1079,00	-1094,00	**-1042,58**	-1351,00	-1288,00	-1534,00	-1404,00	**-1408,67**	-2878,00	-2605,00	-2767,00	-2816,00	**-2729,33**
**Mean**	-885,53	-1028,31	-1065,31	-1089,41	**-1061,01**	-1341,80	-1318,05	-1548,50	-1536,75	**-1467,77**	-2653,50	-2610,00	-2632,75	-2894,50	**-2712,42**
**SD**	61,03	145,27	60,99	73,13	**31,22**	283,03	287,93	158,43	175,45	**162,24**	534,50	41,66	123,39	71,89	**28,33**
**SEM**	30,52	72,63	30,50	36,57	15,61	141,52	143,97	79,22	87,73	81,12	267,25	20,83	61,69	35,95	14,17
**P<**	n.s.	n.s.	n.s.	n.s.	n.s.	n.s.	n.s.	0.01	0.01	0.02	n.s.	0.02	0.02	0.01	0.01

Tab. 8 Effects of RR-Chi-R 10.0 mg/l on pyramidal cell activity in terms of changes of population spike amplitudes. Results from single slices as obtained after single stimuli (SS) or after burst stimuli (TBS). Overview on final results after averaging 4 slices. SD=standard deviation; SEM=standard error of mean. P<=Wilcoxon Mann Whitny U-Test. ACSF=artificial cerebro-spinal fluid.

NCAG 1517															
**slice**	**ACSF +SS**					**RR-Chi-R 20.0 mg/kg + SS**					**RR-Chi-R 20.0 mg/kg + TBS**				
**time [min]**	**10**	**20**	**30**	**40**	**Mean 20-40**	**50**	**60**	**70**	**80**	**Mean 60-80**	**90**	**100**	**110**	**120**	**Mean 100-120**
**1**	-1063,00	-929,31	-1086,00	-1092,00	**-1035,77**	-1483,00	-1855,00	-2034,00	-2095,00	**-1994,67**	-3630,00	-3642,00	-3451,00	-3414,00	**-3502,33**
**2**	-1019,00	-1151,00	-1073,00	-1240,00	**-1154,67**	-1360,00	-1998,00	-1969,00	-1869,00	**-1945,33**	-3631,00	-3378,00	-3180,00	-3257,00	**-3271,67**
**3**	-910,34	-933,75	-1067,00	-931,79	**-977,51**	-1701,00	-2126,00	-2075,00	-2152,00	**-2117,67**	-3546,00	-3267,00	-3179,00	-3255,00	**-3233,67**
**4**	-814,94	-972,50	-1215,00	-1206,00	**-1131,17**	-1818,00	-1941,00	-2036,00	-1904,00	**-1960,33**	-3250,00	-3247,00	-3476,00	-3325,00	**-3349,33**
**Mean**	-951,82	-996,64	-1110,25	-1117,45	**-1074,78**	-1590,50	-1980,00	-2028,50	-2005,00	**-2004,50**	-3514,25	-3383,50	-3321,50	-3312,75	**-3339,25**
**SD**	111,55	104,72	70,28	139,02	**82,75**	207,09	113,70	43,93	139,53	**78,22**	180,61	181,71	164,29	74,93	**118,90**
**SEM**	55,78	52,36	35,14	69,51	41,38	103,54	56,85	21,96	69,77	39,11	90,31	90,86	82,14	37,47	59,45
**P<**	n.s.	n.s.	n.s.	n.s.	n.s.	0.01	0.01	0.01	0.01	0.01	0.01	0.01	0.01	0.01	0.01

Tab. 9 Effects of RR-Chi-R 20.0 mg/l on pyramidal cell activity in terms of changes of population spike amplitudes. Results from single slices as obtained after single stimuli (SS) or after burst stimuli (TBS). Overview on final results after averaging 4 slices. SD=standard deviation; SEM=standard error of mean. P<=Wilcoxon Mann Whitny U-Test. ACSF=artificial cerebro-spinal fluid.

Report NCAG 15/17 H – Hippocampus slice preparation

NCAG 1517															
**slice**	**ACSF +SS**					**RR-Chi-R 30.0 mg/kg + SS**					**RR-Chi-R 30.0 mg/kg + TBS**				
**time [min]**	**10**	**20**	**30**	**40**	**Mean 20-40**	**50**	**60**	**70**	**80**	**Mean 60-80**	**90**	**100**	**110**	**120**	**Mean 100-120**
**1**	-1005,00	-1023,00	-1081,00	-1031,00	**-1045,00**	-2248,00	-2644,00	-2508,00	-2366,00	**-2506,00**	-3224,00	-4444,00	-4254,00	-4874,00	**-4524,00**
**2**	-385,34	-937,85	-1008,00	-1054,00	**-999,95**	-1588,00	-1951,00	-1907,00	-2026,00	**-1961,33**	-4189,00	-4068,00	-4005,00	-4463,00	**-4178,67**
**3**	-627,23	-1074,00	-1089,00	-1003,00	**-1055,33**	-1699,00	-2368,00	-2437,00	-2444,00	**-2416,33**	-3383,00	-3779,00	-4201,00	-3809,00	**-3929,67**
**4**	-1002,00	-916,34	-1082,00	-1124,00	**-1040,78**	-2226,00	-2327,00	-2187,00	-2286,00	**-2266,67**	-3306,00	-3892,00	-3880,00	-3870,00	**-3880,67**
**Mean**	-754,89	-987,80	-1065,00	-1053,00	**-1035,27**	-1940,25	-2322,50	-2259,75	-2280,50	**-2287,58**	-3525,50	-4045,75	-4085,00	-4254,00	**-4128,25**
**SD**	303,58	73,65	38,17	51,72	**24,32**	345,76	284,88	272,50	181,51	**238,86**	447,07	290,91	173,63	507,79	**294,33**
**SEM**	151,79	36,82	19,08	25,86	12,16	172,88	142,44	136,25	90,76	119,43	223,54	145,46	86,81	253,90	147,17
**P<**	n.s.	n.s.	n.s.	n.s.	n.s.	0.01	0.01	0.01	0.01	0.01	0.01	0.01	0.01	0.01	0.01

Tab. 10 Effects of RR-RR-Chi-R 30.0 mg/l on pyramidal cell activity in terms of changes of population spike amplitudes. Results from single slices as obtained after single stimuli (SS) or after burst stimuli (TBS). Overview on final results after averaging 4 slices. SD=standard deviation; SEM=standard error of mean. P<=Wilcoxon Mann Whitny U-Test. ACSF=artificial cerebro-spinal fluid.

NCAG 1517															
**slice**	**ACSF +SS**					**RR-Chi-S 5.00 mg/l + SS**					**RR-Chi-S 5.00 mg/l + TBS**				
**time [min]**	**10**	**20**	**30**	**40**	**Mean 20-40**	**50**	**60**	**70**	**80**	**Mean 60-80**	**90**	**100**	**110**	**120**	**Mean 100-120**
**1**	-886,25	-1169,00	-891,34	-908,74	**-989,69**	-1083,00	-1155,00	-960,93	-1079,00	**-1064,98**	-1979,00	-2565,00	-2713,00	-2173,00	**-2483,67**
**2**	-615,72	-1055,00	-1005,00	-976,61	**-1012,20**	-1328,00	-1214,00	-1229,00	-1111,00	**-1184,67**	-2774,00	-2715,00	-2664,00	-2348,00	**-2575,67**
**3**	-1095,00	-906,56	-1047,00	-975,53	**-976,36**	-846,96	-1032,00	-1015,00	-1256,00	**-1101,00**	-2223,00	-2026,00	-2584,00	-2345,00	**-2318,33**
**4**	-666,50	-859,61	-1025,00	-1038,00	**-974,20**	-1363,00	-1230,00	-1240,00	-1073,00	**-1181,00**	-1969,00	-2153,00	-2511,00	-2460,00	**-2374,67**
**Mean**	-815,87	-997,54	-992,09	-974,72	**-988,12**	-1155,24	-1157,75	-1111,23	-1129,75	**-1132,91**	-2236,25	-2364,75	-2618,00	-2331,50	**-2438,08**
**SD**	220,03	141,42	69,32	52,80	**17,46**	240,32	89,82	144,11	85,80	**59,51**	377,25	327,80	88,97	118,45	**114,56**
**SEM**	110,01	70,71	34,66	26,40	8,73	120,16	44,91	72,05	42,90	29,76	188,62	163,90	44,48	59,22	57,28
**P<**	n.s.	n.s.	n.s.	n.s.	n.s.	n.s.	n.s.	n.s.	n.s.	n.s.	n.s.	n.s.	n.s.	n.s.	n.s.

Tab. 11 Effects of RR-Chi-S 5.00 mg/l on pyramidal cell activity in terms of changes of population spike amplitudes. Results from single slices as obtained after single stimuli (SS) or after burst stimuli (TBS). Overview on final results after averaging 4 slices. SD=standard deviation; SEM=standard error of mean. P<=Wilcoxon Mann Whitny U-Test. ACSF=artificial cerebro-spinal fluid.

NeuroCode AG
Sportparkstr. 9
D-35578 Wetzlar / Germany

EuroPharm, Inc
955 Challenger Drive, Green Bay
Wisconsin 54311, USA

Report NCAG 15/17 H – Hippocampus slice preparation

NCAG 1517															
**slice**	**ACSF +SS**					**RR-Chi-S 10.0 mg/l + SS**					**RR-Chi-S 10.0 mg/l + TBS**				
**time [min]**	**10**	**20**	**30**	**40**	**Mean 20-40**	**50**	**60**	**70**	**80**	**Mean 60-80**	**90**	**100**	**110**	**120**	**Mean 100-120**
**1**	-1177,00	-874,38	-1005,00	-820,34	**-899,91**	-1068,00	-1538,00	-1238,00	-1076,00	**-1284,00**	-2702,00	-2551,00	-2424,00	-2598,00	**-2524,33**
**2**	-798,51	-1004,00	-1002,00	-980,50	**-995,50**	-1390,00	-1547,00	-1408,00	-1529,00	**-1494,67**	-3168,00	-3067,00	-2979,00	-3099,00	**-3048,33**
**3**	-664,81	-1126,00	-1044,00	-1044,00	**-1071,33**	-1694,00	-1780,00	-1739,00	-1991,00	**-1836,67**	-2951,00	-3069,00	-2720,00	-2580,00	**-2789,67**
**4**	-1135,00	-1230,00	-1087,00	-1087,00	**-1134,67**	-2191,00	-2238,00	-1789,00	-1934,00	**-1987,00**	-2924,00	-2894,00	-2769,00	-3073,00	**-2912,00**
**Mean**	-943,83	-1058,60	-1034,50	-982,96	**-1025,35**	-1585,75	-1775,75	-1543,50	-1632,50	**-1650,58**	-2936,25	-2895,25	-2723,00	-2837,50	**-2818,58**
**SD**	251,58	153,67	39,89	116,91	**101,15**	477,64	327,89	264,69	424,20	**319,63**	190,57	243,72	228,81	287,23	**222,81**
**SEM**	125,79	76,83	19,94	58,45	50,57	238,82	163,95	132,34	212,10	159,82	95,28	121,86	114,40	143,62	111,40
**P<**	n.s.	n.s.	n.s.	n.s.	n.s.	0.10	0.01	0.01	0.10	0.01	0.01	0.01	0.01	0.01	0.01

Tab. 12 Effects of RR-Chi-S 10.0 mg/l on pyramidal cell activity in terms of changes of population spike amplitudes. Results from single slices as obtained after single stimuli (SS) or after burst stimuli (TBS). Overview on final results after averaging 4 slices. SD=standard deviation; SEM=standard error of mean. P<=Wilcoxon Mann Whitny U-Test. ACSF=artificial cerebro-spinal fluid.

NCAG 1517															
**slice**	**ACSF +SS**					**RR-Chi-S 20.0 mg/l + SS**					**RR-Chi-S 20.0 mg/l + TBS**				
**time [min]**	**10**	**20**	**30**	**40**	**Mean 20-40**	**50**	**60**	**70**	**80**	**Mean 60-80**	**90**	**100**	**110**	**120**	**Mean 100-120**
**1**	-930,20	-1147,00	-1207,00	-1105,00	**-1153,00**	-1848,00	-2043,00	-1990,00	-1979,00	**-2004,00**	-2910,00	-4165,00	-3345,00	-3917,00	**-3809,00**
**2**	-1028,00	-944,73	-943,43	-1001,00	**-963,05**	-1320,00	-1958,00	-2000,00	-2016,00	**-1991,33**	-3735,00	-3675,00	-3785,00	-3765,00	**-3741,67**
**3**	-993,54	-953,11	-919,46	-967,43	**-946,67**	-1947,00	-2249,00	-2217,00	-2188,00	**-2218,00**	-3474,00	-3514,00	-3277,00	-3496,00	**-3429,00**
**4**	-997,12	-991,39	-846,37	-1108,00	**-981,92**	-2601,00	-1933,00	-2097,00	-2179,00	**-2069,67**	-3715,00	-3444,00	-3230,00	-3459,00	**-3377,67**
**Mean**	-987,22	-1009,06	-979,07	-1045,36	**-1011,16**	-1929,00	-2045,75	-2076,00	-2090,50	**-2070,75**	-3458,50	-3699,50	-3409,25	-3659,25	**-3589,33**
**SD**	41,04	94,18	157,46	71,93	**95,65**	525,79	143,45	105,66	108,51	**104,00**	384,42	325,05	254,91	219,37	**217,54**
**SEM**	20,52	47,09	78,73	35,96	47,83	262,89	71,72	52,83	54,25	52,00	192,21	162,53	127,45	109,68	108,77
**P<**	n.s.	n.s.	n.s.	n.s.	n.s.	0.01	0.01	0.01	0.01	0.01	0.01	0.01	0.01	0.01	0.01

Tab. 13 Effects of RR-Chi-S 20.0 mg/l on pyramidal cell activity in terms of changes of population spike amplitudes. Results from single slices as obtained after single stimuli (SS) or after burst stimuli (TBS). Overview on final results after averaging 4 slices. SD=standard deviation; SEM=standard error of mean. P<=Wilcoxon Mann Whitny U-Test. ACSF=artificial cerebro-spinal fluid.

Report NCAG 15/17 H – Hippocampus slice preparation

NCAG 1517															
**slice**	**ACSF +SS**					**RR-Chi-S 30.0 mg/l + SS**					**RR-Chi-S 30.0 mg/l + TBS**				
**time [min]**	**10**	**20**	**30**	**40**	**Mean 20-40**	**50**	**60**	**70**	**80**	**Mean 60-80**	**90**	**100**	**110**	**120**	**Mean 100-120**
**1**	-886,37	-966,35	-985,26	-1060,00	**-1003,87**	-1250,00	-1788,00	-1971,00	-2039,00	**-1932,67**	-3063,00	-4132,00	-4085,00	-3711,00	**-3976,00**
**2**	-1350,00	-1108,00	-953,25	-1025,00	**-1028,75**	-2152,00	-2401,00	-2220,00	-2140,00	**-2253,67**	-4279,00	-4227,00	-4377,00	-4042,00	**-4215,33**
**3**	-578,02	-1285,00	-1147,00	-810,61	**-1080,87**	-2673,00	-2418,00	-2373,00	-2170,00	**-2320,33**	-3378,00	-3719,00	-3843,00	-3980,00	**-3847,33**
**4**	-882,84	-955,01	-816,85	-1141,00	**-970,95**	-2253,00	-1980,00	-2128,00	-2104,00	**-2070,67**	-3522,00	-4775,00	-4285,00	-4531,00	**-4530,33**
**Mean**	-924,31	-1078,59	-975,59	-1009,15	**-1021,11**	-2082,00	-2146,75	-2173,00	-2113,25	**-2144,33**	-3560,50	-4213,25	-4147,50	-4066,00	**-4142,25**
**SD**	318,48	154,21	135,61	141,00	**46,34**	598,79	313,44	168,36	56,38	**176,22**	515,93	434,60	236,79	341,67	**300,31**
**SEM**	159,24	77,10	67,81	70,50	23,17	299,39	156,72	84,18	28,19	88,11	257,96	217,30	118,39	170,84	150,16
**P<**	n.s.	n.s.	n.s.	n.s.	n.s.	0.01	0.01	0.01	0.01	0.01	0.01	0.01	0.01	0.01	0.01

Tab. 14 Effects of RR-Chi-S 30.0 mg/l on pyramidal cell activity in terms of changes of population spike amplitudes. Results from single slices as obtained after single stimuli (SS) or after burst stimuli (TBS). Overview on final results after averaging 4 slices. SD=standard deviation; SEM=standard error of mean. P<=Wilcoxon Mann Whitny U-Test. ACSF=artificial cerebro-spinal fluid.

NCAG 1517															
**slice**	**ACSF +SS**					**RR-Alt-S 5.00 mg/l + SS**					**RR-Alt-S 5.00 mg/l + TBS**				
**time [min]**	**10**	**20**	**30**	**40**	**Mean 20-40**	**50**	**60**	**70**	**80**	**Mean 60-80**	**90**	**100**	**110**	**120**	**Mean 100-120**
**1**	-786,75	-922,18	-994,67	-1066,00	**-994,28**	-961,66	-1146,00	-1285,00	-1208,00	**-1213,00**	-2305,00	-3191,00	-2651,00	-2754,00	**-2865,33**
**2**	-787,85	-1127,00	-1230,00	-1049,00	**-1135,33**	-1116,00	-1094,00	-1287,00	-1256,00	**-1212,33**	-3221,00	-2841,00	-2532,00	-2827,00	**-2733,33**
**3**	-957,16	-918,04	-988,77	-1143,00	**-1016,60**	-1277,00	-1393,00	-1274,00	-1305,00	**-1324,00**	-2747,00	-2938,00	-2335,00	-2708,00	**-2660,33**
**4**	-933,47	-1059,00	-1090,00	-1117,00	**-1088,67**	-1188,00	-1363,00	-1272,00	-1332,00	**-1322,33**	-2486,00	-2574,00	-2996,00	-2706,00	**-2758,67**
**Mean**	-866,31	-1006,56	-1075,86	-1093,75	**-1058,72**	-1135,67	-1249,00	-1279,50	-1275,25	**-1267,92**	-2689,75	-2886,00	-2628,50	-2748,75	**-2754,42**
**SD**	91,74	103,62	112,75	43,74	**65,04**	133,39	150,96	7,59	54,77	**63,80**	397,93	255,02	277,50	56,68	**84,89**
**SEM**	45,87	51,81	56,37	21,87	32,52	66,70	75,48	3,80	27,38	31,90	198,97	127,51	138,75	28,34	42,44
**P<**	n.s.	n.s.	n.s.	n.s.	n.s.	n.s.	n.s.	0.10	0.10	0.10	0.10	0.01	0.02	0.01	0.01

Tab. 15 Effects of RR-Alt-S 5.00 mg/l on pyramidal cell activity in terms of changes of population spike amplitudes. Results from single slices as obtained after single stimuli (SS) or after burst stimuli (TBS). Overview on final results after averaging 4 slices. SD=standard deviation; SEM=standard error of mean. P<=Wilcoxon Mann Whitny U-Test. ACSF=artificial cerebro-spinal fluid.

Report NCAG 15/17 H – Hippocampus slice preparation

NCAG 1517															
**slice**	**ACSF +SS**					**RR-Alt-S 10.0 mg/l + SS**					**RR-Alt-S 10.0 mg/l + TBS**				
**time [min]**	**10**	**20**	**30**	**40**	**Mean 20-40**	**50**	**60**	**70**	**80**	**Mean 60-80**	**90**	**100**	**110**	**120**	**Mean 100-120**
**1**	-1095,00	-941,46	-1058,00	-1030,00	**-1009,82**	-1193,00	-1697,00	-2017,00	-1584,00	**-1766,00**	-2673,00	-3716,00	-3516,00	-3059,00	**-3430,33**
**2**	-999,93	-938,70	-1157,00	-1132,00	**-1075,90**	-1352,00	-1682,00	-1919,00	-1583,00	**-1728,00**	-2892,00	-3385,00	-3852,00	-4248,00	**-3828,33**
**3**	-1042,00	-1118,00	-1099,00	-1186,00	**-1134,33**	-1640,00	-1732,00	-1632,00	-1670,00	**-1678,00**	-2496,00	-3046,00	-3103,00	-3586,00	**-3245,00**
**4**	-1135,00	-914,91	-1176,00	-1245,00	**-1111,97**	-1725,00	-1637,00	-1681,00	-1687,00	**-1668,33**	-2926,00	-3276,00	-2962,00	-3410,00	**-3216,00**
**Mean**	-1067,98	-978,27	-1122,50	-1148,25	**-1083,01**	-1477,50	-1687,00	-1812,25	-1631,00	**-1710,08**	-2746,75	-3355,75	-3358,25	-3575,75	**-3429,92**
**SD**	59,24	93,91	54,05	91,35	**54,41**	247,89	39,37	185,32	55,29	**45,53**	201,28	278,65	404,49	498,84	**282,07**
**SEM**	29,62	46,96	27,03	45,67	27,20	123,95	19,69	92,66	27,64	22,77	100,64	139,33	202,25	249,42	141,04
**P<**	n.s.	n.s.	n.s.	n.s.	n.s.	0.02	0.01	0.01	0.01	0.01	0.02	0.01	0.01	0.01	0.01

Tab. 16 Effects of RR-Alt-S 10.0 mg/l on pyramidal cell activity in terms of changes of population spike amplitudes. Results from single slices as obtained after single stimuli (SS) or after burst stimuli (TBS). Overview on final results after averaging 4 slices. SD=standard deviation; SEM=standard error of mean. P<=Wilcoxon Mann Whitny U-Test. ACSF=artificial cerebro-spinal fluid.

NCAG 1517															
**slice**	**ACSF +SS**					**RR-Alt-S 20.0 mg/l + SS**					**RR-Alt-S 20.0 mg/l + TBS**				
**time [min]**	**10**	**20**	**30**	**40**	**Mean 20-40**	**50**	**60**	**70**	**80**	**Mean 60-80**	**90**	**100**	**110**	**120**	**Mean 100-120**
**1**	-742,36	-936,46	-971,52	-979,71	**-962,56**	-1513,00	-1968,00	-1777,00	-1974,00	**-1906,33**	-3896,00	-4523,00	-4713,00	-4807,00	**-4681,00**
**2**	-1190,00	-1156,00	-1166,00	-1002,00	**-1108,00**	-2179,00	-1886,00	-2540,00	-2157,00	**-2194,33**	-3613,00	-4055,00	-4198,00	-3881,00	**-4044,67**
**3**	-1081,00	-1082,00	-1195,00	-1065,00	**-1114,00**	-1428,00	-1795,00	-1929,00	-1840,00	**-1854,67**	-3433,00	-3675,00	-4021,00	-3736,00	**-3810,67**
**4**	-1145,00	-1085,00	-901,19	-1212,00	**-1066,06**	-1629,00	-2344,00	-2461,00	-2253,00	**-2352,67**	-4166,00	-4381,00	-4034,00	-3887,00	**-4100,67**
**Mean**	-1039,59	-1064,87	-1058,43	-1064,68	**-1062,66**	-1687,25	-1998,25	-2176,75	-2056,00	**-2077,00**	-3777,00	-4158,50	-4241,50	-4077,75	**-4159,25**
**SD**	203,14	92,18	144,34	104,65	**70,05**	338,03	241,09	380,32	184,74	**236,87**	321,83	377,21	324,49	491,15	**369,82**
**SEM**	101,57	46,09	72,17	52,32	35,03	169,01	120,54	190,16	92,37	118,43	160,91	188,60	162,24	245,58	184,91
**P<**	n.s.	n.s.	n.s.	n.s.	n.s.	0.01	0.01	0.01	0.01	0.01	0.01	0.01	0.01	0.01	0.01

Tab. 17 Effects of RR-Alt-S 20.0 mg/l on pyramidal cell activity in terms of changes of population spike amplitudes. Results from single slices as obtained after single stimuli (SS) or after burst stimuli (TBS). Overview on final results after averaging 4 slices. SD=standard deviation; SEM=standard error of mean. P<=Wilcoxon Mann Whitny U-Test. ACSF=artificial cerebro-spinal fluid.

Report NCAG 15/17 H – Hippocampus slice preparation

NCAG 1517															
**slice**	**ACSF +SS**					**RR-Alt-S 30.0 mg/l + SS**					**RR-Alt-S 30.0 mg/l + TBS**				
**time [min]**	**10**	**20**	**30**	**40**	**Mean 20-40**	**50**	**60**	**70**	**80**	**Mean 60-80**	**90**	**100**	**110**	**120**	**Mean 100-120**
**1**	-626,62	-968,88	-1221,00	-1039,00	**-1076,29**	-2183,00	-2112,00	-1950,00	-2151,00	**-2071,00**	-4245,00	-4126,00	-4426,00	-4485,00	**-4345,67**
**2**	-1258,00	-1056,00	-1211,00	-1173,00	**-1146,67**	-2713,00	-2577,00	-2249,00	-2265,00	**-2363,67**	-4682,00	-4890,00	-4103,00	-4250,00	**-4414,33**
**3**	-949,86	-1189,00	-1162,00	-1171,00	**-1174,00**	-1538,00	-1707,00	-1963,00	-2290,00	**-1986,67**	-3156,00	-3730,00	-3788,00	-4243,00	**-3920,33**
**4**	-571,33	-862,93	-956,85	-1049,00	**-956,26**	-1531,00	-2274,00	-1977,00	-2138,00	**-2129,67**	-3426,00	-3957,00	-4122,00	-4071,00	**-4050,00**
**Mean**	-851,45	-1019,20	-1137,71	-1108,00	**-1088,31**	-1991,25	-2167,50	-2034,75	-2211,00	**-2137,75**	-3877,25	-4175,75	-4109,75	-4262,25	**-4182,58**
**SD**	318,32	138,01	123,30	74,02	**97,18**	570,08	362,48	143,26	77,64	**161,64**	708,68	503,05	260,60	170,01	**235,70**
**SEM**	159,16	69,00	61,65	37,01	48,59	285,04	181,24	71,63	38,82	80,82	354,34	251,52	130,30	85,01	117,85
**P<**	n.s.	n.s.	n.s.	n.s.	n.s.	0.01	0.01	0.01	0.01	0.01	0.01	0.01	0.01	0.01	0.01

Tab. 18 Effects of RR-Alt-S 30.0 mg/l on pyramidal cell activity in terms of changes of population spike amplitudes. Results from single slices as obtained after single stimuli (SS) or after burst stimuli (TBS). Overview on final results after averaging 4 slices. SD=standard deviation; SEM=standard error of mean. P<=Wilcoxon Mann Whitny U-Test. ACSF=artificial cerebro-spinal fluid.

NCAG 1517															
**slice**	**ACSF +SS**					**RR-Alt-B 5.00 mg/l + SS**					**RR-Alt-B 5.00 mg/l + TBS**				
**time [min]**	**10**	**20**	**30**	**40**	**Mean 20-40**	**50**	**60**	**70**	**80**	**Mean 60-80**	**90**	**100**	**110**	**120**	**Mean 100-120**
**1**	-788,99	-1090,00	-1211,00	-1178,00	**-1159,67**	-1021,00	-970,72	-1312,00	-1270,00	**-1184,24**	-1971,00	-2369,00	-2731,00	-2727,00	**-2609,00**
**2**	-708,16	-1062,00	-929,78	-1088,00	**-1026,59**	-1230,00	-1159,00	-1039,00	-898,22	**-1032,07**	-1971,00	-2388,00	-2344,00	-2291,00	**-2341,00**
**3**	-917,49	-1086,00	-1112,00	-1053,00	**-1083,67**	-1228,00	-1200,00	-1066,00	-1162,00	**-1142,67**	-2789,00	-2619,00	-2377,00	-2108,00	**-2368,00**
**4**	-1033,00	-1229,00	-1063,00	-1021,00	**-1104,33**	-1091,00	-1066,00	-1038,00	-1070,00	**-1058,00**	-2413,00	-2521,00	-2468,00	-2342,00	**-2443,67**
**Mean**	-861,91	-1116,75	-1078,95	-1085,00	**-1093,57**	-1142,50	-1098,93	-1113,75	-1100,06	**-1104,25**	-2286,00	-2474,25	-2480,00	-2367,00	**-2440,42**
**SD**	142,97	75,85	116,96	67,77	**54,98**	103,89	102,22	132,80	157,44	**71,23**	394,79	117,83	175,36	260,18	**120,50**
**SEM**	71,48	37,92	58,48	33,88	27,49	51,95	51,11	66,40	78,72	35,62	197,40	58,92	87,68	130,09	60,25
**P<**	n.s.	n.s.	n.s.	n.s.	n.s.	n.s.	n.s.	n.s.	n.s.	n.s.	n.s.	n.s.	n.s.	n.s.	n.s.

Tab. 19 Effects of RR-Alt-B 5.00 mg/l on pyramidal cell activity in terms of changes of population spike amplitudes. Results from single slices as obtained after single stimuli (SS) or after burst stimuli (TBS). Overview on final results after averaging 4 slices. SD=standard deviation; SEM=standard error of mean. P<=Wilcoxon Mann Whitny U-Test. ACSF=artificial cerebro-spinal fluid.

Report NCAG 15/17 H – Hippocampus slice preparation

NCAG 1517															
**slice**	**ACSF +SS**					**RR-Alt-B 10.0 mg/l + SS**					**RR-Alt-B 10.0 mg/l + TBS**				
**time [min]**	**10**	**20**	**30**	**40**	**Mean 20-40**	**50**	**60**	**70**	**80**	**Mean 60-80**	**90**	**100**	**110**	**120**	**Mean 100-120**
**1**	-1084,00	-989,45	-1084,00	-1181,00	**-1084,82**	-1252,00	-1390,00	-1365,00	-1566,00	**-1440,33**	-2535,00	-2964,00	-2534,00	-2326,00	**-2608,00**
**2**	-1083,00	-940,29	-1184,00	-1229,00	**-1117,76**	-727,76	-1503,00	-1615,00	-1560,00	**-1559,33**	-2636,00	-3148,00	-2933,00	-2446,00	**-2842,33**
**3**	-618,06	-985,37	-940,90	-967,85	**-964,71**	-1183,00	-1247,00	-1837,00	-1463,00	**-1515,67**	-3294,00	-2961,00	-2543,00	-2745,00	**-2749,67**
**4**	-802,69	-1020,00	-1090,00	-1146,00	**-1085,33**	-1529,00	-1767,00	-1730,00	-1744,00	*** -1747,00**	-2076,00	-2680,00	-3287,00	-3202,00	**-3056,33**
**Mean**	-896,94	-983,78	-1074,73	-1130,96	**-1063,16**	-1172,94	-1476,75	-1636,75	-1583,25	**-1505,11**	-2635,25	-2938,25	-2824,25	-2679,75	**-2814,08**
**SD**	228,23	32,85	100,28	113,94	**67,42**	332,32	220,03	202,58	117,10	**60,20**	502,25	193,11	360,24	390,21	**188,06**
**SEM**	114,12	16,43	50,14	56,97	33,71	166,16	110,02	101,29	58,55	34,80	251,12	96,55	180,12	195,10	94,03
**P<**	n.s.	n.s.	n.s.	n.s.	n.s.	n.s.	0.01	0.01	0.01	0.01	n.s.	0.01	0.01	n.s.	0.01

Tab. 20 Effects of RR-Alt-B 10.0 mg/l on pyramidal cell activity in terms of changes of population spike amplitudes. Results from single slices as obtained after single stimuli (SS) or after burst stimuli (TBS). Overview on final results after averaging 4 slices. SD=standard deviation; SEM=standard error of mean. P<=Wilcoxon Mann Whitny U-Test. ACSF=artificial cerebro-spinal fluid. Outlier ismarked by *.

NCAG 1517															
**slice**	**ACSF +SS**					**RR-Alt-B 20.0 mg/l + SS**					**RR-Alt-B 20.0 mg/l + TBS**				
**time [min]**	**10**	**20**	**30**	**40**	**Mean 20-40**	**50**	**60**	**70**	**80**	**Mean 60-80**	**90**	**100**	**110**	**120**	**Mean 100-120**
**1**	-727,89	-936,24	-898,24	-936,14	**-923,54**	-1774,00	-1652,00	-1954,00	-1646,00	**-1750,67**	-3278,00	-3988,00	-3418,00	-3433,00	**-3613,00**
**2**	-1261,00	-987,97	-1085,00	-1154,00	**-1075,66**	-1570,00	-1649,00	-1784,00	-1774,00	**-1735,67**	-2906,00	-3415,00	-3849,00	-3697,00	**-3653,67**
**3**	-658,37	-1090,00	-977,81	-1056,00	**-1041,27**	-1151,00	-1907,00	-1391,00	-1534,00	**-1610,67**	-3294,00	-3694,00	-3379,00	-3494,00	**-3522,33**
**4**	-813,16	-1037,00	-963,05	-908,65	**-969,57**	-1063,00	-1467,00	-1579,00	-2228,00	**-1758,00**	-3167,00	-3697,00	-3768,00	-3694,00	**-3719,67**
**Mean**	-865,11	-1012,80	-981,03	-1013,70	**-1002,51**	-1389,50	-1668,75	-1677,00	-1795,50	**-1713,75**	-3161,25	-3698,50	-3603,50	-3579,50	**-3627,17**
**SD**	271,42	65,89	77,45	113,32	**68,74**	338,58	180,87	244,66	304,55	**69,35**	179,29	233,96	239,54	136,25	**82,56**
**SEM**	135,71	32,94	38,73	56,66	34,37	169,29	90,43	122,33	152,27	34,67	89,65	116,98	119,77	68,12	41,28
**P<**	n.s.	n.s.	n.s.	n.s.	n.s.	0.01	0.01	0.01	0.01	0.01	0.01	0.01	0.01	0.01	0.01

Tab. 21 Effects of RR-Alt-B 20.0 mg/l on pyramidal cell activity in terms of changes of population spike amplitudes. Results from single slices as obtained after single stimuli (SS) or after burst stimuli (TBS). Overview on final results after averaging 4 slices. SD=standard deviation; SEM=standard error of mean. P<=Wilcoxon Mann Whitny U-Test. ACSF=artificial cerebro-spinal fluid.

Report NCAG 15/17 H – Hippocampus slice preparation

NCAG 1517															
**slice**	**ACSF +SS**					**RR-Alt-B 30.0 mg/l + SS**					**RR-Alt-B 30.0 mg/l + TBS**				
**time [min]**	**10**	**20**	**30**	**40**	**Mean 20-40**	**50**	**60**	**70**	**80**	**Mean 60-80**	**90**	**100**	**110**	**120**	**Mean 100-120**
**1**	-992,07	-994,81	-1145,00	-1269,00	**-1136,27**	-1649,00	-1970,00	-2233,00	-2243,00	**-2148,67**	-2559,00	-4011,00	-3816,00	-4095,00	**-3974,00**
**2**	-923,25	-1014,00	-901,29	-1139,00	**-1018,10**	-1909,00	-1872,00	-2123,00	-2269,00	**-2088,00**	-2902,00	-3787,00	-4130,00	-3843,00	**-3920,00**
**3**	-901,98	-912,75	-916,96	-834,48	**-888,06**	-1415,00	-1889,00	-2110,00	-1954,00	**-1984,33**	-4000,00	-4501,00	-3927,00	-3982,00	**-4136,67**
**4**	-742,86	-944,85	-1253,00	-1194,00	**-1130,62**	-2187,00	-2311,00	-2347,00	-2335,00	**-2331,00**	-3028,00	-4285,00	-3870,00	-4405,00	**-4186,67**
**Mean**	-890,04	-966,60	-1054,06	-1109,12	**-1043,26**	-1790,00	-2010,50	-2203,25	-2200,25	**-2138,00**	-3122,25	-4146,00	-3935,75	-4081,25	**-4054,33**
**SD**	105,38	46,24	173,19	190,69	**116,91**	332,80	204,85	110,58	168,67	**145,46**	617,82	312,22	137,20	239,18	**127,53**
**SEM**	52,69	23,12	86,59	95,34	58,45	166,40	102,42	55,29	84,34	72,73	308,91	156,11	68,60	119,59	63,76
**P<**	n.s.	n.s.	n.s.	n.s.	n.s.	0.01	0.01	0.01	0.01	0.01	0.01	0.01	0.01	0.01	0.01

Tab. 22 Effects of RR-Alt-B 30.0 mg/l on pyramidal cell activity in terms of changes of population spike amplitudes. Results from single slices as obtained after single stimuli (SS) or after burst stimuli (TBS). Overview on final results after averaging 4 slices. SD=standard deviation; SEM=standard error of mean. P<=Wilcoxon Mann Whitny U-Test. ACSF=artificial cerebro-spinal fluid.

NCAG 1517															
**slice**	**ACSF +SS**					**RR-Alt-X 5.00 mg/l + SS**					**RR-Alt-X 5.00 mg/l + TBS**				
**time [min]**	**10**	**20**	**30**	**40**	**Mean 20-40**	**50**	**60**	**70**	**80**	**Mean 60-80**	**90**	**100**	**110**	**120**	**Mean 100-120**
**1**	-445,19	-1188,00	-1127,00	-953,17	**-1089,39**	-1035,00	-1177,00	-1265,00	-1238,00	**-1226,67**	-1929,00	-2228,00	-2256,00	-2159,00	**-2214,33**
**2**	-747,12	-991,03	-1129,00	-954,39	**-1024,81**	-1607,00	-1154,00	-1392,00	-1420,00	**-1322,00**	-1794,00	-2087,00	-2189,00	-2326,00	**-2200,67**
**3**	-700,55	-1137,00	-1102,00	-1107,00	**-1115,33**	-1292,00	-1252,00	-1316,00	-1493,00	**-1353,67**	-1672,00	-2163,00	-2354,00	-2509,00	**-2342,00**
**4**	-1274,00	-732,24	-1037,00	-1015,00	**-928,08**	-725,14	-828,86	-1392,00	-1335,00	**-1185,29**	-2291,00	-2603,00	-2337,00	-2339,00	**-2426,33**
**Mean**	-791,72	-1012,07	-1098,75	-1007,39	**-1039,40**	-1164,79	-1102,97	-1341,25	-1371,50	**-1271,91**	-1921,50	-2270,25	-2284,00	-2333,25	**-2295,83**
**SD**	347,84	204,37	42,96	72,41	**83,41**	375,00	187,47	62,19	109,95	**79,04**	267,76	229,19	76,42	142,99	**107,80**
**SEM**	173,92	102,19	21,48	36,20	41,70	187,50	93,73	31,09	54,98	39,52	133,88	114,60	38,21	71,49	53,90
**P<**	n.s.	n.s.	n.s.	n.s.	n.s.	n.s.	n.s.	n.s.	n.s.	n.s.	n.s.	n.s.	n.s.	n.s.	n.s.

Tab. 23 Effects of RR-Alt-X 5.00 mg/l on pyramidal cell activity in terms of changes of population spike amplitudes. Results from single slices as obtained after single stimuli (SS) or after burst stimuli (TBS). Overview on final results after averaging 4 slices. SD=standard deviation; SEM=standard error of mean. P<=Wilcoxon Mann Whitny U-Test. ACSF=artificial cerebro-spinal fluid.

Report NCAG 15/17 H – Hippocampus slice preparation

NCAG 1517															
slice	ACSF +SS					RR-Alt-X 10.0 mg/l + SS					RR-Alt-X 10.0 mg/l + TBS				
time [min]	10	20	30	40	Mean 20-40	50	60	70	80	Mean 60-80	90	100	110	120	Mean 100-120
1	-730,29	-913,32	-888,69	-1188,00	* **-996,67**	-2634,00	-2791,00	-2799,00	-2561,00	* **-2717,00**	-3437,00	-3648,00	-3678,00	-3475,00	* **-3600,33**
2	-1024,00	-1167,00	-1080,00	-875,35	**-1040,78**	-1442,00	-1448,00	-1600,00	-1605,00	**-1551,00**	-2745,00	-2785,00	-2810,00	-2573,00	**-2722,67**
3	-464,16	-925,52	-921,26	-1024,00	**-956,93**	-1499,00	-1444,00	-1423,00	-1741,00	**-1536,00**	-2819,00	-2962,00	-3437,00	-3130,00	**-3176,33**
4	-866,81	-1271,00	-813,98	-1175,00	**-1086,66**	-1719,00	-1553,00	-1734,00	-1304,00	**-1530,33**	-2518,00	-3036,00	-2991,00	-3310,00	**-3112,33**
**Mean**	-784,99	-1121,17	-938,41	-1024,78	**-1028,12**	-1553,33	-1481,67	-1585,67	-1550,00	**-1539,11**	-2694,00	-2927,67	-3079,33	-3004,33	**-3003,78**
**SD**	288,75	177,24	133,84	149,83	**65,79**	146,27	61,81	155,99	223,63	**10,68**	156,85	128,97	322,70	384,23	**245,54**
**SEM**	144,37	88,62	66,92	74,91	32,89	73,14	30,90	78,00	111,82	5,34	78,42	64,49	161,35	192,12	122,77
**P<**	n.s.	n.s.	n.s.	n.s.	n.s.	0.01	0.01	0.01	0.01	0.01	0.01	0.01	0.01	0.01	0.01

Tab. 24 Effects of RR-Alt-X 10.0 mg/l on pyramidal cell activity in terms of changes of population spike amplitudes. Results from single slices as obtained after single stimuli (SS) or after burst stimuli (TBS). Overview on final results after averaging 4 slices. SD=standard deviation; SEM=standard error of mean. P<=Wilcoxon Mann Whitny U-Test. ACSF=artificial cerebro-spinal fluid. Outliers are marked by *.

NCAG 1517															
slice	ACSF +SS					RR-Alt-X 20.0 mg/l + SS					RR-Alt-X 20.0 mg/l + TBS				
time [min]	10	20	30	40	Mean 20-40	50	60	70	80	Mean 60-80	90	100	110	120	Mean 100-120
1	-1093,00	-1028,00	-1020,00	-888,21	**-978,74**	-1453,00	-2034,00	-1918,00	-1810,00	**-1920,67**	-3502,00	-3636,00	-3488,00	-3788,00	**-3637,33**
2	-1041,00	-954,76	-986,11	-965,70	**-968,86**	-2138,00	-2037,00	-2040,00	-1910,00	**-1995,67**	-3720,00	-3546,00	-3501,00	-3288,00	**-3445,00**
3	-1443,00	-933,23	-1127,00	-1114,00	**-1058,08**	-1704,00	-1930,00	-1768,00	-1995,00	**-1897,67**	-2960,00	-3425,00	-3244,00	-3282,00	**-3317,00**
4	-943,59	-947,15	-1037,00	-996,87	**-993,67**	-1814,00	-1811,00	-1793,00	-2063,00	**-1889,00**	-2785,00	-2953,00	-3696,00	-3145,00	**-3264,67**
**Mean**	-1130,15	-965,79	-1042,53	-991,20	**-999,84**	-1777,25	-1953,00	-1879,75	-1944,50	**-1925,75**	-3241,75	-3390,00	-3482,25	-3375,75	**-3416,00**
**SD**	217,57	42,42	60,16	93,75	**40,14**	284,02	106,94	125,38	109,35	**48,49**	441,38	303,89	185,15	282,66	**165,86**
**SEM**	108,78	21,21	30,08	46,88	20,07	142,01	53,47	62,69	54,68	24,24	220,69	151,94	92,57	141,33	82,93
**P<**	n.s.	n.s.	n.s.	n.s.	n.s.	0.01	0.01	0.01	0.01	0.01	0.01	0.01	0.01	0.01	0.01

Tab. 25 Effects of RR-Alt-X 20.0 mg/l on pyramidal cell activity in terms of changes of population spike amplitudes. Results from single slices as obtained after single stimuli (SS) or after burst stimuli (TBS). Overview on final results after averaging 4 slices. SD=standard deviation; SEM=standard error of mean. P<=Wilcoxon Mann Whitny U-Test. ACSF=artificial cerebro-spinal fluid.

Report NCAG 15/17 H – Hippocampus slice preparation

NCAG 1517															
**slice**	**ACSF +SS**					**RR-Alt-X 30.0 mg/l + SS**					**RR-Alt-X 30.0 mg/l + TBS**				
**time [min]**	**10**	**20**	**30**	**40**	**Mean 20-40**	**50**	**60**	**70**	**80**	**Mean 60-80**	**90**	**100**	**110**	**120**	**Mean 100-120**
**1**	-1030,00	-1136,00	-1049,00	-1057,00	**-1080,67**	-1341,00	-2091,00	-2400,00	-1787,00	**-2092,67**	-4200,00	-4726,00	-4496,00	-4404,00	**-4542,00**
**2**	-545,97	-931,08	-949,99	-1232,00	**-1037,69**	-2872,00	-1713,00	-2263,00	-2565,00	**-2180,33**	-4965,00	-4490,00	-4786,00	-4115,00	**-4463,67**
**3**	-1021,00	-1287,00	-1222,00	-1132,00	**-1213,67**	-2531,00	-2434,00	-2264,00	-2090,00	**-2262,67**	-3934,00	-3945,00	-4431,00	-3982,00	**-4119,33**
**4**	-886,78	-957,11	-1199,00	-1162,00	**-1106,04**	-1888,00	-1852,00	-2055,00	-2345,00	**-2084,00**	-3549,00	-4136,00	-4601,00	-4717,00	**-4484,67**
**Mean**	-870,94	-1077,80	-1105,00	-1145,75	**-1109,52**	-2158,00	-2022,50	-2245,50	-2196,75	**-2154,92**	-4162,00	-4324,25	-4578,50	-4304,50	**-4402,42**
**SD**	226,33	166,58	128,70	72,50	**74,95**	680,52	315,64	142,37	335,10	**83,98**	598,33	350,31	155,05	326,58	**191,60**
**SEM**	113,16	83,29	64,35	36,25	37,47	340,26	157,82	71,19	167,55	41,99	299,17	175,15	77,53	163,29	95,80
**P<**	n.s.	n.s.	n.s.	n.s.	n.s.	0.01	0.01	0.01	0.01	0.01	0.01	0.01	0.01	0.01	0.01

Tab. 26 Effects of RR-Alt-X 30.0 mg/l on pyramidal cell activity in terms of changes of population spike amplitudes. Results from single slices as obtained after single stimuli (SS) or after burst stimuli (TBS). Overview on final results after averaging 4 slices. SD=standard deviation; SEM=standard error of mean. P<=Wilcoxon Mann Whitny U-Test. ACSF=artificial cerebro-spinal fluid.

NCAG 1517															
**slice**	**ACSF +SS**					**RR-Alt-G 5.00 mg/l + SS**					**RR-Alt-G 5.00 mg/l + TBS**				
**time [min]**	**10**	**20**	**30**	**40**	**Mean 20-40**	**50**	**60**	**70**	**80**	**Mean 60-80**	**90**	**100**	**110**	**120**	**Mean 100-120**
**1**	-1015,00	-985,39	-995,36	-1030,00	**-1003,58**	-1102,00	-1126,00	-1098,00	-1103,00	**-1109,00**	-2592,00	-2490,00	-2394,00	-2074,00	**-2319,33**
**2**	-1086,00	-966,24	-1109,00	-1185,00	**-1086,75**	-1004,00	-1354,00	-1088,00	-1217,00	**-1219,67**	-1753,00	-2573,00	-2188,00	-2244,00	**-2335,00**
**3**	-1006,00	-914,13	-996,07	-1074,00	**-994,73**	-1220,00	-1317,00	-1173,00	-1233,00	**-1241,00**	-2627,00	-2616,00	-2668,00	-2799,00	**-2694,33**
**4**	-1170,00	-1090,00	-1041,00	-1352,00	**-1161,00**	-1653,00	-1286,00	-1253,00	-1208,00	**-1249,00**	-1293,00	-2302,00	-2566,00	-2628,00	**-2498,67**
**Mean**	-1069,25	-988,94	-1035,36	-1160,25	**-1061,52**	-1244,75	-1270,75	-1153,00	-1190,25	**-1204,67**	-2066,25	-2495,25	-2454,00	-2436,25	**-2461,83**
**SD**	76,10	73,80	53,54	143,51	**78,21**	286,13	100,42	76,70	59,08	**64,97**	654,95	139,04	210,31	334,93	**174,93**
**SEM**	38,05	36,90	26,77	71,75	39,10	143,07	50,21	38,35	29,54	32,48	327,48	69,52	105,16	167,47	87,47
**P<**	n.s.	n.s.	n.s.	n.s.	n.s.	n.s.	n.s.	n.s.	n.s.	n.s.	n.s.	n.s.	n.s.	n.s.	n.s.

Tab. 27 Effects of RR-Alt-G 5.00 mg/l on pyramidal cell activity in terms of changes of population spike amplitudes. Results from single slices as obtained after single stimuli (SS) or after burst stimuli (TBS). Overview on final results after averaging 4 slices. SD=standard deviation; SEM=standard error of mean. P<=Wilcoxon Mann Whitny U-Test. ACSF=artificial cerebro-spinal fluid.

Report NCAG 15/17 H – Hippocampus slice preparation

NCAG 1517															
**slice**	**ACSF +SS**					**RR-Alt-G 10.0 mg/l + SS**					**RR-Alt-G 10.0 mg/l + TBS**				
**time [min]**	**10**	**20**	**30**	**40**	**Mean 20-40**	**50**	**60**	**70**	**80**	**Mean 60-80**	**90**	**100**	**110**	**120**	**Mean 100-120**
**1**	-1143,00	-1105,00	-1094,00	-1142,00	**-1113,67**	-1195,00	-1914,00	-1190,00	-1276,00	**-1460,00**	-2171,00	-2566,00	-2968,00	-3104,00	**-2879,33**
**2**	-1086,00	-973,95	-1182,00	-1042,00	**-1065,98**	-1594,00	-1600,00	-1475,00	-1610,00	**-1561,67**	-2029,00	-2655,00	-2547,00	-2426,00	**-2542,67**
**3**	-875,84	-804,84	-878,66	-992,39	**-891,96**	-750,14	-1477,00	-1483,00	-1573,00	**-1511,00**	-2476,00	-2628,00	-2555,00	-2713,00	**-2632,00**
**4**	-1053,00	-1053,00	-901,10	-998,93	**-984,34**	-1758,00	-2015,00	-1739,00	-1449,00	**-1734,33**	-2576,00	-2777,00	-2719,00	-2680,00	**-2725,33**
**Mean**	-1039,46	-984,20	-1013,94	-1043,83	**-1013,99**	-1324,29	-1751,50	-1471,75	-1477,00	**-1566,75**	-2313,00	-2656,50	-2697,25	-2730,75	**-2694,83**
**SD**	115,24	131,15	147,97	69,05	**97,31**	449,89	254,39	224,31	150,65	**119,18**	255,97	88,55	197,14	279,93	**143,84**
**SEM**	57,62	65,58	73,99	34,52	48,66	224,95	127,19	112,15	75,33	59,59	127,98	44,28	98,57	139,96	71,92
**P<**	n.s.	n.s.	n.s.	n.s.	n.s.	n.s.	0.01	0.01	0.01	0.01	n.s.	0.01	0.01	0.02	0.01

Tab. 28 Effects of RR-Alt-G 10.0 mg/l on pyramidal cell activity in terms of changes of population spike amplitudes. Results from single slices as obtained after single stimuli (SS) or after burst stimuli (TBS). Overview on final results after averaging 4 slices. SD=standard deviation; SEM=standard error of mean. P<=Wilcoxon Mann Whitny U-Test. ACSF=artificial cerebro-spinal fluid.

NCAG 1517															
**slice**	**ACSF +SS**					**RR-Alt-G 20.0 mg/l + SS**					**RR-Alt-G 20.0 mg/l + TBS**				
**time [min]**	**10**	**20**	**30**	**40**	**Mean 20-40**	**50**	**60**	**70**	**80**	**Mean 60-80**	**90**	**100**	**110**	**120**	**Mean 100-120**
**1**	-1162,00	-860,33	-803,67	-867,05	**-843,68**	-1481,00	-1919,00	-1682,00	-2191,00	**-1930,67**	-3853,00	-3085,00	-3995,00	-3692,00	**-3590,67**
**2**	-779,77	-960,52	-1131,00	-929,37	**-1006,96**	-1450,00	-1722,00	-1751,00	-1764,00	**-1745,67**	-3640,00	-3552,00	-3655,00	-3709,00	**-3638,67**
**3**	-971,95	-1187,00	-1072,00	-950,08	**-1069,69**	-2149,00	-2302,00	-2200,00	-2170,00	**-2224,00**	-2633,00	-3464,00	-3696,00	-2933,00	**-3364,33**
**4**	-856,88	-944,74	-1151,00	-1285,00	**-1126,91**	-2058,00	-2229,00	-1863,00	-2424,00	**-2172,00**	-3444,00	-3481,00	-3679,00	-3492,00	**-3550,67**
**Mean**	-942,65	-988,15	-1039,42	-1007,88	**-1011,81**	-1784,50	-2043,00	-1874,00	-2137,25	**-2018,08**	-3392,50	-3395,50	-3756,25	-3456,50	**-3536,08**
**SD**	166,19	139,67	160,70	188,09	**122,32**	370,43	270,86	229,78	274,17	**222,07**	533,17	210,48	160,05	362,64	**120,02**
**SEM**	83,10	69,84	80,35	94,04	61,16	185,22	135,43	114,89	137,08	111,03	266,58	105,24	80,03	181,32	60,01
**P<**	n.s.	n.s.	n.s.	n.s.	n.s.	0.01	0.01	0.01	0.01	0.01	0.01	0.01	0.01	0.01	0.01

Tab. 29 Effects of RR-Alt-G 20.0 mg/l on pyramidal cell activity in terms of changes of population spike amplitudes. Results from single slices as obtained after single stimuli (SS) or after burst stimuli (TBS). Overview on final results after averaging 4 slices. SD=standard deviation; SEM=standard error of mean. P<=Wilcoxon Mann Whitny U-Test. ACSF=artificial cerebro-spinal fluid.

Report NCAG 15/17 H – Hippocampus slice preparation

NCAG 1517															
**slice**	**ACSF +SS**					**RR-Alt-G 30.0 mg/l + SS**					**RR-Alt-G 30.0 mg/l + TBS**				
**time [min]**	**10**	**20**	**30**	**40**	**Mean 20-40**	**50**	**60**	**70**	**80**	**Mean 60-80**	**90**	**100**	**110**	**120**	**Mean 100-120**
**1**	-742,19	-989,34	-968,15	-845,76	**-934,42**	-1799,00	-2469,00	-2553,00	-2478,00	**-2500,00**	-2954,00	-4942,00	-4913,00	-3398,00	**-4417,67**
**2**	-1088,00	-1230,00	-1010,00	-725,78	**-988,59**	-2007,00	-1876,00	-1915,00	-2049,00	**-1946,67**	-4977,00	-4881,00	-3872,00	-4385,00	**-4379,33**
**3**	-678,44	-1385,00	-1035,00	-902,39	**-1107,46**	-2022,00	-2482,00	-2557,00	-2357,00	**-2465,33**	-3953,00	-3802,00	-4486,00	-4152,00	**-4146,67**
**4**	-1193,00	-1169,00	-1084,00	-1073,00	**-1108,67**	-2525,00	-2237,00	-2472,00	-2220,00	**-2309,67**	-3633,00	-3732,00	-3947,00	-3964,00	**-3881,00**
**Mean**	-925,41	-1193,34	-1024,29	-886,73	**-1034,79**	-2088,25	-2266,00	-2374,25	-2276,00	**-2305,42**	-3879,25	-4339,25	-4304,50	-3974,75	**-4206,17**
**SD**	253,38	163,59	48,43	144,37	**87,46**	308,44	283,32	308,66	184,42	**253,08**	842,07	661,86	489,24	421,30	**247,65**
**SEM**	126,69	81,80	24,21	72,18	43,73	154,22	141,66	154,33	92,21	126,54	421,03	330,93	244,62	210,65	123,83
**P<**	n.s.	n.s.	n.s.	n.s.	n.s.	0.01	0.01	0.01	0.01	0.01	0.01	0.01	0.01	0.01	0.01

Tab. 30 Effects of RR-Alt-G 30.0 mg/l on pyramidal cell activity in terms of changes of population spike amplitudes. Results from single slices as obtained after single stimuli (SS) or after burst stimuli (TBS). Overview on final results after averaging 4 slices. SD=standard deviation; SEM=standard error of mean. P<=Wilcoxon Mann Whitny U-Test. ACSF=artificial cerebro-spinal fluid.

NCAG 1517															
**slice**	**ACSF +SS**					**Rosavin 0.25 mg/l + SS**					**Rosavin 0.25 mg/l + TBS**				
**time [min]**	**10**	**20**	**30**	**40**	**Mean 20-40**	**50**	**60**	**70**	**80**	**Mean 60-80**	**90**	**100**	**110**	**120**	**Mean 100-120**
**1**	-795,57	-1285,00	-1062,00	-1169,00	**-1172,00**	-1205,00	-1153,00	-1505,00	-1690,00	**-1449,33**	-2868,00	-2811,00	-3048,00	-2709,00	**-2856,00**
**2**	-734,49	-983,34	-965,10	-1072,00	**-1006,81**	-1250,00	-1262,00	-1260,00	-1235,00	**-1252,33**	-2084,00	-2017,00	-2264,00	-2218,00	**-2166,33**
**3**	-728,04	-973,01	-944,83	-1076,00	**-997,95**	-1105,00	-1266,00	-1247,00	-1270,00	**-1261,00**	-2328,00	-2649,00	-2578,00	-2396,00	**-2541,00**
**4**	-832,76	-933,13	-1140,00	-885,06	**-986,06**	-1011,00	-1360,00	-1267,00	-1208,00	**-1278,33**	-2238,00	-2401,00	-2438,00	-2810,00	**-2549,67**
**Mean**	-772,72	-1043,62	-1027,98	-1050,52	**-1040,71**	-1142,75	-1260,25	-1319,75	-1350,75	**-1310,25**	-2379,50	-2469,50	-2582,00	-2533,25	**-2528,25**
**SD**	50,28	162,37	90,51	119,06	**87,94**	106,71	84,63	123,78	227,59	**93,35**	340,89	345,59	336,17	274,29	**282,27**
**SEM**	25,14	81,18	45,25	59,53	43,97	53,35	42,32	61,89	113,79	46,68	170,45	172,79	168,09	137,14	141,13
**P<**	n.s.	n.s.	n.s.	n.s.	n.s.	n.s.	0.10	0.05	0.05	0.05	n.s.	n.s.	n.s.	n.s.	n.s.

Tab. 31 Effects of Rosavin 0.25 mg/l on pyramidal cell activity in terms of changes of population spike amplitudes. Results from single slices as obtained after single stimuli (SS) or after burst stimuli (TBS). Overview on final results after averaging 4 slices. SD=standard deviation; SEM=standard error of mean. P<=Wilcoxon Mann Whitny U-Test. ACSF=artificial cerebro-spinal fluid.

NeuroCode AG
Sportparkstr. 9
D-35578 Wetzlar / Germany

EuroPharm, Inc
955 Challenger Drive, Green Bay
Wisconsin 54311, USA

Report NCAG 15/17 H – Hippocampus slice preparation

NCAG 1517															
**slice**	**ACSF +SS**					**Rosavin 0.50 mg/l + SS**					**Rosavin 0.50 mg/l + TBS**				
**time [min]**	**10**	**20**	**30**	**40**	**Mean 20-40**	**50**	**60**	**70**	**80**	**Mean 60-80**	**90**	**100**	**110**	**120**	**Mean 100-120**
**1**	-803,97	-1024,00	-1168,00	-1148,00	**-1113,33**	-1301,00	-1568,00	-1678,00	-1667,00	**-1637,67**	-2911,00	-2927,00	-2707,00	-2847,00	**-2827,00**
**2**	-1128,00	-990,53	-896,68	-912,55	**-933,25**	-1391,00	-1821,00	-1820,00	-1693,00	**-1778,00**	-2953,00	-3019,00	-2662,00	-2825,00	**-2835,33**
**3**	-994,49	-1224,00	-975,59	-1197,00	**-1132,20**	-1513,00	-1718,00	-1767,00	-1756,00	**-1747,00**	-2746,00	-2506,00	-2877,00	-2949,00	**-2777,33**
**4**	-994,36	-984,69	-1074,00	-921,45	**-993,38**	-1859,00	-1858,00	-1896,00	-1921,00	**-1891,67**	-2691,00	-3113,00	-2967,00	-2937,00	**-3005,67**
**Mean**	-980,21	-1055,81	-1028,57	-1044,75	**-1043,04**	-1516,00	-1741,25	-1790,25	-1759,25	**-1763,58**	-2825,25	-2891,25	-2803,25	-2889,50	**-2861,33**
**SD**	133,30	113,46	117,91	148,91	**95,58**	244,61	129,80	91,67	114,12	**104,47**	126,46	267,82	143,14	62,62	**99,57**
**SEM**	66,65	56,73	58,95	74,45	47,79	122,31	64,90	45,83	57,06	52,24	63,23	133,91	71,57	31,31	49,79
**P<**	n.s.	n.s.	n.s.	n.s.	n.s.	0.01	0.01	0.01	0.01	0.01	0.01	0.01	0.01	0.01	0.01

Tab. 32 Effects of Rosavin 0.50 mg/l on pyramidal cell activity in terms of changes of population spike amplitudes. Results from single slices as obtained after single stimuli (SS) or after burst stimuli (TBS). Overview on final results after averaging 4 slices. SD=standard deviation; SEM=standard error of mean. P<=Wilcoxon Mann Whitny U-Test. ACSF=artificial cerebro-spinal fluid.

NCAG 1517															
**slice**	**ACSF +SS**					**Rosavin 0.75 mg/l + SS**					**Rosavin 0.75 mg/l + TBS**				
**time [min]**	**10**	**20**	**30**	**40**	**Mean 20-40**	**50**	**60**	**70**	**80**	**Mean 60-80**	**90**	**100**	**110**	**120**	**Mean 100-120**
**1**	-697,46	-1069,00	-945,09	-1047,00	**-1020,36**	-1643,00	-2669,00	-2349,00	-2149,00	**-2389,00**	-4229,00	-4334,00	-4114,00	-3924,00	**-4124,00**
**2**	-714,73	-1020,00	-984,82	-980,23	**-995,02**	-1410,00	-1824,00	-1957,00	-2047,00	**-1942,67**	-3396,00	-4153,00	-4330,00	-4339,00	**-4274,00**
**3**	-638,11	-1029,00	-983,84	-1307,00	**-1106,61**	-1900,00	-1860,00	-1875,00	-1978,00	**-1904,33**	-4405,00	-4975,00	-4650,00	-3708,00	**-4444,33**
**4**	-991,26	-1105,00	-976,94	-1203,00	**-1094,98**	-1863,00	-2190,00	-2207,00	-2087,00	**-2161,33**	-2810,00	-3809,00	-4116,00	-4079,00	**-4001,33**
**Mean**	-760,39	-1055,75	-972,67	-1134,31	**-1054,24**	-1704,00	-2135,75	-2097,00	-2065,25	**-2099,33**	-3710,00	-4317,75	-4302,50	-4012,50	**-4210,92**
**SD**	157,37	39,14	18,72	148,22	**54,95**	226,46	391,80	219,46	71,72	**223,85**	744,08	489,29	252,87	265,57	**191,44**
**SEM**	78,69	19,57	9,36	74,11	27,47	113,23	195,90	109,73	35,86	111,92	372,04	244,64	126,43	132,78	95,72
**P<**	n.s.	n.s.	n.s.	n.s.	n.s.	0.01	0.01	0.01	0.01	0.01	0.01	0.01	0.01	0.01	0.01

Tab. 33 Effects of Rosavin 0.75 mg/l on pyramidal cell activity in terms of changes of population spike amplitudes. Results from single slices as obtained after single stimuli (SS) or after burst stimuli (TBS). Overview on final results after averaging 4 slices. SD=standard deviation; SEM=standard error of mean. P<=Wilcoxon Mann Whitny U-Test. ACSF=artificial cerebro-spinal fluid.

Report NCAG 15/17 H – Hippocampus slice preparation

NCAG 1517															
**slice**	**ACSF +SS**					**Rosavin 1.50 mg/l + SS**					**Rosavin 1.50 mg/l + TBS**				
**time [min]**	**10**	**20**	**30**	**40**	**Mean 20-40**	**50**	**60**	**70**	**80**	**Mean 60-80**	**90**	**100**	**110**	**120**	**Mean 100-120**
**1**	-1142,00	-1004,00	-1121,00	-1206,00	**-1110,33**	-1961,00	-1815,00	-2186,00	-2390,00	**-2130,33**	-3992,00	-4236,00	-4047,00	-4349,00	**-4210,67**
**2**	-966,39	-1201,00	-1054,00	-1130,00	**-1128,33**	-2026,00	-2226,00	-2272,00	-2389,00	**-2295,67**	-3988,00	-4054,00	-4171,00	-4051,00	**-4092,00**
**3**	-1208,00	-1113,00	-1104,00	-1305,00	**-1174,00**	-1721,00	-1852,00	-2183,00	-2448,00	**-2161,00**	-2938,00	-3837,00	-4322,00	-4314,00	**-4157,67**
**4**	-963,05	-1080,00	-929,53	-1018,00	**-1009,18**	-2455,00	-2149,00	-1978,00	-2277,00	**-2134,67**	-3582,00	-4187,00	-4947,00	-4582,00	**-4572,00**
**Mean**	-1069,86	-1099,50	-1052,13	-1164,75	**-1105,46**	-2040,75	-2010,50	-2154,75	-2376,00	**-2180,42**	-3625,00	-4078,50	-4371,75	-4324,00	**-4258,08**
**SD**	124,37	81,62	86,54	121,26	**69,56**	305,73	207,34	124,85	71,53	**78,02**	496,75	178,42	399,65	217,42	**214,83**
**SEM**	62,18	40,81	43,27	60,63	34,78	152,87	103,67	62,43	35,77	39,01	248,37	89,21	199,82	108,71	107,42
**P<**	n.s.	n.s.	n.s.	n.s.	n.s.	0.01	0.01	0.01	0.01	0.01	0.01	0.01	0.01	0.01	0.01

Tab. 34 Effects of Rosavin 1.50 mg/l on pyramidal cell activity in terms of changes of population spike amplitudes. Results from single slices as obtained after single stimuli (SS) or after burst stimuli (TBS). Overview on final results after averaging 4 slices. SD=standard deviation; SEM=standard error of mean. P<=Wilcoxon Mann Whitny U-Test. ACSF=artificial cerebro-spinal fluid.

NCAG 1517															
**slice**	**ACSF +SS**					**Salidroside 0.25 mg/l + SS**					**Salidroside 0.25 mg/l + TBS**				
**time [min]**	**10**	**20**	**30**	**40**	**Mean 20-40**	**50**	**60**	**70**	**80**	**Mean 60-80**	**90**	**100**	**110**	**120**	**Mean 100-120**
**1**	-910,75	-975,53	-1127,00	-863,22	**-988,58**	-899,98	-1168,00	-1265,00	-1158,00	**-1197,00**	-2223,00	-2285,00	-2194,00	-2215,00	**-2231,33**
**2**	-895,50	-956,09	-1058,00	-1170,00	**-1061,36**	-1594,00	-1306,00	-1331,00	-1229,00	**-1288,67**	-2109,00	-2062,00	-2051,00	-2163,00	**-2092,00**
**3**	-984,24	-1045,00	-1013,00	-1111,00	**-1056,33**	-1281,00	-1349,00	-1195,00	-1305,00	**-1283,00**	-2851,00	-2137,00	-2257,00	-2597,00	**-2330,33**
**4**	-916,88	-1162,00	-1206,00	-998,13	**-1122,04**	-1091,00	-1334,00	-1204,00	-1235,00	**-1257,67**	-1975,00	-2661,00	-2415,00	-2218,00	**-2431,33**
**Mean**	-926,84	-1034,66	-1101,00	-1035,59	**-1057,08**	-1216,50	-1289,25	-1248,75	-1231,75	**-1256,58**	-2289,50	-2286,25	-2229,25	-2298,25	**-2271,25**
**SD**	39,31	93,08	84,25	135,24	**54,56**	295,86	82,77	63,04	60,06	**41,95**	387,81	266,46	150,87	200,76	**144,73**
**SEM**	19,65	46,54	42,12	67,62	27,28	147,93	41,39	31,52	30,03	20,97	193,91	133,23	75,44	100,38	72,37
**P<**	n.s.	n.s.	n.s.	n.s.	n.s.	n.s.	n.s.	n.s.	n.s.	n.s.	n.s.	n.s.	n.s.	n.s.	n.s.

Tab. 35 Effects of Salidroside 0.25 mg/l on pyramidal cell activity in terms of changes of population spike amplitudes. Results from single slices as obtained after single stimuli (SS) or after burst stimuli (TBS). Overview on final results after averaging 4 slices. SD=standard deviation; SEM=standard error of mean. P<=Wilcoxon Mann Whitny U-Test. ACSF=artificial cerebro-spinal fluid.

Report NCAG 15/17 H – Hippocampus slice preparation

NCAG 1517															
**slice**	**ACSF +SS**					**Salidroside 0.50 mg/l + SS**					**Salidroside 0.50 mg/l + TBS**				
**time [min]**	**10**	**20**	**30**	**40**	**Mean 20-40**	**50**	**60**	**70**	**80**	**Mean 60-80**	**90**	**100**	**110**	**120**	**Mean 100-120**
**1**	-874,30	-1031,00	-974,38	-1055,00	**-1020,13**	-1278,00	-1539,00	-1710,00	-1612,00	**-1620,33**	-2826,00	-3273,00	-3245,00	-3270,00	**-3262,67**
**2**	-955,90	-1137,00	-954,00	-1085,00	**-1058,67**	-1590,00	-1690,00	-1465,00	-1532,00	**-1562,33**	-2564,00	-3231,00	-3155,00	-3186,00	**-3190,67**
**3**	-769,15	-929,22	-922,32	-928,81	**-926,78**	-1511,00	-1595,00	-1671,00	-1634,00	**-1633,33**	-2344,00	-3430,00	-3403,00	-3350,00	**-3394,33**
**4**	-667,61	-988,58	-984,84	-969,92	**-981,11**	-1104,00	-1560,00	-1827,00	-1710,00	**-1699,00**	-2860,00	-3187,00	-3183,00	-2705,00	**-3025,00**
**Mean**	-816,74	-1021,45	-958,89	-1009,68	**-996,67**	-1370,75	-1596,00	-1668,25	-1622,00	**-1628,75**	-2648,50	-3280,25	-3246,50	-3127,75	**-3218,17**
**SD**	125,41	87,62	27,54	72,68	**56,33**	221,73	66,79	150,85	73,23	**56,09**	242,28	105,83	110,90	289,68	**153,93**
**SEM**	62,71	43,81	13,77	36,34	28,17	110,87	33,39	75,42	36,62	28,04	121,14	52,91	55,45	144,84	76,97
**P<**	n.s.	n.s.	n.s.	n.s.	n.s.	0.10	0.01	0.01	0.01	0.01	0.10	0.01	0.01	0.01	0.01

Tab. 36 Effects of Salidroside 0.50 mg/l on pyramidal cell activity in terms of changes of population spike amplitudes. Results from single slices as obtained after single stimuli (SS) or after burst stimuli (TBS). Overview on final results after averaging 4 slices. SD=standard deviation; SEM=standard error of mean. P<=Wilcoxon Mann Whitny U-Test. ACSF=artificial cerebro-spinal fluid.

NCAG 1517															
**slice**	**ACSF +SS**					**Salidroside 0.75 mg/l + SS**					**Salidroside 0.75 mg/l + TBS**				
**time [min]**	**10**	**20**	**30**	**40**	**Mean 20-40**	**50**	**60**	**70**	**80**	**Mean 60-80**	**90**	**100**	**110**	**120**	**Mean 100-120**
**1**	-514,15	-1147,00	-1274,00	-1027,00	**-1149,33**	-1805,00	-1726,00	-1758,00	-1923,00	**-1802,33**	-3313,00	-4028,00	-4282,00	-4781,00	**-4363,67**
**2**	-775,64	-1319,00	-1166,00	-942,65	**-1142,55**	-1636,00	-1911,00	-2128,00	-2056,00	**-2031,67**	-3356,00	-3422,00	-4195,00	-3783,00	**-3800,00**
**3**	-705,12	-830,69	-884,11	-1009,00	**-907,93**	-1729,00	-2187,00	-2118,00	-2021,00	**-2108,67**	-3755,00	-3782,00	-3714,00	-3815,00	**-3770,33**
**4**	-784,14	-1087,00	-1228,00	-1105,00	**-1140,00**	-2732,00	-2724,00	-2133,00	-2227,00	**-2361,33**	-3657,00	-3806,00	-4211,00	-4228,00	**-4081,67**
**Mean**	-694,76	-1095,92	-1138,03	-1020,91	**-1084,95**	-1975,50	-2137,00	-2034,25	-2056,75	**-2076,00**	-3520,25	-3759,50	-4100,50	-4151,75	**-4003,92**
**SD**	125,51	202,32	174,97	66,77	**118,08**	509,05	434,77	184,27	126,69	**230,46**	218,89	250,78	260,43	465,89	**277,85**
**SEM**	62,75	101,16	87,48	33,38	59,04	254,52	217,38	92,14	63,35	115,23	109,45	125,39	130,21	232,94	138,93
**P<**	0.10	n.s.	n.s.	n.s.	n.s.	0.01	0.01	0.01	0.01	0.01	0.01	0.01	0.01	0.01	0.01

Tab. 37 Effects of Salidroside 0.75 mg/l on pyramidal cell activity in terms of changes of population spike amplitudes. Results from single slices as obtained after single stimuli (SS) or after burst stimuli (TBS). Overview on final results after averaging 4 slices. SD=standard deviation; SEM=standard error of mean. P<=Wilcoxon Mann Whitny U-Test. ACSF=artificial cerebro-spinal fluid.

NeuroCode AG
Sportparkstr. 9
D-35578 Wetzlar / Germany

EuroPharm, Inc
955 Challenger Drive, Green Bay
Wisconsin 54311, USA

Report NCAG 15/17 H – Hippocampus slice preparation

NCAG 1517															
**slice**	**ACSF +SS**					**Salidroside 1.50 mg/l + SS**					**Salidroside 1.50 mg/l + TBS**				
**time [min]**	**10**	**20**	**30**	**40**	**Mean 20-40**	**50**	**60**	**70**	**80**	**Mean 60-80**	**90**	**100**	**110**	**120**	**Mean 100-120**
**1**	-726,50	-931,86	-932,73	-857,10	**-907,23**	-1295,00	-1515,00	-1760,00	-1697,00	**-1657,33**	-4099,00	-4368,00	-4292,00	-3995,00	**-4218,33**
**2**	-776,46	-1129,00	-1184,00	-1237,00	**-1183,33**	-2267,00	-1885,00	-1814,00	-1731,00	**-1810,00**	-3560,00	-3968,00	-4068,00	-3889,00	**-3975,00**
**3**	-952,79	-992,72	-967,31	-909,22	**-956,42**	-1944,00	-2297,00	-2131,00	-2269,00	**-2232,33**	-3986,00	-4502,00	-4140,00	-3962,00	**-4201,33**
**4**	-1052,00	-887,43	-1222,00	-1078,00	**-1062,48**	-1785,00	-2256,00	-1920,00	-2160,00	**-2112,00**	-3575,00	-3952,00	-4121,00	-3861,00	**-3978,00**
**Mean**	-876,94	-985,25	-1076,51	-1020,33	**-1027,36**	-1822,75	-1988,25	-1906,25	-1964,25	**-1952,92**	-3805,00	-4197,50	-4155,25	-3926,75	**-4093,17**
**SD**	151,80	105,10	147,56	172,49	**122,51**	404,97	365,90	163,91	292,70	**265,31**	278,16	279,72	96,12	62,31	**134,90**
**SEM**	75,90	52,55	73,78	86,25	61,25	202,49	182,95	81,96	146,35	132,66	139,08	139,86	48,06	31,16	67,45
**P<**	n.s.	n.s.	n.s.	n.s.	n.s.	0.01	0.01	0.01	0.01	0.01	0.01	0.01	0.01	0.01	0.01

Tab. 38 Effects of Salidroside 1.50 mg/l on pyramidal cell activity in terms of changes of population spike amplitudes. Results from single slices as obtained after single stimuli (SS) or after burst stimuli (TBS). Overview on final results after averaging 4 slices. SD=standard deviation; SEM=standard error of mean. P<=Wilcoxon Mann Whitny U-Test. ACSF=artificial cerebro-spinal fluid.

NCAG 1517															
**slice**	**ACSF +SS**					**WS-KSM-66 0.25 mg/l + SS**					**WS-KSM-66 0.25 mg/l + TBS**				
**time [min]**	**10**	**20**	**30**	**40**	**Mean 20-40**	**50**	**60**	**70**	**80**	**Mean 60-80**	**90**	**100**	**110**	**120**	**Mean 100-120**
**1**	-708,14	-953,69	-910,18	-896,30	**-920,06**	-992,45	-1232,00	-1289,00	-944,40	**-1155,13**	-1387,00	-2231,00	-1832,00	-2064,00	**-2042,33**
**2**	-849,73	-1088,00	-1129,00	-996,14	**-1071,05**	-1059,00	-1097,00	-1116,00	-1046,00	**-1086,33**	-1966,00	-2030,00	-2202,00	-2315,00	**-2182,33**
**3**	-806,49	-1153,00	-1011,00	-947,93	**-1037,31**	-1045,00	-1021,00	-1170,00	-1148,00	**-1113,00**	-1796,00	-2037,00	-2065,00	-2098,00	**-2066,67**
**4**	-1225,00	-935,58	-1213,00	-1201,00	**-1116,53**	-1135,00	-1222,00	-1257,00	-1497,00	**-1325,33**	-1755,00	-1999,00	-2075,00	-1998,00	**-2024,00**
**Mean**	-897,34	-1032,57	-1065,80	-1010,34	**-1036,24**	-1057,86	-1143,00	-1208,00	-1158,85	**-1169,95**	-1726,00	-2074,25	-2043,50	-2118,75	**-2078,83**
**SD**	226,33	105,21	132,77	133,48	**83,98**	58,87	101,92	79,31	240,27	**107,39**	243,76	105,80	154,17	137,26	**71,18**
**SEM**	113,17	52,60	66,38	66,74	41,99	29,43	50,96	39,65	120,13	53,70	121,88	52,90	77,09	68,63	35,59
**P<**	n.s.	n.s.	n.s.	n.s.	n.s.	n.s.	n.s.	n.s.	n.s.	n.s.	n.s.	n.s.	n.s.	n.s.	n.s.

Tab. 39 Effects of WS KSM66 0.25 mg/l on pyramidal cell activity in terms of changes of population spike amplitudes. Results from single slices as obtained after single stimuli (SS) or after burst stimuli (TBS). Overview on final results after averaging 4 slices. SD=standard deviation; SEM=standard error of mean. P<=Wilcoxon Mann Whitny U-Test. ACSF=artificial cerebro-spinal fluid.

Report NCAG 15/17 H – Hippocampus slice preparation

NCAG 1517															
**slice**	**ACSF +SS**					**WS-KSM-66 0.50 mg/l + SS**					**WS-KSM-66 0.50 mg/l + TBS**				
**time [min]**	**10**	**20**	**30**	**40**	**Mean 20-40**	**50**	**60**	**70**	**80**	**Mean 60-80**	**90**	**100**	**110**	**120**	**Mean 100-120**
**1**	-917,78	-1075,00	-1193,00	-1122,00	**-1130,00**	-1519,00	-1804,00	-1856,00	-1764,00	**-1808,00**	-2791,00	-3139,00	-3242,00	-3153,00	**-3178,00**
**2**	-998,58	-1014,00	-1074,00	-902,24	**-996,75**	-1400,00	-1668,00	-1501,00	-1474,00	**-1547,67**	-3181,00	-3489,00	-3237,00	-3374,00	**-3366,67**
**3**	-1460,00	-1005,00	-1001,00	-1005,00	**-1003,67**	-1270,00	-1529,00	-1623,00	-1642,00	**-1598,00**	-2284,00	-2468,00	-2451,00	-2614,00	**-2511,00**
**4**	-1216,00	-889,94	-937,45	-919,92	**-915,77**	-1372,00	-1785,00	-1584,00	-1556,00	**-1641,67**	-2033,00	-2454,00	-2478,00	-2467,00	**-2466,33**
**Mean**	-1148,09	-995,99	-1051,36	-987,29	**-1011,55**	-1390,25	-1696,50	-1641,00	-1609,00	**-1648,83**	-2572,25	-2887,50	-2852,00	-2902,00	**-2880,50**
**SD**	243,10	77,23	109,68	100,39	**88,48**	102,41	126,83	152,09	124,03	**112,85**	513,91	512,82	447,59	431,26	**459,32**
**SEM**	121,55	38,62	54,84	50,19	44,24	51,21	63,41	76,05	62,01	56,42	256,95	256,41	223,79	215,63	229,66
**P<**	n.s.	n.s.	n.s.	n.s.	n.s.	0.01	0.01	0.01	0.01	0.01	n.s.	0.02	0.05	0.02	0.02

Tab. 40 Effects of WS KSM66 0.50 mg/l on pyramidal cell activity in terms of changes of population spike amplitudes. Results from single slices as obtained after single stimuli (SS) or after burst stimuli (TBS). Overview on final results after averaging 4 slices. SD=standard deviation; SEM=standard error of mean. P<=Wilcoxon Mann Whitny U-Test. ACSF=artificial cerebro-spinal fluid.

NCAG 1517															
**slice**	**ACSF +SS**					**WS-KSM-66 0.75 mg/l + SS**					**WS-KSM-66 0.75 mg/l + TBS**				
**time [min]**	**10**	**20**	**30**	**40**	**Mean 20-40**	**50**	**60**	**70**	**80**	**Mean 60-80**	**90**	**100**	**110**	**120**	**Mean 100-120**
**1**	-740,09	-1113,00	-924,76	-1127,00	**-1054,92**	-1296,00	-1713,00	-2097,00	-2236,00	**-2015,33**	-4004,00	-3266,00	-3023,00	-3546,00	**-3278,33**
**2**	-1362,00	-1091,00	-1084,00	-992,30	**-1055,77**	-1567,00	-1697,00	-1667,00	-2116,00	**-1826,67**	-2723,00	-2685,00	-3222,00	-2721,00	**-2876,00**
**3**	-1089,00	-1039,00	-1117,00	-1080,00	**-1078,67**	-1474,00	-1555,00	-2447,00	-1823,00	**-1941,67**	-2680,00	-2761,00	-3362,00	-3381,00	**-3168,00**
**4**	-964,57	-1095,00	-1162,00	-1170,00	**-1142,33**	-1739,00	-1800,00	-1630,00	-2202,00	**-1877,33**	-2944,00	-3441,00	-3528,00	-3355,00	**-3441,33**
**Mean**	-1038,92	-1084,50	-1071,94	-1092,33	**-1082,92**	-1519,00	-1691,25	-1960,25	-2094,25	**-1915,25**	-3087,75	-3038,25	-3283,75	-3250,75	**-3190,92**
**SD**	259,30	31,81	103,20	76,14	**41,11**	184,80	101,48	387,59	187,75	**81,65**	621,69	372,26	214,15	363,15	**238,08**
**SEM**	129,65	15,90	51,60	38,07	20,55	92,40	50,74	193,80	93,88	40,82	310,84	186,13	107,08	181,58	119,04
**P<**	n.s.	n.s.	n.s.	n.s.	n.s.	0.01	0.01	0.01	0.01	0.01	0.01	0.01	0.01	0.01	0.01

Tab. 41 Effects of WS KSM66 0.75 mg/l on pyramidal cell activity in terms of changes of population spike amplitudes. Results from single slices as obtained after single stimuli (SS) or after burst stimuli (TBS). Overview on final results after averaging 4 slices. SD=standard deviation; SEM=standard error of mean. P<=Wilcoxon Mann Whitny U-Test. ACSF=artificial cerebro-spinal fluid.

NeuroCode AG
Sportparkstr. 9
D-35578 Wetzlar / Germany

EuroPharm, Inc
955 Challenger Drive, Green Bay
Wisconsin 54311, USA

Report NCAG 15/17 H – Hippocampus slice preparation

NCAG 1517															
**slice**	**ACSF +SS**					**WS-KSM-66 1.00 mg/l + SS**					**WS-KSM-66 1.00 mg/l + TBS**				
**time [min]**	**10**	**20**	**30**	**40**	**Mean 20-40**	**50**	**60**	**70**	**80**	**Mean 60-80**	**90**	**100**	**110**	**120**	**Mean 100-120**
**1**	-1065,00	-1195,00	-1113,00	-1073,00	**-1127,00**	-1728,00	-2352,00	-2230,00	-1952,00	**-2178,00**	-2860,00	-3137,00	-4307,00	-3708,00	**-3717,33**
**2**	-1231,00	-1003,00	-1090,00	-1239,00	**-1110,67**	-1793,00	-2207,00	-2396,00	-1595,00	**-2066,00**	-2902,00	-2949,00	-3160,00	-3144,00	**-3084,33**
**3**	-866,82	-1066,00	-1054,00	-963,59	**-1027,86**	-1720,00	-1923,00	-2338,00	-2406,00	**-2222,33**	-4077,00	-4019,00	-4177,00	-3986,00	**-4060,67**
**4**	-792,53	-1101,00	-1251,00	-1226,00	**-1192,67**	-2148,00	-2030,00	-2187,00	-2270,00	**-2162,33**	-3166,00	-3613,00	-3740,00	-3751,00	**-3701,33**
**Mean**	-988,84	-1091,25	-1127,00	-1125,40	**-1114,55**	-1847,25	-2128,00	-2287,75	-2055,75	**-2157,17**	-3251,25	-3429,50	-3846,00	-3647,25	**-3640,92**
**SD**	198,22	80,18	86,16	131,60	**67,79**	203,15	189,78	96,14	361,31	**65,88**	566,92	482,22	517,66	357,06	**406,39**
**SEM**	99,11	40,09	43,08	65,80	33,90	101,57	94,89	48,07	180,65	32,94	283,46	241,11	258,83	178,53	203,20
**P<**	n.s.	n.s.	n.s.	n.s.	n.s.	0.01	0.01	0.01	0.01	0.01	0.01	0.01	0.01	0.01	0.01

Tab. 42 Effects of WS KSM66 1.00 mg/l on pyramidal cell activity in terms of changes of population spike amplitudes. Results from single slices as obtained after single stimuli (SS) or after burst stimuli (TBS). Overview on final results after averaging 4 slices. SD=standard deviation; SEM=standard error of mean. P<=Wilcoxon Mann Whitny U-Test. ACSF=artificial cerebro-spinal fluid.

NCAG 1517															
**slice**	**ACSF +SS**					**WS-KSM-66 1.50 mg/l + SS**					**WS-KSM-66 1.50 mg/l + TBS**				
**time [min]**	**10**	**20**	**30**	**40**	**Mean 20-40**	**50**	**60**	**70**	**80**	**Mean 60-80**	**90**	**100**	**110**	**120**	**Mean 100-120**
**1**	-858,51	-1073,00	-965,10	-1037,00	**-1025,03**	-2030,00	-1755,00	-2046,00	-2376,00	**-2059,00**	-2985,00	-3689,00	-3840,00	-4175,00	**-3901,33**
**2**	-907,99	-1147,00	-881,85	-923,82	**-984,22**	-1486,00	-2090,00	-1962,00	-2325,00	**-2125,67**	-3938,00	-4160,00	-3204,00	-3446,00	**-3603,33**
**3**	-1014,00	-1136,00	-936,94	-966,29	**-1013,08**	-2256,00	-2079,00	-2193,00	-2189,00	**-2153,67**	-2825,00	-3148,00	-4357,00	-3568,00	**-3691,00**
**4**	-898,32	-959,25	-954,03	-996,23	**-969,84**	-2134,00	-2100,00	-2224,00	-2043,00	**-2122,33**	-3294,00	-3555,00	-3493,00	-3635,00	**-3561,00**
**Mean**	-919,71	-1078,81	-934,48	-980,84	**-998,04**	-1976,50	-2006,00	-2106,25	-2233,25	**-2115,17**	-3260,50	-3638,00	-3723,50	-3706,00	**-3689,17**
**SD**	66,41	86,12	36,95	47,80	**25,44**	339,79	167,55	123,60	149,39	**39,99**	491,83	417,16	495,95	322,31	**151,45**
**SEM**	33,21	43,06	18,47	23,90	12,72	169,90	83,78	61,80	74,69	20,00	245,91	208,58	247,98	161,15	75,73
**P<**	n.s.	n.s.	n.s.	n.s.	n.s.	0.01	0.01	0.01	0.01	0.01	0.01	0.01	0.01	0.01	0.01

Tab. 43 Effects of WS KSM66 1.50 mg/l on pyramidal cell activity in terms of changes of population spike amplitudes. Results from single slices as obtained after single stimuli (SS) or after burst stimuli (TBS). Overview on final results after averaging 4 slices. SD=standard deviation; SEM=standard error of mean. P<=Wilcoxon Mann Whitny U-Test. ACSF=artificial cerebro-spinal fluid.

Report NCAG 15/17 H – Hippocampus slice preparation

NCAG 1517															
**slice**	**ACSF +SS**					**Bryonia alba 0.25 mg/l + SS**					**Bryonia alba 0.25 mg/l + TBS**				
**time [min]**	**10**	**20**	**30**	**40**	**Mean 20-40**	**50**	**60**	**70**	**80**	**Mean 60-80**	**90**	**100**	**110**	**120**	**Mean 100-120**
**1**	-679,16	-1070,00	-1041,00	-1153,00	**-1088,00**	-1264,00	-981,18	-1467,00	-1195,00	**-1214,39**	-2416,00	-2352,00	-2352,00	-2671,00	**-2458,33**
**2**	-947,76	-1092,00	-1158,00	-1240,00	**-1163,33**	-1390,00	-993,55	-1341,00	-1261,00	**-1198,52**	-2063,00	-2141,00	-2188,00	-1978,00	**-2102,33**
**3**	-1159,00	-1094,00	-1239,00	-1021,00	**-1118,00**	-919,07	-972,05	-919,62	-1167,00	**-1019,56**	-2346,00	-1953,00	-2117,00	-1959,00	**-2009,67**
**4**	-762,01	-887,70	-1328,00	-1049,00	**-1088,23**	-955,60	-1034,00	-1290,00	-1162,00	**-1162,00**	-1818,00	-2170,00	-2218,00	-2362,00	**-2250,00**
**Mean**	-886,98	-1035,93	-1191,50	-1115,75	**-1114,39**	-1132,17	-995,20	-1254,41	-1196,25	**-1148,62**	-2160,75	-2154,00	-2218,75	-2242,50	**-2205,08**
**SD**	213,30	99,41	122,01	100,43	**35,54**	231,26	27,33	235,26	45,54	**88,79**	274,77	163,33	98,41	340,70	**195,70**
**SEM**	106,65	49,71	61,01	50,22	17,77	115,63	13,66	117,63	22,77	44,40	137,39	81,66	49,21	170,35	97,85
**P<**	n.s.	n.s.	n.s.	n.s.	n.s.	n.s.	n.s.	n.s.	n.s.	n.s.	n.s.	n.s.	n.s.	n.s.	n.s.

Tab. 44 Effects of Bryonia alba 0.25 mg/l on pyramidal cell activity in terms of changes of population spike amplitudes. Results from single slices as obtained after single stimuli (SS) or after burst stimuli (TBS). Overview on final results after averaging 4 slices. SD=standard deviation; SEM=standard error of mean. P<=Wilcoxon Mann Whitny U-Test. ACSF=artificial cerebro-spinal fluid.

NCAG 1517															
**slice**	**ACSF +SS**					**Bryonia alba 0.50 mg/l + SS**					**Bryonia alba 0.50 mg/l + TBS**				
**time [min]**	**10**	**20**	**30**	**40**	**Mean 20-40**	**50**	**60**	**70**	**80**	**Mean 60-80**	**90**	**100**	**110**	**120**	**Mean 100-120**
**1**	-552,24	-942,99	-1064,00	-1068,00	**-1025,00**	-1228,00	-1290,00	-1445,00	-1814,00	**-1516,33**	-3243,00	-2632,00	-2321,00	-2107,00	**-2353,33**
**2**	-884,50	-1019,00	-845,45	-1222,00	**-1028,82**	-1106,00	-1671,00	-1761,00	-1716,00	**-1716,00**	-1824,00	-1733,00	-2629,00	-2613,00	**-2325,00**
**3**	-699,48	-858,70	-1075,00	-1007,00	**-980,23**	-610,72	-1917,00	-1621,00	-1494,00	**-1677,33**	-1935,00	-2288,00	-2418,00	-2146,00	**-2284,00**
**4**	-596,83	-1148,00	-1028,00	-1097,00	**-1091,00**	-1127,00	-1422,00	-1535,00	-1480,00	**-1479,00**	-2405,00	-2339,00	-2468,00	-3439,00	**-2748,67**
**Mean**	-683,26	-992,17	-1003,11	-1098,50	**-1031,26**	-1017,93	-1575,00	-1590,50	-1626,00	**-1597,17**	-2351,75	-2248,00	-2459,00	-2576,25	**-2427,75**
**SD**	147,64	122,79	107,01	90,47	**45,53**	276,65	277,38	134,48	165,51	**116,97**	645,33	375,31	128,72	619,41	**215,83**
**SEM**	73,82	61,40	53,50	45,24	22,76	138,32	138,69	67,24	82,76	58,48	322,67	187,65	64,36	309,70	107,91
**P<**	n.s.	n.s.	n.s.	n.s.	n.s.	n.s.	0.01	0.01	0.01	0.01	n.s.	n.s.	n.s.	n.s.	n.s.

Tab. 45 Effects of Bryonia alba 0.50 mg/l on pyramidal cell activity in terms of changes of population spike amplitudes. Results from single slices as obtained after single stimuli (SS) or after burst stimuli (TBS). Overview on final results after averaging 4 slices. SD=standard deviation; SEM=standard error of mean. P<=Wilcoxon Mann Whitny U-Test. ACSF=artificial cerebro-spinal fluid.

Report NCAG 15/17 H – Hippocampus slice preparation

NCAG 1517															
**slice**	**ACSF +SS**					**Bryonia alba 0.75 mg/l + SS**					**Bryonia alba 0.75 mg/l + TBS**				
**time [min]**	**10**	**20**	**30**	**40**	**Mean 20-40**	**50**	**60**	**70**	**80**	**Mean 60-80**	**90**	**100**	**110**	**120**	**Mean 100-120**
**1**	-1150,00	-1198,00	-1151,00	-1065,00	**-1138,00**	-1120,00	-1334,00	-1444,00	-1982,00	**-1586,67**	-2868,00	-3316,00	-3025,00	-2692,00	**-3011,00**
**2**	-772,34	-838,80	-1183,00	-1108,00	**-1043,27**	-1506,00	-1726,00	-1859,00	-1858,00	**-1814,33**	-2937,00	-3259,00	-3051,00	-3111,00	**-3140,33**
**3**	-558,63	-1197,00	-1134,00	-1281,00	**-1204,00**	-2414,00	-1612,00	-1941,00	-1939,00	**-1830,67**	-2296,00	-2535,00	-2772,00	-3185,00	**-2830,67**
**4**	-1040,00	-974,04	-920,76	-1210,00	**-1034,93**	-1453,00	-1799,00	-2124,00	-1864,00	**-1929,00**	-3174,00	-2812,00	-2722,00	-2758,00	**-2764,00**
**Mean**	-880,24	-1051,96	-1097,19	-1166,00	**-1105,05**	-1623,25	-1617,75	-1842,00	-1910,75	**-1790,17**	-2818,75	-2980,50	-2892,50	-2936,50	**-2936,50**
**SD**	266,69	176,89	119,36	97,85	**80,85**	554,16	204,22	287,53	60,12	**144,81**	372,33	372,82	169,58	247,55	**171,32**
**SEM**	133,34	88,45	59,68	48,93	40,43	277,08	102,11	143,76	30,06	72,41	186,16	186,41	84,79	123,78	85,66
**P<**	n.s.	n.s.	n.s.	n.s.	n.s.	0.05	0.01	0.01	0.01	0.01	0.02	0.01	0.01	0.01	0.01

Tab. 46 Effects of Bryonia alba 0.75 mg/l on pyramidal cell activity in terms of changes of population spike amplitudes. Results from single slices as obtained after single stimuli (SS) or after burst stimuli (TBS). Overview on final results after averaging 4 slices. SD=standard deviation; SEM=standard error of mean. P<=Wilcoxon Mann Whitny U-Test. ACSF=artificial cerebro-spinal fluid.

NCAG 1517															
**slice**	**ACSF +SS**					**Bryonia alba 1.00 mg/l + SS**					**Bryonia alba 1.00 mg/l + TBS**				
**time [min]**	**10**	**20**	**30**	**40**	**Mean 20-40**	**50**	**60**	**70**	**80**	**Mean 60-80**	**90**	**100**	**110**	**120**	**Mean 100-120**
**1**	-801,78	-1027,00	-1190,00	-1003,00	**-1073,33**	-1341,00	-1932,00	-1914,00	-2019,00	**-1955,00**	-3346,00	-3056,00	-3401,00	-3362,00	**-3273,00**
**2**	-1173,00	-1118,00	-1029,00	-968,85	**-1038,62**	-2618,00	-2199,00	-1988,00	-2238,00	**-2141,67**	-3373,00	-3632,00	-3236,00	-3113,00	**-3327,00**
**3**	-1088,00	-895,23	-945,73	-1065,00	**-968,65**	-1989,00	-1926,00	-1943,00	-1945,00	**-1938,00**	-2761,00	-3462,00	-3499,00	-3264,00	**-3408,33**
**4**	-910,32	-1058,00	-1030,00	-1021,00	**-1036,33**	-2402,00	-2494,00	-1879,00	-1974,00	**-2115,67**	-3128,00	-3647,00	-3383,00	-3002,00	**-3344,00**
**Mean**	-993,28	-1024,56	-1048,68	-1014,46	**-1029,23**	-2087,50	-2137,75	-1931,00	-2044,00	**-2037,58**	-3152,00	-3449,25	-3379,75	-3185,25	**-3338,08**
**SD**	168,15	94,13	102,15	40,04	**43,79**	561,93	269,47	46,14	132,87	**105,94**	282,80	275,26	108,55	159,42	**55,76**
**SEM**	84,08	47,07	51,08	20,02	21,90	280,97	134,73	23,07	66,43	52,97	141,40	137,63	54,27	79,71	27,88
**P<**	n.s.	n.s.	n.s.	n.s.	n.s.	n.s.	0.01	0.01	0.01	0.01	0.01	0.01	0.01	0.01	0.01

Tab. 47 Effects of Bryonia alba 1.00 mg/l on pyramidal cell activity in terms of changes of population spike amplitudes. Results from single slices as obtained after single stimuli (SS) or after burst stimuli (TBS). Overview on final results after averaging 4 slices. SD=standard deviation; SEM=standard error of mean. P<=Wilcoxon Mann Whitny U-Test. ACSF=artificial cerebro-spinal fluid.

Report NCAG 15/17 H – Hippocampus slice preparation

NCAG 1517															
**slice**	**ACSF +SS**					**Bryonia alba 1.25 mg/l + SS**					**Bryonia alba 1.25 mg/l + TBS**				
**time [min]**	**10**	**20**	**30**	**40**	**Mean 20-40**	**50**	**60**	**70**	**80**	**Mean 60-80**	**90**	**100**	**110**	**120**	**Mean 100-120**
**1**	-1007,00	-1115,00	-903,67	-950,26	**-989,64**	-2412,00	-2244,00	-2218,00	-1763,00	**-2075,00**	-4276,00	-3943,00	-4139,00	-3906,00	**-3996,00**
**2**	-1186,00	-1170,00	-937,81	-1046,00	**-1051,27**	-2246,00	-1863,00	-2100,00	-2231,00	**-2064,67**	-3362,00	-3647,00	-4050,00	-4150,00	**-3949,00**
**3**	-820,78	-1145,00	-1014,00	-958,76	**-1039,25**	-1260,00	-2351,00	-2119,00	-1972,00	**-2147,33**	-4096,00	-3735,00	-3837,00	-3840,00	**-3804,00**
**4**	-492,98	-1013,00	-916,66	-864,48	**-931,38**	-1834,00	-1880,00	-1971,00	-1991,00	**-1947,33**	-3588,00	-3741,00	-3815,00	-3914,00	**-3823,33**
**Mean**	-876,69	-1110,75	-943,04	-954,88	**-1002,89**	-1938,00	-2084,50	-2102,00	-1989,25	**-2058,58**	-3830,50	-3766,50	-3960,25	-3952,50	**-3893,08**
**SD**	296,09	68,94	49,36	74,19	**54,63**	513,17	249,90	101,51	191,43	**82,78**	427,11	125,27	159,47	135,78	**94,02**
**SEM**	148,05	34,47	24,68	37,09	27,31	256,59	124,95	50,75	95,71	41,39	213,55	62,63	79,74	67,89	47,01
**P<**	n.s.	n.s.	n.s.	n.s.	n.s.	0.01	0.01	0.01	0.01	0.01	0.02	0.01	0.01	0.01	0.01

Tab. 48 Effects of Bryonia alba 1.25 mg/l on pyramidal cell activity in terms of changes of population spike amplitudes. Results from single slices as obtained after single stimuli (SS) or after burst stimuli (TBS). Overview on final results after averaging 4 slices. SD=standard deviation; SEM=standard error of mean. P<=Wilcoxon Mann Whitny U-Test. ACSF=artificial cerebro-spinal fluid.

NCAG 1517															
**slice**	**ACSF +SS**					**Bryonia alba 1.50 mg/l + SS**					**Bryonia alba 1.50 mg/l + TBS**				
**time [min]**	**10**	**20**	**30**	**40**	**Mean 20-40**	**50**	**60**	**70**	**80**	**Mean 60-80**	**90**	**100**	**110**	**120**	**Mean 100-120**
**1**	-961,97	-933,77	-1133,00	-1116,00	**-1060,92**	-1498,00	-1812,00	-1862,00	-2247,00	**-1973,67**	-3359,00	-4677,00	-4529,00	-3871,00	**-4359,00**
**2**	-1083,00	-1214,00	-1083,00	-907,07	**-1068,02**	-1422,00	-2228,00	-1848,00	-2406,00	**-2160,67**	-2887,00	-4096,00	-4061,00	-4072,00	**-4076,33**
**3**	-1257,00	-1182,00	-1116,00	-961,30	**-1086,43**	-1900,00	-1921,00	-2210,00	-2084,00	**-2071,67**	-2894,00	-3794,00	-3843,00	-3752,00	**-3796,33**
**4**	-1068,00	-1138,00	-875,61	-1100,00	**-1037,87**	-1839,00	-2276,00	-2523,00	-2012,00	**-2270,33**	-3803,00	-4591,00	-4036,00	-3764,00	**-4130,33**
**Mean**	-1092,49	-1116,94	-1051,90	-1021,09	**-1063,31**	-1664,75	-2059,25	-2110,75	-2187,25	**-2119,08**	-3235,75	-4289,50	-4117,25	-3864,75	**-4090,50**
**SD**	122,19	126,03	119,35	102,97	**20,08**	239,75	227,82	321,83	175,87	**126,49**	437,94	417,94	291,27	148,16	**231,24**
**SEM**	61,09	63,01	59,67	51,49	10,04	119,87	113,91	160,91	87,94	63,25	218,97	208,97	145,64	74,08	115,62
**P<**	n.s.	n.s.	n.s.	n.s.	n.s.	0.01	0.01	0.01	0.01	0.01	0.02	0.01	0.01	0.01	0.01

Tab. 49 Effects of Bryonia alba 1.50 mg/l on pyramidal cell activity in terms of changes of population spike amplitudes. Results from single slices as obtained after single stimuli (SS) or after burst stimuli (TBS). Overview on final results after averaging 4 slices. SD=standard deviation; SEM=standard error of mean. P<=Wilcoxon Mann Whitny U-Test. ACSF=artificial cerebro-spinal fluid.

Report NCAG 15/17 H – Hippocampus slice preparation

NCAG 1517															
slice	ACSF +SS					RR-EUR-S 5.00 mg/l+ WS KSM66 0.50 mg/l SS					RR-EUR-S 5.00 mg/l+ WS KSM66 0.50 mg/l TBS				
time [min]	10	20	30	40	Mean 20-40	50	60	70	80	Mean 60-80	90	100	110	120	Mean 100-120
1	-782,76	-1057,00	-1029,00	-1088,00	**-1058,00**	-2362,00	-1814,00	-1951,00	-2186,00	**-1983,67**	-4000,00	-4268,00	-4953,00	-4819,00	**-4680,00**
2	-1129,00	-1138,00	-1246,00	-1190,00	**-1191,33**	-1785,00	-2190,00	-2272,00	-2251,00	**-2237,67**	-3124,00	-3602,00	-3608,00	-4282,00	**-3830,67**
3	-539,03	-1171,00	-1190,00	-1001,00	**-1120,67**	-1622,00	-1953,00	-2014,00	-1964,00	**-1977,00**	-3814,00	-3961,00	-3708,00	-3946,00	**-3871,67**
4	-1186,00	-1306,00	-1299,00	-1366,00	**-1323,67**	-2178,00	-1990,00	-2460,00	-2593,00	**-2347,67**	-4030,00	-4174,00	-4490,00	-4476,00	**-4380,00**
Mean	-909,20	-1168,00	-1191,00	-1161,25	**-1173,42**	-1986,75	-1986,75	-2174,25	-2248,50	**-2136,50**	-3742,00	-4001,25	-4189,75	-4380,75	**-4190,58**
SD	304,38	103,72	116,81	156,84	**114,02**	342,12	155,24	235,75	260,47	**185,85**	422,93	295,53	643,74	365,10	**410,96**
SEM	152,19	51,86	58,41	78,42	57,01	171,06	77,62	117,87	130,23	92,93	211,47	147,77	321,87	182,55	205,48
P<	n.s.	n.s.	n.s.	n.s.	n.s.	0.01	0.01	0.01	0.01	0.01	0.01	0.01	0.01	0.01	0.01

Tab. 50 Effects of the combination RR-EUR-S 5.00 mg/l and WS KSM66 0.50 mg/l on pyramidal cell activity in terms of changes of population spike amplitudes. Results from single slices as obtained after single stimuli (SS) or after burst stimuli (TBS). Overview on final results after averaging 4 slices. SD=standard deviation; SEM=standard error of mean. P<=Wilcoxon Mann Whitny U-Test. ACSF=artificial cerebro-spinal fluid.

NCAG 1517															
slice	ACSF +SS					RR-EUR-S 5.00 mg/l+ Bryonia alba 0.75 mg/l SS					RR-EUR-S 5.00 mg/l+ Bryonia alba 0.75 mg/l TBS				
time [min]	10	20	30	40	Mean 20-40	50	60	70	80	Mean 60-80	90	100	110	120	Mean 100-120
1	-876,36	-863,06	-1163,00	-1126,00	**-1050,69**	-677,83	-2064,00	-1710,00	-1870,00	**-1881,33**	-2717,00	-3093,00	-3284,00	-3189,00	**-3188,67**
2	-716,27	-1109,00	-893,58	-1197,00	**-1066,53**	-1925,00	-1701,00	-1715,00	-1909,00	**-1775,00**	-2548,00	-3229,00	-3528,00	-2967,00	**-3241,33**
3	-150,00	-1156,00	-1154,00	-1013,00	**-1107,67**	-1506,00	-2102,00	-2270,00	-2246,00	**-2206,00**	-3665,00	-3599,00	-3258,00	-3350,00	**-3402,33**
4	-879,98	-857,59	-1181,00	-1181,00	**-1073,20**	-1964,00	-1727,00	-1938,00	-1737,00	**-1800,67**	-2949,00	-3077,00	-3590,00	-3170,00	**-3279,00**
Mean	-655,65	-996,41	-1097,90	-1129,25	**-1074,52**	-1518,21	-1898,50	-1908,25	-1940,50	**-1915,75**	-2969,75	-3249,50	-3415,00	-3169,00	**-3277,83**
SD	345,64	158,32	136,67	83,25	**24,03**	597,38	213,87	263,56	216,57	**198,73**	491,79	242,77	168,53	157,02	**90,89**
SEM	172,82	79,16	68,34	41,63	12,02	298,69	106,93	131,78	108,28	99,37	245,89	121,39	84,26	78,51	45,45
P<	n.s.	n.s.	n.s.	n.s.	n.s.	0.01	0.01	0.01	0.01	0.01	0.02	0.01	0.01	0.01	0.01

Tab. 51 Effects of the combination RR-EUR-S 5.00 mg/l and Bryonia alba 0.50 mg/l on pyramidal cell activity in terms of changes of population spike amplitudes. Results from single slices as obtained after single stimuli (SS) or after burst stimuli (TBS). Overview on final results after averaging 4 slices. SD=standard deviation; SEM=standard error of mean. P<=Wilcoxon Mann Whitny U-Test. ACSF=artificial cerebro-spinal fluid.
