# Supplementary material for: Two Sides of the Same Coin for Health: Adaptogenic Botanicals as Nutraceuticals for Nutrition and Pharmaceuticals in Medicine
Source: Pharmaceuticals (Basel). 2025 Sep 8;18(9):1346. doi: 10.3390/ph18091346 (PMC12472958; doi:10.3390/ph18091346)

ASSAY

# of sitoindosides VII,VIII and withasomnine in

Withania Root

A.Panossian, G.Mamikonian, A.Danielian

Guielbenkian Laboratory of ADMTA, Yerevan, Armenia

Sponsored by Swedish Herbal Institute , Gothenburg, Sweden

2001

Introduction

Withania somnifera has been called “Indian ginseng”, and much like ginseng, it is used to restore vitality and treat nervous exhaustion. Dioscoride in the 1st century AD recommended Withania as a tonic. Today, it is mainly used in the West as a restorative for the elderly and the chronically ill. By reducing over activity and encouraging rest and relaxation, Withania is useful in countering the debility that accompanies long-term stress. In both Ayurveda and Unani systems Withania have been employed as a nervine tonic, aphrodiasic, rejuventor, as a sedative in the treatment of neurological, mental disorders, insanity, epilepsy and in certain forms of hypertension. Trials in 1980 indicated that Withania is useful for anemia mail fertility, and chronic illness. [Chevallier, 1996; Evans, 1996; Kapoor, 1990; Rai and Chaturvedi , 1981].

Research

1. **Anti-stress, anxiolytic-antidepressant activity have** alcoholic extract of the root, and glycowityhanolids - combination **of sitoindosides VII-X** and **withaferin A,** [Bhattacharya et. al, 1987; 2000; Singh et al., 1982;]:
   - elevated plus-maze test in rats,
   - social interaction test in rats,
   - novelty-suppressed feeding latency test in rats,
   - rat brain tribulin activity,
   - forced swimming-induced “behavioral despair’ test in rats,
   - “learned helplessness” test in rats,
   - Rotarod test in rats,
   - immobility in mice,
   - restraint stress-induced gastric ulcers in rats,
   - restraint stress-induced autoanalgesia in rats,
   - restraint stress effect on thermic response of morphine in rats,
   - pentylenetetrazol-induced physiological response in rats,
   - morphyne-induced toxicity in aggregated mice,
   - tribulin output in rat urine,
   - adrenocortical activity in rats,
   - milk induced leukocytosis.
2. Anti-anxiety activity shows alcoholic extract of the root in patients of anxiety neurosis (one month therapy) [Singh and Malviya, 1978; Shukla, 1981]:
   symptomatic relief,
   immediately memory span increase,
   reduction in the total and differential anxiety level,
   reduction in the degree of extroversion and increase in introversion,
   improvement in mental function,
   reduction in the level of plasma cortisol, and urinary catecholamines,
   increased body weight,
   reduction in mental fatigue.
3. **Psychotropic action shows** alcoholic extract of the root in mice [Day and Chatterjee, 1968; Singh et al. 1979; Yidya Prabhu et al., 1990]:

- induction of somnolence state, less response to tactile, auditory or visual stimulus, reduction in locomotor activity,
- strong potentiation of thiopental induced sleep,
- potentiation of barbiturate hypnosis,
- a marked delay in the onset of the semicarbazide and pentyenetetrazole induced seizure (no effect in strychnine and metrazol induced convulsions),
- inhibition the mescaline induced scratching episode.
- reduction catecholamines and acetylcholine and increase of histamine and 5-HT in brain tissue (no hypotensive effect).

Active principles

Alkaloids:.Somniferine, somniferinine, somnine, withasomnine. [Majumdar and Guha,1933; Schroter et al., 1966]. **Whitania alkaloids are sedative**, reduced blood pressure and lower the beat rate.

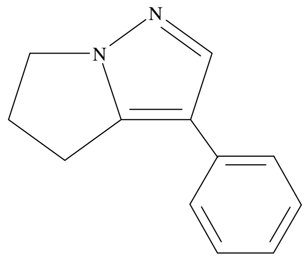

5,6-Dihydro-3-phenyl-4H-pyrrolo[1,2-b]pyrazole
Synonyms: **Withasomnine**. Newbouldine
Molecular Formula: C12H12N2
Molecular Weight: M 184.240.

Isolation and structure (uv, ir, ms, pmr): Schroter, H-B, Newmann D., Katritzky A.R., Swinbourne F.J., Withasomnine. A pyrasole alkaloid from Withania somnifera Dun. *Tetrahedron*,1966, 22, 2895 –7

Steroids: glycowithanolides comprising of **Sitoindosides VII, VIII (acylsterylglucosideas) and IX, X (acylwithanolylglucosides) which have anti-stress activity [Bhattacharya et. al, 1987].**

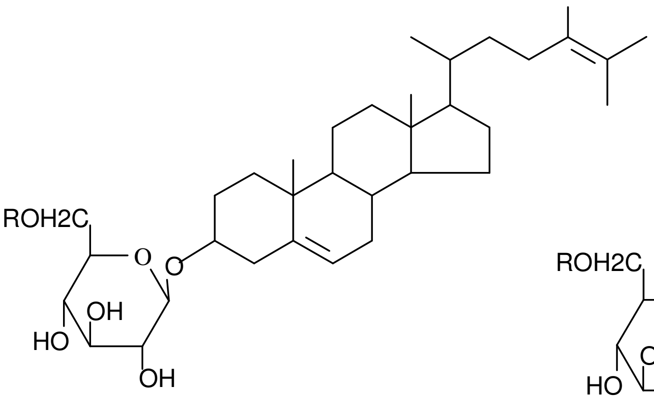

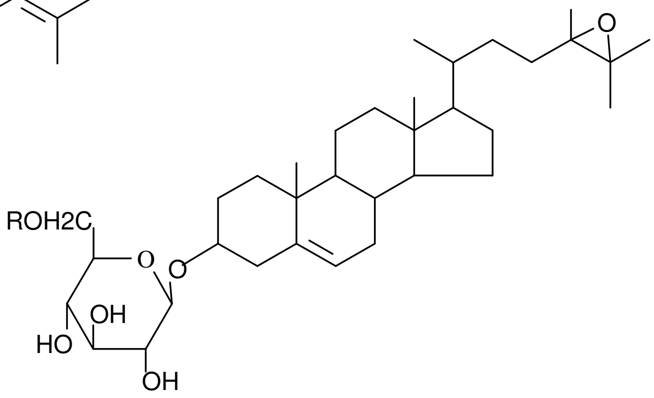

Sitoindoside VII R-palmitoyl *Sitoindoside VIII*

Isolation and structure: Bhattacharya, S.K., Goel R.K., Kaur R., Ghosal S. Anti-stress activity of sito-indosides VII and VIII, new acylsterylglycosides from Withania somnifera: Phytotherapy Res. 1(1), 32-37, 1987.

Withanolides: Though more then 30 withanolides have been isolated from Withania somnifera and much work has been done on the chemistry of withanolides, only limited reports, mainly on **withaferin A** are available on the biological studies of these steroids [Lavie et al., 1965; Kundu et al., 1976; Nittala and Lavie, 1981]. These reports show **that withanolides possess promising antiinflammatory, antitumour, immunomodulatory e**ffects in vitro and in vivo studies [Dudhiraja and Sudhir, 1987].

Though Withania somnifera is described in Indian pharmaceutical codex and included as a monograph in the Indian Pharmacopoeia [The Pharmacopoiea of India, 1956, Monograph on Ashwaganhdha, p. 748] it is important to prepare an updated version of The Monography on standardized drug. Only three reports have been published where the methods of analysis and standardization were described [Bessalle and Lavie, 1987; Ghosal, 1986; Bhattacharya et al., 2000]. However, a standardized drug and an appropriate methods of analysis and standardization still have to be developed.

REFERENCES

Bhattacharya S.K., Goel R.K., Kaur R., Ghosal S.: Anti-stress activity of Sitoindosides VII and VIII, New acylsterylblucosides from Withania somnifera. Phytotherapy research, 1(1), 32-37, 1987.

Bhattacharya S.K., Bhattacharya S.K., Sairam K., Ghosal S.: Anxiolytic-antidepressant activity of Withania somnifera glycowithanolides: an experimental study, Phytomedicine 7(6): 463-469, 2000.

Day P.K., Chatterjee B.K.: Studies on the neuropharmacological properties of several Indian medicinal plants. Jour.Res.Ind.Med. 3(1): 9-17, 1968.

Ghosal S., Lal J., Srivatava R., Battacharya R., Upadhyay S.N., Jaiswal A.K., Chattopadhyay U.: Sitoindosides IX and X, New glycowithanolides from Withania somnifera. *Phytotherapy Res.* 3(5): 201- ,1989.

Shuka S.P.: Anti-anxiety agents of plant origin. *Probe* : 201-208, 1981

Singh N., Nath R., Lata A., Singh S.P., Kohli R.P., Bhargava K.P.: Withania somifera (Ashwagandha), a rejuvenating herbal drug which enhances survival during stress (anadaptogen). *Int.J. Crude Drug Res*. 20(1): 29-35, 1982..

Singh R.H., Malviya P.C.: Studies on the psychotropic effect of an indigenous rasayana drug, Asvagandha (Withania somnifera dunol) Part I: (Clinical studies). *Jour.Res.Ind. Med.Yoga & Homeo*. 13(1): 15-24, 1978.

Singh R.H., Malviya P.C., Sarkar F.H., Udupa K.N.: Studies on the psychotropic effect of an indigenous rasayana drug, Asvagandha (Withania somnifera dunol) Part II: (Experimental studies). *Jour.Res.Ind. Med.Yoga & Homeo*. 14(1): 49-54, 1979.

Yidya Prabhu M., Rao A.: Neuropharmacological activity of Withania somnifera. *Fitoterapia.* 66(3): 237-240, 1990.

Rajiv Asthana, Raina M.K.: Pharmacology of Withania somnifera (Linn) Dunal - a review. *Indian Drugs* 26(5): 199-205, 1989.

Budharaja R.D., Sudhir.S.: Review of biological activity of withanolides. J.Sci. & Ind.Res.46: 488-491, 1987.

Kundu A.B., Mukherjee A., Dey A.K.: Recent Developments in the Chemistry of Withanolides. J.Scient.Ind.Res. 35: 616-626, 1976.

Rai N.P. Chaturvedi G.N.: A medical study on Ashvagandha (Withania somnifera Dunal, Solanaceae). *Nagarjun* - May, 199-201, 1981

Evans W.C.: Trease and Evans Pharmacognosy. WB Saunders Coompany Ltd, London-Philadelphia-Toronto-Sydnay-Tokio. 1996, 612 p.

Kapoor L.D.: Handbook of Aurvedic Medicinal Plants. CRC Press Inc., Boca Raton, Florida, 1990, 416 p.

Nittala S.S., Lavie D.: Chemistry and genetics of witanolides in Withania somnifera hybrides. Phytochemistry 20(12), 2741-2748, 1981.

Majumdar D.N., Guha P.C.: On withania somnifera. J.Ind.Inst.Sc. 16A: 29-33,1933.

Schroter H.-B., Neumann D., Katrizky A.R., Swinbourne F.J.: Withasomnine. A Pyrazole alkaloid from Withania somnifera Dun. Tetrahedron. 22: 2895-1897, 1966.

Lavie D., Glotter E., Shvo Y.: Costituents of Withania somnifera Dun. Part IV. The structure of Withaferin A. J.Chem.Soc.:7517-7531, 1965.

Bessalle R., Lavie D.: Semi-quantitative reversed-phase high-performance liquid chromatographic analysis of the ecotypes of Withania somnifera chemotype III. J.Chromatography. 389: 195-210, 1987.

Ghosal S.: In purruit of standardization of Ayurvedic Drugs. Ann.NatlAcad.Ind.Med. 1(1): 1-13, 1986.

PRINCIPLE

Phytochemical analysis of Withania root extracts show that they contain a complex mixture of acylated steroids glycosides (see above and attachment I). It is unlikely that one of them is active and others inactive. Moreover, biological activity of Withania was shown for their mixture. Therefore it is worthwhile to standardize the Withania extracts (or row material) for total sitoindosides, but not for a particular component.
For this purpose a suitable method of analysis of the total sitoindosides content in extracts is the method based on Liberman-Burchard reaction. In this method, sterines are treated with acid reagent under controlled conditions and the colored complex thus obtained is measured photometrically in the maximum at 662 nm. The calculation of the total sterods content is carried out according to the external standard method against cholesterol as a standard sterol in the maximun at 620 nm.

We developed this method using for analysis an purified fraction of sitoindosides and cholesterol as standards for calculation of the total sitoindosides content.

REAGENTS AND MATERIALS

Quality grades and listed below are currently being used.

A) Sulfuric acid 96%(UN 1830), PENTA
B) Glacial Acetic acid (Art 3738.3), ROTH
C) Methanol,(Cat.No: 17,933-7), ALDRICH
D) Petroleum ether
E) Cholesterol
F) Ashwaganda (*Withania somnifera* Dun root) , Swedish Herbal Institute, Gothenburg
G) **Fraction of sitoindosides** of *Withania somnifera* Dun (Attachment 1).

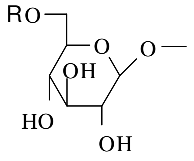

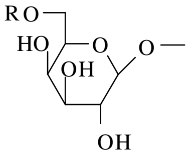

R – Fatty acids:
- Myristic (C-14)
- Palmitic (C-16)
- Stearic (C-18)
- Oleic (C-18:1)
- Linolic (C-18:2)
- Arachidic (C-20:0)
- Behenic (C-22:0)
- Azelaic (C-6 dicarboxy)

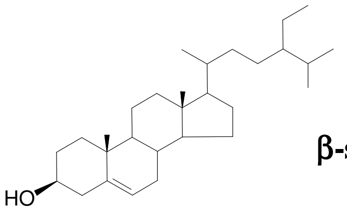

β-sitosterol

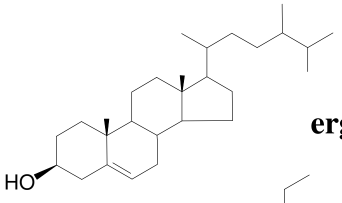

ergost-5-en-3βol

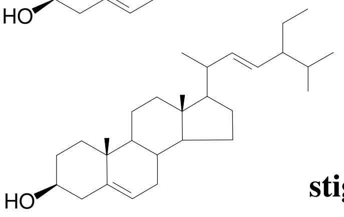

stigmasterol

INSTRUMENTATION
BECKMAN DU-640i Spectrophotometer.

Instrumental parameters
Samples are analyzed by BECKMAN DU-640i Spectrophotometer according to the conditions mentioned below:

Start wavelength	:	*λ=400.0 nm*
End wavelength	:	*λ=800.0 nm*
Scans per sample	:	*35.00 sec*
Scan speed	:	600 *nm/min*

PROCEDURE

1. Standard Solution - SS

Weight accurately 10.0 mg of cholesterol into 20 ml volumetric flask. Dissolve and dilute to the volume with methanol (500mg/l). Evaporate 1.0 ml of this solution to dryness and add to the residue 100 µl of glacial acetic acid and 5.0 ml of Libermann-Burchards' (LB) reagent , shake the sample for 2 min. Measure the absorption of sample within 5-10 min in quartz glass cuvettes at 620 nm (UV- spectra – Figure 1). As the blank solution use a mixture of 100 µl of glacial acetic acid and 5.0 ml of LB reagent

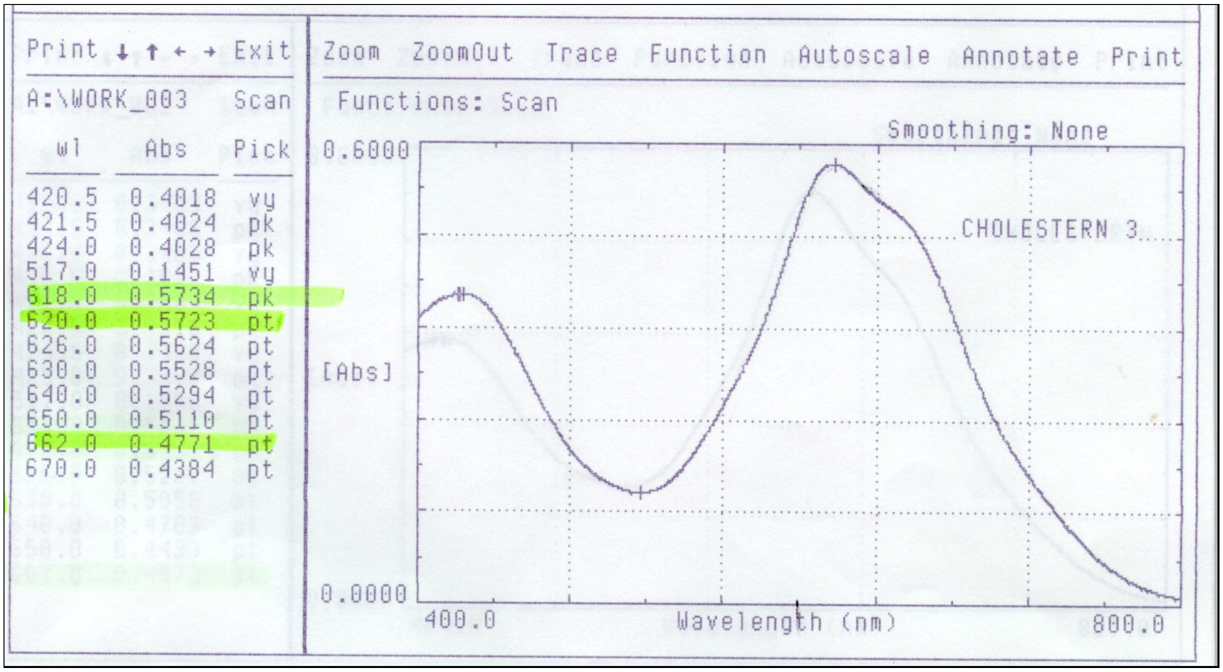

2. Analyte Solution - AS

Weight accurately about 10.0 g of powdered roots in Erlenmeyer flask. Add 30 ml of petroleum ether, shake vigorously for 10 min, sonicate for 15 min and remove the petroleum ether extract. Repeat extraction procedure twice again by the same way. Combined extracts filter into the 100ml volumetric flask and bring up to the volume in with petroleum ether. Evaporate 10.0 ml of this solution to dryness and add to the residue 100 µl of glacial acetic acid and 5.0 ml of color reagent, shake the sample for 2 min. Measure the absorption of sample within 5-10 min in quartz glass cuvettes at 662 nm (UV- spectra – Figure 2) . As the blank solution use a petroleum ether extract of powdered roots

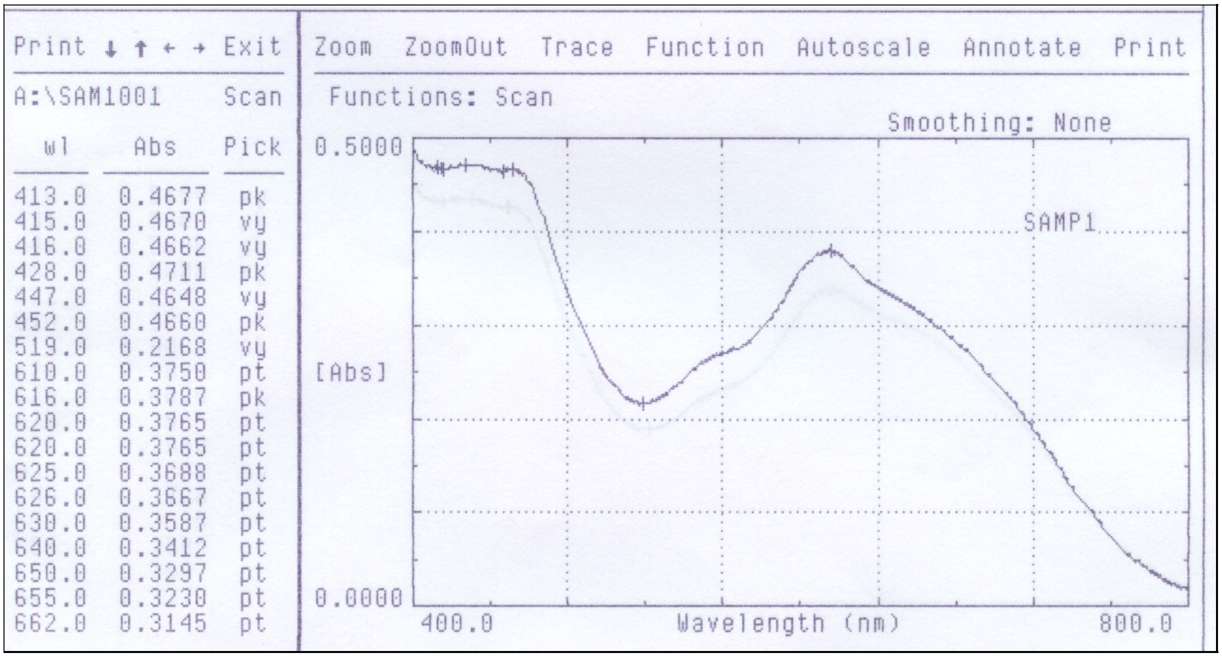

3. Sitoindosides Standard Solution - SSS

Weight accurately 10.0 mg of fraction of Sitoindosides into 20 ml volumetric flask. Dissolve and dilute to the volume with chloroform (500mg/l). Evaporate 1.0 ml of this solution to dryness and add to the residue 100 µl of glacial acetic acid and 5.0 ml of Liberman-Burchards' (LB) reagent , shake the sample for 2 min. Measure the absorption of sample within 5-10 min in quartz glass cuvettes at 662 nm nm (UV- spectra – Figure 3). As the blank solution use a mixture of 100 µl of glacial acetic acid and 5.0 ml of LB reagent

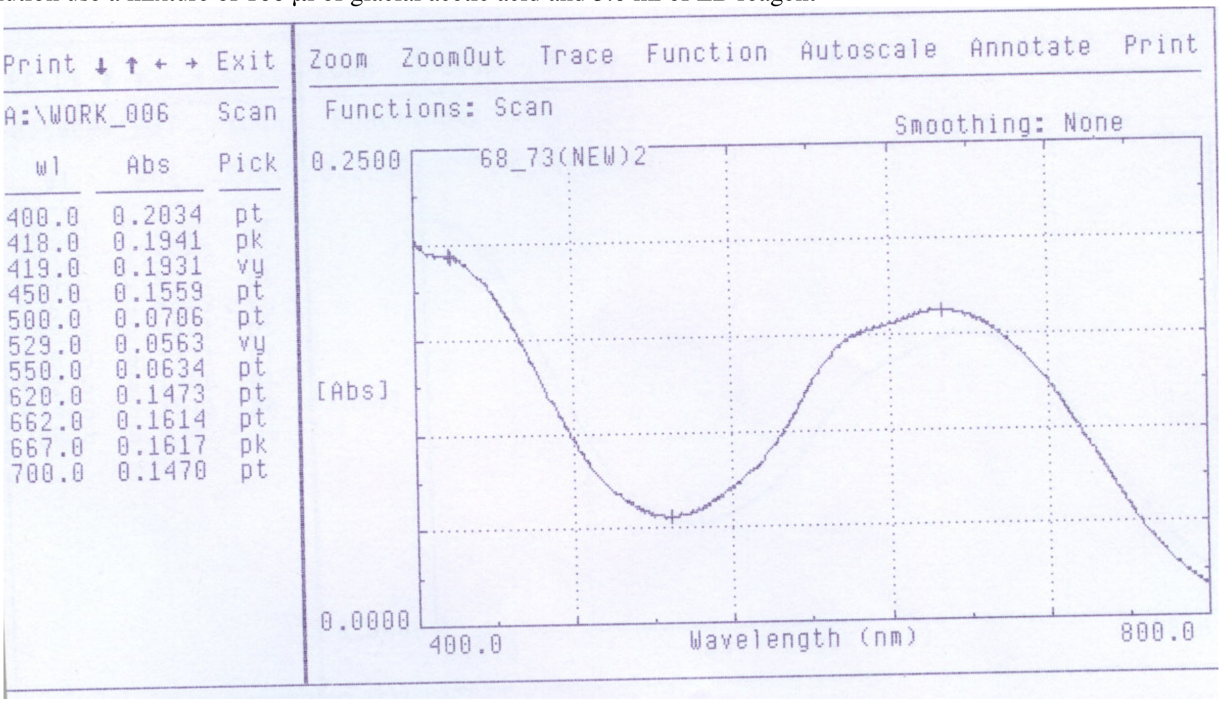

Libermann-Burchard (LB) reagent

Carefully mix 50.0 ml of acetic anhydride with 1.0 ml of 96 % sulfuric acid and shake the cooled mixture. The solution must be freshly prepared.

CALCULATION

Calculate the content of sitoindosides using the formula:

$$C = \frac{D_2 \bullet m_1 \bullet n_2}{A_1 \bullet m_2 \bullet n_1 \bullet (100-h)/100} \bullet k = \frac{D_2 \bullet m_1}{D_1 \bullet m_2 \bullet 2 \bullet (100-h)/100} \bullet k,$$

where

C - content of the sitoindosides in the Withania root, (mg/g);
D_1 - optical density of cholesterol standard solution SS (λ=620 ± 5 nm);
D_2 - optical density of AS (λ=620 ± 5 nm);
m_1 - weight of cholesterol standard (mg);
m_2 - weight of the powered roots sample (g);
n_1 - dilution of cholesterol standard solution SS (=100);
n_2 - dilution of AS (=50);
h - loss of weight after drying, %
k - ratio of optical density of the equal concentrations of cholesterol and fraction of sitoindosides (=3.398),

k can be obtained by formula

$$k = \frac{C_1 \bullet D_2}{D_1 \bullet C_2},$$

Where,

C_1 - concentration of sitoindosides in SSS (0.1mg/ml)
C_2 - concentration of the cholesterol in SS (0.1mg/ml)
D_1 - optical density of sitoindosides in SSS (λ=662 ± 5 nm)
D_2 - optical density of the cholesterol in SS (λ=620 ± 5 nm)

Absorption of the Fraction of sitoindosides (SSS, 0.1mg/ml) (Table 1; Figures 1.a-c)

Table 1.

N	File name	Sample name	D=ε • c • l at λ=620±5 nm	D=ε • c • l at λ=662±5 nm
1	WORK_005	SSS- 6873 (1)	0.1670	0.1627
2	WORK_006	SSS- 6873 (2)	0.1473	0.1614
3	WORK_007	SSS- 6873 (3)	0.1271	0.1486
Mean			**0.1471**	**0.1576**
Standard deviation			0.01995	0.007793
Standard error			0.01152	0.004499
Relative standard deviation (%)			13.56%	4.95%
Confidence interval of the mean			0.09757-0.1967	0.1382-0.1769

Absorption of the cholesterol (0.1mg/ml) (Table 2; Figures 2.a-c)

Table 2.

N	File name	Sample name	D= ε • c • l at λ=620±5 nm	D= ε • c • l at λ=662±5 nm
1	WORK_002	Cholesterol (1)	0.5384	0.4073
2	WORK_002	Cholesterol (2)	0.4961	0.4133
3	WORK_002	Cholesterol (3)	0.5723	0.4771
Mean			**0.5356**	**0.4326**
Standard deviation			0.03818	0.03868
Standard error			0.02204	0.02233
Relative standard deviation (%)			7.13%	8.94%
Confidence interval of the mean			0.4408 - 0.6304	0.3365 - 0.5287

Validation of the Method

Precision

A. System repeatability

The system repeatability is characterized by mean, the standard deviation, the relative standard deviation and the error of the mean calculated on a serie of n=6 determinations of the same solution of the sample. (Table 3; Figures 3.a-f)

Table 3

N	File name	Sample name	D= ε • c • l at λ=620±5 nm	D= ε • c • l at λ=662±5 nm
1	LIN017	Cholesterol 1	0.3115	0.4270
2	LIN018	Cholesterol 1	0.3017	0.4196
3	LIN019	Cholesterol 1	0.2966	0.4161
4	LIN020	Cholesterol 1	0.2933	0.4134
5	LIN021	Cholesterol 1	0.2883	0.4094
6	LIN022	Cholesterol 1	0.2831	0.4051
Mean			0.2958	0.4151
Standard deviation			0.01006	0.007723
Standard error			0.004106	0.003153
Relative standard deviation (%)			3.40%	1.86%
Confidence interval of the mean			0.2852 - 0.3063	0.4070 - 0.4232

B. System reproducibility

The reproducibility of the method is characterized by mean, the standard deviation, the relative standard deviation, the error of the mean by analysis (n=6) of the different preparations of the standard solution (Table 4; Figure 4 a-f).

Table 4

N	File name	Sample name	Optical density of cholesterol solution, D_1 at	
			λ=620±5 nm	λ=662±5 nm
1	CHOL001	Cholesterol -1	0.3570	0.3165
2	CHOL002	Cholesterol -2	0.3950	0.3564
3	CHOL003	Cholesterol -3	0.4413	0.4068
4	CHOL2001	Cholesterol -4	0.4738	0.3451
5	CHOL2002	Cholesterol -5	0.5592	0.4301
6	CHOL2003	Cholesterol -6	0.5846	0.4626
Mean			0.4685	0.3863
Standard deviation			0.08979	0.05590
Standard error			0.03666	0.02282
Relative standard deviation (%)			19.17%	14.47%
Confidence interval of the mean			0.3743 - 0.5627	0.3276 - 0.4449

C. Repeatability of the method

The precision of the method is established by analysis of Withania somnifera extracts (n=6), which have been separately prepared from the same sample of Ashwaganda (Withania root, SHI, h (loss of drying) = 6.8%) under the conditions described in the method (Table 5; Figures 5.a-e).

Table 5

N	File name	λ=620±5 nm	λ=662±5 nm	C_1, 620/620	C_2, 662/662	**C_3, mg/g 620/662**
1	SAM1001	0.3787	0.3145	1.579	1.198	**1.224**
2	SAM1002	0.4031	0.3507	1.680	1.336	**1.365**
3	SAM1003	0.4072	0.3720	1.697	1.418	**1.447**
4	SAM1004	0.3224	0.2865	1.344	1.092	**1.115**
5	SAM1005	0.3688	0.3456	1.537	1.318	**1.344**
6	SAM1006	0.3888	0.3764	1.621	1.435	**1.465**
Mean, mg/g				1.576	1.300	**1.327**
Standard deviation				0.1287	0.1322	**0.1347**
Standard error				0.05255	0.05399	**0.05499**
Relative standard deviation (%)				8.17%	10.18%	**10.15%**
Confidence interval of the mean				1.441 - 1.711	1.161 - 1.438	**1.185 - 1.468**

Accuracy

The accuracy of the method is characterized by mean, the standard deviation, the relative standard deviation, the error of the mean by analysis of samples (n=5) obtained by addition of known amounts of fraction of Sitoindosides (0.1; 0.15; 0.25; 0.5 mg) to the 0.5 g of the Ashwaganda (Withania root, SHI) and prepared separately under the conditions described in the method (Table 6.a-c; Figures 6.a-e).

Table 6.a

N	File name	Sample name	Internal Sitoindo-sides, T= C_1 / 2 , mg	Externally added fraction of Sitoindo-sides added, FS mg	Total Sitoindo-sides content, S = T+FP mg	Optical density, D_2 λ=620±5 nm	Content of Sitoindosides [620/620] , mg $E_1= D_2 x E_{ch}{*} x\ k_1 / D_1 x\ (100-h^{**})/100$	Recovery, R=Ex100 / S %
1	WORK_01	AC-1	**0.788**	-	**0.788**	0.1912	0.798	101.27
2	WORK_02	AC-2	**0.788**	0.1	**0.888**	0.2008	0.838	94.37
3	WORK_03	AC-3	**0.788**	0.15	**0.938**	0.2341	0.976	104.05
4	WORK_04	AC-4	**0.788**	0.25	**1.038**	0.2541	1.060	102.12
5	WORK_05	AC-5	**0.788**	0.5	**1.288**	0.3085	1.202	93.32
Mean								99.03
Standard deviation								4.850
Standard error								2.169
Relative standard deviation (%)								4.90%
Confidence interval of the mean								93.00-105.0

***- E_{ch} –** weight of the cholesterol standard (=0.5 mg)

****-** h - loss of weight after drying, %

Table 6.b

N	File name	Sample name	Added			Optical density, D_2 λ=662±5 nm	Content of Sitoindosides [662/662] , mg $E_2 = D_2 x E_{ch}{}^{*} x\ k_1 / D_1 x\ (100-h^{**})/100$	Recovery, R=Ex100 / S %
			Internal Sitoindo-sides, T= C_1 / 2 , mg	Externally added fraction of Sitoindo-sides added, FS mg	Total Sitoindo-sides content, S = T+FP mg			
1	WORK_01	AC-1	**0.650**	-	**0.650**	0.1734	0.661	101.69
2	WORK_02	AC-2	**0.650**	0.1	**0.750**	0.1959	0.747	99.60
3	WORK_03	AC-3	**0.650**	0.15	**0.800**	0.2066	0.788	98.50
4	WORK_04	AC-4	**0.650**	0.25	**0.900**	0.2469	0.941	104.56
5	WORK_05	AC-5	**0.650**	0.5	**1.150**	0.3223	1.229	106.87
Mean								102.2
Standard deviation								3.467
Standard error								1.550
Relative standard deviation (%)								3.39%
Confidence interval of the mean								97.94 - 106.5

*** - E_{ch} –** weight of the cholesterin standard (=0.5 mg)

****-** h - loss of weight after drying, %

Table 6.c

N	File name	Sample name	Added			Optical density, D_2 λ=662±5 nm	Content of Sitoindosides [620/662] , mg $E_3 = D_2 x E_{ch}{}^{*} x\ k_1 / D_1 x\ (100-h^{**})/100$	**Recovery, R=Ex100 / S %**
			Internal Sitoindo-sides, T= C_1 / 2 , mg	Externally added fraction of Sitoindo-sides added, FS mg	Total Sitoindo-sides content, S = T+FP mg			
1	WORK_01	AC-1	**0.664**	-	**0.664**	0.1734	0.675	**101.66**
2	WORK_02	AC-2	**0.664**	0.1	**0.764**	0.1959	0.763	**99.87**
3	WORK_03	AC-3	**0.664**	0.15	**0.814**	0.2066	0.804	**98.77**
4	WORK_04	AC-4	**0.664**	0.25	**0.914**	0.2469	0.961	**105.14**
5	WORK_05	AC-5	**0.664**	0.5	**1.164**	0.3223	1.254	**107.73**
Mean								**102.6**
Standard deviation								**3.734**
Standard error								**1.670**
Relative standard deviation (%)								**3.64%**
Confidence interval of the mean								**98.00 - 107.3**

*** - E_{ch} –** weight of the cholesterin standard (=0.5 mg)

****-** h - loss of weight after drying, %

Linearity

The linearity of the method is performed by correlation between the amounts of Cholesterol and determined absorption (Table 7; Figures 7.a-i).

Table 7

Level	File name	Amount of Cholesterin, mg/ml	Optical density of cholesterol solution	
			λ=620±5 nm	λ=662±5 nm
1	LIN011	0.5	1.7707	2.2248
2	LIN009	0.2	0.7467	0.9003
3	LIN008	0.1	0.3980	0.4689
4	LIN016	0.04	0.1591	0.2176
5	LIN006	0.02	0.1062	0.1242
6	LIN004	0.01	0.0554	0.0651
7	LIN003	0.005	0.0294	0.0339
8	LIN002	0.002	0.0128	0.0141
9	LIN001	0.001	0.0077	0.0084
Variables				
Slope of the regression line			3.525 ± 0.03649	4.414 ± 0.02934
Y-intercept			0.02120 ± 0.006686	0.02018 ± 0.005375
X-intercept			-0.006013	-0.004570
1/slope			0.2837	0.2265
95% Confidence Intervals				
Slope			3.439 to 3.612	4.345 to 4.484
Y-intercept			0.005385 to 0.03701	0.007463 to 0.03289
Goodness of Fit				
r^2			0.9993	0.9997
S y.x			0.01698	0.01365
Is slope significantly non-zero?				
F			9332	22640
DFn, DFd			1.000, 7.000	1.000, 7.000
P value			< 0.0001	< 0.0001
Deviation from zero?			Significant	Significant
Residuals				
Points above line			3	4
Points below line			6	5
Number of runs			5	3
P value (runs test)			0.6429	0.0714
Significantly nonlinear?			Not Significant	Not Significant

The method has been shown to be linear in the range from 0.01 to 0.5 mg/ml. The regression analysis is shown in the table. The correlation coefficient was determined to be r= 0.9993 and r= 0.9997, showing a good linear fit within the range tested.

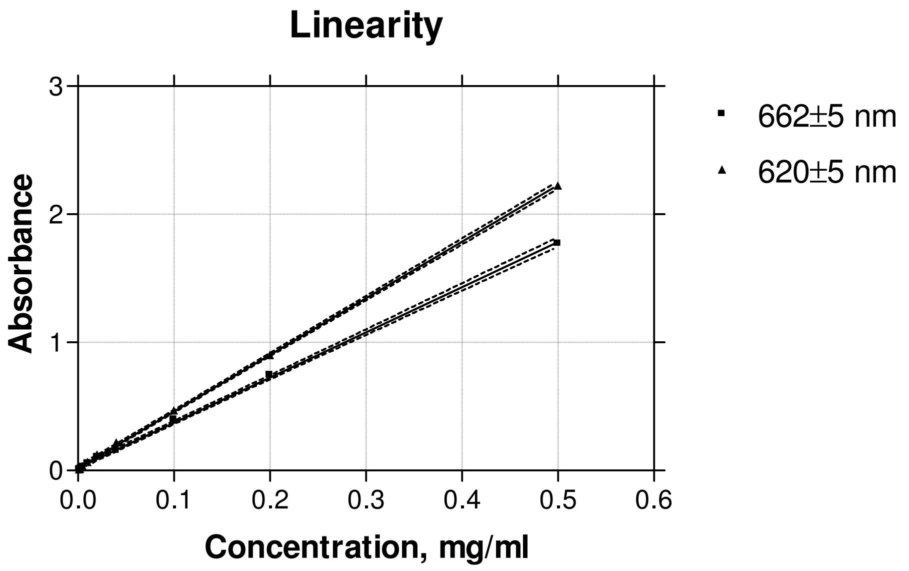

Limit of Detection

Limit of detection was found experimentally (Table 8; Figures 7.i; 8.a,b)

Table 8

Level	File name	Amount of Cholesterol, mg/ml	Optical density of cholesterol solution	
			λ=620±5 nm	λ=662±5 nm
9	LIN001	0.001	0.0077	0.0084
10	WORK_003	0.0002	0.0073	-
12	LIMBL001	0.00005	-	-

PRINCIPLE

The content of Withasomnine in Withania root is determined by High Performance Liquid Chromatography. Quantitation is performed by an internal standard method.

REAGENTS AND MATERIALS

Quality grades and listed below are currently being used.

H) Water, distilled
I) Acetonitrile, (Art. 7330.2), ROTH
J) Ethanol
K) Ethylacetate, (Un-No: 1173), RIEDEL-DeHa¸n
L) o-Phosphoric Acid, 85% (Art. 6366.1), ROTH
M) Chloroform, (Art. 7331.1), ROTH
N) Ashwaganda (Withania root), Swedish Herbal Institute, Gothenburg
O) Withasomnine
P) Diethylstilbestrol (D-4628, Lot 64H0690), SIGMA

INSTRUMENTATION

BECKMAN HPLC GOLD system consisting of:

Detector:	BECKMAN UV-Detector Module 166
Pump:	BECKMAN Double pumps Programmable Solvent Module 125
Injection Valve:	Rheodyne mod. 7725I with 20 µl loop.
Data Collection:	PS/1 Computer 486 DX-33 with management software supplied by Beckman; Epson FX - 800 printer.
Statistical analysis**:**	Prism software, version 2.0, GraphPad Software Inc.USA, 1996

Experimental conditions

Column	*LiChroCART 250x4 mm HPLC cartridge with Superspher60 RP-select B (Merck, Darmstadt)*	
Mobile phases	*Pump A - Water—0.1N phosphoric acid (99:1, v/v);*	
	Pump B - Acetonitrile-0.1N phosphoric acid (99:1, v/v)	
Gradient	*Min.*	*%B*
	00	*50*
	00 – 20	*50 – 50*
	20 – 21	*50 – 100*
	21 – 25	*100 -100*
	25 –26	*100 – 50*
	26	*50*
Flow rate	*0.7ml/min*	
Detection	*250 nm*	
Temperature	*Ambient (about 25^0 C)*	
Injection volume	*20µl*	

PROCEDURE

4. Internal Standard Stock Solution – ISSS

Weight accurately 20.0 mg of diethylstilbestrol into 50ml volumetric flask. Dissolve it with ethanol and dilute to the volume – 0.4 mg/ml. Transfer 1ml of this solution into 20ml volumetric flask and bring up to volume with ethanol. Concentration of diethylstilbestrol - 0.02 mg/ml.

5. Internal Standard Solution – ISS

Transfer 5 ml of ISSS into 10ml volumetric flask and bring up to volume with ethanol. Concentration of diethylstilbestrol – 0.01 mg/ml.

6. Withasomnine Standard Solution – WSS

Weight accurately 4.0 mg of withasomnine into 20ml volumetric flask. Dissolve with chloroform in ultrasonic bath for 5 min and dilute to the volume. Concentration of withasomnine – 0.2 mg/ml.

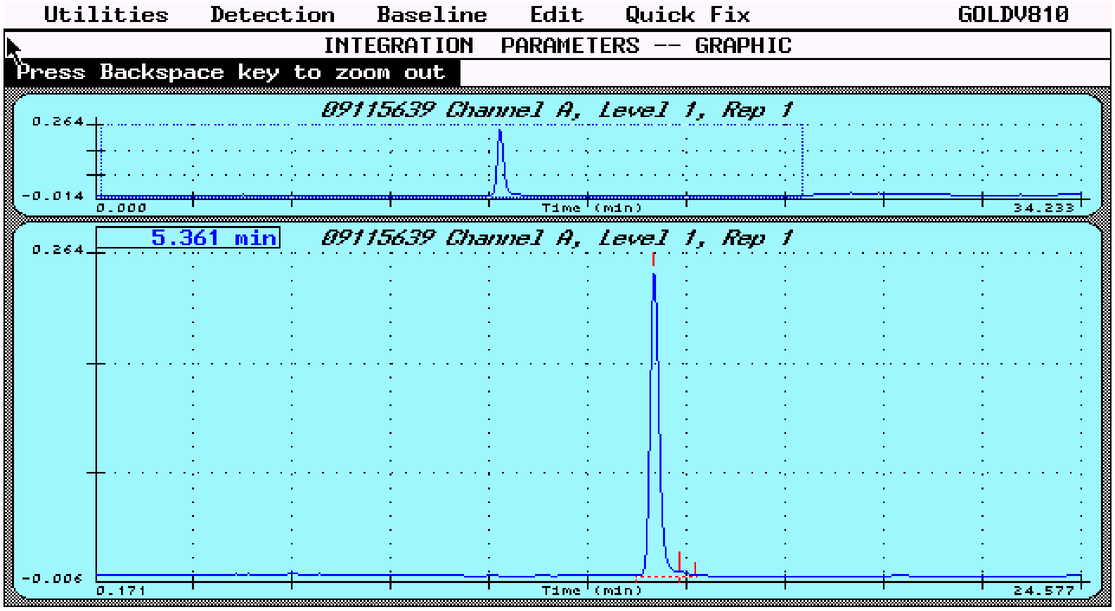

Figure 1. Chromatogram of the isolated Withasomnine.

7. Standard Solution – SS

Mix 5 ml of ISS-1 with 5 ml of WSS to obtain Standard Solution (SS).
Concentrations of diethylstilbestrol and withasomnine – 0.1 mg/ml

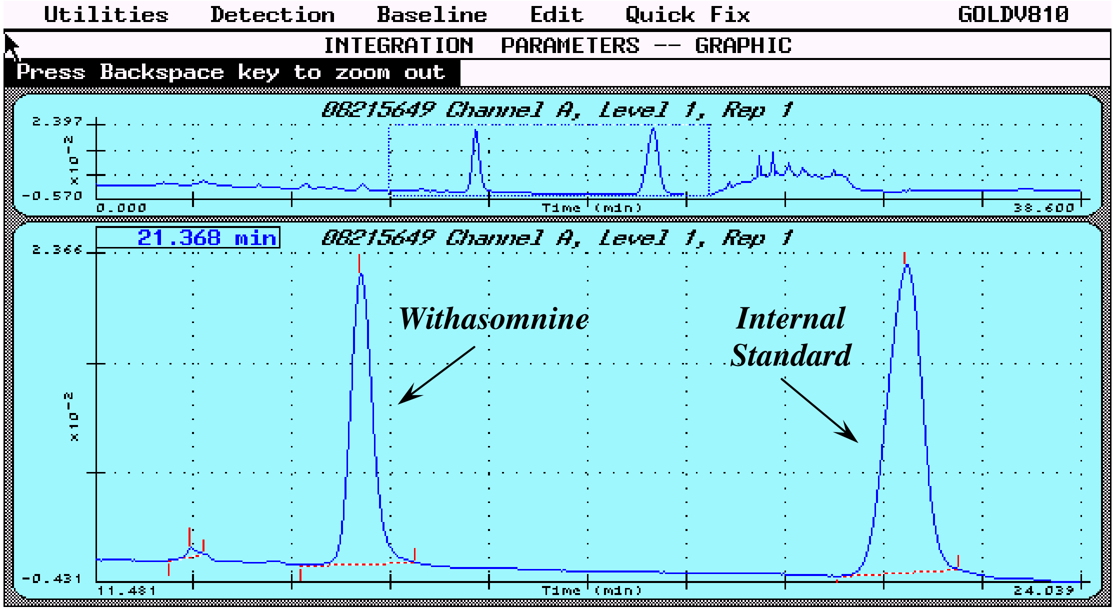

Figure 2. Chromatogram of the Standard Solution (SS).

8. Analyte Solution – AS

Weight accurately about 10.0 g of powdered roots in Erlenmeyer flask. Add 50 ml of ethylacetate, shake vigorously for 5 min, sonicate for 15 min and remove the ethylacetate extract. Repeat extraction procedure twice. Combine removed extracts, filter, add 1ml of Internal Standard Solution – ISS and evaporate to the dryness. Dissolve the residue with mobile phase (water—acetonitrile - 0.1N phosphoric acid (50:50:1, v/v/v)) by heating (t=50^{0}C) for 3-5 min and filter.

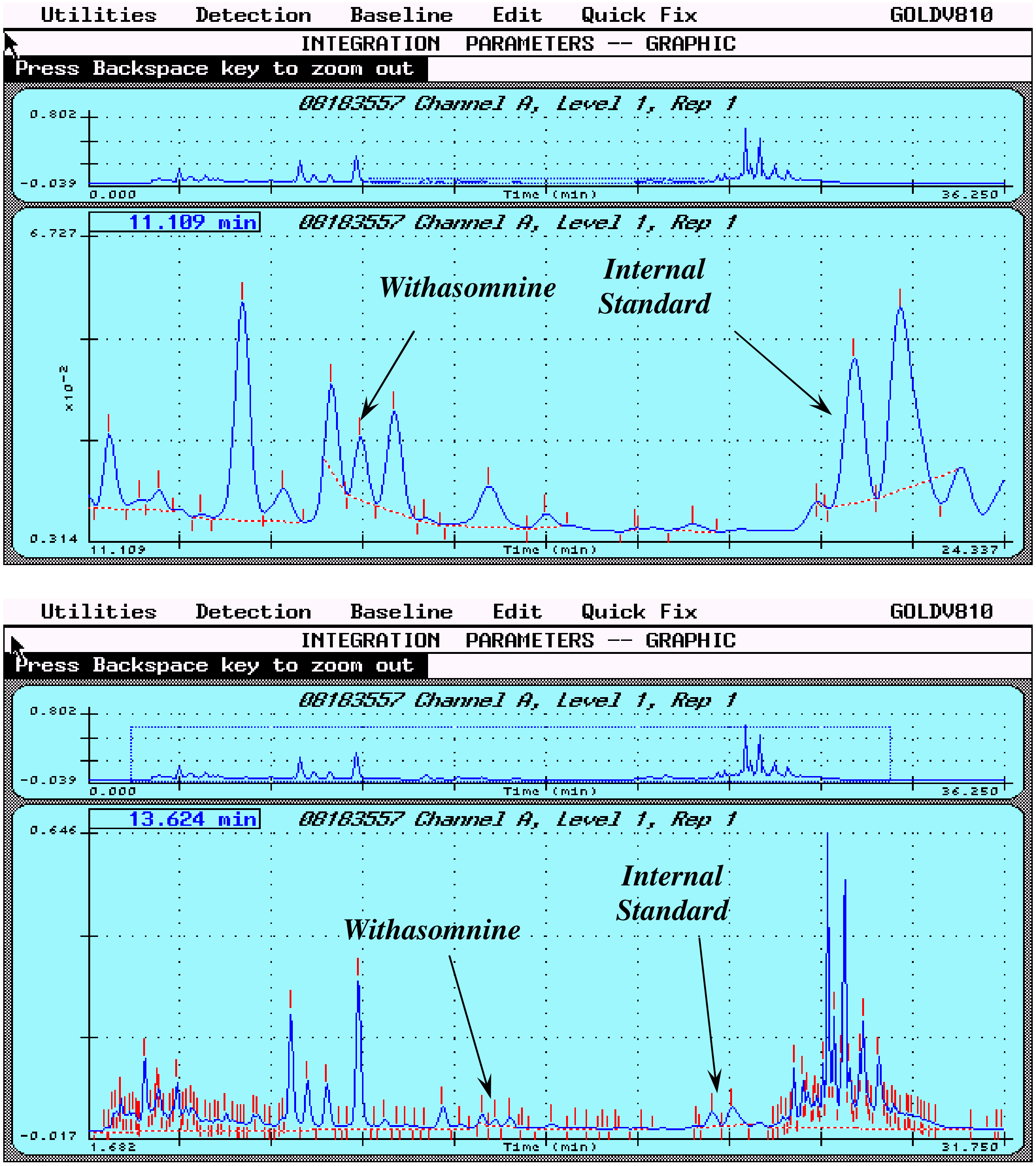

Figure 3. Chromatogram of the Analyte Solution (AS).

CALCULATIONS

$$M_{wit} = M_{IS} \bullet \frac{S_{wit}}{S_{IS} \bullet W \bullet (100-h)/100} \bullet \frac{1}{K_{wit}}$$

M_{wit} - content of the withasomnine in withania root, (mg/g)
M_{IS} - weight of internal standard in AS (=0.01mg)
S_{wit} - peak area of withasomnine on the chromatogram of AS
S_{IS} - peak area of diethylstilbestrol on the chromatogram of AS
W - weight of the test sample (g)
h - loss of weight after drying, %
K_{wit} - ratio of peak areas of withasomnine and internal standard on the chromatogram of Standard Solution (SS),

$$K_{wit} = \frac{S_{wit} \bullet C_{IS}}{S_{IS} \bullet C_{wit}},$$

C_{wit} - concentration of the withasomnine in SS, (0.1mg/ml)
C_{IS} - concentration of the internal standard in SS, (0.01mg/ml)
S_{wit} - peak area of withasomnine on the chromatogram of SS
S_{IS} - peak area of diethylstilbestrol on the chromatogram of SS

Validation of the Method

Precision

A. Repeatability of HPLC

The repeatability of HPLC is characterized by mean, the standard deviation, the relative standard deviation and the error of the mean calculated on a series of n=6 injections of the same standard solution. (Table 1)

Table 1.

N	File name	Sample name	Area of Internal Standard , S_{IS}	Area of Withasomnine peak , Swit	Ratio of peaks areas of Withasomnine and internal standard, S_{wit}/S_{IS}
1	21053618	Rep – 1	17.500	1.105	0.063
2	21060949	Rep – 2	18.037	1.076	0.060
3	21063100	Rep – 3	18.075	1.103	0.061
4	21065445	Rep – 4	18.324	1.115	0.061
5	21072719	Rep – 5	18.565	1.113	0.060
6	21081017	Rep – 6	19.119	1.185	0.062
Mean			18.27	1.116	0.06117
Standard deviation			0.5470	0.03650	0.001169
Standard error			0.2233	0.01490	0.0004773
Relative standard deviation (%)			2.99%	3.27%	1.91%
Confidence interval of the mean			17.70 - 18.84	1.078 - 1.154	0.05994 - 0.06239

B. Reproducibility.

The reproducibility is characterized by the mean, the standard deviation, the relative standard deviation, the standard error of the mean by injection of different preparations (n=5) of Standard Solution. (Table 2)

Table 2.

N	File name	Sample name	Area of Internal Standard , S_{IS}	Area of Withasomnine peak , Swit	Ratio of peaks areas of Withasomnine and internal standard, S_{wit}/S_{IS}
1	09053445	SS-1	14.066	7.6000	0.540
2	08203516	SS-2	13.24	7.6870	0.581
3	08215649	SS-3	13.524	8.0550	0.596
4	09030839	SS-4	12.791	8.1230	0.635
5	09074747	SS-5	12.872	7.5210	0.584
Mean			13.30	7.797	0.5872
Standard deviation			0.5203	0.2738	0.03405
Standard error			0.2327	0.1225	0.01523
Relative standard deviation (%)			3.91%	3.51%	5.80%
Confidence interval of the mean			12.65 - 13.94	7.457 - 8.137	0.5449 - 0.6295

C. Repeatability of the method

The precision of the method is established by analysis of Withania somnifera extracts (n=5), which have been prepared separately from the same sample of Ashwaganda (Withania root, SHI, h (loss of drying) = 6.8%) under the conditions described in the method (Table 3)

Table 3

N	File name	Sample name	Area of Internal Standard , S_{IS}	Area of Withasomnine peak , Swit	Ratio of peaks areas of Withasomnine and internal standard, S_{wit} / S_{IS}	Content of the Withasomnine, (mg/g) , M_{wit}
1	08183557	Sample – 1	9.234	2.630	0.284	0.0052
2	08191410	Sample – 2	9.769	2.719	0.278	0.0051
3	08195612	Sample – 3	9.716	2.599	0.267	0.0049
4	08211816	Sample – 4	9.961	2.218	0.223	0.0041
5	08223717	Sample – 5	10.229	2.241	0.219	0.0041
Mean						0.00468
Standard deviation						0.0005404
Standard error						0.0002417
Relative standard deviation (%)						11.55%
Confidence interval of the mean						0.004009 – 0.00535

Accuracy

The accuracy of the method is characterized by mean, the standard deviation, the relative standard deviation, the error of the mean by analysis of samples (n=5) obtained by addition of known amounts of the standards to the drug samples and measured under conditions described in the method (Table 4).

Table 4.

Sample name	File Name	Initial content, C (mg/ml)	Added, I (mg/ml)	Sum, S = C + I (mg/ml)	Ratio of peaks areas of Withasomnine and internal standard, S_{wit} / S_{IS}	Determined content of Withasomnine (mg/ml) $E=\frac{S_{wit} \bullet C_{IS}}{S_{IS} \bullet K_{wit}}$	Recovery, R=E x 100 / S (%)
Sample – 1	08223717	0.0205	0	0.0205	-	-	-
Sample – 2	09013335	0.0205	0.015	0.0355	0.203	0.035	98.59
Sample – 3	08175039	0.0205	0.05	0.0705	0.434	0.074	104.96
Sample – 4	08231057	0.0205	0.1	0.1205	0.720	0.123	102.07
Sample – 5	09003103	0.0205	0.3	0.3205	1.832	0.312	97.35
Mean							100.7
Standard deviation							3.449
Standard error							1.725
Relative standard deviation (%)							3.42%
Confidence interval of the mean							95.25 - 106.2

Linearity

The linearity of the method is performed by correlation between the amount ratios of Withasomnine to Internal Standard and the corresponding peak area ratios determined on chromatograms (Table 5).

Table 5

Level	File name	Amounts ratios, A_{wit} / A_{IS} **(X)**	Ratio of peaks areas, S_{wit} / S_{IS} **(Y)**
9	09101743	0.5	0.038
8	09082616	1.0	0.080
7	20133514	2.0	0.116
6	20120647	4.0	0.234
5	09090042	5.0	0.288
4	09053445	10.0	0.558
3	09060408	25.0	1.502
2	09063828	50.0	3.054
1	09071546	100.0	5.721
Variables			
Slope of the regression line			0.05777 ± 0.0007060
Y-intercept			0.02022 ± 0.02711
X-intercept			-0.3500
1/slope			17.31
95% Confidence Intervals			
Slope			0.05610 to 0.05944
Y-intercept			-0.04389 to 0.08433
Goodness of Fit			
r^2			0.9990
S y.x			0.06674
Is slope significantly non-zero?			
F			6696
DFn, DFd			1.000, 7.000
P value			< 0.0001
Deviation from zero?			Significant
Residuals			
Points above line			3
Points below line			6
Number of runs			5
P value (runs test)			0.6429
Significantly nonlinear?			Not Significant

The method has been shown to be linear in the range from 0.005 to 1.0 mg/ml. The regression analysis is shown in the table. The correlation coefficients showing a good linear fit within the range tested.

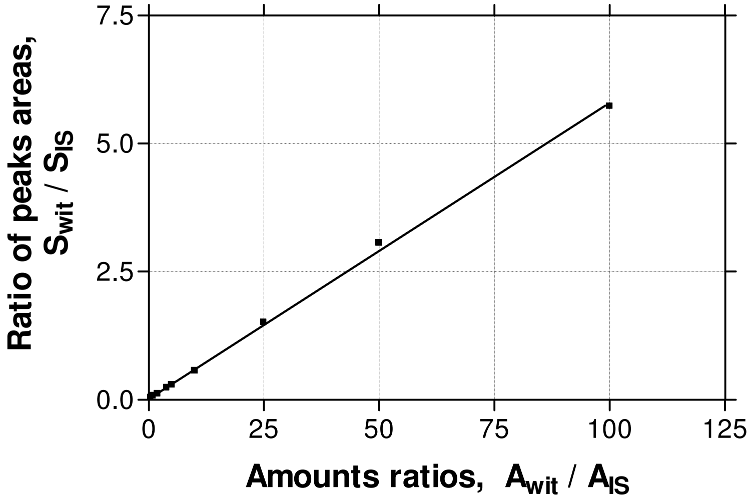

Figure 4. Linearity of the method.

Limit of Detection

Limit of detection were experimentally found (Table 6)

Table 6

Level	File name	Amount of Withasomnine, mg/ml	Ratio of areas, S_{wit} / S_{IS}
9	20114222	0.0025	0.0006
10	20111223	0.0005	-

Selectivity

The selectivity of the method is characterized by the mean, the standard deviation, the relative standard deviation, the standard error of the mean of the relative retention time of Withasomnine to Internal Standard (Table 7).

Table 7

N	File name	RT *of **Internal Standard**, min	**Withasomnine** RT(min)	**Withasomnine** RRT(min)
1	08203516	21.78	14.81	0.680
2	08215649	21.84	14.88	0.681
3	08183557	22.19	15.06	0.679
4	08195612	21.98	14.93	0.679
5	08175039	22.55	15.22	0.675
6	08211816	21.74	14.79	0.680
Mean			14.95	0.6790
Standard deviation			0.1646	0.002098
Standard error			0.06720	0.0008563
Relative standard deviation (%)			1.10%	0.31%
Confidence interval of the mean			14.78 - 15.12	0.6768 - 0.6812

* - RT – Retention Time of withasomnine, (min)
RRT-Relative Retention Time to Internal Standard = $RT_{withasomnine}$ / $RT_{Internal\ Standard}$

Isolation, purification and identification of withasomnin and fraction of Sitoindosides from *Withania somnifera* Dun. roots

A.Panossian, G.Mamikonyan, A.Danielyan

Guielbenkian Laboratory of ADMTA, Yerevan, Armenia

Sponsored by Swedish Herbal Institute , Gothenburg, Sweden

2001

Reagents and materials

A) Water, distilled
B) Acetonitrile, (Art. 7330.2), ROTH
C) Methanol,(Cat.No: 17,933-7), ALDRICH
D) Petroleum ether
E) n-Hexane, (Art. 17,933-7), ROTH
F) Diethyl ether, (Cat.No: 31670), FLUKA
G) Chloroform, (Art. 7331.1), ROTH
H) Ammonia solution
I) o-Phosphoric Acid, 85% (Art. 6366.1), ROTH
J) Silica Gel 100-200 mesh (70-140 μ) average pore diameter: 25À ,(Cat.No: S-4133), SIGMA
K) Ashwaganda (*Withania somnifera* Dun.root), Swedish Herbal Institute, Gothenburg

Extraction

Air dry and powdered roots of Withania somnifera Dun. (300,0g) were extracted with petroleum ether (3 x 300 ml) for 24 h (every extraction). Extracts were discarded, the roots were dampened with 500 ml of 10 % ammonia water solution for 15 min, and extracted three times with diethyl ether (3 x 300 ml, first extraction – overnight, second and third - in ultrasonic bath for 30 min). The combined extract (650 ml) was filtered, evaporated to dryness (0.8 g). The residue was taken up in 50 ml 1% phosphoric acid and extracted with chloroform (3x50 ml). The lower phase was separated and evaporated to dryness. The residue (0.5 g) was applied to silica-gel column (50 g, 30 x 2.5 cm).

Column chromatography and isolation of Sitoindosides and withasomnine
The column was eluted gradually with the following solvent systems:

I- hexane - 250 ml (fractions 1-23)
II- II hexane – diethyl ether, 247.5:2.5 (fractions 24-42)
III- hexane – diethyl ether, 237.5:12.5 (fractions 43-58)
IV- hexane – diethyl ether, 230:20 (fractions 59-75)
V- hexane – diethyl ether, 212.5:37.5 (fractions 76-91)
VI- diethyl ether, 400 (fractions 92-120)

Collected fractions of 10 ml were analyzed by TLC (figures 1-4,) and GC/MS (figures 5,6)
.

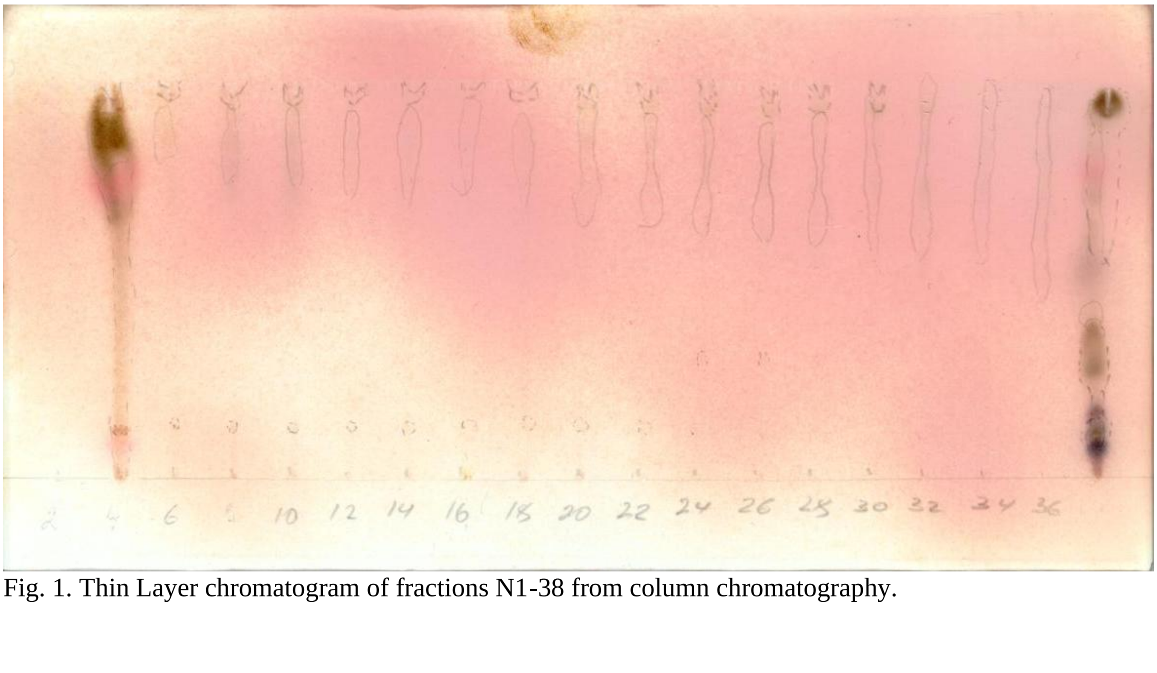

Fig. 1. Thin Layer chromatogram of fractions N1-38 from column chromatography.

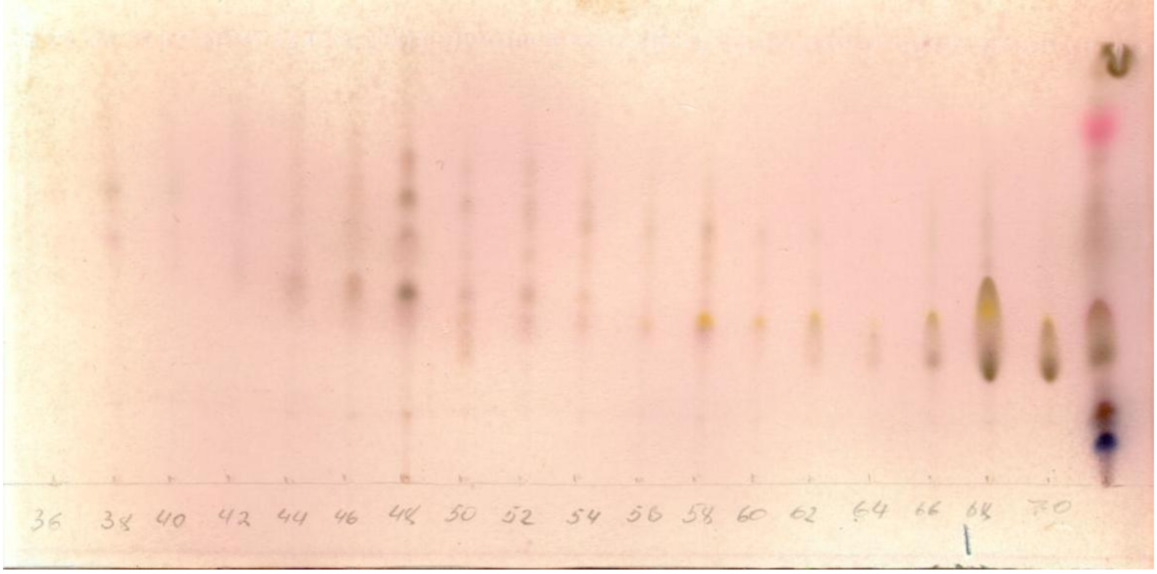

Fig. 2. Thin Layer chromatogram of fractions N40-71 from column chromatography.

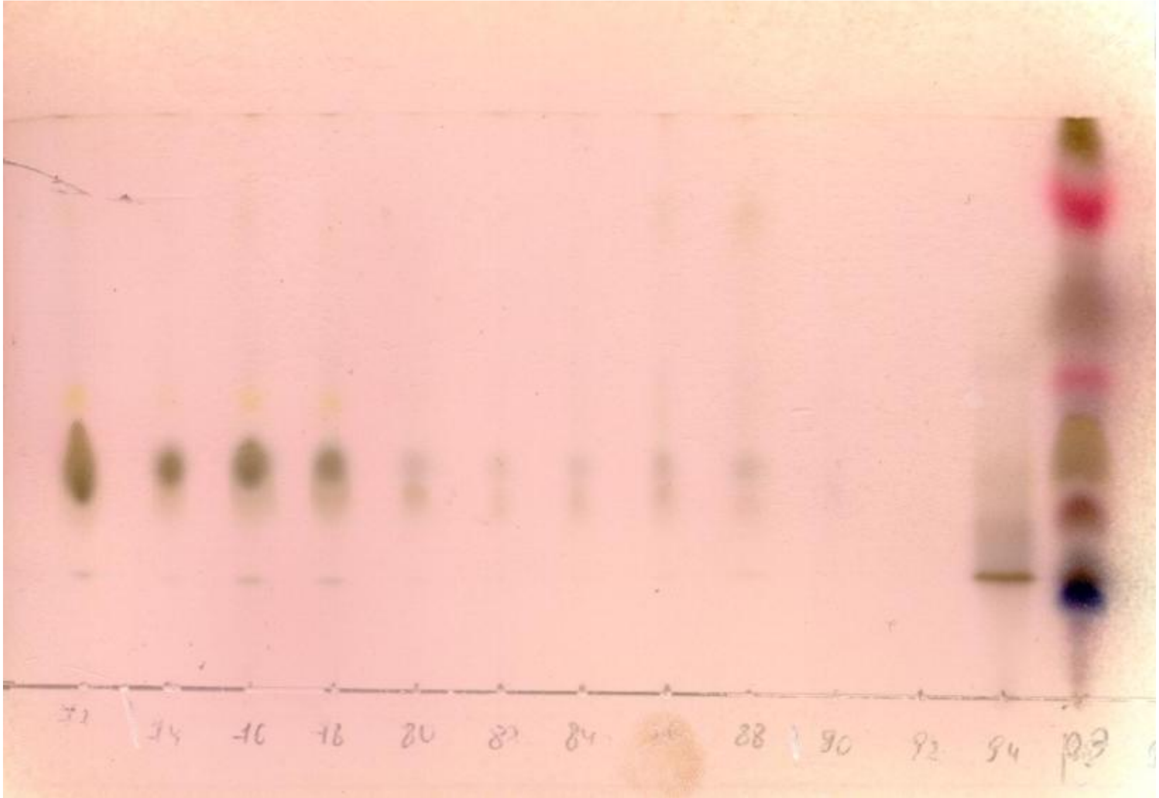

Fig. 3. Thin Layer chromatogram of fractions N71-94 from column chromatography.

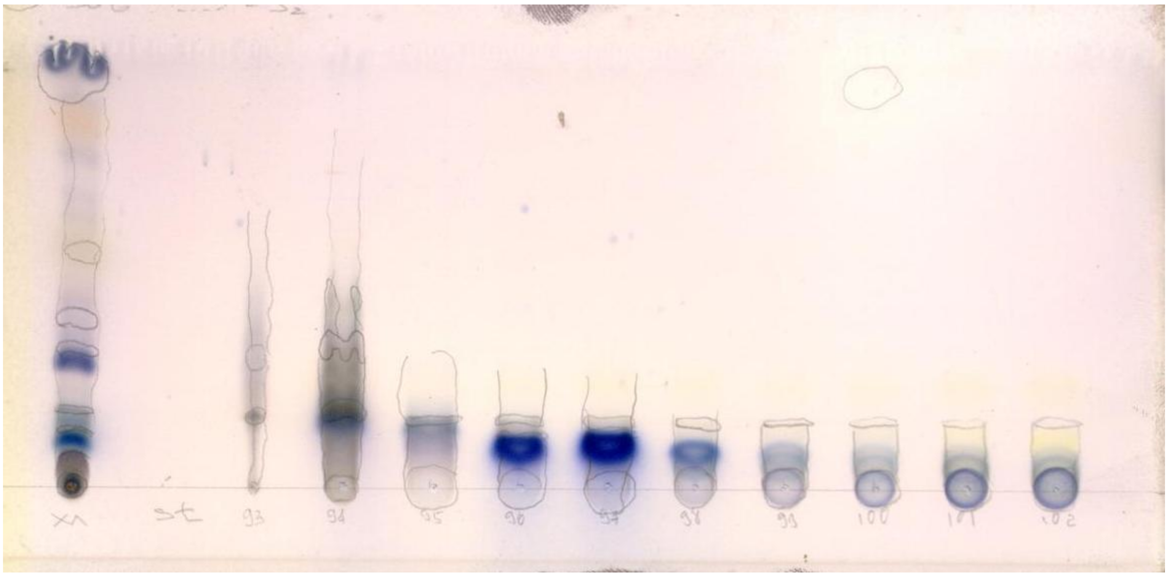

Fig. 4. Thin Layer chromatogram of fractions N93-102 from column chromatography.

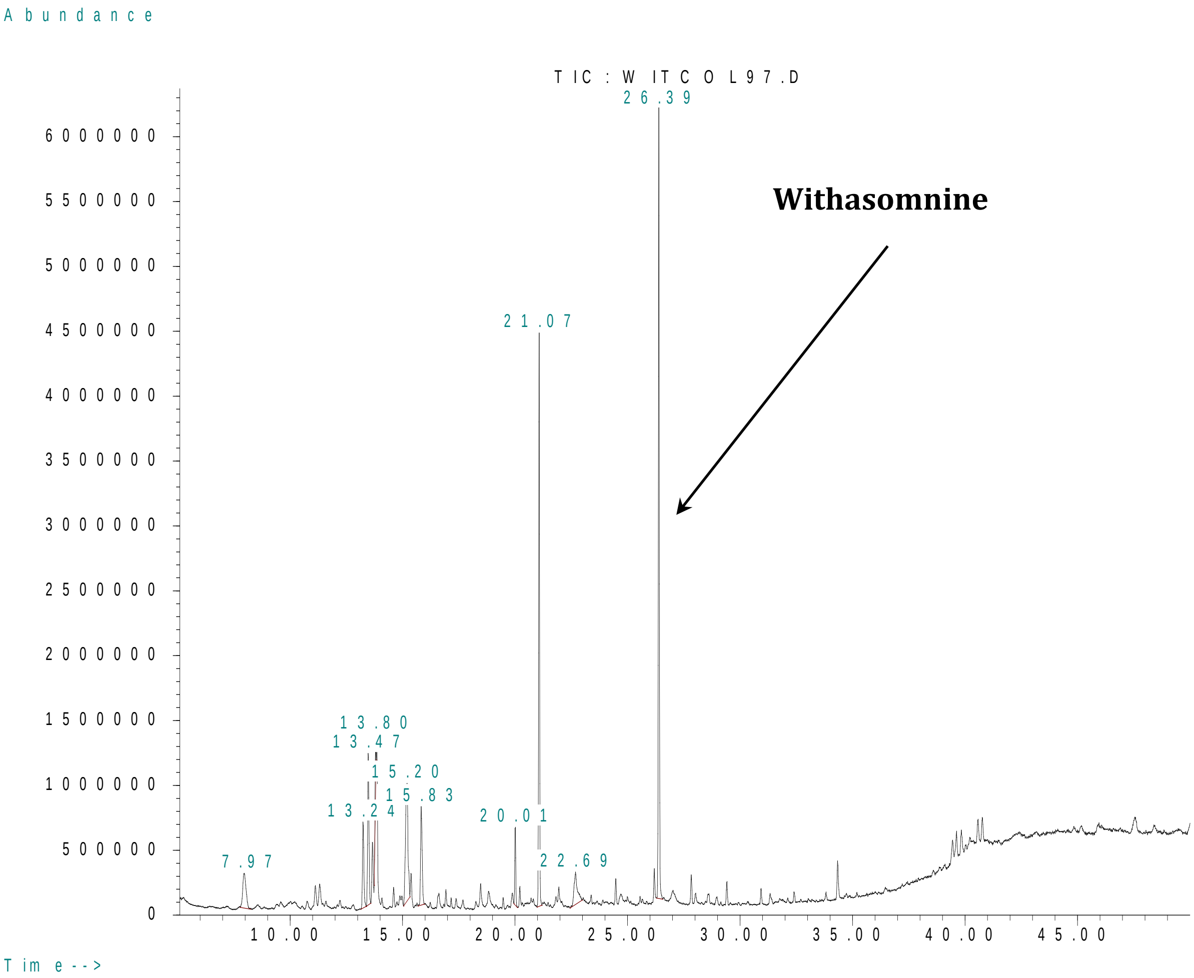

Fig. 5. Chromatogram of the fraction N97 from column chromatography in Total Ion Current Mode (R_t of Withasomnine - 29.4 min).

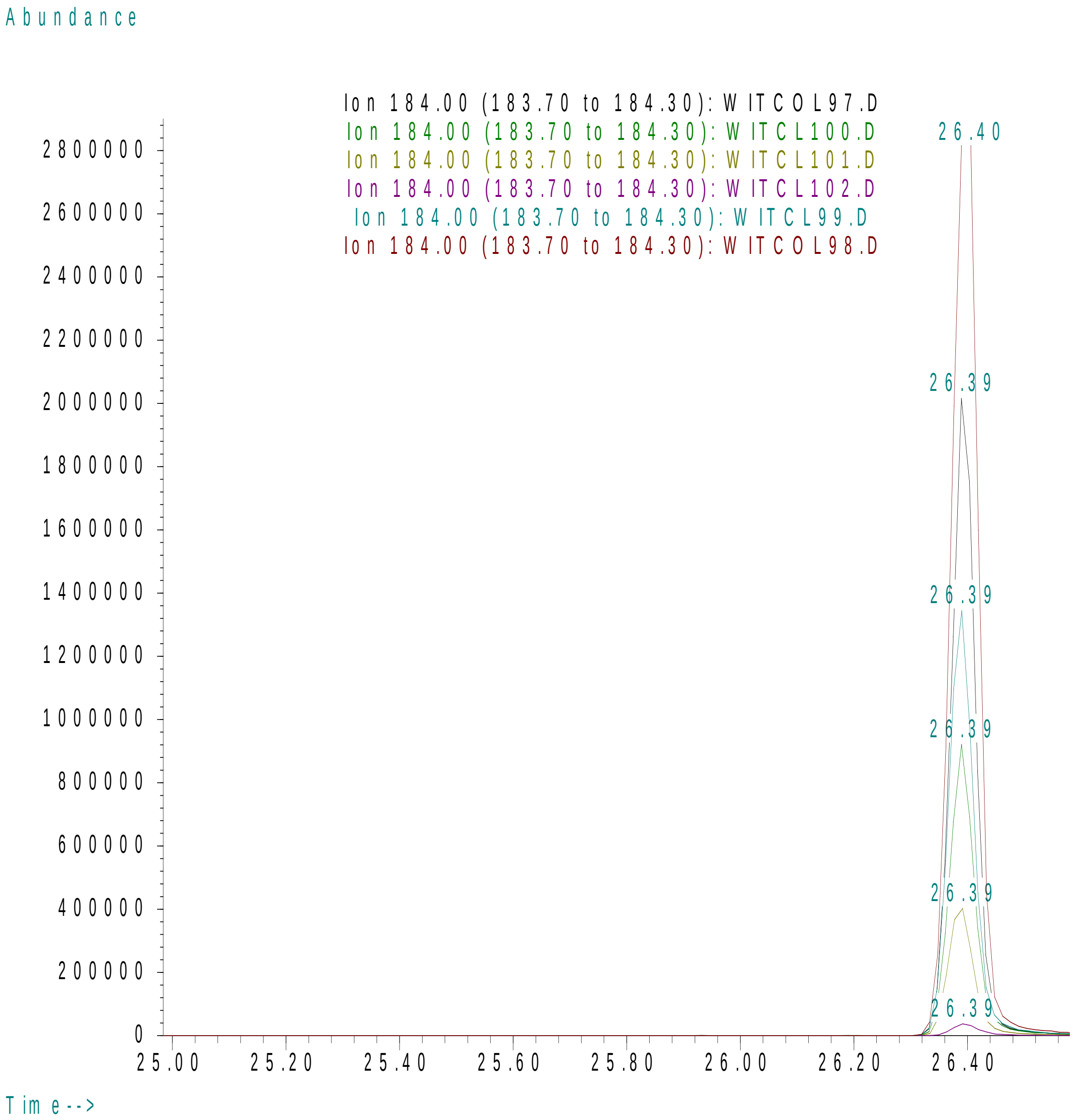

Fig. 6. Overlay of GC-MS chromatograms carried out in selected ion (m/z 184) mode of fractions NN 97-102 obtained from silicagel column chromatography (R_t of Withasomnine - 29.4 min).

Analyzed fractions were combined as indicated below, and evaporated to dryness:

68-73 - subfraction I (24.7 mg) Figure 7
74-88 - subfraction II (17.7 mg) Figure 7
97-102 – subfraction III (26.5 mg) Figure 5,6

Subtractions I and II gave fractions of sitoindosides (white crystals, Soluble in chloroform; insoluble in water m.p 117-118$^{0)}$. by repeated re-crystallization from methanol - **20.7 mg and 5.11mg**.

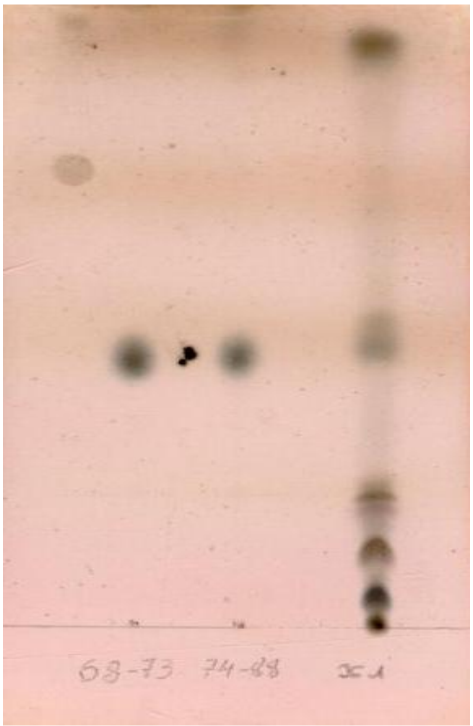

Fig. 7. Thin Layer chromatogram of subfractions I and II.

Sub-fraction III containing withasomnine was dissolved in 1 ml methanol and subjected to preparative HPLC. Withasomnine containing fractions (eluted at ~ 25 min, Fig. 8) were collected, combined and evaporated to the dryness.

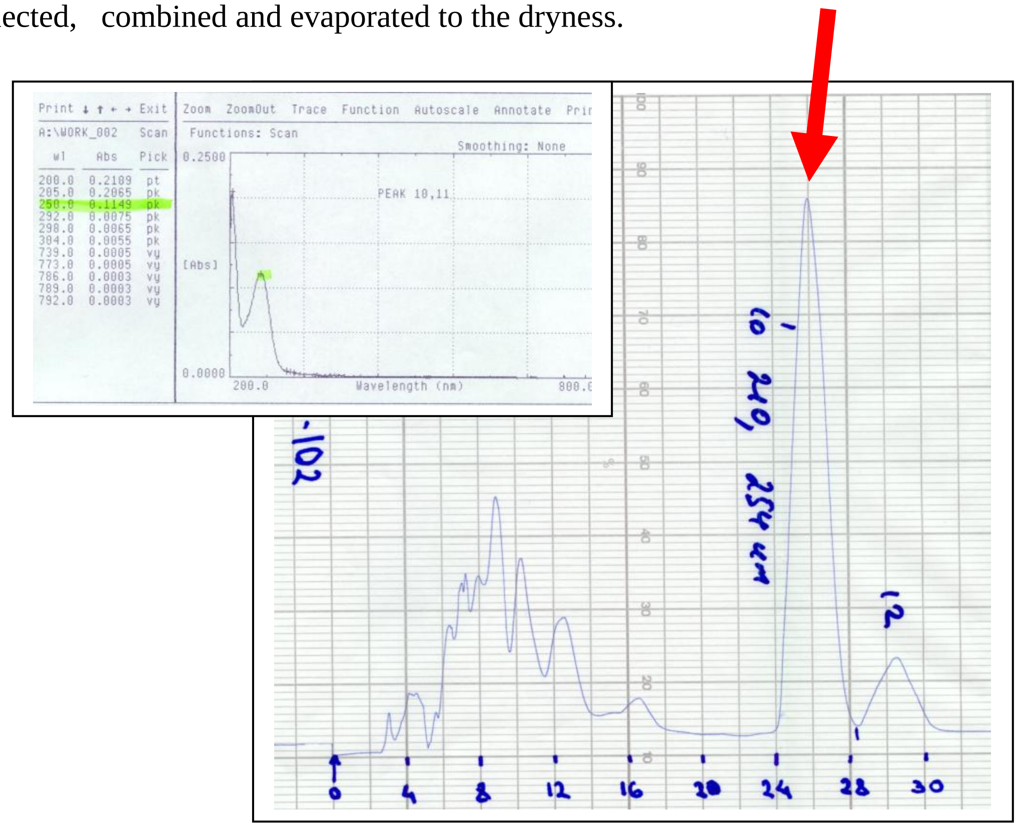

Fig. 8. Preparative HPLC of withasomnine and UV spectra of isolated withasomnine.

Totally **5.11mg** of pure withasomnine (white crystals Soluble in chloroform; insoluble in water, m.p 117-118^0) was obtained. CG-MS data are shown on figure 9,10.

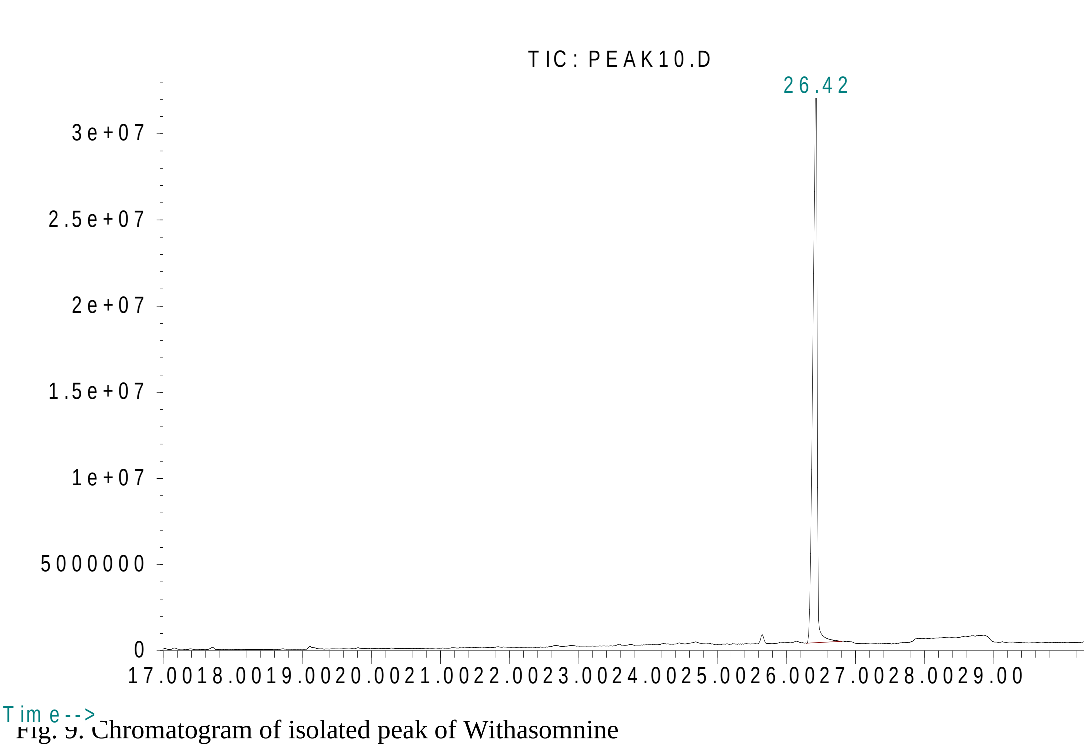

Fig. 9. Chromatogram of isolated peak of Withasomnine

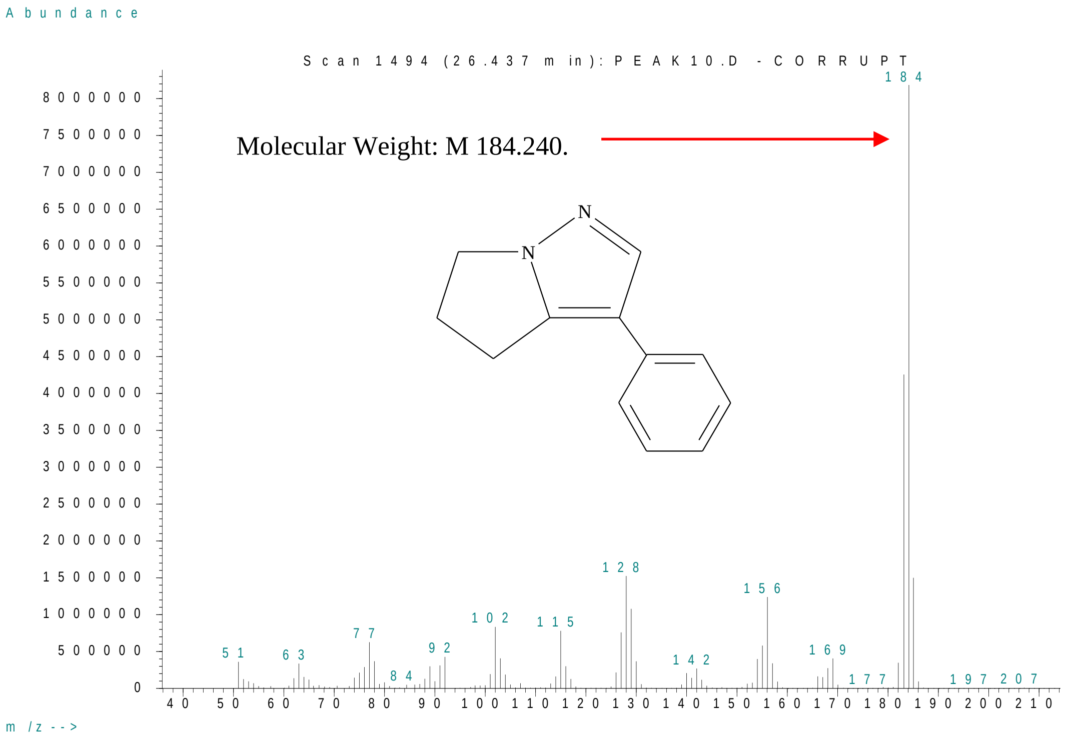

Fig. 10. Mass Spectrum of Withasomnine. 5,6-Dihydro-3-phenyl-4H-pyrrolo[1,2-b]pyrazole
Synonyms: **Withasomnine**. Newbouldine

This mass spectra is identical with described earlier for withasomnine
[Schroter, H-B, Newmann D., Katritzky A.R., Swinbourne F.J., Withasomnine. A pyrasole alkaloid from Withania somnifera Dun. *Tetrahedron*,1966, 22, 2895 –7]

TLC Conditions

TLC Plate	0.25 mm Silica gel 60, Durasil – 25, 10x20 cm (Macherey-Nagel)
Mobile phase	*Toluene /Ethyl acetate / Glacial acetic acid = 80:17.5:2.5 parts by volume*
Spotting	*100 µl*
Migration path	*8 cm*
Detection*:*	*UV/VIS – 254, 366nm*<br>*Iodine vapors*<br>*Komarovsky reagent (1 ml of 50% ethanolic sulfuric acid and 10 ml of 2% methanolic para-benzaldehyde are mixed shortly before use, heat at 100°C for 3-5 min.)*

Preparative HPLC conditions

Column	*LiChroCART 250x10 mm HPLC cartridge with LiChrospher100 RP-18 (5µm) (Merck, Darmstadt)*
Mobile phase	*Water – Acetonitrile =50:50 (v/v)*
Flow rate	3*.0ml/min*
Detection	*UV = 210 nm*
Temperature	*Ambient (about 25^{0}C)*
Injection volume	*200µl*

Analytic HPLC conditions

Column	*LiChroCART 250x4 mm HPLC cartridge with LiChrospher100 RP-18 (5µm) (Merck, Darmstadt)*
Mobile phase	*Water – Acetonitrile =50:50 (v/v)*
Flow rate	1*.0ml/min*
Detection	*UV = 210 nm*
Temperature	*Ambient (about 25^{0}C)*
Injection volume	*20µl*

GC/MS conditions

Column	*HP-50+ 50% Phenyl Methyl Siloxane, 30.0m x 0.25mm x 0.25um film*
Carrier Gas	*Helium*
Flow	*1.0 ml/min*
Oven	100^{0}*C, hold 5.0 min; to 275^{0}C at 5^{0}C/min, hold 10 min*
Injection	*Pulsed Splitless, 1µl*
Interface	*230^{0}C*
Detector	*Electron impact ionization, 70 eV, Total Ion Current mode,*<br>*Detector Temperature of 285^{0}C*

Maximum sensitivity autotune +100

Identification of Sitoindosides was performed by GC-MS analysis of their acetates, TMS ethers, free sterols, as well as their acidic hydrolysis products (Figures 11-16).

Results of analysis indicates that Sitoindosides fraction comprises of free sterols subtraction (mixture of   , in ratio), and their acylated glycosides. Sugar moiety consists of glucose and galactose, Fatty acid residue – palmitic, stearic, linolic acids.

In conclusion: revision of chemical composition of Sitoindosides fraction isolated from *Withania somnifera* Dun indicates that it comprises of mixture of free sterols subtraction (mixture of β-sitosterol – stigmasterol – ergost-5-en-3β-ol, 1.2:1:0.9), and their acylated glycosides.

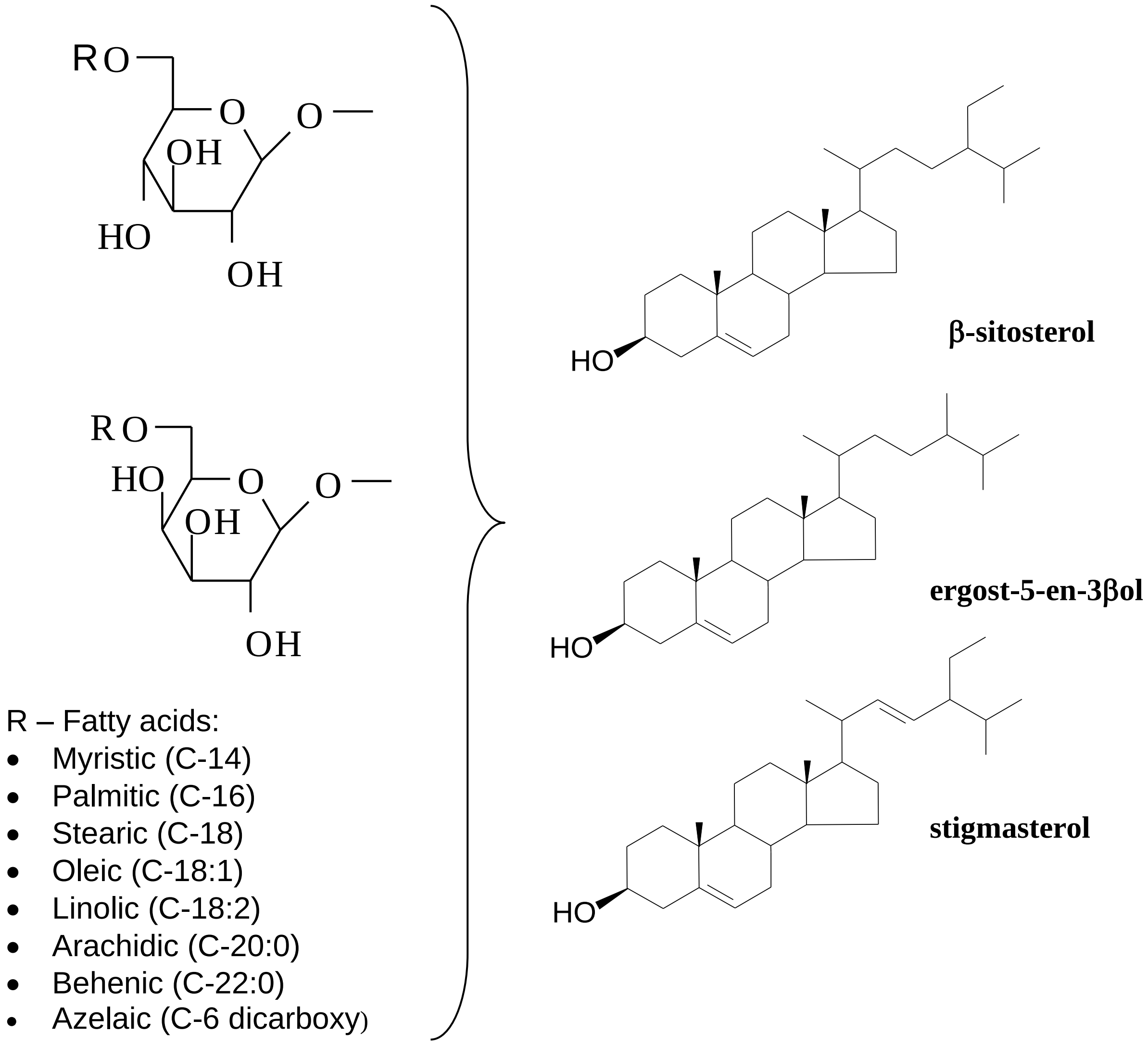

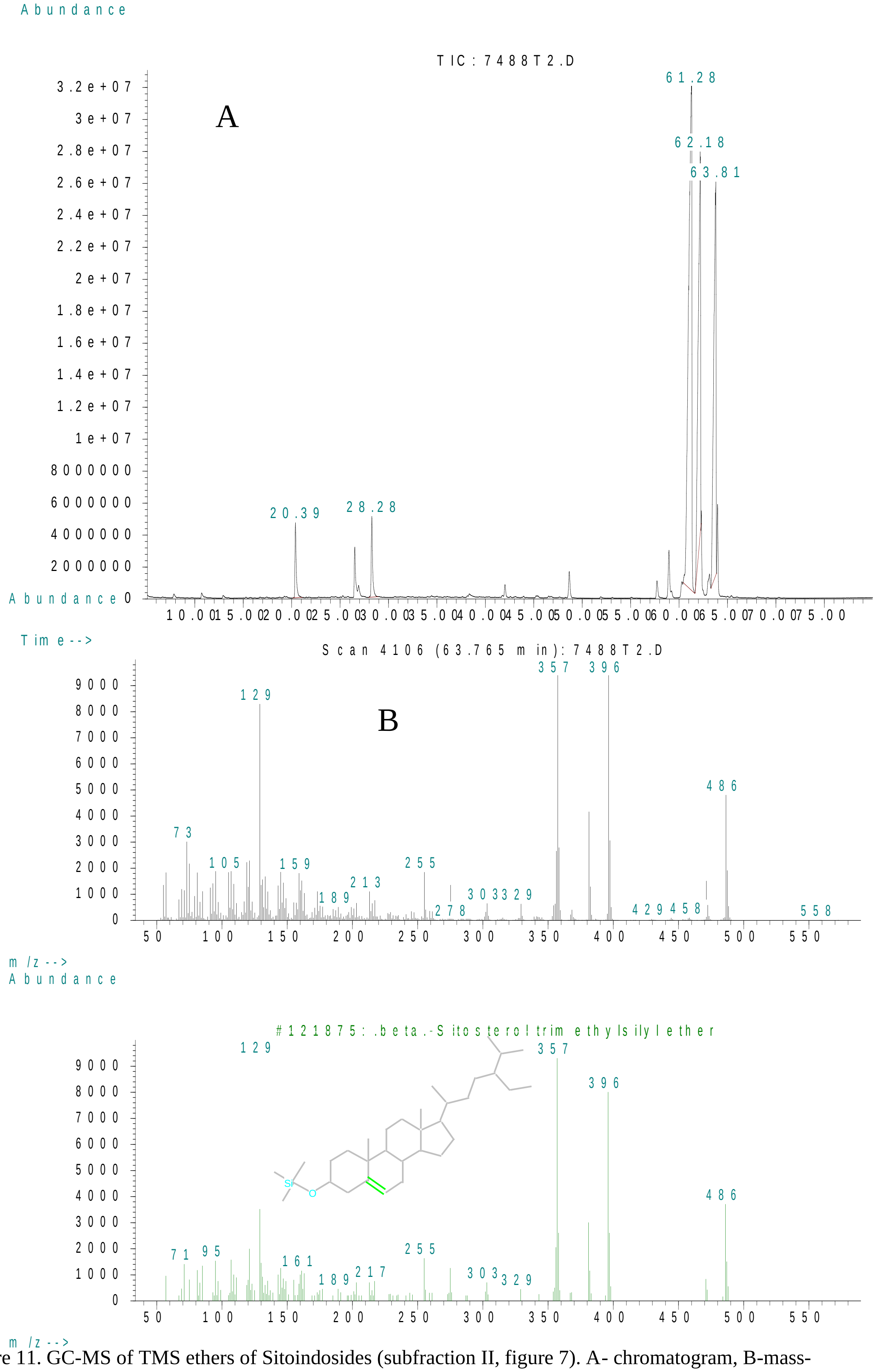

Figure 11. GC-MS of TMS ethers of Sitoindosides (subfraction II, figure 7). A- chromatogram, B-mass-spectra of peak with Rt 61.28 – identical with mass mass-spectra of 24R-Ergost-5-en-3β-ol-TMS ether (NIST – database), C - mass-spectra of peak with Rt 62.18 – identical with mass mass-spectra of Stigmasterol TMS ether (NIST – database), D-mass-spectra of peak with Rt 63.81 – identical with mass mass-spectra of β-Sitosterol TMS ether (NIST – database).

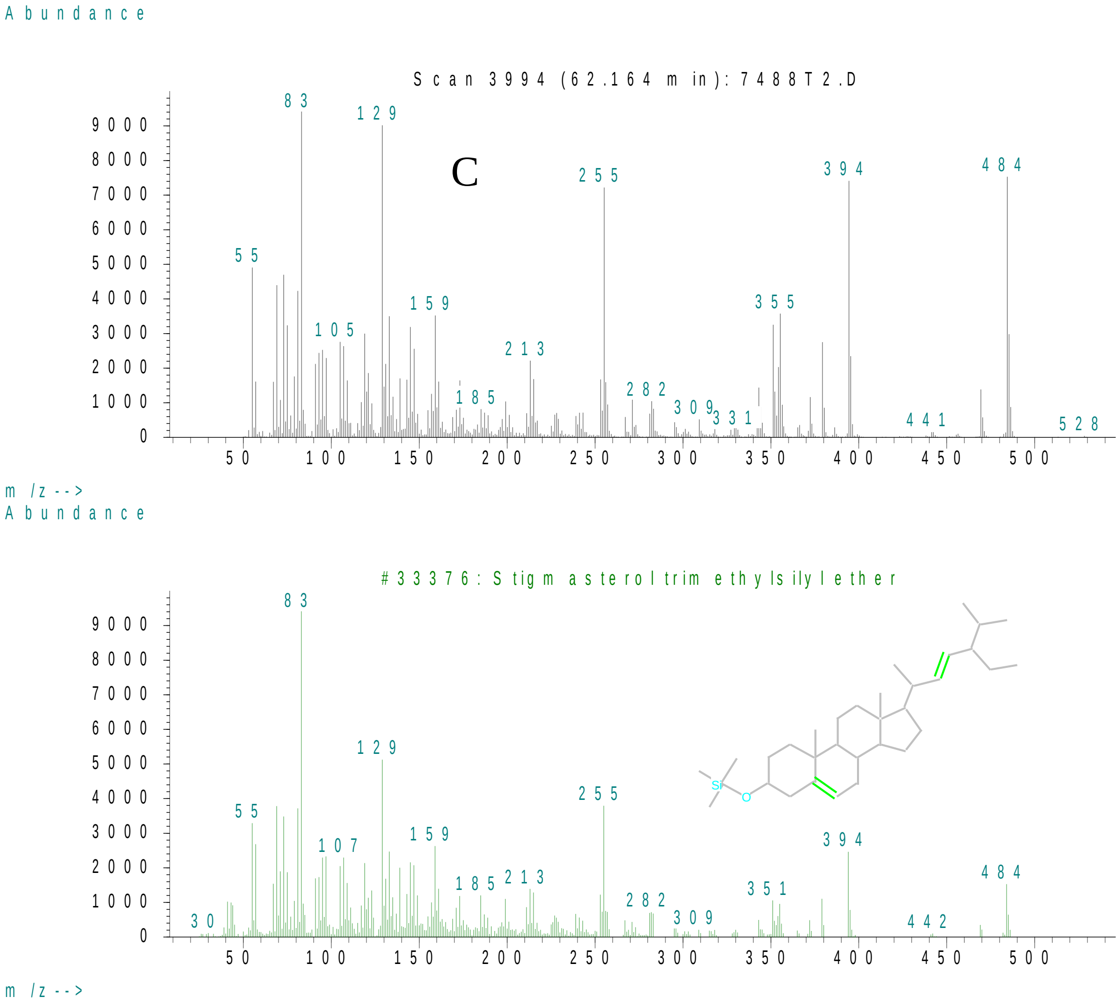
Abundance
Scan 3994 (62.164 min): 7488T2.D
C
m/z-->
Abundance
#33376: Stigmasterol trimethylsilyl ether
Si
O
m/z-->

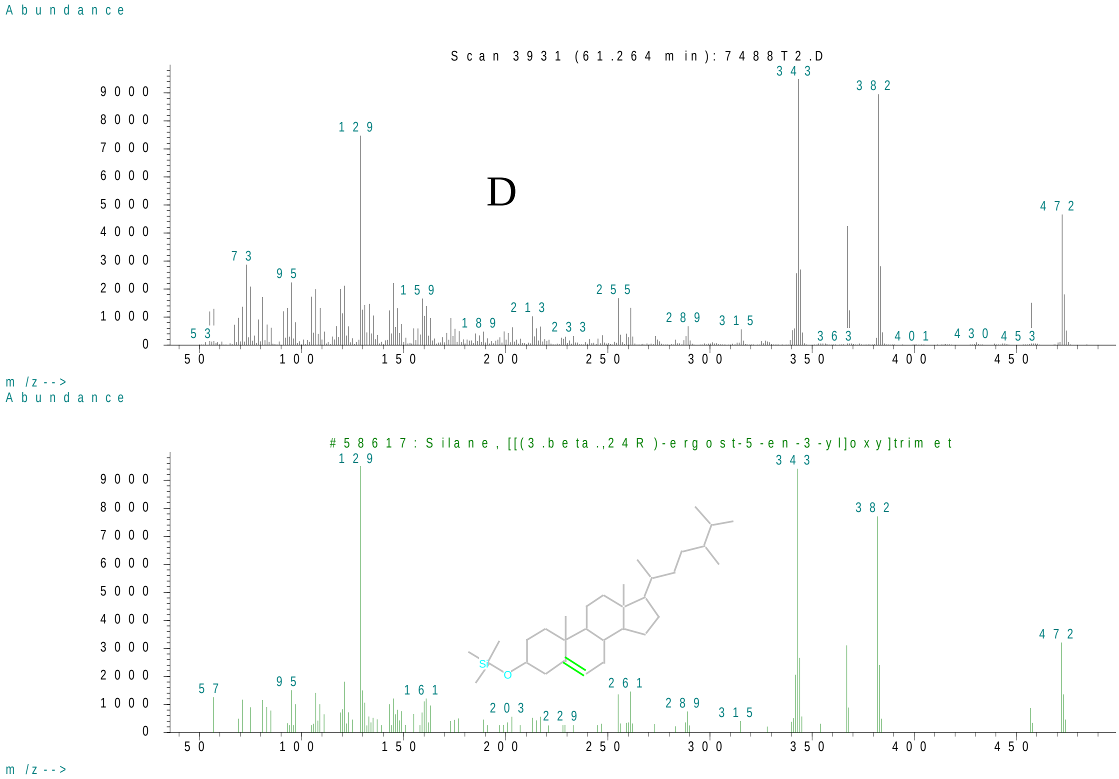
Abundance
Scan 3931 (61.264 min): 7488T2.D
D
m/z-->
Abundance
#58617: Silane, [[(3.beta.,24R)-ergost-5-en-3-yl]oxy]trimet
Si
O
m/z-->

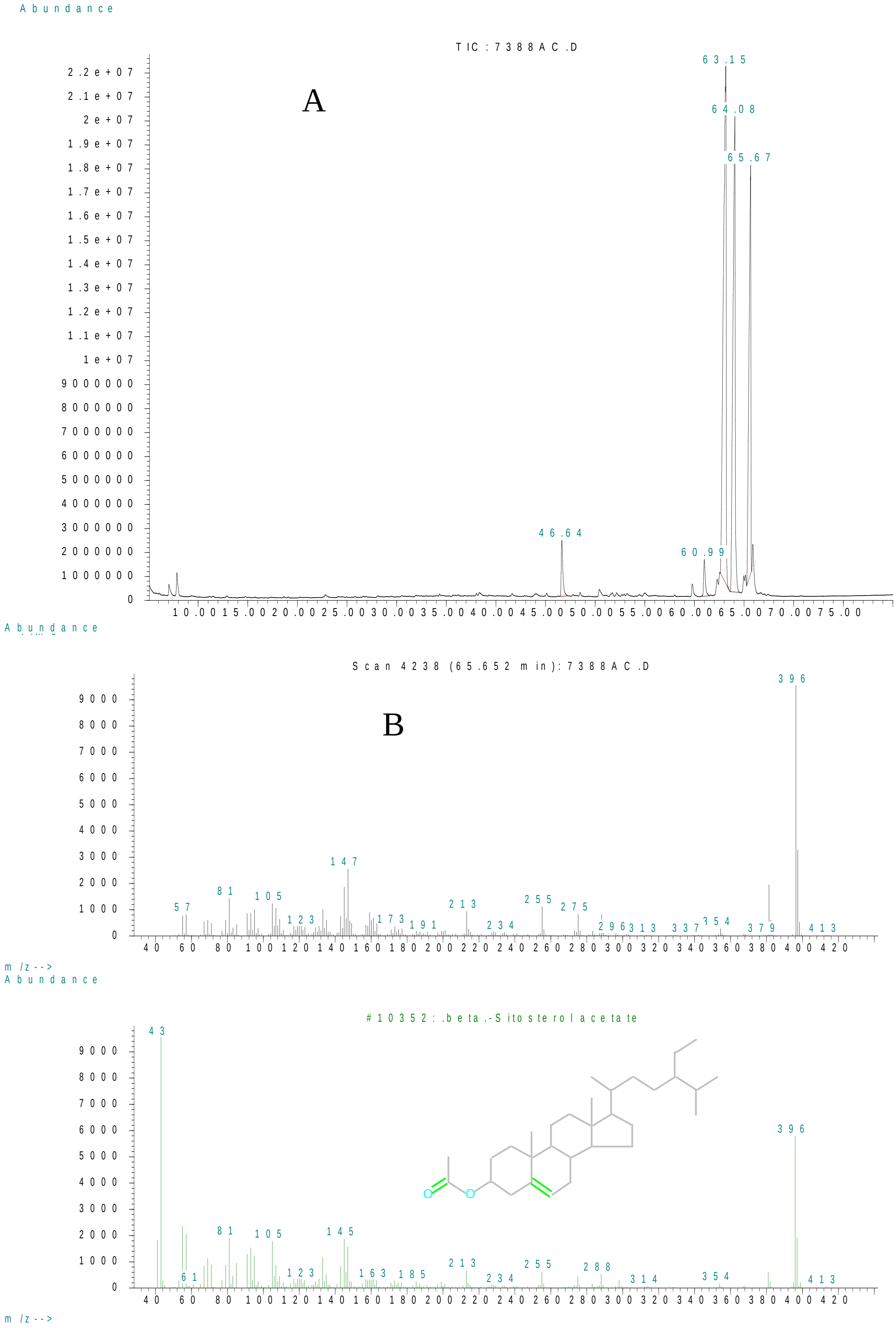

Figure 12. GC-MS of acetates of Sitoindosides (subfraction II, figure 7). A- chromatogram, B-mass-spectra of peak with Rt 61.28 – identical with mass mass-spectra of 24R-Ergost-5-en-3β-ol-acetate (NIST – database), C - mass-spectra of peak with Rt 62.18 – identical with mass mass-spectra of Stigmasterol acetate (NIST – database), D-mass-spectra of peak with Rt 63.81 – identical with mass mass-spectra of β- Sitosterol acetate (NIST – database).

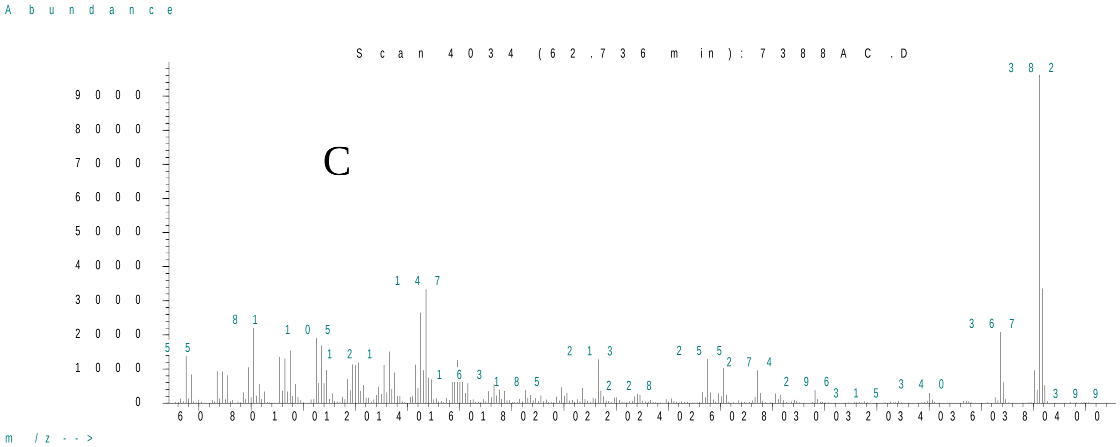
Abundance
Scan 4034 (62.736 min): 7388AC.D
C
55
81
105
121
147
163
185
213
228
255
274
296
315
340
367
382
399
m/z-->

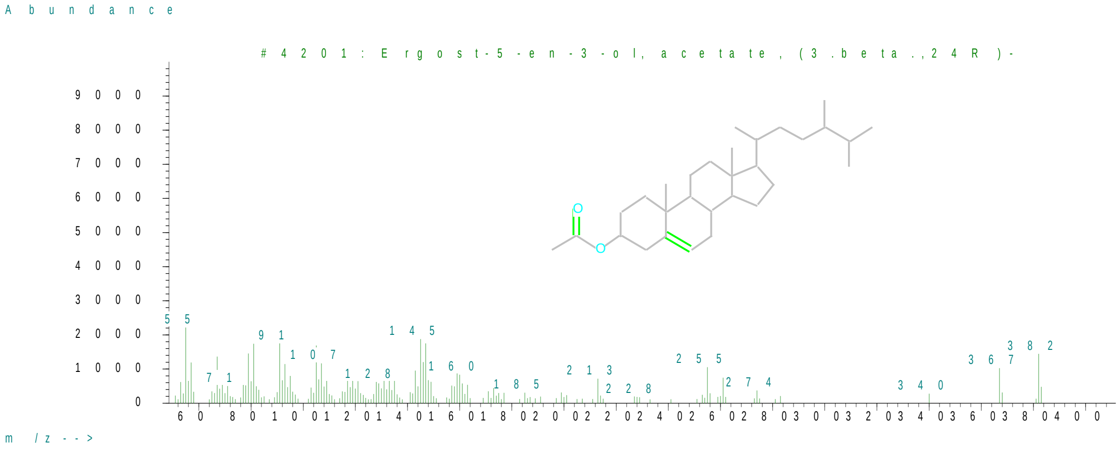
Abundance
#4201: Ergost-5-en-3-ol, acetate, (3.beta.,24R)-
55
71
91
107
128
145
160
185
213
228
255
274
340
367
382
m/z-->

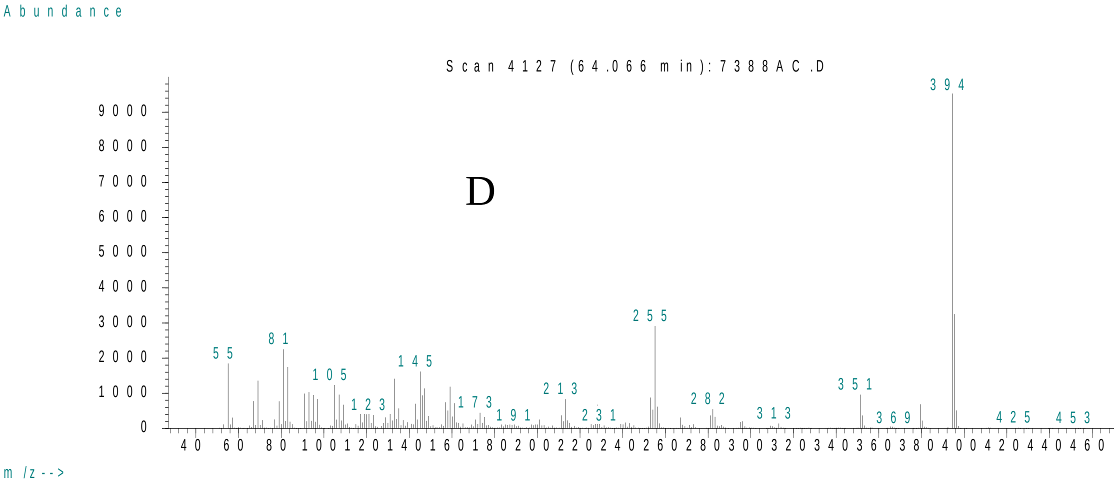
Abundance
Scan 4127 (64.066 min): 7388AC.D
D
55
81
105
123
145
173
191
213
231
255
282
313
351
369
394
425
453
m/z-->

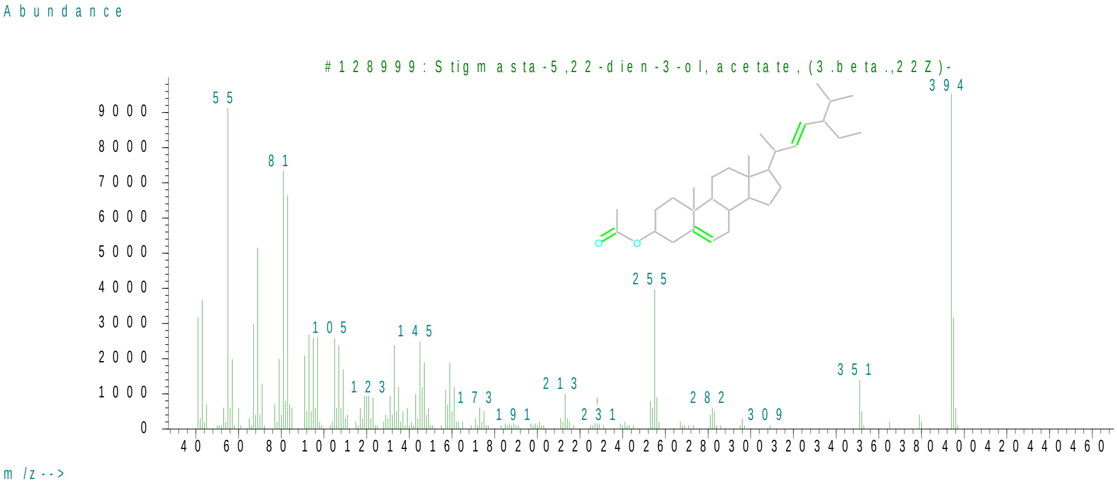
Abundance
#128999: Stigmasta-5,22-dien-3-ol, acetate, (3.beta.,22Z)-
55
81
105
123
145
173
191
213
231
255
282
309
351
394
m/z-->

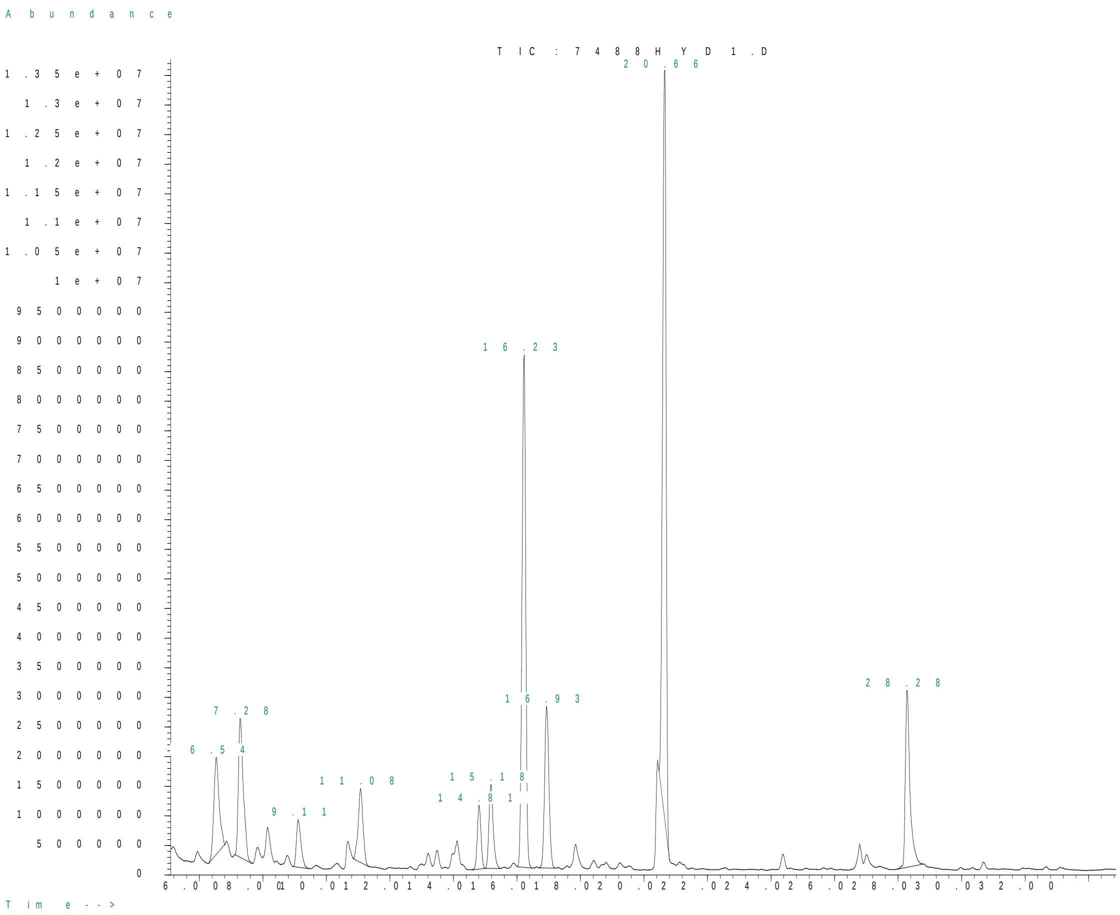

Figure 13. TMS ethers of water soluble products of acidic hydrolysis products of Sitoindosides (Subfraction II, figure 6)

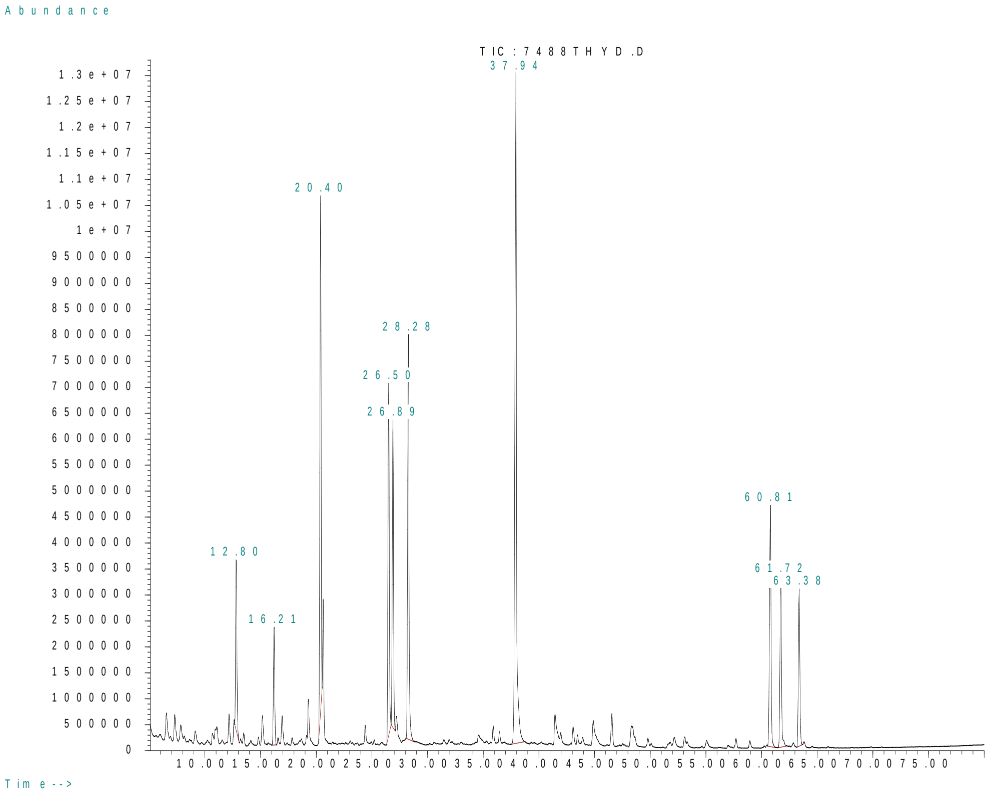

Figure 14. TMS ethers of chloroform soluble products of acidic hydrolysis products of Sitoindosides (Subfraction II, figure 6)

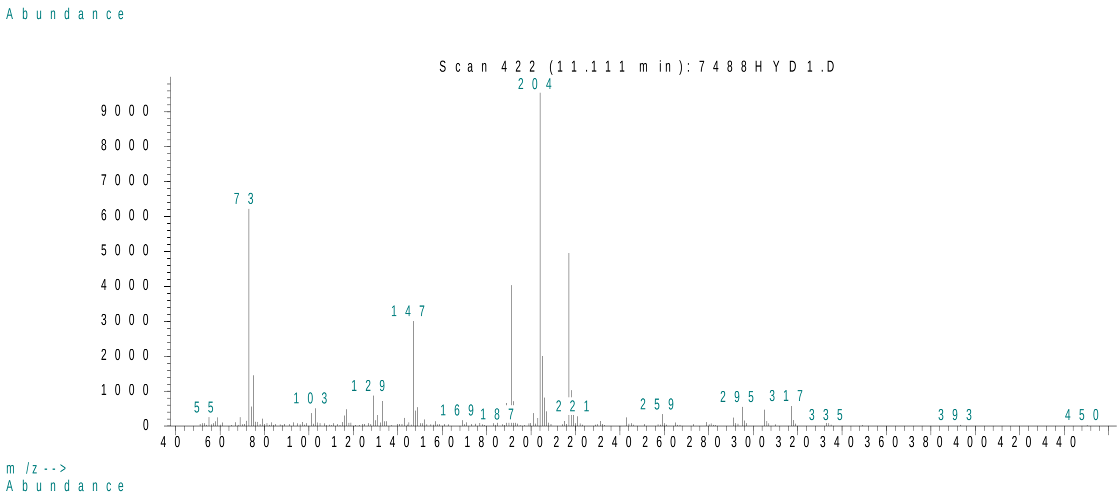
Abundance
Scan 422 (11.111 min): 7488HYD1.D
204
73
147
55
103
129
169
187
221
259
295
317
335
393
450
m/z-->

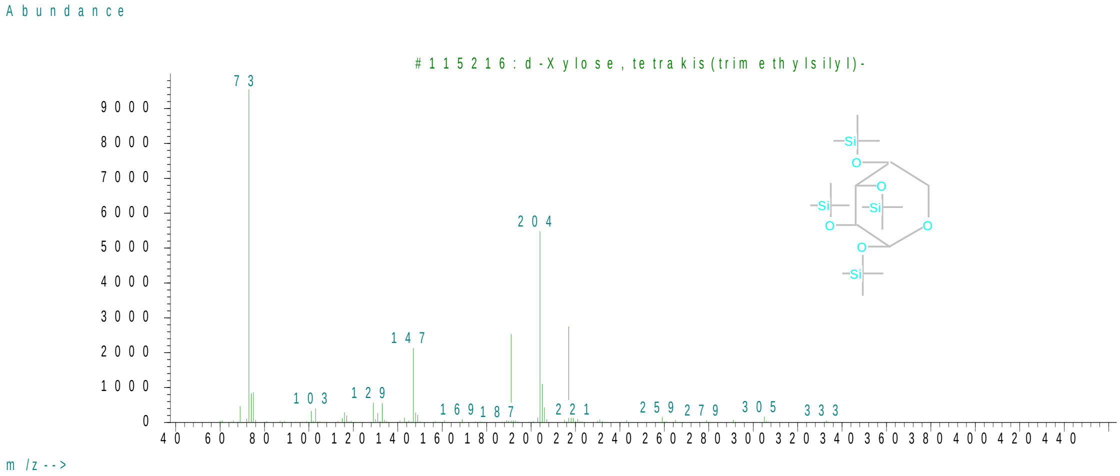
Abundance
#115216: d-Xylose, tetrakis(trimethylsilyl)-
73
204
147
103
129
169
187
221
259
279
305
333
m/z-->

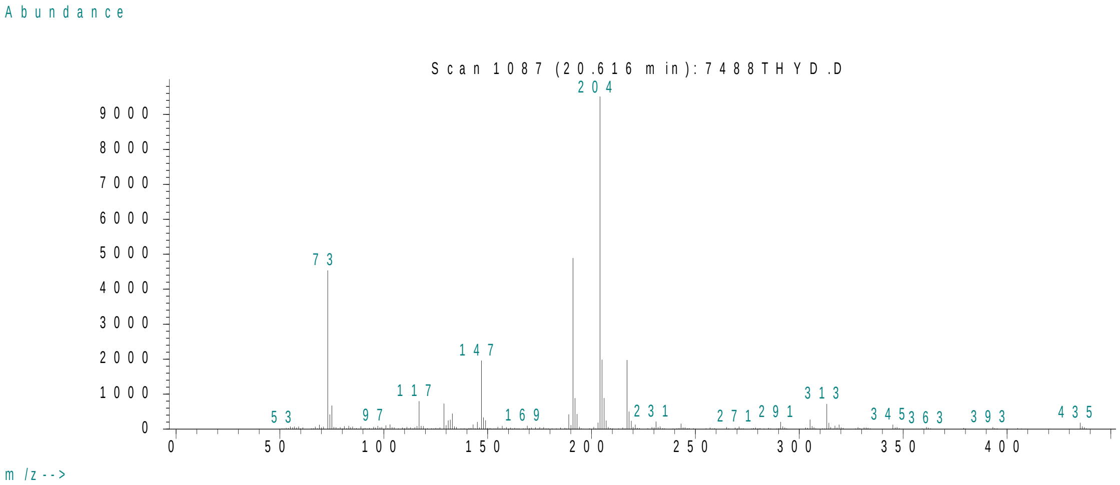
Abundance
Scan 1087 (20.616 min): 7488THYD.D
204
73
147
117
53
97
169
231
271
291
313
345
363
393
435
m/z-->

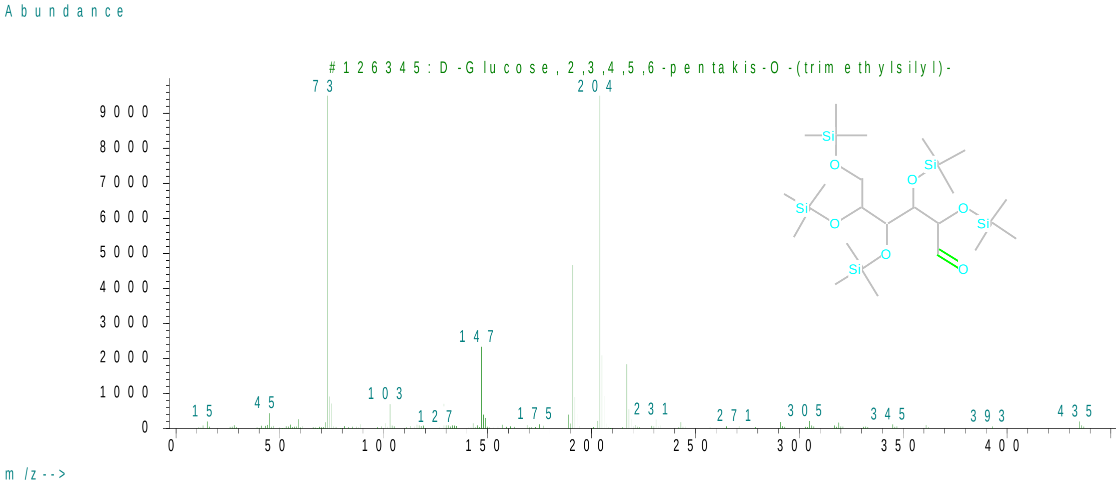
Abundance
#126345: D-Glucose, 2,3,4,5,6-pentakis-O-(trimethylsilyl)-
73
204
147
15
45
103
127
175
231
271
305
345
393
435
m/z-->

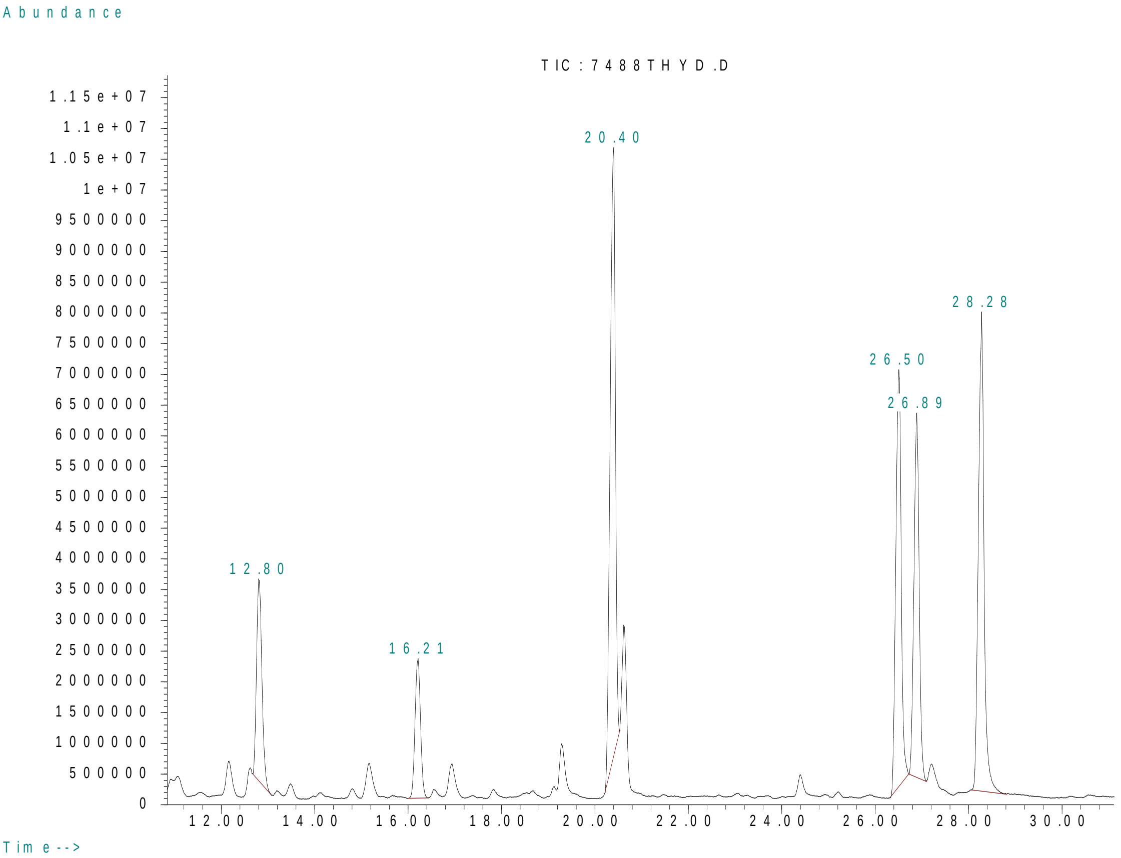
Abundance
TIC: 7488THYD.D
12.80
16.21
20.40
26.50
26.89
28.28
Time-->

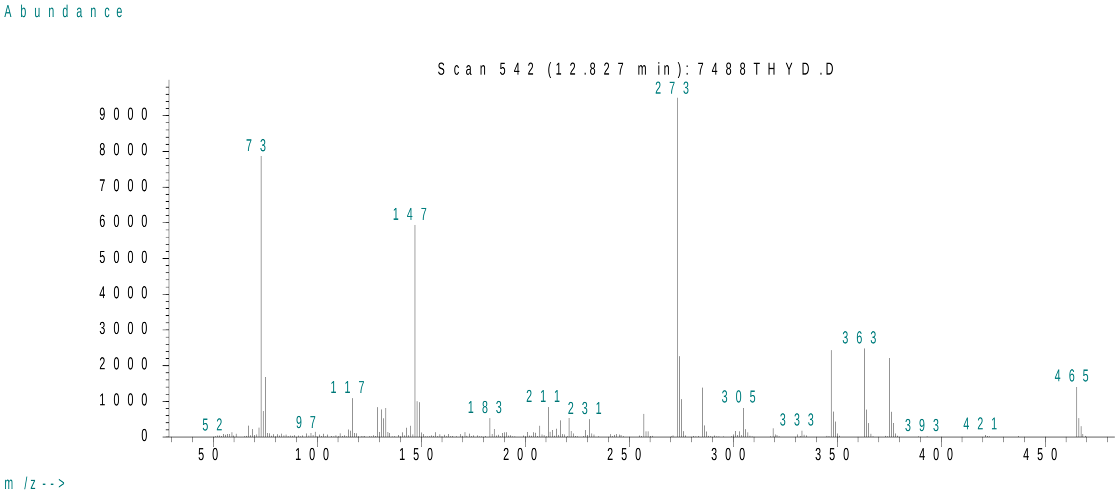
Abundance
Scan 542 (12.827 min): 7488THYD.D
52
73
97
117
147
183
211
231
273
305
333
363
393
421
465
m/z-->

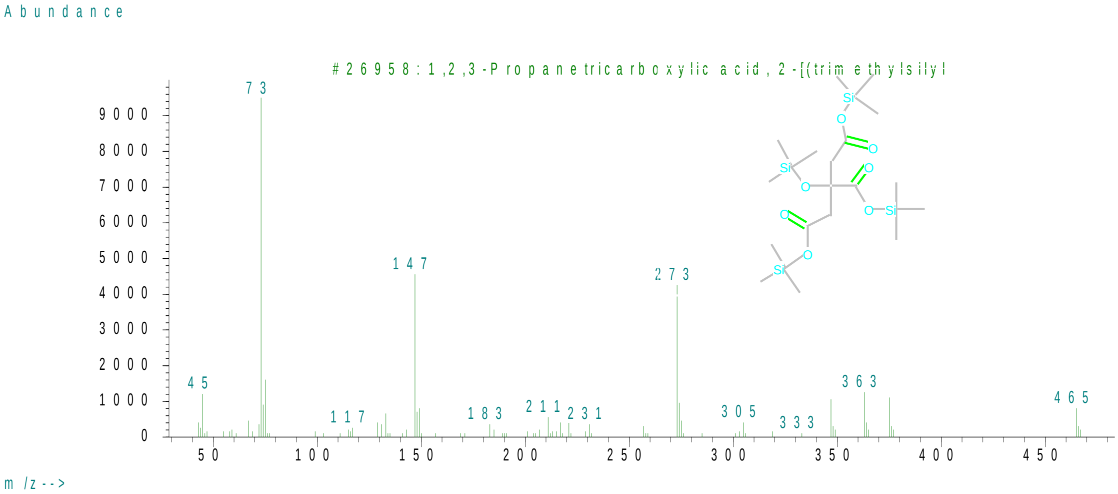
Abundance
#26958: 1,2,3-Propanetricarboxylic acid, 2-[(trimethylsilyl
45
73
117
147
183
211
231
273
305
333
363
465
m/z-->

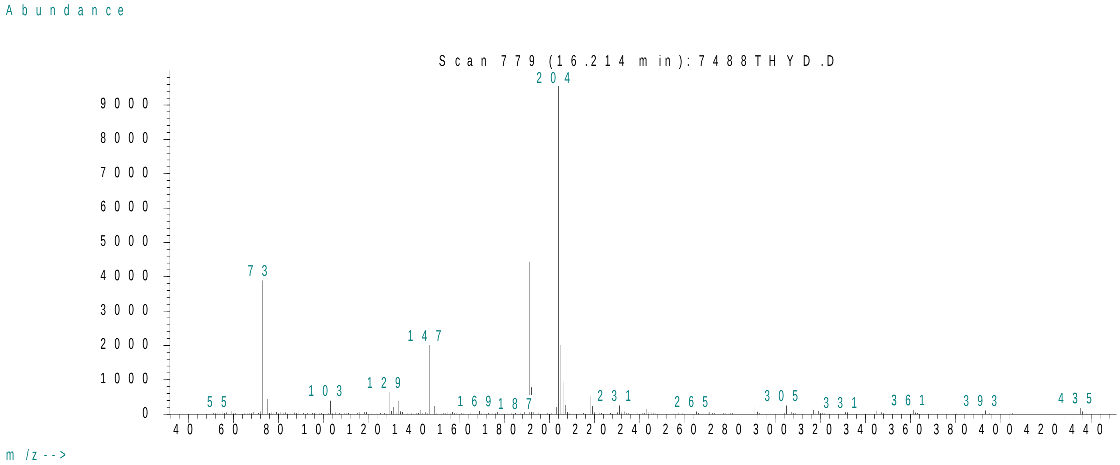
Abundance
Scan 779 (16.214 min): 7488THYD.D
204
73
147
55
103
129
169
187
231
265
305
331
361
393
435
9000
8000
7000
6000
5000
4000
3000
2000
1000
0
40 60 80 100 120 140 160 180 200 220 240 260 280 300 320 340 360 380 400 420 440
m/z-->

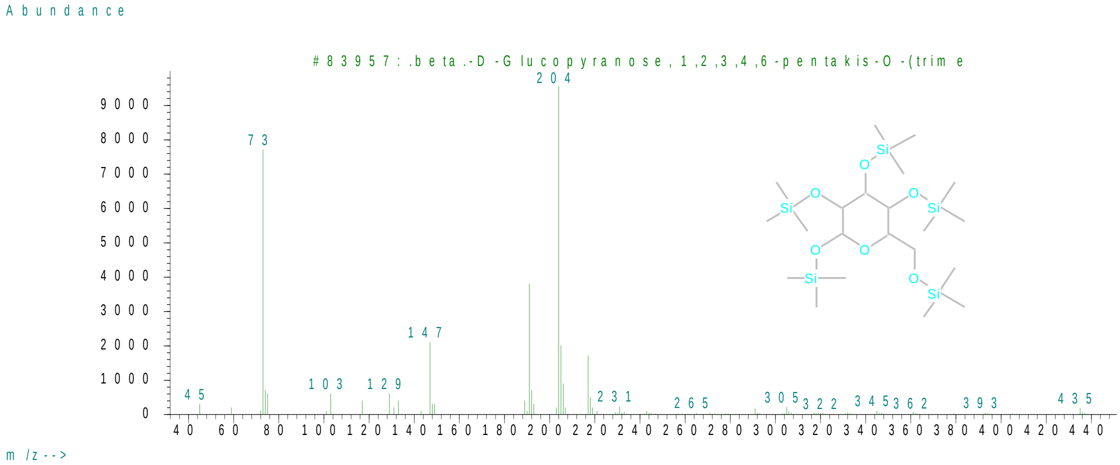
Abundance
#83957: .beta.-D-Glucopyranose, 1,2,3,4,6-pentakis-O-(trime
204
73
147
45
103
129
231
265
305
322
345
362
393
435
9000
8000
7000
6000
5000
4000
3000
2000
1000
0
40 60 80 100 120 140 160 180 200 220 240 260 280 300 320 340 360 380 400 420 440
m/z-->
Si
O
Si
O
O
Si
O
O
Si
O
Si

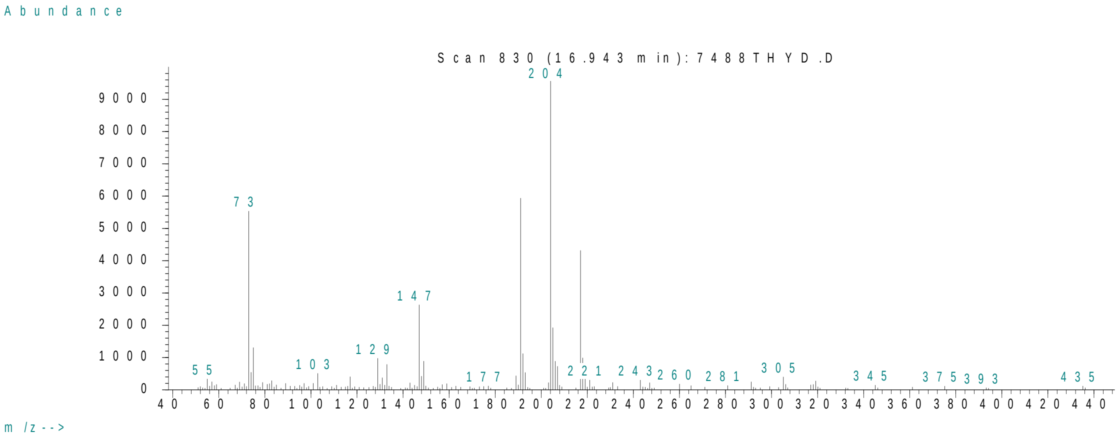
Abundance
Scan 830 (16.943 min): 7488THYD.D
204
73
147
55
103
129
177
221
243
260
281
305
345
375
393
435
9000
8000
7000
6000
5000
4000
3000
2000
1000
0
40 60 80 100 120 140 160 180 200 220 240 260 280 300 320 340 360 380 400 420 440
m/z-->

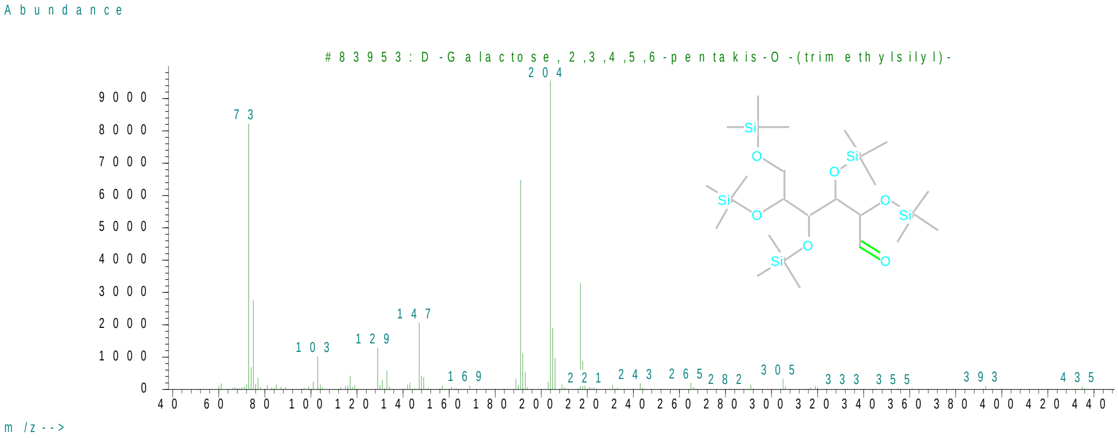
Abundance
#83953: D-Galactose, 2,3,4,5,6-pentakis-O-(trimethylsilyl)-
204
73
147
103
129
169
221
243
265
282
305
333
355
393
435
9000
8000
7000
6000
5000
4000
3000
2000
1000
0
40 60 80 100 120 140 160 180 200 220 240 260 280 300 320 340 360 380 400 420 440
m/z-->
Si
O
O
Si
Si
O
Si
O
O
Si
O
Si

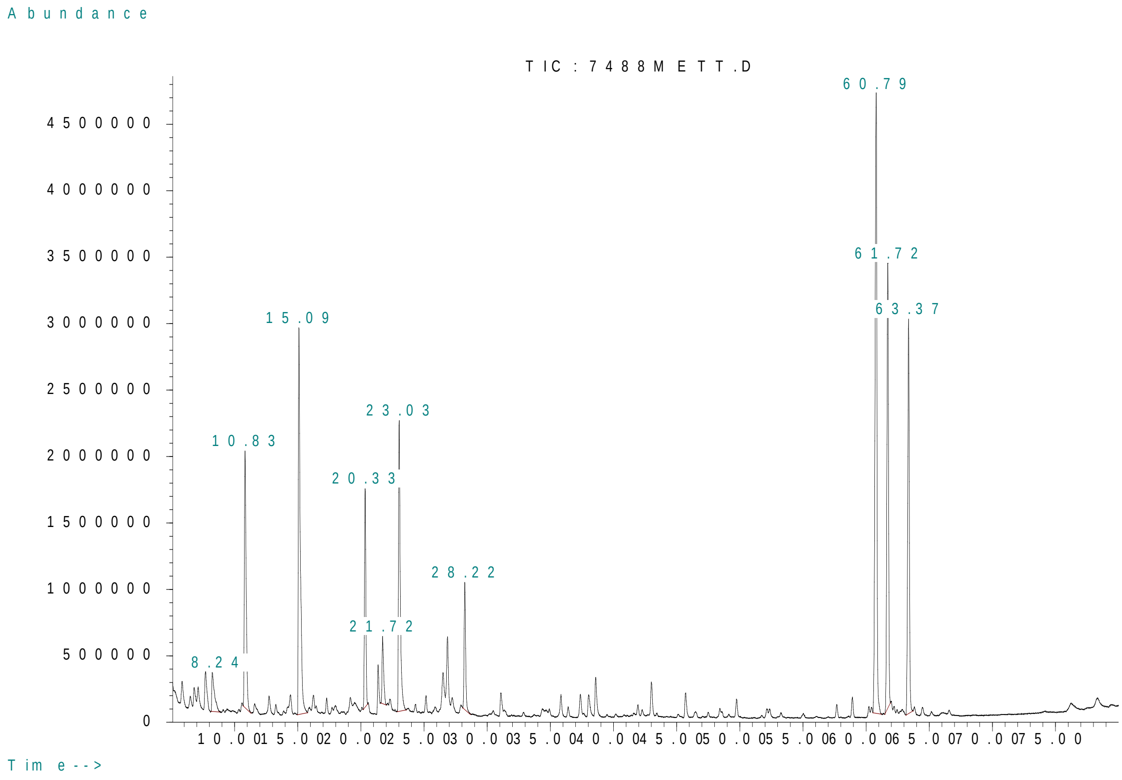

Figure 15. TMS ethers of acidic methanolysis products of Sitoindosides (Subfraction II, figure 6)

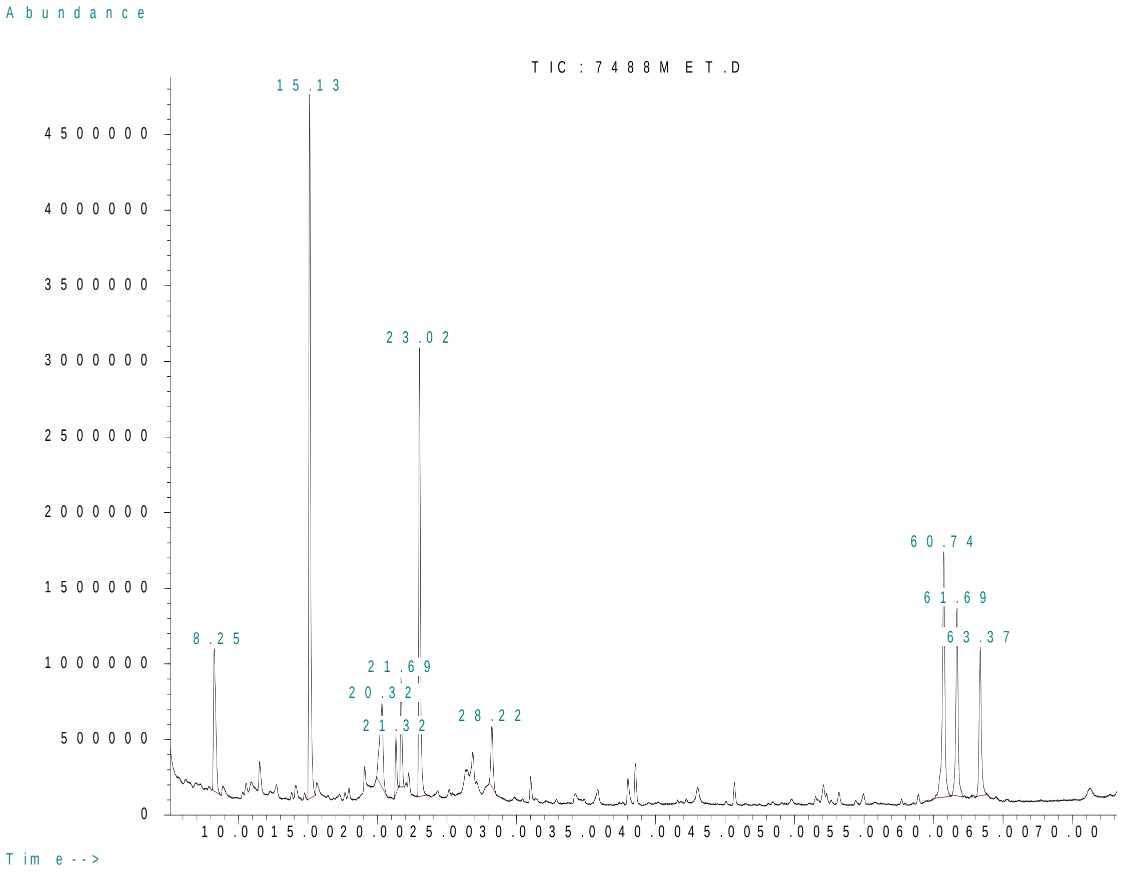

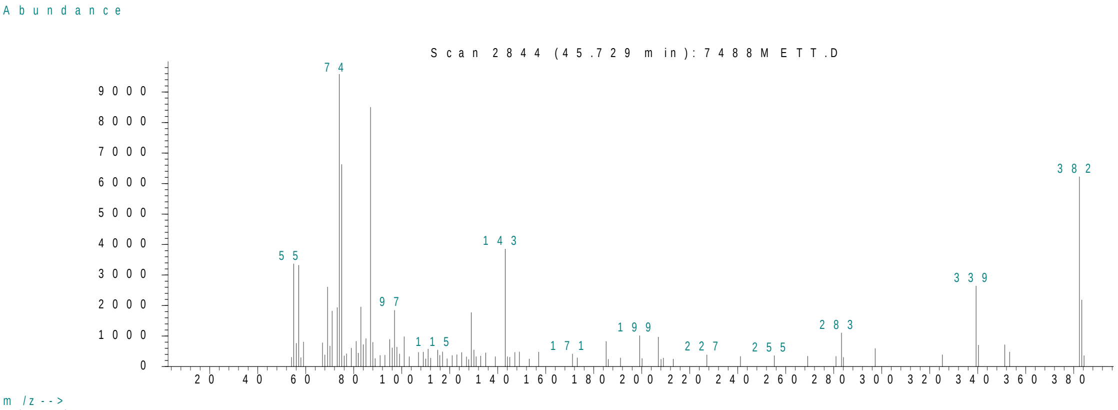
Abundance
Scan 2844 (45.729 min): 7488METT.D
74
55
97
115
143
171
199
227
255
283
339
382
m/z-->

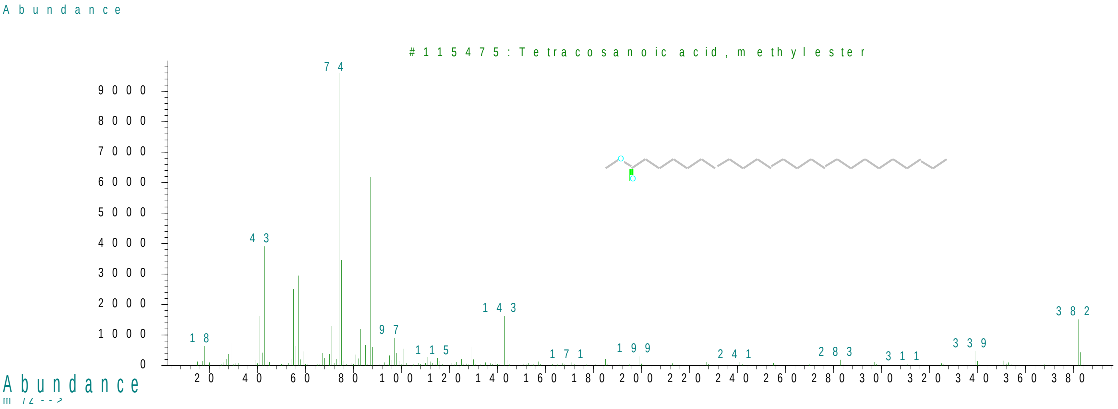
Abundance
#115475: Tetracosanoic acid, methyl ester
74
43
18
97
115
143
171
199
241
283
311
339
382
m/z-->

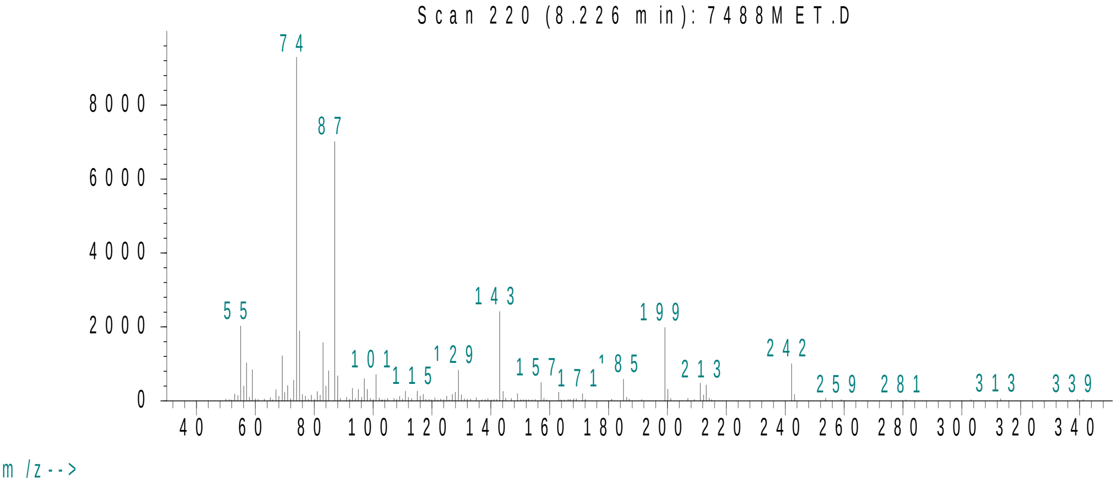
Abundance
Scan 220 (8.226 min): 7488MET.D
74
87
55
101
115
129
143
157
171
185
199
213
242
259
281
313
339
m/z-->

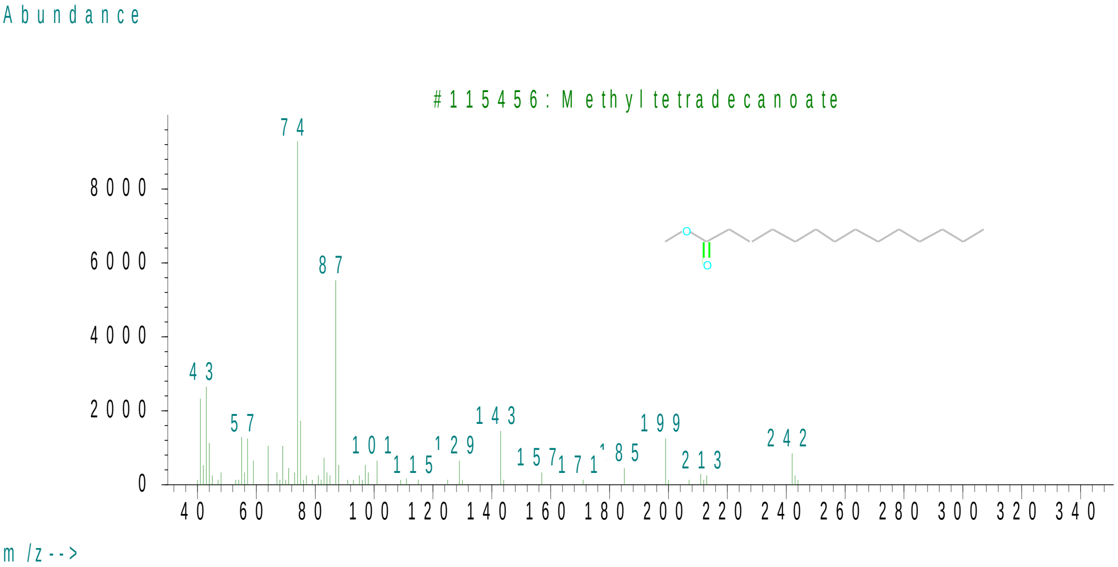
Abundance
#115456: Methyltetradecanoate
74
87
43
57
101
115
129
143
157
171
185
199
213
242
m/z-->

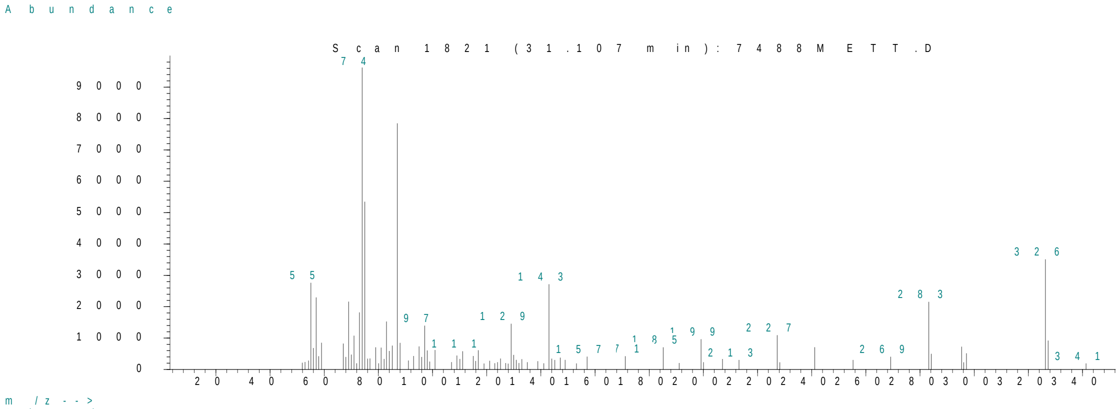
Abundance
Scan 1821 (31.107 min): 7488METT.D
m/z-->

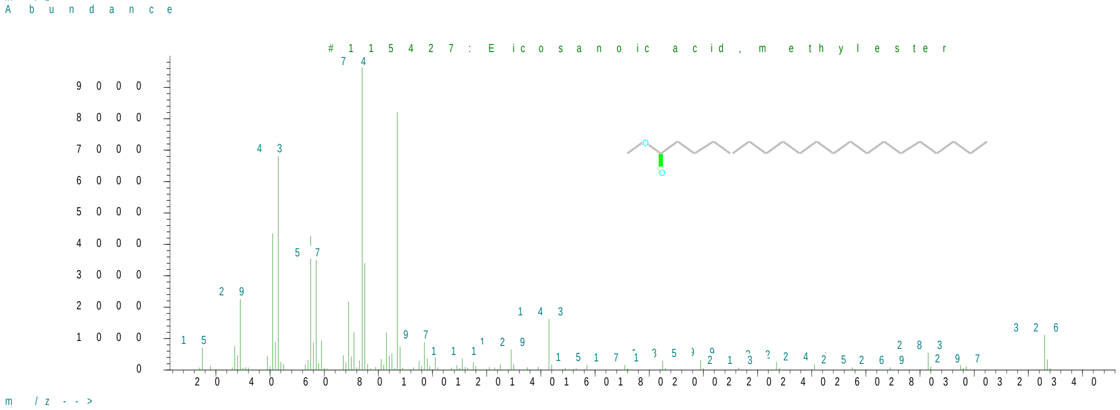
Abundance
#115427: Eicosanoic acid, methyl ester
m/z-->

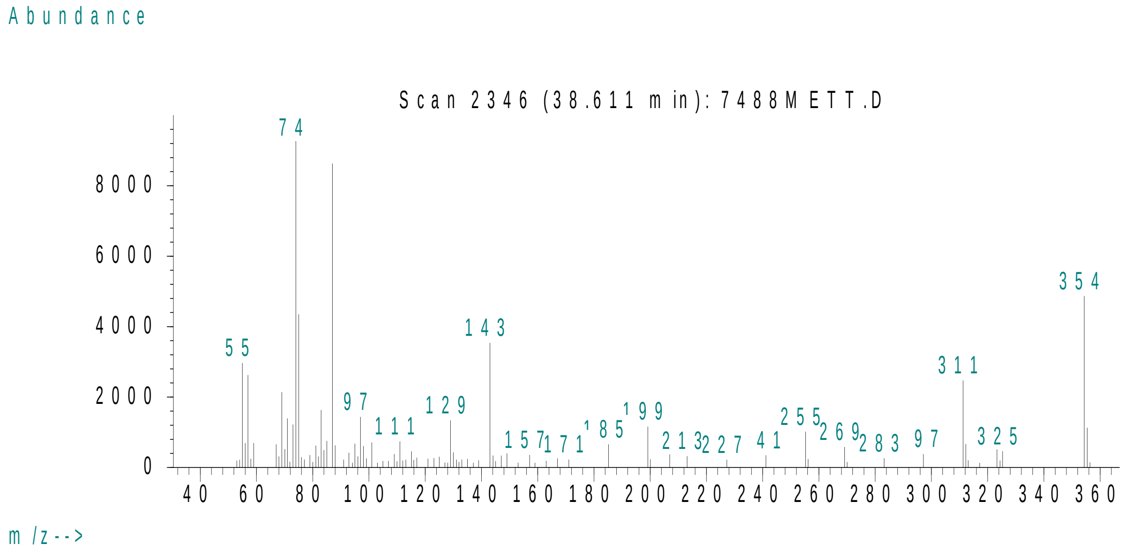
Abundance
Scan 2346 (38.611 min): 7488METT.D
m/z-->

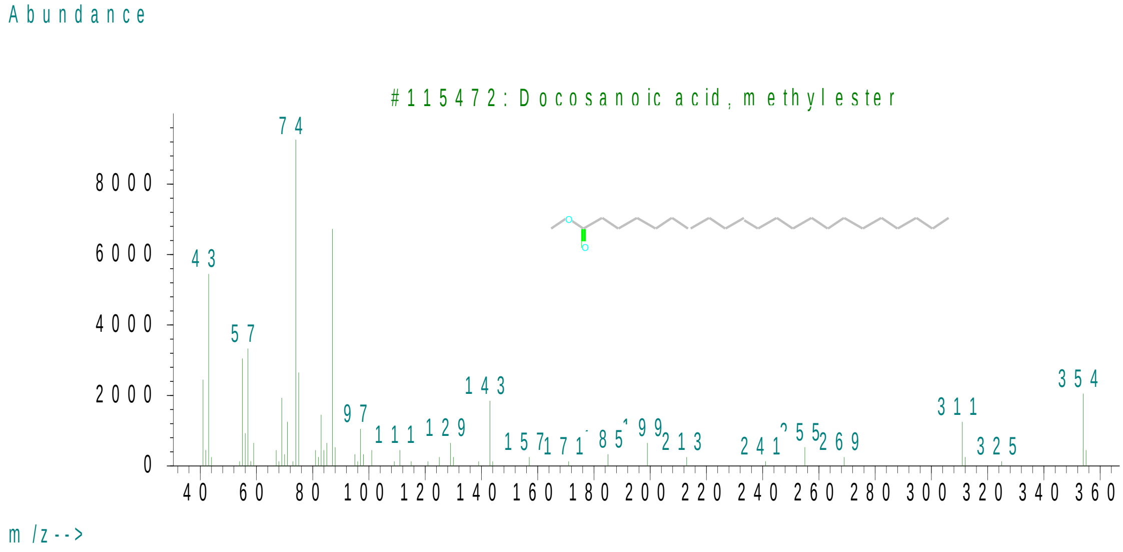
Abundance
#115472: Docosanoic acid, methyl ester
m/z-->

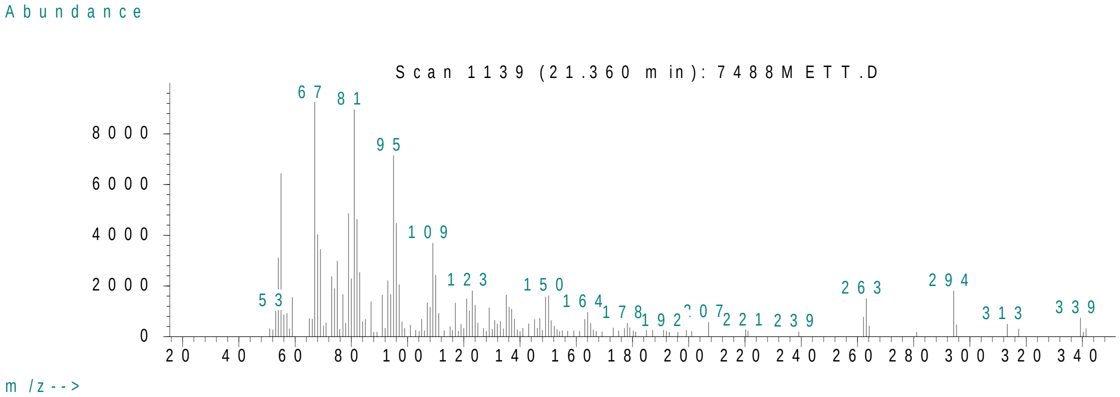
Abundance
Scan 1139 (21.360 min): 7488METT.D
67
81
95
109
123
150
164
178
53
263
294
313
339
221
239
8000
6000
4000
2000
0
20 40 60 80 100 120 140 160 180 200 220 240 260 280 300 320 340
m/z-->

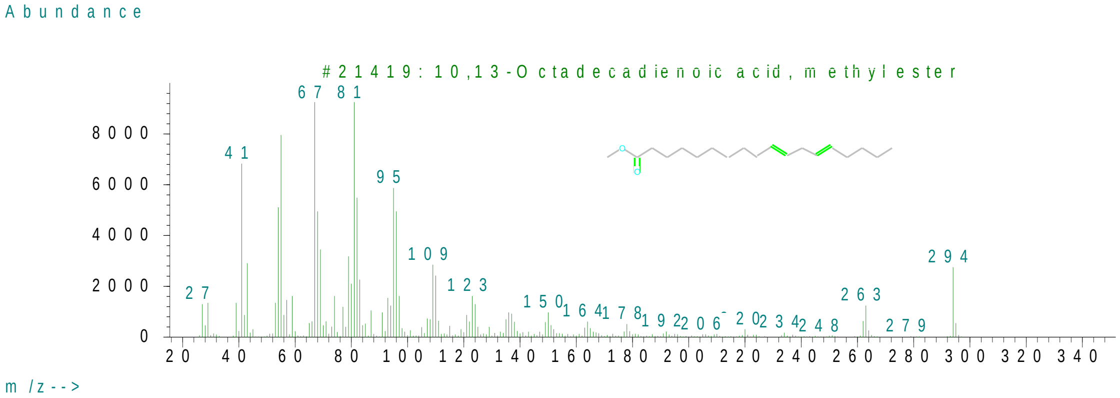
Abundance
#21419: 10,13-Octadecadienoic acid, methyl ester
67
81
41
95
109
123
150
27
263
294
279
8000
6000
4000
2000
0
20 40 60 80 100 120 140 160 180 200 220 240 260 280 300 320 340
m/z-->

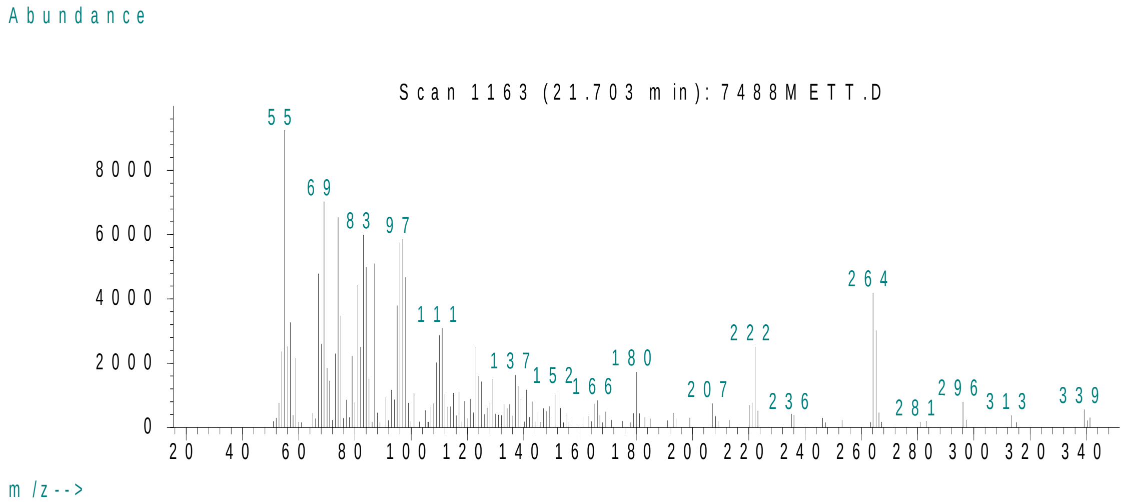
Abundance
Scan 1163 (21.703 min): 7488METT.D
55
69
83
97
111
137
152
166
180
207
222
236
264
281
296
313
339
8000
6000
4000
2000
0
20 40 60 80 100 120 140 160 180 200 220 240 260 280 300 320 340
m/z-->

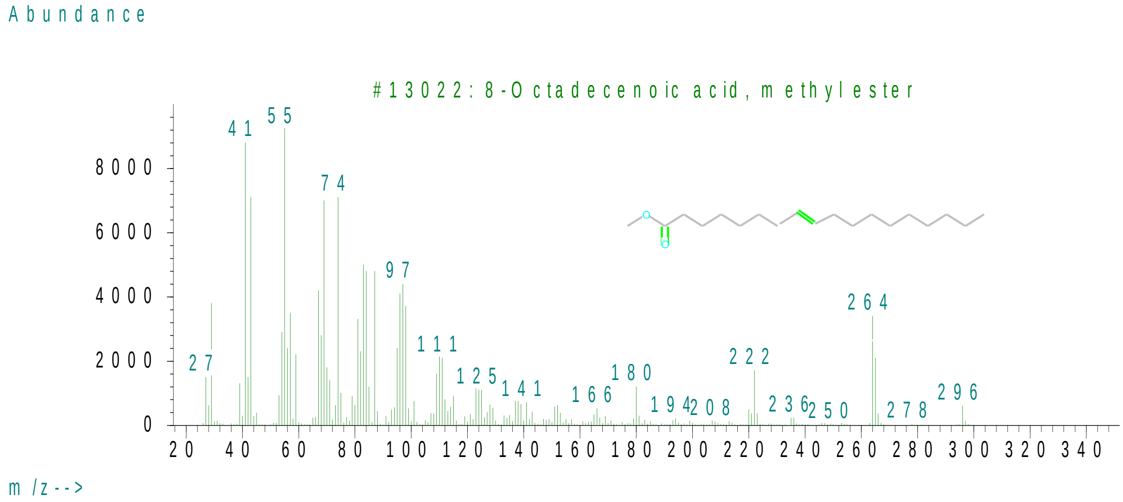
Abundance
#13022: 8-Octadecenoic acid, methyl ester
41
55
74
97
27
111
125
141
166
180
208
222
236
250
264
278
296
8000
6000
4000
2000
0
20 40 60 80 100 120 140 160 180 200 220 240 260 280 300 320 340
m/z-->

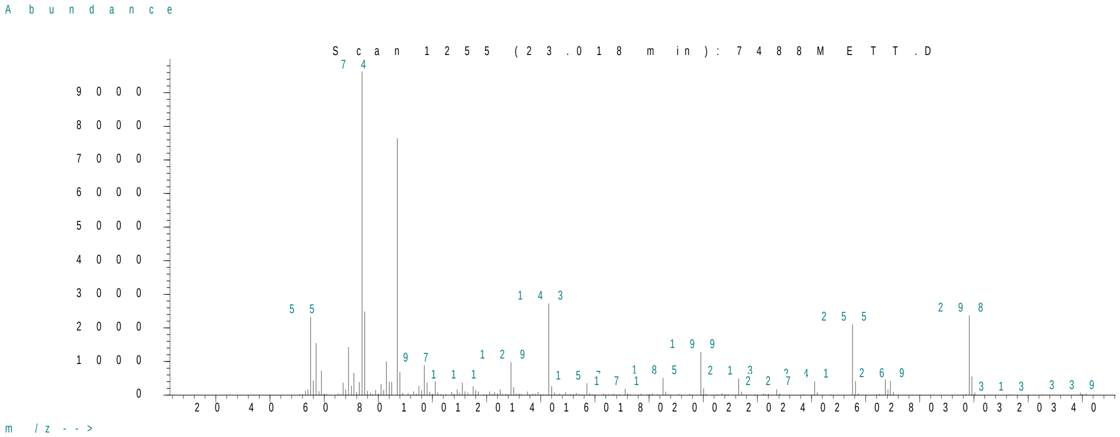
Abundance
Scan 1255 (23.018 min): 7488METT.D
m/z-->

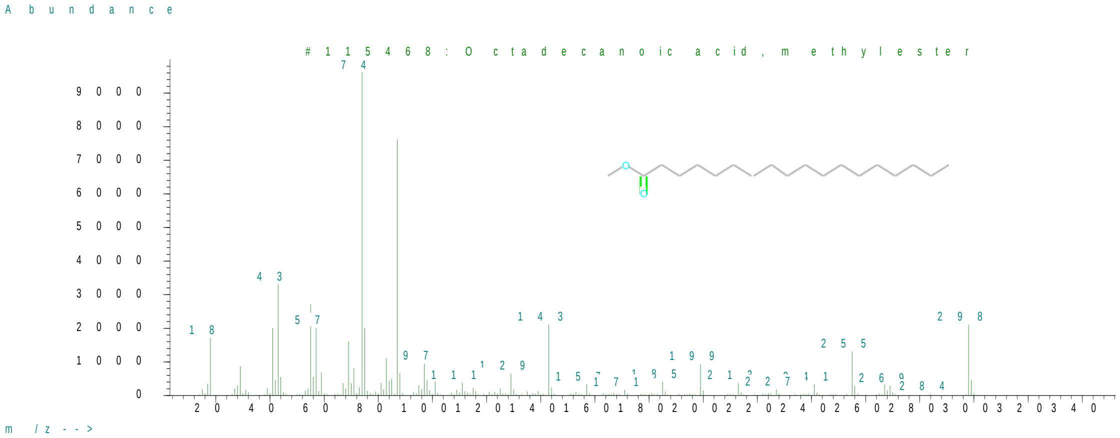
Abundance
#115468: Octadecanoic acid, methyl ester
m/z-->

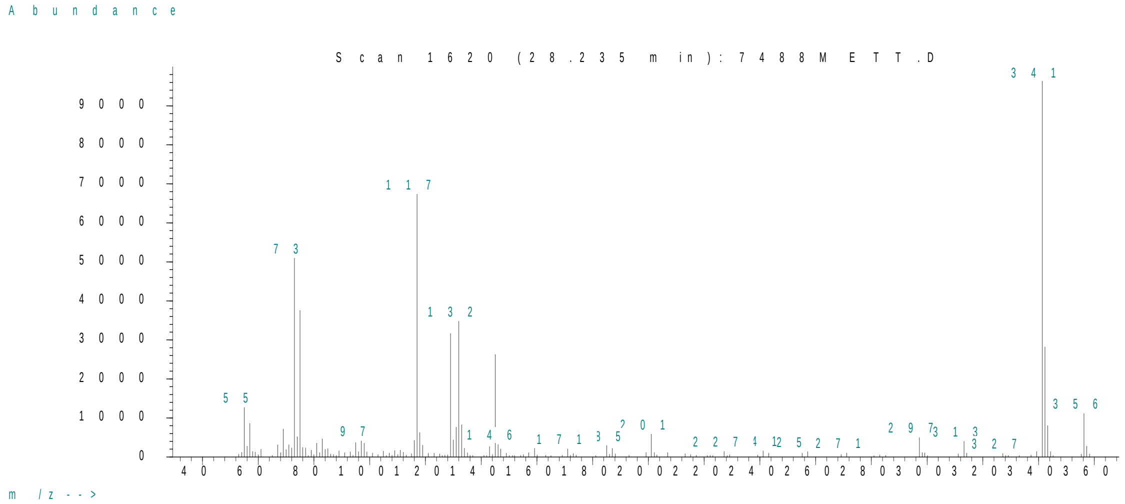
Abundance
Scan 1620 (28.235 min): 7488METT.D
m/z-->

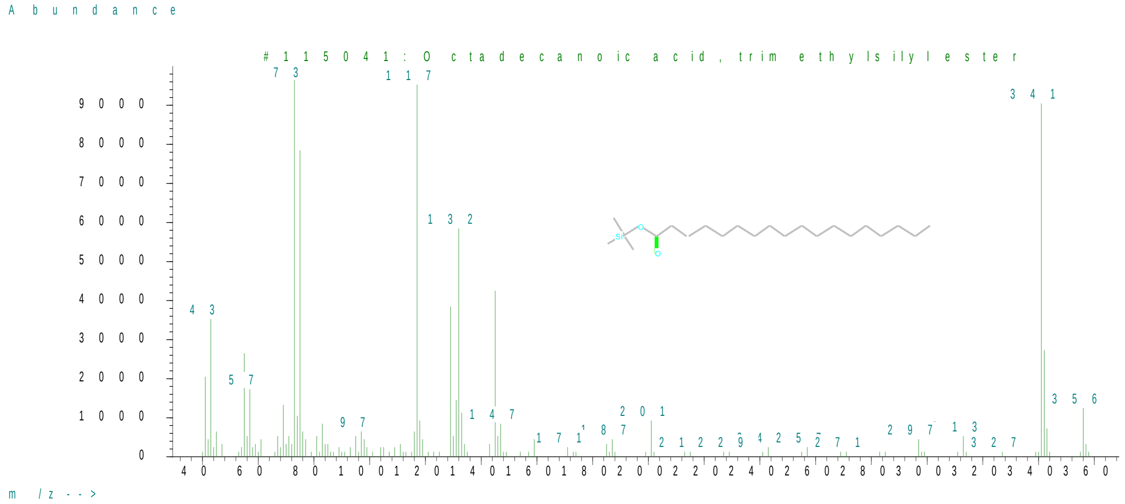
Abundance
#115041: Octadecanoic acid, trimethylsilyl ester
m/z-->

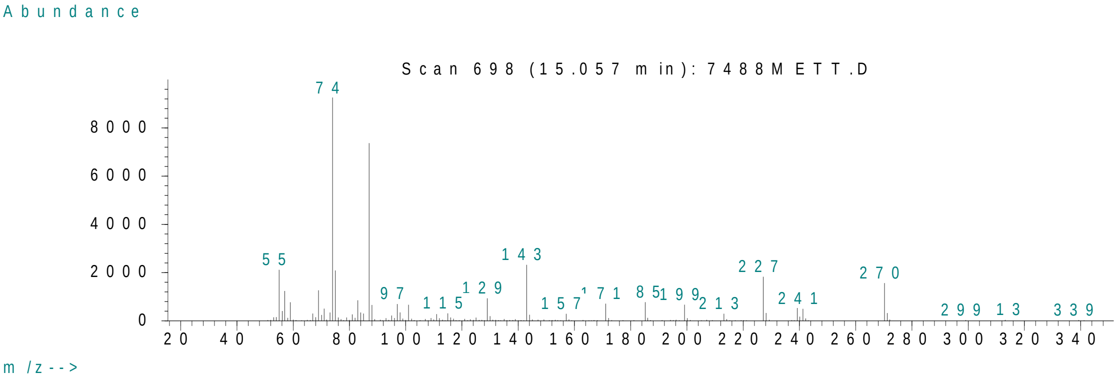
Abundance
Scan 698 (15.057 min): 7488METT.D
74
55
143
227
270
97
115
129
157
171
185
199
213
241
299
313
339
m/z-->

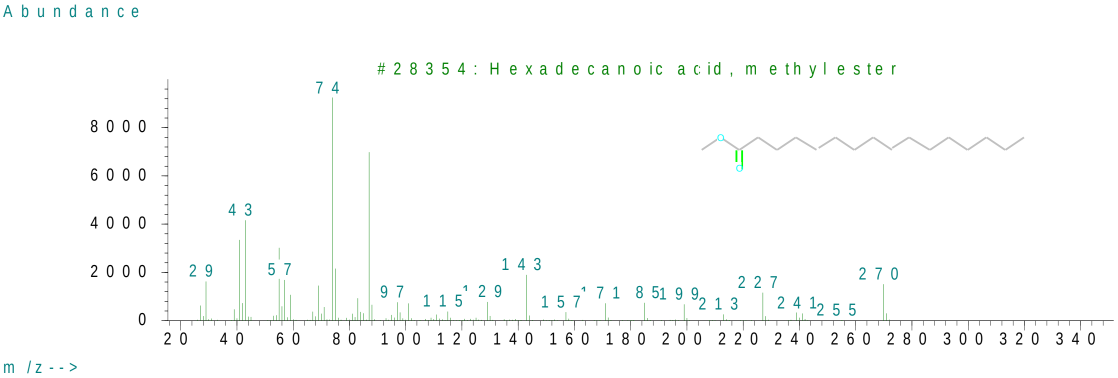
Abundance
#28354: Hexadecanoic acid, methyl ester
74
43
29
57
143
227
270
97
115
129
157
171
185
199
213
241
255
m/z-->

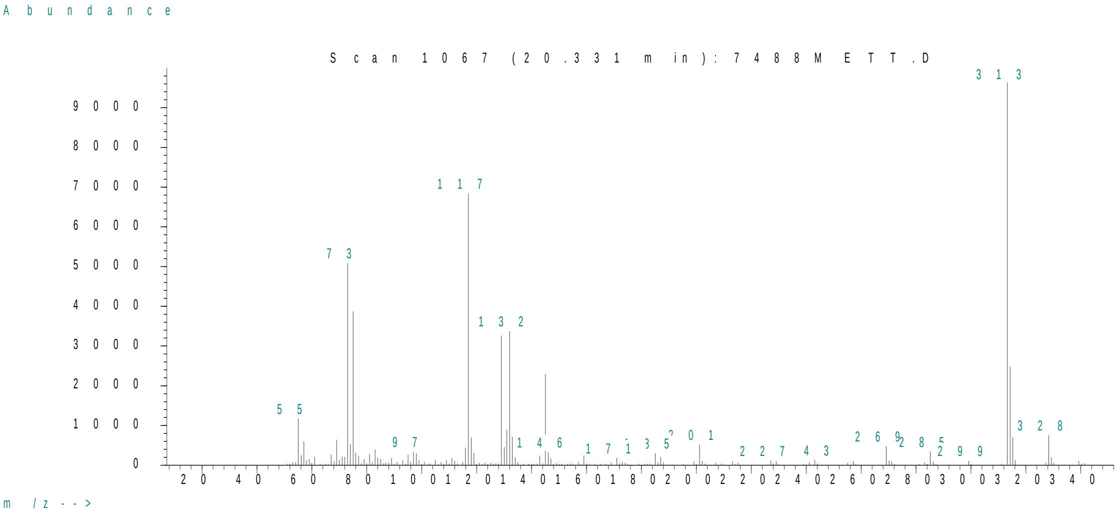
Abundance
Scan 1067 (20.331 min): 7488METT.D
313
117
73
132
55
97
146
201
227
243
269
285
299
328
m/z-->

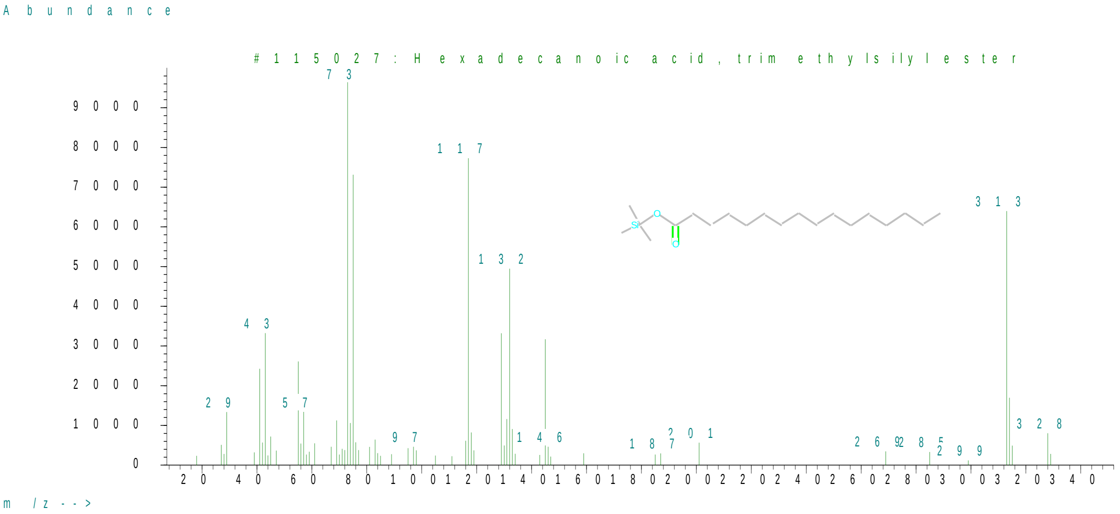
Abundance
#115027: Hexadecanoic acid, trimethylsilyl ester
73
117
313
132
43
29
57
97
146
187
201
269
285
299
328
m/z-->

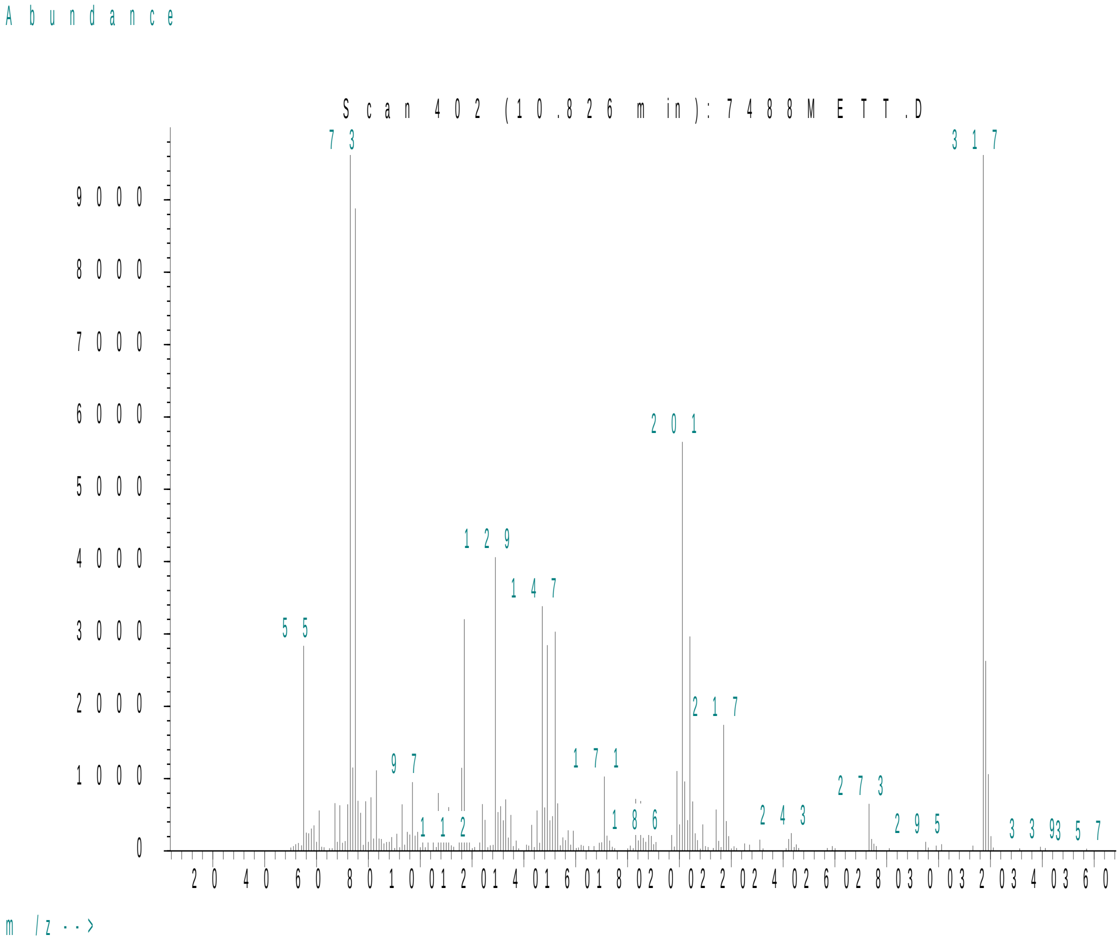
Abundance
Scan 402 (10.826 min): 7488METT.D
73
317
201
129
147
55
217
171
97
112
186
243
273
295
339
357
m/z-->

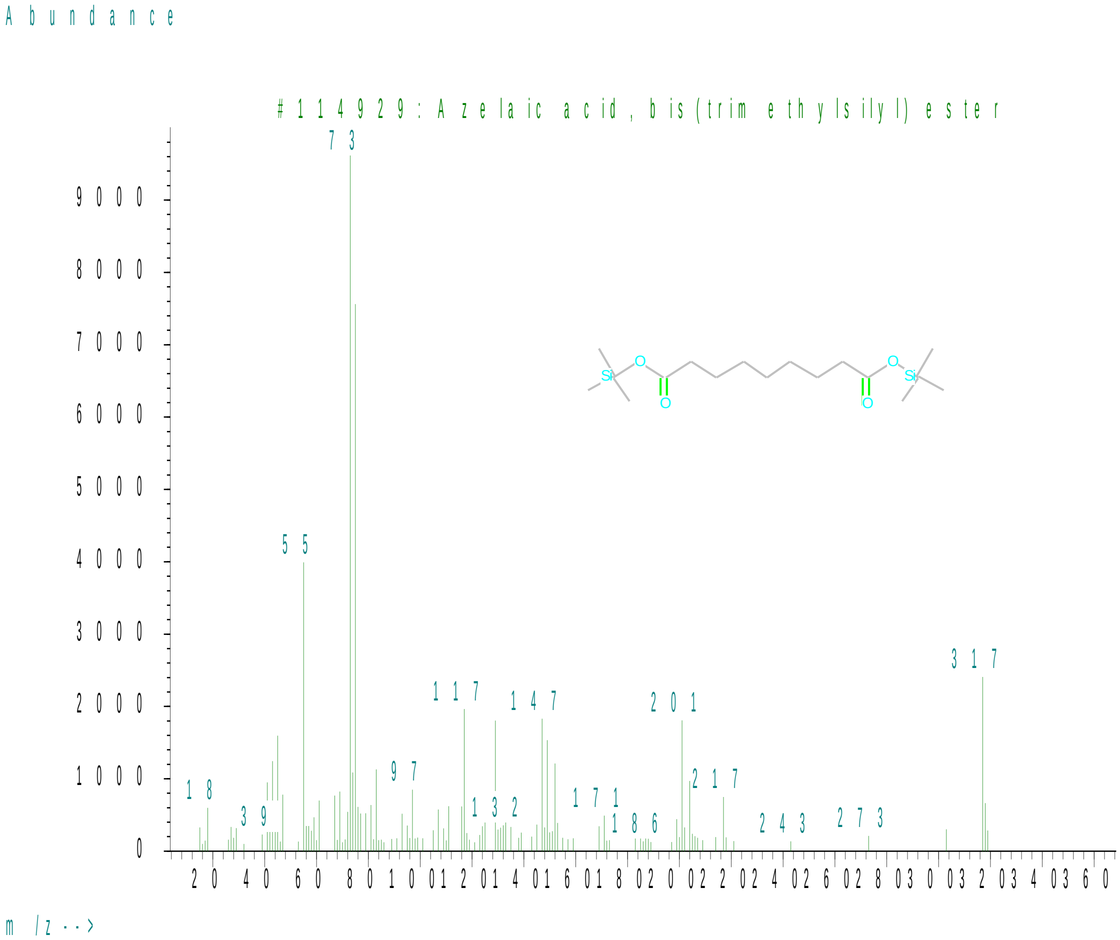
Abundance
#114929: Azelaic acid, bis(trimethylsilyl) ester
73
55
117
147
201
317
97
132
171
186
217
243
273
18
39
m/z-->

Panossian A, Amaterdam J. 2017. Adaptogens in Psychiatric Practice. Chapter 8 , Pp 155-181 in: Complementary and Integrative Treatments. Eds. PL. Gerbarg, PR. Muskin, RP. Brown, American Psychiatric Association Publishing. Arlington, 2018, 425 pages. https://www.appi.org/Complementary_and_Integrative_Treatments_in_Psychiatric_Practice

CHAPTER 8

Adaptogens in Psychiatric Practice

Rhodiola rosea, Schisandra chinensis, Eleutherococcus senticosus, and *Withania somnifera*

Alexander Panossian, Ph.D., D.Sci.
Jay D. Amsterdam, M.D.

Health is the ability to adapt to one's environment.

Georges Canguilhem, Le Normal et le pathologique, *1943*

In this chapter, we review molecular mechanisms and clinical studies of four botanical adaptogens: golden root (*Rhodiola rosea* [L.], radix and rhizome), Siberian ginseng (*Eleutherococcus senticosus*), schisandra (*Schisandra chinensis* [Turcz.] Baill., fructus), and ashwagandha (*Withania somnifera* [L.] Dunal, radix). These herbs are of particular interest because they have an adequate evidence base of safety and efficacy for treating disorders commonly encountered in psychiatric practice. Used for thousands of years in traditional folk medicines, they are included as conventional therapies in several national pharmacopoeias in Asia and Europe for treatment of neuropsychiatric disorders.

Botanical adaptogens are plant extracts and constituents that increase the ability of an organism to tolerate, adapt, survive, and perform under a wide range of stressors. The term *adaptogen* was introduced in 1959 to describe substances that increase the "state of non-specific resistance" of an organism under stress, "normalize" physiological states, and mitigate the effects of environmental or emotional stress (Brekhman and Dardymov 1969). Recent definitions of adaptogen may include mechanisms of action such as upregulating expression of neuropeptide Y (NPY) and heat shock protein 70 (Hsp70; Asea et al. 2013; Panossian and Wikman 2010; Panossian et al. 1999).

Molecular Mechanisms

Adaptive stress response involves activation of intracellular and extracellular signaling pathways and increased expression of antiapoptotic proteins, neuropeptides, and antioxidant enzymes. Accordingly, botanical adaptogens have been proposed to act as mild stress "vaccines," inducing stress-protective responses. Although acute administration of an adaptogen may produce a stimulatory effect, the stress-protective effects of chronic adaptogen administration are more likely the result of adaptive changes in response to repeated stress-mimetic or booster vaccine–like effects. These may be mediated through the hypothalamic-pituitary-adrenal (HPA) axis and nitric oxide (Panossian and Wagner 2005).

Russian medical manuals recognize adaptogens as a group of synthetic and natural medications with stimulant and/or psychotropic properties, which are prescribed for psychiatric disorders such as asthenia (mental or physical fatigue), neurosis, depression, and alcoholism and as an adjuvant with conventional psychotropics (Mashkovskiy 2000; Panossian and Wikman 2008; Panossian et al. 2010). Compared with prescription stimulants such as amphetamines, adaptogens are lower in side effects, provide a better quality of arousal, have no addiction potential, cause no withdrawal syndrome, do not deplete energy, enhance performance and survival under stress, improve recovery after exhausting physical workload, and cause no loss of appetite (Panossian 2003).

Adaptogens may exert *polyvalent* (more than one direction of change) biological activity and multi-target effects on transcriptional, proteomic, and metabolomic regulation, potentially affecting signaling pathways and molecular networks with a beneficial effect on stress-induced limbic-hypothalamic–mediated responses (Panossian et al. 2012, 2013, 2014). The following molecular mechanisms have been proposed to contribute to adaptogen effects on neuroendocrine regulation of the HPA axis through mediators of stress response (see Panossian 2013 for review of molecular mechanisms): molecular chaperones Hsp70 and NPY, membrane-bound G-protein-coupled receptors (GPCRs) and G-protein-signaling pathways, regulation of cyclic adenosine monophosphate (cAMP) and protein kinase A (PKA), and G-protein-signaling phosphatidylinositol and phospholipase C pathways.

Molecular Chaperones

HEAT SHOCK PROTEIN 70

Hsp70 proteins protect cells from stress-induced damage by temporarily binding to partially denatured proteins, preventing aggregation and allowing repair of the proteins. In addition, Hsp70 participates in disposal of damaged or defective proteins and inhibits programmed cell death. In mice, ADAPT-232 (combination of *R. rosea*, *E. senticosus*, and *S. chinensis*) significantly increased stress tolerance, accompanied by a dramatic increase in serum levels of circulating Hsp70. ADAPT-232 and one active constituent, salidroside, both stimulated expression and release of Hsp70 from isolated human neuroglial cells (Panossian et al. 2012).

NEUROPEPTIDE Y

Salidroside and ADAPT-232 stimulate expression and release of NPY from isolated human brain glioblastoma T98G cells (Panossian et al. 2012). NPY is a stress-responsive hormone present throughout the central and peripheral nervous systems. NPY stimulates the HPA axis and modulates secretion of hypothalamic neuropeptides. Stressors such as strenuous exercise, cold exposure, panic disorder, and chronic fatigue syndrome (CFS) produce sympathoadrenal activation and NPY release from sympathetic nerve endings. The elevation of serum NPY in CFS patients correlates with the severity of stress, negative mood, and clinical symptoms. In healthy subjects, psychological stress elevates plasma NPY. In the periphery, sympathetic nerve–derived and platelet-derived NPY are stimulatory, potentiate stress response synergistically with glucocorticoids and catecholamines, and induce vasoconstriction. In the central nervous system, NPY paradoxically serves as an anxiolytic and inhibits sympathetic activity and cortisol production (Hirsch and Zukowska 2012). NPY can regulate immune response, that is, inhibit nitric oxide synthesis, which prevents interleukin (IL-1β) release. NPY promotes adenosine triphosphate (ATP) formation by decreasing expression of mitochondrial uncoupling protein.

Preclinical and clinical evidence suggests mood and cognitive enhancement by NPY. Elevated NPY concentration was found in soldiers who had reduced psychological distress or who belonged to elite Special Forces. In contrast, decreased NPY levels were documented in depressed patients and in brain tissues of persons who committed suicide (Panossian 2013). The release of stress hormones, NPY, and Hsp70 into the blood can be considered an innate defense response to mild stressors, such as adaptogens, which increases tolerance and adaptation to stress.

Membrane-Bound G-Protein-Coupled Receptors and G-Protein-Signaling Pathways

Genes that encode cell membrane–bound GPCRs include serotonin type 3 receptor (5-HT_3) and key proteins of G-protein intracellular signaling downstream pathways: cAMP and phospholipase C/phosphatidylinositol (Panossian et al. 2013). GPCRs are involved in many physiological processes related to stress tolerance:

- Binding of neurotransmitters, including serotonin, dopamine, γ-aminobutyric acid (GABA), and glutamate, that are involved in regulation of mood, behavior, and cognitive functions
- Regulation of sympathetic and parasympathetic nervous systems
- Regulation of immune system activity and inflammation
- Maintaining homeostasis in response to stressors

Many neuropsychiatric drugs either bind directly to specific GPCRs (e.g., antipsychotics) or act indirectly via GPCRs, affecting the amount of available agonist (e.g., antidepressants). Adaptogens downregulate the gene *HTR1A* encoding the 5-HT_3 GPCR that activates an intracellular second-messenger cascade to produce excitatory or inhibitory neurotransmission. Serotonin receptors modulate release of glutamate, GABA, dopamine, epinephrine, norepinephrine, and acetylcholine, as well as hor-

mones, including oxytocin, prolactin, vasopressin, cortisol, corticotropin, and substance P. Serotonin receptors influence aggression, anxiety, appetite, cognition, learning, memory, mood, nausea, sleep, and thermoregulation. Downregulation of *HTR1A* by salidroside and tyrosol (active constituents of *R. rosea*) is consistent with involvement of serotonin receptors in preclinical and clinical effects of *R. rosea*, including the antidepressant effects (Panossian et al. 2014).

Regulation of Cyclic Adenosine Monophosphate and the Activity of Protein Kinase A

In brain cells, adaptogens regulate the dopamine-cAMP-PKA–ceramide transfer protein (CERT) signaling pathway (Panossian et al. 2013; Rao et al. 2007). Adaptogens downregulate expression of adenylate cyclase, which activates generation of cAMP from ATP and upregulates expression of phosphodiesterases, which activates degradation of cAMP (Panossian et al. 2013). Predicted downstream effect of *Rhodiola* and salidroside is an inhibition of PKA- and mitogen-activated protein kinase kinase (MEK)–mediated pathways followed by inhibition of cAMP response element-binding protein, a transcription factor involved in both the mechanism of action of antidepressants and the disease itself. Regulation of cAMP and PKA is a key mechanism of energy homeostasis and metabolism—the shifting between catabolic and anabolic states. Downregulation of cAMP and PKA by adaptogens decreases stress-induced catabolic transformations and is associated with stress-protective effects. Inhibition of adenylate cyclase by adaptogens can increase intracellular ATP (Abidov et al. 2003) when less of it is converted to cAMP. This contributes to the ability of adaptogens to maintain energy supplies during stress over long periods of time.

Prefrontal cortex cells contain hyperpolarization-activated channels that can open when exposed to cAMP during stress. Excess opening of these channels has been associated with impairment of higher cognitive function. It has been suggested that cAMP inhibitors may close the channels, enabling neurotransmission of information connecting neural networks, thus improving working memory, which plays a key role in such tasks as abstract thinking, planning, and organizing. This mechanism could contribute to treatment of age-related cognitive decline and cognitive dysfunctions in schizophrenia, bipolar disorder, and attention-deficit/hyperactivity disorder. It may contribute to cognitive-enhancing effects of ADAPT-232 and *R. rosea* in humans (Panossian et al. 2013, 2014).

G-Protein-Signaling Phosphatidylinositol and Phospholipase C Pathways

Adaptogens upregulate the gene *PLCB1* encoding phosphoinositide-specific phospholipase C and phosphatidylinositol 3-kinases (Panossian et al. 2013). G proteins catalyze the hydrolysis of phosphatidylinositol 4,5-bisphosphate into diacylglycerol and inositol-1,4,5-triphosphate, which is involved in cellular signaling pathways associated with depression. Phosphatidylinositol 4,5-bisphosphate is required for the long-term potentiation (long-lasting enhancement in neuronal signal transmission), which is critical for memory and learning.

Safety and Effectiveness of Adaptogens in Psychiatric Disorders

Studies of adaptogens for treatment of psychological conditions have been published since the 1940s. The methodology of most of the older studies does not meet current criteria, and diagnostic categories differ from those in use today. Nevertheless, such studies provide useful information regarding potential benefits that would be worth current investigation. Level I and II evidence (defined in Table 8–2 footnote) supports the use of *R. rosea* for fatigue, life stress, and depression; *S. chinensis* for depression; *E. senticosus* for bipolar disorder; and *W. somnifera* for bipolar disorder, anxiety, and schizophrenia (Tables 8–1 and 8–2). For insomnia, *S. chinensis* and *W. somnifera* have shown benefits. In addition, *R. rosea*, *S. chinensis*, and *E. senticosus* have been found to improve cognitive function. Tables 8–1 and 8–2 summarize research in mental disorders and potential applications; details on active constituents can be found in Figure 8–1. For a summary of clinical trials of *E. senticosus*, *R. rosea*, *S. chinensis*, and *W. somnifera* in mental disorders in tabulated form, see Panossian and Wikman (2010; available at www.mdpi.com/1424-8247/3/1/188/pdf). Table 8–3 summarizes clinical trials of *W. somnifera* in anxiety and bipolar disorders.

TABLE 8–1. *Rhodiola rosea*, *Eleutherococcus senticosus*, *Schisandra chinensis*, and *Withania somnifera*: types of evidence and applications

	Type of evidence					
**Herbal medicine**	**Dep**	**Anx**	**Ins**	**Sch**	**Ast**	**Potential applications**
Roseroot (*R. rosea*)	1, 2, 3	1, 2, 3	–	–	1, 2, 3	Stress-induced fatigue, cognitive impairment, depression, anxiety
Schisandra (*S. chinensis*)	1	2	2	1	1, 2, 3	Fatigue, cognitive impairment, nervous exhaustion, schizophrenia
Siberian ginseng (*E. senticosus*)	1, 2	2	–	–	1, 2, 3	Chronic fatigue, cognitive impairment, bipolar disorder
Withania (*W. somnifera*)	1, 2, 3	1, 2, 3	2, 3	1, 2	1, 2, 3	Anxiety, insomnia, bipolar disorder

Note. Anx=anxiety; Ast=asthenia, fatigue; Dep=depression; Ins=insomnia; Sch=schizophrenia. 1=human clinical data; 2=experimental preclinical evidence of activity; 3=traditional systems of medicine and pharmacopoeias endorse use.

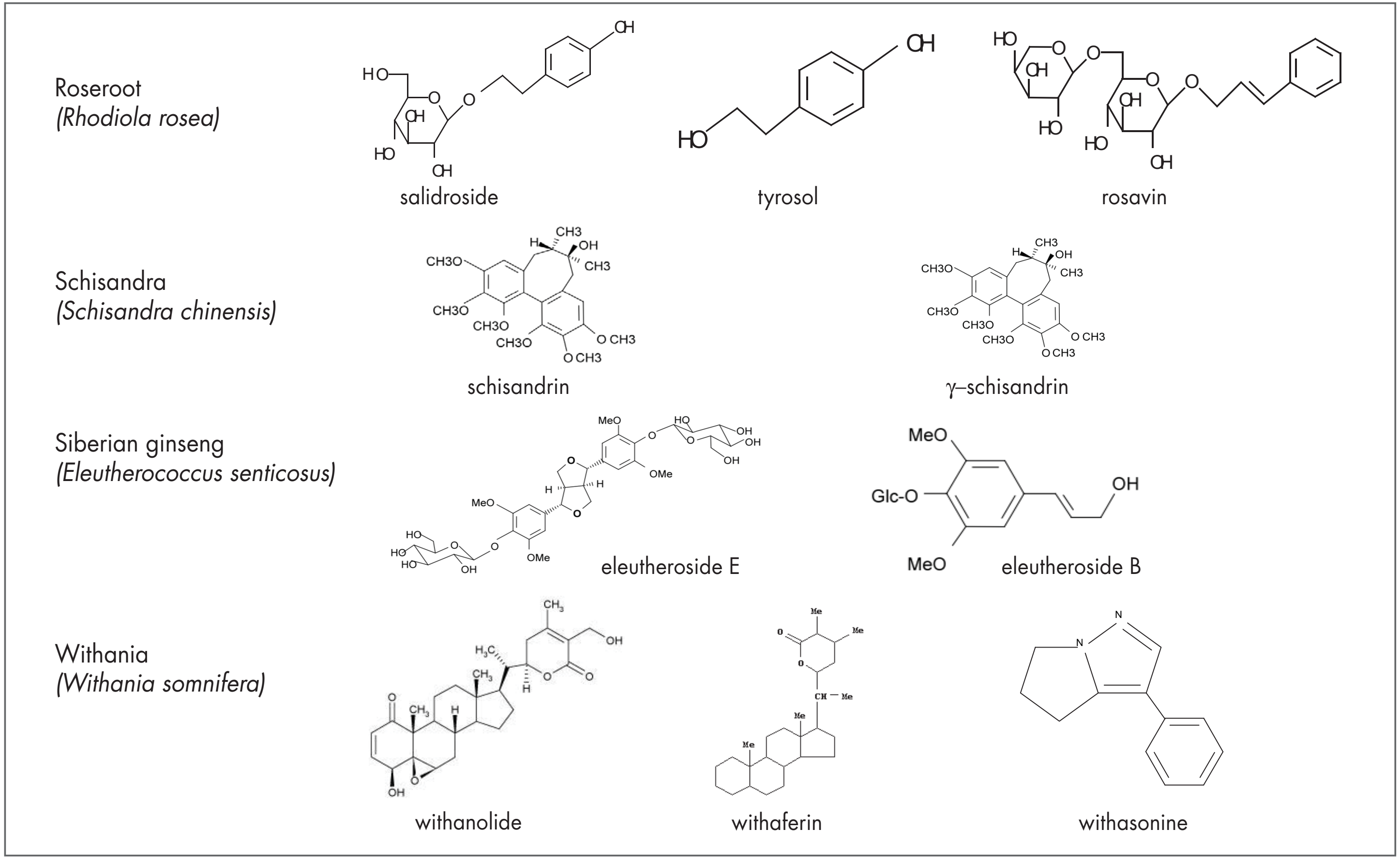

FIGURE 8–1. Major active constituents of *R. rosea*, *S. chinensis*, *E. senticosus*, and *W. somnifera*.

TABLE 8–2. Clinical trials of *Eleutherococcus senticosus, Rhodiola rosea, Schisandra chinensis,* and *Withania somnifera* in psychiatric disorders

Condition	Adaptogen	Design	Duration, weeks	Jadad score[b]	Number of subjects	Level of evidence[e]	Reference[f]
Chronic fatigue syndrome	*E. senticosus*	DBRPCT	4, 8, 16	5	96	Ib	Hartz (2004)
Fatigue syndrome (stress induced)	*R. rosea*	DBRPCT	4	5	60	Ib	Olsson et al. (2009)
Life-stress symptoms	*R. rosea*	OL	4	1	101	IIa	Edwards et al. (2012
Diagnosis	*R. rosea*	OL	6, 12	0	120	III	Fintelmann and Gruenwald (2007)
Depression	*R. rosea*	DBRPCT	12	5	57	Ib	Mao et al. (2014, 2015)
	R. rosea	DBRPCT	6	5	91	Ib	Darbinyan et al. (2007)
	R. rosea	OL, C[a]		0	78/56[a]	IIa	Brichenko (1986)[a]
	S. chinensis	OL, UC		0	37		Staritsina (1946)
Astheno-depressive syndrome (stress-induced depression)	*R. rosea*	OL, UC	2–3	0	128		Krasik et al. (1970)d
	R. rosea	OL, UC	2–3	0	135/27		Krasik et al. (1970)[d]
	R. rosea	OL	8	0	58		Mikhailova (1983)
	S. chinensis	OL, UC		0	13		Zakharov (1956)
	S. chinensis	OL, UC		0	40		Leman (1952)
	S. chinensis	OL	2–6		36		Galant (1958)
	S. chinensis	OL	1.5		30		Zakharova (1948)

TABLE 8–2. Clinical trials of *Eleutherococcus senticosus, Rhodiola rosea, Schisandra chinensis,* and *Withania somnifera* in psychiatric disorders *(continued)*

Condition	Adaptogen	Design	Duration, weeks	Jadad score[b]	Number of subjects	Level of evidence[e]	Reference[f]
Neurosis (stress-induced depression)	*R. rosea*	OL	1.5	0	65		Saratikov 1965)[d]
	R. rosea	SBPC		1	70/80[c]		Kaliko (1966)
	S. chinensis	OL	2–8	0	386[d]		Sudakov (1986)
	S. chinensis	OL	2–10	0	250		Rossijskij (1952)
	E. senticosus	OL, PC	3–4	2	80		Strokina (1966, 1967)
Bipolar disorder/manic depression	*W. somnifera*	DBRPCT	8	4	60	Ib	Chengappa et al. (2013)
	W. somnifera	DBRPCT	8	3	60		Gannon et al. (2014)
	E. senticosus		6	5	76	Ib	Weng et al. (2007)
Anxiety	*R. rosea*	OL	10	0	10	III	Bystritsky (2008)
	W. somnifera	DBRPCT	8.5	2	64	Ib	Chandrasekhar et al. (2012)
	W. somnifera	R, C, PC	6	1	40/41		Cooley et al. (2009)
	W. somnifera	DBRPCT	6	2	39		Andrade et al. (2000)
	W. somnifera	DBRPCT	8.5	3	30/35/35/30		Auddy et al. (2008)

TABLE 8–2. Clinical trials of *Eleutherococcus senticosus, Rhodiola rosea, Schisandra chinensis,* and *Withania somnifera* in psychiatric disorders *(continued)*

Condition	Adaptogen	Design	Duration, weeks	Jadad score[b]	Number of subjects	Level of evidence[e]	Reference[f]
Schizophrenia	*S. chinensis*	OL	?	0	79/41[d]	III	Romas (1958, 1962)
	S. chinensis	OL, C	?	0	30/20[d]		Zakharova (1948)[d]
	S. chinensis	OL	?	0	48		Lastovetskiy (1963)
	W. somnifera	DBRPCT	4.5	2	30	IIa	Agnihotri et al. (2013)

Note. C=controlled; CO=crossover; DBRPCT=double-blind, randomized placebo-controlled trial; OL=open-label; PC=placebo-controlled; R=randomized; SB=single-blind; UC=uncontrolled.

[a]The control group was treated with tricyclic antidepressants; the study group was treated with tricyclic antidepressants and *R. rosea* as adjuvant therapy.

[b]Jadad AR, Moore RA, Carroll D, et al: "Assessing the quality of reports of randomized clinical trials: is blinding necessary?" *Controlled Clinical Trials* 17:1–12, 1996.

[c]Neurotic, stress-related, and somatoform disorders (ICD-10-CM F40–F48).

[d]Mixed patient population, sick/healthy subjects.

[e]According to the World Health Organization, U.S. Food and Drug Administration, and European Medicines Evaluation Agency, Ia=meta-analyses of randomized and controlled studies; Ib=evidence from at least one randomized study with control group; IIa=evidence from at least one well-performed study with control group; IIb=evidence from at least one well-performed quasi-experimental study; III=evidence from well-performed nonexperimental descriptive studies as well as comparative studies, correlation studies, and case studies; IV=evidence from expert committee reports or appraisals and/or clinical experiences by prominent authorities.

[f]For references not listed in the reference list, see Panossian and Wikman (2010).

Grade of recommendation based on the European Medicines Agency Assessment Scale (European Medicines Agency. Committee on Medicinal Products EMEA/HMPC/104613/2005. Available at: http://www.ema.europa.eu/ema/index.jsp?curl=pages/regulation/general/general_content_000830.jsp&mid=WC0b01ac0580033a9b. Accessed February 2, 2017):

- *Grade A: Evidence levels quality Ia, Ib*—Requires at least one randomized controlled trial as part of the body of literature of overall good consistency addressing the specific recommendation
- *Grade B: Evidence levels IIa, IIb, III*—Requires availability of well-conducted clinical studies but no randomized clinical trials on the topic of recommendation
- *Grade C: Evidence level IV*—Requires evidence from expert committee reports or opinions and/or clinical experience of respected authorities but indicates absence of directly applicable studies of good quality

Source. Updated and adapted from Panossian and Wikman 2010; www.mdpi.com/1424-8247/3/1/188/pdf.

TABLE 8–3. Summary of clinical trials: *Withania somnifera* in anxiety and bipolar disorders

Author, year	Sample size	Design	Duration, weeks	Daily dose, extraction solvent, marker, trade name	Diagnosis	Primary outcomes	Adverse events (*n*)	Jadad score[b]
Gannon et al. (2014)[a]	30+30	DBRPCT	8	500 mg NS, NS, NS, Sensoril	Bipolar disorder	Serum thyroxine	NS	2
Chengappa et al. (2013)[a]	24+29	DBRPCT	8	500 mg, 250-mg capsules water, 8% withanolides, 2% withaferin Sensoril	DSM-IV bipolar disorder, YMRS, MADRS, and HARS scores	Cognitive tasks: auditory digit span, flanker test, Penn Emotional Acuity Test; social cognition test: Penn Emotional Acuity Test	26 placebo 19 *verum*	4
Chandrasekhar et al. (2012)	32+32	DBRPCT	8.5	300-mg capsules NS, 5% withanolides, KSM-66	Stress; score: <5 (WHO-5), <14 (PSS)	Serum cortisol, PSS, DASS, GHQ-28 questionnaires	6 placebo 5 *verum*	2
Cooley et al. (2009)	40+41	R, C, PC	12	300-mg pills NS, 1.5% withanolides, ISRCTN78958974	Anxiety	BAI, SF-36, FSI, MY-MOP	7+7	1
Andrade et al. (2000)	20+19	DBRPCT	6	500 mg, 250-mg tablets ethanol, NS	Generalized anxiety, mixed anxiety, depression	Hamilton anxiety score, global rating scale, SAFTEE symptom checklist	17 placebo 16 *verum*	3

TABLE 8–3. Summary of clinical trials: *Withania somnifera* in anxiety and bipolar disorders *(continued)*

Author, year	Sample size	Design	Duration, weeks	Daily dose, extraction solvent, marker, trade name	Diagnosis	Primary outcomes	Adverse events (*n*)	Jadad score[b]
Auddy et al. (2008)	30+35+ 35+30	DBRPCT	8.5	125-mg and 250-mg capsules NS, 11.9% withanolides, 1% withaferin A, Sensoril	Anxiety	Modified HARS, serum cortisol, serum C-reactive protein, pulse rate and blood pressure	0	3
Singy and Malviya (1978)	30	OL	4	40 mL fermented water extract corresponding to 12 g dry roots	Anxiety, neurosis	Anxiety: nervousness, palpitation, tremors, headache, anorexia, insomnia, lack of concentration, dyspepsia, fatigue, irritability	NS	0

[a]Two publications are related to the same study: NCT00761761.
[b]Jadad AR, Moore RA, Carroll D, et al.: "Assessing the Quality of Reports of Randomized Clinical Trials: Is Blinding Necessary?" *Controlled Clinical Trials* 17:1–12, 1996.
Note. BAI=Beck Anxiety Inventory; C=positive control, psychotherapy intervention; DASS=Depression Anxiety Stress Scales; DBRPCT=double-blind, randomized placebo-controlled trial; FSI=Fatigue Symptom Inventory; GHQ-28=28-Item General Health Questionnaire; HARS=Hamilton Anxiety Rating Scale; MADRS=Montgomery-Åsberg Depression Rating Scale; MY-MOP=Measure Yourself Medical Outcomes Profile; NS=not specified; OL=open-label; PC=placebo-controlled; PSS=Perceived Stress Scale; R=randomized; SAFTEE=Systematic Assessment for Treatment Emergent Events technique scale; SF=Short Form 36; WHO-5=WHO (Five) Well-Being Index; YMRS=Young Mania Rating Scale.

Rhodiola rosea

Rhodiola rosea is an ancient medicinal herb that grows at high altitudes in Europe and Asia. The roots and rhizomes are used to make tea, alcohol extracts, and water extracts, which are used to either promote physical fortitude or reduce mental strain during conditions of stress, including physical exertion, military training and warfare, cold stress, low oxygen, bacterial and viral infections, wound healing, mental performance, cognitive demands, cancer, athletic competition, and space travel (Cuerrier and Ampong-Nyarko 2014).

Effects of *Rhodiola rosea* on Asthenia, Fatigue, and Exhaustion

In 1969, the Pharmacological Committee of the Ministry of Health of the former Soviet Union recommended *R. rosea* extract for the treatment of neuropsychiatric disorders including "asthenic syndrome," "neurosis," "vascular dystonia," hypotension, and "schizophrenia asthenic type" (Rhizome and roots of Rhodiola rosea 1999). Asthenic syndrome, characterized by general weakness, reduced work capacity, poor concentration and memory, irritability, headache, insomnia, and anorexia, usually occurs after prolonged intensive work requiring high levels of mental exertion. *R. rosea* extract was found to improve performance and shorten recovery time in essentially healthy individuals when given during heavy physical or mental work (Krasik et al. 1970). It also protected individuals prone to asthenia from the adverse effects of intensive workloads. Other uncontrolled, open-label studies suggested that *R. rosea* extract also may be effective in reducing psychogenic and somatogenic exhaustion caused by CFS and infectious diseases. For example, individuals suffering from post-influenza fatigue showed improved mental and physical work capacity and concentration ability and a reduction in headaches after 3 days of *R. rosea* extract therapy (Krasik et al. 1970; Saratikov and Krasnov 2004).

Effects of *Rhodiola rosea* on Cognitive Function

In an early observational study, *R. rosea* extract in combination with piracetam, a synthetic *nootropic* (brain function enhancer), improved cognitive function, memory, and affect in individuals with organic amnestic disorders, such as Korsakoff syndrome, dementias, intellectual disability, and acquired organic mental syndromes (Sudakov et al. 1986). Limited studies in subjects with cognitive impairment showed improvement in cognitive deficits: forgetfulness, memory loss, and impaired concentration. Randomized controlled trials (RCTs) in healthy adults found that *R. rosea* standardized extract alone and in combination with *E. senticosus* and *S. chinensis* (ADAPT formula) improved intellectual performance on complex tasks, short-term memory, concentration, learning, and performance under stress (Aslanyan et al. 2010).

Studies of *Rhodiola rosea* for Treatment of Anxiety and Depression

In an open-label study (Bystritsky et al. 2008), *R. rosea* (Rhodax) 340 mg given daily for 10 weeks to 10 subjects with DSM-IV (American Psychiatric Association 1994)

generalized anxiety disorder (Table 8–2) significantly reduced the mean Hamilton Anxiety Rating Scale score ($P=0.01$) and Hamilton Rating Scale for Depression (Ham-D) score ($P=0.001$) at endpoint.

An observational study reported that *R. rosea* used as an adjunctive treatment for depression may have enhanced antidepressant efficacy and may reduce side effects of tricyclic antidepressants in depressed patients. More recently, Darbinyan et al. (2007) conducted a randomized double-blind, placebo-controlled trial of *R. rosea* extract (SHR-5; Swedish Herbal Institute, Vallberga, Halland, Sweden) in 89 subjects ages 18–70 years with mild to moderate DSM-IV-classified major depressive disorder. Subjects received either *R. rosea* extract 340 mg daily ($n=31$), *R. rosea* extract 680 mg daily ($n=29$), or placebo ($n=29$) for 6 weeks. At endpoint, mean Ham-D score significantly declined for both doses of *R. rosea* ($P<0.0001$) versus no significant reduction in Ham-D score for placebo ($P=0.2206$). Intergroup analysis found lower mean Ham-D scores for both *R. rosea* treatment conditions versus placebo ($P<0.001$, $P<0.001$, respectively) (Panossian and Wikman 2014). However, such dramatic drug-placebo differences in an underpowered pilot study are questionable. These promising results need to be replicated in larger RCTs.

Subsequently, Mao et al. (2015) performed a 12-week RCT of *R. rosea* extract versus sertraline or placebo in 57 adults with mild to moderate major depressive disorder (DSM-IV), baseline Ham-D score≥10. Identically appearing capsules containing either pharmaceutical-grade *R. rosea* (SHR-5) powdered extract 340 mg (rosavins 3.07%, rhodioloside [salidroside] 1.95%) (Swedish Herbal Institute, Vallberga, Halland, Sweden), sertraline 50 mg HCl (North Star Pharmaceuticals, Memphis, Tennessee), or placebo (lactose monohydrate NF; Spectrum Quality Products, New Brunswick, New Jersey) were dispensed under the aegis of the U.S. Food and Drug Administration (investigational new drug 105,063). Starting with one capsule daily for 2 weeks, subjects with ≤50% reduction in Ham-D score (versus baseline) had the dose increased to two capsules daily during weeks 3 and 4 of therapy. This continued every 2 weeks up to a maximum of four capsules daily during weeks 6–12. Subjects unable to tolerate increased drug doses had their dosage reduced to a minimum of one capsule daily. Measurements were obtained at baseline and after 2, 4, 6, 8, and 12 weeks. The study was powered to detect relatively large differences between treatment conditions and to identify trends in the data that might inform future study design. There was no statistically significant difference in change over time on Ham-D among treatment groups ($P=0.79$), and the decline in Ham-D scores by week 12 was slightly greater for sertraline (–8.2; 95% confidence interval [CI] –12.7 to –3.6) versus *R. rosea* (–5.1; 95% CI –8.8 to –1.3) and placebo (–4.6; 95% CI –8.0 to –0.6). Nevertheless, there were clinically meaningful odds ratios (95% CI) of global improvement by week 12 (versus placebo) of 1.39 (0.38–5.04) for *R. rosea* and 1.90 (0.44–8.20) for sertraline, indicating that subjects taking *R. rosea* had 1.4 times the odds of improvement, and subjects taking sertraline had 1.9 times the odds of improvement versus placebo. More subjects who were taking sertraline (63.2%) versus *R. rosea* (30.0%) or placebo (16.7%) reported adverse events ($P=0.012$). Two subjects prematurely discontinued sertraline; none discontinued *R. rosea* or placebo.

Overall, these studies suggest a possible antidepressant action for *R. rosea* in adults.

Safety and Drug Interactions

In comparison with most conventional antidepressants, *R. rosea* is well tolerated in short-term studies and shows a favorable safety profile (Amsterdam and Panossian 2016). In 31 patients with schizophrenia, high doses of *R. rosea* extract (25–40 drops twice daily) for 4–6 weeks reduced neuroleptic-induced extrapyramidal side effects. A similar reduction in extrapyramidal symptoms was also observed in 9 patients with schizophrenia in whom an anticholinergic drug was ineffective (Krasik et al. 1970).

R. rosea ethanolic extract inhibits cytochrome P450 (CYP) 3A4 in isolated Caco-2 cells in vitro (Hellum et al. 2010), suggesting that it may suppress metabolic transformation of warfarin or other drugs in the liver, increasing serum concentration of such drugs. However, these effects on the activity of isolated enzymes do not appear to have clinical significance because concomitant treatment of rats with warfarin or theophylline and SHR-5 brand of *R. rosea* did not give rise to significant effects on the pharmacokinetics of warfarin or theophylline. Simultaneous administration of SHR-5 and warfarin did not alter significantly the pharmacokinetics or the anticoagulant activity of warfarin. It was concluded that interaction of *Rhodiola* with coadministered drugs is likely to be negligible (Panossian et al. 2009).

Schisandra chinensis

Schisandra chinensis, known in traditional Chinese medicine as bei wu wei zi or gomischi, is a woody vine that grows naturally in northern China, eastern Russia, Korea, and Japan. Its berries and seeds have been used for millennia to increase strength, energy, and stress resilience. Extracts of *S. chinensis* show antidepressant and anti-stress properties (for a review, see Panossian and Wikman 2008).

Effects of *Schisandra chinensis* on Asthenia

An open case study of more than 250 individuals found that taking *S. chinensis* extract for 2–10 weeks reduced symptoms of “asthenia,” “exhaustion,” and poor physical and mental work performance. A nonrandomized open-label comparative study of 95 subjects with “neurasthenia” syndrome reported that virtually all subjects receiving *S. chinensis* seed tincture had a substantial reduction in generalized weakness, insomnia, anorexia, irritability, and headaches versus 55% of the control group not receiving *S. chinensis.* These open-label, uncontrolled studies are subject to bias and require validation by RCTs (Panossian and Wikman 2005, 2008, 2010).

Effects of *Schisandra chinensis* on Depression and Other Neuropsychiatric Disorders

Studies using older diagnostic categories and methodologies have reported a beneficial effect of *S. chinensis* extract on “asthenic” and depressive states. For example, Leman (1952) observed that *S. chinensis* administration for up to 40 days in 40 individuals with asthenic and “psychogenic” or “somatic” depression led to improvement in

mood, energy, activity, insomnia, and anorexia. *S. chinensis* was well tolerated, with a stimulatory effect less troubling than either caffeine or amphetamine. Another uncontrolled, open-label study (Galant 1957) reported better recovery from psychosis following *S. chinensis* treatment in 19 patients with schizophrenia, 6 patients with "reactive psychosis," 4 patients with "alcoholic psychosis," and 3 patients with psychotic depression. A subsequent uncontrolled, open-label study of *S. chinensis* in 41 subjects with schizophrenia and 197 subjects with chronic alcoholism (Romas 1967) showed that *S. chinensis* tincture (5–25 drops per day) helped patients become calm, sociable, active, undepressed, free of anxiety, and willing to work. These early open studies suggest a possible role for *S. chinensis* in the treatment of psychosis. Considering the adverse effects of chronic medications used to treat psychoses, these preliminary observations warrant future controlled study. Adjunctive administration of *S. chinensis* with conventional neuroleptic or antidepressant medications appeared to reduce the side-effect burden (e.g., anticholinergic effects) of synthetic drugs. None of these studies were rigorously controlled or randomized, and the results need to be validated using modern methodologies.

Safety and Adverse Reactions

Overall, *S. chinensis* has a favorable safety profile; however, it can stimulate uterine contractions and should be avoided during pregnancy. Other possible side effects include heartburn, upset stomach, decreased appetite, stomach pain, skin rash, urticaria, and itching. *S. chinensis* may affect serum levels of medications metabolized by CYP3A4 isozymes, such as warfarin. However, in vivo and human studies have not shown clinically significant effects on serum levels of medications metabolized by the same enzymes. Such discrepancies are not uncommon when herbal extracts are tested in vitro.

Eleutherococcus senticosus

Eleutherococcus (Acanthopanax) senticosus, or ben cao gang mu in traditional Chinese medicine, is a thorny shrub that is widely used in Russia and China to increase performance and quality of life and to treat respiratory infections and flu. It is sometimes called Siberian ginseng, although it does not belong to the ginseng family. Studies of extracts from *E. senticosus* roots show antistress, antiulcer, anti-irradiation, anticancer, anti-inflammatory, and hepatoprotective activity (Panossian 2003; Panossian and Wikman 2010).

Therapeutic Effect of *Eleutherococcus senticosus* in Chronic Fatigue Syndrome

Ben Cao Gang Mu (An Outline of Herbs), written by Li Shi Zhen in 1596, describes the use of *E. senticosus* root for promoting vigor, vitality, and longevity (Weng et al. 2007). In one recent study (Hartz et al. 2004), an extract of *E. senticosus* (standardized to a total of 0.12% eleutherosides B and E) 2,000 mg daily was given to subjects with CFS. The bo-

tanical preparation was effective after 2 months; however, by 4 months the efficacy was not significantly different from that of placebo. In China, standardized *E. senticosus* extracts (0.82% eleutherosides B and E) are approved for treatment of insomnia, weakness, lack of appetite, and muscle soreness—symptoms associated with depression and CFS (Weng et al. 2001). Another study found no statistically significant difference between improvements with the *E. senticosus* extract versus imipramine in subjects with moderate depression (Weng et al. 2001). In numerous studies, *E. senticosus* was examined in the combination formula, with *R. rosea* rhizome and *S. chinensis* berry extracts (ADAPT) (e.g., Aslanyan et al. 2010; Narimanian et al. 2005; Panossian et al. 2012, 2013).

Effects of *Eleutherococcus senticosus* in Bipolar Disorder

A 6-week RCT compared the safety and efficacy of *E. senticosus* extract plus lithium with fluoxetine plus lithium for bipolar depression in 76 adolescents (45 females and 31 males), ages 12–17 (Weng et al. (2007). Outcomes were assessed using the Ham-D and Young Mania Rating Scale (YMRS) at baseline and after weeks 1, 2, 4, and 6. Response was defined as >50% reduction in baseline Ham-D score, remission as Ham-D score <7, and mania as an increase in YMRS score >16 for at least 7 days at any time during treatment. Subjects were queried regarding adverse events. Response rates were comparable between adjunctive *E. senticosus* and fluoxetine (67.6% vs. 71.8%). Remission rates between groups were also similar (51.4% vs. 48.7%). During treatment, there was a significant time effect (F=183.06; P<0.01) but not a significant group effect (F=0.99) or effect by duration of treatment interaction (F=0.779). Three subjects in the fluoxetine group versus no subjects in the *E. senticosus* group experienced a manic switch episode. A greater frequency of adverse events was reported with fluoxetine versus *E. senticosus*. For adolescents with bipolar disorder, *E. senticosus* is a promising adjunctive treatment (Weng et al. 2007). Studies of *E. senticosus* with mood stabilizers other than lithium are warranted.

Safety and Adverse Reactions

E. senticosus generally produces few side effects and is usually well tolerated. The contraindication of arterial hypertension is not evidence based and should be carefully reevaluated (Schmidt et al. 2014). Safety during pregnancy or breast-feeding has not been established.

Withania somnifera

Withania somnifera (L.) Dunal (*W. somnifera*; Solanaceae), known as ashwagandha in Sanskrit and as Indian ginseng (Kulkarni and Dhir 2008), is used both in Ayurveda and Unani traditional medicinal systems for treating numerous conditions, including neurological and mental disorders. *W. somnifera* has more than 30 steroidal lactone withanolides; withaferin A is used for standardization of extracts (Lavie et al. 1965).

Other phytosterols (sitoindosides), alkaloids, saponins, flavonoids, and other phenolic compounds also contribute to the herb's broad spectrum of activities (Choudhary et al. 2013).

Withania somnifera for Stress, Anxiety, Insomnia, and Depression

Anxiolytic-like and antidepressant-like activity of the alcohol root extract and isolated glycowithanolides, sitoindosides, and withaferin A has been demonstrated in animal models of depression and anxiety (Bhattacharya and Muruganandam 2003; Bhattacharya et al. 2000; Singh et al. 1982). *W. somnifera* root extract produced sedation, reduction in locomotor activity, potentiation of thiopental-induced sleep, reduction of catecholamines and acetylcholine with an increase in histamine and serotonin in brain tissue of mice, and a delay in semicarbazide- and pentylenetetrazole-induced seizures (Day and Chatterjee 1968; Singh et al. 1979; Yidya Prabhu and Rao 1990). It is possible that GABAergic activity is the mechanism for improving GABAergic signaling dysfunctions such as in anxiety disorders and insomnia (Candelario et al. 2015).

Seven clinical studies have described *W. somnifera* as a safe and effective adaptogen for treating stress-related neuropsychiatric conditions—anxiety and bipolar disorder (Tables 8–1 and 8–3). For example, in a double-blind, randomized placebo-controlled trial, 64 adult subjects exposed to chronic stress were randomly assigned to take either one capsule twice daily of *W. somnifera* extract 300 mg or placebo for 60 days (Chandrasekhar et al. 2012). Telephone interviews assessed safety and compliance on days 15, 30, and 45 and at endpoint day 60. At study endpoint, subjects taking *W. somnifera* extract showed significantly greater reductions on all stress scales versus placebo ($P<0.0001$) and a significantly greater reduction in serum cortisol levels versus placebo ($P=0.0006$). Adverse event rates were comparable in both groups.

Table 8–3 shows anxiolytic effects of five different *Withania* preparations from five clinical studies with a total of 157 patients. Three of these were double-blind, randomized placebo-controlled trials with quality scores 2–3 out of 5. Study limitations include insufficient details regarding extract preparation, treatment administration, and randomization procedures. The best study (quality score 4), an 8-week double-blind RCT, used a water extract, 250–500 mg/day *Withania* (Sensoril), in 60 subjects with bipolar disorder (Chengappa et al. 2013). Compared with the control subjects, subjects taking *W. somnifera* had significantly better performance on three cognitive tasks: digit span backward ($P=0.035$), flanker neutral response time ($P=0.033$), and social cognition response of the Penn Emotional Acuity Test ($P=0.045$). Other cognitive tests were not significantly different between groups. Adverse events were similar in both groups. These encouraging results need confirmation in clinical trials of standardized products.

Safety, Adverse Events, and Contraindications

W. somnifera has a benign side-effect profile. Compared with other adaptogens, it is more calming. *Withania* is contraindicated in pregnancy because it may precipitate abortion. A 2010 review noted the potential for additive effects if given with sedatives

(Brinker 2010). The presence of *W. somnifera* may cause false positive readings for digoxin levels in fluorescence polarization immunoassay.

Limitations

An important limitation in assessment of herbal studies in general is variability in the composition and activity of bioactive constituents due to genetic and environmental (e.g., climate, soil characteristics, plant infections, fertilization) factors. Differences in processing raw materials, extraction, purification, and storage also affect the final composition and activity. Batch-to-batch reproducibility is not always evaluated. Heterogeneity in results among clinical studies may be due in part to the use of nonidentical herbal preparations. Of note, the dose-effect curve of adaptogens is bell-shaped in animal studies, such that excessively high doses are not more effective (Kurkin et al. 2003; Panossian and Wagner 2005; Wiegant et al. 2009). This may be due to feedback regulation of signaling systems and/or differences in threshold concentration for binding of extract constituents to receptors.

KEY POINTS

- Adaptogens demonstrate multi-target effects on the regulation of cellular responses to stress by influencing neuroendocrine, neurotransmitter, receptor, and molecular networks associated with beneficial effects on mood and cognitive function.
- Adaptogens may be better tolerated than conventional psychotropics; however, this needs confirmation in controlled comparative trials.
- Pilot clinical trials show therapeutic benefits of adaptogens for depression and anxiety, as well as cognitive and memory impairment.
- Further large-scale studies of standardized products are indicated for exploration of numerous potential clinical applications.

References

Abidov M, Crendal F, Grachev S, et al: Ziegenfuss T. Effect of extracts from Rhodiola rosea and Rhodiola crenulata (Crassulaceae) roots on ATP content in mitochondria of skeletal muscles. Bull Exp Biol Med 136(6):585–587, 2003 15500079

Agnihotri AP, Sontakke SD, Thawani VR, et al: Effects of Withania somnifera in patients of schizophrenia: a randomized, double blind, placebo controlled pilot trial study. Indian J Pharmacol 45(4):417–418, 2013 24014929

American Psychiatric Association: Diagnostic and Statistical Manual of Mental Disorders, 4th Edition. Washington, DC, American Psychiatric Association, 1994

Amsterdam JD, Panossian AG: Rhodiola rosea L. as a putative botanical antidepressant. Phytomedicine 23(7):770–783, 2016 27013349

Andrade C, Aswath A, Chaturvedi SK, et al: A double-blind, placebo-controlled evaluation of the anxiolytic efficacy of an ethanolic extract of Withania somnifera. Indian J Psychiatry 42(3):295–301, 2000 21407960

Asea A, Kaur P, Panossian A, Wikman KG: Evaluation of molecular chaperons Hsp72 and neuropeptide Y as characteristic markers of adaptogenic activity of plant extracts. Phytomedicine 20(14):1323–1329, 2013 23920279

Aslanyan G, Amroyan E, Gabrielyan E, et al: Double-blind, placebo-controlled, randomised study of single dose effects of ADAPT-232 on cognitive functions. Phytomedicine 17(7):494–499, 2010 20374974

Auddy B, Hazra J, Mitra A, et al: A standardized Withania somnifera extract significantly reduces stress related parameters in chronically stressed humans: a double blind, randomized, placebo controlled study. Journal of American Nutraceutical Association 11:50–56, 2008

Bhattacharya SK, Muruganandam AV: Adaptogenic activity of Withania somnifera: An experimental study using a rat model of chronic stress. Pharmacol Biochem Behav 75(3):547–555, 2003 12895672

Bhattacharya SK, Bhattacharya A, Sairam K, Ghosal S: Anxiolytic-antidepressant activity of Withania somnifera glycowithanolides: an experimental study. Phytomedicine 7(6):463–469, 2000 11194174

Brekhman II, Dardymov IV: New substances of plant origin which increase non-specific resistance. Ann Rev Pharmacol 9:419–430, 1969 4892434

Brinker F: Herbal Contraindications and Drug Interactions Plus Herbal Adjuncts With Medicines. Sandy, OR, Eclectic Medical Publishers, 2010

Brown RP, Gerbarg PL, Ramazanov Z, et al: A phytomedical review of Rhodiola rosea. Herbalgram 56:40–62, 2002

Bystritsky A, Kerwin L, Feusner JD: A pilot study of Rhodiola rosea (Rhodax®) for generalized anxiety disorder (GAD). J Altern Complement Med 14(2):175–180, 2008 18307390

Candelario M, Cuellar E, Reyes-Ruiz JM, et al: Direct evidence for GABAergic activity of Withania somnifera on mammalian ionotropic GABAA and GABAp receptors. J Ethnopharmacol 171:264–272, 2015 26068424

Chandrasekhar K, Kapoor J, Anishetty S: A prospective, randomized double-blind, placebo-controlled study of safety and efficacy of a high-concentration full-spectrum extract of ashwagandha root in reducing stress and anxiety in adults. Indian J Psychol Med 34(3):255–262, 2012 23439798

Chengappa KN, Bowie CR, Schlicht PJ, et al: Randomized placebo-controlled adjunctive study of an extract of Withania somnifera for cognitive dysfunction in bipolar disorder. J Clin Psychiatry 74(11):1076–1083, 2013 24330893

Choudhary MI, Yousuf S, Rahman AU: Chemistry and antitumor activity., in Natural Products. Edited by Ramawat KG, Merillon JM. Berlin, Springer-Verlag, 2013, pp 3465–3495

Cooley K, Szczurko O, Perri D, et al: Naturopathic care for anxiety: a randomized controlled trial ISRCTN78958974. PLoS One 4(8):e6628, 2009 19718255

Cuerrier A, Ampong-Nyarko K: *Rhodiola rosea* (Traditional Herbal Medicines for Modern Times). Boca Raton, FL, CRC Press, 2014, pp 203–221

Darbinyan V, Aslanyan G, Amroyan E, et al: Clinical trial of Rhodiola rosea L. extract SHR-5 in the treatment of mild to moderate depression. Nord J Psychiatry 61(5):343–348, 2007 17990195

Day PK, Chatterjee BK: Studies on the neuropharmacological properties of several Indian medicinal plants. J Res Indian Med 3(1):9–17, 1968

Edwards D, Heufelder A, Zimmermann A, et al: Therapeutic effects and safety of Rhodiola rosea extract WS® 1375 in subjects with life-stress symptoms—results of an open-label study. Phytother Res 26(8):1220–1225, 2012 22228617

Fintelmann V, Gurenwald J: Efficacy and tolerability of a Rhodiola rosea extract in adults with physical and cognitive deficiencies. Adv Ther 24(4):929–939, 2007 17901042

Galant IB, Kuznetsova AI, Suvorina NA, et al: The experience in using Schizandra chinensis in psychiatric practice, in Clinical Development and Therapy of Mental Diseases and Organisation of Psychoneurological Assistance. Moscow, State Research Institute of Psychiatry of the Ministry of Health of RSFSR, 1957, pp 112–113

Gannon JM, Forrest PE, Roy Chengappa KN: Subtle changes in thyroid indices during a placebo-controlled study of an extract of Withania somnifera in persons with bipolar disorder. J Ayurveda Integr Med 5(4):241–245, 2014 25624699

Hartz AJ, Bentler S, Noyes R, et al: Randomized controlled trial of Siberian ginseng for chronic fatigue. Psychol Med 34(1):51–61, 2004 14971626

Hirsch D, Zukowska Z: NPY and stress 30 years later: the peripheral view. Cell Mol Neurobiol 32(5):645–659, 2012 22271177

Hellum BH, Tosse A, Hoybakk K, et al: Potent in vitro inhibition of CYP3A4 and P-glycoprotein by Rhodiola rosea. Planta Med 76(4):331–338, 2010 19790032

Krasik ED, Morozova ES, Petrova KP: Therapy of asthenic conditions: clinical perspectives of applica-tion of Rhodiola rosea extract (golden root), in Proceedings Modern Problems in Psycho-pharmacology. Edited by Avrutskiy GY. Kemerovo City, USSR, Siberian Branch of Russian Academy of Sciences, 1970, pp 298–330

Kulkarni SK, Dhir A: Withania somnifera: an Indian ginseng. Prog Neuropsychopharmacol Biol Psychiatry 32(5):1093–1105, 2008 17959291

Kurkin VA, Zapesochnaya GG: Chemical composition and pharmacological properties of Rhodiola rosea. Journal of Medicinal Plants 20(10):1231–1244, 1986

Kurkin VA, Dubishchev AV, Titova IN, et al: Potent in vitro inhibition of CYP3A4 and P-glycoprotein by Rhodiola rosea. Rastit Resursi 3:115–122, 2003

Lavie D, Glotter E, Shvo Y: Constituents of Withania somnifera dun—part IV: the structure of Withaferin A. J Chem Soc 30:7517–7531, 1965

Leman MF: Treatment of reactive and asthenic states of exogenous etiology using the Far East Schizandra. Zh Nevropatol Psikhiatr Im S S Korsakova 52:67–70, 1952

Mao JJ, Lie QS, Soeller J, et al: Rhodiola rosea therapy for major depressive disorder: a study protocol for a randomized, double-blind, placebo-controlled trial. J Clin Trials 4:170, 2014 25610752

Mao JJ, Xie SX, Zee J, et al: Rhodiola rosea versus sertraline for major depressive disorder: a randomized placebo-controlled trial. Phytomedicine 22(3):394–399, 2015 25837277

Mashkovskiy MD: Extractum Eleutherococci fluidum, in Medicinal Agents (Drug Index) Manual for Doctors, Vol 1 [in Russian]. Moscow, Novaya Volna, 2000, pp 133–136

Narimanian M, Badalyan M, Panosyan V, et al: Impact of Chisan (ADAPT-232) on the quality-of-life and its efficacy as an adjuvant in the treatment of acute non-specific pneumonia. Phytomedicine 12(10):723–729, 2005 16323290

Olsson EM, von Schéele B, Panossian AG: A randomised, double-blind, placebo-controlled, parallel-group study of the standardised extract shr-5 of the roots of Rhodiola rosea in the treatment of subjects with stress-related fatigue. Planta Med 75(2):105–112, 2009 19016404

Panossian AG: Adaptogens: Tonic herbs for fatigue and stress. Alternative & Complementary Therapies 9(6):327–331, 2003

Panossian AG: Adaptogens in mental and behavioral disorders. Psychiatr Clin North Am 36(1):49–64, 2013 23538076

Panossian A, Wagner H: Stimulating effect of adaptogens: an overview with particular reference to their efficacy following single dose administration. Phytother Res 19(10):819–838, 2005 16261511

Panossian A, Wikman G: Effect of adaptogens on the central nervous system. Arq Bras Fitomed Cient 2:108–130, 2005

Panossian AG, Wikman G: Pharmacology of Schisandra chinensis Bail.: an overview of Russian research and uses in medicine. J Ethnopharmacol 118(2):183–212, 2008 18515024

Panossian A, Wikman G: Effects of adaptogens on the central nervous system and the molecular mechanisms associated with their stress-protective activity. Pharmaceuticals (Basel) 3(1):188–224, 2010

Panossian A, Wikman G: Evidence based efficacy and effectiveness of Rhodiola SHR-5 extract in treating stress- and age-associated disorders, in Rhodiola Rosea. Edited by Cuerrier C, Ampong-Nyarko K. Boca Raton, CRC Press, 2014, pp 203–221

Panossian A, Wikman G, Wagner H: Plant adaptogens: new concepts on their mode of action. Phytomedicine 6:287–300, 1999

Panossian A, Hovhannisyan A, Abrahamyan H, et al: Pharmacokinetic and pharmacodynamic study of interaction of Rhodiola rosea SHR-5 extract with warfarin and theophylline in rats. Phytother Res 23(3):351–357, 2009 18844284

Panossian A, Wikman G, Sarris J, et al: Rosenroot (Rhodiola rosea): traditional use, chemical composition, pharmacology and clinical efficacy. Phytomedicine 17(7):481–493, 2010 20378318

Panossian A, Wikman G, Kaur P, et al: Adaptogens stimulate neuropeptide Y and Hsp72 expression and release in neuroglia cells. Front Neurosci 6:6, 2012 22347152

Panossian A, Hamm R, Kadioglu O, et al: Synergy and antagonism of active constituents of ADAPT-232 on transcriptional level of metabolic regulation of isolated neuroglial cells. Front Neurosci 7:16, 2013 23430930

Panossian A, Hamm R, Wikman G, et al: Mechanism of action of Rhodiola, salidroside, tyrosol and triandrin in isolated neuroglial cells: an interactive pathway analysis of the downstream effects using RNA microarray data. Phytomedicine 21(11):1325–1348, 2014 25172797

Rao RP, Yuan C, Allegood JC, et al: Ziegenfuss T. Ceramide transfer protein function is essential for normal oxidative stress response and lifespan. Proc Natl Acad Sci USA 104(27):11,364–11,369, 2007 17592126

Rhizome and roots of Rhodiola rosea, in National Pharmacopoeia of the USSR, 11th Edition, Vol 2, Pharmacopoeia Paper 75, Update 2, Moscow, USSR Ministry of Health, 1999, pp 317–319

Romas RS: About the effect of Schizandra chinensis on higher brain structures of schizophrenia patients and chronic alcoholics. Thesis, Faculty of Medicine, Pirogov's Medical Institute, Vinnitsa, USSR, 1967

Saratikov AS, Krasnov EA: Rhodiola Rosea (Golden Root), 4th Edition. Tomsk, Russia, Tomsk State University Publishing House, 2004

Schmidt M, Thomsen M, Kelber O, et al: Myths and facts in herbal medicines: Eleutherococcus senticosus (Siberian ginseng) and its contraindication in hypertensive patients. Botanics: Targets and Therapy 4:27–32, 2014

Singh N, Nath R, Lata A, et al: Withania somnifera (Ashwagandha), a rejuvenating herbal drug which enhances survival during stress (an adaptogen). Int J Crude Drug Res 20(1):29–35, 1982

Singh RH, Malviya PC: Studies on the psychotropic effect of an indigenous rasayana drug, Asvagandha (Withania somnifera dunol) part I: (clinical studies). J Res Educ Indian Med 13(1):15–24, 1978

Singh RH, Malviya PC, Sarkar FH, et al: Studies on the psychotropic effect of an indigenous rasayana drug, Asvagandha (Withania somnifera dunol) part II: (experimental studies). J Res Educ Indian Med 14(1):49–54, 1979

Sudakov VN, Savinykh AB, Agapov YK: The role of adaptogens in the psychoprophylaxis of patients with borderline states of exogenous-organic genesis, in Modern Problems of Pharmacology and Search for New Medicines, Vol 2. Edited by Goldsberg ED. Tomsk, Russia, Tomsk State University Press, 1986, pp 298–330

Udintsev SN, Schakhov VP: Decrease of cyclophosphamide haematotoxicity by Rhodiola rosea root extract in mice with Ehrlich and Lewis transplantable tumors. Prog Neuropsychopharmacol Eur J Cancer 27(9):1182, 1991 1835634

Weng S, Cheng Z, Wang H, et al: An open-label study of capsule Acanthopanax senticosus for treatment of depression in patients. Hubei Journal of Chinese Traditional Medicine 6:8–9, 2001

Weng S, Tang J, Wang G, et al: Comparison of the addition of Siberian ginseng (Acanthopanax senticosus) versus fluoxetine to lithium for the treatment of bipolar disorder in adolescents: a randomized, double-blind trial. Curr Ther Res Clin Exp 68(4):280–290, 2007 24683218

Wiegant FAC, Surinova S, Ytsma E, et al: Plant adaptogens increase lifespan and stress resistance in C. elegans. Biogerontology 10(1):27–42, 2009 18536978

Yidya Prabhu M, Rao A: Neuropharmacological activity of Withania somnifera. Fitoterapia 66(3):237–240, 1990

Active principles – alkaloids withasomnine, somniferine, somniferinine, somnine.

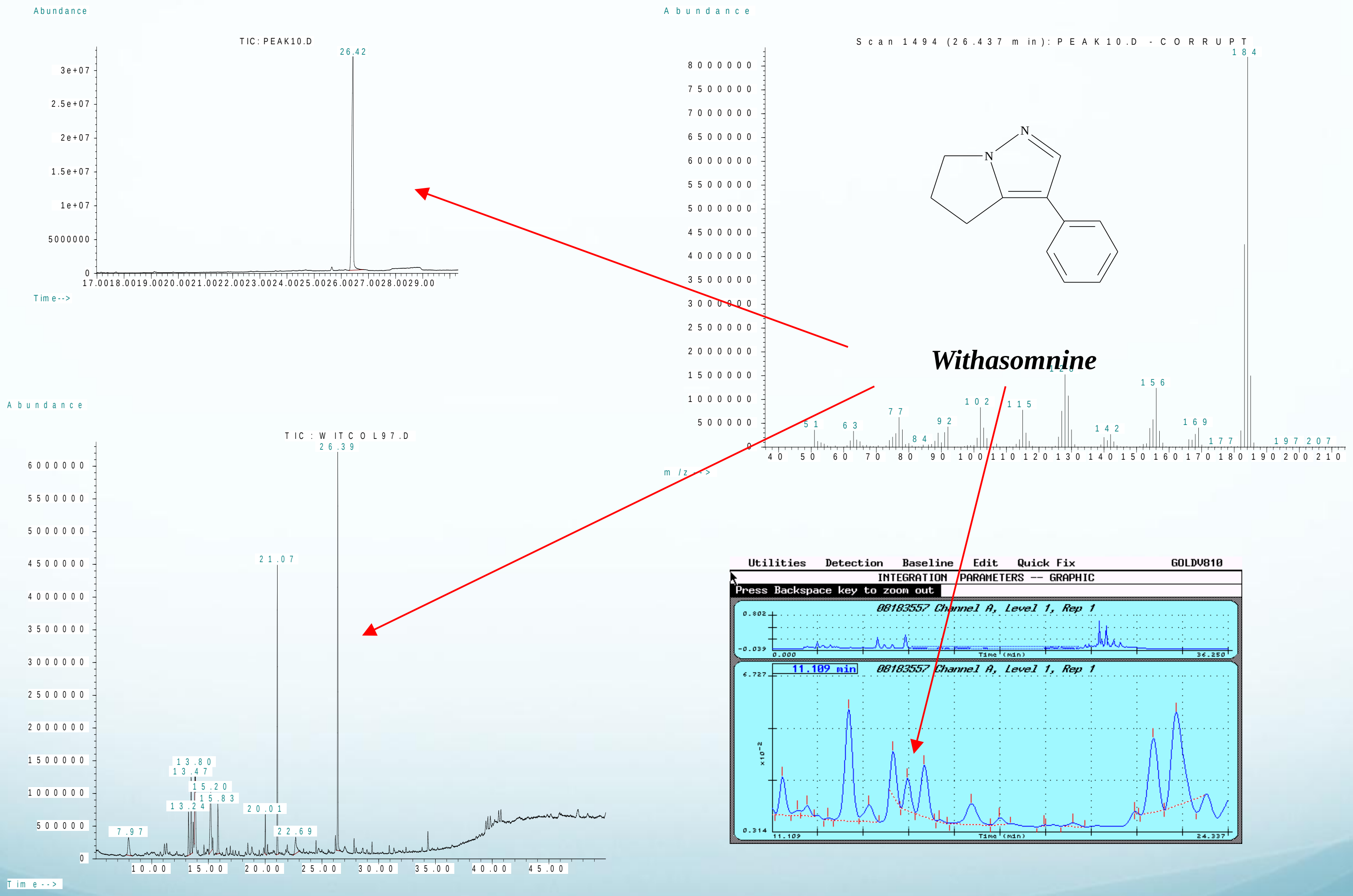

Supplement: Supplementary file 1 [file pharmaceuticals-18-01346-s001.zip › Supplement S2_Withasomnin..pdf]
