# Supplementary material for: Two Sides of the Same Coin for Health: Adaptogenic Botanicals as Nutraceuticals for Nutrition and Pharmaceuticals in Medicine
Source: Pharmaceuticals (Basel). 2025 Sep 8;18(9):1346. doi: 10.3390/ph18091346 (PMC12472958; doi:10.3390/ph18091346)
Supplement: Supplementary file 1 [file pharmaceuticals-18-01346-s001.zip › Supplement S3-Panossian Rhodiola 2023 FINAL Presentation.pdf]

HMPPA and GA e-Symposium"RHODIOLA ROSEA - PHYTOCHEMICAL, PHARMACOLOGICAL AND CLINICAL EVIDENCE". NOVEMBER 23, 2023

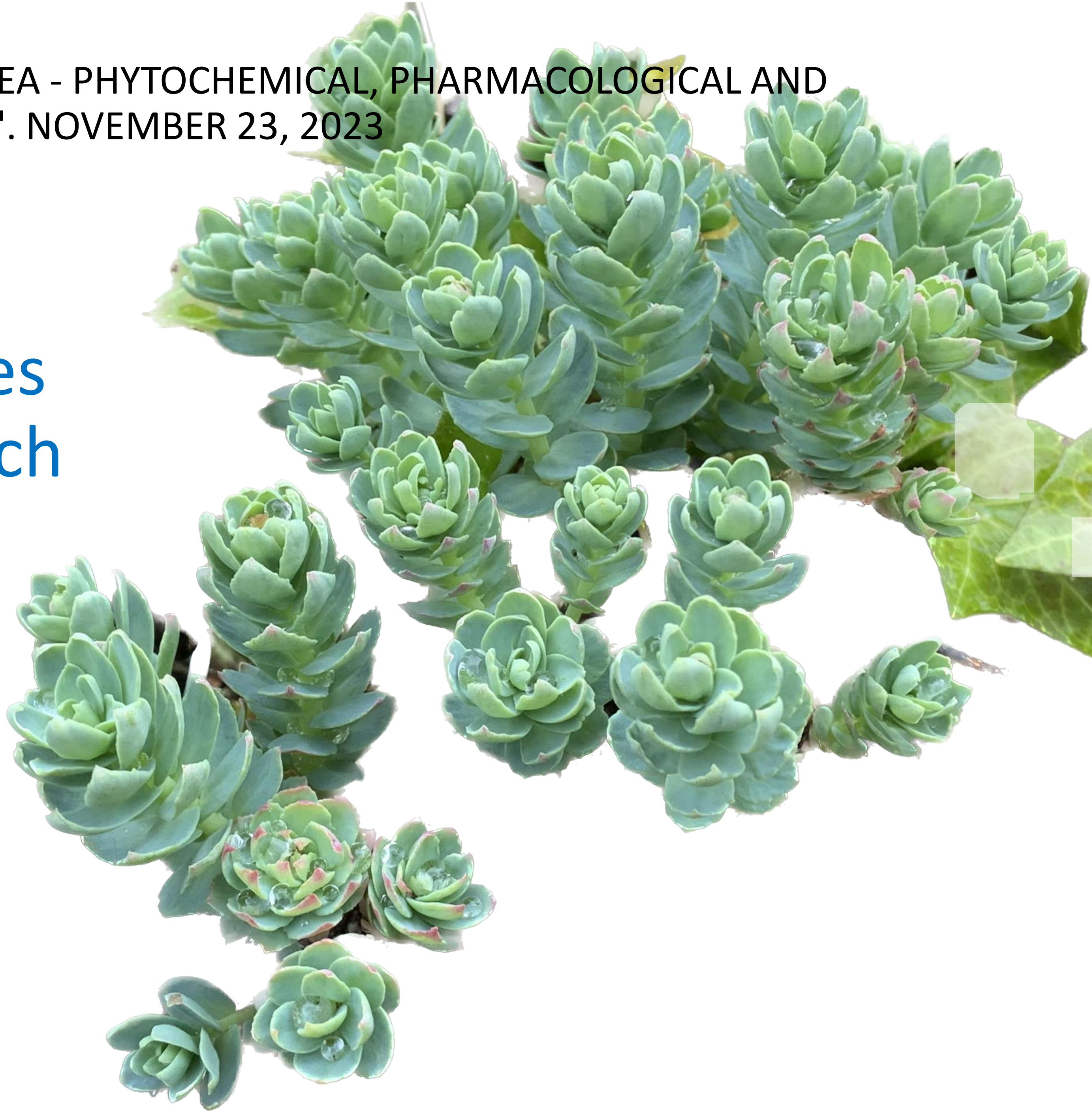

Progress and the challenges in the phytotherapy research of *Rhodiola rosea* L.

Alexander Panossian
Phytomed AB, Sweden

Progress in Phytotherapy Research of *Rhodiola rosea* L. Worldwide publications on Rhodiola since 1960 to 2023

- Overall, 910 pre-clinical and 35 clinical studies were conducted in Europe, America, and China in 2000-2023, assessed in 35 systematic and 84 descriptive reviews.

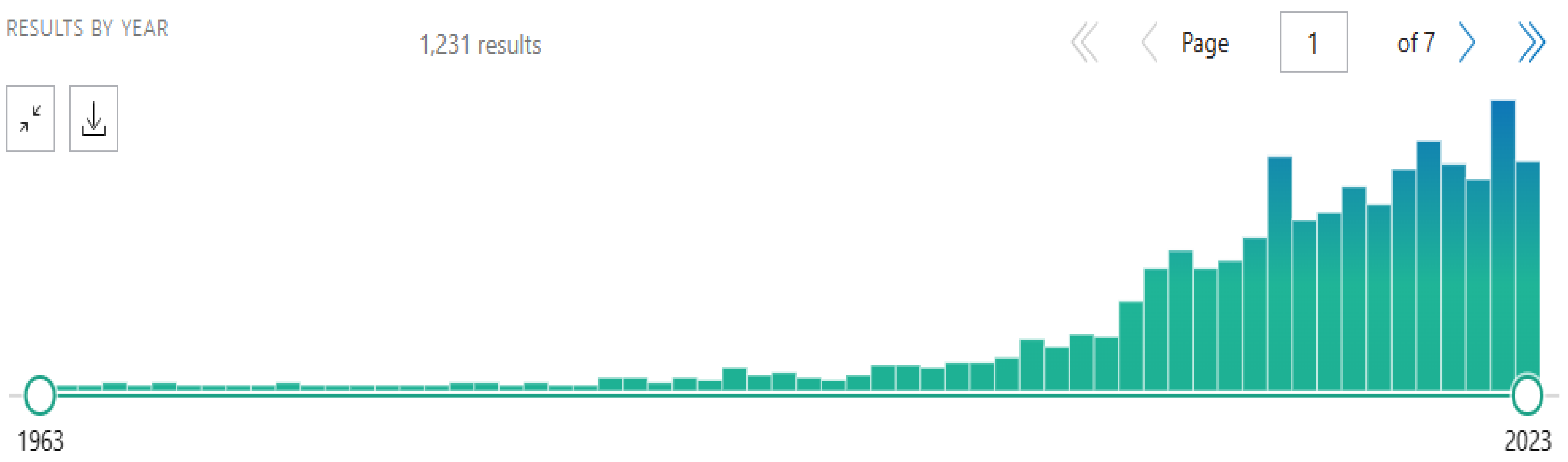

Medicinal uses of *Rhodiola rosea* L.

- **Uses described in pharmacopeias and well-established documents.**
  - CNS stimulant in asthenic conditions, increased fatigue, in neurasthenic conditions, and somatic or infectious diseases, in patients with functional diseases of the nervous system, as well as in healthy people with asthenia and decreased performance;
    - Liquid extract, DER 1:1, extraction solvent - 40% ethanol, Conventional Drug 1974, USSR.
- **Uses described in pharmacopeias and as traditional herbal medicine.**
  - THMP used as an adaptogen at decreased performance, such as fatigue and weakness;
    - Dry extract of root and rhizome (2.5-5:1) first extraction solvent ethanol 70%, second extraction solvent water; tablet containing 144 mg dry extract. Natural remedy (national legislation) 1987-2008, since 2008 registered as a THMP, SE.
  - THMP for the temporary relief of symptoms related to stress such as fatigue and exhaustion (EMA, 2023), convalescence (DK 2001) mild anxiety state (IT, 2010), irritability and tension (Be, 2014);
    - Dry extract (1.5-5:1), extraction solvent 60% ethanol m/m (= 67.7% V/V); film-coated tablet containing 200 mg dry extract. Registrations based on the above mentioned dry extract in several member states since 2008.

Rhodiola is an adaptogen promoting adaptability, improving resilience, and increasing survival of organisms in stress.

- Adaptogens are mild stressors ("stress vaccines"), activating the defence response of the organism after single or repeated administration (in the appropriate dose range), by triggering adaptive stress response pathways of body cells regulated by the neuroendocrine-immune complex (stress system).
- The adaptogenic effect covers a wide range of pharmacological activities (pleiotropic pharmacological profile) in stress-Induced and aging-related disorders.
- Under the stressor, we imply negatively affecting environmental factors of psychological, physical, viral, bacterial, and chemical origins.

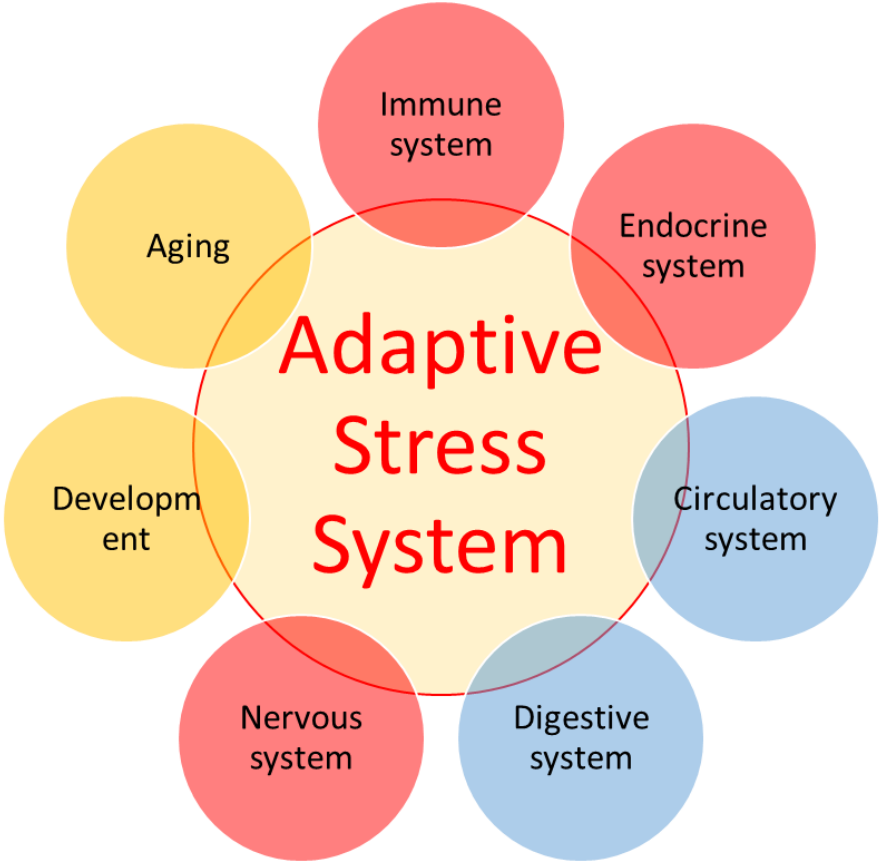

Health is the ability to adapt to one's environment

George Canguilhem
1943

- Adaptability shows the ability to learn and improve from experience.
- Adaptability is the ability of an organism to alter itself or its responses to the changed environment or circumstances.

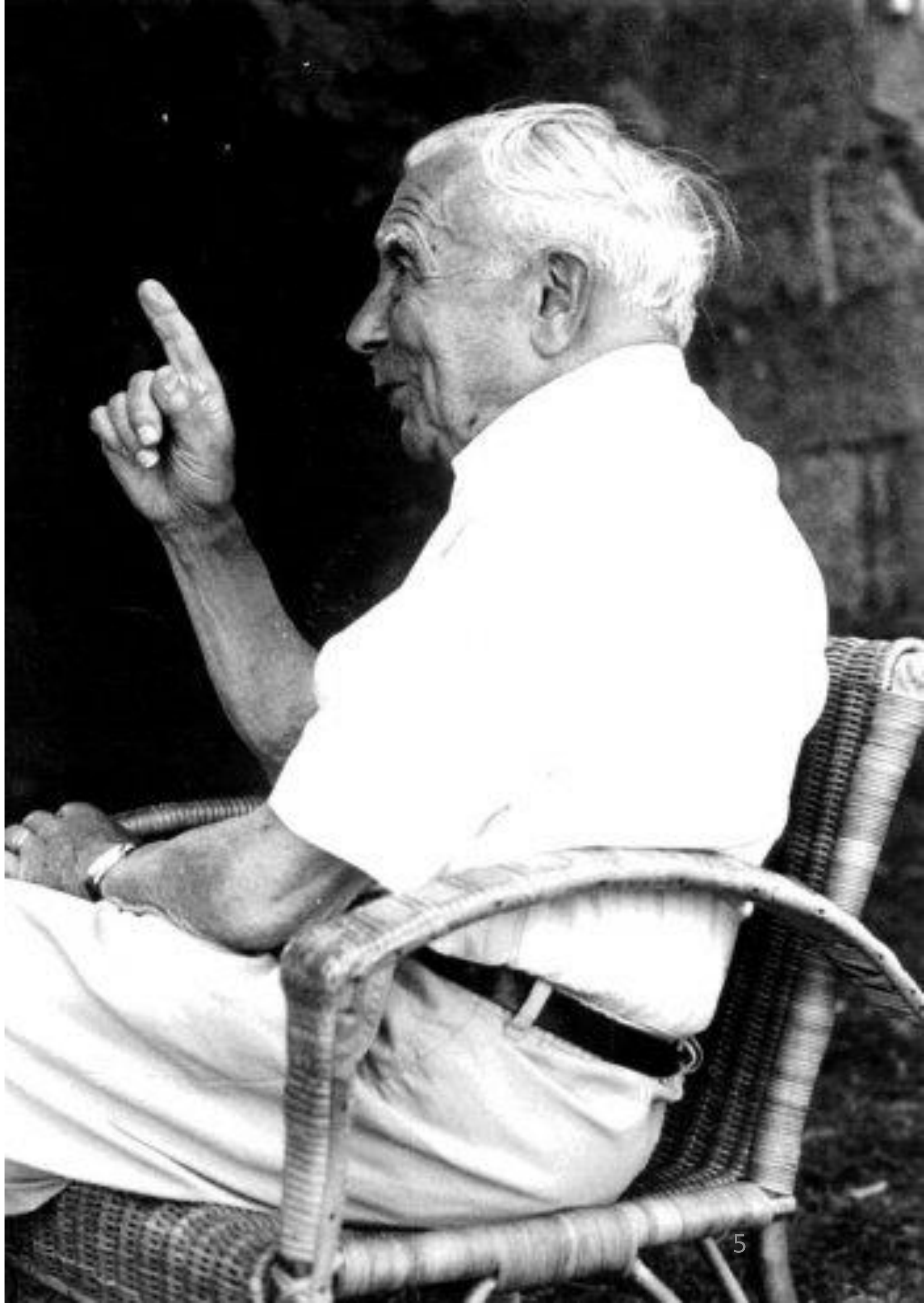

Adaptive homeostasis

Adaptive homeostasis is the transient reversible adjustments of the homeostatic range in response to exposure to mild stressors (e.g. exercise or **adaptogens**).

Chronically increased cortisol and corticotropin releasing hormone (CRH) secretion is associated with:

- immune suppression,
- depression,
- anxiety,
- increased blood pressure, tachycardia
- gastrointestinal dysfunction,
- anorexia,
- loss of libido
- chronic active alcoholism,
- alcohol and narcotic withdrawal, etc.

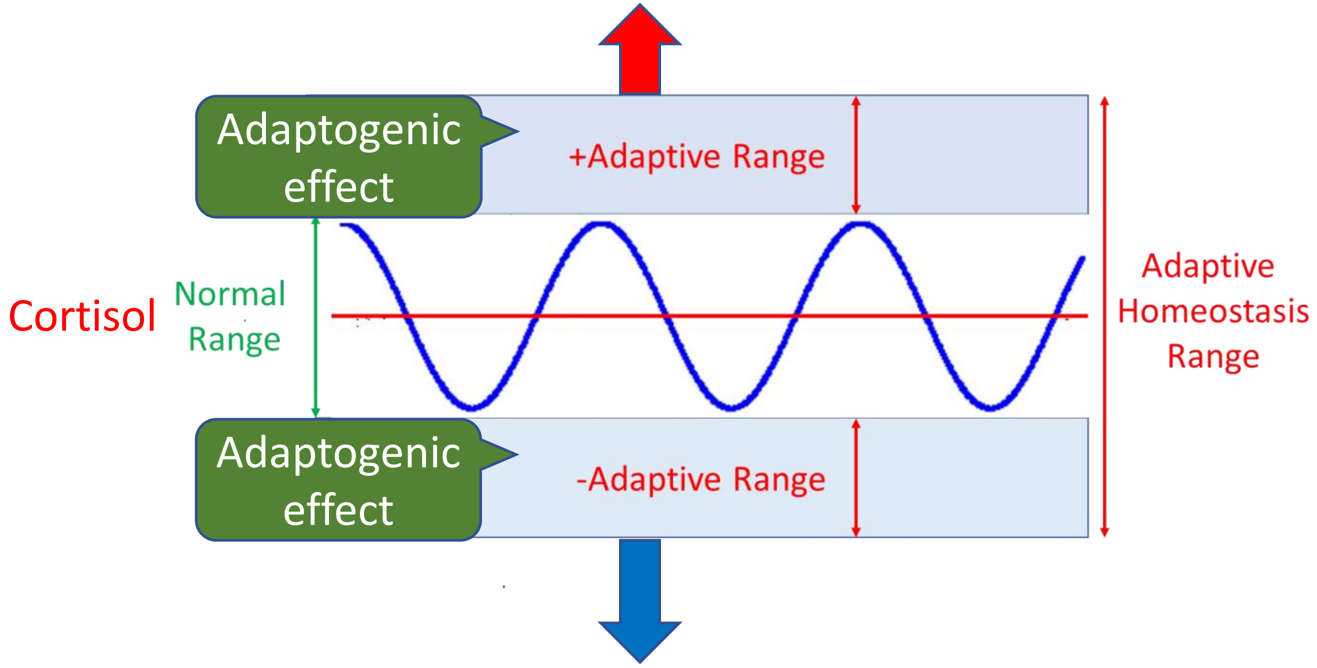

Biological functions and many biomolecules oscillate around a median within a normal (homeostatic) range.

Chronically decreased cortisol or CRH secretion is associated with:

- the chronic fatigue,
decreased arousal and performance of task
- fibromyalgia syndromes,
- increase in appetite and weight gain,
- somnolence, etc.

Adaptive stress response

Adaptive stress response factors trigger the expression of mediators and effectors of stress response in intracellular and extracellular systems boosting the defense response of an organism resulting in increased survival.

ADAPTIVE STRESS RESPONSE FACTORS

- exercise
- dietary energy restriction
- nutrition and medication **(adaptogens)**
- cognitive stimulation / emotions
- toxins
- radiation
- temperature

MEDIATORS OF CELL ADAPTIVE STRESS RESPONSE SIGNALING SYSTEM

Receptors (GPCR, NTFR, TLR, IR, etc.) and ion channels
Enzymes (PLC, AC,GC) and second messengers (IP3, DAG, cAMP)
Kinases (PKC, PI3K, MAPK, PERK)
Transcription and nuclear factors (Nrf-2, FOXOs, CREB, NF-kB)

ADAPTIVE STRESS RESPONSE EFFECTORS

Free radicals, antioxidant enzymatic system

- superoxide dismutase
- catalase
- glutathione peroxidase
- glutathione

Protein chaperones, growth factors and defense response proteins

- HSP-70
- GRP-78
- BDNF
- VEGF
- bFGF

Rhodiola and other adaptogens deregulate expression of mediators of adaptive stress response in human brain cells

- CRH - corticotropin releasing hormone
- UCN - urocortins
- GNRH1 - gonadotropin releasing hormone

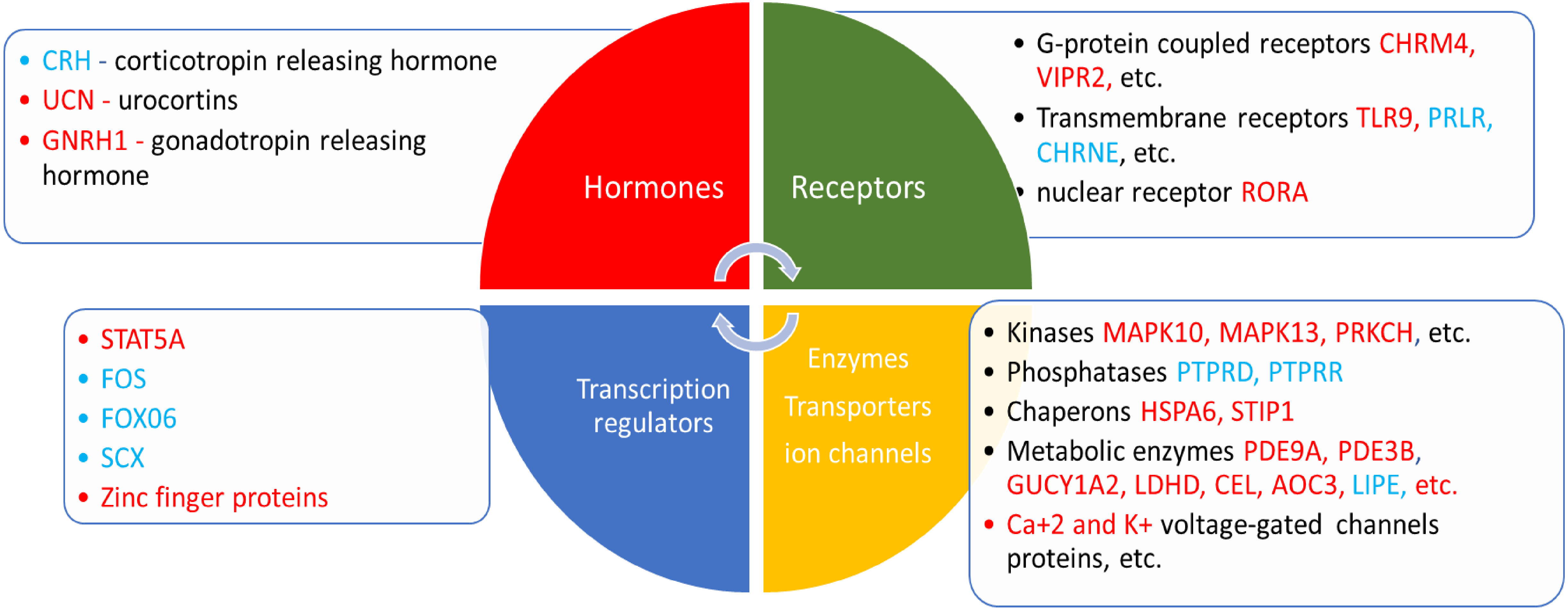

- G-protein coupled receptors CHRM4, VIPR2, etc.
- Transmembrane receptors TLR9, PRLR, CHRNE, etc.
- nuclear receptor RORA

- STAT5A
- FOS
- FOX06
- SCX
- Zinc finger proteins

- Kinases MAPK10, MAPK13, PRKCH, etc.
- Phosphatases PTPRD, PTPRR
- Chaperons HSPA6, STIP1
- Metabolic enzymes PDE9A, PDE3B, GUCY1A2, LDHD, CEL, AOC3, LIPE, etc.
- Ca+2 and K+ voltage-gated channels proteins, etc.

Dose response effect of Rhodiola and heat shock on lifespan of nematode *Caenorhabditis elegans*

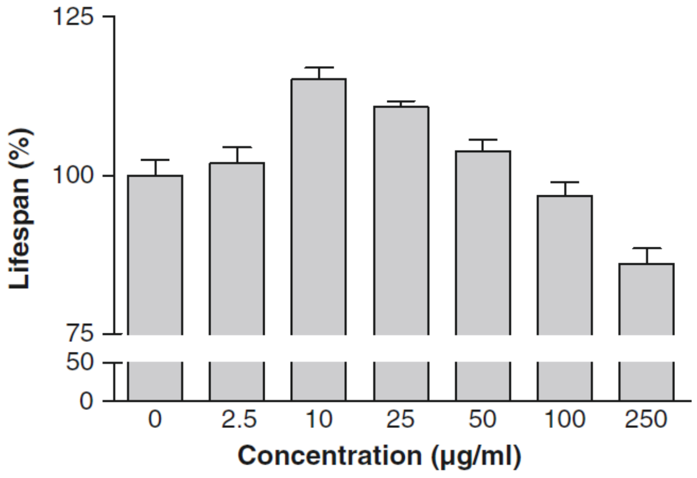

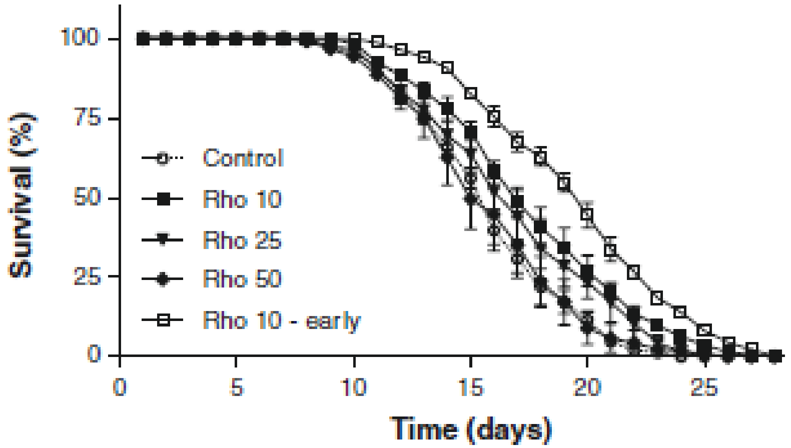

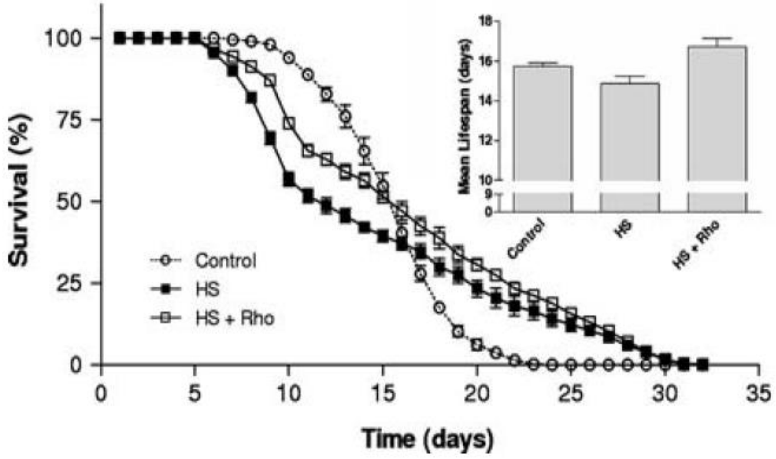

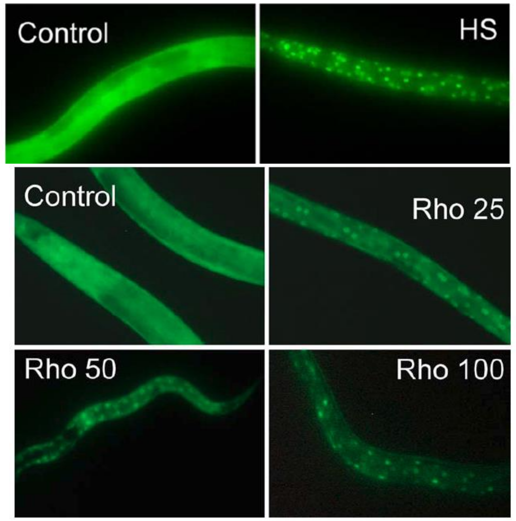

"*All things are poisons. It is only the dose which makes a thing poison*."

Rhodiola has a mild stress-mimetic effect (acting as a "stress vaccine" like a heat shock), activating cellular defence machinery to adapt the cell to stress and to increase survival and longevity *via* translocation of transcription nuclear factors of DAF-16 (FOXO) into the nucleus.

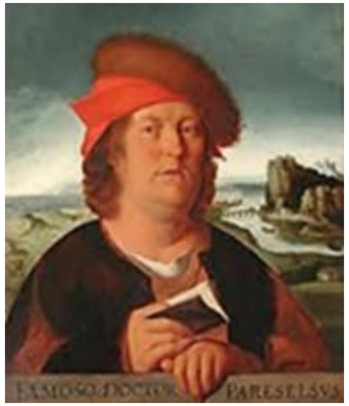

Paracelsus

The Rhodiola Modify the Response to Immobilization Stress in Rabbits by Suppressing the Increase of Stress Markers

- The stress was induced by immobilisation of the animals for 2 hours. In the placebo-group the levels of phosphorylated *Stress Activated Protein Kinase* (p-SAPK/p-JNK), *nitric oxide*, and *cortisol* were increased significantly.
- In animals treated with repeated doses of Rhodiola, the levels of nitric oxide and cortisol remained unchanged, after acute stress.
- Rhodiola and salidroside inhibit p-SAPK/p-JNK, suggesting their beneficial effects on stress.

nitric oxide

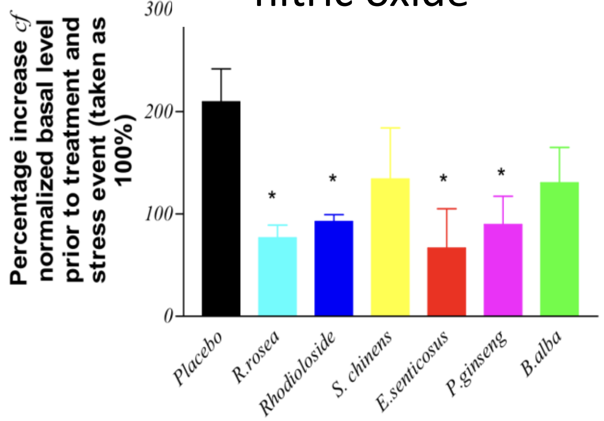

* p < 0.05 *vs* placebo

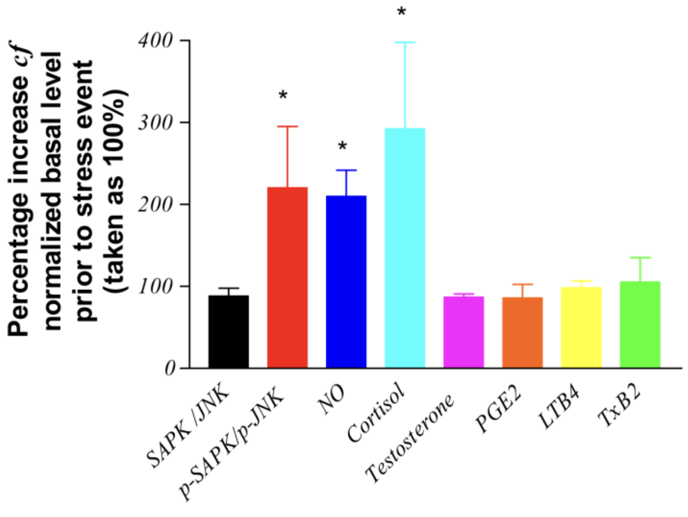

* p < 0.05 *vs* basal level

p-SAPK/JNK

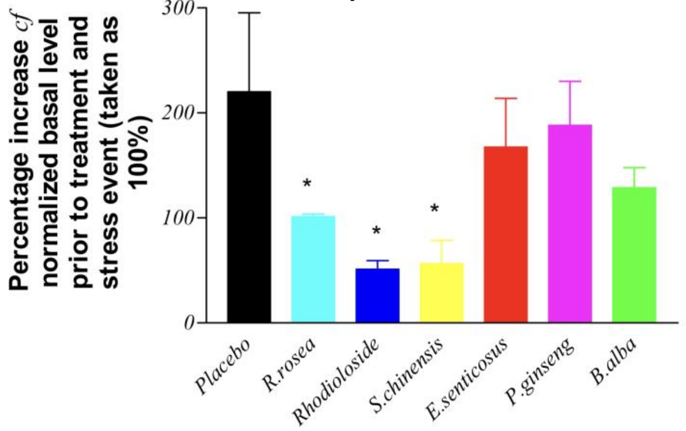

* p < 0.05 *vs* placebo

cortisol

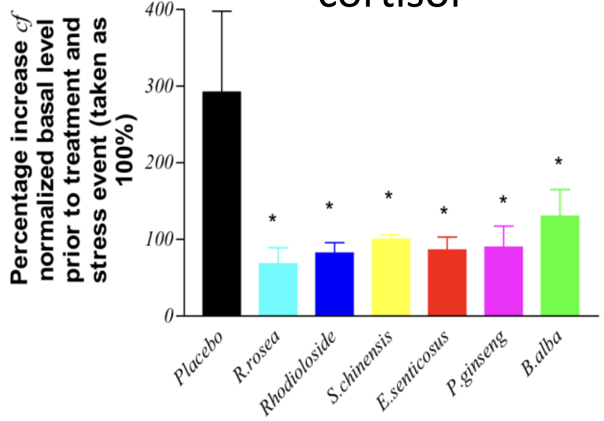

* p<0.05 *vs* placebo

2. Stress-induced changes in the concentration of: (**a**) phosphorylated stress-activated protein kinase (p-SAPK/p-JNK), (**b**) nitric id (**c**) cortisol in the blood of rabbits treated with a placebo or multiple doses of adaptogens/stress-protectors.

Effects of Rhodiola and salidroside on adaptive stress response in HPA axis

Extracellular signaling

Intracellular signaling

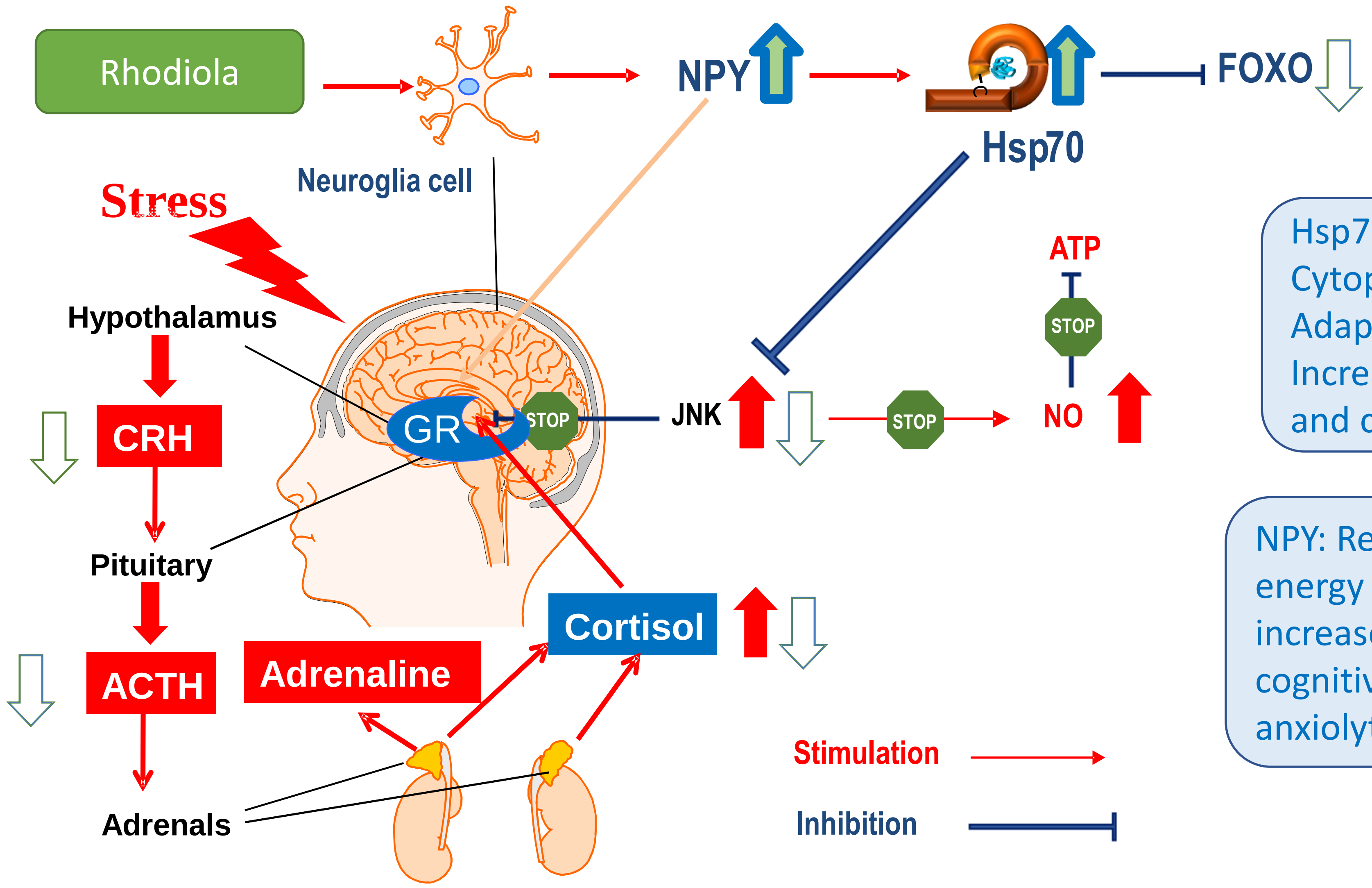

Activation of innate immunity, adaptation to stress, survival, longevity, cognitive function

Hsp70: Stimulation of immune system
Cytoprotective effect
Adaptation to stress,
Increased survival, lifespan, longevity and cognitive function

NPY: Regulation of HPA axis, maintaining energy balance, adaptation to stress, increased survival, lifespan, longevity and cognitive function, anti-depressive, anxiolytic and antinarcotic effects. etc.

Effects of Rhodiola on FOXO, Hsp70 and SAPK/JNK in oxidative stress induced inflammaging

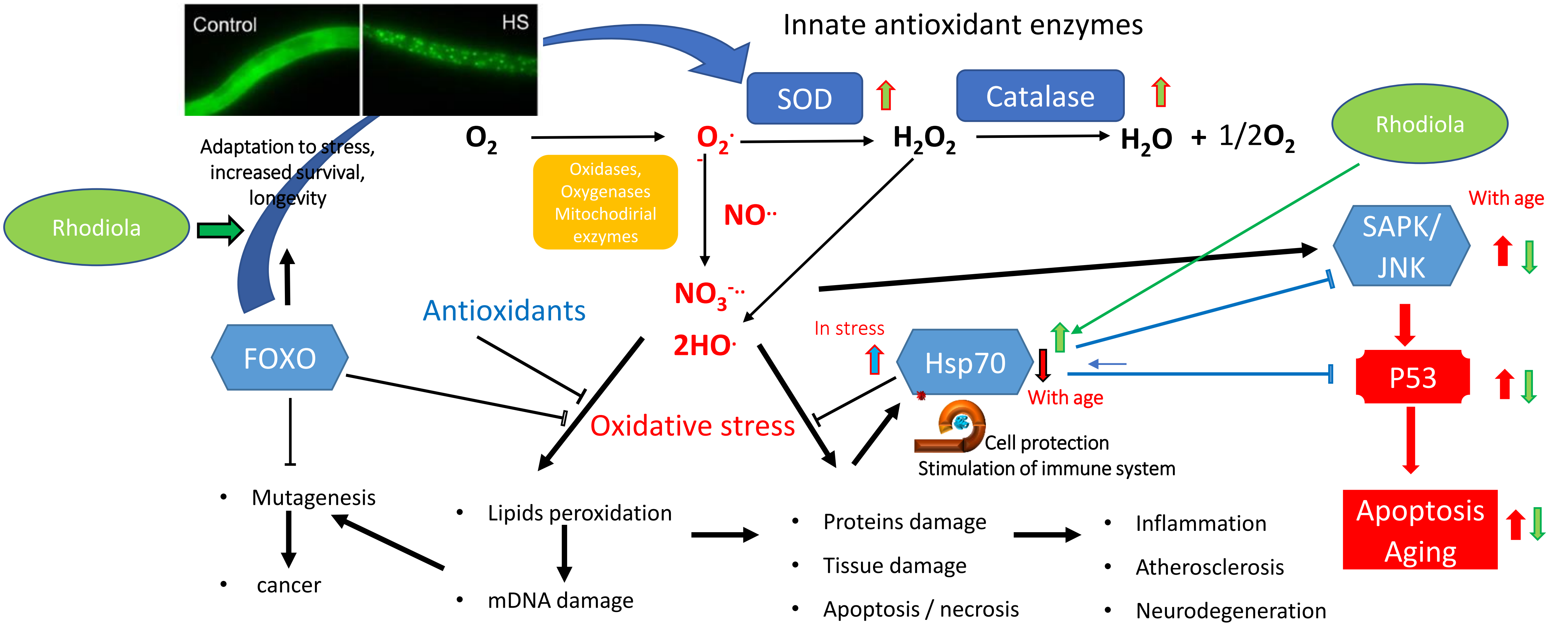

Panossian A.G. 2017. Understanding adaptogenic activity: specificity of the pharmacological action of adaptogens and other phytochemicals. Ann. N.Y. Acad. Sci. 1401(1):49-64.

Effect of salidroside on expression of NPY in neuroglia cells

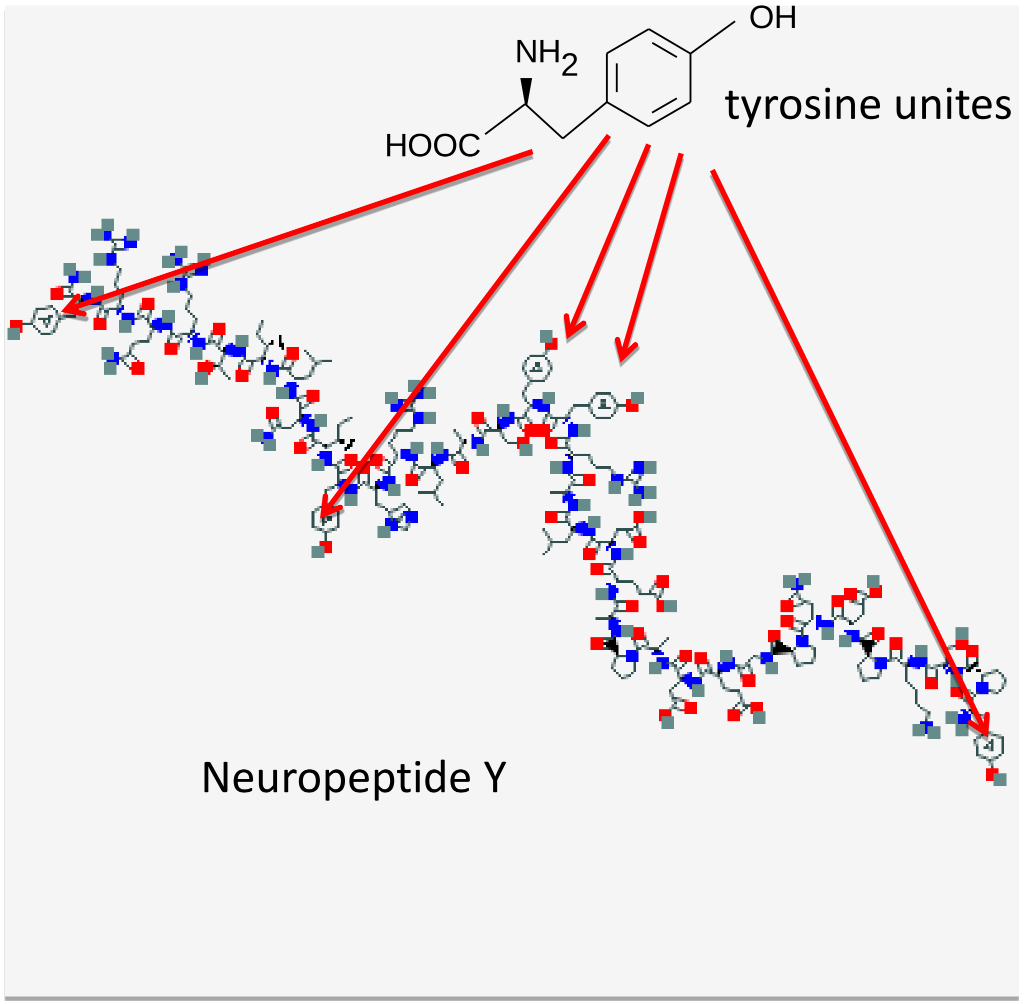

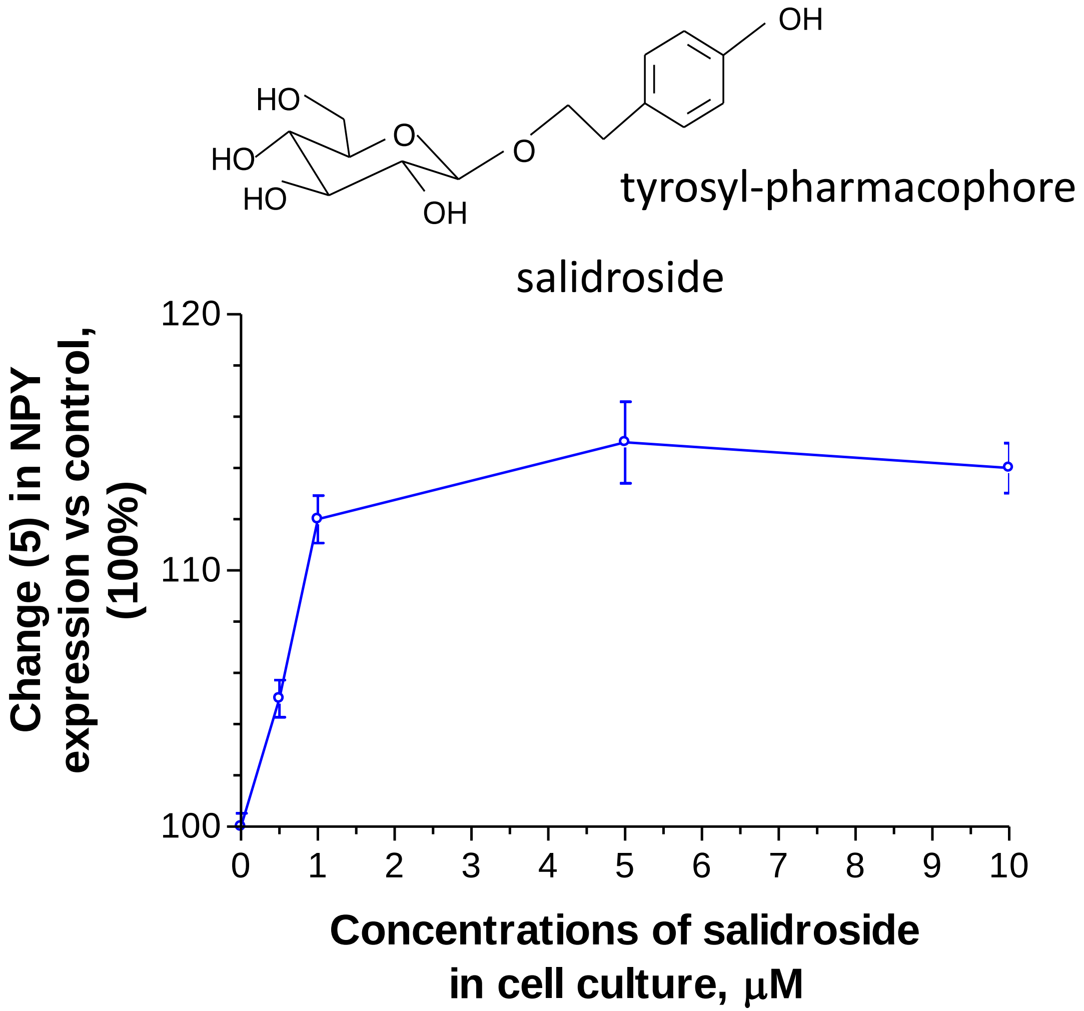

Rhodiola triggers the adaptive stress response to reduce chronic inflammation (inflammaging) and promote healthy aging

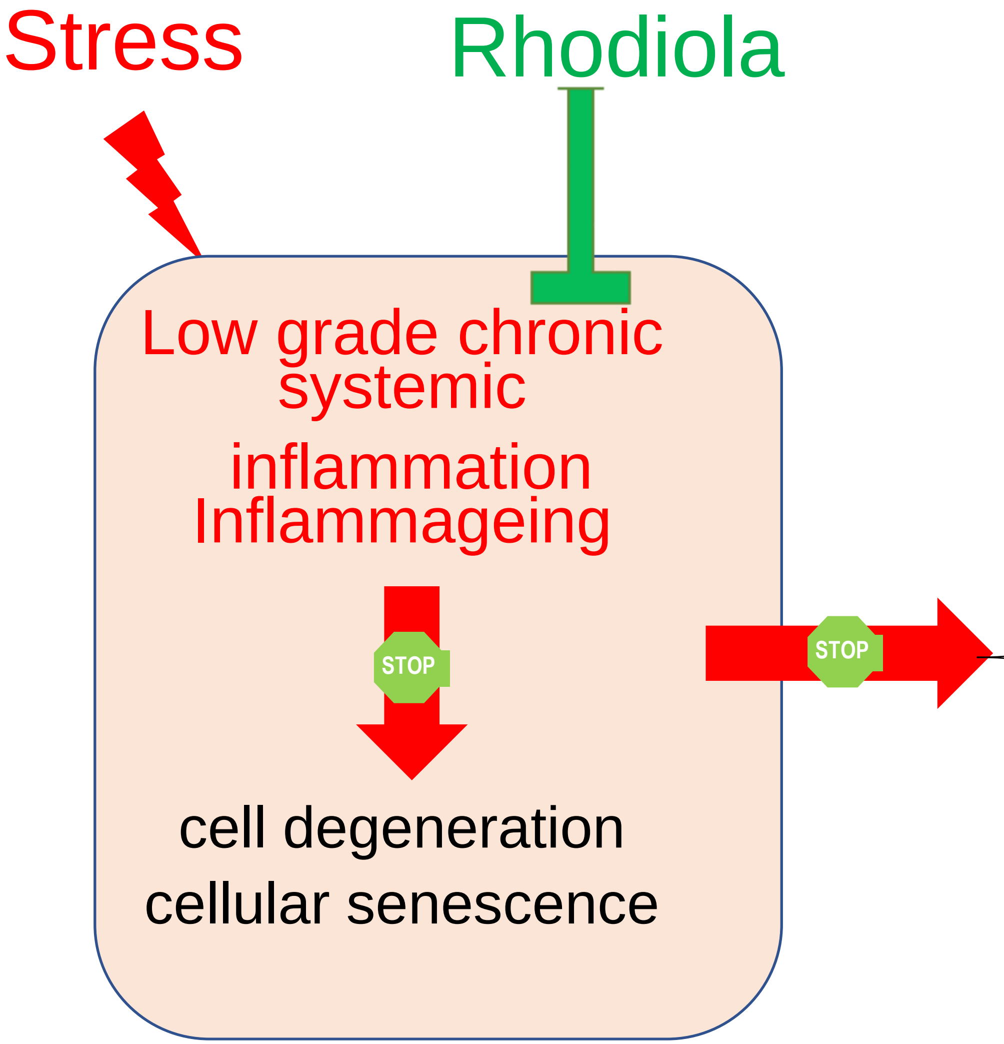

- neurodegenerative diseases
- atherosclerosis - thrombosis, infarction, stroke
- cardiovascular disease and hypertension
- cancer
- degenerative joint disease (osteoarthritis)
- type 2 diabetes, obesity
- muscle degeneration (sarcopenia), etc.

Effect of Rhodola SHR-5 on human genome in neuroglia cells: predictable effects on physiological functions and diseases

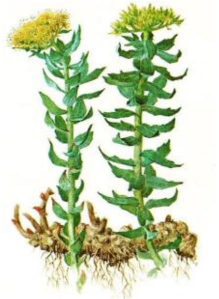

Radix Rhodiola

SHR-5 extract

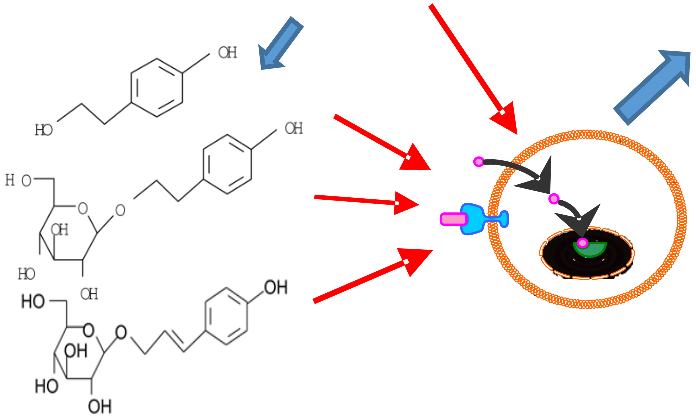

Microarray Data Analysis

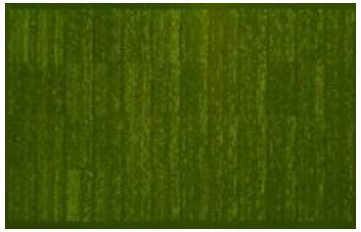

Gene Expression profiling

deregulated genes set

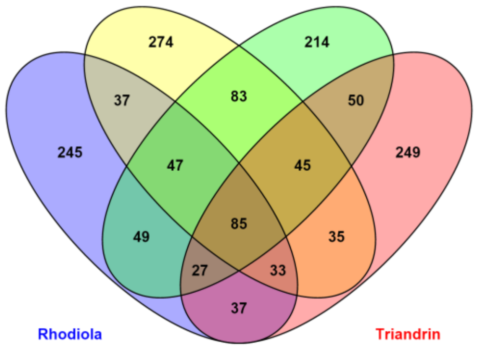

Interactive pathways downstream effect analysis

- intracellular signalling pathways :
- molecular and cellular functions,
- physiological systems functions, associated with:
    - cardiovascular (72 deregulated genes),
    - metabolic (63 genes),
    - gastrointestinal (163 genes),
    - neurological diseases (95 genes),
    - endocrine (60 genes),
    - behavioral (50 genes), or
    - psychological disorders (62 genes).

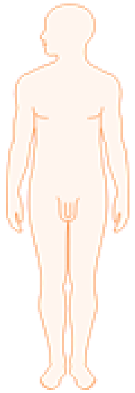

Pharmacological profile / “Signature”

Signalling pathways and disease

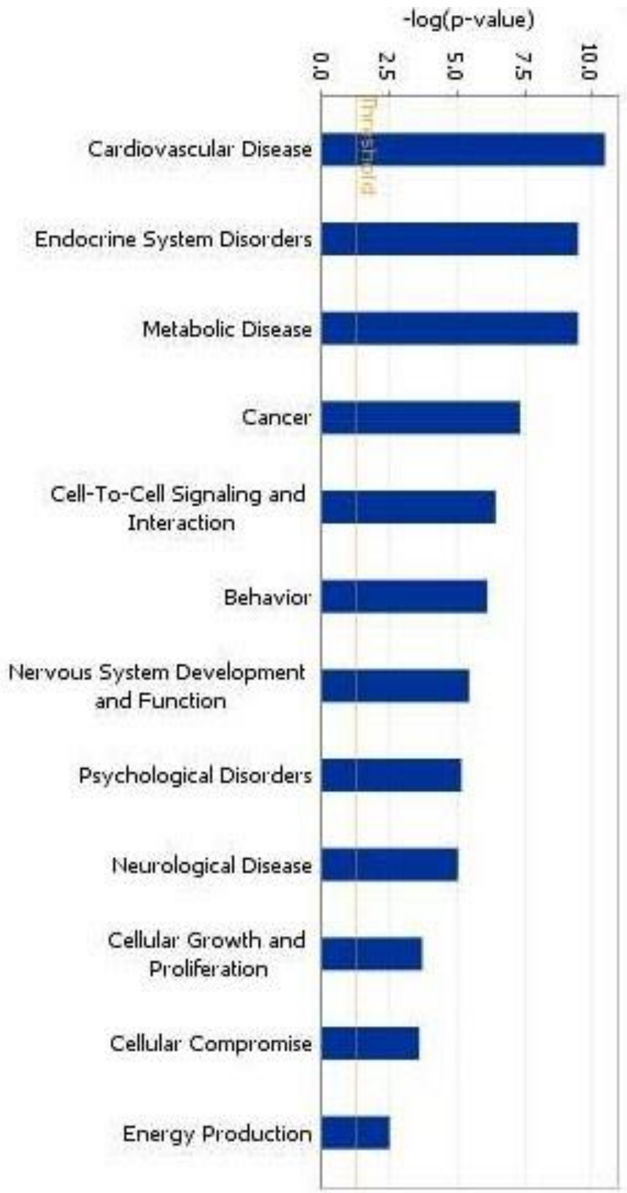

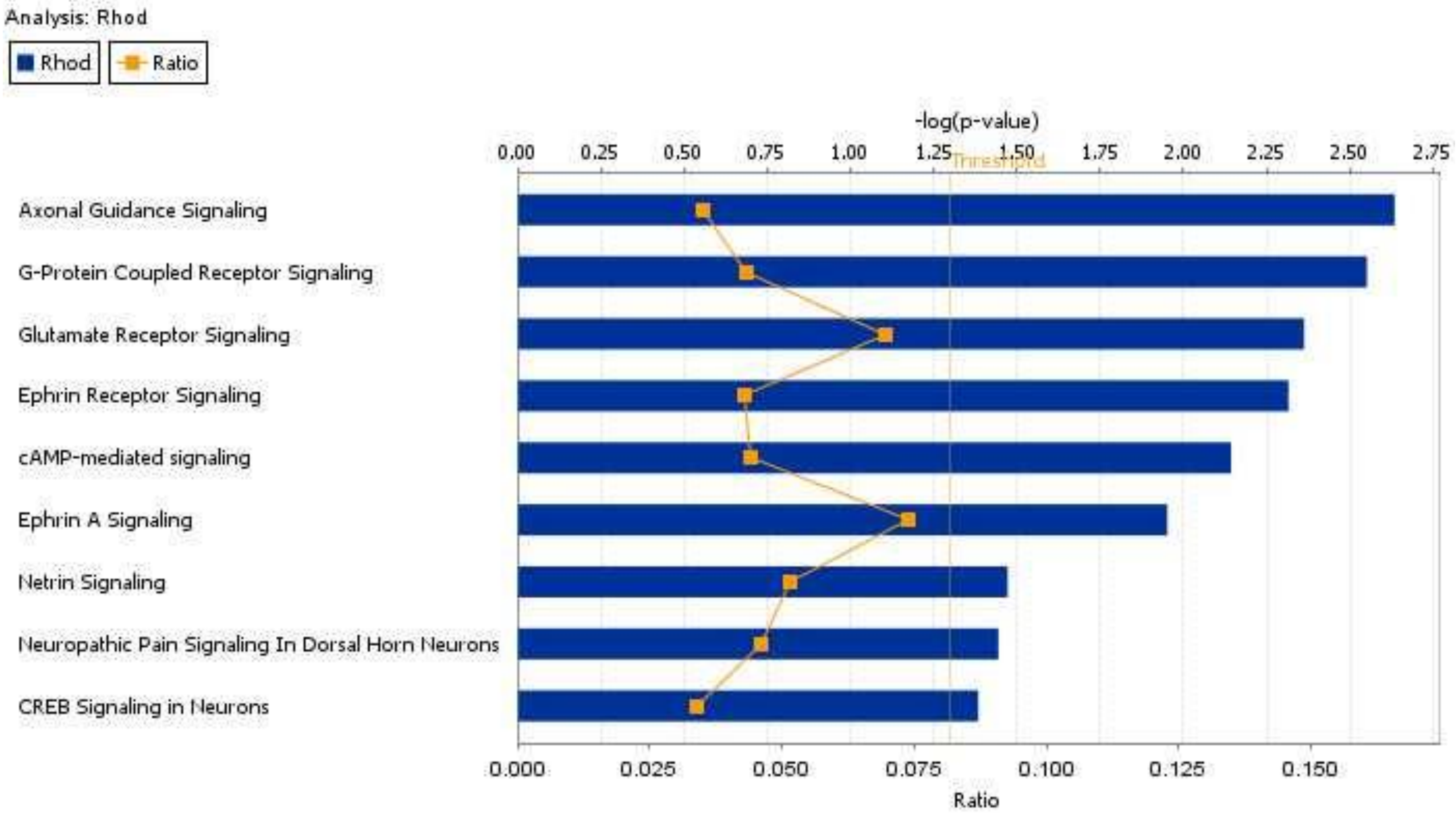

© 2000-2014 Ingenuity Systems, Inc. All rights reserved.

Pleotropic effects of Rhodiola in viral infections

Rhodiola

Direct antiviral effect

- Targets Preventing the Virus RNA Synthesis and Replication
- Termination of viral life cycle in infected host cell

Anti-inflammatory activity

- Inhibition of NFkB mediated signaling
- Inhibition of PLA2, arachidonic acid release and metabolism
- Inhibition of nitric oxide generation

Modulation of the immune response

- Increased expression of pathogen pattern recognition proteins - TLRs,
- Increased expression of interferons,
- Inhibition of cytokines release,
- Activation of melatonin signaling pathway

Detoxification and reparation of oxidative stress induced damages in compromised cells

- Activation of NRF2-signaling pathway proteins (KEAP1)
- Production of Phases I,II metabolizing and antioxidant enzymes : glutathione S-transferase (GST), NAD(P)H quinone oxidoreductase 1 (NQO1) , superoxide dismutase (SOD), and heme oxygenase 1 (HO1).
- Molecular chaperons Hsp70 mediated cytoprotecting, and repairing processes
- Activation of melatonin signaling pathway

Rhodiola SHR-5 extract deregulates gene expression of G-proteins receptors and key mediators of GPCR-mediated stress-response signalling pathways. Reducing the expression of GPCRs, decreases sensitivity to stressors and increases resilience to stressors, including emotional, physical, heat, chemical, toxic, infectious, malignant, etc.

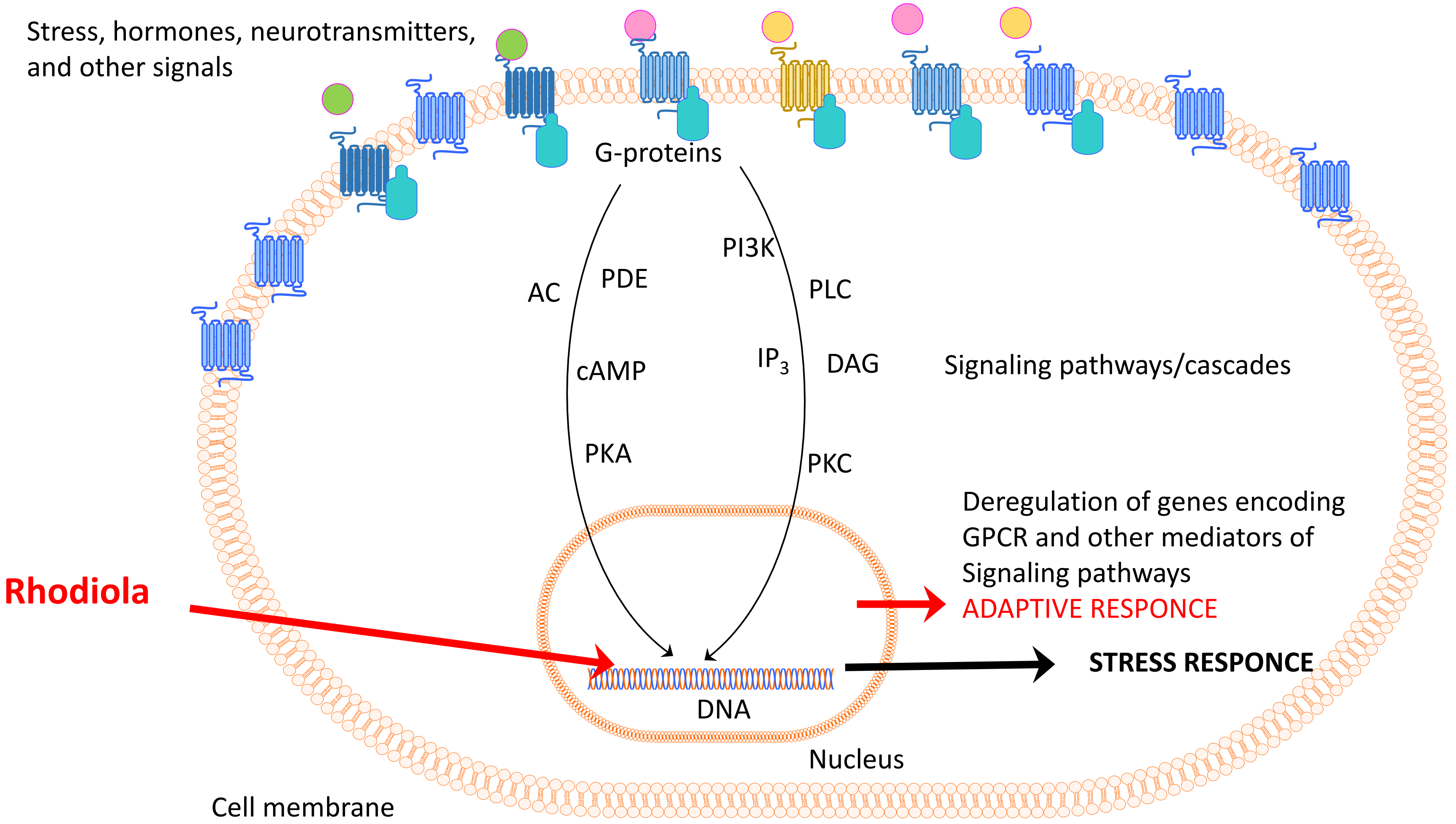

The effect of Rhodiola and other adaptogens on adaptive stress response signaling pathways

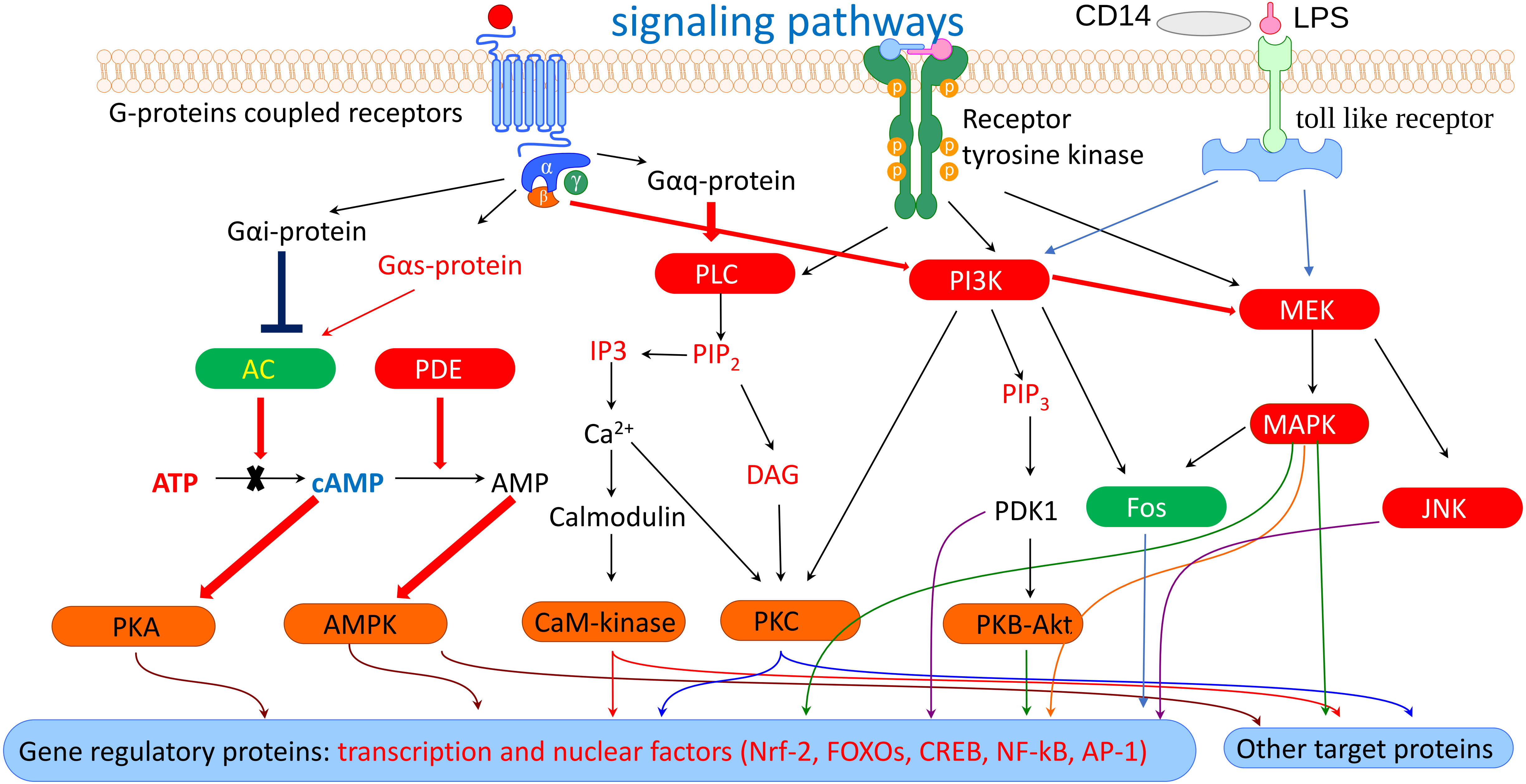

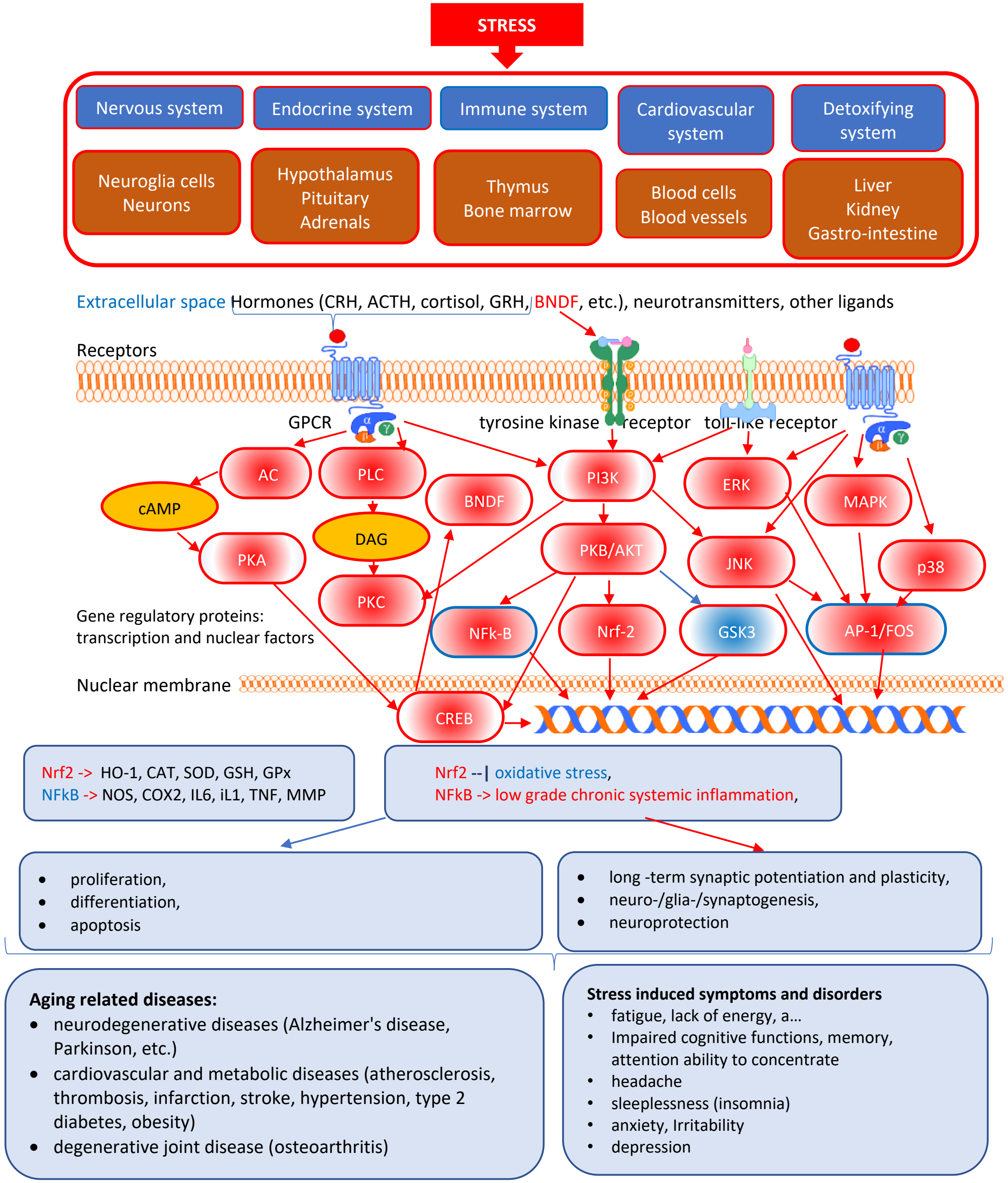

The molecular mechanisms and modes of the pharmacological action of Rhodiola: Effects of Rhodiola and salidroside on key mediators of neuro-endocrine immune complex.

The mechanisms of adaptogenic action of Rhodiola describe the molecular changes and their extracellular and intracellular interactions

The modes of the pharmacological action of Rhodiola describe functional changes of cells and regulatory systems involved in defense response at various levels of regulation of homeostasis and the phases of progression of diseases

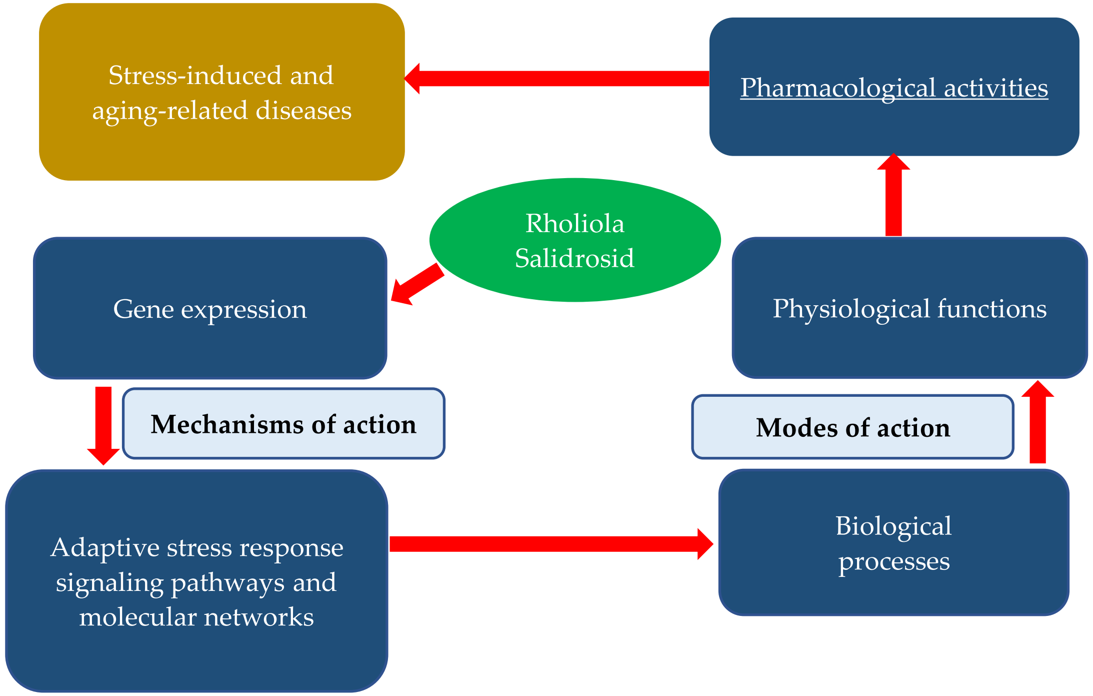

Effects of Rhodiola and other adaptogens on adaptive stress response signaling pathways that protect neurons against degeneration and promote synaptic plasticity.

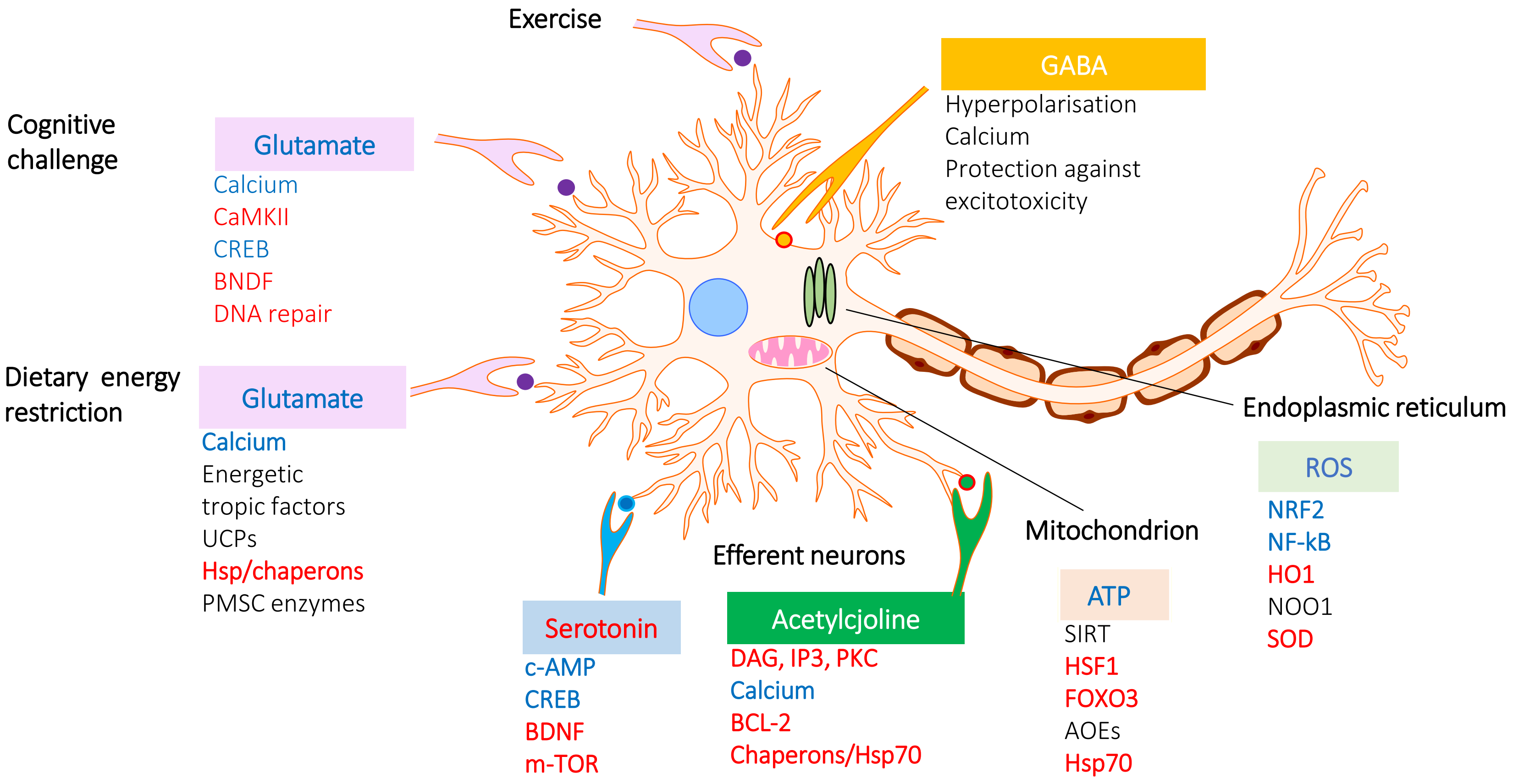

Upregulation or activation

Downregulation or inhibition

Stranahan, A.M. &M.P.Mattson. 2012. Recruiting adaptive cellular stress responses for successful brain ageing. *Nat. Rev. Neurosci.* **13:** 209–216.
Panossian A.G. 2017. Understanding adaptogenic activity: specificity of the pharmacological action of adaptogens and other phytochemicals. Ann. N.Y. Acad. Sci. 1401(1):49-64.

Pleiotropic effects of leukotrienes in Alzheimer disease and leukotriene inhibition by Rhodiola

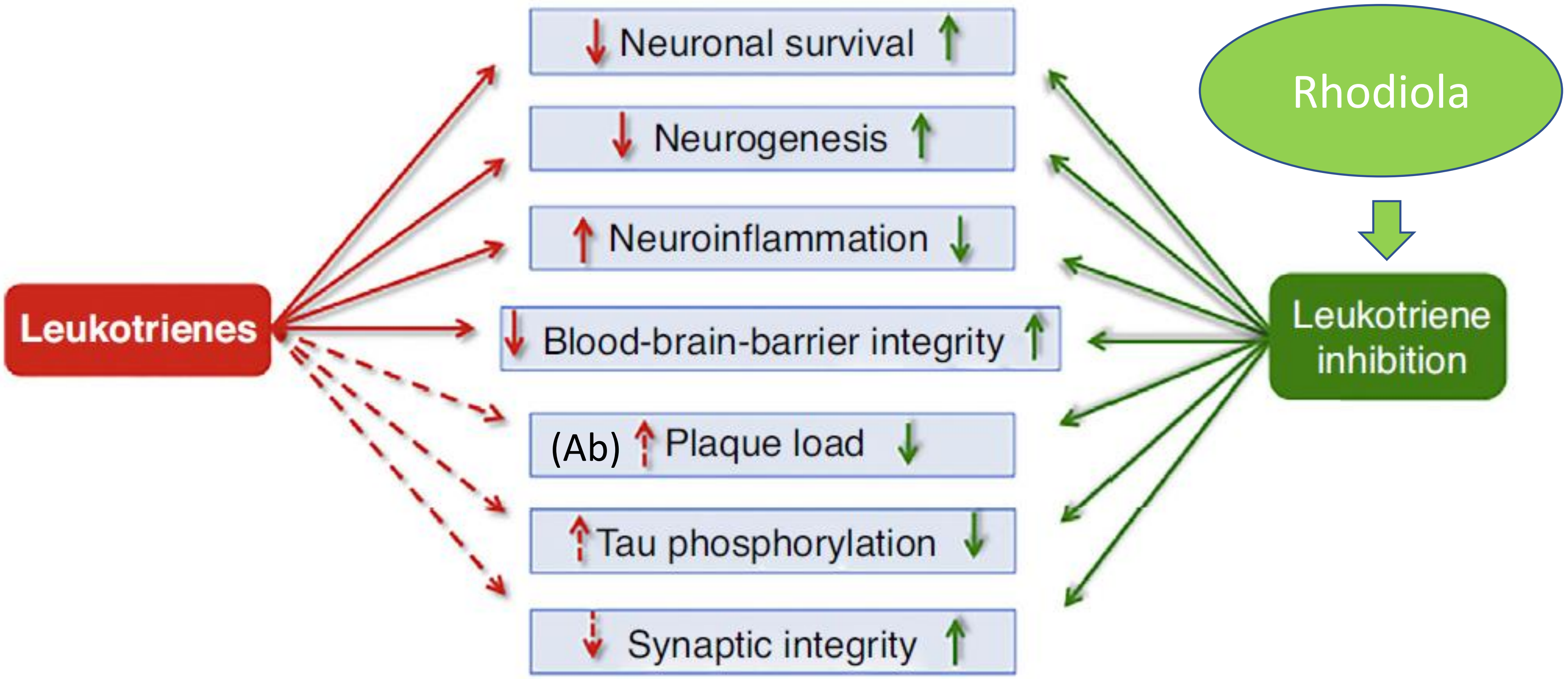

Michael et al. 2018. Drug Discov. Today. DOI: 10.1016/j.drudis.2018.09.008

Panossian et al. 2019 Phytomedicine

Effect Rhodiola on eicosanoid signaling pathway

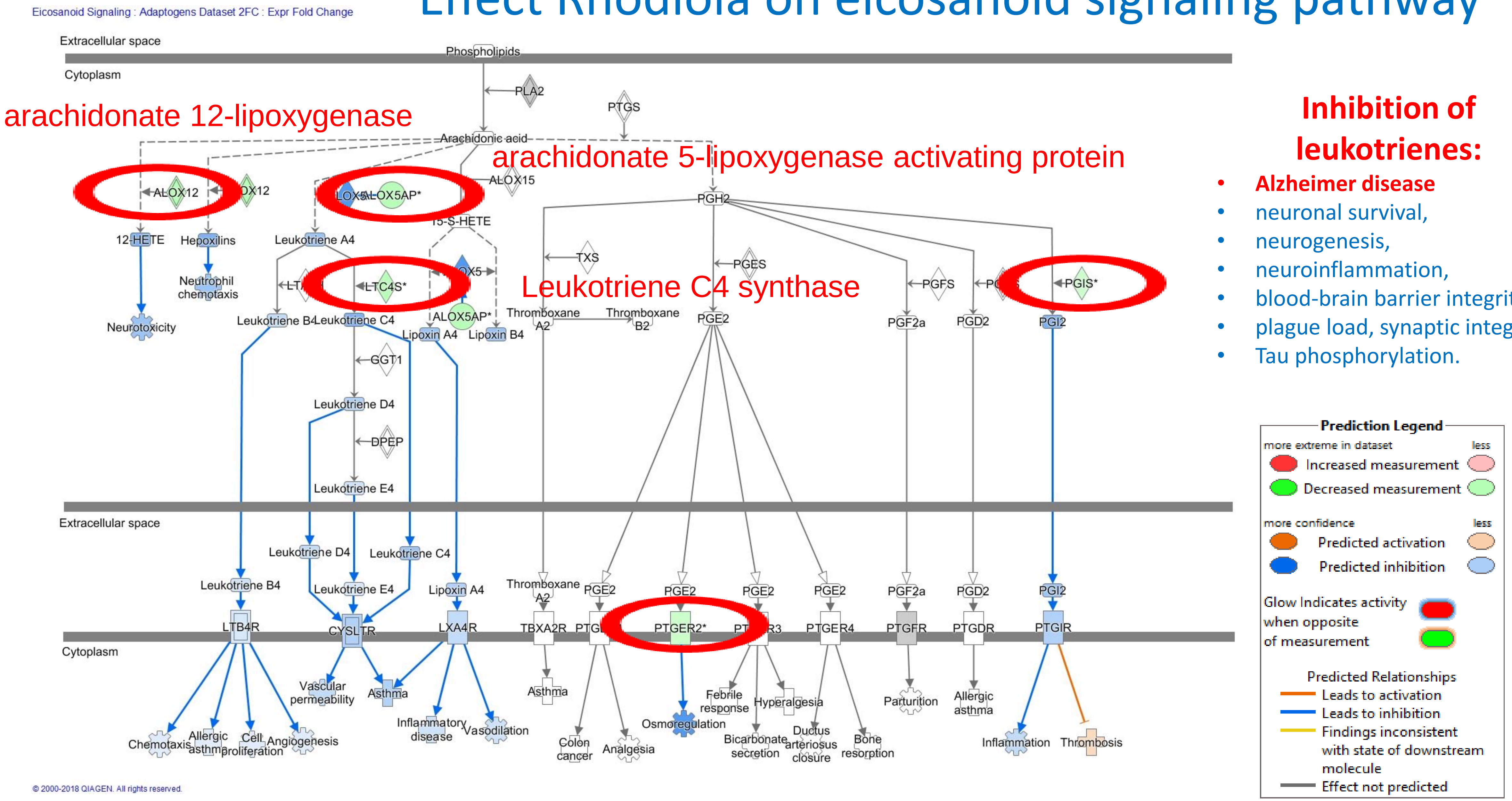

Inhibition of leukotrienes:

- **Alzheimer disease**
- neuronal survival,
- neurogenesis,
- neuroinflammation,
- blood-brain barrier integrity,
- plague load, synaptic integrity,
- Tau phosphorylation.

Summary of characterisation

- *Chemical class:* predominantly tetracyclic triterpene, phenethyl-and phehylpropanoids, etc.
- *Pharmacological activity profile*: adaptogenic - nonspecific and pleiotropic, including:
  - stress-protective, neuroprotective, anti-narcotic, anxiolytic, antidepressive, cardioprotective, anti-hypoxic, radioprotective, hepatoprotective, anti-toxic
  - stimulating cognitive functions (attention, learning, and memory), physical strength and endurance, anti-fatigue, geriatric,
  - anti-inflammatory, anti-allergic, immunotropic (mediated *via* the immune system – anti-viral, anti-bacterial, and anti-carcinogenic effects), antiviral.
- *Mechanism of action:* multitarget effect on neuroendocrine-immune complex (stress-system) including:
  - triggering of intracellular and extracellular adaptive signaling pathways that promote cell survival and organismal resilience in stress,
  - regulation of metabolism and homeostasis via effects on expression of stress hormones (corticotropin and gonadotropin releasing hormones, urocortin, cortisol, neuropeptide Y, heat shock proteins Hsp70) and their receptors.
- *Potential indications for use and health claims:* stress-induced fatigue, mental and behavioral disorders, infectious diseases, and aging-associated disorders.

Clinical trials of *Rhodiola rosea* L. preparations

- **Uses supported by clinical data – 35 including 23 RCT**
  - Mild/moderate depression – 4/7,
  - Anxiety – 2,
  - Burnout symptoms -3,
  - Fatigue syndrome -3,
  - Fatigue - 9
  - Life-Stress Symptoms and Stress-Induced Conditions in healthy subjects -21
  - Aging related cognitive deficiencies of healthy subjects -2
  - Exercise performance in healthy subjects - 17

Clinical studies of hybrid herbal preparations (HHP) comprising fixed combinations of Rhodiola with other plants

Twenty clinical studies were conducted with HHPs containing Rhodiola in combinations with:

- Green Tea, in healthy subjects under stress - 4,
- Cordyceps, in healthy subjects under stress -2,
- Gingko, in healthy subjects under stress -1,
- Caffeine in healthy subjects under stress -1,
- Schisandra, and Eleutherococcus in healthy subjects under stress, and in patients with symptoms of acute pneumonia or Long COVID-19 -5,
- Black Cohosh in patients with menopausal symptoms -1,
- Saffron in patients with mild depression - 1,
- Glycyrrhiza glabra and Eleutherococcus in patients with chronic parodontids -1,
- Carnosine in patients with sensitive skin – 1.

Hybrid herbal preparations (HHP) have different pharmacological profiles/conditional "signatures" compared to their ingredients.

Challenges in Phytotherapy Research of *Rhodiola rosea* L.

1. Elucidate the modes of pleiotropic pharmacological activity of Rhodiola extracts and uncover molecular mechanisms of action of Rhodiola extracts, purified active constituents, and HHPs - the combinations of Rhodiola with other plants' extracts.
   - *Comment:* An assumption that the efficacy (and safety) of Rhodiola extract has the same pharmacological profile with identical conditional "signature" as its active marker (e.g., salidroside) or an HHP (e.g., Rhodiola with Green Tea) is a myth, which is not supported by observations and evidence.
2. Adequately to ensure a consistent clinical efficacy and safety of Rhodiola preparations by the reproducible quality of Rhodiola extracts with identical "HPLC/TLC fingerprint" and conditional biological "signature."
   - *Comment:* Ensuring the reproducible quality of the product based on a product specification by validated analytical methods and HPLC/TLC fingerprinting is mandatory, however can be insufficient for the reproducible efficacy of the product, which has a different pharmacological profile with a biological conditional signature. Comparative assessment of pharmacological profile/conditional signature is imperative for adequate conclusion of efficacy and safety of herbal preparations.

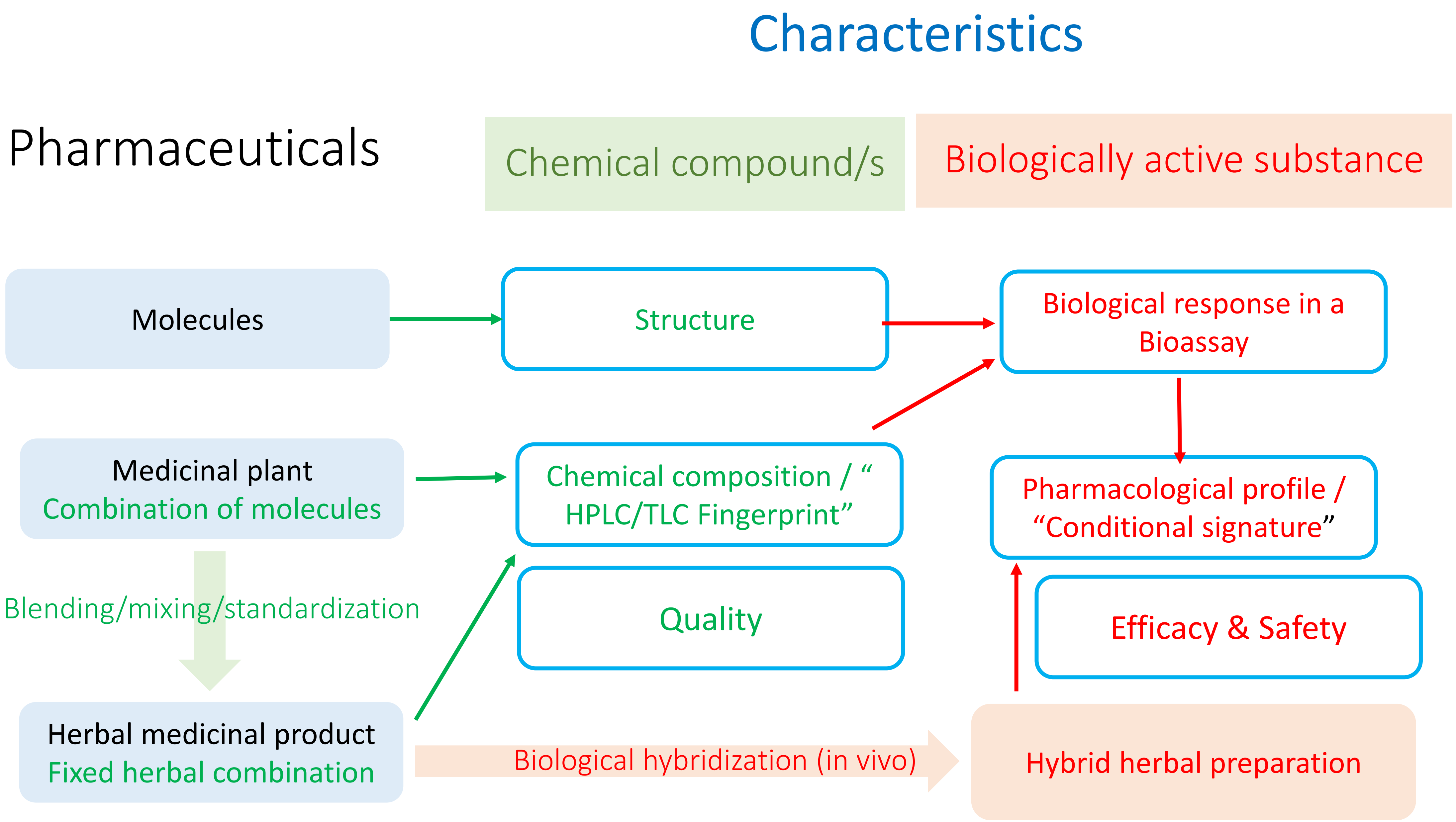
Characteristics
Pharmaceuticals
Chemical compound/s
Biologically active substance
Molecules
Structure
Biological response in a Bioassay
Medicinal plant
Combination of molecules
Chemical composition / " HPLC/TLC Fingerprint"
Pharmacological profile / "Conditional signature"
Blending/mixing/standardization
Quality
Efficacy & Safety
Herbal medicinal product
Fixed herbal combination
Biological hybridization (in vivo)
Hybrid herbal preparation

Challenges

Pharmaceuticals

Reproducible Quality

Reproducible Efficacy and Safety

Medicinal plant
Combination of molecules

Herbal medicinal product
Fixed herbal combination

Composition / “Fingerprint”

Pharmacological profile / Conditional “ Signature”

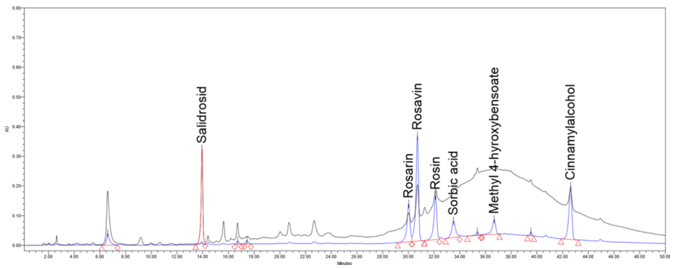

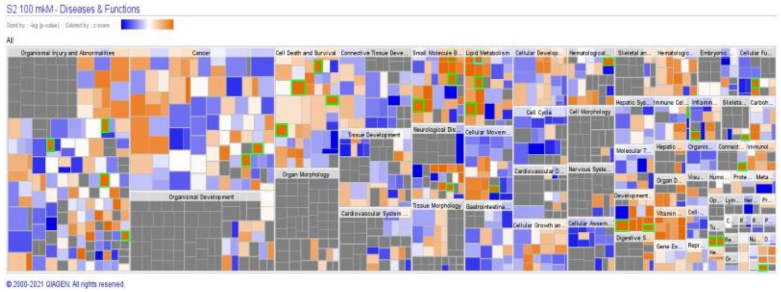

USP Herbal Medicines Compendium
U.S. Pharmacopeial Convention
http://hmc.usp.org

Rhodiola rosea Root and Rhizome — Identification

Thin-Layer Chromatography

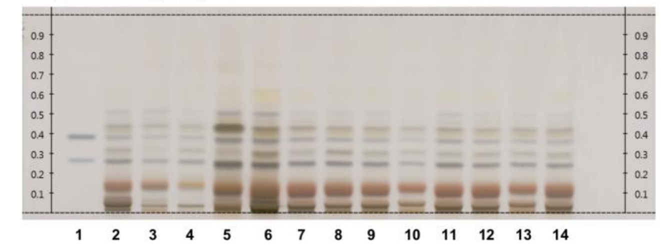

Typical HPTLC Chromatograms

These chromatograms are supplied for information only

Track assignment: 1) USP Rosavin RS and rosarin, with increasing R_F, (1.0 mg/mL); 2) USP *Rhodiola rosea* Powdered Extract RS (50 mg/mL); 3-14) *Rhodiola rosea* Root and Rhizome, commercial samples

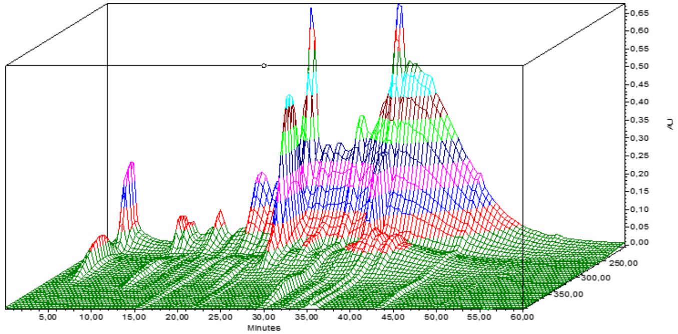

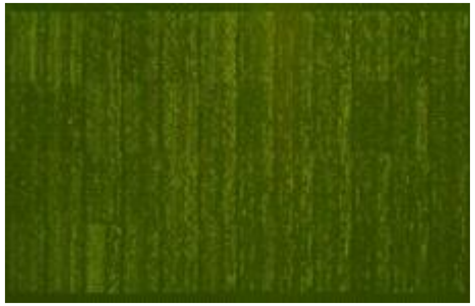

Microarray of gene expression profile: deregulated genes

Characterization of pharmacological profile by Integrative OMICS profiling - "signature" of pharmacological intervention

Predicting the response of the human body to medication requires an understanding:

- How Integrative OMICS profiling changes in health and disease?
- How Integrative OMICS profiling changes after pharmacological intervention?

The transcriptome found the most informative, Michael Snyder, 2012

Integrative Personal Omics Profiling

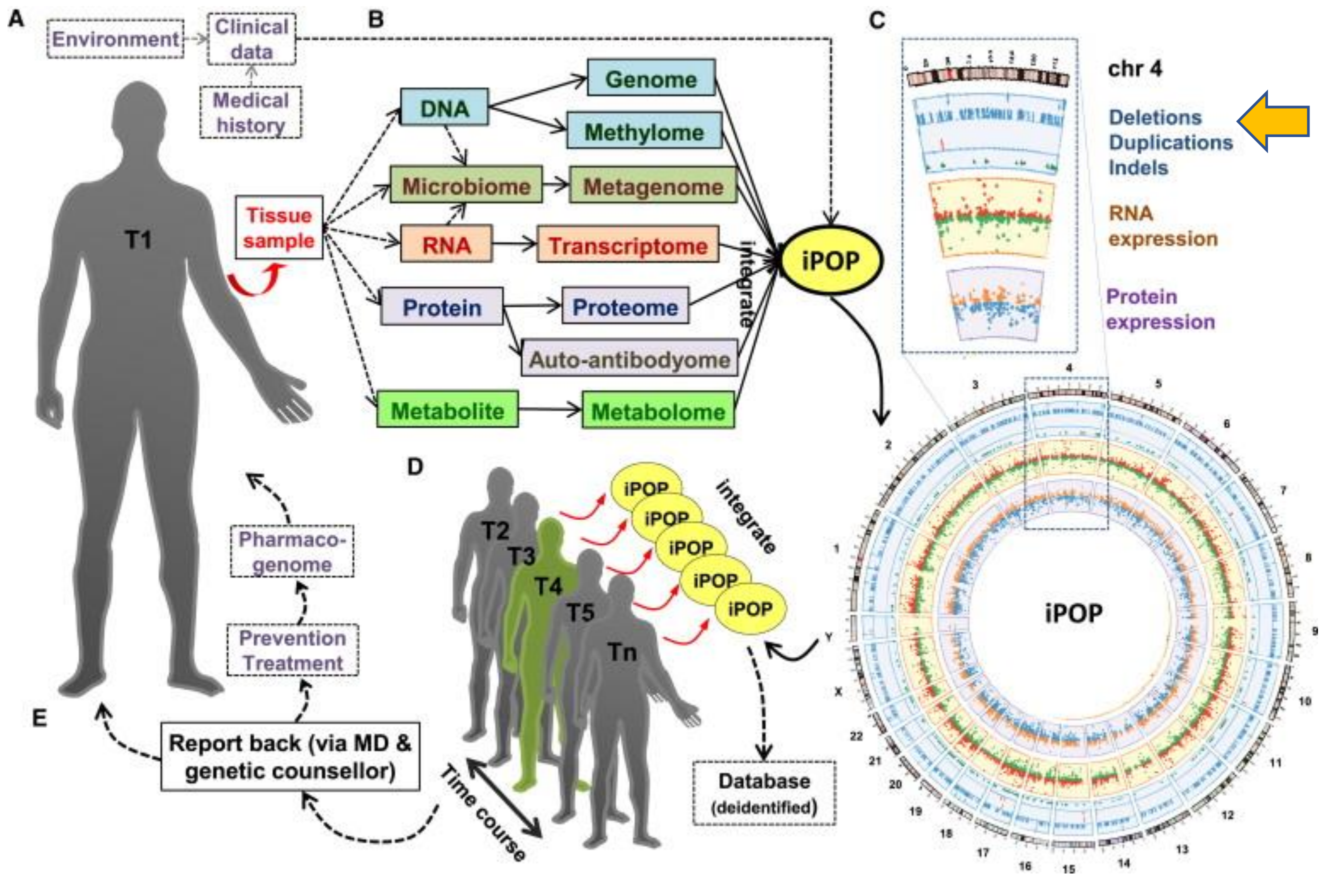

Herbal extracts	Chemical compositions “3D HPLC Fingerprint”<br><br>Number of plant secondary metabolites identified in Rhodiola extracts,<br>n = A+B+C=ABC	Pharmacological effects, Conditional “Signatures”,<br><br>Number of deregulated genes in host cells on transcriptome level of regulation in target cells,<br>N = A+B+C=D
A - Rhodiola	140	631
B - Eleutherococcus	35	669
C - Schisandra	32	625
Combination A+B+C	140+32+35=207	678
E – Salidroside isolated from Rhodiola	1	640

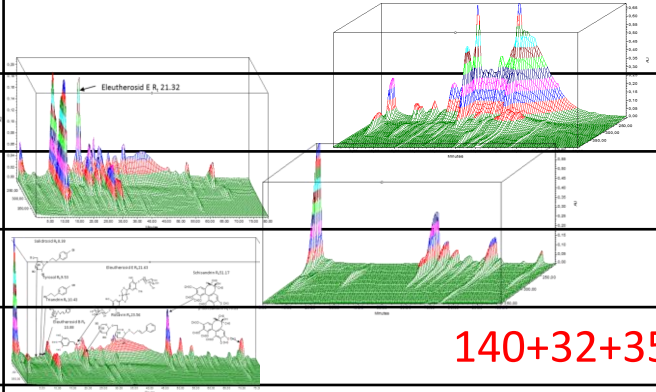

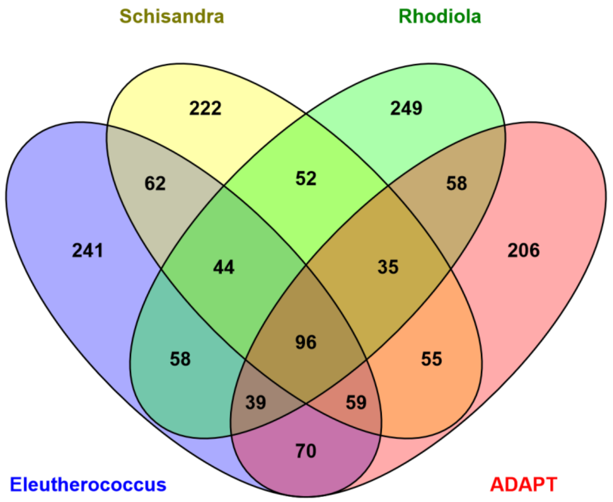

Synergy and antagonism: 3 component combination

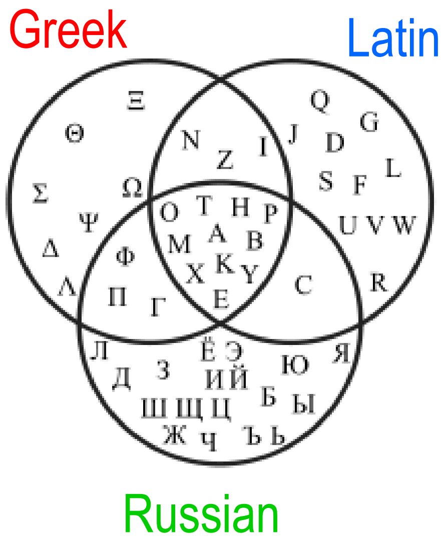

Intersections of Greek, Latin and Russian alphabets

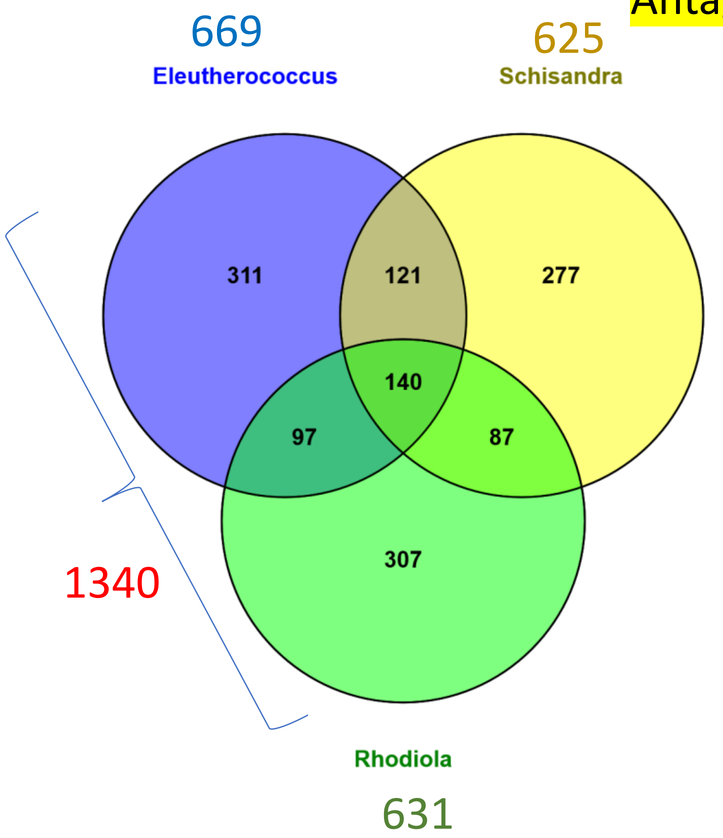

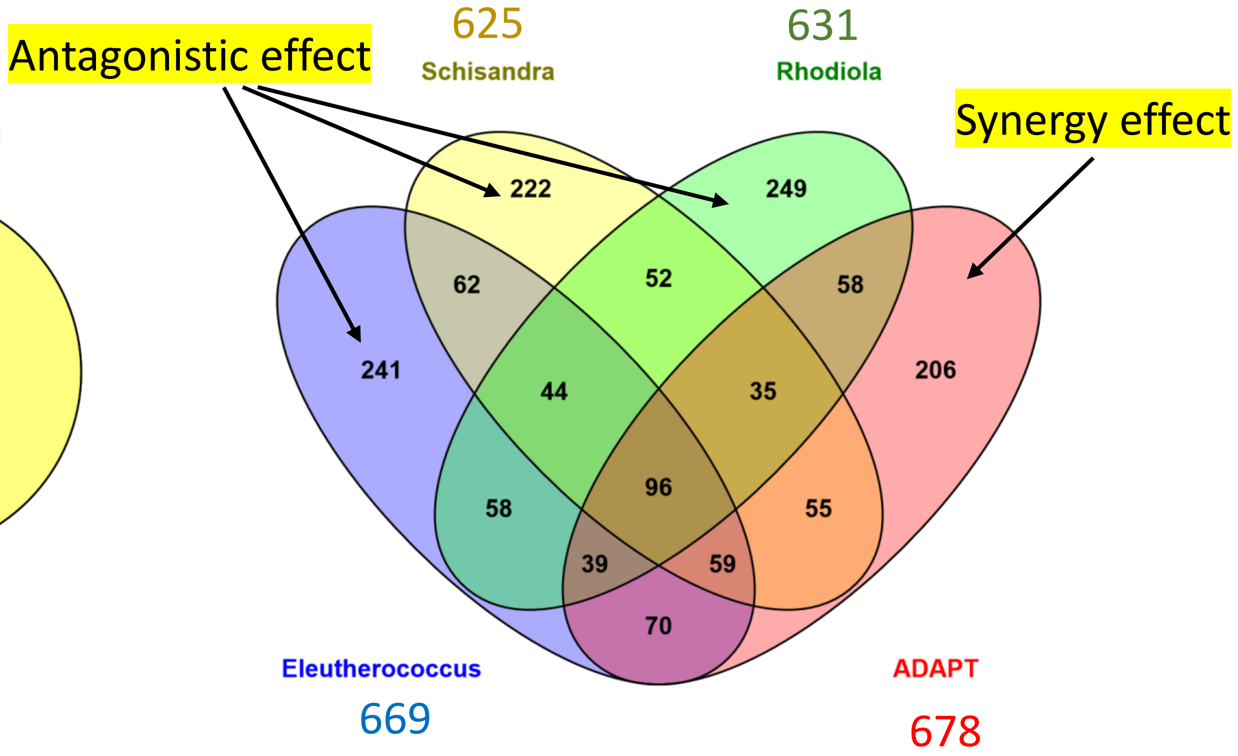

Signatures of deregulated genes of eicosanoids signalling pathways in neuroglia cells

Name
prostaglandin E receptor 3
phospholipase A2 group V
PG-endoperoxide synthase 2, COX-2
phospholipase A2 group VII
phospholipase A2 group IB
prostaglandin F receptor
phospholipase B1
thromboxane A2 receptor
phospholipase A2 group XVI
prostaglandin E receptor 2
dipeptidase 2
leukotriene C4 synthase
AA 5-lipoxygenase activating protein
prostacyclin (PGI2) synthase
arachidonate 12-lipoxygenase

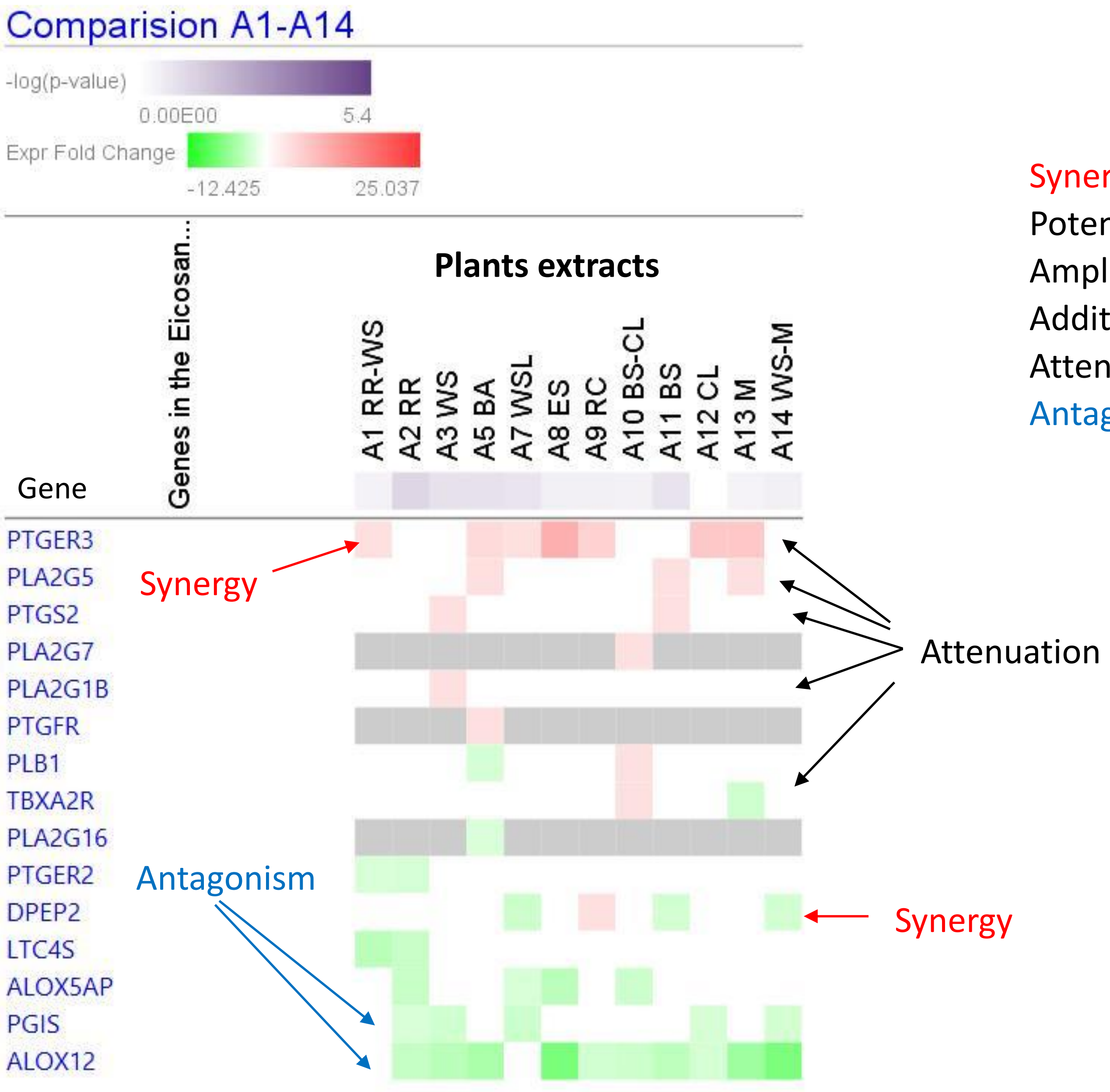

Synergy: 0+0>0

Potentiation: 0+1>1

Amplification: 1+1>2

Addition: 1+1=2

Attenuation: 1+1<2

Antagonism: 1+1=0

Black Cohosh Is More Effective in Combination with Golden Root for Relief of Menopausal Symptoms: A Randomized, Double-Blind, Placebo-Controlled Study

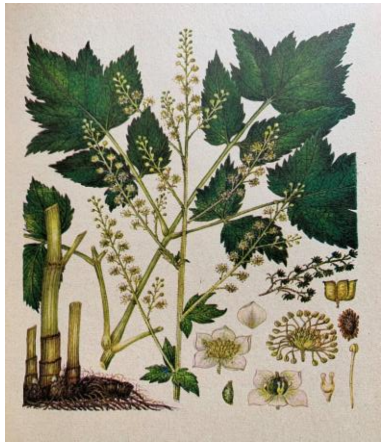

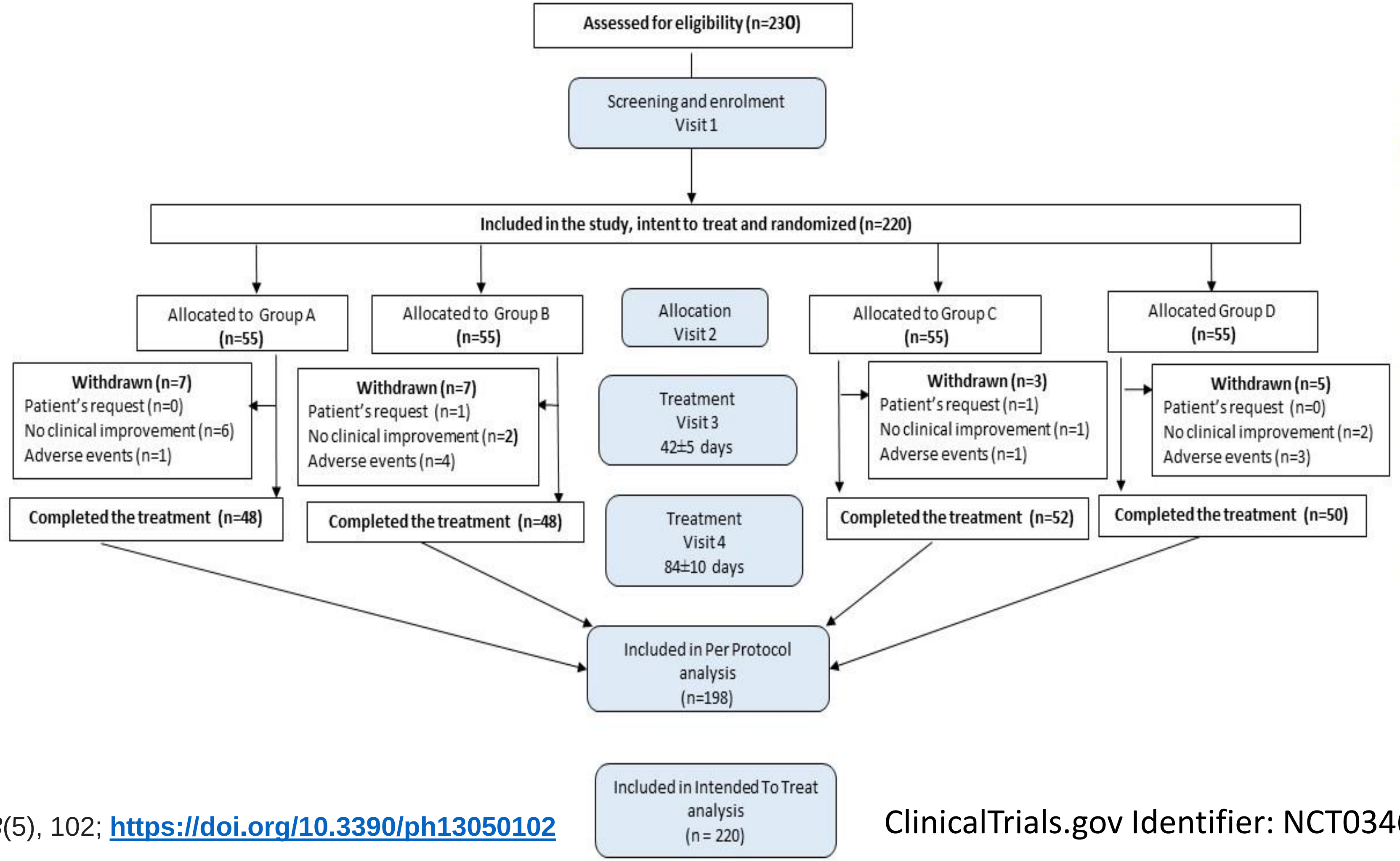

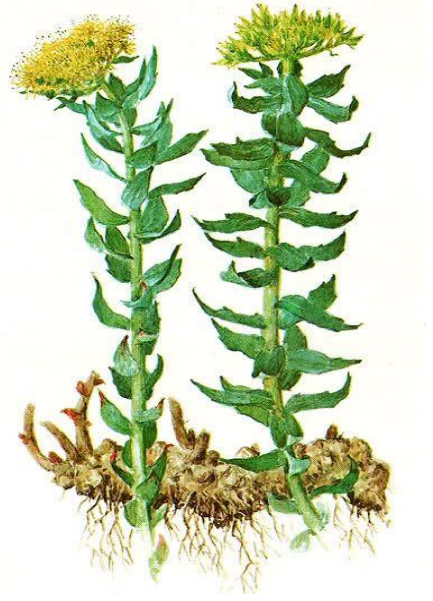

Pharmaceuticals **2020**, *13*(5), 102; https://doi.org/10.3390/ph13050102

ClinicalTrials.gov Identifier: NCT03461380

All Menopausal Symptoms:

- hot flushes, sweating, heart discomfort, sleep problems, joint and muscular discomfort;
- depressive mood, irritability, anxiety, physical, and mental exhaustion;
- sexual problems, bladder problems, and dryness of vagina.

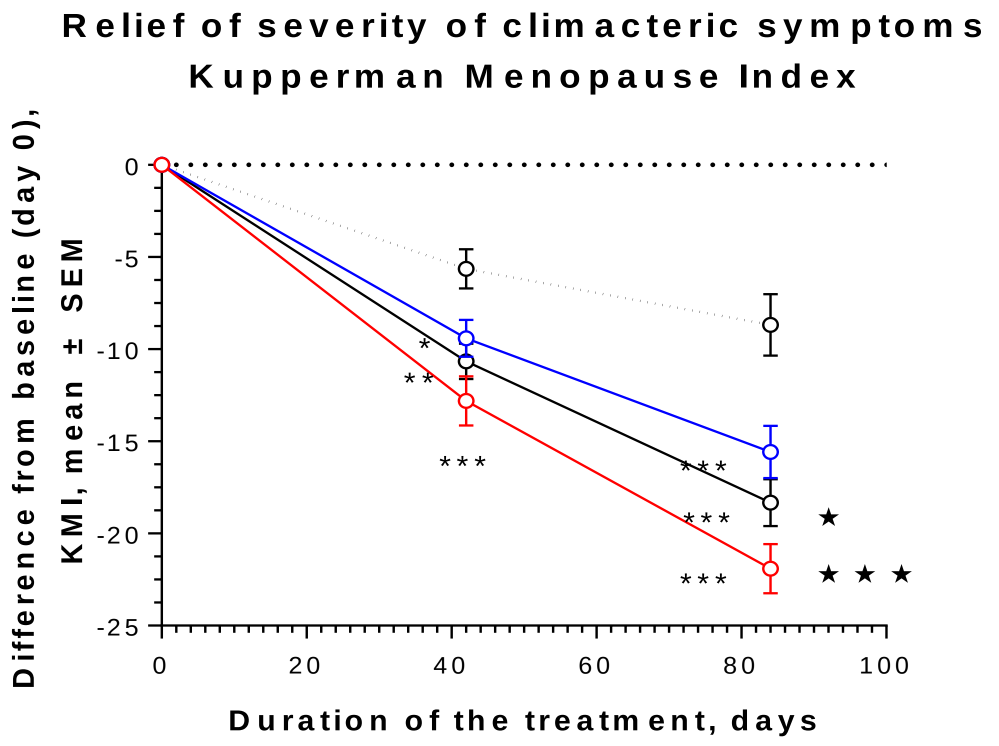

Group A, Placebo

Group B, Black Cohosh in low dose of 13 mg daily

Group C, Black Cohosh in high dose of 1000 mg daily

Group D, Rhodiola 400 mg + Black Cohosh 13 mg daily

Study Results: Hot flashes

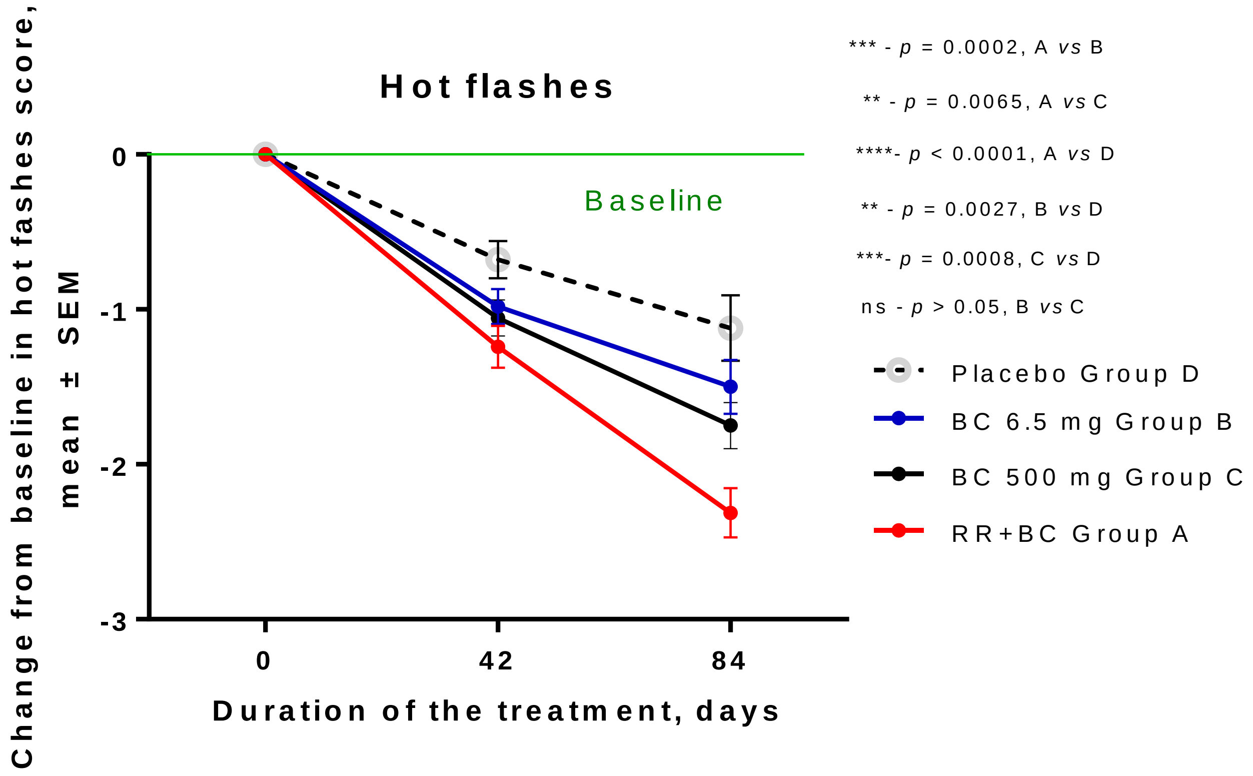

Group D, Placebo

Group B, Black Cohosh in low dose of 13 mg daily

Group C, Black Cohosh in high dose of 1000 mg daily

Group A, Rhodiola 400 mg + Black Cohosh 13 mg daily

Quality of life domains

- Occupational health
- Physical health
- Emotional health
- Sexual activity
- Total QOL

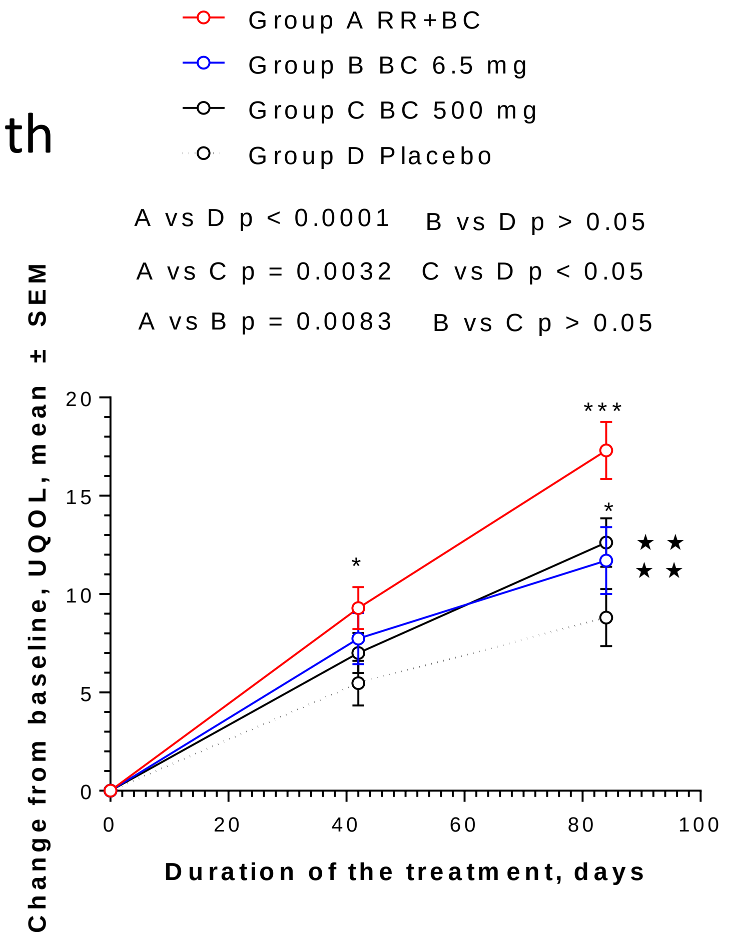

Utian Quality of Life Scale (UQOL) Scoring Summary

Instructions: Each of the four subscales of the UQOL is represented by a unique color, as shown below. Sum the circled responses by color and enter the sum in the scoring summary section at the bottom of the page.

1.	I am able to control things in my life that are important to me.	1	2	3	4	5
2.	I feel challenged by my work.	1	2	3	4	5
3.	I believe my work benefits society.	1	2	3	4	5
4.	I am not content with my sexual life.	5	4	3	2	1
5.	I am content with my romantic life.	1	2	3	4	5
6.	I have gotten a lot of personal recognition in my community or at my job.	1	2	3	4	5
7.	I am unhappy with my appearance.	5	4	3	2	1
8.	My diet is not nutritionally sound.	5	4	3	2	1
9.	I feel in control of my eating behavior.	1	2	3	4	5
10.	Routinely, I engage in active exercise three or more times each week.	1	2	3	4	5
11.	My mood is generally depressed.	5	4	3	2	1
12.	I frequently experience anxiety.	5	4	3	2	1
13.	Most things that happen to me are out of my control.	5	4	3	2	1
14.	I am content with the frequency of my sexual interactions with a partner.	1	2	3	4	5
15.	I currently experience physical discomfort or pain during sexual activity.	5	4	3	2	1
16.	I believe I have no control over my physical health.	5	4	3	2	1
17.	I am proud of my occupational accomplishments.	1	2	3	4	5
18.	I consider my life stimulating.	1	2	3	4	5
19.	I continue to set new personal goals for myself.	1	2	3	4	5
20.	I expect that good things will happen in my life.	1	2	3	4	5
21.	I feel physically well.	1	2	3	4	5
22.	I feel physically fit.	1	2	3	4	5
23.	I continue to set new professional goals for myself.	1	2	3	4	5

Scoring Summary

	Lower QoL -2SD	-1SD	Mean	+1SD	Higher QoL +2SD
Occupational QoL	13	19	25	31	35
Health QoL	11	16	21	26	31
Emotional QoL	12	16	20	24	28
Sexual QoL	0	4	8	12	15
Total QoL	48	61	74	87	100

Physical, emotional health and sexual activity QOL indexes

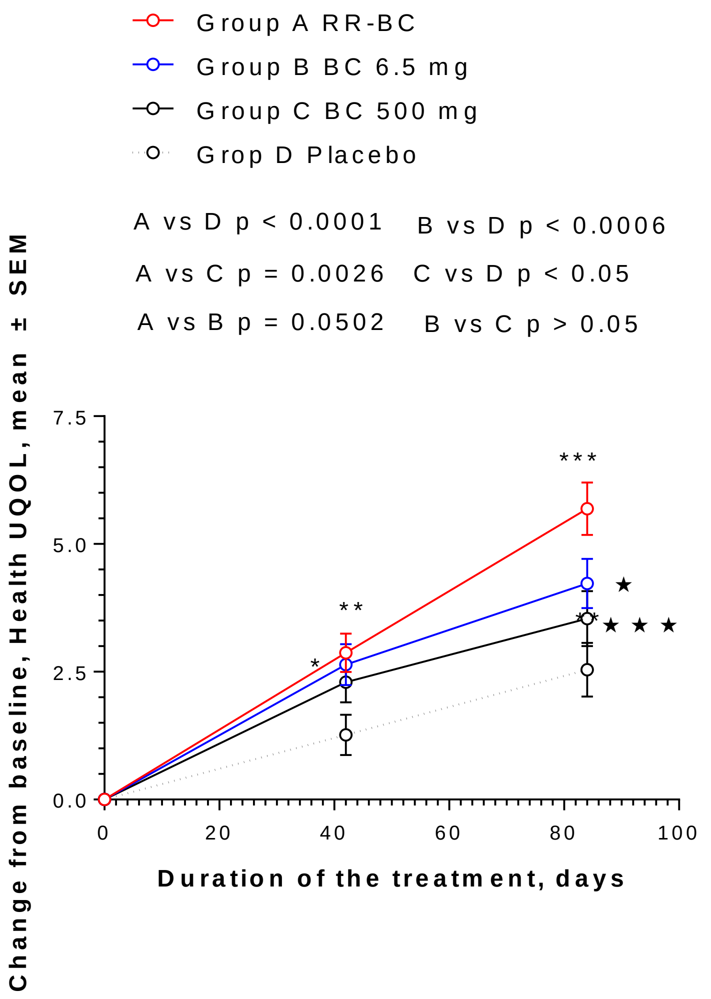

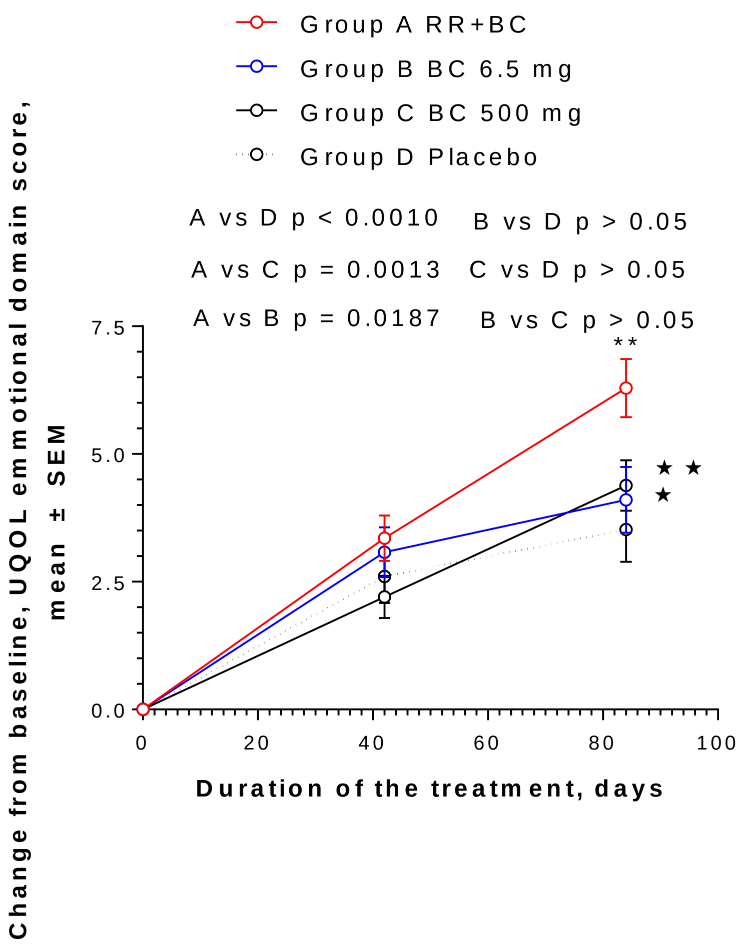

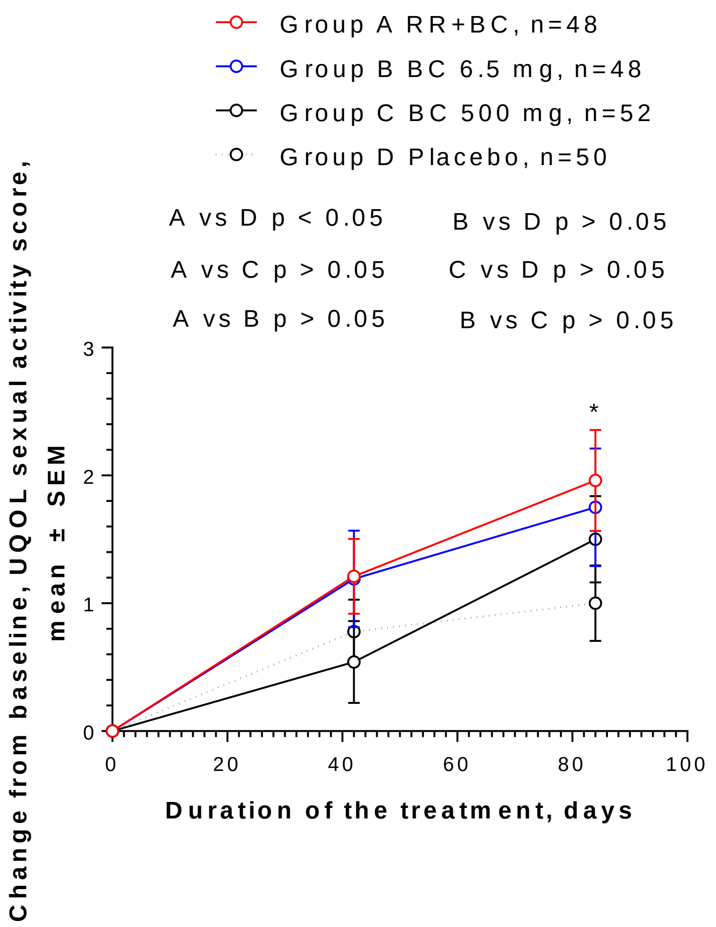

Efficacy of Adaptogens in Patients with Long COVID-19: A Randomized, Quadruple-Blind, Placebo-Controlled Trial

ADAPT-232: Rhodiola+Eletherococcus+Schisandra

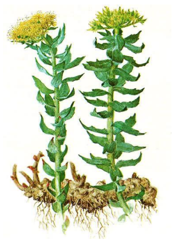

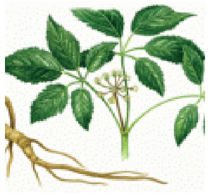

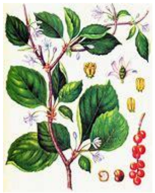

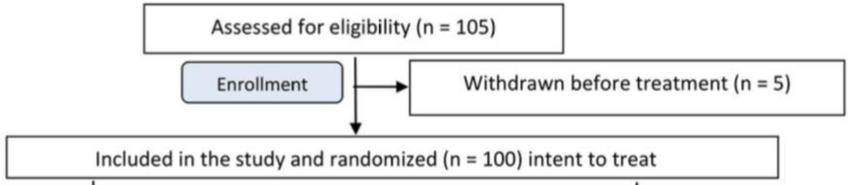

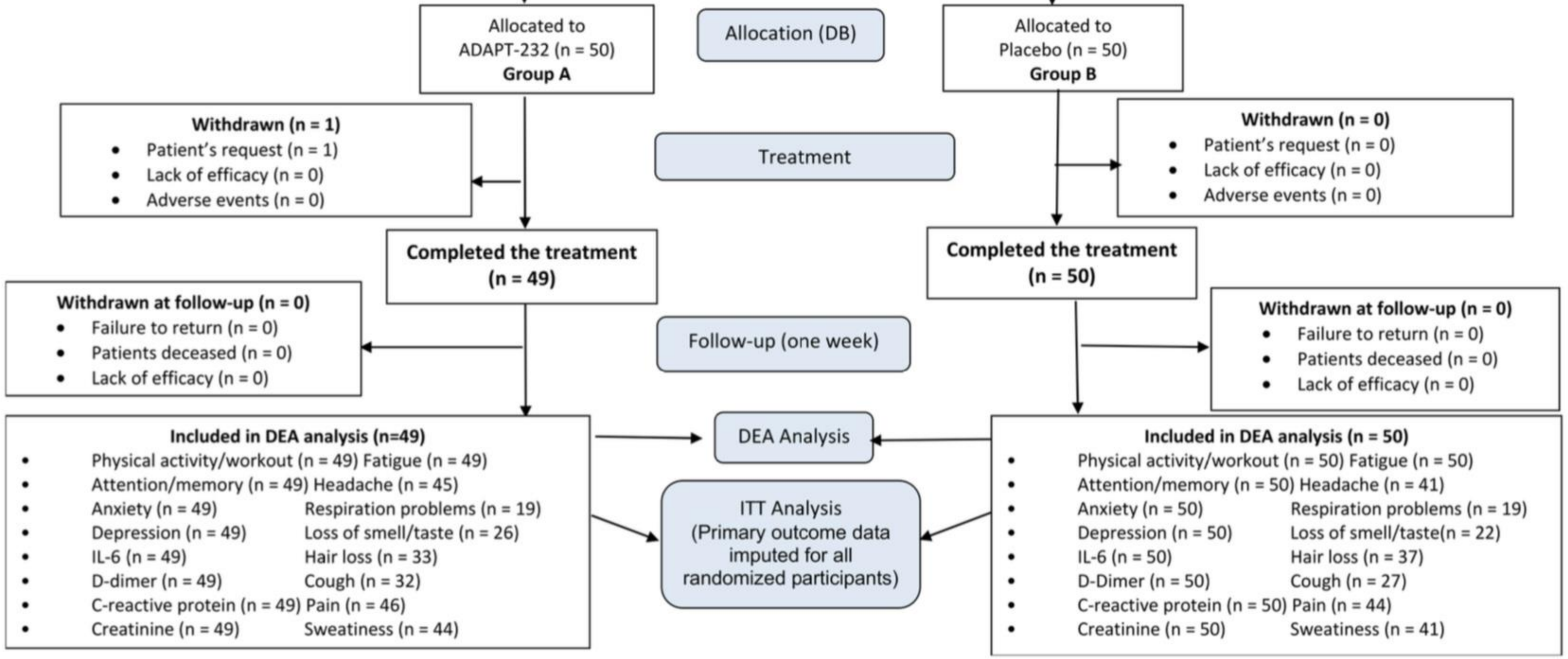

ClinicalTrials.gov Identifier: NCT04795557

Pharmaceuticals 2022, 15, 345. https://doi.org/10.3390/ph15030345

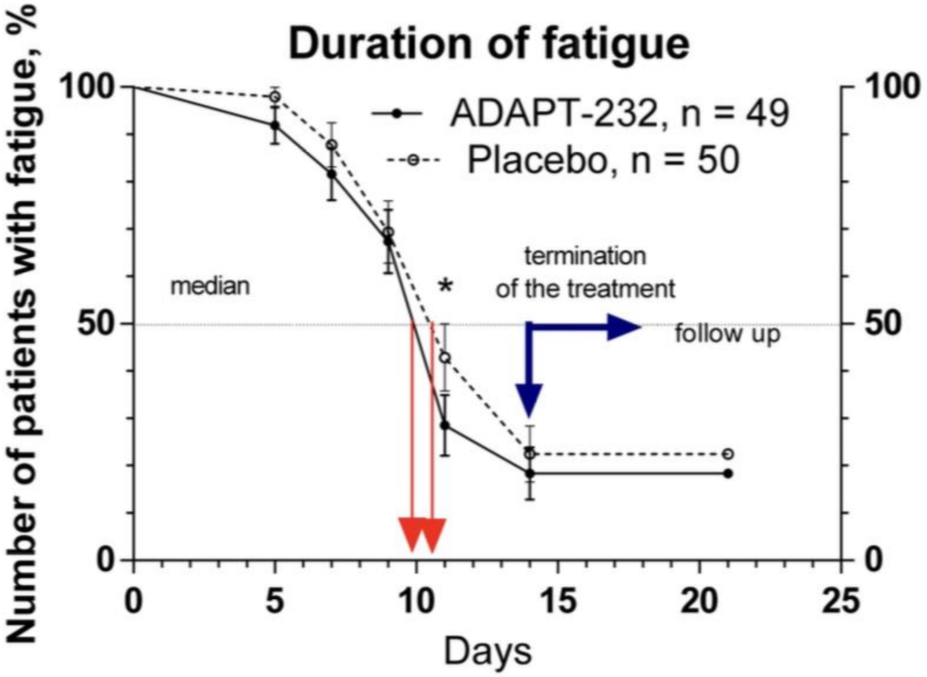

Duration of fatigue
Number of patients with fatigue, %
ADAPT-232, n = 49
Placebo, n = 50
median
termination
of the treatment
follow up
Days

(a)

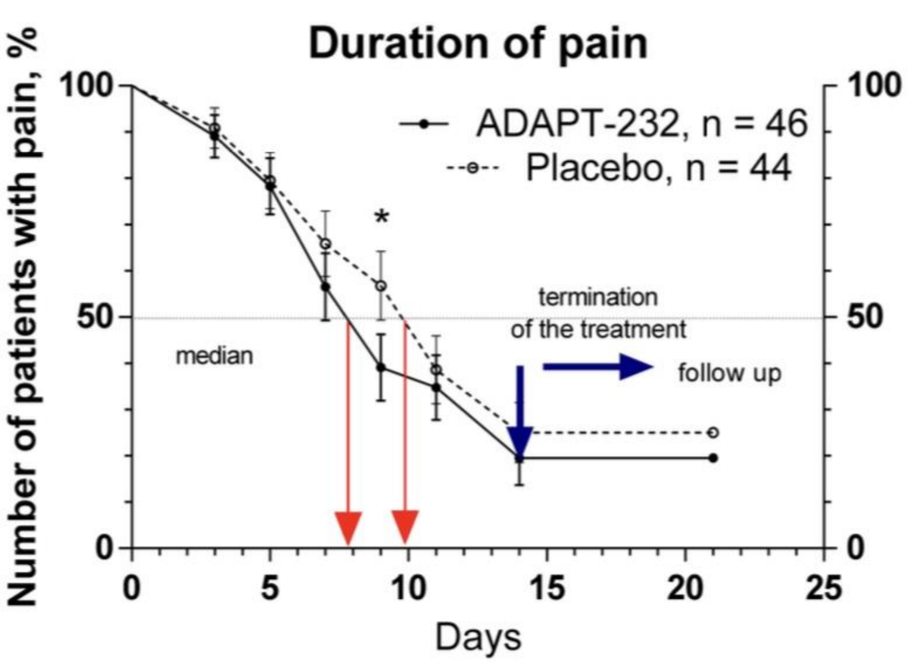

Duration of pain
Number of patients with pain, %
ADAPT-232, n = 46
Placebo, n = 44
median
termination
of the treatment
follow up
Days

(b)

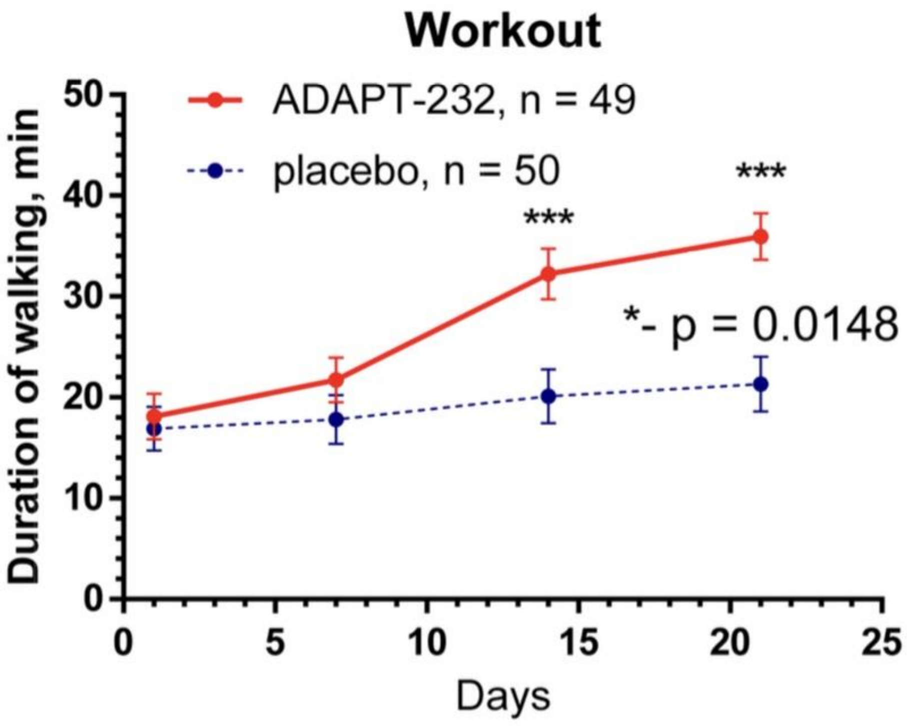
Workout
ADAPT-232, n = 49
placebo, n = 50
***
***
*- p = 0.0148
Duration of walking, min
50
40
30
20
10
0
0
5
10
15
20
25
Days

(a)

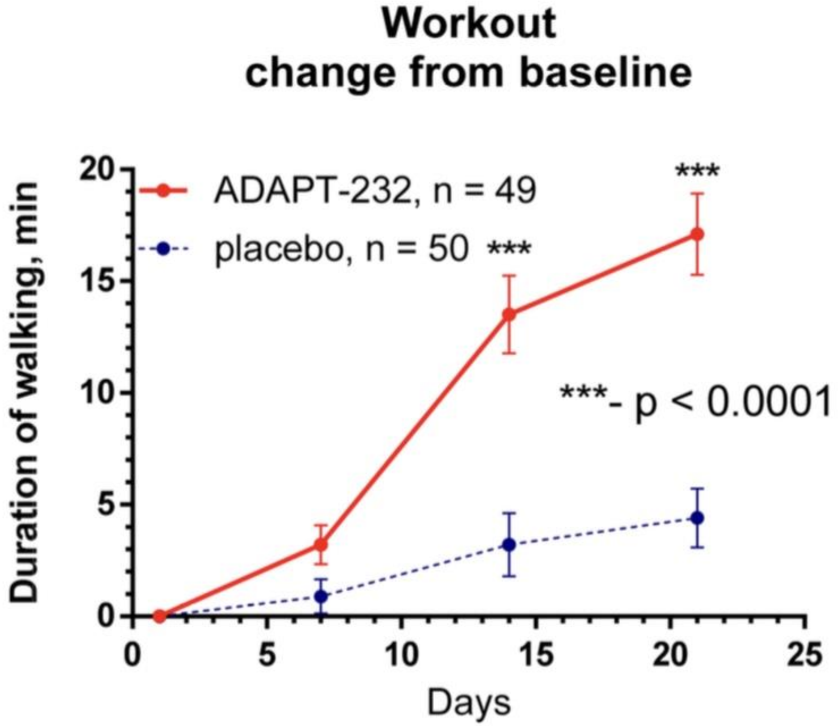
Workout
change from baseline
ADAPT-232, n = 49
placebo, n = 50
***
***
***- p < 0.0001
Duration of walking, min
20
15
10
5
0
0
5
10
15
20
25
Days

(b)

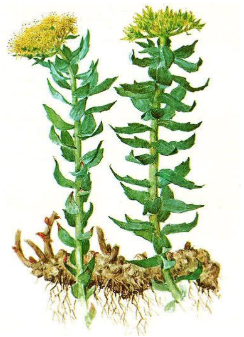

Mg-Teadiola: *Rhodiola rosea* L. + *Camelia chinensis* [L.] Kuntze + Mg + vitamins B6, B9, B12+L-theanine

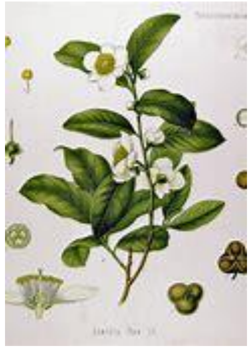

Published of controlled, randomized clinical studies	Study design Dosage form	Number of subject
Dye L, Billington J, Lawton C, Boyle N. **2020.** A combination of magnesium, B vitamins, green tea and rhodiola attenuates the negative effects of acute psychosocial stress on subjective state in adults. *Curr Dev Nutr*. 4:nzaa067_023.	DB-R-PC-PG, Capsules Tablets	25+25+25+25
Boyle NB, Billington J, Lawton C, Quadt F, Dye L. **2021.** A combination of green tea, rhodiola, magnesium and B vitamins modulates brain activity and protects against the effects of induced social stress in healthy volunteers. *Nutritional neuroscience*, 25(9), 1845–1859.	DB-R-PC-PG, Capsules Tablets	25+25+25+25
Boyle NB, Dye L, Lawton CL,Billington J**. 2022.**A Combination of Green Tea, Rhodiola, Magnesium, and B Vitamins Increases Electroencephalogram Theta Activity During Attentional Task Performance Under Conditions of Induced Social Stress. *Frontiers in nutrition*, 9, 935001.	DB-R-PC-PG, Capsules Tablets	25+25+25+25
Noah L, Morel V, Bertin C, Pouteau E, Macian N, Dualé C, Pereira B, Pickering G. **2022.** Effect of a Combination of Magnesium, B Vitamins, Rhodiola, and Green Tea (L-Theanine) on Chronically Stressed Healthy Individuals-A Randomized, Placebo-Controlled Study. *Nutrients,* 14(9), 1863.	SB-R-PC-PG, Tablets	49+51
Pickering G, Noah L, Pereira B, Goubayon J, Leray V, Touron A, Macian N, Bernard L, Dualé C, Roux V, Chassain C. **2023.** Assessing brain function in stressed healthy individuals following the use of a combination of green tea, Rhodiola, magnesium, and B vitamins: an fMRI study. *Frontiers in nutrition*10:1211321	SB-R-PC-PG, Tablets	20+20

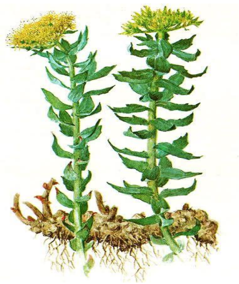

Mg-Teadiola: *Rhodiola rosea* L. + *Camelia chinensis* [L.] Kuntze + Mg + vitamins B6, B9, B12+L-theanine

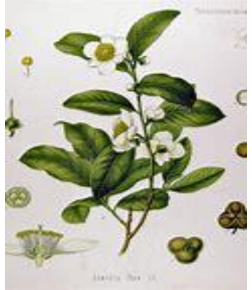

Clinical studies	Subjects	Experimental Stress	Dosage Mg-Teadiola	Outcome measures
Dye et al., **2020**	Healthy, moderately stressed (DASS score: 13–25)	Acute, TSST	Single dose	Subjective stress (Stress and Arousal), Mood (Profile of Mood States)
Boyle et al., **2021**	Healthy, moderately stressed (DASS score: 13–25)	Acute, TSST	Single dose	Spectral theta brain activity associated with cognitive task performance. Subjective stress (Stress and Arousal), Mood (Profile of Mood States) Salivary cortisol, Cardiovascular parameters (BP, HRV)
Boyle et al., **2022**	Healthy, moderately stressed (DASS score: 13–25)	Acute TSST	Single dose	Spectral theta brain activity, attentional capacity
Noah et al., **2022**	Healthy, moderately stressed (DASS score: >14)	none	Repeated for 4 weeks	Stress, Anxiety, Depression, Sleep, Cortisol
Pickering et al., **2023**	Healthy, moderately stressed (DASS score: >14)	Thermal stimulation	Repeated for 4 weeks	blood-oxygen-level-dependent (BOLD) signal, stress, anxiety, depression, and sleep, cortisol

DASS - The Depression Anxiety Stress Scale-42 questionnaire assessing the negative emotional states over the last week: Stress and pain scores were categorized as 0–14 “normal”, 15–18 “mild”, 19–25 “moderate”, 26–33 “severe”, or 34+ “extremely severe”.
TSST - Acute stress was experimentally induced by The Trier Social Stress Test (TSST): Speech and mental mathematics tasks.

Single dose effect of Mg-Teadiola on experimentally induced by The Trier Social Stress Test (TSST): Speech and mental mathematics tasks.

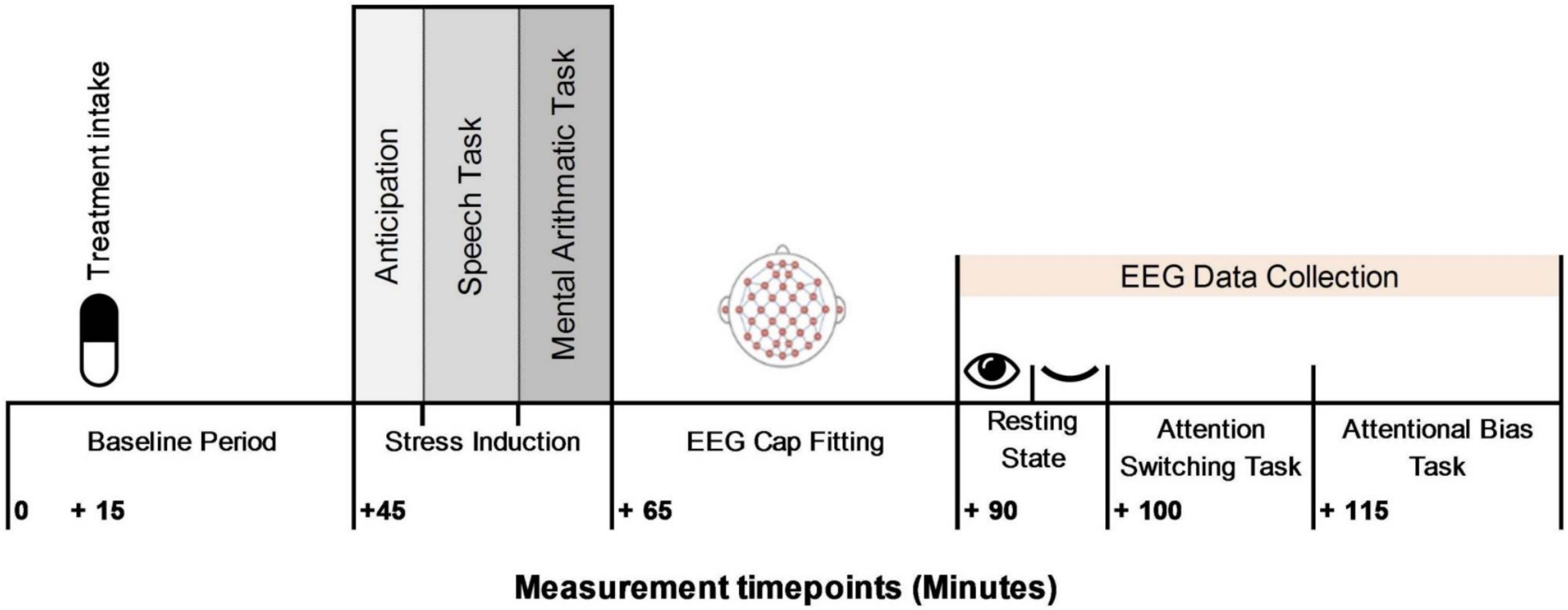

Single dose effect of Rhodiola, Green tea and Mg-Teadiola on experimentally induced by The Trier Social Stress Test

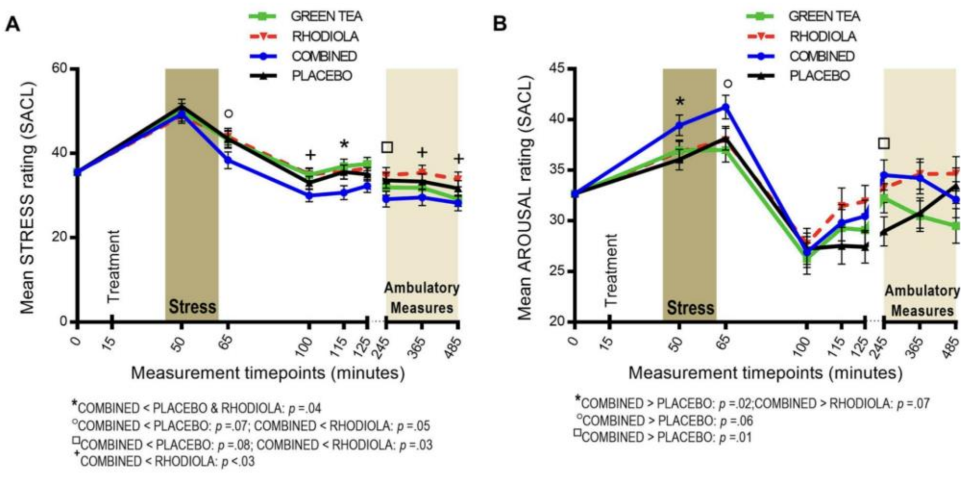

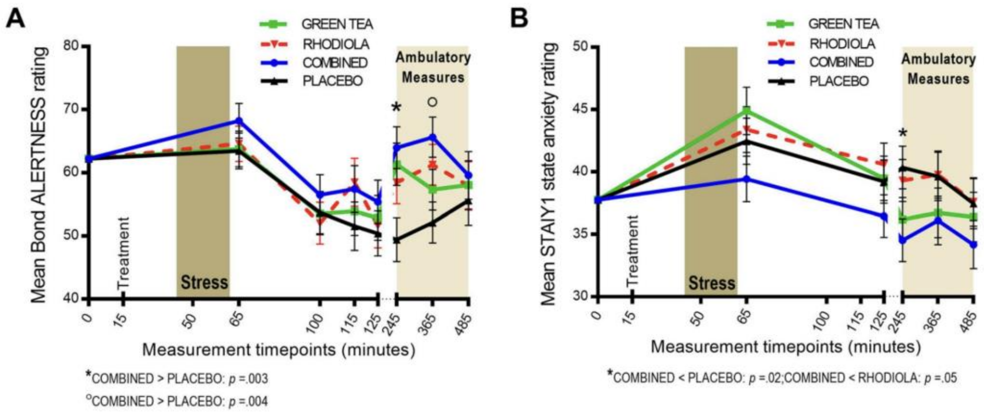

Figure 6. Response profiles for: (A) Bond-Lader Alertness; (B) STAI state anxiety.

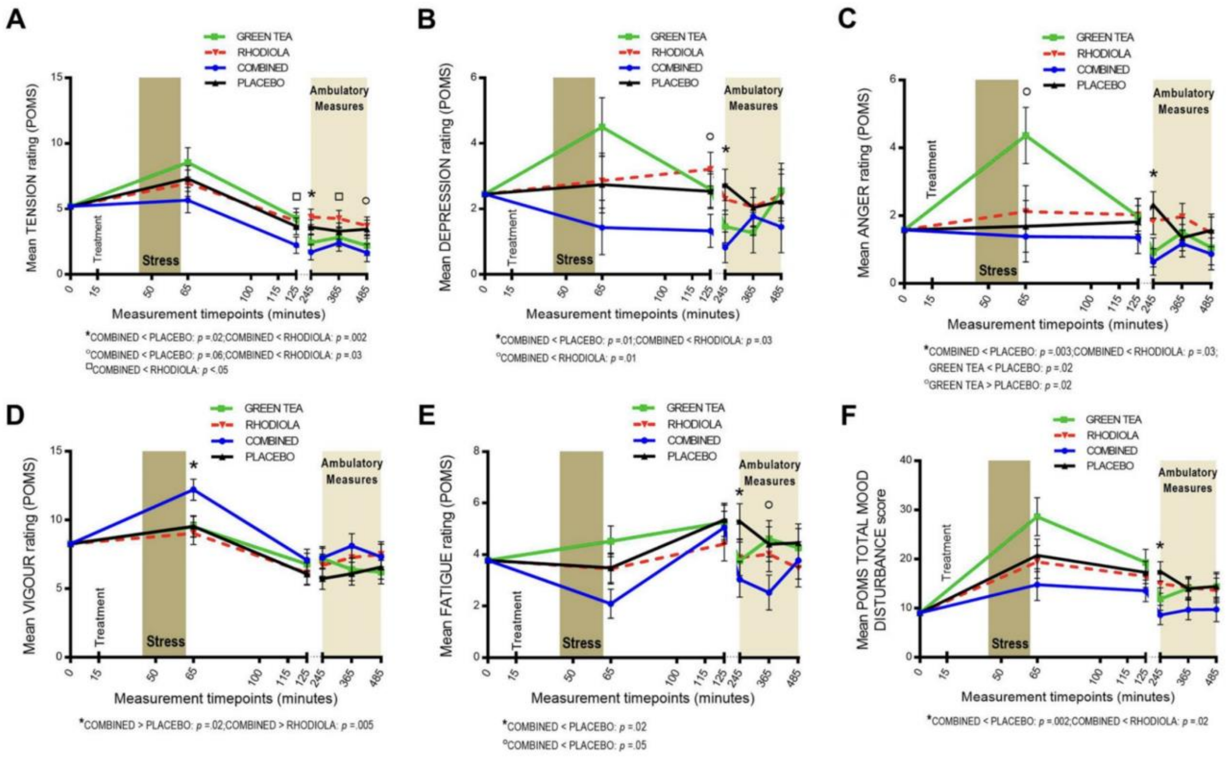

Figure 5. POMS subscale response profiles: (A) Tension-Anxiety; (B) Depression-Dejection; (C) Anger-Hostility; (D) Vigour-Acti[illegible] (E) Fatigue-Inertia; (F) Total Mood Disturbance score.

Repeated doses effect of Mg-Teadiola on chronically stressed healthy subjects

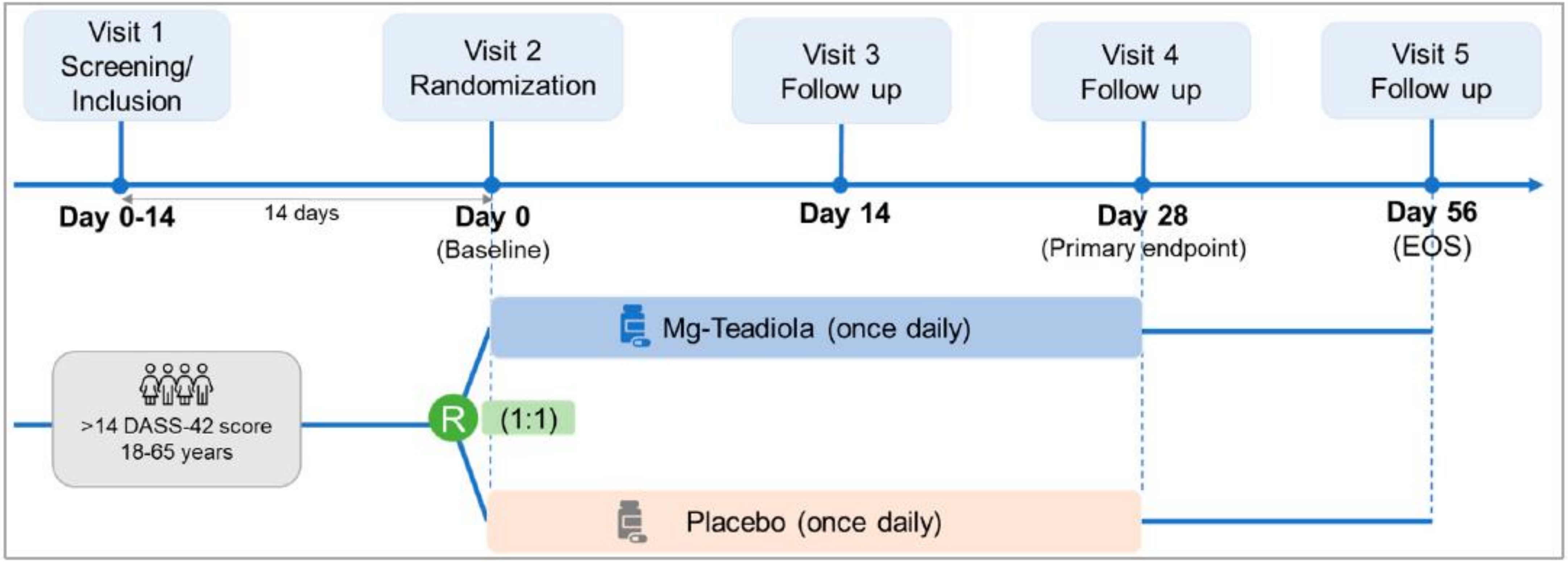

Mg-Teadiola increased EEG resting state theta (p < .02) – considered indicative of a relaxed, alert state, attenuated subjective stress, anxiety and mood disturbance

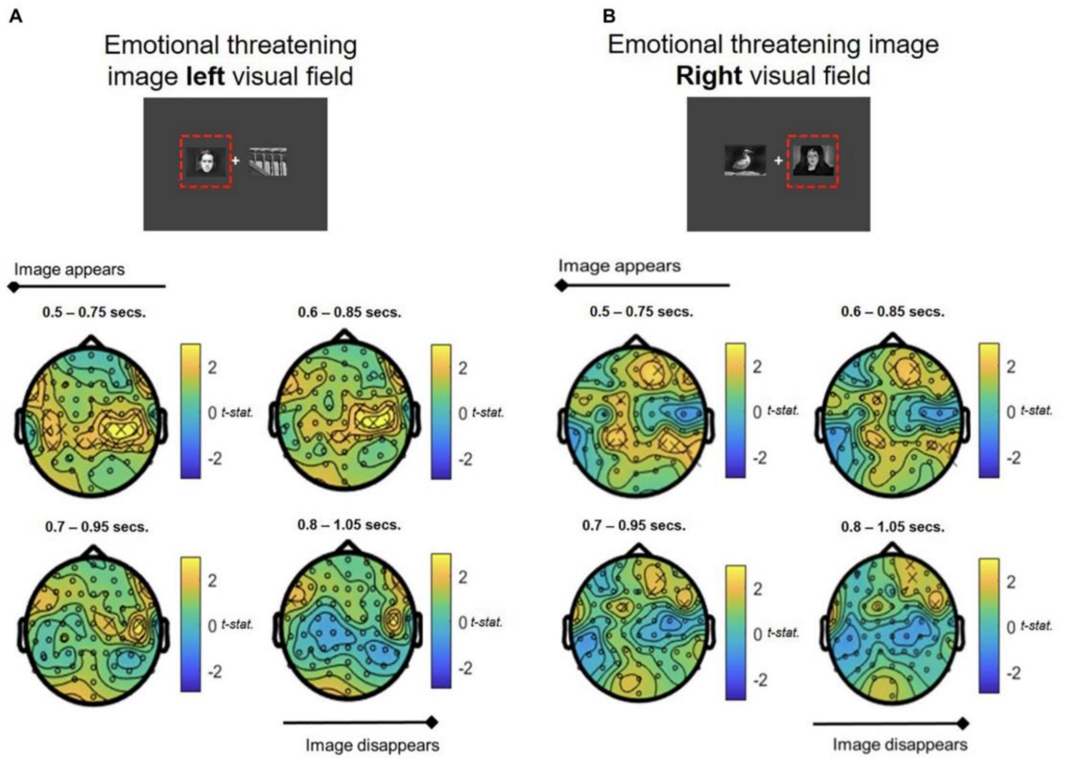

FIGURE 7 | Theta band power differences between COMBINED and PLACEBO treatments during presentation of emotionally threatening and neutral images [illegible] stress images shown in **(A)** LEFT and **(B)** RIGHT visual fields. Values > 0 indicate COMBINED treatment theta values are higher than PLACEBO treatment. Significant electrodes ($p < 0.05$) marked with an *x*. Each figure depicts a series of *T*-tests along the time course of the presentation of the images (0.5–1.05 s, [illegible] 0.1 s steps, 0.25 s sample widths).

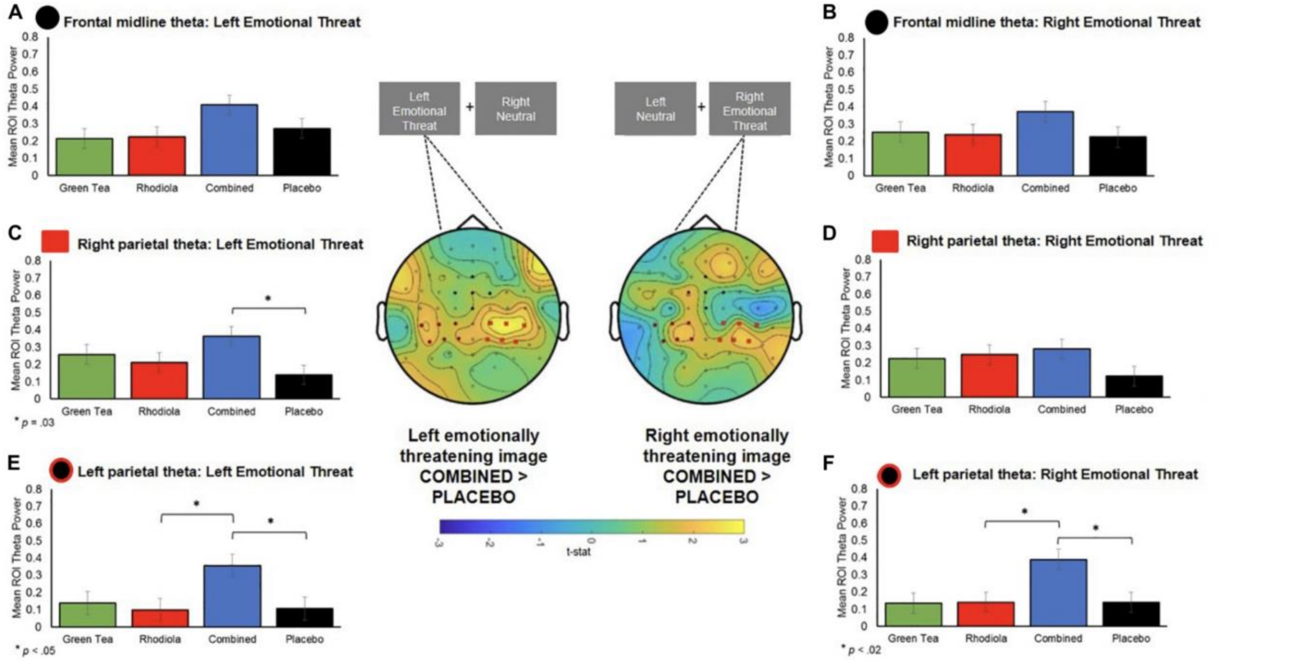

FIGURE 8 | Central figure: T-statistic maps of spectral theta activity difference between COMBINED and PLACEBO treatments 0.5–0.75 s after presentation of the images, distinguished by left and right emotionally threatening image presentation. Positive values in maps indicate COMBINED > PLACEBO. ROI electrodes symbols shown on maps and in respective summary bar chart. **(A,B)** Theta band activity in *a priori* defined ROI midline frontal region for left and right presented images. **(C,D)** RIGHT parietal *a priori* defined ROI theta activity when emotionally threatening image shown on left and right. **(E,F)** LEFT parietal *a priori* defined ROI theta activity when emotionally threatening image shown on left and right.

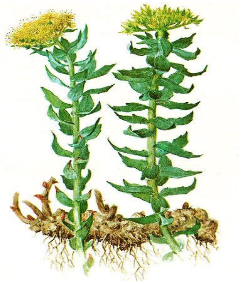

Mg-Teadiola: *Rhodiola rosea* L. + *Camelia chinensis* [L.] Kuntze + Mg + vitamins B6, B9, B12+L-theanine

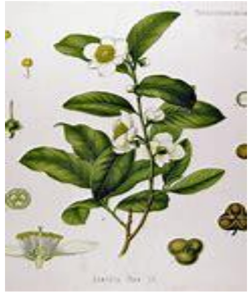

Clinical studies	Conclusions
Dye et al., **2020**	Mg-Teadiola significantly alleviated subjective stress and mood responses to acute stress provocation. This preliminary evidence suggests the capacity of these ingredients in combination to confer protective effects under conditions of stress in adults.
Boyle et al., **2021**	The combined treatment significantly increased EEG resting state theta – considered indicative of a relaxed, alert state, attenuated subjective stress, anxiety and mood disturbance, and heightened subjective and autonomic arousal; Mg-Teadiola may enhance coping capacity and offer protection from the negative effects of stress exposure.
Boyle et al., **2022**	The combination of Mg + B vitamins + green tea + rhodiola increased spectral theta brain activity during the execution of two attentional tasks suggestive of a potential to increase attentional capacity under conditions of stress
Noah et al., **2022**	Mg-Teadiola was effective in relieving stress on Days 14 and 28 in chronically stress and may diminish pain perception, underlines its potential benefits for patients suffering from pain, in whom comorbidities such as stress and sleep disorders are frequent.
Pickering et al., **2023**	Supplementation with Mg-Teadiola reduced stress on D28 in chronically stressed but otherwise healthy individuals and modulated the stress and pain cerebral matrices during stressful thermal stimulus.

Shortcomings of the studies

- The authors declare the labeled amount of active ingredients but do not adhere to CONSORT regarding the quality of the product, which is not adequately characterized in respect of:
  - extraction solvents, - ?
  - dry herb: dry native Extract Ratio (DER), - ?
  - the content of active markers (caffeine, theanine, Mg+2, salidroside, Rosavin, etc.) -???
  - the analytical methods validated for selectivity, accuracy, and procession and providing TLC and HPLC fingerprints to ensure reproducible quality and reproducible pharmacological activity.
- The placebo and Mg-Teadiola were distinguishable by appearance. Reporting the masking procedure is not convincing to ensure adequate double-blind study design.
- The content of magnesium and caffeine contained in Green Tea was uncontrolled and not specified; that is no guarantee that the effect of Mg-Teadiola provides consistently reproducible efficacy.
- Rhodiola and Green Tea products are known as herbs of significant variability depending on numerous factors.
- Finally, there is no sense in combining Rhodiola with caffeine, which is known to induce addiction and other undesired effects, unlike adaptogens. In this context, the rationale of the formulation does not stand up to scrutiny.

The difference between stimulants and adaptogens

	Stimulants	Adaptogens
Stress protective (neuro-, hepato-, cardio-protective)	No	High
Recovery process after exhaustive physical load	Low	High
Energy depletion	Yes	No
Performance in stress	-	Increased
Survival in stress	-	Increased
Quality of arousal	Poor	Good
Addiction potential	Yes	No
Side effects	Yes	Rare
DNA/proteins synthesis	Decreased	Increased
NPY mediated activation of Hsp70	-	Increased

Critical conclusions of EMA/HMPC/24186/2023 from assessment report on Rhodiola rosea L., rhizoma et radix

- The published clinical trials exhibit considerable deficiencies in their quality and show methodological problems.
- The results from trials on clinical pharmacology are contradictory.
- There is a lack of independent replications of the single different studies.
- Therefore ‘well-established use’ cannot be supported in the monograph

EMA assessor's rejection criteria of clinical relevance of studies

- insufficiently characterised herbal preparations,
- open (label) studies,
- small sample size,
- missing ITT analysis, regardless of detailed description of dropouts and reasons of excluded in analysis of a outcome measure,
- healthy subjects,
- efficacy score not validated.

The author's comment: the EMA assessment report is not free of shortcomings, including inconsistent assignments, classification and exclusion of some studies, unjustified assessor's comments and conclusions, and misinterpretation of the results of some publications.

Quality of Randomized Controlled Trials of Rhodiola Species

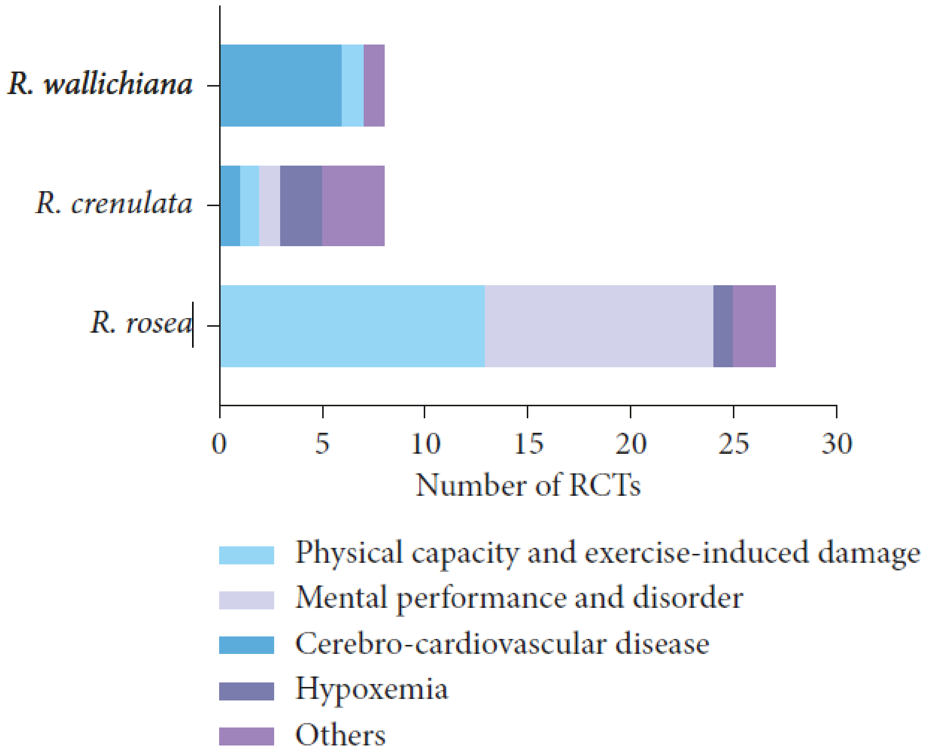

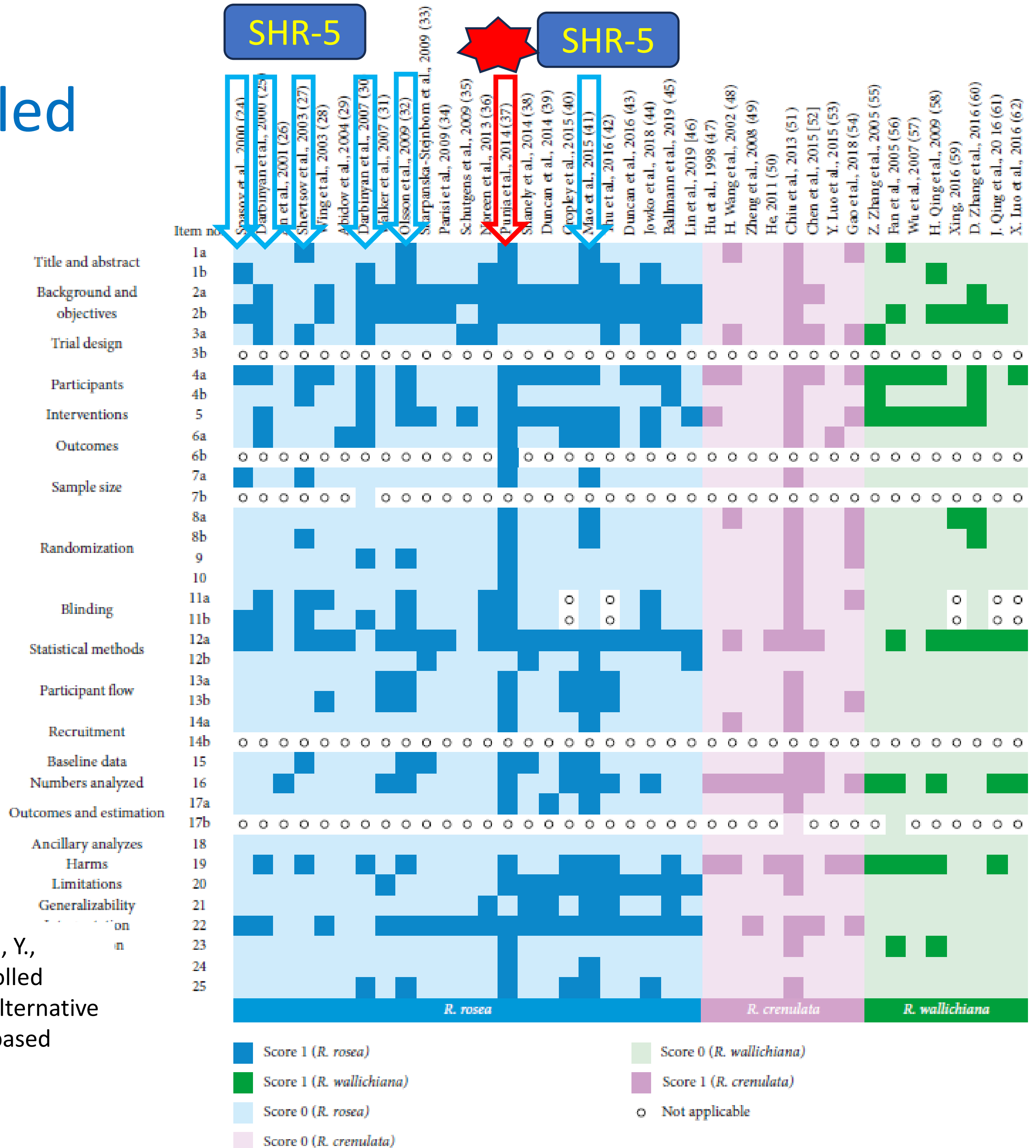

Li, X., Chen, W., Xu, Y., Liang, Z., Hu, H., Wang, S., & Wang, Y. (2021). Li, X., Chen, W., Xu, Y., Liang, Z., Hu, H., Wang, S., & Wang, Y. (2021). Quality Evaluation of Randomized Controlled Trials of Rhodiola Species: A Systematic Review. Evidence-based complementary and alternative medicine : eCAM, 2021, 9989546. https://doi.org/10.1155/2021/9989546. Evidence-based complementary and alternative medicine : eCAM, 2021, 9989546. https://doi.org/10.1155/2021/9989546

The CONSORT scores of RCT of Rhodiola Species

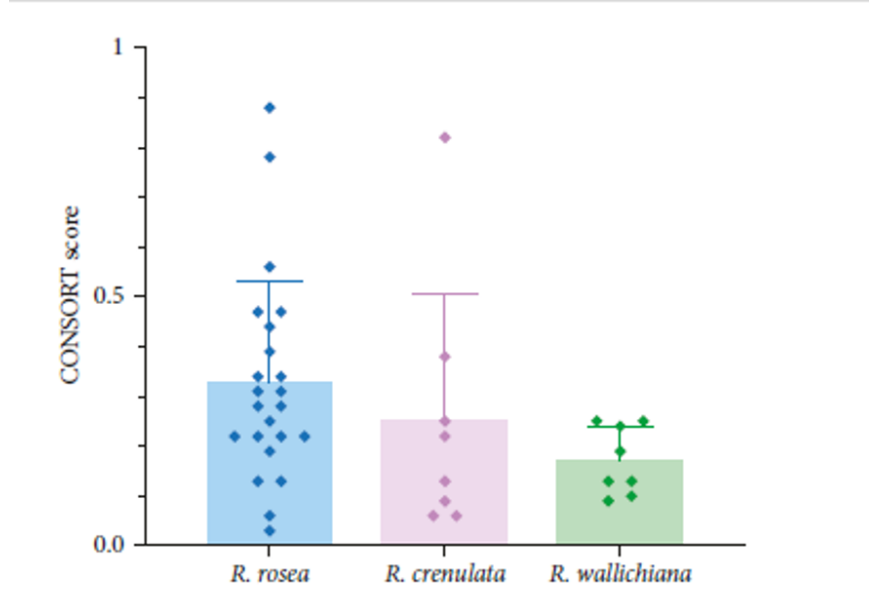

Risk of bias assessment

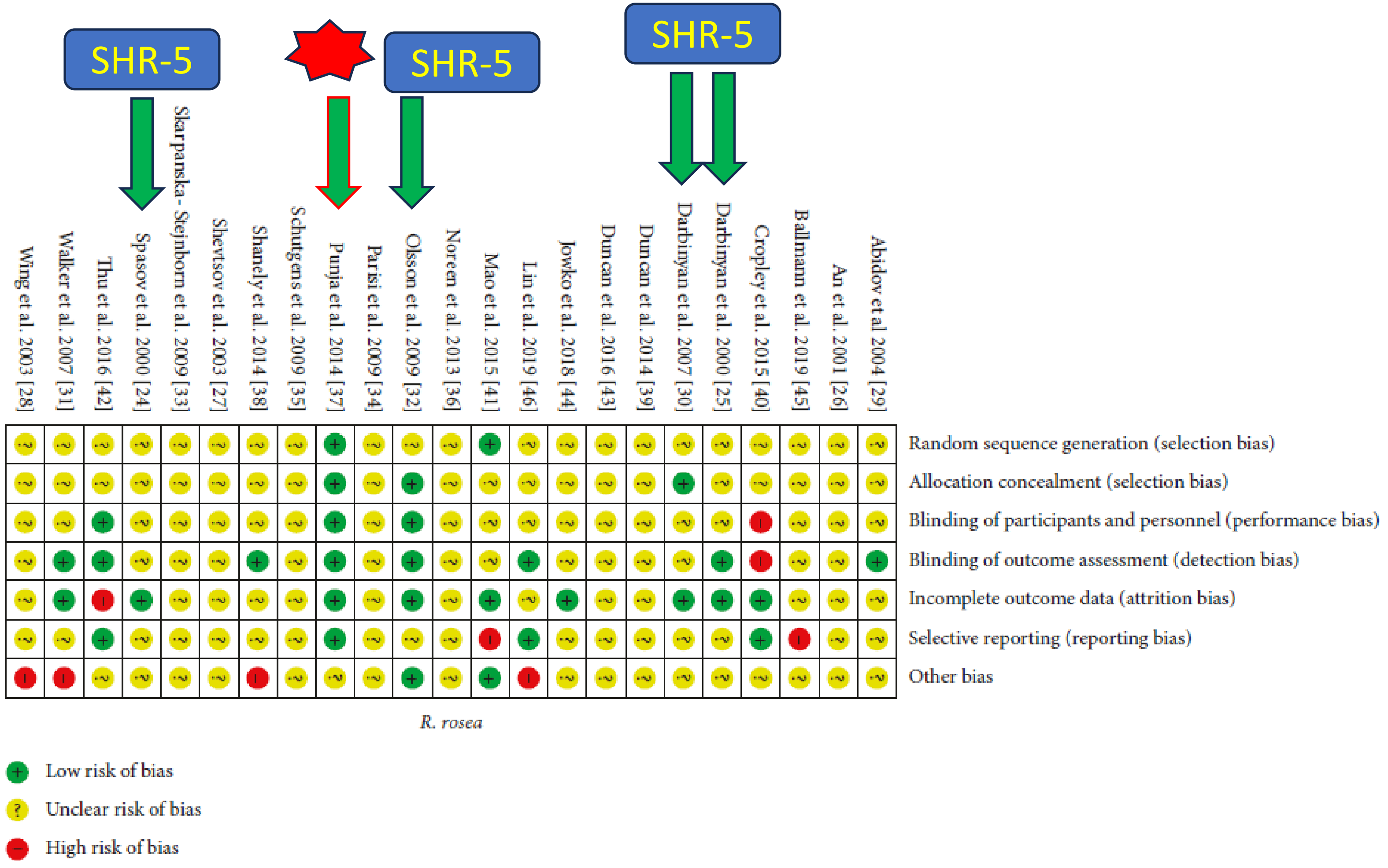

Possible sources of inconsistency of the results of various studies of *Rhodiola rosea*

- The authors concluded: *This study indicates that among nursing students on shift work, a 42-day course of R. Rosea compared with placebo worsened fatigue.*
- The authors declared the labeled amount of a powdered extract standardized to 2.8% of total rosavins, in the daily dose of 364 mg/day, but have not adhered to CONSORT regarding the quality of the Herbal Interventions, which was not adequately characterized in respect of:
  - extraction solvents, and dry herb: dry native Extract Ratio (DER),
  - the content of active markers, providing HPLC fingerprints to ensure consistent quality and reproducible pharmacological activity.
  - The analytical methods were not validated for selectivity, accuracy, and precision.
- The authors declared that placebo capsules containng microcrystalline cellulose and silicon dioxide had the same appearance, odour, and taste as the R. rosea product that is very unlikely due to their strong specific rose odour, test and color, particularly when “*participants were asked to self-determine their need for one additional capsule (i.e., a half dose), to be taken within four hours of the initial dose*.
- The authors have not reported (or not assessed) the results of treatment compliance (counting of unused tablets) and that is a serious flaw.
- All outcome measures of the study were subjective based on self-assessment questionnaires of QOL in 48 nurses instead of the only doctor having the same unified “standard.”
- The imbalance between Rhodiola treatment and placebo groups in medication use, and physical and emotional health problems have had a significant impact on the results of the study.

Lotaustralin in Rhodiola extract detected by LC/MS or LC/DA

Representative Chromatogram of *Rhodiola rosea* rhizome extract detected with positive mode electrospray **SCAN** mass spectrometry.

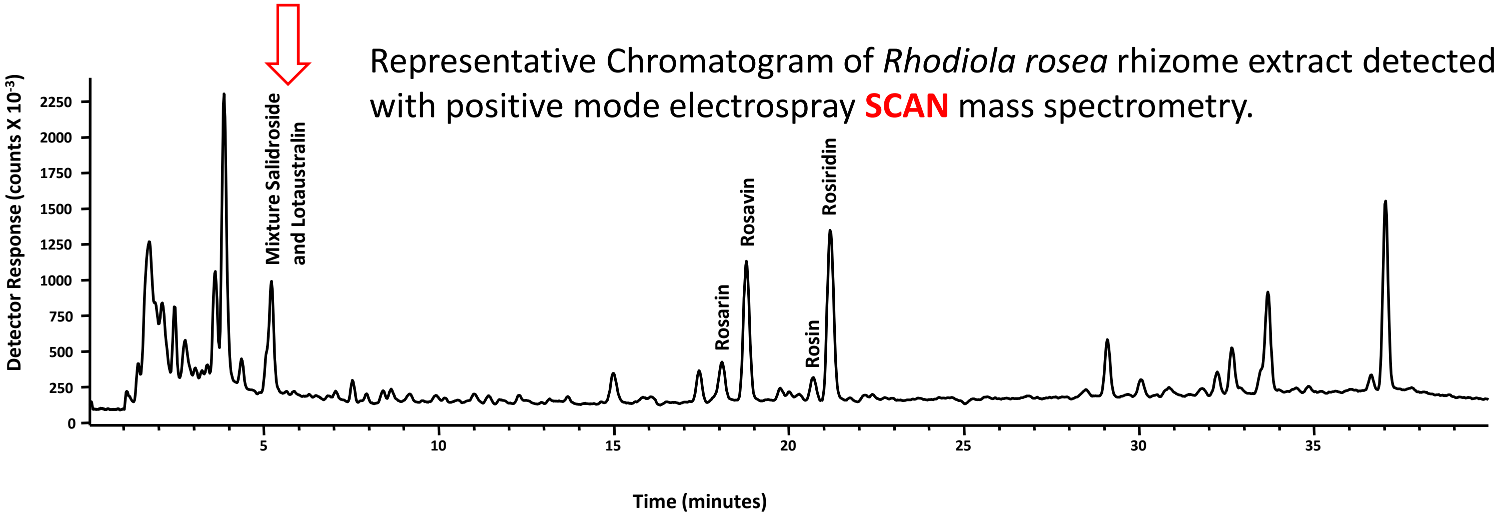

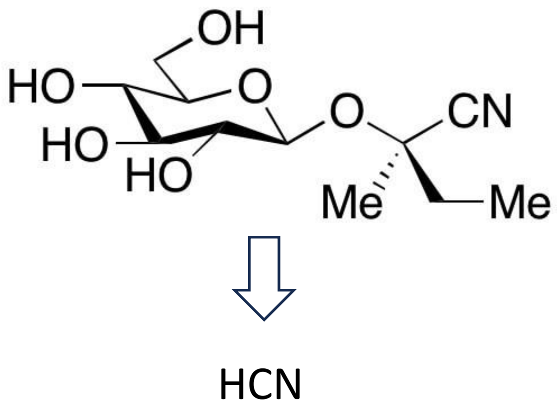

Representative Chromatogram of *Rhodiola rosea* rhizome extract detected with positive mode electrospray **SIM** mass spectrometry.

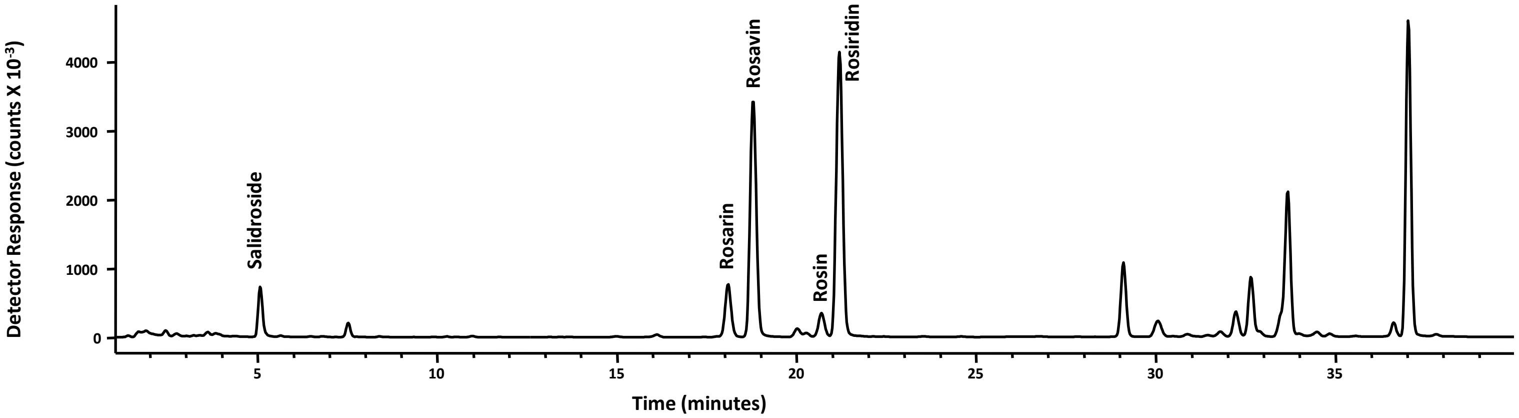

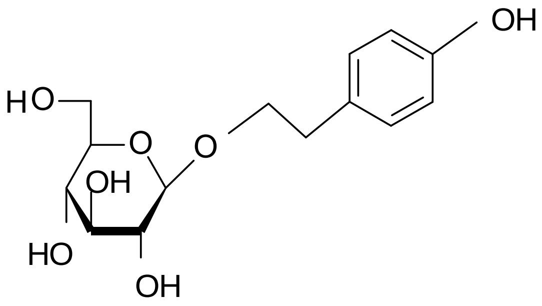

- Adapted from: Semple HA, NHP Research Targeted Toward Commercialization: Application of the Field to Medicine Cabinet Concept. 7th NHPRS Research Conference, Halifax, Canada, May 23-26, 2010

Variability of HPLC fingerprints of phenylpropanoids in so called ”Total Rosavins”

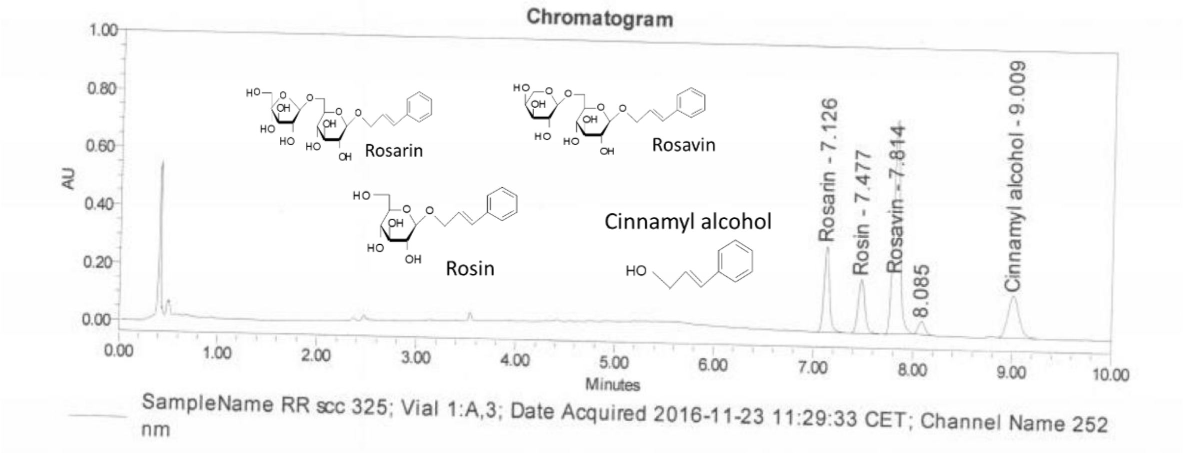

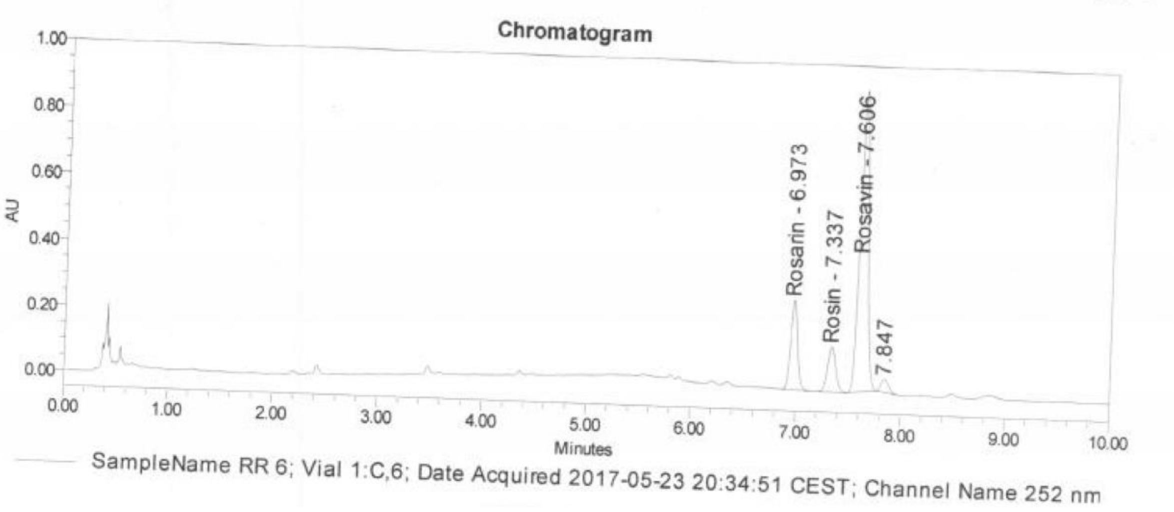

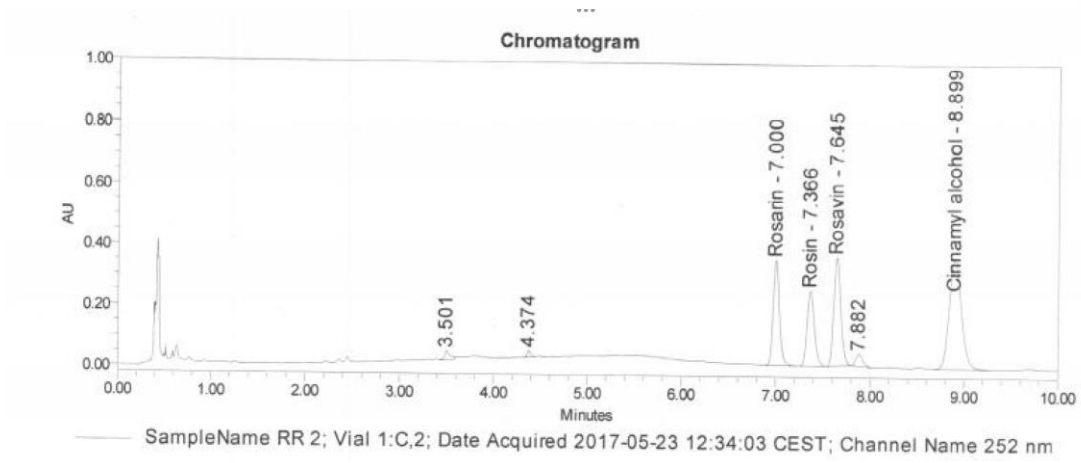

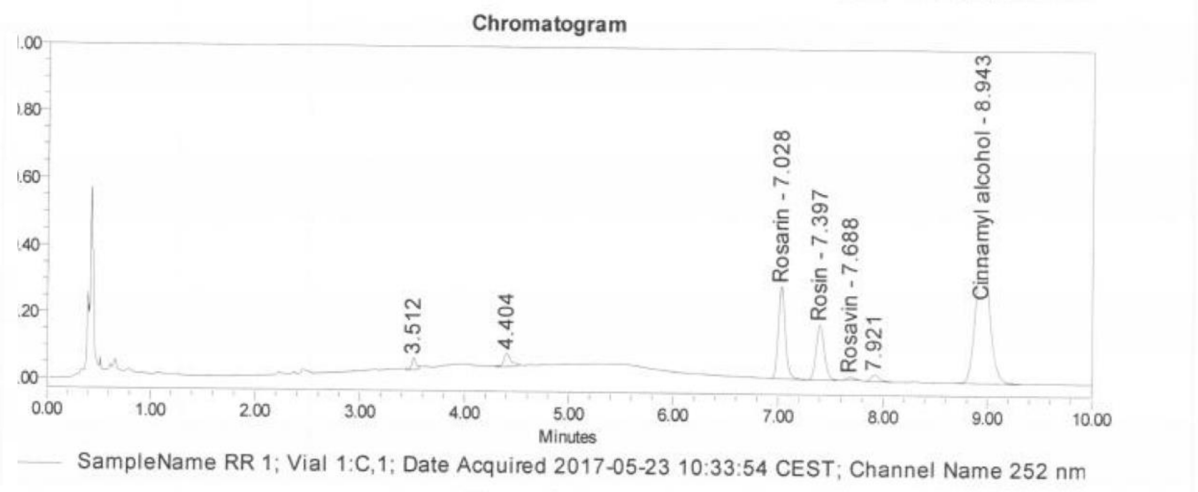

Chemical composition and active principles of SHR-5

In total approximately 140 compounds were isolated from roots and rhizome:

- phenylethanoids, phenylpropanoids and their glycosides,

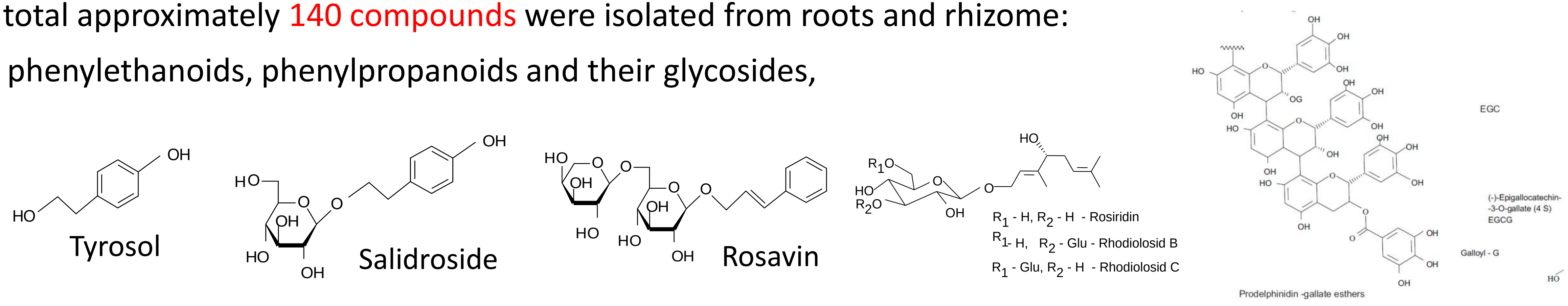

- monoterpene alcohols and their glycosides,
- flavonoids, flavonlignans,
- aryl glycosides.
- cyanogenic glycosides,

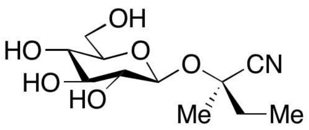

- proanthocyanidins and gallic acid derivatives

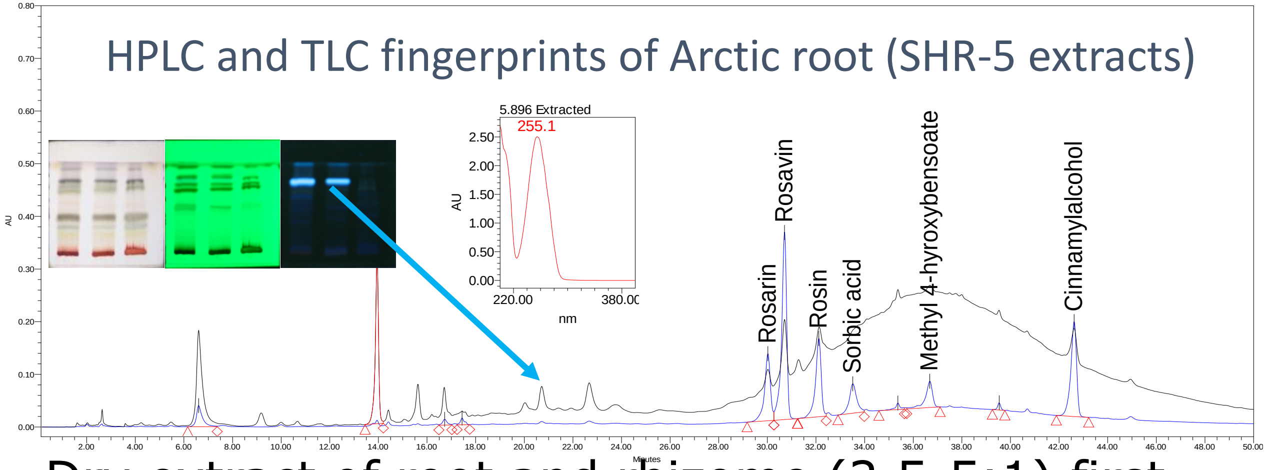

Dry extract of root and rhizome (2.5-5:1) first extraction solvent ethanol 70%, second extraction solvent water;

Factors affecting the chemical composition of Rhodiola rosea preparations

- Genetic factors - chemical races variability

- Environmental factors
    - Climate (temperature, light, rain),
    - soil (pH, fertilization, heavy metals),
    - insects, pest, microbiological infection

- Processing
    - Pulverisation (fine coarse cut, grinding temperature)
    - Extraction solvent (solvent polarity, temperature, duration)
    - Distillation (temperature)
    - Expression (temperature)
    - Fermentation (temperature duration)
    - Purification (removal of undesired components like chlorophylls, etc.

- Storage
    - Light, oxygen (radical building, self-oxidation),
    - Humidity (hydrolysis, enzymatic transformations, microbiological infection)
    - Temperature (polymerization, decomposition, microbiological transformation

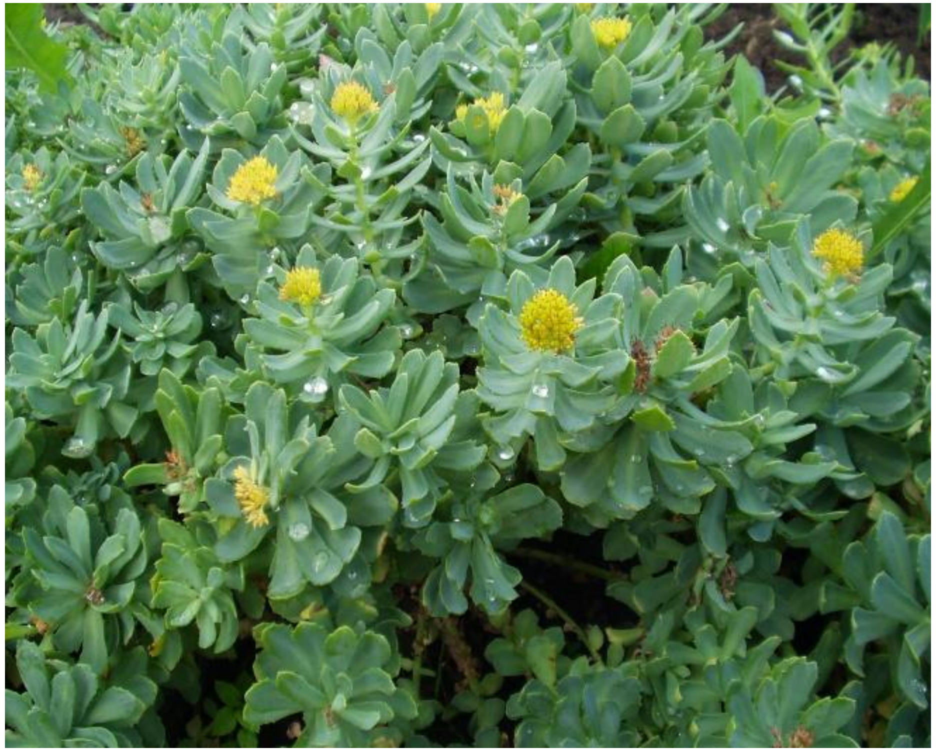

Content of Active Ingredients in Selected Rhodiola Commercial Products*

Fill Weight mg/cap		Salidroside mg/mg fill	Rosavin mg/mg fill	"Rosavins" mg/mg fill	Manufacturer's Claim
305.6	Found<br>Claim	6.3 mg/cap<br>3.0 mg/cap	4.6 mg/cap<br>12.0 mg	16.3 mg/cap<br>24 mg/cap	300 mg 5.4-6.6% total rosavins,<br>3.6-4.4% rosavin, 0.9-1.1% salidroside
333.3	Found<br>Claim	2.0 mg/cap<br>1.0 mg/cap	0.3 mg/cap<br>1.0 mg/cap	0.8 mg/cap	100 mg extract,<br>1 mg (1%) rosavins, 1mg (1%) salidroside
553.0	Found<br>Claim	4.1 mg/cap<br>5.0 mg/cap		10.7 mg/cap<br>15.0 mg/cap	500 mg extract, 3% rosavins, 1% salidroside
158.9	Found<br>Claim	3.7 mg/cap<br>1.5 mg/cap	1.9 mg/cap<br>3.0 mg/cap	7.0 mg/cap	150 mg rhodiola,<br>3% rosavins, 1% salidroside, 0.1% tyrosol
277.3	Found<br>Claim	2.1 mg/cap<br>2.0 mg/cap	2.1 mg/cap	6.2 mg/cap<br>6.1 mg/cap	205 mg extract, 3% rosavins, 1% salidroside
898.5<br>mg/tab	Found<br>Claim	1.1 mg/cap<br>1.0 mg/cap	0.7 mg/cap	2.0 mg/cap<br>2.5 mg/cap	50 mg extract, 5% rosavins, 2% salidroside

* Adapted from: Semple HA, NHP Research Targeted Toward Commercialization: Application of the Field to Medicine Cabinet Concept. 7th NHPRS Research Conference, Halifax, Canada, May 23-26, 2010

…

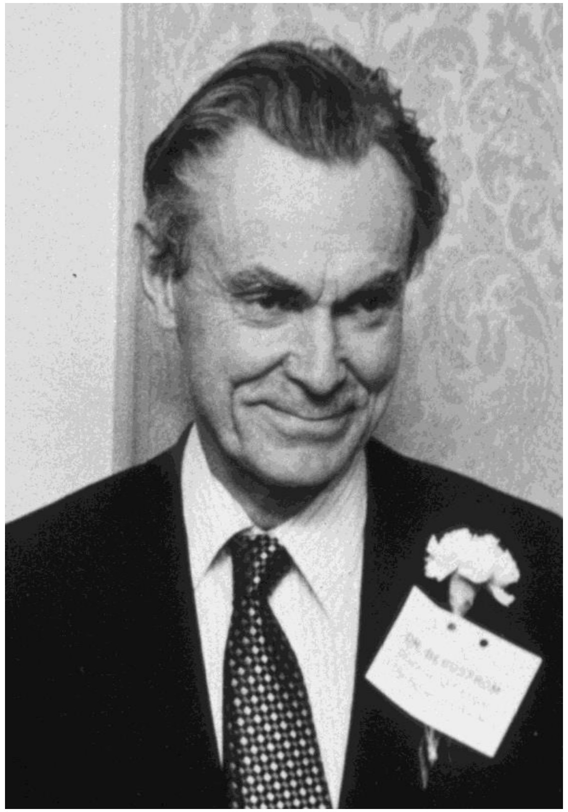

"...the traditional boundaries between various fields of science are rapidly disappearing and what is more important **science does not know any national borders.**
The scientists of the world are forming an invisible network with a very free flow of scientific information – **a freedom accepted by the countries of the world irrespective of political systems or religions.**...“ , (S.K.B.)
...and corporative interests and regulatory bodies (A.P.).

- Sune K. Bergström's speech at the Nobel Banquet, December 10, 1982
- https://www.nobelprize.org/prizes/medicine/1982/bergstrom/speech/
