# Supplementary material for: Two Sides of the Same Coin for Health: Adaptogenic Botanicals as Nutraceuticals for Nutrition and Pharmaceuticals in Medicine
Source: Pharmaceuticals (Basel). 2025 Sep 8;18(9):1346. doi: 10.3390/ph18091346 (PMC12472958; doi:10.3390/ph18091346)
Supplement: Supplementary file 1 [file pharmaceuticals-18-01346-s001.zip › Supplement S4_Herbal medicines vs Dietary supplements Quality and efficacy.pdf]

Herbal medicine vs Dietary supplement

- The efficacy and safety of Herbal medicines containing Rhodiola SHR-5 were demonstrated in clinical studies on humans and preclinical studies on animals.
- The efficacy of these products is evidence-based.
- The results obtained on SHR-5 can not be extrapolated to Rhodiola dietary supplements and similar products produced by other manufacturers.

Why ?

Chemical composition of herbal preparation depends on numerous factors:

- Genetic factors - chemical races variability,
- Environmental factors
    - Climate (temperature, light, rain),
    - soil (pH, fertilization, heavy metals),
    - insects, pest, microbiological infection
- Part of plant (root, bark, leaf, fruit, etc)
- Treatment of raw material to produce drug substances
    - Pulverisation (fine coarse cut, grinding temperature)
    - Extraction (solvent polarity, temperature, duration)
    - Distillation (temperature)
    - Expression (temperature)
    - Fermentation (temperature duration)
    - Purification (removal of undesired components like sugars, resins, chlorophylls, etc.
- Storage
    - Light, oxygen (radical building, self-oxidation),
    - Humidity (hydrolysis, enzymatic transformations, microbiological infection)
    - Temperature (polymerization, decomposition, microbiological transformation

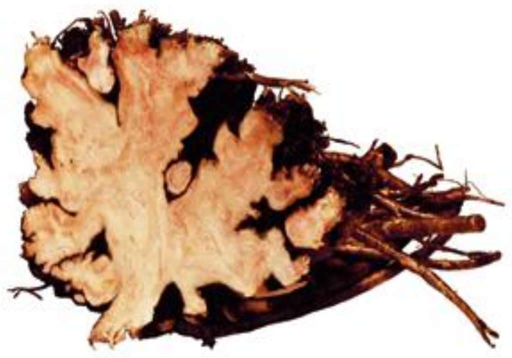

HPTLC comparison of Rhodiola products suspected of adulteration

SHR-5

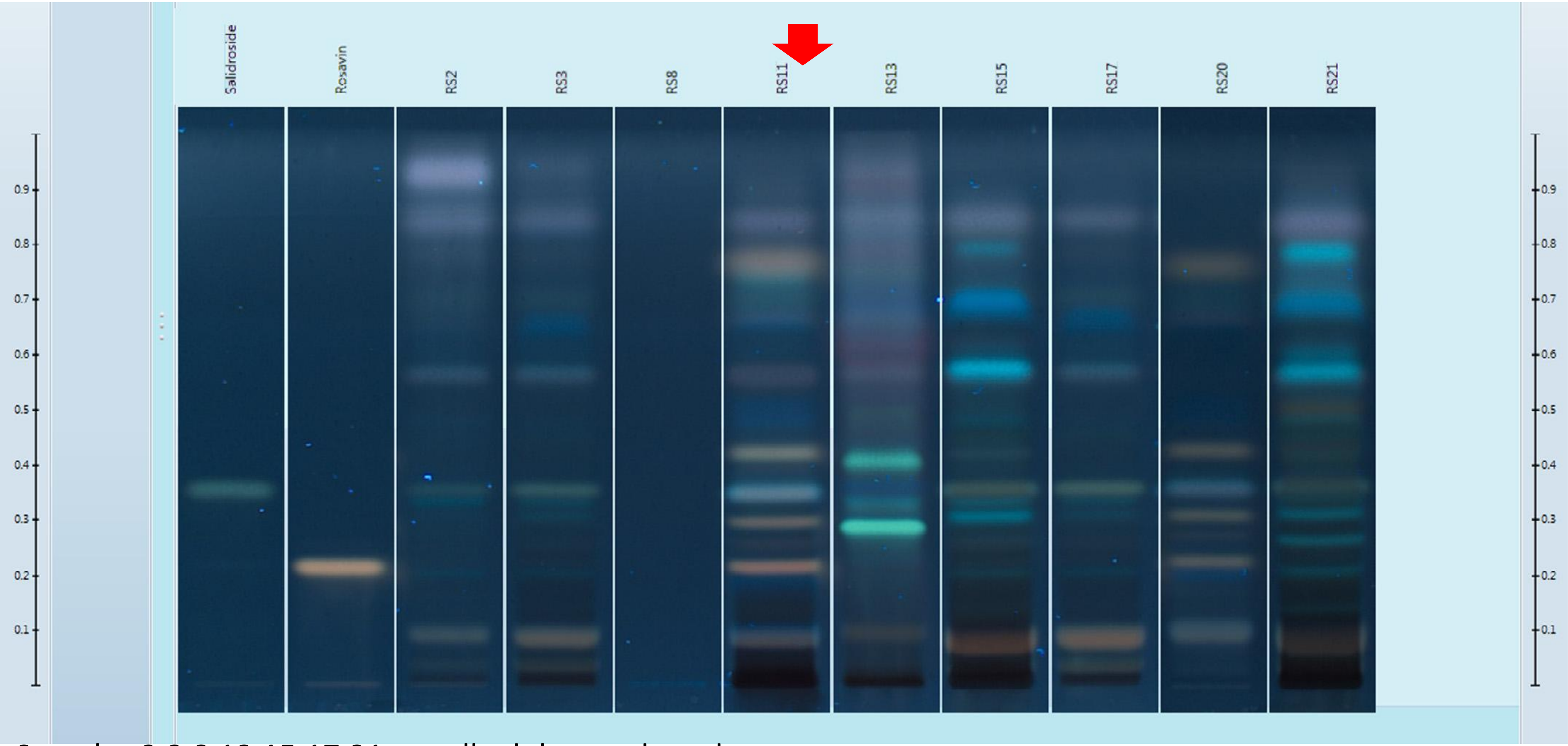

Samples 2,3,8,13,15,17,21 are all adulterated products,
Sample 11 is a THR product –SHR-5,
Sample 20 is an expired product.

Booker et al. 2016. The authenticity and quality of Rhodiola rosea products. Phytomedicine. 23, 754-762 .

Variation of Rhodiola products – HPTLC fingerprints

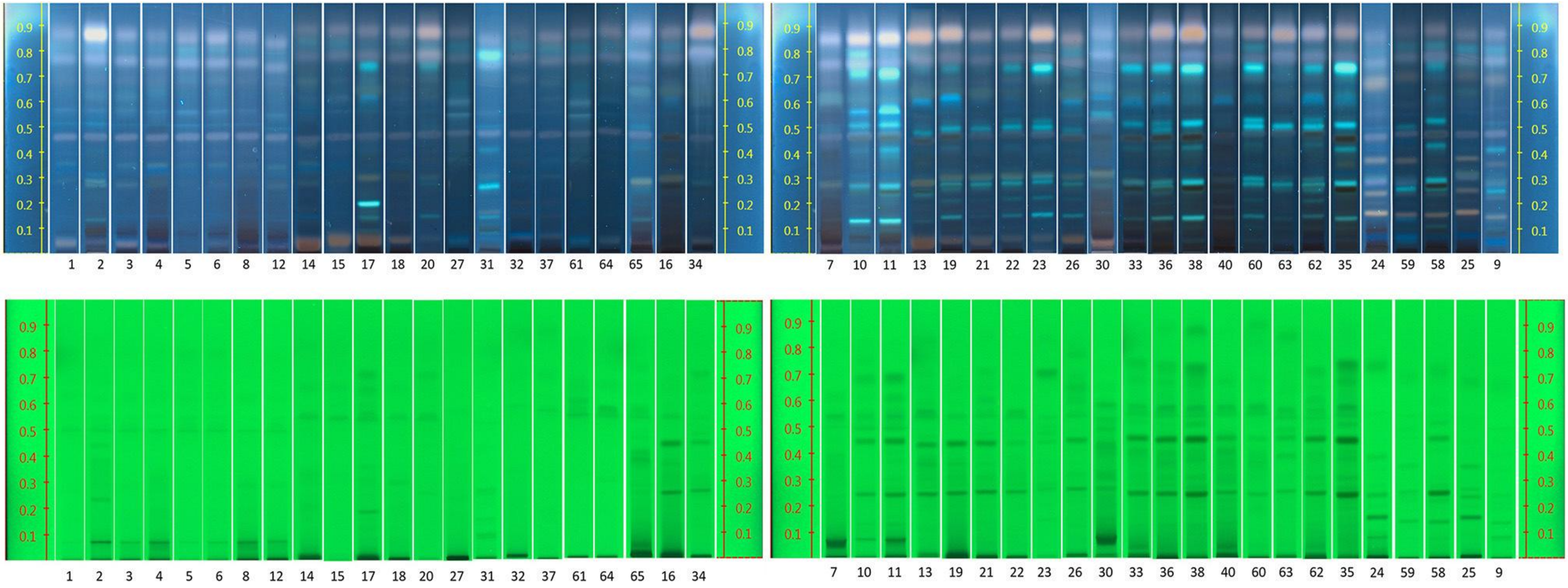

HPTLC results for all *Rhodiola* market samples, mobilephase[Ethylacetate,methanol,water,formicacid(77:13:10:2)].

Booker et al. ComparativeQualityStudyof *Rhodiola* Species

Content of Active Ingredients in Selected Rhodiola Commercial Products*

Fill Weight mg/cap		Salidroside µg/mg fill	Rosavin µg/mg fill	Total Rosavins µg/mg fill	Rosiridin SIM counts/20 µg product	Manufacturer's Claim
305.6	Found<br>**Found**<br>Claim	20.5<br>6.3 mg/cap<br>3.0 mg/cap	15.1<br>4.6 mg/cap<br>12.0 mg	53.4<br>16.3 mg/cap<br>24 mg/cap	3.24E+7	300 mg 5.4-6.6% total rosavins, 3.6-4.4% rosavin, 0.9-1.1% salidroside
333.3	Found<br>**Found**<br>Claim	5.9<br>2.0 mg/cap<br>1.0 mg/cap	1.0<br>0.3 mg/cap<br>1.0 mg/cap	2.7<br>0.8 mg/cap	6.43E+5	100 mg extract,<br>1 mg (1%) rosavins,<br>1mg (1%) salidroside
313.0	Found<br>**Found**<br>Claim	8.9	4.5	15.6<br>4.9 mg/cap<br>9.75 mg/cap	8.81E+6	150 mg rosavins =<br>3.5% total rosavins
553.0	Found<br>**Found**<br>Claim	7.4<br>4.1 mg/cap<br>5.0 mg/cap	7.7	19.3<br>10.7 mg/cap<br>15.0 mg/cap	7.27E+6	500 mg extract, 3% rosavins, 1% salidroside
158.9	Found<br>**Found**<br>Claim	23.3<br>3.7 mg/cap<br>1.5 mg/cap	11.7<br>1.9 mg/cap<br>3.0 mg/cap	44.2<br>7.0 mg/cap	2.56E+7	150 mg rhodiola,<br>3% rosavins, 1% salidroside, 0.1% tyrosol
385.3	Found<br>**Found**<br>Claim	5.1<br>2.0 mg/cap	0.03<br>0.01 mg/cap	0.5<br>0.2 mg/cap	Not Detected	250 mg, 1% rhodiola
277.3	Found<br>**Found**<br>Claim	7.7<br>2.1 mg/cap<br>2.0 mg/cap	7.7<br>2.1 mg/cap	22.3<br>6.2 mg/cap<br>6.1 mg/cap	5.46E+6	205 mg extract, 3% rosavins, 1% salidroside
898.5<br>mg/tab	Found<br>**Found**<br>Claim	1.2<br>1.1 mg/cap<br>1.0 mg/cap	0.8<br>0.7 mg/cap	2.2<br>2.0 mg/cap<br>2.5 mg/cap	4.15E+5	50 mg extract, 5% rosavins, 2% salidroside

* Adapted from: Semple HA, NHP Research Targeted Toward Commercialization: Application of the Field to Medicine Cabinet Concept. 7th NHPRS Research Conference, Halifax, Canada, May 23-26, 2010

Herbal medicine Arctic root SHR-5 *vs* Dietary supplement Ferox

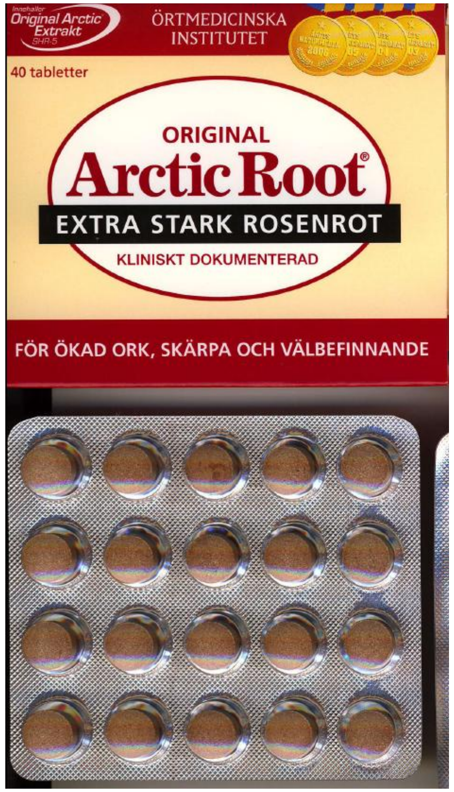

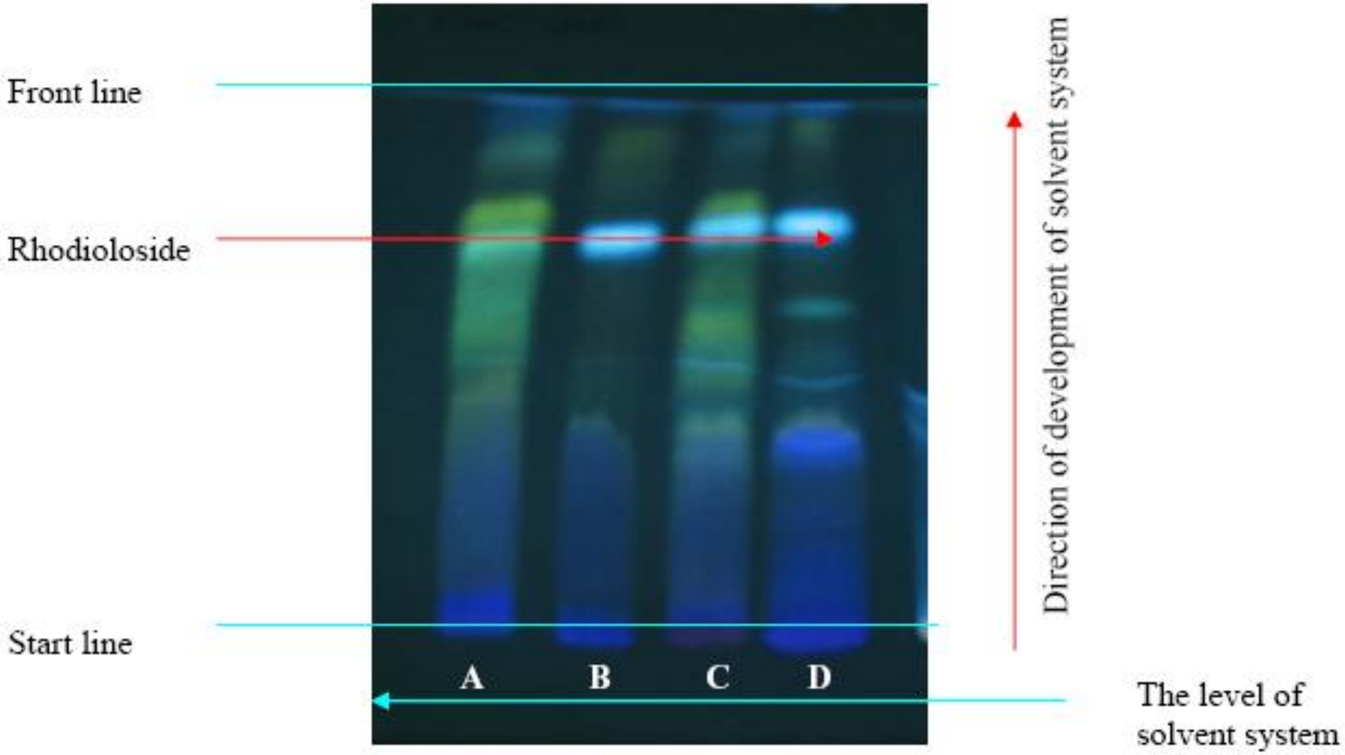

Figure 3. Thin Layer chromatography of Ferox and Arctic root tablets:

- Line A – Ferox tablets - ehylacetate extract
- Line B – Arctic root tablets – ethylacetate extract
- Line C - Ferox tablets – methanolic extract
- Line D - Arctic root tablets – methanolic extract

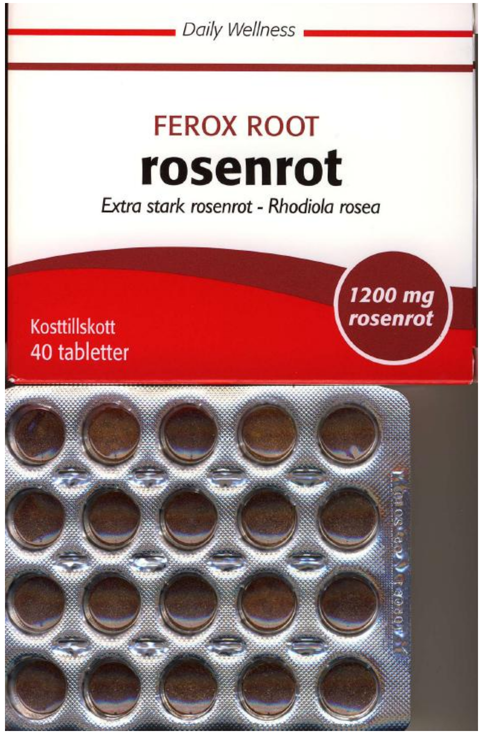

[Buy Bioroot Rhodiola Rosea Forte 60 Tablets | Apohem]

Comparison: Arctic root - Ferox

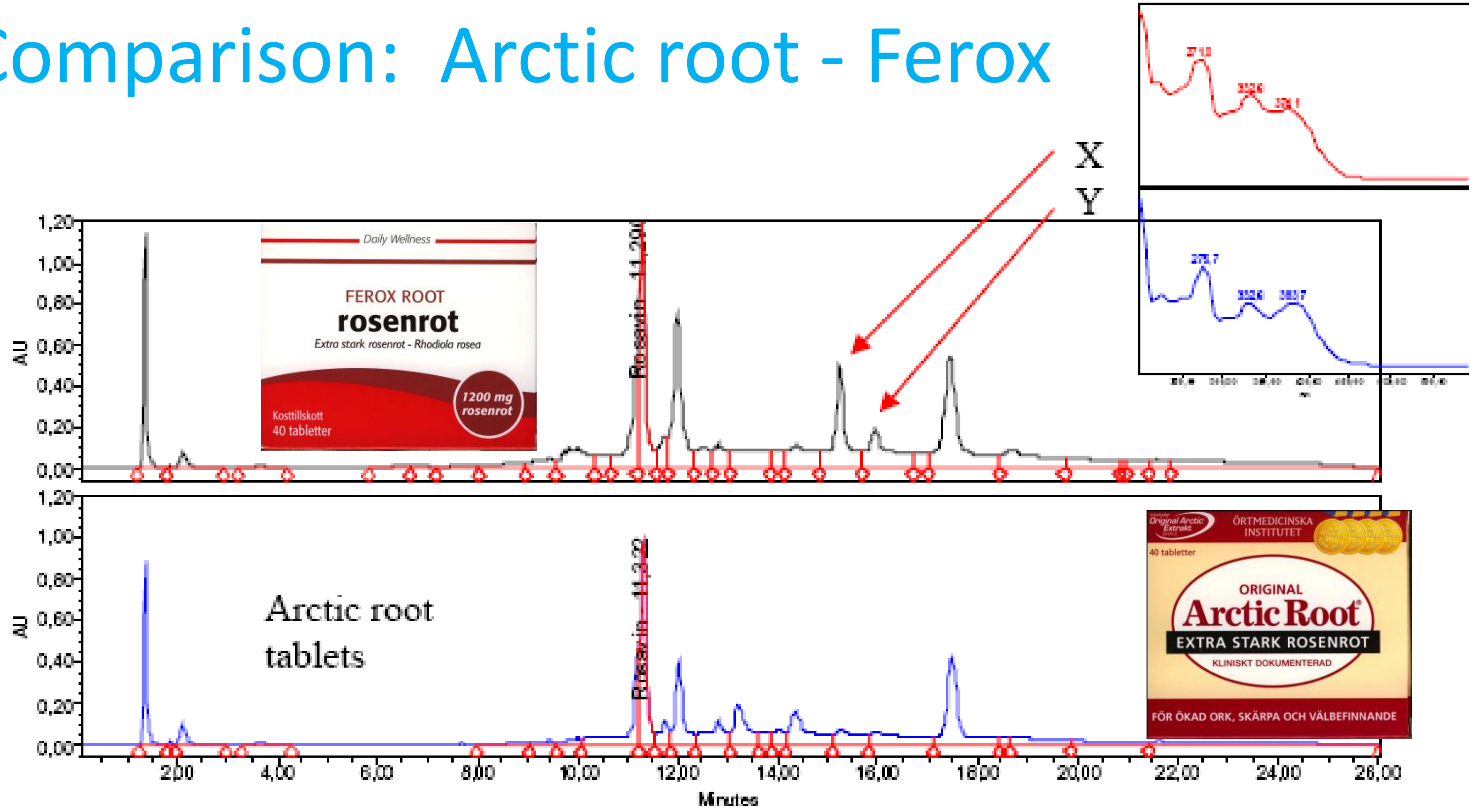

Figure 3. HPLC fingerprints of Ferox and Arctic root extracts detected at 252 nm. Unidentified impurities (X and Y) in Ferox Rhodola native extracts. In upper window UV spectra of compounds X and Y are shown.

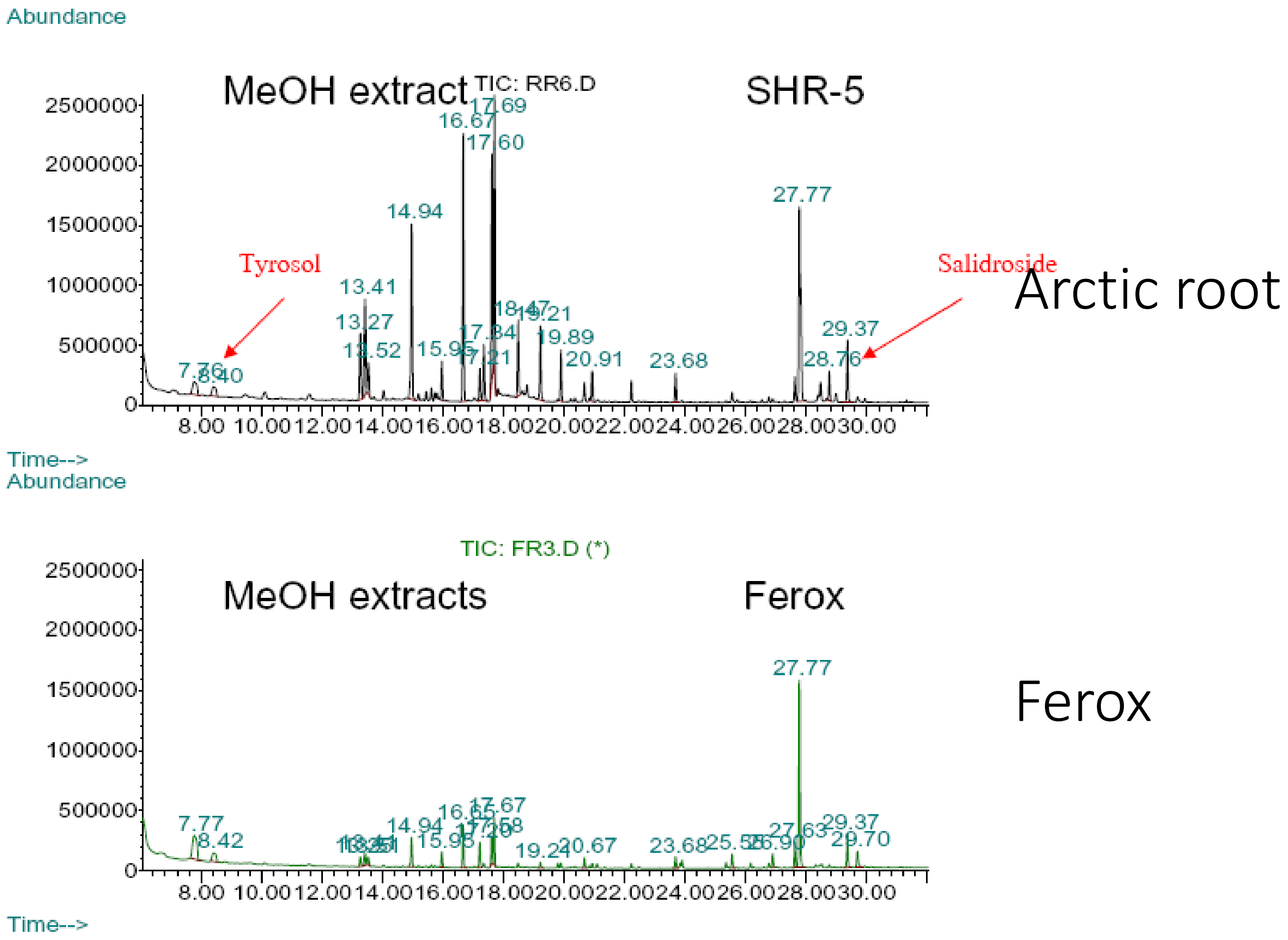

Figure 6. GC-MS fingerprints of Ferox and Arctic root MeOH extracts, TMS- derivatives.

Conclusions

- It is a challenge to produce standardized extracts with reproducible chemical composition, and consequently with reproducible pharmacological activity, particularly when extracts are produced by different manufacturers.
- Multicomponent herbal medicinal products manufactured from the same plant can not be absolutely identical as it is in case of active pharmaceutical ingredients with ONE single compound.
- The results therapeutic efficacy obtained for one herbal preparation can not be extrapolated on other preparations or similar products unless they are specified/standardized and pharmacologically investigated analogues to reference product.
- Otherwise, the evidence of therapeutic efficacy comparable to therapeutic outcomes of positive control is required.

Bioequivalence

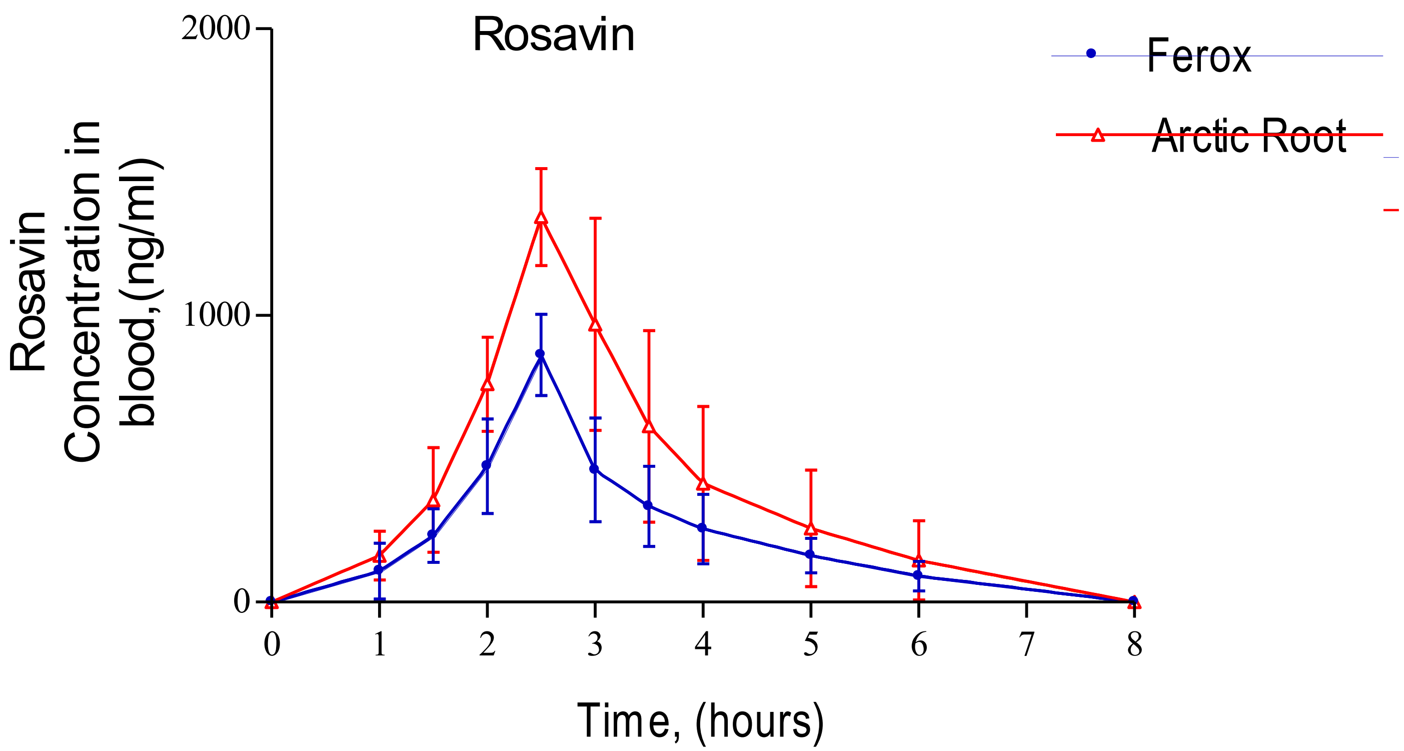

"Rosenrot uncoated tablets 720 mg" (Naturapoteket) are not bioequivalent to the reference "Rosenrot" (Swedish Herbal Institute), film-coated tablets 500 mg, when orally administrated in equivalent doses.

Why sometimes we have contradictory results from different studies where “the same” preparations from different manufacturers were used?

- Because the composition and the content of active constituents is quit different in various medications used in the clinical studies,
- Because the dose-effect dependence of active ingredients (like in Rhodiola extract) is not linear and has a bell shape.
- Consequently, the dose which is efficient in one study with one preparation could be not efficient in another one with another extract.
- Therefore the results obtained on SHR-5 can not be extrapolated on any Rhodiola preparation and similar products produced by other manufacturers
