# Supplementary material for: Two Sides of the Same Coin for Health: Adaptogenic Botanicals as Nutraceuticals for Nutrition and Pharmaceuticals in Medicine
Source: Pharmaceuticals (Basel). 2025 Sep 8;18(9):1346. doi: 10.3390/ph18091346 (PMC12472958; doi:10.3390/ph18091346)
Supplement: Supplementary file 1 [file pharmaceuticals-18-01346-s001.zip › Supplement S5_AESGP 2010 Legal and Regulatory framework for Herbal Medicine.pdf]

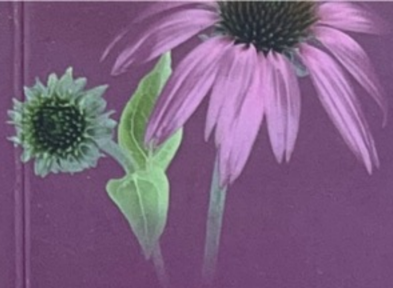

LEGAL AND REGULATORY FRAMEWORK FOR HERBAL MEDICINES

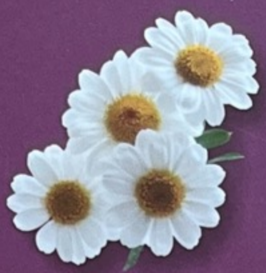

APRIL 2010

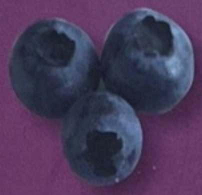

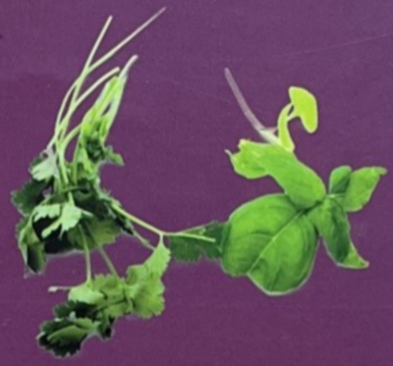

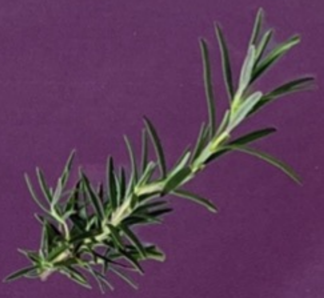

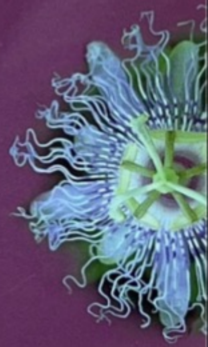

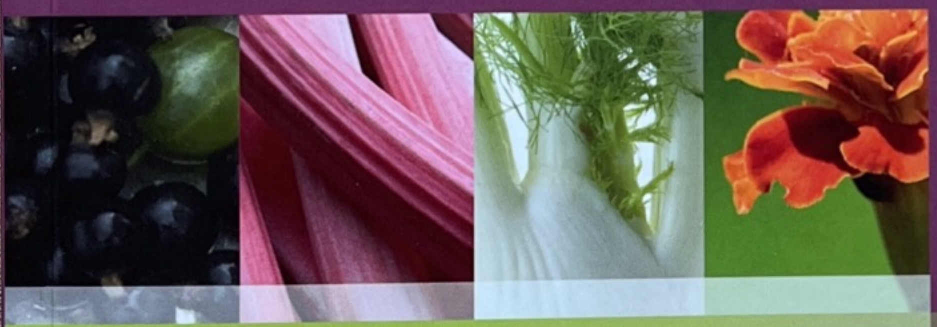

AESGP

LEGAL AND REGULATORY FRAMEWORK FOR HERBAL MEDICINES

AESGP

April 2010

Association Européenne des Spécialités Pharmaceutiques Grand Public

Association of the European Self-Medication Industry

Europäischer Verband der Arzneimittel-Hersteller

7, avenue de Tervuren
B-1040 Brussels
Tel: +32 2 735 51 30
Fax: +32 2 735 52 22
Email: info@aesgp.be
Website: www.aesgp.be

Legal framework for herbal medicines in the European Union

The main principles of the legislation on human medicines in the European Union are laid out in *Directive 2001/83/EC of the European Parliament and of the Council of 6 November 2001 on the Community code relating to medicinal products for human use*[1], as amended (the so-called "Community Code").

As any medicine, herbal medicinal products need a marketing authorisation or registration from a Member States' Competent Authority in order to be marketed in the European Union.

As defined in the Community Code, herbal medicines can be authorised on the basis of a "full marketing authorisation" which is however quite rarely the case. The only alternative legal basis until 2005 was the bibliographic application in which pre-clinical tests and clinical trials are replaced by bibliographic references provided that the safety and efficacy of the medicine are adequately proven. The other pre-condition is that the medicine has been on the market for at least 10 years.

In 1998, AESGP carried out a study[2] on behalf of the European Commission, which looked at the regulatory situation in EU Member States and analysed the difference in legislation and assessment criteria. It was found that some countries had national legislations around traditional herbal medicines or remedies. The study concluded in favour of a harmonised European legal framework for traditional herbal medicines. The legislator too felt that *"the differences that currently exist between the provisions laid down in the Member States may hinder trade in traditional herbal medicinal products within the Community and lead to discrimination and distortion of competition between manufacturers of these products. They may also have an impact on the protection of public health since the necessary guarantees of quality, safety and efficacy are not always provided at present."*

A regulatory framework for traditional herbal medicines was hence created with *Directive 2004/24/EC amending, as regards traditional herbal medicinal products, Directive 2001/83/EC* which came into force on 30 April 2004 and had to be transposed by EU Member States into their national legislation and implemented by 30 October 2005. It *de facto* introduces a third option to market herbal medicines provided they meet the criteria as set out in the Directive.

Definitions

The Community Code defines a **herbal medicinal product** as *"Any medicinal product, exclusively containing as active ingredients one or more herbal substances or one or more herbal preparations, or one or more such herbal substances in combination with one or more such herbal preparations"* and;

Herbal substances: *"All mainly whole, fragmented or cut plants, plant parts, algae, fungi, lichen in an unprocessed, usually dried, form, but sometimes fresh. Certain exudates that have not been subjected to a specific treatment are also considered to be herbal substances. Herbal substances are precisely defined by the plant part used and the botanical name according to the binomial system (genus, species, variety and author)."*

Herbal preparations: *"Preparations obtained by subjecting herbal substances to treatments such as extraction, distillation, expression, fractionation, purification, concentration or fermentation. These include comminuted or powdered herbal substances, tinctures, extracts, essential oils, expressed juices and processed exudates."*

A **traditional herbal medicinal product** is *"A herbal medicinal product that fulfils the conditions laid down in Article 16a(1)."*

The conditions or criteria are as follows:

- their indication makes them appropriate for a use not requiring the intervention of a medical doctor i.e. they are *non-prescription medicines*

- their *strength and posology* is *specified* and derived from tradition
- their route of administration is either *oral, external* and/ or *inhalatory*
- they are not harmful in the specified conditions of use and their *efficacy is plausible* based on a long-standing tradition i.e. *30 years of medical use of which at least 15 years in Europe.*

Traditional herbal medicines can contain vitamins and minerals provided that the action of these minerals and vitamins is ancillary to that of the herbal ingredients linked to the indication.

COMMITTEE ON HERBAL MEDICINAL PRODUCTS (HMPC)[3]

Besides the introduction of the concept of traditional herbal medicines benefiting from a simplified registration process, the Directive also established a full-fledged committee within the European Medicines Agency (EMA): the Committee on Herbal Medicinal Products (HMPC).

The Committee is composed of representatives of the EU Member States (one member and one alternate per country) having expertise and experience in the evaluation of herbal medicines. Members of the HMPC can be accompanied by experts in specific scientific or technical fields.
The legislation makes it possible for the Committee to complement its overall expertise by co-opting up to five members and five experts in respectively clinical pharmacology, paediatric medicine, experimental/non-clinical pharmacology, toxicology, and general and family medicine have been appointed on this basis. In addition, observers from the European pharmacopoeia, candidate and potential candidate countries to the EU sit on the Committee. Co-opted members are chosen among experts nominated by Member States or the European Medicines Agency.

The chair and vice-chair are elected among and by the committee members for 3 years, renewable once. Members, alternates and co-opted members serve a three-year term as well.
The Committee had its inaugural meeting in September 2004. It meets during 2 consecutive days, 6 times a year, at the EMA premises in London.

Its tasks are defined in Directive 2004/24/EC and encompass:

- developing *Community list entries* and *Community monographs* on plants
- developing *guidance* on the content of a simplified application
- issuing an *opinion in case the 15 year-criteria* of standing medical use in Europe is *not met*
- issuing a decision in case of *referral* on a traditional herbal medicine and an opinion in case of a referral on a herbal medicine

A Working Party on Community Monographs and Community List (MLWP) and two drafting groups, one on organisational matters (ORGAM DG) and one on quality (Q DG), assist the HMPC in its tasks.

COMMUNITY HERBAL MONOGRAPHS & LIST ENTRIES

The development of Community Monographs and List entries is one of the main tasks of the EMA Committee on Herbal Medicinal Products. Both Community Monographs and Community List, by harmonising indications and instruction for use, will contribute to a greater coherence and homogeneity of the EU herbal market and increase ease of circulation and marketing of these medicines.

Community monograph

A Community herbal monograph is the result of the Committee's evaluation of available scientific data (well-established use) or of the historic use of the herbal substance, preparations or combinations thereof in the European Community (traditional use). For some plants, the Community monograph covers both well-established use and traditional use. The *Guideline on the assessment of clinical safety and efficacy in the preparation of Community herbal monographs for well-established and of Community herbal monographs / Entries to the Community list for traditional herbal medicinal products / substances / preparations*[4] provides important clarification on the data needed to support a well-established use indication or a traditionally used one.

The structure of Community monographs has been designed following the Summary of Product Characteristics (SPC) structure, as established by Article 8(3)j of Directive 2001/83/EC.

Community herbal monographs provide a harmonised approach to the scientific assessment of herbal medicinal products in the EU, and, according to Directive 2004/24/EC, the Member States shall take them into account when they examine an application relating to a product for which a Community monograph has been established. Hence, applications based on such monographs should be granted without the need for re-evaluation of data encompassed in the monographs. This is unfortunately not always the case to date as some Member States add further restrictions (e.g. in age limits or indications) than those laid down in the monograph. As such, these actions contravene the intent of the legislation which was to harmonise the requirements so that the free circulation of traditional herbal medicines could be ensured in the European Union. This echoes the Commission's opinion, which is that *"even though the Member States are not obliged to follow the monograph, any decision not to accept the content of the monograph as it is adopted by the HMPC should be duly justified taking into account their important role to bring harmonisation to this field, and to facilitate the use of the simplified registration procedure."*

Recital 5 of the Directive does not exclude that even in case of long tradition, additional safety data may be needed by Competent Authorities and they are entitled to ask for such data. However, given the wide field of literature references reviewed and the amount of expertise and knowledge going into the monograph, this should remain an exception.

Community list

The 'Community list of herbal substances, preparations and combinations thereof for use in traditional herbal medicinal products' is being gradually developed through entries of structured information relating to individual herbal substances or preparations.

Following the provisions of the Directive, the list shall contain, for each herbal substance or preparation, the indication, the specified strength and the posology, the route of administration and any other information necessary for the safe use of the herbal substance or preparation contained in a traditional herbal medicinal product.

In contrast to the Community herbal monographs, the Community list is legally binding to applicants and competent authorities in the Member States insofar as:

- If an application for traditional use registration relates to a herbal substance, the preparation or combination thereof contained in the list, the applicant does not have to provide the following:
  - information on previous authorisations or registration;
  - bibliographic or expert evidence of proof of traditional use; and
  - bibliographic review of safety data.

- Competent authorities will not have the opportunity to require additional data to assess the safety and the traditional use of the product.

According to the legislation and to the fact that they are endorsed by the Commission, list entries have a specific status. The existence of a list entry makes it clear that the product cannot be harmful under normal conditions of use and that the data on traditional use are sufficient. *De facto*, the exhaustiveness of list entries is a pre-condition to their scientific finalisation. For this reason, list entries of *Valeriana officinalis* L., radix and *Linum usitatissimum* L., semen are still pending as the Committee could not finalise them on the basis that genotoxicity data were incomplete.

If a herbal substance, preparation or a combination thereof ceases to be included in the Community list, registration based on the plant on the list will have to provide the relevant documentation (referred to in Article 16c(1)) within 3 months. If no documentation is submitted, the registration will be repealed.

Development of Monographs and List Entries

The HMPC has established a priority list of plants for which monographs should be developed, based on the AESGP priority list and the lists of other interested parties. The AESGP list ranked plants (as high, medium and low priority) based on their commercial importance.

The development of a Community monograph or list entry is carried out by a **rapporteur,** appointed among HMPC members. When nominating rapporteurs, the Committee takes into consideration the expression of interest from its members whilst striving to reach a balance between countries. However, to date, Germany has been the rapporteur of a great number of monographs.

The next step is the gathering of the bibliographical references and scientific data on the plant which will be subject to the monograph. To help the rapporteur in this task, a public call for scientific data[5] can be issued. It lasts two months.

When the proposal for a monograph or list entry is ready, it is discussed first within the MLWP and then by the full HMPC. When consensus is reached, the draft is released for a three-month public consultation. The comments received are subsequently reviewed, the draft monograph/list entry revised and then discussed within the MLWP and then by the HMPC.

The HMPC vote and the *approved monograph* is published as final on the EMEA website along with its assessment report, the Committee's opinion, the literature reference, and an overview of the comments received.

However, in case of a draft *list entry*, its approval by the Committee does not constitute the final step of the procedure. The draft list entry (which is final from a scientific point of view) proceeds to the European Commission for endorsement. Following this approval, the final version of the Community list entry is published in the Official Journal.

From the call for data to its publication as final on the EMEA website, the development of a Community monograph takes on average about 2 years. However, it should be noted that sometimes delays occur between endorsement by the Committee and time of publication.

53 monographs have been issued as final so far, 18 are ongoing and 4 are suspended (cf. Annex I).
In the case of the following plants, the assessment work had indeed to be interrupted due to missing data or issues encountered and the HMPC issued public statements to explain the situation:

- *Centella Asiatica* (L.) Urban, herba – major issue related to the level of purification of extracts which are highly refined.
- *Euphrasia officinalis* L. and *Euphrasia rostkoviana* Hayne, herba – no adequate data could be found concerning the Euphrasia-containing preparations to confirm the safe use.
- *Salvia officinalis* L., aetheroleum – quality data have been insufficient to confirm the positive benefit-risk assessment.
- *Urtica dioica* L., Urtica urens L., their hybrids or mixtures, radix – the indication (symptomatic treatment of benign prostatic hyperplasia) was not considered acceptable for self-care and hence was not meeting the criteria laid down in Article 16a(1)(a). On the other hand, the quality of clinical trials conducted with this plant was not considered adequate to develop a positive Community herbal monograph for well-established use.

Seven list entries figure on the Community List, 5 are pending (cf. Annex II).

An interested party can submit a *proposal for the development of a monograph or list entry* on a plant which is not on the HMPC priority list. As detailed in the procedural document[6], the interested party needs to send a justification as to why the plant should be subject to a monograph or list entry, the supporting documentation and a completed draft monograph or list entry.

Reasons for which a Community monograph can be revised and timelines for doing so have been clarified by the HMPC in a reflection document[7].

All timelines, procedures and templates are published on the EMA website[8].

OTHER TASKS OF THE HMPC

Referrals

The Committee plays an active role in the case where a product does not fulfil the 15 years criteria of use in the Community. If the product is otherwise eligible for the simplified registration, the Member State will refer the product to the Committee when the application for traditional-use registration has been submitted. The HMPC will review the literature provided and if possible will develop a Community monograph on the given plant. The Committee's opinion (or the monograph if established) will have to be taken into account by the Member States when taking their final decision.

Guidance has been developed by the HMPC to provide details around the documentation that needs to be provided by Member States and Applicants/Marketing Authorisation Holders in support of a simplified registration referral under Articles 16c(1)c and 16c(4)[9].

So far the Committee has handled one referral procedure. This referral procedure started in January 2008 at the request of a Member State (Slovak Republic) regarding a traditional use registration application for a fixed combination herbal medicinal product (Doppelherz Energovital Tonik K) for which the number of ingredients had been reduced during the 30 years of medicinal use. The HMPC concluded in March 2008 that the evidence of the long-standing use of the herbal medicinal product was adequate. The referring Member State followed the conclusions of the HMPC and Doppelherz Energovital Tonik

K was registered as a traditional herbal medicinal product by the Slovak Republic on 24 September 2008.

The Committee also has a role to play in case of referral of herbal medicines arising from a mutual recognition procedure or decentralised procedure. It is responsible for providing its scientific conclusions in case of a referral involving a traditional herbal medicine and will advise the Committee for Medicinal Products for Human Use (CHMP) in the case of all other herbal medicines. The Committee has not yet had the occasion to exert its competence in this field.

Advisory role

The Committee can provide its expert opinion on a herbal medicinal product upon request. A specific form has to be completed[10] for this purpose.

Dossier requirements

Herbal medicines are subject to the same general requirements as conventional medicines. The applicants need to submit an application containing all the necessary information on the product itself as well as the data on quality, safety and efficacy. However, the type and necessary information differs depending on the legal basis.

LEGAL BASIS

Full application

The particulars and documents that need to be contained in the application are defined in Article 8(3) of Directive 2001/83/EC – also called Community Code. Results of pharmaceutical tests, pre-clinical (toxicological and pharmacological) tests and of clinical trials have to be provided.
It is rather rare nowadays that a herbal medicine is authorised on the basis of a full application, given the level of requirements and the cost associated with the performance of clinical trials. In addition, most of the requirements were developed for purely chemical entities and do not always apply as such to the specificities of herbal ingredients.

Bibliographic application

Article 10a of Directive 2001/83/EC as amended offers the possibility of replacing the results of pre-clinical tests and clinical trials by scientific literature provided that the active substance of the medicinal product has been in *well-established* medicinal use within the Community for at least ten years.

Simplified procedure on the basis of traditional use

Traditional herbal medicines that meet the criteria laid down in Article 16a of the Directive can then be subject to a simplified authorisation, the so-called 'registration' procedure.
The pharmaceutical part of the dossier should be complete, there is no difference compared to full or bibliographic applications. The application dossier is simplified as far as modules IV and V are concerned.

According to Article 16c, an application for registration shall be accompanied by, among other items, a bibliographical review of safety data together with an expert report. All aspects that are relevant for the safety of the patient or consumer must be covered by appropriate literature or appropriate reference to a review of literature and must be addressed in the expert report in a registration procedure. Justification for the lack of data should be submitted. Further detailed guidance is provided in the *Guideline on non-clinical documentation for herbal medicinal products*[11].

A well-documented, consistent and long-standing use over at least 30/15 years will, in most cases, provide the basis for acceptance of an indication. It is made clear that the requirements to demonstrate medicinal use throughout the period of 30 years are satisfied even when the marketing of the product has not been based on a specific authorisation, if the number or amount of ingredients has been reduced or if reference is made to a "corresponding" product.
Plausibility of a traditional indication may include, but is not limited to, clinical data, pharmacological studies or case reports. Types of bibliographical and/or expert evidence are provided by the HMPC as examples of references that may be used in the *Guideline on the assessment of clinical safety and efficacy in the preparation of Community herbal monographs for well-established and of Community herbal monographs / Entries to the Community list for traditional herbal medicinal products / substances / preparations.*

In the case of combinations, information on the combination is to be provided; if the individual active ingredients are not sufficiently known, the data shall also relate to the active ingredients.

Content of the Dossier

Dossier requirements in terms of quality, safety and efficacy are laid down in *Directive 2001/83/EC as amended* by *inter alia Directive 2004/24/EC* relative to traditional herbal medicines and *Directive 2003/63/EC amending Annex I of*

the Directive on the analytical, pharmaco-toxicological and clinical standards and protocols in respect of the testing of medicinal products[12]. Section 4, Part III of the Annex focuses on herbal medicinal products.

In addition, a number of guidance documents provide additional clarification or scientific and technical details. Guidance documents are not binding; they represent 'soft law'.

The Notice to Applicants[13] constitutes the main general reference. It is prepared by the Commission in conjunction with the EMA and the Member States and provides general clarification on three main areas:

- Procedures for marketing authorisation (Volume 2A)
- Presentation and content of the application dossier (Volume 2B)
- Guidelines (Volume 2C)

Requirements, guidelines and recommendations specific to herbal medicines and traditional herbal medicines are set out below. Most of the guidance documents were elaborated by the HMPC and its sub-groups.

QUALITY / GMP

The provisions of Module 3 on quality, including compliance with monograph(s) of the European Pharmacopoeia, apply to the authorisations and registrations of herbal medicinal products.

The following documents provide guidance in the quality area and on good manufacturing practices:

- Guideline on Good Agricultural and Collection Practice (GACP) for starting materials of herbal origin[14]
- GMP requirements for herbal medicinal products are specified in Annex 7[15]
- Guideline on Quality of Herbal Medicinal Products/ Traditional Herbal Medicinal Products[16]
- Guideline on Quality of Combination Herbal Medicinal Products / Traditional Herbal Medicinal Products[17]
- Guideline on Declaration of Herbal Substances and Herbal Preparations in Herbal Medicinal Products/Traditional Herbal Medicinal Products in the SPC[18]
- Guideline on specifications: Test procedures and Acceptance Criteria for Herbal Substances, Herbal Preparations and Herbal Medicinal Products / Traditional Herbal Medicinal Products[19]
- Reflection paper on Markers used for quantitative and qualitative analysis of Herbal Medicinal Products and Traditional Herbal Medicinal Products[20]
- Reflection Paper on the use of Fumigants[21]
- List of Questions & Answers received during the HMPC assessors training on quality issues emerging for herbal medicinal products[22]

SAFETY

The following HMPC guidance documents should be taken into account for Module 4:

- Guideline on non-clinical documentation for herbal medicinal products in applications for marketing authorisation (bibliographic and mixed applications) and in applications for simplified registration[23]
- Guideline on the assessment of genotoxicity of herbal substances/preparations[24]
- Guideline on selection of test materials for genotoxicity testing for traditional herbal medicinal products/herbal medicinal products[25]
- Reflection paper on the risks associated with furocoumarins contained in preparations of *Angelica archangelica* L.[26]
- Reflection paper on ethanol content in herbal medicinal products and traditional herbal medicinal products used in children[27]
- Public Statement on "CPMP List of Herbal Drugs with serious risks, dated 1992."[28]

EFFICACY

The following two guidelines are relevant for Module 5:

- Guideline on the assessment of clinical safety and efficacy in the preparation of Community herbal monographs for well-established and of Community herbal monographs/ entries to the Community list for traditional herbal medicinal products/substances/preparations
- Guideline on the clinical assessment of fixed combinations of herbal substances/herbal preparations[29]

PHARMACOVIGILANCE

The pharmacovigilance requirements apply by analogy to herbal medicines and traditional herbal medicines. Those are laid down in *Directive 2001/83/EC* as amended and explicated in *Volume 9A*[30].

In addition, the EMEA has issued a number of guidance documents specific to species, plants or chemical compounds:

- EMEA Public Statement on herbal medicinal products containing Cimicifugae racemosae rhizome (Black cohosh, root)[31]
- Public Statement on Chamomilla containing herbal medicinal products[32]
- Public Statement on the allergenic potency of herbal medicinal products containing soya or peanut protein[33]
- Public Statement on the use of herbal medicinal products containing asarone[34]
- Public Statement on the risks associated with the use of herbal medicinal products containing Aristolochia species[35]
- Public Statement on the use of herbal medicinal products containing pulegone and methofuran[36]
- Public Statement on Capsicum/ capsaicin containing herbal medicinal products[37]
- Public Statement on the use of herbal medicinal products containing estragole[38]
- Public Statement on the use of herbal medicinal products containing methyleugenol[39]
- Public Statement on "CPMP list of herbal drugs with serious risks"[40]

FORMAT OF THE DOSSIER

Dossiers should follow the Common Technical Document (CTD) format.

Guidance concerning the use of the CTD format in the preparation of a registration application for traditional herbal medicinal products can be found in a specific HMPC guideline[41].

CLASSIFICATION HERBAL MEDICINES VS. FOOD SUPPLEMENTS

The classification of products as medicinal products or as food supplements is not harmonised at EU level and remains a national competence.

Article 2.2 of Directive 2001/83/EC as amended foresees that "*In cases of doubt, where, taking into account all its characteristics, a product may fall within the definition of a 'medicinal product' and within the definition of a product covered by other Community legislation the provisions of this Directive shall apply.*"

However, recent rulings of the European Court of Justice (Cases C-140/07 Hecht-Pharma GmbH / Staatliches Gewerbeaufsichtsamt Lüneburg[42] and C-88/07 Commission of the European Communities/ Kingdom of Spain[43]) have given a noteworthy interpretation of Article 2.2 by ruling in favour of products being classified as food supplements.

PRODUCT INFORMATION

The requirements for information on medicinal products (i.e. labels and package leaflets) are laid down in Title V of Directive 2001/83/EC as amended.

A package leaflet for all medicines is mandatory unless all the required information can be conveyed on the outer packaging (i.e. on the label).

Particulars to be included in both labels and package leaflets are set out in Title V.

There are also requirements (laid down in Article 56a) for the name of medicines to appear in Braille on the packaging and for the marketing authorisation holder to ensure that the package information leaflet is made available on request from patients' organisations in a format appropriate for the blind and partially sighted.

The leaflet also needs to undergo patient consultation to ensure that it is legible, clear and easy to use (Article 59).

In addition to the requirements of Articles 54 to 65, any labelling and user package leaflet of a traditional herbal medicine shall contain a statement to the effect that:
(a) the product is a traditional herbal medicinal product for use in specified indication(s) exclusively based upon long-standing use; and
(b) the user should consult a doctor or a qualified health care practitioner if the symptoms persist during the use of the medicinal product or if adverse effects not mentioned in the package leaflet occur.

A Member State may require that the labelling and the user package leaflet should also state the nature of the tradition in question.

Trade name

There are no restrictions at European level regarding the use of the same trade name for a medicine, a food supplement, a cosmetic or a medical device.

Advertising

The provisions concerning the advertising of medicinal products are laid down in Title VIII of Directive 2001/83/EC as amended.

In application of Article 88, all non-prescription medicines can be advertised to the general public. However, Member States have the possibility of prohibiting advertising of reimbursable non-prescription medicines to the general public.

The rules make it clear that the primary role of public advertising is to inform the consumer of the existence of a medicinal product and to incite the consumer to carefully read the product pack information.

In addition to the requirements of Articles 86 to 99, advertisements for traditional herbal medicinal products have to contain the following statement: "*Traditional herbal medicinal product for use in specified indication(s) exclusively based upon long-standing use*".

Distribution

The distribution of medicinal products is not regulated at EU level but remains a national competence.

Useful links

- European Commission – DG Sanco "Health and consumers": http://ec.europa.eu/dgs/health_consumer/index_en.htm
- European Parliament: http://www.europarl.europa.eu/
- European Court of Justice: http://curia.europa.eu/jcms/jcms/Jo1_6308/curia
- European Medicines Agency: http://www.ema.europa.eu/home.htm
- European Heads of Agencies: http://www.hma.eu/human_heads.html
- Council of Europe: http://www.coe.int/
- European Pharmacopoeia: http://www.edqm.eu/site/News_and_General_Information-43.html
- EU legislation online: http://eur-lex.europa.eu/

References

1 Directive 2001/83/EC of the European Parliament and of the Council of 6 November 2001 on the Community code relating to medicinal products for human use
http://eur-lex.europa.eu/LexUriServ/LexUriServ.do?uri=CONSLEG:2001L0083:20070126:EN:PDF

2 AESGP on behalf of the European Commission. Herbal medicinal products in the European Union. 1998.
http://ec.europa.eu/enterprise/pharmaceuticals/pharmacos/docs/doc99/herbal_medecines_en.pdf

3 European Medicines Agency Committee on Herbal Medicinal Products (HMPC)
http://www.ema.europa.eu/htms/general/contacts/HMPC/HMPC.html

4 Guideline on the assessment of clinical safety and efficacy in the preparation of Community Herbal monographs for well-established and of Community Herbal monographs / entries to the Community List for Traditional herbal medicinal products/ substances / preparations
http://www.ema.europa.eu/pdfs/human/hmpc/10461305en.pdf

5 Calls for submission of scientific data
http://www.ema.europa.eu/htms/human/hmpc/hmpcdata.htm

6 Procedure on management of proposals submitted by interested parties for Community list entries or Community herbal monographs
http://www.ema.europa.eu/pdfs/human/hmpc/32857507enfin.pdf

7 Reflection paper on the reasons and timelines for revision of final community herbal monographs and Community list entries
http://www.ema.europa.eu/pdfs/human/hmpc/32644007en.pdf

8 Regulatory and procedural guidance documents
http://www.ema.europa.eu/htms/human/raguidelines/herbal.htm

9 Guidance on documentation to be provided by Member States and applicants/ MAHs in support of a simplified registration referral under articles 16c(1)c and 16c(4)
http://www.ema.europa.eu/pdfs/human/hmpc/43112905en.pdf

10 Template for submission of a request for expert advice on herbal medicinal products
http://www.ema.europa.eu/pdfs/human/hmpc/11988905en.pdf

11 Guideline on non-clinical documentation for herbal medicinal products in applications for marketing authorisation (bibliographical and mixed applications) and in applications for simplified registration.
http://www.ema.europa.eu/pdfs/human/hmpc/3211605en.pdf

12 Commission Directive 2003/63/EC of 25 June 2003 amending Directive 2001/83/EC of the European Parliament and of the Council on the Community Code relating to medicinal products for human use
http://eur-lex.europa.eu/LexUriServ/LexUriServ.do?uri=CELEX:32003L0063:EN:NOT

13 Pharmaceutical legislation - Notice to applicants
http://ec.europa.eu/enterprise/sectors/pharmaceuticals/documents/eudralex/vol-2/index_en.htm

14 Guideline on Good Agricultural and Collection Practice (GACP) for starting materials of herbal origin
http://www.ema.europa.eu/pdfs/human/hmpc/24681605en.pdf

15 GMP requirements for herbal medicinal products are specified in Annex 7
http://ec.europa.eu/enterprise/pharmaceuticals/eudralex/vol-4/vol4_an7_2008_09.pdf

16 Guideline on Quality of Herbal Medicinal Products/Traditional Herbal Medicinal Products
http://www.ema.europa.eu/pdfs/human/qwp/281900en.pdf

17 Guideline on Quality of Combination Herbal Medicinal Products / Traditional Herbal Medicinal Products
http://www.ema.europa.eu/pdfs/human/hmpc/21486906enfin.pdf

18 Guideline on Declaration of Herbal Substances and Herbal Preparations in Herbal Medicinal Products/Traditional Herbal Medicinal Products in the SPC
http://www.ema.europa.eu/pdfs/human/hmpc/28753905en2.pdf

19 Guideline on specifications: Test procedures and Acceptance Criteria for Herbal Substances, Herbal Preparations and Herbal Medicinal Products / Traditional Herbal Medicinal Products
http://www.ema.europa.eu/pdfs/human/qwp/282000en.pdf

20 Reflection paper on Markers used for quantitative and qualitative analysis of Herbal Medicinal Products and Traditional Herbal Medicinal Products
http://www.ema.europa.eu/pdfs/human/hmpc/25362907enfin.pdf

21 Reflection Paper on the use of Fumigants
http://www.ema.europa.eu/pdfs/human/hmpc/12556206en.pdf

22 List of questions & answers (Q&A) received during the HMPC assessors training on quality issues emerging for herbal medicinal products
http://www.ema.europa.eu/pdfs/human/hmpc/53130008en.pdf

23 Guideline on non-clinical documentation for herbal medicinal products in applications for marketing authorisation (bibliographic and mixed applications) and in applications for simplified registration
http://www.ema.europa.eu/pdfs/human/hmpc/3211605en.pdf

24 Guideline on the assessment of genotoxicity of herbal substances/preparations
http://www.ema.europa.eu/pdfs/human/hmpc/10707907enfin.pdf

25 Guideline on selection of test materials for genotoxicity testing for traditional herbal medicinal products/herbal medicinal products
http://www.ema.europa.eu/pdfs/human/hmpc/6764409enfin.pdf

26 Reflection paper on the risks associated with furocoumarins contained in preparations of Angelica archangelica L.
http://www.ema.europa.eu/pdfs/human/hmpc/31791306en.pdf

27 Reflection paper on ethanol content in herbal medicinal products and traditional herbal medicinal products used in children
http://www.ema.europa.eu/pdfs/human/hmpc/8511408enfin.pdf

28 Public Statement on "CPMP List of Herbal Drugs with serious risks, dated 1992"
http://www.ema.europa.eu/pdfs/human/hmpc/24673605en.pdf

29 Guideline on the clinical assessment of fixed combinations of herbal substances/herbal preparations
http://www.ema.europa.eu/pdfs/human/hmpc/16632605en.pdf

30 Eudralex – Volume 9A pharmacovigilance guidelines
http://ec.europa.eu/enterprise/sectors/pharmaceuticals/documents/eudralex/vol-9/index_en.htm

31 EMEA public Statement on herbal medicinal products containing Cimicifugae racemosae rhizome (Black cohosh, root)
http://www.ema.europa.eu/pdfs/human/hmpc/26925906en.pdf

32 Public statement on Chamomilla containing herbal medicinal products
http://www.ema.europa.eu/pdfs/human/hmpc/13830905en.pdf

33 Public Statement on the allergenic potency of herbal medicinal products containing soya or peanut protein
http://www.ema.europa.eu/pdfs/human/hmpc/13813905en.pdf

34 Public statement on the use of herbal medicinal products containing asarone
http://www.ema.europa.eu/pdfs/human/hmpc/13921505en.pdf

35 Public statement on the risks associated with the use of herbal medicinal products containing Aristolochia species
http://www.ema.europa.eu/pdfs/human/hmpc/13838105en.pdf

36 Public statement on the use of herbal medicinal products containing pulegone and methofuran
http://www.ema.europa.eu/pdfs/human/hmpc/13838605en.pdf

37 Public statement on Capsicum/ capsaicin containing herbal medicinal products
http://www.ema.europa.eu/pdfs/human/hmpc/13837905en.pdf

38 Public statement on the use of herbal medicinal products containing estragole
http://www.ema.europa.eu/pdfs/human/hmpc/13721205en.pdf

39 Public statement on the use of herbal medicinal products containing methyleugenol
http://www.ema.europa.eu/pdfs/human/hmpc/13836305en.pdf

[40] Public statement on "CPMP list of herbal drugs with serious risks"
http://www.ema.europa.eu/pdfs/human/hmpc/24673605en.pdf

[41] Guideline on the use of the CTD format in the preparation of a registration application for traditional herbal medicinal products
http://www.ema.europa.eu/pdfs/human/hmpc/7104907en.pdf

[42] European Court of Justice – Case C-140/07
http://eur-lex.europa.eu/LexUriServ/LexUriServ.do?uri=OJ:C:2009:102:0003:0004:EN:PDF

[43] European Court of Justice – Case C-88/07
http://curia.europa.eu/jurisp/cgi-bin/form.pl?lang=en&Submit=Rechercher&alldocs=alldocs&docj=docj&docop=docop&docor=docor&docjo=docjo&numaff=C-88/07%20&datefs=&datefe=&nomusuel=&domaine=&mots=&resmax=100

DENMARK

AREA:	43 094.4 KM²
INHABITANTS (2009):	5 506 279
POPULATION DENSITY (2009):	127.8 INHABITANTS PER KM²
GROSS DOMESTIC PRODUCT (2009) (E):	DKK 1 674.86 BILLION = EURO 224.927 BILLION

Sources: Eurostat 2010.

LEGAL FRAMEWORK

PAST

Herbal medicinal products were first mentioned in *Executive Order No. 341 of 30 June 1978*. Formalised marketing authorisation requirements were introduced with *Medicines Act No. 194 of 23 March 1992 as amended by Act No. 452 of 10 June 1992, Executive Order No. 790 of 21 September 1992.*

Since 1 March 1997, herbal medicines can no longer be sold in Denmark without a marketing authorisation/registration. The safety and efficacy requirements for these medicinal products were however less stringent than for medicines of chemical origin and the regulatory requirements were based on bibliographic applications with indications pertaining to minor illnesses.

PRESENT

The new *Medicines Act No. 1180 of 12 December 2005*[1] which transposed and implemented both Directives 2004/27/EC and 2004/24/EC, together with executive orders, entered into force on 17 December 2005 and was later on amended by *Act No. 538 of 8 June 2006* and *Act No. 1557 of 20 December 2006*. A *Questions & Answers section*[2] provides more information on the new Act.

CLASSIFICATION

All herbal medicinal products currently on the market have a marketing authorisation. A few applications for traditional herbal medicinal products have been submitted to the Danish authorities but no registration has been granted yet.

A list of herbal ingredients that have been evaluated by the Danish Veterinary and Food administration as being safe (part of plant and dosage evaluated) has been established[3]. This list is not exhaustive.

The Danish Medicines Agency is the deciding authority in borderline cases. The physiological function and the presentation of the product are determining factors.

TRANSITIONAL PERIOD

Applications submitted before 29 October 2005 for a plant which is listed on the EU Commission list of traditional herbal ingredients should apply for a re-classification no later than 30 April 2010 if they wish to market the product after 30 April 2011.

DOSSIER

The Danish Medicines Agency states the requirements for the manufacture, quality, safety and efficacy of the product. Herbal medicinal products may only be manufactured in companies that are continually supervised by the pharmaceutical authorities. The requirements for herbal medicinal products follow the Directive and the HMPC *'Guideline on the use of the CTD format in the preparation of a registration application for traditional herbal medicinal products'*.

According to the Danish Medicines Agency, herbal medicinal products should be documented in the same way as other medicinal products i.e. full requirements with regard to modules 1 (administrative data), 2 (summaries and overviews) and 3 (quality) with full GMP requirements for active substances, validated analytical methods for all active ingredients both as raw material and in the finished product and ICH stability testing carried out with a marker substance. The content of Modules 4 (safety) and 5 (efficacy) depends on the legal basis chosen i.e. it can be based on literature alone, on new studies or on a combination of both.

Evaluation process

All herbal medicinal products are registered/authorised by the Danish Medicines Agency. There are dedicated assessors for (traditional) herbal medicines. The evaluation process is the same as for other medicinal products.

FEES[4] *(fees applying as of 1 January 2010)*

Marketing authorisation application

- National applications:
  - Application for marketing authorisation (full and bibliographic applications): DKK 76,326 (approximately € 10,250)
  - Annual fee (for each pharmaceutical form and strength): DKK 4,136 (approximately € 555)
- Mutual Recognition procedure where Denmark is acting as
  - CMS
    - Application for marketing authorisation (full and bibliographic applications): DKK 65,083 (approximately € 8,740)
    - Annual fee (for each pharmaceutical form and strength): DKK 8,549 (approximately € 1,150)
  - RMS
    - Application for marketing authorisation (full and bibliographic applications): DKK 68,725 (approximately € 9,230)
- Decentralised procedure where Denmark is acting as
  - CMS
    - Application for marketing authorisation (full and bibliographic applications): DKK 153,929 (approximately € 20,672)
    - Annual fee (for each pharmaceutical form and strength): DKK 8,549 (approximately € 1,150)
  - RMS
    - Application for marketing authorisation (full and bibliographic applications): DKK 242,028 (approximately € 32,503)
    - Annual fee (for each pharmaceutical form and strength): DKK 8,549 (approximately € 1,150)

Bibliographic application for herbal medicinal products:

- Application for national marketing authorisation: DKK 13,041 (approximately € 1,751)
- Annual fee (for each pharmaceutical form and strength): DKK 4,136 (approximately € 555)

Registration application for traditional herbal medicinal products:

- Application for national marketing authorisation: DKK 7,499 (approximately € 1,007)
- Annual fee (for each pharmaceutical form and strength): DKK 4,136 (approximately € 555)

LIST OF TRADITIONAL HERBAL MEDICINAL PRODUCTS REGISTERED

An overview of all the herbal medicinal products that are authorised in Denmark is published on the Danish Medicines Agency's website[5]. The list will eventually include traditional herbal medicinal products, as there are pending applications but so far no such product has been registered in Denmark.

Product Information

Herbal medicinal products and traditional herbal medicinal products must have a summary of product characteristics, labelling and patient information leaflet as all other medicines.

The provisions on patient information implementing Directive 2004/27/EC are laid down in the new *Medicines Act No. 1180* which entered into force on 17 December 2005.

Since the end of 1998, all medicines should contain a package leaflet. For new medicines, the patient information leaflet should be submitted together with the application dossier. With regard to patient consultation, one single language will be sufficient. The results of the user testing should be provided in Danish or English.

The language used for product labelling and in the package leaflet has to be Danish. The same outer packaging can be used in all Nordic countries (Denmark, Norway, Sweden, Finland and Iceland), provided that it is multilingual even when a different national leaflet is included in the pack. If the summary of product characteristics (SmPC) is identical in several countries, it is possible to include all the package leaflets approved on the basis of the summary of product characteristics in a multilingual package without further approval from the Danish Medicines Agency. However, such approval is usually needed by the other Scandinavian countries, which may create difficulties if the authorities do not agree.

The Braille requirements apply as for any other medicines categories.

Trade Name

Herbal and traditional herbal medicines are subject to the same principles which apply to medicines in general.

The use of trademarks is governed by the *Danish Trademarks Act*[6] which states that a trademark should not be misleading, offensive or similar to another company's name.

No special written rules or guidelines on trademarks for medicines have been issued. A trade name needs to be approved by the Danish Medicines Agency, and approval is not granted if the name is misleading or if it is too close to that of an already marketed product.

Umbrella branding is not allowed in Denmark.

Advertising

The following general advertising rules apply, without exemptions, to herbal medicinal products and traditional herbal medicinal products.

The provisions for pharmaceutical advertising are laid down in the *Medicines Act*; *Executive order No. 793 of 10 September 2001*; and in the *Guidance on the advertising of medicines No. 166 of 5 October 1998*.

Non-prescription medicines can be advertised in all media in Denmark, including TV.

The mandatory text for advertising in the electronic media (TV, radio, film, and video) differs from the one required in printed advertisements.

The following information should be included in advertisements:

- TV – The advertisement must include the following details in text or in speech:
  - Name and use of the medicinal product
  - If appropriate, significant side effects
  - Invitation to read the patient information leaflet
  - Invitation to read more information about the medicinal product on teletext or on the website of the company marketing the product.
- Radio – Complete product information, as required for televised advertisements, is not mandatory, but a statement that further information is available in the pharmacy should be included.
- Printed media – The advertisement must include the following details:
  - Name of the medicinal product
  - Pack size and price including VAT
  - Invitation to read the instructions for use / guidance
  - Effects, side-effects and dosage
  - Other information necessary for the correct use of the product.

Following a specific agreement, public advertising is monitored and assessed by a committee under LIF, the Danish Association of the Pharmaceutical Industry. The authorities are only asked to intervene if LIF finds that the legislation has been infringed. Besides this, the Danish Medicines Agency is only involved if a competitor files a complaint.

The advertising of OTC products to health professionals has to follow the same rules as those for prescription-only medicines.

Comparative advertising is allowed in Denmark as long as it comprises every important competitor on the market and every aspect of the product i.e. not only the beneficial characteristics can be put forward, but also potential disadvantages must be presented.

Distribution

Herbal medicinal products as well as highly-dosed vitamins and minerals and homeopathic medicines can be sold both in pharmacies and in all other shops (mainly health stores and supermarkets).

Other Information

Taxes

The VAT rate on herbal medicinal products and traditional herbal medicinal products is 25% in Denmark.

LINKS

- LIF:
  http://www.lifdk.dk/
- Danish Medicines Agency:
  http://www.laegemiddelstyrelsen.dk / http://www.dkma.dk/1024/visUKLSForside.asp?artikelID=728 (English)
- Pharmaceutical legislation:
  http://www.dkma.dk/1024/visUKLSArtikel.asp?artikelID=742
- Ministry of Health and Disease Prevention:
  http://www.im.dk
- National Board of Health:
  http://www.sst.dk
- Retsinformation, the official online legal information system of the Danish State:
  http://www.retsinfo.dk/

AESGP MEMBER

Ferrosan
Sydmarken 5
DK-2860 Soeborg
Denmark
Phone: +45 39 69 21 11
Fax: +45 39 69 22 41
E-mail: info@ferrosan.com
Website: www.ferrosan.com

ACKNOWLEDGEMENTS

Marianne Hauge, Nycomed Denmark
Steffen Bager, Danish Medicines Agency / Member of [illegible] European Medicines Agency's Committee on Herbal Med[illegible]nal Products

REFERENCES

1 **New Medicines Act No. 1180 of 12 December 2005**
http://lms-lw.lovportaler.dk/ShowDoc.aspx?docId=lov20051180uk-full

2 **Questions and Answers Section on the New Medicines Act**
http://www.dkma.dk/1024/visUKLSArtitkel.asp?arikelID=8094

3 **List of herbal ingredients evaluated by the Danish Veterinary and Food administration as being safe**
http://www.dfvf.dk/Admin/Public/DWSDownload.aspx?File=Files%2fFiler%2fPublikationer%2fdrogeliste%2frapport.pdf

4 **Fees**
http://www.dkma.dk/db/filarkiv/6682/bek_om_%20gebyrer_for_laegemidler_inkl_bilag_1_UK.pdf (fees in Appendix 1)

5 **Overview of all herbal medicinal products authorised in Denmark**
http://www.dkma.dk/db/filarkiv/6545/naturlaegemidler.pdf

6 **Danish Trademarks Act**
http://www.wipo.int/clea/docs_new/en/dk/dk058en.html

INDIA

AREA:	2 973 190 KM²
INHABITANTS (2009):	1 207 497 000
POPULATION DENSITY (2009):	406 INHABITANTS PER KM²
GROSS DOMESTIC PRODUCT (2009) (E):	INR 56 764.66 BILLION = EURO 842.69 BILLION

Sources: Eurostat 2010.

LEGAL FRAMEWORK

India has a federal form of government and the medical regulatory structure is divided between national and state authorities. The principal national drug authority based in New Delhi is the *Central Drug Standards Control Organisation* (CDSCO). CDSCO is controlled by the Drug Controller General of India (DCGI). There are also 35 *state-level Food and Drug Administrations*, one per each India's State and Union Territory.

The DCGI registers all imported drugs, new drugs, biological and drugs in selected categories. It also has responsibility for medical devices, clinical trials and quality standards.

The state-FDAs register all other products, accredit manufacturing plants and conduct the bulk of quality monitoring and inspections.

An *Ayurvedic, Siddha and Unani Drugs Consultative Committee* is appointed by the Central government to advise the Central Government, the State Governments and the Ayurvedic, Siddha and Unani Drugs Technical Advisory Board on any matter for the purpose of securing uniformity throughout India in the administration of this Act insofar as it relates to Ayurvedic, Siddha or Unani drugs. This Committee is composed of two persons nominated by the Central Government as representatives of that Government and one representative of each State.

LEGAL BASIS

All regulatory aspects related to the manufacture, sale, import, export and clinical research of drugs and cosmetics are covered under the following Acts and Rules:

- the Drug and Cosmetic Act, 1940 (D and C Act)[1];
- the Pharmacy Act, 1948
- the Medicinal and Toilet Preparations (excise duties) Act 1956,
- the Drugs and Magic Remedies (objectionable advertisements), Act 1954
- the Narcotic Drugs and Psychotropic Substances Act, 1985, and
- the Drugs (prices control) Order 1995 (under the Essential Commodities Act)

The legislations apply to the whole of India and to all categories of medicines (e.g. allopathic, Ayurvedic, Siddha, Unani and homeopathy), whether imported or manufactured in India. The legislation is enforced by the Central Government in New Delhi, which is responsible for its overall supervision. The First four Acts are implemented by the Ministry of Health & Family Welfare, whilst the 5th Act is implemented by the Finance Ministry and the last one by the Ministry of Chemical & Fertilizers.

DEFINITIONS

Drug includes

- all medicines for internal or external use in human beings or animals and all substances intended to be used for or in the diagnosis, treatment, mitigation or prevention of any disease or disorder in human beings or animals, including preparations applied on human body for the purpose of repelling insects like mosquitoes;
- such substances (other than food) intended to affect the structure or any function of the human body or intended to be used for the destruction of vermin or insects which cause disease in human beings or animals, as may be specified from time to time by the Central Government by notification in the Official Gazette;
- all substances intended for use as components of a drug including empty gelatin capsules; and
- such devices intended for internal or external use in the diagnosis, treatment, mitigation or prevention of disease or disorder in human beings or animals, as may be

specified from time to time by the Central Government by notification in the Official Gazette, after consultation with the Board.

Ayurvedic, Siddha or Unani drug includes all medicines intended for internal or external use for or in the diagnosis, treatment, mitigation or prevention of disease or disorder in human beings or animals, and manufactured exclusively in accordance with the formulae described in the authoritative books of Ayurvedic, Siddha and Unani Tibb systems of medicine, specified in the First Schedule.

- *Ayurveda*, first described in Vedic religious scriptures dating from 6000 B.C. is considered the traditional medicine of India. Central to Ayurvedic philosophy is the belief that optimal health consists of physical, mental and spiritual harmony. The pathway to harmony depends on the individual's predominant dosha, or constitution. Ayurvedic practitioners interview new patients in great detail about their personal as well as medical history. The four pillars of Ayurvedic health maintenance are: (1) cleansing and detoxification, (2) palliation, (3) rejuvenation, and (4) mental and spiritual hygiene. Diet is an important concern in Ayurveda, but specific dietary recommendations depend on the individual's primary constitution, and vary according to the season. Treatment may include dietary modification, herbal preparations, massage, yoga, meditation, and pranayama, or breathing exercises.
- *Siddha medicine* is one of the most ancient medical systems of India. Siddha is the mother medicine of ancient Tamils/ Dravidians of peninsular South India. The word 'Siddha' means established truth. The persons who were associated with establishing such a Siddha school of thought were known as Siddhars. They recorded their mystic findings in medicine, yoga, and astrology in Tamil. Fundamental Principles of Siddha include theories of Five Elements (Aimpootham), and Three Forces/ Faults (Mukkuttram). The Eight Methods of Examination (Envakai Thervukal) are used to determine diagnosis, etiology, treatment and prognosis. Siddha is based on herbal and herbo-mineral treatments. Lifestyle modifications including diet are important.
- *Unani* is a traditional healing system prevalent in India according to which the body comprises four basic elements —earth, air, water, and fire— and four humors —blood, phlegm, yellow bile and black bile. An equilibrium in the humors indicates good health while a disturbance in this equilibrium results in disease.

CLASSIFICATION

The D & C Act 1940 has listed drugs in schedules. Prescription-only medicines are listed in Schedules H and X. Drugs listed in Schedule G (mostly antihistamines) do not need a prescription for purchase but require the following mandatory text on the label "caution: it is dangerous to take this preparation except under medical supervision". Drugs falling in the above three schedules are currently not advertised to the public under voluntary commitment by the pharmaceutical industry.

Contrary to allopathic and homeopathic medicines, Ayurvedic, Siddha and Unani drugs do not require a license prior to marketing. No Ayurvedic, Siddha and Unani drugs are included in Schedule H or X.
All medicinal products not included in the list of 'prescription-only drugs' are considered as non-prescription drugs (OTCs) in India, though the term OTC drug has no legal recognition.

Medicines registered as Ayurvedic, Siddha or Unani Medicines are also regulated by the D & C Act and D & C Rules. Ayurvedic drugs are manufactured for sale under a manufacturing licence issued by the Ayurvedic State Licensing Authorities.
No drugs are allowed to be sold as "herbal medicines", as there is no such provision in the existing legislation in India. Drugs of herbal origin can be sold in the name of "Ayurvedic/Siddha/ Unani", if they are manufactured as per the conditions prescribed by the law and have a manufacturing licence for Ayurvedic/ Siddha/Unani. But presently, this is an important issue which is under serious consideration of the concerned authority.

Some of the largest OTC brands in India (e.g. Vicks VapoRub, Amrutanjan Pain Balm, Zandu Pain Balm, Iodex Pain Balm, Moov Pain Cream, Itch Guard Cream, Eno Fruit Salt antacid, Vicks Cough Drops, Halls Lozenges, Dabur's Pudina Hara etc.), are registered as 'Ayurvedic Medicines' because of their herbal-based active ingredients.

LICENSING & LICENSING REQUIREMENTS

QUALITY

Medicines registered as Ayurvedic, Siddha or Unani Medicines are subject to quality requirements and need to be manufactured according to GMP standards as stipulated in Schedule T. Drugs included in Ayurvedic pharmacopoeia have to fulfil the standards for identity, purity and strength as given in the editions of Ayurvedic Pharmacopoeia of India in force for the time being.

For preparations (Asavas and Arishtas), the upper limit of alcohol as self-generated alcohol should not exceed 12% v/v excepting those that are otherwise notified by the Central Government from time to time.

Note: Asavas and Aristas are medicinal preparations made by soaking the drugs, either in powder form or in the form of decoction (Kasaya), in a solution of sugar or jaggery, as the case may be, for a specified period of time, during which it undergoes a process of fermentation generating alcohol, thus facilitating the extraction of the active principles contained in the drugs. The alcohol, so generated, also serves as a preservative. (Ref. The Ayurvedic Formulary of India Part-I, First Edition, Govt. of India, 1978)

So called **"modern medicines"** must comply with the standards laid down in the Second Schedule of the D & C Act and in Schedule V of the D & C rules. Patent or proprietary medicines (other than homeopathic medicines) must have the formula of list of ingredients displayed on the label of the container. They also need to meet the identity, purity and strength requirements specified in the Indian Pharmacopoeia or in an official Pharmacopoeia of any other country.

GMP

In order to obtain a certificate of 'Good Manufacturing Practices' for **Ayurveda-Siddha-Unani drugs**, the applicant shall file an application providing the information on existing infrastructure of the manufacturing unit, and the licensing authority shall, after verification of the requirements as per Schedule T, issue the certificate within a period of 3 months.

The manufacture of Ayurvedic (including Siddha) or Unani drugs shall be conducted under the direction and supervision of competent technical staff consisting of at least one person, who is a whole time employee and who

- possesses qualifications in Ayurveda or Ayurvedic Pharmacy, Siddha or Unani system of medicine; or
- has a diploma in Ayurveda, Siddha or Unani medicine; or
- is graduated in Pharmacy or Pharmaceutical Chemistry or Chemistry or Botany; or
- is a Vaid or Hakim having experience of at least four years; or
- is an Ayurvedic or Unani Pharmacist with at least 8 years experience

Its competent technical staff to direct and supervise the manufacture of Ayurvedic, Siddha or Unani drugs shall also have the respective qualifications.

The licensee shall maintain proper records of the details of manufacture and of the tests, if any, carried out by him or by any other person on his behalf, of the raw materials and finished products and allow an Inspector appointed under the Act to inspect the premises, to take samples of the raw material as well as finished products and to inspect the records maintained under these rules.

The licensee shall maintain an Inspection Book to enable an Inspector to record his impressions and the defects noticed.

In accordance with rules 71, 74, 76 and 78, licensees of **modern medicines** have to comply with the requirements of "Good manufacturing practices" as laid down in Schedule M. The first part of Schedule M (Part I) lays down the general requirements and is complemented by specific parts for respectively sterile products, oral solid dosage forms, oral liquids, topical products, metered-dose inhalers, active pharmaceutical ingredients. Part II lays down the requirements for plant and equipments. Schedule M1 is applicable to homeopathic medicines.

SAFETY & EFFICACY

Medicines registered as Ayurvedic, Siddha or Unani Medicines do not have to comply with any specific safety and efficacy requirement. Safety and efficacy are based on traditional and historical use as documented in the Ayurvedic and Siddha systems listed in the First Schedule of the D & C Act.

Medicinal products count as **'new drugs'** in India if they fall into one of the following categories:

- drugs not previously available on the market in India
- drugs with new therapeutic indications or dosages that have not been marketed in India
- new fixed-dose combinations of two or more drugs
- any drug which was first approved in India less than four years ago, unless it is included in the Indian pharmacopoeia.

Safety and efficacy requirements for 'new drugs' are laid down in Schedule Y of the D & C Rules.

Schedule Y covers information that needs to figure in the application for permission to import or manufacture new drugs for sale or to perform clinical trials.

A guidance document on the requirements for permission of new drugs approval is available to aid applicants[2]. The format is that of the ICH Common Technical Document.

The regulatory scheme which would need to be applied to be able to market a new occidental herbal-based medicine in India (i.e. scheme applying to Ayurvedic, Siddha or Unani medicines or new drug application) would be decided on a case-by-case basis by the DCGI.

FEES

A fee of Rs. 60 is required for obtaining a manufacturing licence for sale for any Ayurvedic (including Siddha) or Unani Drugs.

A new drug registration carries a fee of 50,000 rupees.

EVALUATION

No pre-marketing evaluation is taking place for Ayurvedic, Unani and Siddha medicines.

For new drugs, there is no fixed timeframe in the legislation but a typical range is about 12-18 months. In case of a new drug application, the DCGI would evaluate the application according to a procedure similar to that of a modern medicine.

PRODUCT INFORMATION

Rule 96 of the D & C Rules ('Manner of Labelling') mandates the minimum information which needs to be put on the label of all medicines other than Ayurvedic, Siddha and Unani medicines.

This includes:

- proper (generic) and trade (brand) name
- net contents and content of active ingredients,
- name and address of manufacturer including manufacturing licence number,
- distinctive batch number, manufacturing and expiry date etc.
- Maximum Retail Price (inclusive of all taxes)

The labelling provisions of Ayurvedic, Siddha and Unani medicines are covered by Rule 161 *'Labelling, packing and limit of alcohol'*.
All the ingredients used in the manufacture of the preparation together with the quantity of each of the ingredients incorporated therein and a reference to the method of preparation thereof as detailed in the reference books specified in the First Schedule to the Act shall be listed.
If the list of ingredients contained in the medicine is large and cannot be accommodated on the label, the same may be printed separately and enclosed with packing and reference be made to this effect on the label.
The container of a medicine for internal use shall, if it is made up from a substance specified in Schedule E (1), be labelled clearly with the words 'Caution: To be taken under medical supervision' both in English and Hindi language. This is simply a word of caution to the patient as the medicine is legally available without any prescription.

In addition, the following particulars shall be either printed or written in indelible ink and shall appear in a clear manner on the label of the immediate and secondary pack of the medicine:

- Name of the drug. For this purpose the name shall be the same as mentioned in the reference books included in the First Schedule of the Act.
- A correct statement on the net content in terms of weight, measure or number as the case may be.
- The name and address of the manufacturer
- The number of the licence under which the drug is manufactured, the figure representing the manufacturing license number being preceded by the words "manufacturing license number" or "Mfg. Lic. No." or "M.L".
- Batch number
- The date of manufacture
- The words "Ayurvedic medicine" or "Siddha medicine" or "Unani medicine" as the case may be.
- The words "For external use only" if the medicine is for external application

TRADE NAME

Trade names are regulated by the *Trade and Merchandise Marks Act* (TMMA). The TMMA provides for registration of trademarks for a period of seven years at a time, renewable after each period. For any item, trademarks should not be objectionable from a religious or social point of view. They should not contravene the *Emblems and Names (Prevention of Improper Use) Act*, 1950. They should also not yet be registered or applied to be registered in India. The trademark can be registered even if the item is not produced or sold in India at present. This Act has been replaced by the *Trademarks Act, 1999*.

A foreign trademark can be used without any restriction. Foreign companies can license their trademark to their local subsidiaries or joint ventures. The *Indian Copyright Act*[3] also provides protection for unique logos and designs on packaging.

Look-alike copies of popular non-prescription drugs are a major issue because the licence to manufacture and sell drugs is issued by state-level FDAs who do not independently verify whether they issue a manufacturing and selling licence to a look-alike drug. However, the Indian courts are known to provide quick and corrective action against a look-alike product, although the burden of search and taking the pass off manufacturer to court falls on the individual affected company.

There is no special provision for Ayurvedic, Siddha or Unani medicines; their names are usually those described in historical reference books.

ADVERTISING

The *Drug & Magic Remedies (Objectionable Advertisement) Act* & Rules mentions a list of ailments for which no advertising is permitted. It also prohibits false or misleading advertisements which, directly or indirectly, give false impressions regarding the true character of the drug, make false claims, or are otherwise false or misleading in any particular respect. The DCGI's office - in collaboration with the *Organisation of Pharmaceutical Producers of India* (OPPI) - has released a *Voluntary Code on OTC Advertising* which is being followed by all OPPI member companies. There is also an OPPI *Code of Pharmaceutical Marketing Practices, January 2007*[4], based on the IFPMA code. Based on the DCGI code, the Advertising Standards Council of India (ASCI) has brought out a code of advertising for pharmaceutical products.

Ayurvedic, Siddha and Unani medicines and preparations can be advertised on TV in India.

DISTRIBUTION

Drug products can only be sold through retail pharmacies and licensed stores. Ayurvedic, Unani and Siddha medicines can be sold freely by non-chemists.

Distance selling and teleshopping of non-prescription medicines is not permitted in India.

OTHER INFORMATION

TAXES

The tax structure is quite complicated. In general 4% VAT is applicable for all formulated medicines. Products containing alcohol generally have to pay excise duties, although provisions specific to Union territories and States may apply.

LINKS

- Ministry of Health and Family Welfare:
  http://mohfw.nic.in/
- Director General of Health Services:
  http://mohfw.nic.in/ph/tdghs.htm
- Central Drugs Standard Control Organization (CDSCO)/ Drugs Controller General of India:
  http://cdsco.nic.in/index.html
- Medicines laws:
  http://cdsco.nic.in
- Department of Chemicals (Ministry of Chemical & Fertilizers):
  http://chemicals.nic.in/
- National Pharmaceutical Pricing Authority (NPPA):
  http://nppaindia.nic.in/index1.html
- Department of Ayurveda, Yoga & Naturopathy, Unani, Siddha and Homeopathy
  http://indianmedicine.nic.in/html/ayurveda/afmain.htm
- Department of Pharmaceuticals:
  http://pharmaceuticals.gov.in/
- Controller General of Patents, Designs and Trademarks:
  http://www.patentoffice.nic.in/tmr_new/default.htm

TRADE NAME

Trade names are regulated by the *Trade and Merchandise Marks Act* (TMMA). The TMMA provides for registration of trademarks for a period of seven years at a time, renewable after each period. For any item, trademarks should not be objectionable from a religious or social point of view. They should not contravene the *Emblems and Names (Prevention of Improper Use) Act, 1950*. They should also not yet be registered or applied to be registered in India. The trademark can be registered even if the item is not produced or sold in India at present. This Act has been replaced by the *Trademarks Act, 1999*.

A foreign trademark can be used without any restriction. Foreign companies can license their trademark to their local subsidiaries or joint ventures. The *Indian Copyright Act*[3] also provides protection for unique logos and designs on packaging.

Look-alike copies of popular non-prescription drugs are a major issue because the licence to manufacture and sell drugs is issued by state-level FDAs who do not independently verify whether they issue a manufacturing and selling licence to a look-alike drug. However, the Indian courts are known to provide quick and corrective action against a look-alike product, although the burden of search and taking the pass off manufacturer to court falls on the individual affected company.

There is no special provision for Ayurvedic, Siddha or Unani medicines; their names are usually those described in historical reference books.

ADVERTISING

The *Drug & Magic Remedies (Objectionable Advertisement) Act & Rules* mentions a list of ailments for which no advertising is permitted. It also prohibits false or misleading advertisements which, directly or indirectly, give false impressions regarding the true character of the drug, make false claims, or are otherwise false or misleading in any particular respect. The DCGI's office - in collaboration with the *Organisation of Pharmaceutical Producers of India* (OPPI) - has released a *Voluntary Code on OTC Advertising* which is being followed by all OPPI member companies. There is also an OPPI *Code of Pharmaceutical Marketing Practices, January 2007*[4], based on the IFPMA code. Based on the DCGI code, the Advertising Standards Council of India (ASCI) has brought out a code of advertising for pharmaceutical products.

Ayurvedic, Siddha and Unani medicines and preparations can be advertised on TV in India.

DISTRIBUTION

Drug products can only be sold through retail pharmacies and licensed stores. Ayurvedic, Unani and Siddha medicines can be sold freely by non-chemists.

Distance selling and teleshopping of non-prescription medicines is not permitted in India.

OTHER INFORMATION

TAXES

The tax structure is quite complicated. In general 4% VAT is applicable for all formulated medicines. Products containing alcohol generally have to pay excise duties, although provisions specific to Union territories and States may apply.

LINKS

- Ministry of Health and Family Welfare: http://mohfw.nic.in/
- Director General of Health Services: http://mohfw.nic.in/ph/tdghs.htm
- Central Drugs Standard Control Organization (CDSCO)/ Drugs Controller General of India: http://cdsco.nic.in/index.html
- Medicines laws: http://cdsco.nic.in
- Department of Chemicals (Ministry of Chemical & Fertilizers): http://chemicals.nic.in/
- National Pharmaceutical Pricing Authority (NPPA): http://nppaindia.nic.in/index1.html
- Department of Ayurveda, Yoga & Naturopathy, Unani, Siddha and Homeopathy http://indianmedicine.nic.in/html/ayurveda/afmain.htm
- Department of Pharmaceuticals: http://pharmaceuticals.gov.in/
- Controller General of Patents, Designs and Trademarks: http://www.patentoffice.nic.in/tmr_new/default.htm

WSMI MEMBER ASSOCIATION

Organisation of Pharmaceutical Producers of India (OPPI)
Peninsula Chambers, Ground Floor, Ganpatrao Kadam Marg,
Lower Parel
Mumbai 400 013
India
Phone: +91 22 / 2491 8123 – 2491 2486 – 6662
7007
Fax: +91 22 / 2491 5168
E-mail: indiaoppi@vsnl.com
Website: www.indiaoppi.com

REFERENCES

1 Drug and Cosmetic Act, 1940 and Drug and Cosmetic Rules, 1945
http://cdsco.nic.in/Drugs&CosmeticAct.pdf

2 Guidance for industry – requirements for permission of New Drugs Approval
http://cdsco.nic.in/CDSCO-GuidanceForIndustry.pdf

3 Indian Copyright Act
http://www.education.nic.in/CprAct.pdf

4 OPPI Code of Pharmaceutical Marketing Practices, January 2007
http://www.indiaoppi.com/OPPI%20Code%20of%20Marketing%202007.pdf

UNITED STATES OF AMERICA

AREA:	9 629 091 KM²
INHABITANTS (2009):	307 199 900
POPULATION DENSITY (2009):	31.9 INHABITANTS PER KM²
GROSS DOMESTIC PRODUCT (2009) (E):	USD 14 162.26 BILLION = EURO 10 153.744 BILLION

Sources: Eurostat 2010.

LEGAL FRAMEWORK

LEGAL BASIS

The *Federal Food, Drug, and Cosmetic Act* (FD&C Act)[1] is a set of laws enacted by Congress giving authority to the U.S. Food and Drug Administration (FDA)[2] to regulate food, drugs, and cosmetics. Recently, it was amended by the *Food and Drug Administration Amendments Act* of 2007 (FDAAA)[3] which, among other things, reauthorised the *Best Pharmaceuticals for Children Act* (BPCA) and the *Pediatric Research Equity Act* (PREA) and expanded the *Prescription Drug User Fee Act* (PDUFA).

The FDA issues FDA regulations regrouped in Title 21 of the *Code of Federal Regulations* (CFR)[4] and guidance documents which describe the Agency's current thinking on a regulatory issue. Whilst regulations are binding, guidance documents are not.

The U.S. FDA is an agency within the U.S. Department of Health and Human Services, one of the United States federal executive departments, responsible for protecting and promoting public health through the regulation and supervision of prescription and over-the-counter pharmaceutical drugs, food safety, dietary supplements, vaccines, biopharmaceuticals, blood transfusions, medical devices, electromagnetic radiation emitting devices, veterinary products, cosmetics, and tobacco products. It consists of six product centres (of which the Center for Drug Evaluation and Research (CDER) which regulates prescription and over-the-counter medicines), one research centre, and two offices.

DEFINITIONS

Drug is defined as follows in the FD&C Act:
(A) articles recognised in the official United States Pharmacopoeia, official Homoeopathic Pharmacopoeia of the United States, or official National Formulary, or any supplement to any of them; and (B) articles intended for use in the diagnosis, cure, mitigation, treatment, or prevention of disease in man or other animals; and (C) articles (other than food) intended to affect the structure or any function of the body of man or other animals; and (D) articles intended for use as a component of any article specified in clause (A), (B), or (C). A food or dietary supplement for which a claim, subject to sections 403(r)(1)(B) and 403(r)(3) or sections 403(r)(1)(B) and 403(r)(5)(D), is made in accordance with the requirements of section 403(r) is not a drug solely because the label or the labelling contains such a claim. A food, dietary ingredient, or dietary supplement for which a truthful and not misleading statement is made in accordance with section 403(r)(6) is not a drug under clause (C) solely because the label or the labelling contains such a statement.

New drug is defined as follows in the FD&C Act:
(1) Any drug (except a new animal drug or an animal feed bearing or containing a new animal drug) the composition of which is such that such drug is not generally recognised, among experts qualified by scientific training and experience to evaluate the safety and effectiveness of drugs, as safe and effective for use under the condition prescribed, recommended, or suggested in the labelling thereof, except that such a drug not so recognised shall not be deemed to be a "new drug" if at any time prior to the enactment of this Act [enacted June 25, 1938] it was subject to the *Food and Drugs Act* of June 30, 1906, as amended, and if at such time its labelling contained the same representations concerning the conditions of its use; or
(2) Any drug (except a new animal drug or an animal feed bearing or containing a new animal drug) the composition of which is such that such drug, as a result of investigations

UNITED STATES OF AMERICA

to determine its safety and effectiveness for use under such conditions, has become so recognised, but which has not, otherwise than in such investigations, been used to a material extent or for a material time under such conditions.

Botanical; Botanical Product: A finished, labelled product that contains vegetable matter, which may include plant materials (see below), algae, macroscopic fungi, or combinations of these. Depending in part on its intended use, a botanical product may be a food, drug, medical device, or cosmetic. Fermentation products and highly purified or chemically modified botanical substances are not considered botanical drug products.

Botanical Drug Product; Botanical Drug: A botanical product that is intended for use as a drug; a drug product that is prepared from a botanical drug substance. Botanical drug products are available in a variety of dosage forms, such as solutions (e.g. teas), powders, tablets, capsules, elixirs, and topicals.

Botanical Drug Substance: A drug substance derived from one or more plants, algae, or macroscopic fungi. It is prepared from botanical raw materials by one or more of the following processes: pulverisation, decoction, expression, aqueous extraction, ethanolic extraction, or other similar process. It may be available in a variety of physical forms, such as powder, paste, concentrated liquid, juice, gum, syrup, or oil. A botanical drug substance can be made from one or more botanical raw materials (see Single-Herb and Multi-Herb Botanical Drug Substance or Product). A botanical drug substance does not include a highly purified or chemically modified substance derived from natural sources.

Botanical Ingredient: A component of a botanical drug substance or product that originates from a botanical raw material.

CLASSIFICATION

The U.S. classifies medicines as either prescription or non-prescription. As with the EU's classification provisions, prescription medicines are specifically defined and non-prescription medicines are those which are not limited to prescription status.

The U.S. *Food, Drug, and Cosmetic Act* (section 503(b)(1) (21 U.S.C. 353(b)(1)) limits to prescription status: "a drug intended for use by man which –

- because of its toxicity or other potentiality for harmful effect, or the method of use, or the collateral measures necessary to its use, is not safe for use except under the supervision of a practitioner licensed by law to administer such drug; or
- is limited by an approved application under section 505 [the section describing the approval process for new drugs] to use under professional supervision of a practitioner licensed by law to administer such drug ...".

A botanical product may be a food (including a dietary supplement), a drug (including a biological drug), a medical device, or a cosmetic under the Act. Whether an article is a drug, medical device, or cosmetic under the Act turns on its "intended use" (21 U.S.C. 312(g)(1)(B) and (C), (h)(2) and (3), (i)).
If a botanical product is intended for use in diagnosing, mitigating, treating, or curing disease, it is a drug under section 201(g)(1)(B) of the Act and is subject to regulation as such.

If a botanical product is intended to prevent disease, it is usually a drug. However, under the *Dietary Supplement Health and Education Act* of 1994 (DSHEA), an orally ingested product that meets the definition of a "dietary supplement" under section 201(ff) of the Act may be lawfully marketed with a statement that (1) claims a benefit related to a classical nutrient deficiency disease (and discloses the prevalence of the disease in the United States); (2) describes how the product is intended to affect the structure or function of the human body, (3) characterises the documented mechanism by which the product acts to maintain such structure or function, or (4) describes general well-being from consumption of the product (section 403(r)(6)(A) of the Act).

If the intended use of a botanical product is to affect the structure or function of the human body, it may be regulated either as a dietary supplement or as a drug, depending on the circumstances.

Some botanical drugs, including elm bark (oral health care, demulcents), pyrethrum extract (pediculicide, non-aerosol combination), and psyllium (laxative) are included in the over-the-counter (OTC) drug monographs. The great majority of botanical products are legally available in the United States as dietary supplements.

Veregen™ was approved by the FDA in October 2006 for the topical treatment of genital wart caused by human papil-

loma virus (HPV) and it is currently the only approved botanical prescription drug through the IND/NDA (Investigational New Drug/New Drug Application) processes since the publication of the botanical guidance. The drug substance in Veregen™ is Kunecatechins, which is a partially purified fraction of the water extract of green tea leaves from *Camellia sinensis* (L.) O Kuntze, and is a mixture of catechins and other green tea components.[5]

LICENSING & LICENSING REQUIREMENTS

GENERAL

A botanical drug product may be marketed in the United States under (1) an OTC drug monograph or, (2) an approved NDA (or even an abridged new drug application – ANDA).

1. OTC monograph

- ***Existing OTC Monograph***

When a final OTC drug monograph is published for a specific use of a botanical drug, any person may market a product containing the same substance and for the same use, provided the labelling and other active ingredients (if present) are in accord with all relevant monographs and other applicable regulations. Any pharmaceutical company can market products containing category I (generally recognised as safe and effective – GRASE) botanical ingredients according to OTC monographs[6].

Code of Federal Regulations (CFR) Part 330[7] is GRASE and is not misbranded if it meets the conditions of 330.1 and each of the conditions contained in the specific final monograph.

Marketing pre-clearance of OTC drug products by the FDA is not required if the standards of the applicable monograph are met.

- ***Inclusion in an OTC Monograph***

A botanical product that has been marketed in the United States for a material time and to a material extent for a specific OTC drug indication may be eligible for inclusion in an OTC drug monograph.

Citizen petition:

A request to amend an OTC drug monograph to include a botanical substance must be submitted by citizen petition in accordance with §§ 10.30[8] and 330.10(a)(12).

For a botanical drug substance to be included in an OTC drug monograph, there must be published data establishing general recognition of safety and effectiveness, usually including results of adequate and well-controlled clinical studies. Requirements related to safety, effectiveness, and labelling for drugs to be included in an OTC drug monograph are set forth in 21 CFR Part 330.

There should be publicly available quality standards for such a botanical drug substance in the drug section (i.e. not in the National Formulary or other non-drug sections) of the *United States Pharmacopeia* (USP). In the absence of a USP drug monograph, the petitioner should include suitable quality standards for the botanical drug substance in its citizen petition and simultaneously propose adoption of those standards in the USP.

If requirements are fulfilled and FDA believes the request should be granted, the Agency could issue a notice of proposed rulemaking (proposed rule) that states the proposed action and explains the reason for the action. FDA would then receive comments to the proposed rule, review these comments and publish a final rule that would amend the monograph or withdraw the proposed rule. All steps in the petition process are open to the public, and petitions do not require payment of a monetary fee to FDA. Although response letters are generally sent to the sponsor of a petition within 180 days, the public rulemaking process to amend the monograph for an OTC drug product historically has taken more time than a final decision on an NDA. After a condition has been incorporated into the final monograph for an OTC drug product (or in a notice of enforcement policy under 21 CFR 330.14(h) if a finalisation of the monograph is not imminent), it can be marketed by any interested party without prior approval by FDA.

Currently, there are several botanical drugs, including cascara, psyllium, and senna, that are included in the OTC drug review.

Conditions covered by existing proposed or final OTC drug monographs are handled through the submission of a citizen petition as described in § 10.30 (21 CFR 10.30). To request that a new condition be considered for inclusion in the OTC drug monograph system, a time and extent application (TEA) has to be submitted.

> *Note: A condition refers to an active ingredient or botanical drug substance (or combination of both), dosage form, dosage strength, or route of administra-*

tion marketed for a specific over-the-counter use. This includes conditions regulated as cosmetic products or dietary supplements in a foreign country(ies) that would be regulated as OTC drugs in the United States.

Time and extent application:
The final regulations (21CFR 330.14) for criteria and procedures for a Time and Extent Application (TEA) were published in the *Federal Register* on January 23, 2002. A copy of the Final Rule[9] and the draft Guidance for Industry for TEA applications[10] is available as well.

A TEA would only be submitted for conditions that the applicant believes have been marketed OTC to a material extent and for a material time as follows:

- conditions initially marketed (under an NDA) in the United States after the OTC drug review began in 1972 – previously, companies were required to submit an NDA if their OTC drug products were initially marketed in the United States after the beginning of the OTC drug review in 1972.
- conditions marketed only outside the United States (and that would be regulated as OTC drugs in the United States) - Previously, if an OTC drug condition had been marketed solely in a foreign country, a firm was required to submit a new drug application (NDA) before the condition could be marketed in the United States.
- conditions not generally recognised as safe and effective (i.e. *non-monograph*) in the original OTC drug review but conditions for which additional data and information are being presented.

Under § 330.14(b), the conditions must be marketed for OTC purchase by consumers and for at least 5 continuous years in the same country in sufficient quantity (although more than one country may be appropriate depending on the extent of marketing).

The TEA is the first step in a two-step process. If a condition is determined to be eligible for inclusion in the monograph system, the TEA would be placed on public display and a notice of eligibility would be published in the *Federal Register*. This notice would request data that demonstrate general recognition of safety and efficacy for the condition.
The second step of the process, requiring demonstration of general safety and effectiveness, follows.

After reviewing the data, if appropriate, FDA would publish a proposed rule and then a final rule incorporating the new condition. Similar to a petition, FDA would typically respond to a TEA within 180 days, and a monetary fee would not be required.

After a condition has been incorporated into a final monograph (or in a notice of enforcement policy under 21 CFR 330.14(h), if a finalisation of the monograph is not imminent), the condition could be marketed by any interested party without prior approval by FDA. For marketing to occur, § 330.14(i) requires that the active ingredient or botanical drug substance be recognised in the U.S. Pharmacopeia-National Formulary (USP-NF).

2. New drug application (NDA)

Under current regulations, if there is no marketing history in the United States or a foreign country for a botanical drug product, if available evidence of safety and effectiveness does not warrant inclusion of the product in an OTC drug monograph, or if the proposed indication would not be appropriate for non-prescription use, the manufacturer must submit an NDA to obtain FDA approval to market the product for the proposed use (sections 201(p) and 505 of the Act). An NDA for a botanical drug could seek approval for either prescription or OTC use, depending on the indication and characteristics of the product and whether it is safe for use outside of the supervision of a practitioner licensed by law to administer it. If existing information on the safety and effectiveness of a botanical drug product is insufficient to support an NDA, new clinical studies are recommended to be conducted to demonstrate safety and effectiveness.

When a product is approved under an NDA, the approval is specific to the drug product that is the subject of the application (the applicant's drug product), and the applicant may be eligible for marketing exclusivity for either 5 years (if it is a new chemical entity) or 3 years from the time of approval, even in the absence of patent protection. A new botanical drug (containing multiple chemical constituents) may qualify as a "new chemical entity" under § 314.108(a). If a product qualifies as a new chemical entity, during the period of exclusivity, FDA will not approve, or in some cases even review, certain competitor products unless the second sponsor conducts all studies necessary to demonstrate the safety and effectiveness of its product and submits a 505(b)(1) application. Therefore, if a person wishing to market a botanical drug product that is not included in an existing

OTC drug monograph desires marketing exclusivity for the product, the person should seek approval of an NDA rather than petition the Agency to amend a monograph.

An applicant can also submit an NDA for a new OTC drug condition (21 CFR part 314) or to request approval of an OTC drug product that deviates in any respect from a monograph that has become final (see 21 CFR 330.11).

An NDA seeks approval of a specific product that is formulated and labelled as it is to be marketed. Benefits of this approach include (1) confidentiality during the approval process; (2) a period of marketing exclusivity upon approval if certain conditions are met; and (3) historically, less time for review of the application from submission to a final decision, compared to other routes of approval (i.e. citizen petitions and TEAs). However, as described in Part 314, an NDA (1) generally requires a fee, (2) is approved only for a specific product (including formulation and labelling), (3) has reporting requirements subsequent to approval, and (4) requires prior approval for most subsequent labelling and formulation changes to the product.

LICENSING REQUIREMENTS

Requirements related to safety, effectiveness, and labelling for drugs to be included in an OTC drug monograph are set forth in 21 CFR Part 330.

An NDA must contain substantial evidence of effectiveness derived from adequate and well-controlled clinical studies, evidence of safety, and adequate chemistry, manufacturing and control (CMC) information. The format of an NDA submission and the requirements for its various sections are set forth in Part 314 and discussed in several CDER guidance documents[11]. The guidance for industry on botanical drug products provides specific recommendations tailored to this category of drug products[12].

QUALITY

Given their specific characteristics, the CMC documentation that should be provided for botanical drugs will often differ from that of synthetic or highly purified drugs, whose active constituents can be more readily chemically identified and quantified.

Because of the complex nature of a typical botanical drug and the lack of knowledge of its active constituent(s), FDA may rely on a combination of tests and controls to ensure the identity, purity, quality, strength, potency, and consistency of botanical drugs. These tests and controls include (1) multiple tests for drug substance and drug product (e.g. spectroscopic and/or chromatographic fingerprints, chemical assay of characteristic markers, and biological assay), (2) raw material and process controls (e.g. strict quality controls for the botanical raw materials and adequate in-process controls), and (3) process validation (especially for the drug substance).

Starting materials of botanical origin that are used to produce a botanical drug substance should also be evaluated for quality. The use of appropriate starting materials and the drug substance manufacturer's ability to control the source depend on appropriate specifications (tests, analytical procedures, and acceptance criteria). In addition to establishing specifications, manufacturers are encouraged to apply the principles outlined in FDA's botanical guidance and by following good agricultural and good collection practice for starting materials of herbal origin (e.g. European Medicines Agency *GACP guidance – ref. EMEA/HMPC/246816/2005*) in order to achieve adequate quality control of starting materials. Upon receipt of the starting materials at a processing facility, it is the responsibility of the drug substance manufacturer to determine the suitability of these raw materials before use. This can be accomplished by examining and/or testing to ensure that the acceptance criteria are met and by documenting the quality control for the processing of the starting materials.

Pharmacopoeial chapters

The USP-NF is a combination of two official compendia, the United States Pharmacopeia (USP) and the National Formulary (NF). USP includes quality monographs for botanicals in two sections: in the drugs section, for which compliance with USP monographs is mandatory; in the dietary supplements section, where compliance is voluntary. NF includes botanical monographs that are considered excipients. Currently, USP has quality monographs for about 60 botanical drug monographs and about 112 botanical dietary supplement monographs. USP monographs include the name of the ingredient or preparation, the definition, identification tests, packaging, storage, and labelling requirements, limits for contaminants (such as aflatoxins, elemental impurities, microbial limits, pesticides, and organic solvents) and the specifications for content of active or marker principles. The specification consists of a series of tests, procedures for the tests, and acceptance criteria. These tests and procedures require the use of official USP Reference Standards. The botanical ingredients and products

will have the stipulated strength, quality, and purity if they conform to the requirements of the monograph and relevant USP General Chapters. Tests and procedures referred to in multiple monographs are described in detail in the USP-NF General Chapters.

GMP

FDA regulations on current good manufacturing practices (CGMPs) apply to all OTC drug monograph products, including any listed botanical drug products (see § 330.1(a)).

The manufacturing, processing, and controls (receipt, identification, storage, handling, sampling, testing, and approval or rejection of components, drug products, and container closures) for botanical drug products must be in conformance with CGMP as set forth in 21 CFR Parts 210 and 211. In addition, the manufacturing, processing, and controls for the botanical drug substance (starting from the botanical raw material) should be in conformance with CGMP because these elements can affect the quality, safety, and efficacy of the drug product. A satisfactory inspection is necessary for NDA approval.

SAFETY & EFFICACY

The standards for the safety and efficacy required for marketing approval of a botanical drug are the same as those required for a conventional chemical drug for the same indication.

If available information is insufficient to support an NDA for a botanical drug, the sponsor will need to develop further data. An IND is required under section 505(i) of the Act and 21 CFR Part 312 (unless exempt under § 312.2(b)) when a botanical product is studied in the United States for a drug use (see section 201(g) of the Act), even if such study is intended solely for research purposes. Under § 312.22, an IND must contain sufficient information to demonstrate that the drug product is safe for testing in humans and that the clinical protocol is properly designed for its intended objectives. Guidance on data to be provided is given in the Guidance document for botanical drug products.

FEES[13]

The *Prescription Drug User Fee Act* (PDUFA)[14], enacted in 1992 and renewed in 1997 (PDUFA II) and 2002 (PDUFA III) authorises FDA to collect fees from companies that produce certain human drug and biological products. PDUFA established three types of user fees - application fees, establishment fees, and product fees. Since the passage of PDUFA, user fees have played an important role in expediting the drug approval process.

Application fee means approval of a new drug (NDA, ANDA, and certain Biological License Applications (BLAs)).
Prescription drug **product fees** are assessed annually on products for each person who is the applicant in a human drug application or had a human drug application or supplement pending after September 1, 1992. A number of exceptions apply nonetheless.

- Application
  - Requiring clinical data $1,405,500
  - Not requiring clinical data $ 702,750
  - Supplements requiring clinical data $ 702,750
- Products $ 79,720

There are **no fees** for medicinal products marketed on the basis of OTC monographs as there is no pre-marketing review by the FDA.

EVALUATION

No evaluation takes place if the botanical drug product follows an existing monograph.

In case of an NDA, the appropriate therapeutic review division within CDER will take the lead of the evaluation of the drug product.

The CDER Botanical Review Team (BRT) will participate in all phases of review, meetings and decision-making processes as well as for all botanical pre-Investigational New Drug (IND) applications and INDs. The involvement of the BRT in the review process is explained in details in the Manual of Policies and Procedure (MaPP) 6007.1 Review of Botanical Drug Products[15].

Product Information

The final rule on labelling requirements for OTC medicines of April 16, 1999[16] established a standardised format and standardised content requirements for the labelling of OTC drug products. The rule is intended to assist consumers in reading and understanding OTC drug product labelling.

All OTC drug products are required to carry the new, easy-to-read format and the revised content.

The rule requires the following labelling outline:

- the heading "Drug Facts"
- active ingredient(s) (in each dosage unit)
- purpose
- uses
- warnings – do not use ..., ask a doctor before use if you have ..., ask a doctor or pharmacist before use if you are ..., when using this product ..., stop use and ask a doctor if ...
- a warning to ask a health professional before use if pregnant or breast-feeding
- warnings to keep out of reach of children and to get medical help or contact a Poison Control Center right away in case of overdose
- directions
- other information
- inactive ingredients
- space for companies to voluntarily ask for questions, with a telephone number.

The rule spells out very detailed format requirements.

Ordinarily, leaflets (usually called 'inserts' in the U.S.) are not required, although some products subject to new drug applications include a required insert, and other products choose to include an insert.

For (botanical) prescription drugs, the term "labelling" is generally defined by section 321(m) of the U.S. Code as "all labels and other written, printed, or graphic matter (1) upon any article or any of its containers or wrappers, or (2) accompanying such article."
The requirements for prescription drug labelling as defined in the Federal Regulations (21 CFR 201.56) are as follows:

- Contain a summary of essential scientific information for the safe and effective use of the drug
- Be informative and accurate
- Use language that is not promotional in tone, false, or misleading
- Not make claims or suggest uses for drugs when there is not sufficient evidence of safety and a substantial evidence of effectiveness
- Contain information based whenever possible on data derived from human experience

On January 24, 2006, the *"Final Rule: Requirements on the Content and Format of Labeling for Human Prescription Drug and Biological Products"*[17] was issued. They are also explicated in a guidance document for industry.[18]
Changes include:

- A "Highlights" section which provides the overview of a drug's benefits and risks for healthcare professionals. A Contents section to serve as a navigational tool,
- Reordered and reorganised frequently referenced sections,
- Format changes that make the labelling easier to read, and
- Consolidated safety information.

Trade Name

In general, there are no special rules for the use of trademarks for botanical medicines beyond those that apply to product trademarks as a whole. However, a product name evaluation is included as a part of the labelling review for new drug applications. FDA pays special attention to names which may be a source of name-related medication errors.

Advertising

The advertising of non-prescription botanical medicines and dietary supplements in the U.S. falls under the jurisdiction of the Federal Trade Commission (FTC)[19] – a government agency whose responsibilities include monitoring advertising and acting on deceptive or unfair practices.

FTC's authority translates into three basic regulatory standards that apply to all consumer product advertising:

PRODUCT INFORMATION

The final rule on labelling requirements for OTC medicines of April 16, 1999[16] established a standardised format and standardised content requirements for the labelling of OTC drug products. The rule is intended to assist consumers in reading and understanding OTC drug product labelling.

All OTC drug products are required to carry the new, easy-to-read format and the revised content.

The rule requires the following labelling outline:

- the heading "Drug Facts"
- active ingredient(s) (in each dosage unit)
- purpose
- uses
- warnings – do not use ..., ask a doctor before use if you have ..., ask a doctor or pharmacist before use if you are ..., when using this product ..., stop use and ask a doctor if ...
- a warning to ask a health professional before use if pregnant or breast-feeding
- warnings to keep out of reach of children and to get medical help or contact a Poison Control Center right away in case of overdose
- directions
- other information
- inactive ingredients
- space for companies to voluntarily ask for questions, with a telephone number.

The rule spells out very detailed format requirements.

Ordinarily, leaflets (usually called 'inserts' in the U.S.) are not required, although some products subject to new drug applications include a required insert, and other products choose to include an insert.

For (botanical) prescription drugs, the term "labelling" is generally defined by section 321(m) of the U.S. Code as "all labels and other written, printed, or graphic matter (1) upon any article or any of its containers or wrappers, or (2) accompanying such article."
The requirements for prescription drug labelling as defined in the Federal Regulations (21 CFR 201.56) are as follows:

- Contain a summary of essential scientific information for the safe and effective use of the drug
- Be informative and accurate
- Use language that is not promotional in tone, false, or misleading
- Not make claims or suggest uses for drugs when there is not sufficient evidence of safety and a substantial evidence of effectiveness
- Contain information based whenever possible on data derived from human experience

On January 24, 2006, the *"Final Rule: Requirements on the Content and Format of Labeling for Human Prescription Drug and Biological Products"*[17] was issued. They are also explicated in a guidance document for industry.[18]
Changes include:

- A "Highlights" section which provides the overview of a drug's benefits and risks for healthcare professionals. A Contents section to serve as a navigational tool,
- Reordered and reorganised frequently referenced sections,
- Format changes that make the labelling easier to read, and
- Consolidated safety information.

TRADE NAME

In general, there are no special rules for the use of trademarks for botanical medicines beyond those that apply to product trademarks as a whole. However, a product name evaluation is included as a part of the labelling review for new drug applications. FDA pays special attention to names which may be a source of name-related medication errors.

ADVERTISING

The advertising of non-prescription botanical medicines and dietary supplements in the U.S. falls under the jurisdiction of the Federal Trade Commission (FTC)[19] – a government agency whose responsibilities include monitoring advertising and acting on deceptive or unfair practices.

FTC's authority translates into three basic regulatory standards that apply to all consumer product advertising:

- Advertising claims must be substantiated – that is, the advertiser must have a "reasonable basis" for believing the claim is true before the ad runs. As a general rule, a claim must be supported by the level of substantiation it communicates to the consumer.
- Advertisements may not be deceptive. Deceptive conduct is a material representation, omission, or practice likely to mislead a consumer acting reasonably under the circumstances.
- Advertisements may not be unfair. This is a more subjective standard and, as such, has been difficult to apply. Unfairness is defined as an act or practice that is likely to cause substantial injury to consumers and that is neither reasonably avoidable by consumers nor outweighed by benefits to consumers or business.

In addition to the FTC Act[20] at the federal level, many - if not most - individual states have their own version of the FTC Act, which allows state consumer protection authorities to act against deceptive or unfair advertising.

OTC and dietary supplement advertising to the public is allowed in all media. The ads do not have mandatory text. Comparative advertising is allowed and, indeed, encourages competition that provides consumers with information about products. OTC and dietary supplement advertising is not subject to preclearance by FTC. Rather, FTC exercises post-publication control.

In addition to government post-publication oversight of advertising, there are a number of self-regulatory advertising systems. The major broadcast television networks maintain their own clearance departments, which must be satisfied with an ad before the network will accept it. Networks particularly look for documentation to support claims.

The National Advertising Division (NAD) of the Council of Better Business Bureau[21] investigates complaints based on the truth and accuracy of an advertisement. The ad in question goes through a multi-step procedure including the advertiser, and concludes with a final case decision that is published in a case reporter. The decision could report that the ad was substantiated, modified, or discontinued; or that the case was referred to a government agency.

The Consumer Healthcare Products Association (CHPA) has a *Code of Advertising Practices*[22] which includes a series of points to help guide OTC medicine advertisers and it is typically integrated by individual companies in their clearance procedures. The code includes a provision asking that ads urge viewers to read and follow label directions. The code also includes a reference to the NAD.

The advertising of prescription medicines falls under the jurisdiction of FDA. Since 1997, direct to consumer (DTC) ads have been permitted. In 1999, the FDA finalised guidance to allow the advertiser to include a "major statement" relating to side effects and contraindications and if they include "adequate provision" to get approved package labelling to consumers. "Adequate provision" tools can include a toll-free telephone number for consumers to call for the labelling, reference to print ads running at the same time that include the information, distributing information through a range of accessible locations, or a website address with the information.

Under the *FDA Amendments Act* of 2007[23], the FDA now has the authority to require the submission of prescription DTC ads prior to dissemination of the ad, and to require specific disclosures in prescription DTC ads. The 2007 law also clarifies that the "major statement" must be presented in a "clear, conspicuous, and neutral manner."

Distribution

Under Federal law, non-prescription medicines and dietary supplements may be sold in any and all retail outlets in the U.S. – pharmacies, mass merchandisers, food stores, or others.

Mail order sales, Internet sales, and teleshopping of OTC medicines and dietary supplements are permitted in the U.S.

Other information

Taxes

State sales taxes vary. Roughly a third of the states exempt medicines, including OTCs, from their state sales tax.

LINKS

- US Department of Health and Human Services: http://www.hhs.gov/
- Food and Drug Administration (FDA): http://www.fda.gov
- Federal legislation and regulations: http://www.gpoaccess.gov/
- Code of Federal Regulations Title 21: http://www.accessdata.fda.gov/scripts/cdrh/cfdocs/cfCFR/CFRSearch.cfm
- US Pharmacopeia (USP): http://www.usp.org/
- National institute of health – national center for complementary and alternative medicines: http://nccam.nih.gov/

WSMI MEMBER ASSOCIATION

Consumer Healthcare Products Association (CHPA)
900 19th Street, N.W.
Washington, D.C. 20006, USA
Phone: +1 202 / 429 9260
Fax: +1 202 / 223 6835
E-mail: info@chpa-info.org
Website: www.chpa-info.org

ACKNOWLEDGMENTS

Jinhui Dou, Botanical Review Team, US Food and Drug Administration, who served as expert resource on botanical review in the US
Nandakumara D. Sarma, USP, who helped us on the pharmacopoeial section
Freddie Ann Hoffman, Heterogeneity, for her help on the fees

REFERENCES

1 Federal Food, Drug, and Cosmetic Act (FD&C Act)
http://www.fda.gov/RegulatoryInformation/Legislation/FederalFoodDrugandCosmeticActFDCAct/default.htm

2 U.S. Food and Drug Administration
http://www.fda.gov

3 Food and Drug Administration Amendments Act (FDAAA) of 2007
http://www.fda.gov/RegulatoryInformation/Legislation/FederalFoodDrugandCosmeticActFDCAct/SignificantAmendmentstotheFDCAct/FoodandDrugAdministrationAmendmentsActof2007/default.htm

4 Title 21 of the Code of Federal Regulations (CFR)
http://www.accessdata.fda.gov/scripts/cdrh/cfdocs/cfcfr/cfrsearch.cfm

5 Chen et al . New therapies from old medicines. Nat Biotechnol 2008; 26: 1077-1083
http://www.nature.com/nbt/journal/v26/n10/full/nbt1008-1077.html

6 OTC ingredient list (including botanical ingredients), by monograph category
http://www.fda.gov/downloads/AboutFDA/CentersOffices/CDER/UCM135688.pdf

7 21 CFR Part 330 – Over-the-Counter (OTC) Human Drugs which are generally recognized as safe and effective and not misbranded
http://www.access.gpo.gov/nara/cfr/waisidx_09/21cfr330_09.html

8 21 CFR 10.30 – Citizen petition
http://www.accessdata.fda.gov/scripts/cdrh/cfdocs/cfCFR/CFRSearch.cfm?fr=10.30

9 Final rule – "additional criteria and procedures for classifying over-the-counter drugs as generally recognized safe and effective and not misbranded"
http://frwebgate.access.gpo.gov/cgi-bin/getdoc.cgi?dbname=2002_register&docid=fr23ja02-7.pdf

10 Draft guidance for industry – time and extent applications
http://www.fda.gov/downloads/Drugs/GuidanceComplianceRegulatoryInformation/Guidances/ucm078902.pdf

[11] NDA Guidance documents
http://www.fda.gov/Drugs/DevelopmentApprovalProcess/HowDrugsareDevelopedandApproved/ApprovalApplications/NewDrugApplicationNDA/default.htm

[12] Guidance for industry – Botanical Drug products
http://www.fda.gov/downloads/AboutFDA/CentersOffices/CDER/UCM106136.pdf

[13] PDUFA 2010
http://www.regulations.gov/search/Regs/contentStreamer?objectId=09000064809ffea1&disposition=attachment&contentType=html

[14] Prescription Drug User Fee Act (PDUFA)
http://www.fda.gov/ForIndustry/UserFees/PrescriptionDrugUserFee/default.htm

[15] MaPP – review of botanical drug products
http://www.fda.gov/downloads/AboutFDA/CentersOffices/CDER/ucm106137.pdf

[16] Over-the-counter human drugs; labelling requirements; Final Rule
http://frwebgate.access.gpo.gov/cgi-bin/getdoc.cgi?dbname=1999_register&docid=99-6296-filed.pdf

[17] Requirements on Content and Format of Labeling for Human Prescription Drug and Biological Products and Draft Guidances and Two Guidances for Industry on the Content and Format of Labeling for Human Prescription Drug and Biological Products; Final Rule and Notices
http://www.fda.gov/OHRMS/DOCKETS/98fr/06-545.pdf

[18] Guidance for industry – Labeling for human prescription drug and biological products – implementing the new content and format requirements
http://www.fda.gov/downloads/Drugs/GuidanceComplianceRegulatoryInformation/Guidances/ucm075082.pdf

[19] Federal Trade Commission
http://www.ftc.gov/

[20] Federal Trade Commission Act
http://www.ftc.gov/ogc/FTC_Act_IncorporatingUS_SAFE_WEB_Act.pdf

[21] Better Business Bureau
http://www.bbb.org/us/

[22] CHPA code of practice
http://www.chpa-info.org/scienceregulatory/Voluntary_Codes.aspx#AdvertisingPractices

[23] FDA Amendments Act section 901(d)(3)(A), amending the Food, Drug, and Cosmetic Act section 502(n)
http://www.fda.gov/oc/advisory/FDAAATitle_VII.htm
