# Supplementary material for: Two Sides of the Same Coin for Health: Adaptogenic Botanicals as Nutraceuticals for Nutrition and Pharmaceuticals in Medicine
Source: Pharmaceuticals (Basel). 2025 Sep 8;18(9):1346. doi: 10.3390/ph18091346 (PMC12472958; doi:10.3390/ph18091346)
Supplement: Supplementary file 1 [file pharmaceuticals-18-01346-s001.zip › Supplement S6_Chenese Pharmacopoe 2010 Ginseng Eleutherococcus Schisandra R.crenulata Andro.pdf]

PHARMACOPOEIA
OF THE PEOPLE'S REPUBLIC OF CHINA

(2010)

Volume I

Chinese Pharmacopoeia Commission

China Medical Science Press

summer and autumn, washed clean, the bark is stripped off, and dried in the sun.

Description Irregular quills, 5-15 cm long, 0.4-1.4 cm in diameter, about 2 mm thick. Outer surface greyish-brown, with slightly twisted longitudinal wrinkles and transverse lenticel-like scars; inner surface pale yellow or greyish-yellow, with fine longitudinal striations. Texture light, fragile, easily broken, fracture irregular, greyish-white. Odour, slightly aromatic; taste, slightly pungent and bitter.

Identification Transverse section: Cork cells several layers. Phellem narrow, scattered with a few secretory canals. Phloem broad, with clefts in the outer part, rays 1-5 cells wide; secretory canals fairly frequent, surrounded with 4-11 secretory cells. Parenchymatous cells containing clusters of calcium oxalate and small starch granules.

Powder: Greyish-white. Clusters of calcium oxalate 8-64 μm in diameter, sometimes the crystal cells linked together, with clusters arranged in rows. Cork cells rectangular or polygonal, thin-walled; sometimes the walls of cork cells of older root barks unevenly thickened, less pitted. Fragments of secretory canals containing colourless or pale yellow secretion. Starch granules abundant, simple granules polygonal or subspherical, 2-8 μm in diameter; compound granules consisting of 2 to tens of components.

Water Not more than 13.0 per cent (Appendix Ⅸ H, method 1).

Total ash Not more than 12.0 per cent (Appendix Ⅸ K).

Prepared slices

Processing Eliminate foreign matter, wash clean, soften thoroughly, cut into thick slices, and dry in the sun.

Property and Flavor Warm; pungent and bitter.

Meridian tropism Liver and kidney meridians.

Actions To dispel wind and remove dampness, tonify and replenish the liver and kidney, strengthen sinew and bone, promote urination to alleviate edema.

Indications Wind-dampness impediment disease, limp wilting sinew and bone, infantile walk retardation, weak constitution and lack of strength, edema, tinea pedis.

Administration and dosage 5-10 g.

Storage Preserve in a dry place, protected from mould and moth.

Acanthopanacis Senticosi Radix et Rhizoma seu Caulis (刺五加, Ciwujia)

Manyprickle Acanthopanax

Manyprickle Acanthopanax is the dried root and rhizome or stem of *Acanthopanax senticosus* (Rupr. et Maxim.) Harms (Fam. Araliaceae). The drug is collected in spring or autumn, washed clean, and dried.

Description Rhizomes irregular, nodular cylindrical, 1.4-4.2 cm in diameter. Root cylindrical, mostly tortuous, 3.5-12 cm long, 0.3-1.5 cm in diameter. Externally greyish-brown or blackish-brown, rough, with fine longitudinal furrows and wrinkles, bark relatively thin, sometimes exfoliated, the exposed surface appearing greyish-yellow. Texture hard, fracture yellowish-white, fibrous. Odour, characteristic and aromatic; taste, slightly pungent, somewhat bitter and astringent.

Stems long cylindrical, much-branched, varying in length, 0.5-2 cm in diameter. Externally pale grey, the old branches greyish-brown, furrowed longitudinally, spineless; the young branches yellowish-brown, densely spiculate. Texture hard, uneasily broken, fracture showing thin bark, yellowish-white, wood being broad, pale yellow, and pith at the centre. Odour, slight; taste slight pungent.

Identification (1) Transverse section of root: Cork cells of 10 or more layers. Phelloderm thin, scattered with secretory canals; most of parenchymatous cells containing clusters of calcium oxalate, 11-64 μm in diameter. The outside of phloem scattered with more fibre bundles, which lessen inwards; secretory canals subrounded or elliptical, 25-51 μm long radially, 48-97 μm long tangentially; parenchymatous cells containing clusters. Cambium in a ring. The majority of root occupied by xylem, rays 1-3 rows of cells wide; vessels relatively thin-walled, mostly several grouped; xylem fibres well developed.

Transverse section of rhizome: Fibre bundles in phloem much more than those in root; pith visible.

Transverse section of stem: Pith well developed.

(2) To 5 g of the powder add 50 ml of 75% ethanol, heat under reflux for 1 hour, filter and evaporate the filtrate to dryness, dissolve the residue with 10 ml of water, extract by shaking with two 5-ml quantities of chloroform, combine the chloroform solutions, evaporate to dryness, dissolve the residue in 1 ml of methanol as the test solution. Prepare a solution of 5 g of Acanthopanacis Senticosi Radix et Rhizoma seu Caulis reference drug in the same manner as the reference drug solution. Dissovle isofraxidin CRS in methanol to produce a solution containing 1 mg per ml as the reference solution. Carry out the method for thin layer chromatography (Appendix Ⅵ B), using silica gel G as the coating substance and a mixture of chloroform and methanol (19 : 1) as the mobile phase. Apply separately 10 μl of each of the above three solutions to the plate. After developing and removal of the plate, dry in air, examine under ultraviolet light at 365 nm. The fluorescent spots in the chromatogram obtained with the test solution correspond in position and colour to the fluorescent spots in the chromatogram obtained with reference drug solution. A blue fluorescent spot in the chromatogram obtained with the test solution corresponds in position and colour to the fluorescent spot in the chromatogram obtained with the reference solution.

Water Not more than 10.0 per cent (Appendix Ⅸ H, method 1).

Total ash Not more than 9.0 per cent (Appendix Ⅸ K).

Extractives Carry out the method for determination of ethanol-soluble extractives (Appendix Ⅹ A, the hot extraction method), using methanol as the solvent, not less than 3.0 per cent.

Assay Carry out the method for high performance liquid chromatography (Appendix Ⅵ D).

Chromatographic system and system suitability Use octadecylsilane bonded silica gel as the stationary phase and a mixture of methanol and water (20 : 80) as the mobile phase. As detector a spectrophotometer set at 265 nm. The number of theoretical plates of the column is not less than 2000, calculated with reference to the peak of syringoside.

Reference solution Weigh accurately a quantity of syringoside CRS, dissolve in methanol to produce a solution containing 80 μg per ml.

Test solution Weigh accurately 2 g of the coarse powder to a stopper conical flask, add accurately 25 ml of methanol, and weigh. Ultrasonicate (power 250 W, frequency 33 kHz) for 30 minutes, cool and weigh again, replenish the loss of solvent with methanol and mix well, filter and use the successive filtrate as the test solution.

Procedure Inject accurately 10 μl of each of the reference solution and the test solution into the column, respectively, and calculate the content.

It contains not less than 0.050 per cent of syringoside ($C_{17}H_{24}O_9$), calculated with reference to the dried drug.

Prepared slices

Processing Remove from foreign matters, wash clean, soak briefly and soften thoroughly, cut into thick slices and dry.

In subrounded or irregular thick slices. Root and rhizome externally greyish-brown or blackish-brown, rough, with longitudinal furrows and wrinkles; bark relatively thin, sometimes exfoliated, the exposed parts appearing greyish-yellow; stem externally pale grey or greyish-brown, spineless, twig yellowish-brown and with dense spinlets. Cut surface yellowish-white, fibrous; stem with a thin bark and a broad wood, medullated. Odour of root and rhizome characteristic and aromatic; taste slightly pungent, somewhat bitter and astringent. Odour of stem slight; taste slightly pungent.

Identification As required for the crude drug except for the transverse section.

Water Not more than 8.0 per cent, following the method for the crude drug.

Total ash Not more than 7.0 per cent, following the method for the crude drug.

Extractives As required for the crude drug.

Assay As required for the crude drug.

Property and Flavor Warm; pungent and mild bitter.

Meridian tropism Spleen, kidney and heart meridians.

Actions To tonify qi and fortify the spleen, tonify the kidney and tranquilize the mind.

Indications Spleen-lung qi deficiency, weak constitution and lack of strength, anepithymia, dual deficiency of lung and kidney, chronic cough and dyspnea of deficiency type, limp aching in the lower back and knees caused by kidney deficiency, deficiency of heart and spleen, insomnia and dream-distrubed sleep.

Administration and dosage 9-27 g.

Storage Preserve in a ventilated and dry place, protected from moisture.

Achilleae Herba
(蓍草, Shicao)

Alpine Yarrow Herb

Alpine Yarrow Herb is the dried aerial part of *Achillea alpina* L. (Fam. Compositae). The drug is collected at flowering in summer and autumn, removed from foreign matter, and dried in the shade.

Description Stems cylindrical, 1-5 mm in diameter. Externally yellowish-green or yellowish-brown, longitudinally angustate, covered with white pubescences; texture fragile, easily broken, fracture white, medullated or hollowed in the centre. Leaves frequently crumpled, broken, when whole, linear-lanceolate, segments linear, externally greyish-green to yellowish-brown, pubescent on both surfaces. Capitula gathered to compound corymbose, yellowish-brown; involucres ovate or oblong, imbricated. Odour, slightly aromatic; taste, slightly bitter.

Identification (1) Powder: Greyish-green. Non-glandular hairs numerous, mostly 5-celled, apical cell slender, long whip-shaped. Stomata anomocytic, with 3-5 subsidiary cells. Pollen grains subrounded, 20-40 μm in diameter, exine with fine spinate prominences, bearing 3 germinal apertures. Fibres in bundles or scattered, mostly broken, with thick walls and distinct pit-canals.

(2) To 1 g of the powder add 20 ml of petroleum ether (60-90℃), ultrasonicate for 10 minutes, discard the petroleum ether solution, and expel the residue to dryness, add 1 ml of dilute hydrochloric acid and 50 ml of ethyl acetate, ultrasonicate for 30 minutes, filter, evaporate the filtrate to dryness, and dissolve the residue in 2 ml of methanol as the test solution. Prepare a solution with 1 g of Achilleae Herba reference drug in the same manner as the reference drug solution. Dissolve chlorogenic acid CRS in methanol to prepare a solution containing 1 mg per ml as the reference solution. Carry out the method for thin layer chromatography (Appendix Ⅵ B), using polyamide as the coating substance and the upper layer of the mixture of toluene, ethyl acetate, formic acid, acetic acid and water (1 : 15 : 1.5 : 1.5 : 2) as the mobile phase. Apply separately to the plate 2 μl of each of the test solution and the reference drug solution and 1 μl of the reference solution. After developing and removal of the plate, dry in air and examine under ultraviolet light at 365 nm. The fluorescent spots in the chromatogram obtained with the test solution correspond in position and colour to the spots in the chromatograms obtained with the reference drug solution.

The fluorescent spot in the chromatogram obtained with the test solution corresponds in position and colour to the spot in the chromatogram obtained with the reference solution.

Water Not more than 10.0 per cent (Appendix Ⅸ H, method 1).

Total ash Not more than 7.0 per cent (Appendix Ⅸ K).

Acid-insoluble ash Not more than 2.0 per cent (Appendix Ⅸ K).

Extractives Carry out the method for determination of ethanol-soluble extractives (Appendix Ⅹ A, the hot extraction method), using ethanol as the solvent, not less than 8.0 per cent.

Assay Carry out the method for high performance liquid chromatography (Appendix Ⅵ D).

Chromatographic system and system suitability Use octadecylsilane bonded silica gel as the stationary phase and a mixture of acetonitrile and 0.4% phosphoric acid (11 : 89) as the mobile phase. As detector a spectrophotometer set at 327 nm. The number of theoretical plates of the column is not less than 6000, calculated with reference to the peak of chlorogenic acid.

Reference solution Dissolve a quantity of chlorogenic acid CRS, accurately weighed, in 50% methanol in an amber volumetric flask to prepare a solution containing 40 μg per ml.

longitudinal segments or oblique slices, and dried in the sun.

Description Longiluminal segments oblong or nearly fusiform, 4-10 cm long, 1-2 cm in diameter; edges usually rolled inwards, a raised ridge occurring at the centre. The outer bark reddish-brown, with longitudinal wrinkles, transverse fine striations and transverse elongated lenticels, easily detachable in flakes, the exposed layer reddish-brown. Oblique slices ovate, 2.5-5 cm long, 2-3 cm wide, cut surface whitish or pale reddish-brown, exhibiting radial lines, edges relatively thick, slightly raised upward or slightly curved. Texture light, hard and fragile, easily broken, dusting on breaking. Odour, slight; taste, sweetish.

Identification (1) Powder: Pale reddish-brown. Starch granules single, oblong, long-ovoid, reniform or irregular, 3-13 μm in diameter, hilum indistinct; compound granules less. Needles of calcium oxalate 86-169 μm long, scattered or grouped into bundles in mucilage cells. Clusters of calcium oxalate 25-78 μm in diameter, with broad and large angles. Bordered pitted vessels 35-60 μm in diameter.
(2) To 2 g of the powder add 30 ml of ethanol, heat under reflux for 1 hour, filter, and evaporate to dryness. Dissolve the residue in 2 ml of ethanol as the test solution. Prepare a solution of 2 g of Ampelopsis Radix reference drug in the same manner as the reference drug solution. Carry out the method for thin layer chromatography (Appendix Ⅵ B), using silica gel G as the coating substance and a mixture of chloroform and methanol (6 : 1) as the mobile phase. Apply separately to the plate 5 μl of each of the above two solutions. After developing and removal of the plate, dry in air. Spray with a 10% solution of sulfuric acid in ethanol and heat at 105℃ to the spots clear. The spots in the chromatogram obtained with the test solution correspond in position and colour to the spots in the chromatogram obtained with the reference drug solution.

Foreign matter Not more than 3.0 per cent (Appendix Ⅸ A).

Water Not more than 15.0 per cent (Appendix Ⅸ H, method I).

Total ash Not more than 12.0 per cent (Appendix Ⅸ K).

Acid-insoluble ash Not more than 3.0 per cent (Appendix Ⅸ K).

Extractives Carry out the method for determination of ethanol-soluble extractives (Appendix Ⅹ A, the cold extraction method), using 25% ethanol as the solvent, not less than 18.0 per cent.

Prepared slices

Processing Eliminate foreign matter, wash clean, soften thoroughly, cut into thick slices, and dry.

Property and Flavor Mild cold; bitter.

Meridian tropism Heart and stomach meridians.

Actions To clear heat and remove toxin, disperse abscesses and dissipate binds, promote wound healing and promote tissue regeneration.

Indications Abscess and cellulitis, carbuncle of the back, deep-rooted boil and sore, scrofula, burns and scald.

Administration and dosage 5-10g. Appropriate amount for topical application, decocted for bathing, or ground into extreme fine powder for applying to pars affecta.

Precautions and Warnings Incompatible with Aconiti Radix, Aconiti Radix cocta, Aconiti Kusnezoffii Radix, Aconiti Kusnezoffii Radix cocta, Aconiti Lateralis Radix.

Storage Preserve in a ventilated and dry place, protected from moth.

Andrographis Herba (穿心莲, Chuanxinlian)

Common Andrographis Herb

Common Andrographis Herb is the dried aerial part of *Andrographis paniculata* (Burm. f.) Nees (Fam. Acanthaceae). The drug is collected in early autumn when foliage branch growing luxuriantly, and dried in the sun.

Description Stems square and frequently branched, 50-70 cm long, nodes slightly swollen; texture fragile, easily broken. Leaves simple, opposite, short petioled or nearly sessile; lamina crumpled and easily broken, when whole, lanceolate or ovate-lanceolate, 3-12 cm long, 2-5 cm wide, with acuminate apex and cuneate-decurrent base, margin entire or undulate; the upper surface green, the lower surface greyish-green, glabrous on both surfaces. Odour, slight; taste, extremely bitter.

Identification (1) Transverse section of leaf: Upper epidermal cells subsquare or rectangular, lower epidermal cells relatively small, both surfaces with crystal cells containing rounded, long-elliptical or clavate cystoliths. Glandular scales and sometimes non-glandular hairs visible. Palisade 1-2 layers of cells, across the upper part of midrib; spongy cells arranged loosely. Vascular bundles of midrib collateral and grooved, crystal cells occurring above the xylem.
Surface view of leaf: Enlarged crystal cells occurring in the upper and lower epidermis, containing large conchoidal cystoliths, up to 36 μm in diameter and 180 μm long, with a hilum-shaped scar in the large end, concentric striations wavy. Stomata more frequent on the lower epidermis, diacytic, sometimes anomocytic, subsidiary cells significantly varying in size. Head of glandular scales oblate, 4,6 (8) -celled, up to 40 μm in diameter, stalk very short. Non-glandular hairs 1-4 celled, up to 160 μm long and 40 μm in diameter, with cuticular striations on the surface.
(2) Use the test solution obtained under the Assay as the test solution. To 0.5 g of Herba Andrographis reference drug add 30 ml of ethanol, ultrasonicate for 30 minutes, filter and concentrate the filtrate to about 5 ml as the reference drug solution. Dissolve dehydroandrographolide CRS and andrographolide CRS in dehydrated ethanol to produce a mixture containing 1 mg of each per ml as the reference solution. Carry out the method for thin layer chromatography (Appendix Ⅵ B), using silica gel GF_{254} as the coating substance and a mixture of chloroform, ethyl acetate and methanol (4 : 3 : 0.4) as the mobile phase. Apply separately to the plate 6 μl of each of the test solution and the reference drug solution and 4 μl of the reference solution. After developing and removal of the plate, dry in air, examine under ultraviolet light at 254 nm. The spots in the chromatogram obtained with the test solution correspond in position and colour to the spots in the chromatograms obtained with the reference drug solution and the reference solution. Spray with a mixture of 2% solution of 3,5-dinitro-benzoic acid in ethanol and 2 mol/L potassium hydroxide (mixed in equal portions before use), examine in daylight immediately. The spots in the chromatogram obtained with the test solution correspond in position and colour to the spots in the chromatograms obtained with the reference drug solution and the reference solution.

Leaves Not less than 30 per cent.

Extractives Carry out the method for determination of ethanol-soluble extractives (Appendix X A, the hot extraction method), using ethanol as the solvent, not less than 8.0 per cent.

Assay Carry out the method for high performance liquid chromatography (Appendix VI D).

Chromatographic system and system suitability Use octadecylsilane bonded silica gel as the stationary phase and a mixture of methanol and water (52 : 48) as the mobile phase. As detector a spectrophotometer set at 225 nm for andrographolide and 254 nm for dehydroandrographolide. The number of theoretical plates of the column is not less than 2000, calculated with reference to the peaks of andrographolide and dehydroandrographolide.

Reference solution Dissolve a quantity of andrographolide CRS and dehydroandrographolide CRS, accurately weighed, in methanol to produce two solutions each containing 0.1 mg per ml.

Test solution Weigh accurately 0.5 g of the powder (through No. 4 sieve), add accurately 25 ml of 40% methanol, weigh, macerate for 1 hour and ultrasonicate (power 250 W, frequency 33 kHz) for 30 minutes, weigh again and compensate the loss of the weight with methanol, mix well and filter. Apply accurately 10 ml of the successive filtrate to a small column (1.5 cm in inner diameter) packed with neutral aluminium oxide (200-300 mesh, 5 g), elute with 15 ml of methanol. Collect the eluate in a 50 ml volumetric flask, dilute with methanol to volume, and mix well.

Procedure Inject accurately 5 μl of each of the test solution and the reference solution, respectively, into the column, and calculate the content.

It contains not less than 0.80 per cent of total amount of andrographolide ($C_{20}H_{30}O_5$) and dehydroandrographolide ($C_{20}H_{28}O_4$), calculated with reference to the dried drug.

Processing Eliminate foreign matter, wash clean, cut into sections and dry.

Irregular sections, stems are rectangular, nodes slight swollen; texture fragile, easily broken; fracture uneven, marrow almost white. Leaves mostly crumpled or broken, when whole, lanceolate or ovate-lanceolate, with acuminate apex and cuneate-decurrent base, margin entire or undulate; upper surface green, the lower surface greyish-green, glabrous on both sides. Odour, slighty; taste, extremely bitter.

Identification As required for the crude drug except for the transverse section of leaf.

Property and Flavor Cold; bitter.

Meridian tropism Heart, lung, large intestine and bladder meridians.

Actions To clear heat and remove toxin, cool the blood, and disperse swelling.

Indications Common cold with fever, swollen sore throat, mouth and tongue sores, whooping cough, cough caused by consumptive disease, diarrhea and dysentery, heat strangury with slow pain, swelling abscess, sore and ulcer, bite wound of insect, worm or snake.

Administration and dosage 6-9g; Appropriate amount for topical application.

Storage Preserve in a dry place.

Anemarrhenae Rhizoma (知母, Zhimu)

Common Anemarrhena Rhizome

Common Anemarrhena Rhizome is the dried rhizome of *Anemarrhena asphodeloides* Bge. (Fam. Liliaceae). The drug is collected in spring or autumn, removed from fibrous roots and soil, dried in the sun, known as "Maozhimu"; or removed from outer tissue, and dried in the sun.

Description Slat-shaped, slightly curved, somewhat compressed, branched occasionally, 3-15 cm long, 0.8-1.5 cm in diameter, with pale yellowish stem and leaf scars. Externally yellowish-brown to brown, the upper part exhibiting a concave groove and closely arranged annular nodes with dense yellowish-brown remains of leaf bases growing upward bilaterally; the lower part raised and somewhat shrivelled, exhibiting depressed or protruding dotted root scars. Texture hard, easily broken, fracture yellowish-white, Odour slight; taste, slightly sweetish, bitterish and viscous on chewing.

Identification (1) To 0.5 g of the powder add 10 ml of dilute ethanol, ultrasonicate for 20 minutes, use the supernatant as the test solution. Dissolve magiferin CRS in dilule ethanol to produce a solution containing 0.5 mg per ml as the reference solution. Carry out the method for thin layer chromatography (Appendix VI B), using polyamide film as the stationary phase and a mixture of ethanol and water (1 : 1) as the mobile phase. Apply separately to the plate 4 μl of each of the above two solutions. After developing and removal of the plate, dry in ajr, examine under ultraviolet light at 365 nm. The spot in the chromatogram obtained with the test solution corresponds in position and colour to the spot in the chromatogram obtained with the reference solution.

(2) To 0.2 g of the powder add 10 ml of 30% acetone, ultrasonicate for 20 minutes, and use the supernatant as the test solution. Dissolve timosaponin B II CRS in 30% acetone to produce a solution containing 1 mg per ml as the reference solution. Carry out the method for thin layer chromatography (Appendix VI B), using silica gel G as the coating substance and the upper layer of a mixture of *n*-butanol, glacial acetic acid and water (4 : 1 : 5) as the mobile phase. Apply separately to the plate 4 μl of each of the above two solutions. After developing and removal of the plate, dry in air, spray with a solution of vanillin in sulfuric acid and heat at 105℃ to the spots clear. The spot in the chromatogram obtained with the test solution corresponds in position and colour to the spot in the chromatogram obtained witll the reference solution.

Water Not more than 12.0 per cent (Appendix IX H, method 1).

Total ash Not more than 9.0 per cent (Appendix IX K).

Acid-insoluble ash Not more than 4.0 per cent (Appendix IX K).

Assay ***Magiferin*** Carry out the method for high performance liquid chromatography (Appendix VI D).

Chromatographic system and system suitability Use octadecylsilane bonded silica gel as the stationary phase and a mixture of acetonitrile and 0.2% glacial acetic acid (15 : 85) as the mobile phase. As detector a spectrophotometer set at 258 nm.

Administration and dosage 3-6 g, decocted with water or soaked in wine or liquor. Appropriate amount for topical application.

Contraindications Contraindicated for pregnant woman.

Storage Preserve in a cool and dry place.

Schisandrae Chinensis Fructus (五味子, Wuweizi)

Chinese Magnoliavine Fruit

Chinese Magnoliavine Fruit is the dried ripe fruit of *Schisandra chinensis* (Turcz.) Barll. (Fam. Magnoliaceae). The drug is known as "Bei Wuweizi" (Northern Magnoliavine Fruit). The drug is collected in autumn when ripe, dried in the sun or after steamed, removed from stalk and foreign matter.

Description Irregularly spheroidal or compressed-spheroidal, 5-8 mm in diameter. Externally red, purplish-red or dark red, shrunken, oily, with soft pulp, sometimes externally blackish-red or covered with "white frost". Seeds 1-2, reniform, externally brownish-yellow, lustrous, testa thin and fragile. Odour of pulp, slight; taste, sour. Odour of seeds, aromatic on crushing; taste pungent and slightly bitter.

Identification (1) Transverse section: Pericarp consisting of 1 layer of square or rectangular epidermal cells, walls relatively thickened, covered with cuticle, oil cells scattered; mesocarp consisting of 10 or more layers of parenchymatous cells containing starch granules, scattered with small collateral vascular bundles; endocarp consisting of 1 layer of small square parenchymatous cells. The most outer layer of testa consisting of radially elongated stone cells, thick-walled, with fine and dense pits and pit-canals; beneath showing several layers of stone cells, subrounded, triangular or polygonal with larger pits; underneath the stone cell layers occurring a few layers of parenchymatous cells, raphe having vascular bundles; oil cell layer consisting of 1 layer of rectangular oil cells containing brownish-yellow oil droplets, with 3-5 layers of small cells lying below; inner epidermal cells of testa in one row, small, slightly thick-walled; endosperm cells containing fatty oil droplets and aleurone grains.

Powder: Dark purple, Stone cells of epidermis of testa polygonal or elongated-polygonal in surface view, 18-50 μm in diameter, walls thickened with very fine and dense pit canals, lumina containing dark brown contents. Stone cells of the inner layers of testa polygonal, subrounded or irregular, up to about 83 μm in diameter, walls slightly thickened, with relatively large pits. Epidermal cells of pericarp polygonal in surface view, anticlinal walls slightly beaded, with cuticle striations, scattered with oil cells. Cells of mesocarp shrivelled, containing dark brown contents and starch granules.

(2) To 1 g of the powder add 20 ml of chloroform, heat under reflux on a water bath for 30 minutes, filter, evaporate the filtrate to dryness. Dissolve the residue in 1 ml of chloroform as the test solution. Produce a solution of 1 g of Schisandrae Chinensis Fructus reference drug in the same manner as the reference drug solution. Dissolve deoxyschizandrin CRS in chloroform to produce a solution containing 1 mg per ml as the reference solution. Carry out the method for thin layer chromatography (Appendix Ⅵ B), using silica gel GF_{254} as the coating substance and the upper layer of petroleum ether (30-60℃), ethyl formate and formic acid (15 : 5 : 1) as the mobile phase. Apply separately 2 μl each of the three solutions to the plate. After developing and removal of the plate, dry in air, examine under ultraviolet light at 254 nm. The spots in the chromatogram obtained with the test solution correspond in position and colour to the spots in the chromatogram obtained with the reference drug solution and the reference solution.

Foreign matter Not more than 1 per cent (Appendix Ⅸ A).

Water Not more than 16.0 percent (Appendix Ⅸ H, method 1).

Total ash Not more than 7.0 percent (Appendix Ⅸ K).

Assay Carry out the method for high performance liquid chromatography (Appendix Ⅵ D).

Chromatographic system and system suitability Use octadecylsilane bonded silica gel as the stationary phase and a mixture of methanol-water (65 : 35) as the mobile phase. As detector a spectrophotometer set at 250 nm. The number of theoretical plates of the column is not less than 2000, calculated with the reference to the peak of schizandrol A.

Reference solution Weigh accurately schisandrin CRS 15 mg in a 50 ml volumetric flask, dissolve and dilute with methanol to volume, mix well and use as the reference solution (containing 0.3 mg of schisandrin per ml).

Test solution Weigh accurately about 0.25 g of the powder (through No. 3 sieve) in a 20 ml volumetric flask, add about 18 ml of methanol, ultrasonicate (power, 250 W; frequency, 20 kHz) for 20 minutes, add methanol to volume, mix well, filter and use the successive filtrate as the test solution.

Procedure Inject accurately 10 μl of each of the reference solution and the test solution into the column, and calculate the content.

It contains not less than 0.40 per cent of schisandrin ($C_{24}H_{32}O_7$).

Prepared slices

Processing *Schisandrae Fructus* Eliminate foreign matter. Break into pieces before use.

Description As required for the crude drug.

Identification As required for the crude drug.

Water and Total ash As required for the crude drug.

Assay Same as required for the crude drug.

Schisandrae Fructus (prscessed with vinegar) Steam the clean Schisandrae Fructus as described under the method for steaming with vinegar (Appendix Ⅱ D) until the drug becomes black in colour. Break into pieces before use.

Similar to Schisandrae Fructus slices in shape, externally black, oily, somewhat lustrous. Odour, vinegar-aromatic.

Extractives Carry out the hot extraction method for determination of ethanol-soluble extractives (Appendix Ⅹ A), using ethanol as solvent, not less than 28.0 per cent.

Identification As required for the crude drug **Identification (2)**.

Water and Total ash As required for the crude drug.

Assay As required for the crude drug.

Property and Flavor Warm; sour and sweet.

Meridian tropism Lung, heart and kidney meridians.

Actions To astringe and secure, tonify qi and engender fluid, tonify the kidney and calm the heart.

Indications Chronic cough, dyspnea of deficiency type, dream emission and spermatorrhea, enuresis and frequent

urination, chronic diarrhea, spontaneous sweating and night sweating, thirst caused by fluid comsumption. interior heat wasting-thirst, palpitation and insomnia.

Administration and dosage 2-6 g.

Storage Preserve in a ventilated and dry place, protected form moud.

Schisandrae Sphenantherae Fructus (南五味子, Nanwuweizi)

Southern Magnoliavine Fruit

Southern Magnoliavine Fruit is the dried ripe fruit of *Schisandra Sphenanthera* Rehd. et Wils. The drug is collected in autumn when ripe, dried in the sun, removed from stalk and foreign matter.

Description Spheroidal or compressed-spheroidal, 4-6 mm in diameter. Externally brownish-red to dark brown, shrunken and shrivelled, pulp usually adhered to seeds closely. Seeds 1-2, kidney-shaped, externally brownish-yellow, lustrous, testa thin and fragile. Odour of pulp, slight; taste, slightly sour.

Identification To 1 g of the powder, add 10 ml of cyclohexane, ultrasonicate for 30 minutes, filter, evaporate the filtrate to dryness. Dissolve the residue in 2 ml of methanol, centrifuge, eveporate the supernatant to dryness, and dissolve the residue in 1 ml of cyclohexane as the test solution. Prepare a solution of 1 g of Schisandrae Sphenantherae Fructus reference drug in the same manner as the reference drug solution. Dissolve anwuligan CRS in cyclohexane to produce a solution containing 2 mg per ml as the reference solution. Carry out the method for thin layer chromatography (Appendix Ⅵ B), using silica gel G as the coating substance and a mixture of chloroform and acetone (60 : 1) as the mobile phase. Apply separately 10 μl of each of the above three solutions to the plate. After developing and removal of the plate, dry in air. Spray with phosphomolybdic acid TS, heat at 110℃ to the spots clear, the dark blue spots in the chromatogram obtained with the test solution correspond in position and colour to the spots in the chromatogram obtained with the reference drug solution; the dark blue spot in the chromatogram obtained with the test solution corresponds in position and colour to the spot in the chromatogram obtained with the reference solution.

Foreign matter Not more than 1 per cent (Appendix Ⅸ A).

Water Not more than 12.0 per cent (Appendix Ⅸ H, method 2).

Total ash Not more than 6.0 per cent (Appendix Ⅸ K)

Assay Carry out the method for high performance liquid chromatography (Appendix Ⅵ D).

Chromatographic system and system suitability Use octadecylsilane bonded silica gel as the stationary phase and a mixture of tetrahydrofuran-water (38 : 62) as the mobile phase. As detector a spectrophotometer set at 254 nm. The number of theoretical plates of the column is not less than 3000, calculated with the reference to the peak of schisantherin A.

Reference solution Dissolve a quantity of schisantherin A CRS, accurately weighed, in methanol to produce a solution containing 40 μg per ml as the reference solution.

Test solution Weigh accurately 0.5 g of the powder (through No. 3 sieve) in a stoppered conical flask, add accurately 50 ml of methanol, weigh ultrasonicate (power, 250 W; frequency, 40 kHz) for 30 minutes, cool and weigh again, replenish the loss of weight with methanol. Mix well, filter, use the successive filtrate as the test solution.

Procedure Inject accurately 20 μl of each of the reference solution and the test solution into the column, and calculate the content.

It contains not less than 0.20 per cent of schisantherin A ($C_{30}H_{32}O_9$), calculated with reference to the dried drug.

Prepared slices

Processing *Schisandrae Sphenantherae Fructus* Eliminate foreign matter. Break into pieces before use.

Description and Identification As required for the crude drug.

Water and Total ash As required for the crude drug.

Assay As required for the crude drug.

Schisandrae Sphenantherae Fructus (processed with vinegar) Steam the clean Schisandrae Sphenantherae Fructus as described under the method for steaming with vinegar (Appendix Ⅱ D) until the drug becomes black in colour. Break into pieces before use.

Similar to schisandrae Chinese Fructus in shape. Externally black, oily, somewhat lustrous. Odour, vinegar-aromatic.

Identification As required for the crude drug

Water As required for the crude drug.

Assay As required for the crude drug.

Property and Flavor Warm; sour and sweet.

Meridian tropism Lung, heart and kidney meridians.

Actions To astringe and secure, tonify qi and engender fluid, tonify the kidney and calm the heart.

Indications Chronic coughand dyspnea of deficiency type, dream emission and spermatorrhea, enuresis and frequent urination, Chronic diarrhea, spontaneous sweating and night sweating, thirst caused by fluid comsumption, interior heat wasting-thirst, palpitation and insomnia.

Administration and dosage 2-6 g.

Storage Preserve in a ventilated and dry place, protected from mould.

Schizonepetae Herba (荆芥, Jingjie)

Fineleaf Schizonepeta Herb

Fineleaf Schizonepeta Herb is the dried aerial part of *Schizonepeta tenuifolia* Briq. (Fam. Labia tae). The drug is collected in summer and autumn when the tops of the flowering stems are in bloom and spikes keep green in colour, removed from foreign matter, and dried in the sun.

Description Stems square, branched at the upper part, 50-80 cm long, 2-4 mm in diameter; externally yellowish-green or pale purplish-red, pubescent; texture light and fragile, fracture almost white. Leaves opposite, mostly fallen off, lamina 3-5 pinnatipartite, the lobes slender. Spike verticillasters terminal, 2-9 cm long, about 7 mm in diameter. Corolla mostly fallen off, persistent calyx campanulate, 5-toothed at the apex, pale brown or

respectively.

Test solution Weigh accurately 0.2 g of the powder to a Soxhlet's extractor, add 30 ml of chloroform, heat under reflux for 1 hours. Discard the chloroform solution, expel chloroform of the residue, add 30 ml of methanol, heat under reflux for 3 hours, evaporate the extract to dryness at a lower temperature, Dissolve the residue in 10 ml of water, extract with two 10 ml quantities of petroleum ether (30-60℃), discard the petroleum ether extracts, apply the water solution to a column packed with D101 macroporous resin (1.5 cm in inner diameter, 15 cm in length), elute with 50 ml of water, discard the water eluate, elute again with 50 ml of 20% ethanol, discard the 20% ethanol eluate, elute successively with 80 ml of 80% ethanol, collect 70 ml of the eluate and evaporate to dryness. Dissolve the residue in methanol and transfer accurately to a 10 ml volumetric flask, add methanol to volume, mix well.

Procedure Inject accurately 10μl of each of the reference solutions and the test solution into the column, respectively, and calculate the content.

It contains not less than 2.25 per cent of the total amount of ginsenoside Rg_1 ($C_{42}H_{72}O_{14}$) and ginsenoside Re ($C_{48}H_{82}O_{18}$).

Property and Flavor Cold; bitter and sweet.

Meridian tropism Lung, stomach meridians.

Actions To tonify qi, replenish lung, dispel summerheat, and engender fluid.

Indications Cough caused by qi deficiency, vexation and restlessness caused by summerheat, thirsty caused by fluid damage, mental confusion and blurred vision, fatigue of limbs.

Administration and dosage 3-9 g.

Precautions and Warnings Incompatible with Veratri Nigri Radix et Rhizoma, Trogopterori Faeces.

Storage Preserve in a cool and dry place, protected from moisture.

Ginseng Radix et Rhizoma (人参, Renshen)

Ginseng

Ginseng is the dried root of *Panax ginseng* C. A. Mey. (Fam. Araliaceae). The drug derived from the cultivated form is known as "Yuanshen" (garden ginseng) and the drug derived from the wild origin is known as "Linxia Shanshen" (Zihai). The drug is collected in autumn and washed clean.

Description Main roots fusiform or cylindrical, 3-15 cm long, 1-2 cm in diameter; externally greyish-yellow, the upper part or entire root exhibiting sparse, shallow, interrupted and coarse transverse-striations and distinct longitudinal wrinkles; the lower part bearing 2-3 branch roots and numerous slender rootlets with inconspicuous minute tubercles. Rhizomes (Lutou) 1-4 cm long, 0.3-1.5 cm in diameter, mostly constricted and curved, bearing adventitious roots (Ding) and showing sparse depressed-circular stem scars (Luwan). Texture relatively hard, fracture yellowish-white, starchy, cambium ring brownish-yellow, bark exhibiting yellow-brown dotted resin canals and radial clefts. Odour, characteristic; taste, slightly bitter and sweet.

Alternatively, main roots as long as or shorter than rhizome, cylindrical, rhomboid or V-shaped, 1-6 cm long; externally greyish-yellow, longitudinally wrinkled, the upper or middle-lower part with annulations, branch roots mostly 2, rootlets less and slender, orderly arranged and showing some distinct warts. Rhizomes slender, a kew stout, the upper part exhibiting sparse or dense deep depressed stem scars, adventitious roots relatively thin, mostly reclinate.

Identification (1) Transverse section: Cork consisting of several rows of cells. Phelloderm narrow. Phloem showing clefts in the outer part, and parenchymatous cells densely arranged and scattered with resin canals containing yellow secretions in the inner part. Cambium in a ring. Xylem rays broad, vessels singly scattered or grouped, interruptedly arranged radially, occasionally accompanied by non-lignified fibres. Parenchymatous cells containing clusters of calcium oxalate.

Powder: Yellowish-white. Fragments of resin canals containing yellow masses of secretion. Clusters of calcium oxalate 20-68 μm in diameter, with acute angles. Cork cells subsquare or polygonal with sinuous walls. Reticulated and scalariform vessels 10-56 μm in diameter. Starch granules fairly abundant, simple granules subspheroidal, semi-circular or irregular poly-gonal, 4-20 μm in diameter, hilum pointed or slit-shaped; compound granules of 2-6 components.

(2) To 1 g of the powder add 40 ml of chloroform, heat under reflux on a water bath for 1 hour, discard the chloroform layer, evaporate the residue to dryness. Moisten the residue with 0.5 ml of water, add 10 ml of *n*-butanol saturated with water, ultrasonicate for 30 minutes. To the supernatant liquid add 3 volumes of ammonia TS, mix well, stand for demixing. Evaporate the supernatant liquid to dryness, dissolve the residue in 1 ml of methanol as the test solution. Prepare a solution of 1 g of Ginseng Radix et Rhizoma reference drug in the same manner as the reference drug solution. Dissolve ginsenosides Rb_1 CRS, Re CRS, Rf CRS and Rg_1 CRS in methanol to produce a mixture containing 2 mg of each per ml as the reference solution. Carry out the method for thin layer chromatography (Appendix Ⅵ B), using silica gel G as the coating substance (500 μm thick) and the lower layer of a mixture of chloroform, ethyl acetate, methanol and water (15 : 40 : 22 : 10), standing below 10℃, as the mobile phase. Apply separately to the plate 1-2 μl of each of the above three solutions. After developing and removal of the plate, dry in air, spray with a 10% solution of sulfuric acid in ethanol, heat at 105℃ to the spots clear. Examine in daylight and under ultraviolet light at 365 nm. The spots or fluorescent spots in the chromatogram obtained with the test solution correspond in position and colour to the spots or fluorescent spots in the chromatogram obtained with the reference drug solution and the reference solution.

Water Not more than 12.0 per cent (Appendix Ⅸ H, method 1).

Total ash Not more than 5.0 per cent (Appendix Ⅸ K).

Acid-insoluble ash Not more than 1.0 per cent (Appendix Ⅸ K).

Assay Carry out the method for high performance liquid chromatography (Appendix Ⅵ D).

Chromatographic system and system suitability Use octadecylsilane bonded silica gel as the stationary phase, acetonitrile as the mobile phase A and water as the mobile

phase B, elute in gradient as the following:
As detector a spectrophotometer set at 203 nm. The number of theoretical plates of the column is not less than 6000, calculated with the reference to the peak of ginsenosideRg_1.

Time (min)	Mobile phase A (per cent V/V)	Mobile phase B (per cent V/V)
0-35	19	81
35-55	19→29	81→71
55-70	29	71
70-100	29→40	71→60

Reference solution Dissolve ginsenosides Rg_1 CRS, Re CRS, Rf CRS and Rb_1 CRS, accurately weighed, in methanol to produce a mixture containing 0.2 mg of each per ml as the reference solution.

Test solution Weigh accurately 1 g of the powder (through No. 4 sieve) to a Soxhlet's extractor, add 40 ml of chloroform, heat under reflux on a water bath for 3 hours, discard the chloroform solution, expel the solvent from the residue. Transfer it with the filter paper tube into a 100 ml conical flask. Accurately add 50 ml of *n*-butanol saturated with water, tightly stopper, allow to stand overnight, ultrasonicate (power, 250 W; frequency, 50 kHz) for 30 minutes and filter. Evaporate accurately 25 ml of the successive filtrate to dryness in an evaporating dish, dissolve the residue in methanol, transfer to a 5 ml volumetric flask, dilute with methanol to volume and mix well.

Procedure Inject accurately 10 μl of the reference solution and 10-20 μl of the test solution, respectively, into the column, and calculate the content.
It contains not less than 0.30 per cent of the total amount of gensenoside Rg_1 ($C_{42}H_{72}O_{14}$) and ginsenoside Re ($C_{48}H_{82}O_{18}$), and not less than 0.20 per cent of gensenoside Rb_1 ($C_{54}H_{92}O_{23}$), calculated with reference to the dried drug.

Prepared slices

Processing Soften thoroughly, cut into thin slices, and dry, or pulverize or break to pieces before use.

Property and Flavor Mild warm; sweet and mild bitter.

Meridian tropism Spleen, lung, heart and kidney meridians.

Actions To tonify the original qi greatly, resume pulse and secure collapse, tonify spleen and replenish kidney, engender fluid and nourish blood, tranquilize the mind and replenish wisdom.

Indications Being just going to collapse caused by body deficiency, cold limbs and faint pulse, low appetite caused by spleen deficiency, dyspnea and cough caused by lung deficiency, thirsty caused by fluid damage, interior heat and wasting-thirst, deficiency of qi and blood, frail caused by long-term illness, fright palpitations and insomnia, impotence and uterine coldness.

Administration and dosage 3-9 g. Decocted separately and added into decoction; or ground into powder for oral administration. 2 g per time, twice a day.

Precautions and Warnings Incompatible with Veratri Nigri Radix et Rhizoma, Trogopterori Faeces.

Storage Preserve in a well closed container, stored in a cool and dry place, protected from moth.

Ginseng Radix et Rhizoma Rubra (红参, Hongshen)

Red Ginseng

Red Gingseng is the steamed and dried root of the cultivar of *Panax ginseng* C. A. Mey. (Fam. Ara-liaceae). The drug is collected in autumn, washed clean, steamed, and dried.

Description Main roots fusiform, cylindrical or flat squared columnar, 3-10 cm long, 1-2 cm in diameter. Externally translucent, reddish-brown, occasionally exhibiting a few dark yellowish-brown patches, furrowed longitudinally, wrinkled and with rootlet scars; the upper part sometimes exhibiting interrupted indistinct annulations, the lower part bearing 2-3 twisted and intersected branch roots and curved rootlets or just showing remains of rootlets. Rhizomes 1-2 cm long, showing several depressed-circular stem scars (Luwan), some bearing 1-2 entire or broken adventitious roots (Ding). Texture hard and fragile, fracture even, horny. Odour delicate fragrant and characteristic; taste, sweet and slight bitter.

Identification (1) Carry out the method of test (1) for Identification in the monograph of Radix et Rhizoma Ginseng. It shows the same characteristics except the starch granule.
(2) Carry out the method of test (2) for Identification in the monograph of Ginseng Radix et Rhizoma. It shows the same result.

Water Not more than 12.0 per cent (Appendix IX H, method 1).

Assay Carry out the method for high performance liquid chromatography (Appendix VI D).

Chromatographic system and system suitability Use octadecylsilane bonded silica gel as the stationary phase, acetonitrile as the mobile phase A and water as the mobile phase B, elute in gradient as the following:

Time (min)	Mobile phase A (per cent V/V)	Mobile phase B (per cent V/V)
0-35	19	81
35-55	19→29	81→71
55-70	29	71
70-100	29→40	71→60

As detector a spectrophotometer set at 203 nm. The number of theoretical plates of the column is not less than 6000, calculated with the reference to the peak of ginsenoside Rg_1.

Reference solution Dissolves ginsenosides Rg_1, Re and Rb_1 CRS, accurately weighed, in methanol to produce a mixture containing 0.5, 0.3 and 0.5 mg of each per ml as the reference solution.

Test solution Weigh accurately 1 g of the powder (through No. 4 sieve) to a Soxhlet's extractor, add 40 ml of chloroform, heat under reflux on a water bath for 3 hours, discard the chloroform solution, expel the solvent from the residue. Transfer it with the filter paper tube to a stoppered conical flask, accurately add 50 ml of *n*-butanol saturated with water, tightly stopper, allow to stand overnight,

ultrasonicate (power, 250 W; frequency, 50 kHz) for 30 minutes and filter. Evaporate accurately 25 ml of the successive filtrate to dryness in an evaporating dish, dissolve the residue in methanol, transfer to a 5 ml volumetric flask, dilute with methanol to volume and mix well.

Procedure Inject accurately 10μl of the reference solution and 10-20 μl of the test solution, respectively, into the column, and calculate the content.

It contains not less than 0.25 per cent of the total amount of ginsenoside Rg_1 ($C_{42}H_{72}O_{14}$), ginsenoside Re ($C_{48}H_{82}O_{18}$), and not less than 0.20 per cent of ginsenoside Rb_1 ($C_{54}H_{92}O_{23}$), calculated with reference to the dried drug.

Prepared slice

Processing Soften thoroughly, cut into thin slices, and dry, Break to pieces before use.

Property and Flavor Warm; sweet and mild bitter.

Meridian tropism Spleen, lung, heart and kidney meridians.

Actions To greatly tonify the original qi, regain pulse and secure collapse, tonify qi and control the blood.

Indications Tending to collapse caused by body deficiency, coldness of limbs and faint pulse, qi failing to control the blood, flooding and spotting.

Administration and dosage 3-9 g, decocted alone and mixed with other decoction before taking.

Precautions and Warnings Incompatible with Veratri Nigri Radix et Rhizoma, Trogopterori Faeces.

Storage Preserve in well closed containers, stored in a cool and dry place, protected from moth.

Glechomae Herba (连钱草, Lianqiancao)

Longtube Ground Ivy Herb

Longtube Ground Ivy Herb is the dried aerial part of *Glechoma longituba* (Nakai) Kupr. (Fam. Labiatae). The drug is collected from spring to autumn, removed from foreign matter, and dried in the sun.

Description 10-20 cm long, sparsely pubescent. Stems square, slender and twisted; externally yellowish-green or purplish-red, bearing adventitious roots at nodes; texture fragile, easily broken, fracture often hollowed. Leaves opposite, mostly crumpled, when whole, reniform or subcordate, 1-3 cm long, 1.5-3 cm wide, greyish-green or greenish-brown, margin crenate; petioles slender, 4-7 cm long. Verticillasters axillary, corolla bilabiate, up to 2 cm long. Odour, aromatic on rubbing; taste, slightly bitter.

Identification (1) Powder: Greyish-green., non-glandular hairs multicellular, 1 to several cells frequently shrunken, and some non-glandular hairs unicellular and conical. Glandular scales with a head of 8 cells. Small glandular hairs with an unicellular head and an unicellular stalk. The walls of leaf lower epidermis cells sinuous. Stomata diacytic. The anticlinal walls of leaf upper epidermal cells sinuous, with relatively fine and dense cuticular striations. Spiral vessels and reticulate vessels 20-30 μm in diameter.

(2) To 2.5 g of the powder add 50 ml of 70% methanol, heat under reflux for 1 hour, filter, and evaporate the filtrate to dryness. Macerate the residue successively with 5 ml of petroleum ether (30-60℃) and dichloromethane for 3 minutes, respectively. Discard the petroleum ether and dichloromethane solutions. Dry the residue and dissolve in 5 ml of water, and apply to a column (1.5 cm in inner diameter, 12 cm long) packed with D101 macroporus absorbing resin. Pre-elute with 80 ml of water, discard the water eluate. Re-elute with 150 ml of 55% ethanol, and discard the eluate. Elute with 40 ml of 70% ethanol, and collect the eluate. Evaporate to dryness and dissolve the residue in 2 ml of methanol as the test solution. Prepare a solution with 2.5 g of Glechomae Herba reference drug in the same manner as the reference drug solution. Dissolve lutedin CRS in methanol to produce a solution containing 0.1 mg per ml as the reference solution. Carry out the method for thin layer chromatography (Appendix Ⅵ B), using silica gel G as the coating substance and a mixture of cyclohexane, ethyl acetate and formic acid (8 : 9 : 0.5) as the mobile phase. Apply separately to the plate 2-6 μl of each of the test solution and the reference drug solution and 2 μl of the reference solution. After developing and removal of the plate, dry in air. Spray with a 3% solution of aluminium trichloride in ethanol, heat at 105℃ for several minutes, and examine under ultraviolet light at 365 nm. The fluorescent spots in the chromatogram obtained with the test solution correspond in position and colour to the spots in the chromatograms obtained with the reference drug solution and the reference solution.

Foreign matter Not more than 2 per cent (Appendix Ⅸ A).

Water Not more than 13.0 per cent (Appendix Ⅸ H, method 1).

Total ash Not more than 13.0 per cent (Appendix Ⅸ K).

Acid-insoluble ash Not more than 3.0 per cent (Appendix Ⅸ K).

Extractives Carry out the method for determination of ethanol-soluble extractives (Appendix Ⅹ A, the hot extraction method), using dilute ethanol as the solvent, not less than 25.0 per cent.

Prepared slices

Processing Eliminate foreign matter, wash clean, cut into sections and dry.

In irregular sections. Stems square, externally yellowish-green or purplish-red. Cut sarface frequently hollowed. Leaves opposite, mostly crumpled, greyish-green or greenish-brown. Verticillasters axillary, corolla labiate. Odour, aromatic on rubbing; taste, slightly bitter.

Identification As required for the crude drug.

Water, Total ash, Acid-insoluble ash and Extractives As required for the crude drug.

Property and Flavor Mild cold; pungent and mild bitter.

Meridian tropism Liver, kidney and bladder meridians.

Actions To drain dampness and relieve stranguria, clear heat and remove toxin, dissipate stasis and disperse swelling.

Indications Heat strangury, stone strangury, dampness-heat jaundice, sore, abscess, swelling and pain, injuries from falls and fights.

Administration and dosage 15-30 g. Appropriate amount for topical application, decocted for bathing.

Storage Preserve in a dry place, protected from mould.

the slices of Rahix et Rhizoma Rhei as described under the method for stir-baking with wine (Appendix Ⅱ D) to dryness.

Rhei Radix et Rhizoma (prepared) Stew or steam the pieces of Rhei Radix et Rhizoma as described under the method for stewing or steaming with wine (Appendix Ⅱ D) until the drug darkens thoroughly.

Rhei Radix et Rhizoma (carbonized) Stir-bake the slices of Rhei Radix et Rhizoma as described under the method for carbonizing by stir-baking (Appendix Ⅱ D) until the outer surface is charred and the inner turns to be dark brown.

Property and Flavor Cold; bitter.

Meridian tropism Spleen, stomach, large intestine, liver and pericardium meridians.

Actions To remove accumulation with purgation, clear heat and purge fire, cool the blood and remove toxin, expel stasis to unblock the meridian, drain dampness to abate jaundice.

Indications Accumulation, stagnation and constipation caused by excess heat, hematemesis caused by blood heat, red eyes and swollen throat, swelling abscess, deep-rooted boil and sore, abdominal pain caused by intestinal abscess, blood-stasis amenorrhea, postpartum stasis and obstuction, injuries from falls and fights, dampness-heat dysentery, jaundice and red urine, stranguria, edema; topical application for burn and scald. Wine-fried Rhei Radix et Rhizoma is good at clearing heat toxin in the upper energizer blood aspect, which can be applied for red eyes, swollen throat, painful swollen gum. The purgation power of prepared Rhei Radix et Rhizoma is mild, and it can purge fire and remove toxin to treat sore and ulcer caused by fire-toxin. Charred Rhei Radix et Rhizoma can cool the blood, resolve stasis and stanch bleeding which can be applied to bleeding symptoms caused by blood heat and stasis.

Administration and dosage 3-15g. It should not be decocted long for purgation. Appropriate amount for topical application, ground into powder and applied to the locations of injuries.

Precautions and Warnings Used with cantion for pregnant woman, or woman in menstrual period or lactation.

Storage Preserve in a ventilated and dry place, protected from moth.

Rhodiolae Crenulatae Radix et Rhizoma (红景天, Hongjingtian)

Bigflower Rhodiola Root

Bigflower Rhodiola Root is the dried root and rhizome of *Rhodiola crenulata* (Hook. f. et Thomas.) H. Ohba (Fam. Crassulaceae). The drug is collected in autumn when the scape is withered, removed from coarse bark, washed clean, and dried in the sun.

Description Rhizome cylindrical, stout, slightly curved, few branched, 5-20 cm long, 2.9-4.5 cm in diameter. Externally brown or chocolate brown, coarse, wrinkled, a layer of yellow membranous epidermis with cerise decorative pattern visible when baring the outer epidermis; some old scapes persisted, base bearing triangular or ovate membranous scales; internode irregular, fracture cerise to purplish-red, with a ring; texture light and lax. Tap root cylindrical, stout, about 20 cm long and 1.5 cm in diameter at the upper part, lateral root 10-30 cm long; fracture orange-red or purplish-red, some with cracks. Odour, aromatic; taste, firstly slightly bitter and astringent, then sweet.

Identification (1) Transverse section of root: Cork of 5-8 layers of cells. Phelloderm cells elliptic or subrounded. Stele very broad, numerous vascular bundles arranged in 2-4 rings, outer ring consisting of collateral vascular bundles and relatively large, other 2-3 rings of amphivasal type and gradually smaller toward inside.

Transverse section of rhizome: Two to three rings of cork layer presented in old rhizome and absent in young rhizomes. Cork of several layers of cells, phelloderm cells obliterated. Cortex narrow. Vascular bundles of stele, large and amphicribral, radially arranged in a circle. Vascular tissue in the inner part and outer part of vascular bundles, well developed and showing a opposite arrangement, parenchyma occurring in the centre. Phloem and xylem nearly equal in length, divided by secondary ray into slat-shaped. Cambium distinct. Pith broad, consisting of parenchymatous cells, amphicribral meddulary bundles scattered. Parenchymatous cells containing brown secretion.

(2) Carry out the method for thin layer chromatography (Appendix Ⅵ B), using silica gel G as the coating substance and the lower layer of a mixture of chloroform, methanol, acetone and water (6 : 3 : 1 : 1) as the mobile phase. Apply separately 10 μl of each of the reference solution and the test solution both obtained under *Assay* to the plate. After developing 18 cm and removal of the plate, dry it in air, expose to iodine vapour until the spots clear. The spot in the chromatogram obtained with the test solution corresponds in position and colour to the spot in the chromatogram obtained with the reference solution.

Water Not more than 12.0 per cent (Appendix Ⅸ H, Method 1).

Total ash Not more than 8.0 per cent (Appendix Ⅸ K).

Acid-insoluble ash Not more than 2.0 per cent (Appendix Ⅸ K).

Extractives Carry out the hot extraction method as described under the determination of ethanol-soluble extractives (Appendix Ⅹ A), using 70% ethanol as the solvent, not less than 22.0 per cent.

Assay Carry out the method for high performance liquid chromatography (Appendix Ⅵ D).

Chromatographic system and system suitability Use octadecylsilane bonded silica gel as the stationary phase and a mixture of methanol and water (15 : 85) as the mobile phase. As detector a spectrophotometer set at 275 nm. The number of theoretical plates of the column is not less than 2000, calculated with the reference to the peak of salidroside.

Reference solution Dissolve salidroside CRS, accurately weighed, in methanol to produce a solution containing 0.5 mg per ml as reference solution.

Test solution Weigh accurately 0.5 g of the powder (through No. 3 sieve) to a stopperd conical flask, accurately add 10 ml of methanol and weigh. Ultrasonicate for 30 minutes, cool, weigh again, replenish the loss of the weight with methanol and mix well. Filter and use the successive filtrate as the test solution.

Procedure Inject accurately 10 μl of each of the reference
