# Supplementary material for: Two Sides of the Same Coin for Health: Adaptogenic Botanicals as Nutraceuticals for Nutrition and Pharmaceuticals in Medicine
Source: Pharmaceuticals (Basel). 2025 Sep 8;18(9):1346. doi: 10.3390/ph18091346 (PMC12472958; doi:10.3390/ph18091346)
Supplement: Supplementary file 1 [file pharmaceuticals-18-01346-s001.zip › Supplement S7_Withania _ Indian Herbal Pharmacopea 2002.pdf]

INDIAN HERBAL PHARMACOPOEIA
REVISED NEW EDITION 2002

INDIAN DRUG MANUFACTURERS' ASSOCIATION
102-B, 'A-Wing', Poonam Chambers
Dr. A.B. Road, Worli, Mumbai - 400 018
Tel : 022-2494 4624 / 2497 4308
Fax: 022-2495 0723
E-mail : idma@vsnl.com Website: www.idma-assn.org

WITHANIA SOMNIFERA

Drug consists of dried roots of *Withania somnifera* (Linn.) (Syn. *Physalis somnifera* Linn., *P. flexuosa* Linn. *P. arborescence DC.)*; Fam. Solanaceae. The plant is widely distributed in North-Western India, Bombay, Gujarat, Rajasthan, Madhya Pradesh, Uttar Pradesh, Punjab plains and extends to the mountainous regions of Himachal Pradesh and Jammu.

OTHER NAMES

Sans.	-	Ashvagandha, Ashvakandika, Vajigandha
Beng.,	-	Ashvagandha
Guj.	-	Ghodakun, Asan, Asoda
Hindi	-	Asgandh
Kan.	-	Angarberu, Asvagandhi
Mal.	-	Amukkuram
Mar.	-	Askandha
Tam.	-	Amukkira, Asuvagandhi
Tel.	-	Pennerugadda, Panneru, Pulivendram, Vajigandha

DESCRIPTION

MACROSCOPIC

Roots 20-30 cm long and 6-12 mm in dia. with a few (2 to 3) lateral roots of slightly smaller size; straight, unbranched. Outer surface is buff to grey-yellow with longitudinal wrinkles and in the center soft, solid mass with scattered pores. Odour characteristic; taste bitter and acrid.

MICROSCOPIC[1-5]

The cork cells are isodiametric and non-lignified. Intercellular spaces are present in phloem parenchyma while it is absent in xylem parenchyma. Fibres absent in phloem and present in xylem. Starch grains simple, reniform and oval, normally found in parenchyma of the cortex and vascular region.

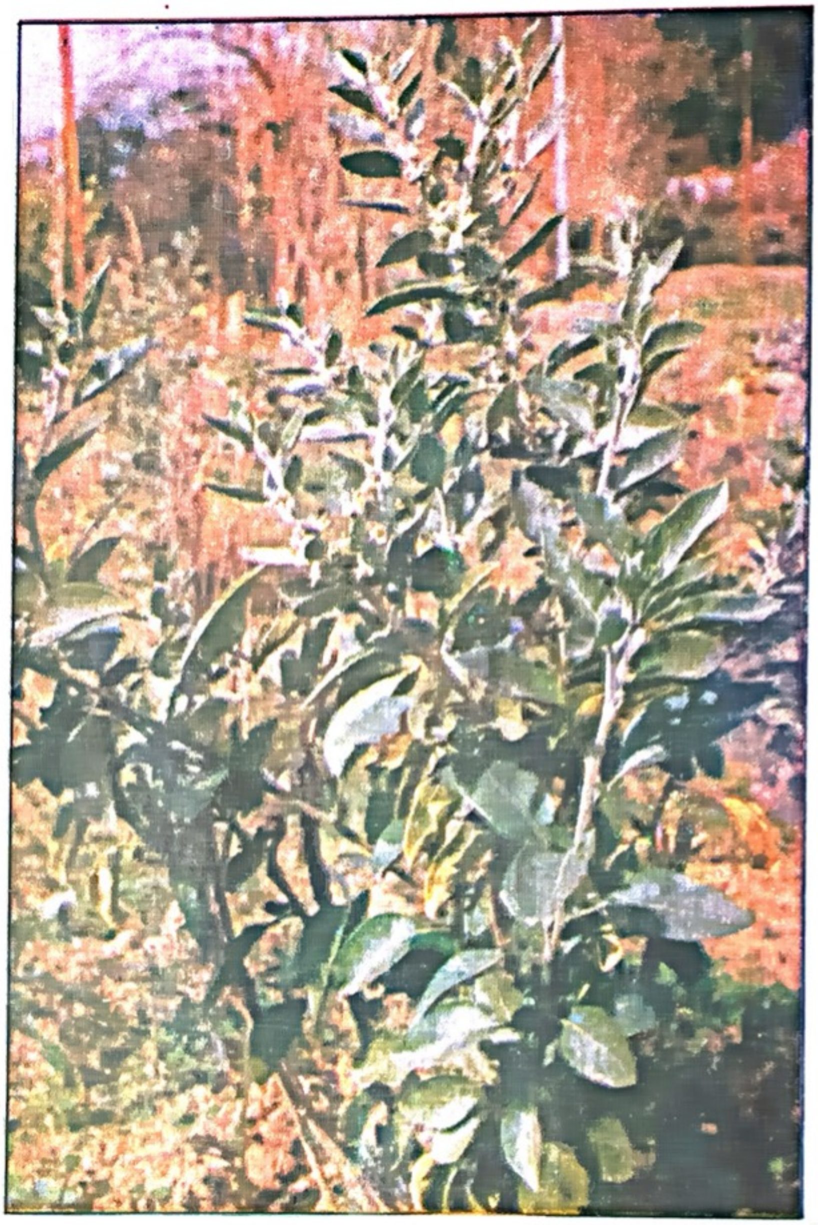

WITHANIA SOMNIFERA

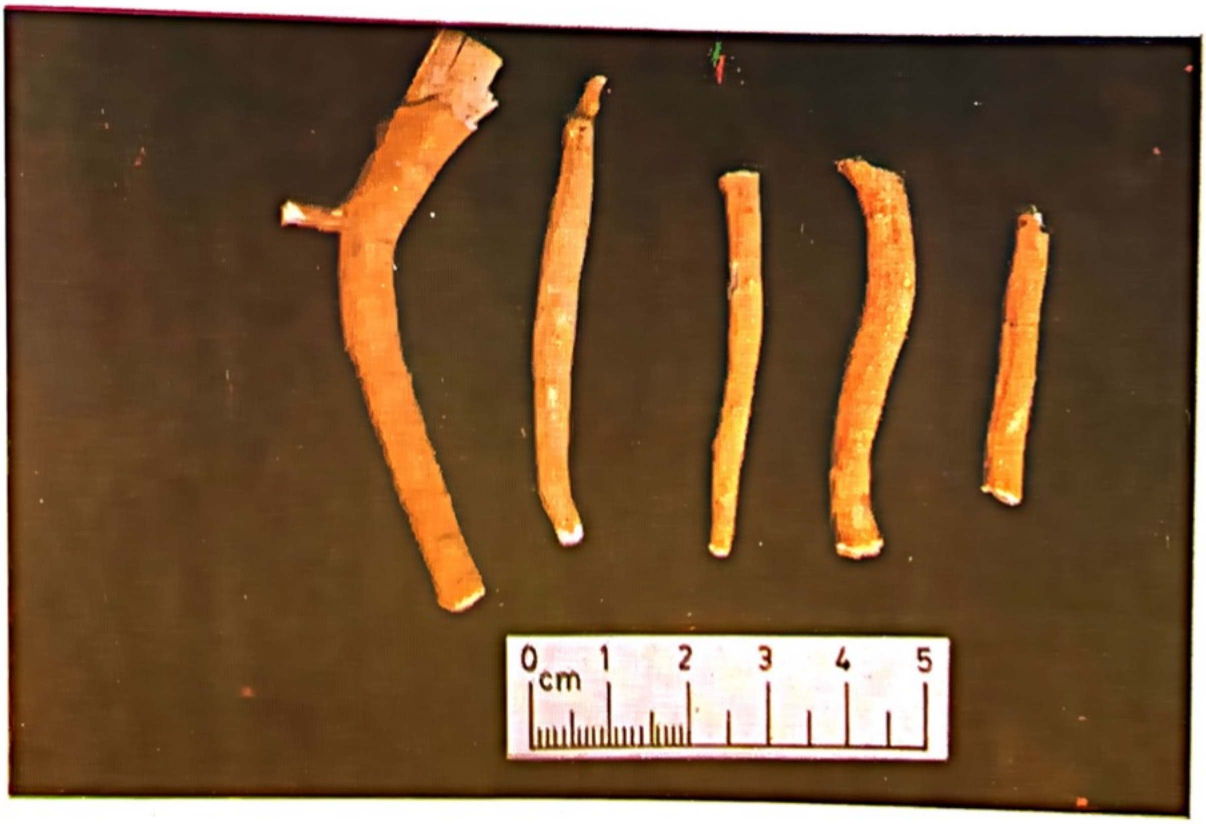

WITHANIA SOMNIFERA : ROOTS

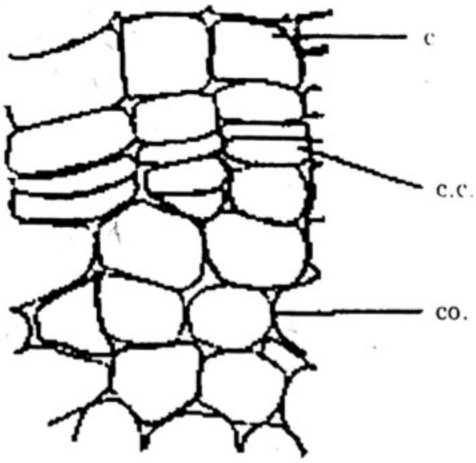

L.S. of root showing cork and cortex x175
c. - cork, c.c. - cork cambium, co. - cortex

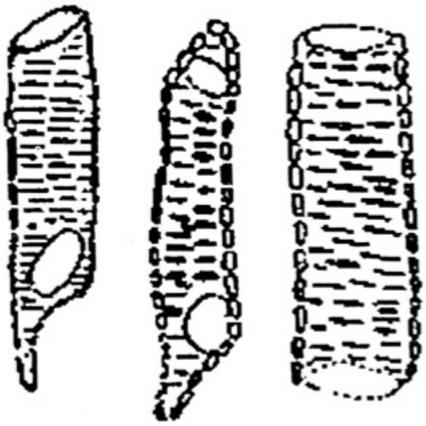
Vessels x240

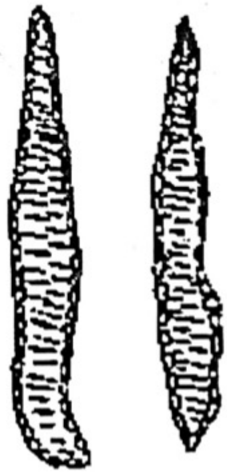
Tracheids x240

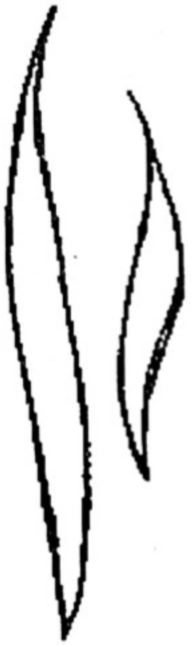
Fibres x240

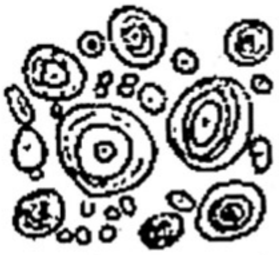
Starch grains x300

CHEMICAL CONSTITUENTS

Majority of the constituents are withanolides (steroidal lactones with ergostane skeleton) and alkaloids. These include Withanone,[6] Withaferin A,[7] Withanolides[8] I, II, III, A, D, E, F, G, H, I, J, K, L, M, WS-I, P and S, withasomidienone,[9] withanolide C,[10] and alkaloids[8] viz., cuscohygrine,

anahygrine, tropine, pseudotropine, anaferine, isopellatierine, 3-tropyltigloate. Total alkaloids about 0.2%

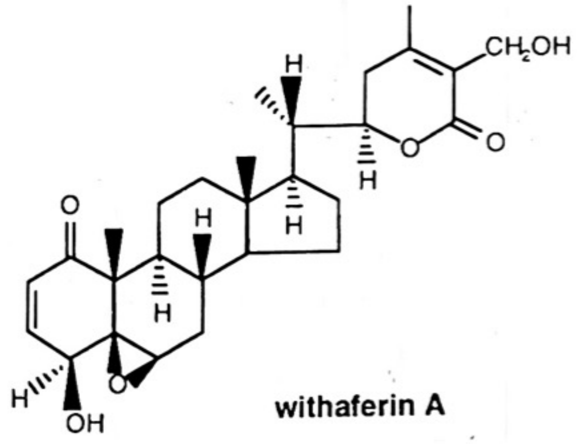

withaferin A

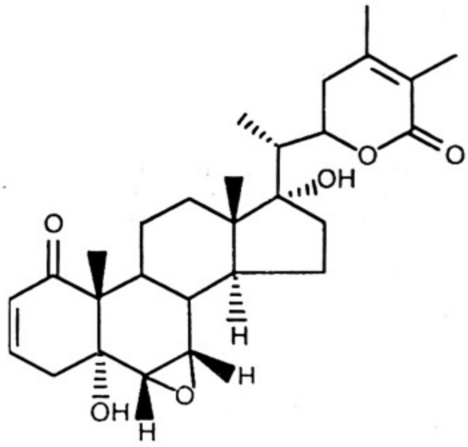

withanone

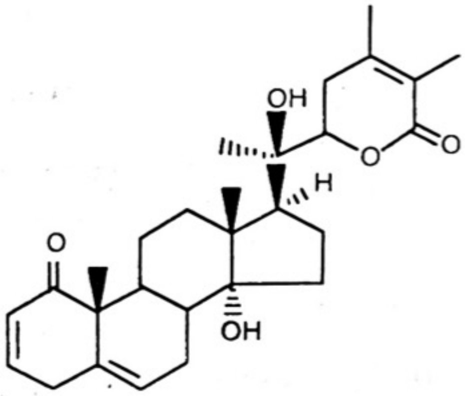

withanolide G

ASSAY / ANALYTICAL METHODS

1. HPLC conditions for the separation of withaferin A in extracts.[11]

Mobile phase	:	n-hexane : isopropanol (9:1)
Flow rate	:	0.2 ml/min.
Column	:	Porasil A coiled column (12 ft. x 1/8 inch)
Detector	:	UV at 225 nm.

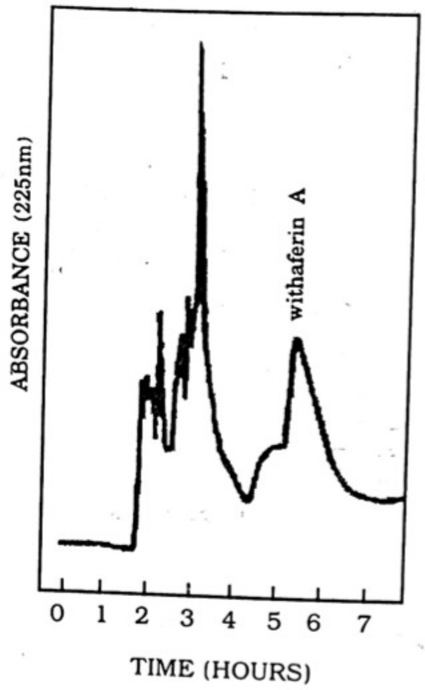

HPLC chromatogram ether extract of *W. somnifera* chemotype I

[Ref. Hunter et al., *J Chromatogr.* 170, 437 (1979)]

2. Analysis of withanolide J in *in vitro* culture by HPLC[12]

Mobile phase	:	Solvent A : water
		Solvent B : acetonitrile
Gradient	:	solvent composition from 25-100% B and flow from 0.38 to 0.8 ml/min in a total period of 51 min.
Column	:	Lichrospher RP-18 (5 mm x 25 cm)
Detection	:	UV at 214 nm and 335 nm.

QUANTITATIVE STANDARDS

Foreign matter	:	Not more than 2.0%
Ash	:	Not more than 7.0%
Acid insoluble ash	:	Not more than 1.2%

Alcohol soluble extractive : Not less than 20.0%

Alcohol (25%) soluble matter : Not less than 16.0%

SUBSTITUTES / ADULTERANTS

Commercial drug consists of the dried roots from cultivation. The roots of wild growing population are not acceptable because of high carbohydrate content and variable composition of alkaloids present and are hence considered as adulterants. The only other major species of *Withania* found in India is W. coagulans Dun. The roots of *W. somnifera* differ from those of *W. coagulans* in having a parenchymatous cortex and simple round starch grains.[14]

PHARMACOLOGY

Roots of the plant show antitumour and radiosensitizing effects in animal models.[15] Total alkaloidal fraction of the root extract exhibits hypotensive, bradycardic and respiratory stimulant activities in dogs. It shows relaxant and antispasmodic effects against several plasmogens on intestinal, uterine, bronchial, tracheal and blood vascular muscles.[8] Withanolides possess remarkable antibacterial, antitumour, antiarthritic and immunosuppressive properties and protective effect against carbon tetrachloride induced toxicity.[16]

FURTHER REPORTED ACTIVITIES

Antistress,[17-19] immunomodulatory,[20-23] anticancer,[24-28] antioxidant,[29,30] anticonvulsive,[31-33] antihelminthic,[34] antiarthritic,[35-37] chemopreventive,[38-41] antibacterial,[42,43] cardioprotective,[44] antidepressant,[45] antitoxic,[46] hypoglycaemic, diuretic and hypocholesterolemic,[47] immunosuppresive,[48] adaptogenic,[49,50] antiradical.[51]

THERAPEUTIC CATEGORY

Adaptogen

DOSAGE

3-6 g of drug in powdered form and 4-8 ml of ashwagandha liquid extract.[13]

REFERENCES

1. *The Ayurvedic Pharmacopoeia of India;* Ministry of Health & Family Welfare, Department of Health, Govt. of India, part I, 1st ed., **1**, 15(1989).
2. Prasad S., Luthra S.P., Gupta P.K., and Bhattacharya L.C., *Indian J. Pharm.* **21**, 189(1959).
3. Shah C.S., Sukkawala V.M., and Vyas L.S., *Indian J. Pharm.* **21**, 195(1959).
4. Datta S.C., and Mukerji B., *Pharmacognosy of Indian Leaf Drugs; Govt. of Indian press, Calcutta,* 101(1952).
5. Aiyer K.N., and Kolammal M., *Pharmacognosy of Ayurvedic drugs; Department of Pharmacognosy* Uni. of Kerala, Trivandrum, Series 1, No 8, 35(1964).
6. Dhalla N.S., Sastry M.S., and Malhotran C.L., *J. Pharm. Sci.* **50**, 876(1961).
7. Mc Phail A.T., and Sim G.A., *J. Chem. Soc.* B., 962(1968).
8. Bhakuni D.S., and Sudha, J., Medicinal and Aromatic Plants in Chadha K.L., and Gupta R (eds.) Advances in Horticulture; Malhotra Publishing House, New Delhi. **11**, 115(1995).
9. Atta ur-Rehman, Abbas A., Shahwar D.E., Jamal S.A., and Choudhary M.I., *J. Nat. Prod.* **56**, 1000(1993).
10. Bessale R., and Lavie D., *Phytochemistry* **31**, 3648(1992).
11. Hunter I.R., Walden M.K., and Heftmann E., *J. Chromatogr.* **170**, 437(1979).
12. Vitali G., Conte L., and Nicoletti M., *Planta Med.* **62**, 287(1996).
13. *Pharmacopoeia of India;* Ministry of Health, Govt. of India, 2nd ed., 74(1966).
14. *Wealth of India (Raw Materials);* Publication and Information Directorate, CSIR, New Delhi,**10**, 581(1976).
15. Devi P.U., Sharada A.C., and Solomon E.E., *Indian J. Expt. Biol.* **31**, 607(1993).
16. Sudhir S., Budhiraja R.D., Miglan G.P., Arora B., Gupta L.C., and Garg N., *Planta Med* **1**, 61 and references cited therein (1986).
17. Archana R. and Namasivayam A., antistressor effect of *Withania somnifora. Journal of Ethnopharmacology* **64**, 91-93, (1999).
18. Kaur Gurpreet and Kulkarni, S.K. Reversal of forced swimming - induced chronic fatigue in mice by antidepressant and herbal psychotropic drugs. *Indian Drugs* **35**, 771-777, (1998).
19. Davis L. and Kuttan G., Suppressive effect of cyclophosphamide - induced toxicity by W. somnifera extract in mice. Journal of Ethnopharmacology, **62**, 209-214, (1998).

20. Dhuley J.N., Effect of some Indian herbs on macrophage functions in ochratoxin A treated mice *Journal of Ethanopharmacology* **58**, 5-20, 1997.

21. Gupta Y.K., Sharma S.S. Rai K., et al. Reversal of paclitaxel induced neutropenia by *Withania somnifera* in mice [In Process Citation]. *Indian J Physiol Pharmacol* (India), **45**, 253-57, (2001).

22. Davis L. and Kuttan G., Immunomodulatory activity of *Withania somnifera J Ethnopharmacol* (Ireland), **71**, 193-200, (2000).

23. Agarwal R., Diwanay S., Patki P., et al. Studies on immunomodulatory activity of *Withania somnifera* (Ashwagandha) extracts in experimental immune inflammation. *J Ethnopharmacol* (Ireland), **67**, 27-35, (1999).

24. Kulkarni A., A ray of hope for cancer patients. Proceedings of International Seminar on Holistic management of cancer & Ayurveda Education Series No.64, 5 -11, (1998).

25. Udupa N., A Battle against cancer with pharmaceutical weapon, *Phytochemistry*, **45**, (1997).

26. Sheena I.P., Singh U.V., Kamath R.U. and Udupa N., Niosomal withaferin A with better antitumor efficacy *Indian Journal of Pharmaceutical science*. **60**, 45-48, (1997).

27. Uma Devi P., Kamath R. and Rao B.S., Radiosenitization of a mouse melanoma by withaferin A., Invivo studies. *Indian Journal of Experimental Biology*. 38, 432-437, (2000).

28. Davis L. and Kuttan G., Effect of *Withania somnifera* on DMBA induced carcinogenesis. *J Ethnopharmacol* (Ireland), **75**, 165-68, (2001).

29. Panda S. and Kar A., Evidence for free radical scavenging activity of Ashwagandha root powder in mice. *Indian Journal of Physiology & Pharmacology* **41**, 424-426, (1997).

30. Mohanty I., Joshi S., Adhikary N. and Gupta S.K., Antioxidant activity of W. somnifera on selenite induced oxidative damage lenses (XXXI Annual conference of Indian Pharmacological society, Lucknow, Dec 18-20, 1998). *Indian Journal of Pharmacology* **31**, 75, (1999).

31. Kulkarni S.K., George B. and Mathur R., Neuroprotection by *Withania somnifera* root extract against lithium-pilocarpine-induced seizures. *Indian Drugs*. **35**, 208-215, (1998).

32. Ashoka D.B. and Vaidya The status and scope of Indian medicinal plants acting on CNS. *Indian Journal of Pharmacology* **29**, 340-343, (1997).

33. Jain S., Shukla S.D., Sharma K., et al. Neuroprotective Effects of *Withania somnifera* Dunn. in Hippocampal Sub-regions of Female Albino Rat [In Process Citation] *Phytother Res* (England), **15**, 544-48, (Sep. 2001).

34. Qamar F., Kalhoro M.A. and Badar U., Antihelminthic properties of some indigenous plants. *Hamdard Medicus* **41**, 115-117, (1998).

35. Bikshapathi T. and Krishna K., Clinical evaluation of Ashwagandha in the management of Amavala *Journal of Research in Ayurveda & Siddha* **20**, 46-53, (1999).

36. Madhavikutty P., Santhakumari K., Vijayan N.P. and Nair S.K., Comparative clinical study on must, Ashwagandha, Panchakarma therapy in Amavala (rheumaloid arthritis). *Journal of Research in Ayurveda & Siddha* **18**, 1-10, (1997).

37. Joshi Y.K., A synergistic formulation for the treatment of rhematic diseases, South Africa, ZA 00908, (1995).

38. Aphale A.A., et al., Subacute toxicity study of the combination of ginseng (Panax ginseng) and ashwagandha *Withania somnifera*) in rats: a safety assessment *Indian J Physiol Pharmacol*, **42**, 299-302, (1998).

39. Davis L. and Kuttan G., Suppressive effect of cyclophosphamide-induced toxicity by *Withania somnifera* extract in mice. *J Ethnopharmacol* (Ireland), **62**, 209-14, (1998).

40. Prakash J., et al. Chemopreventive activity of *Withania somnifera* in experimentally induced fibrosarcoma tumours in Swiss albino mice. *Phytother Res* (England), **15**, 240-44, (2001).

41. Shukla S.D., et al., Stress induced neuron degeneration and protective effects of Semecarpus anacardium Linn. and *Withania somnifera* Dunn. in hippocampus of albino rats: an ultrastructural study. *Indian J Exp Biol* (India), **38**, 1007-13, (2000).

42. Furmanowa N., Gajdzis-Kuls D., Starosciak B. and Stefarska J., Antibacterial activity of W. somnifera (L.) Dun. organs cultivated in vitro. *Herbs Polonica* **44**, 265-269, (1998).

43. Ali N.A., et al. Screening of Yemeni medicinal plants for antibacterial and cytotoxic activities. *J Ethnopharmacol* (Ireland) **74**, 173-79, (2001).

44. Dhuley J.N., Adaptogenic and cardioprotective action of ashwagandha in rats and frogs. *J Ethnopharmacol* (Ireland), **70**, 57-63, (2000).

45. Bhattacharya S.K., et al. Anxiolytic-antidepressant activity of *Withania somnifera* glycowithanolides. an experimental study. *Phytomedicine* (Germany), **7**, 463-69, (2000).

46. Chaurasia S.S., Panda S. and Kar A., *Withania somnifera* root extract in the regulation of lead-induced oxidative damage in male mouse. *Pharmacol* Res (England), **41**, 663-66, (2000).

47. Anadallu B. and Radhika B., Hypoglycemic, diuetic and hypochloesterolemic effect of Winter (*W. Somnifera*, Dunal) root. *Indian Journal of Experimental Biology* **38**, 607-609, (2000).

48. Furmanowa M., et al., In vitro propagation of *Withania somnifera* and isolation of withanolides with immunosuppressive activity. *Planta Med* (Germany), **67**, 146-49, (2001).

49. Bhattacharya S.K., Bhattacharya A. and Chakarbarti A., Adaptogenic activity of siotone, a polyherbal formulation of Ayurvedic rasayanas. *Indian Journal of Experimental Biology* **38**, 119-128, (2000).

50. Singh. B.; Gupta, D.K. and Chandan, B.K. Adaptogenic activity of glyco-peptidolipid fraction from the alcoholic extract of Trihopus zeylanicum Gaerta. *Phytomedicine,* b.8, 283-291, (2001).

51. Russo, A.; Izzo, A.A.; Cardile, V.;Borrelli, F. and Vanella, A. Indian medicinal plants as antiradicals and DNA cleavage protectors. *Phytomedicine,* **8**, 125-132, (2001).

ADDITIONAL REFERENCES

- Kulkarni R., The effects of Indian Medicinal Plants on experimentally induced convulsions in mice. M.D. dissertation in Pharmacology, University of Mumbai (1996).
- Malhotra C.L., Das P.K. and Dhalla N.S., Studies on Withania ashwagandha Kaul (Part I): Effect of total extract on the CNS and smooth muscle. *Indian J. Physiology & Pharmacology* **4**, 35 (1960).
- Malhotra C.L., Das P.K. and Dhalla N.S., Studies on Withania ashwagandha Kaul (Part II): Effect of total extract on the CNS, respiration and skeletal muscle. *Indian J. Physiology & Pharmacology* **4**, 49 (1960a).
- Malhotra C.L., Mehta V.L., Das P.K. and Dhalla N.S., Studies on Withania ashwagandha Kaul (Part V): The effect of total alkoloids (ashwagandholine) on the CNS. *Indian J. Physiology & Pharmacology* **9**, 127 (1965).
- Prasad S. and Malhotra C.L. Studies on Withania ashwagandha Kaul (Part VI) : Effect of the alkaloidal fractions (acetone, alcohol & water soluble) on the CNS *Indian J. Physol & Pharmacology* **12**, 175, (1968).
- Singh R.H., Malviya P.C., Sarkar H and Udupa K.N., Studies on the psychotropic effect of Indian indigenous drug, Ashwagandha (Withania somnifera Dunol) Part II experimental studies *J Res Ind. Med. Yoga & Hom,* **14**, 49, (1979).
- Budiraja R.D. and Sudhir S., Review of the biological activity of Withanolides *J. Scientific & Industrial Research* **46**, 488 (1987).
- Mehta A.K., Binkley P., Gandhi S.S. and Ticku M.K., Pharmacological effects of Withania somnifera root extract on GABA receptor complex. *Indian J. Medical Research* **94**, 312 (1991).
- Kulkarni S.K., Sharma A., Verma A. and Ticku M.K., GABA receptor medicated anticonvulsant action of Withania somnifera root extract *Indian Drugs* **30**, 305, (1993).
- Gandhi A., Muzumdar A.M. and Patwardhan B., A comparative pharmacological investigation of Ashwagandha & Ginseng. *J. Ethnopharmacology* **44**, 131, (1994).
- Bhattacharya S.K., Kumar A. and Ghosal S., Effects of glycowithanolides from Withania somnifera on an animal model of Alzheimer's disease and perturbed central cholinergic markers of cognition in rats. *Phytotherapy Research* **9**, 110, (1995).
- Kulkarni S.K. and George B., Anticonvulsant action of *Withania somnifera* (Ashwagandha) root extract against pentylene tetrazol induced kindling in mice *Phytotherapy Research* **10**, 447, (1996).

- Ziauddin M., Phansalka N., Patki P., Diwanay S. and Patwardhan B., Studies on the immunomodulatory effect of Ashwagandha. *J. Ethnopharmacol* **50**, 69 (1996).
- Ghosal S., et al, Immunomodulatory and CNS effects of sitomindosides IX and X, Two new glycowithanolides from *W somnifera*, *Phytotherapy Res.* **3**, 5, (1989).
- Singh N. et al, *Withania somnifera* a rejuvnating herbal drug which enhances survival during stress, N Singh, et al. *Ind J Crude Drugs Res* **20**, 1, 29-35, (1982).
- Singh R.H. and Malaviya P. C., Studies on the Psychotropic effect of an Indigenous Rasayan Drug Asvagandha (*W. sommifera*) Part I, Clinical study, *J Res Ind Med Yoga Hom*, **13**, 1, (1978).
- Ahummuda F, Aspee F., Wikman G., and Hancke J. *Withania somnifera* Extract, Its effect on arterial blood pressure in anesthetised dogs, *Phytotherapy Res* **5**, 111-114, (1991).
- Dwivedi S., Gupta D., and Sharma K.K., Modification of coronary risk factors by medicinal & aromatic plants. *Journal of Medicinal & Aromatic Plant Sciences*. **21**, (Suppl. 1), 41, (1999).
- Mathur A.K., Lad S., and Parikh D., Preliminary report on the role of the Y-Spur capsules in male infertility. Antiseptic. **96**, 301-302, (1999).
- Kulkarni S.K., George B. and Mathur R., Protective effect of *W. somnifera* root extract on electrographic activity in a lithium pilocarpine model of status epilepticus *Phytotherapy Research*, **12**, 451-453, (1998).
- Bose S. and Gupta Y.K., Effect of CNS active herbal drugs on swim test in mice. *Indian J. of Pharmacology*. **31**, 75, (1999).
- Al-Quarawi, A.A. Abdel-Rahman H.A., El-Bardry A-A., Harraz F., Razing N.A. and Adbel-Magied E.M., The effect of extract of *Cynomonium coccineum* & *W. somnifera* on gonadotropiuns & ovarian follicles of immature wistar rats. *Phytotherapy Research* **14**, 228-290, (2000).
- Singh A., Saxena E. and Bhutani K.K., Adrinocorticosterone alterations in male albinomice treated with *Trichopus zeylanicus*, *W. somnifera* & *Panax ginseng* preparations. Phytotherapy Research **14**, 122-125, (2000).
- Chauhan K.R. and Gaikwad B.Y., Clinical trial of Sensa forte in the management of sexual dysfunction & premature ejaculation. *Antiseptic*. **95**, 43-44, (1998).
- Dwivedi S.C. and Mathur B., Screening of plant extracts as repellent against *spodoptera litura (Lipidopten noctuidae)* Indian Biologists, **320**, 71-73, 2000.Vaishamya - A clinical study. *Journal of Research in Ayurveda & Siddha* **20**, 148-157, (1999).
- Dhuley J.N., Noo tropic-like effect of ashwagandha (*Withania somnifera* L.) in mice [In Process Citation] *Phytother Res* (England), **15**, 524-28, (2001).
- Kulkarni S.K. and Ninan I., Inhibition of morphine tolerance and dependence by *Withania somnifora, J. of Ethnopharmacology* **57**, 213-217, (1997).
- Asthana R. and Raina M.K., Pharmacology of *Withania somnifera* - A Review, *Indian Drugs*, 26(5), 199-205, (1989).
